# PhenTAA: A Redox-Active N_4_-Macrocyclic Ligand Featuring Donor and Acceptor Moieties

**DOI:** 10.1021/acs.inorgchem.3c03708

**Published:** 2024-01-12

**Authors:** Roel F. J. Epping, Felix J. de Zwart, Nicolaas P. van Leest, Jarl Ivar van der Vlugt, Maxime A. Siegler, Simon Mathew, Joost N. H. Reek, Bas de Bruin

**Affiliations:** † Homogeneous, Supramolecular Catalysis and Bio-Inspired Catalysis Group, van ’t Hoff Institute for Molecular Sciences (HIMS), University of Amsterdam, Science Park 904, 1098 XH Amsterdam, The Netherlands; ‡ Department of Chemistry, Johns Hopkins University, Baltimore, Maryland 21218, United States

## Abstract

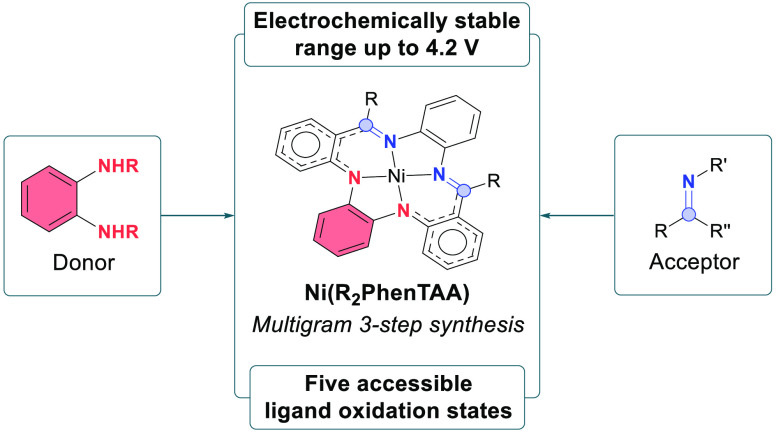

Here, we present the development and characterization of the novel PhenTAA macrocycle as well as a series of [Ni(R_2_PhenTAA)]^n^ complexes featuring two sites for ligand-centered redox-activity. These differ in the substituent R (R = H, Me, or Ph) and overall charge of the complex n (n = −2, −1, 0, +1, or +2). Electrochemical and spectroscopic techniques (CV, UV/vis–SEC, X-band EPR) reveal that all redox events of the [Ni(R_2_PhenTAA)] complexes are ligand-based, with accessible ligand charges of −2, −1, 0, +1, and +2. The *o*-phenylenediamide (OPD) group functions as the electron donor, while the imine moieties act as electron acceptors. The flanking *o*-aminobenzaldimine groups delocalize spin density in both the oxidized and reduced ligand states. The reduced complexes have different stabilities depending on the substituent R. For R = H, dimerization occurs upon reduction, whereas for R = Me/Ph, the reduced imine groups are stabilized. This also gives electrochemical access to a [Ni(R_2_PhenTAA)]^2–^ species. DFT and TD–DFT calculations corroborate these findings and further illustrate the unique donor–acceptor properties of the respective OPD and imine moieties. The novel [Ni(R_2_PhenTAA)] complexes exhibit up to five different ligand-based oxidation states and are electrochemically stable in a range from −2.4 to +1.8 V for the Me/Ph complexes (vs Fc/Fc^+^).

## Abstract 2

Redox-active ligands have seen a significant rise in interest in recent years due to their synthetic tunability of redox properties. Here, the combination of established donor and acceptor moieties has led to the development of a new redox-active ligand, PhenTAA (or tetra**phen**ylene**t**etra**a**za[14]**a**nnulene). Nickel(II)-derived PhenTAA complexes are shown to exhibit five different ligand-based oxidation states as a result of these donor-acceptor properties and are electrochemically stable up to 4.2 V.

## Introduction

The release and storage of electrons are intrinsic to many metalloenzymatic transformations.^1^ The prevalence of first row transition metals in such enzymes is often accompanied by redox-active ligands to impart multielectron reactivity.^2^ Inspired by nature, redox-active ligands have gained a significant foothold in inorganic chemistry,^3^ catalysis,^3^ and material sciences.^4^ Among numerous applications, the ability and application of redox-active ligands to function as redox shuttles have allowed the synthesis of first-row transition metal complexes featuring redox-active ligands to exhibit “noble-metal like” reactivity.^5^ The unique redox properties of these ligands, coupled with their high degree of synthetic versatility, allow for unexplored stoichiometric and catalytic reactivities.^3e^ Consequently, the development of new redox-active scaffolds receives considerable interest. Popular motifs are *ortho*-substituted heteroaromatic systems^5^ (i.e., catechol, aminophenol, or *o*-phenylenediamine) and delocalized π-systems (i.e., diimines).^6^ Metal complexes bearing such building blocks can exhibit several ligand-based redox events, originating from the gain (or loss) of an electron onto (or from) the heteroaromatic system.

**Figure 1:**
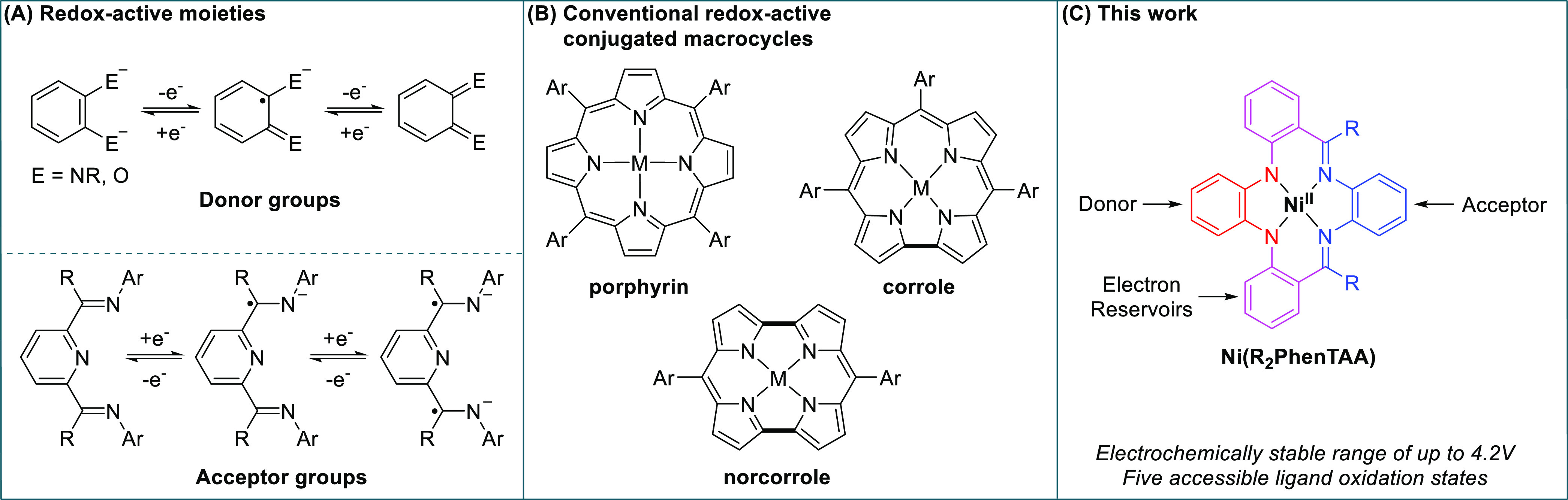
Examples of popular redox-active donor and acceptor groups and their respective oxidation states (A). Prominent members of the porphyrinoid family that exhibit ligand-based redox activity, dependent on their global (anti)aromatic conjugated nature (B). Novel redox-active scaffold (R_2_PhenTAA) based on the sum of popular redox-active donor/acceptor moieties (this work) (C).

For instance, *o*-phenylenediamine initially experiences a 1e^–^ oxidation to the radical *o*-diiminosemiquinone, followed by another 1e^–^ oxidation to the fully oxidized benzoiminoquinone (Figure 1A). These *ortho*-substituted heteroaromatic systems are often employed as dianionic donor moieties, whereas π-systems are used as acceptor sites (Figure 1A).^5,6^ While examples of such motifs in multidentate ligands are abundant, examples of conjugated, redox-active macrocycles are not, despite the coordinating power imparted by the macrocyclic effect.^7^ This phenomenon enhances the stability of metal complexes,^7^ with the macrocyclic conjugation further stabilizing the buildup of charge and/or spin density.^8^ Nevertheless, a few examples of macrocyclic redox-active ligands have been reported thus far. A prominent group of conjugated macrocyclic ligands is the porphyrinoid family, consisting primarily of aromatic porphyrins^9^ and corroles,^10^ alongside antiaromatic norcorrole^11^ ligands (Figure 1B). Electrochemical investigations revealed that these porphyrinoid ligands can access both multiply reduced and oxidized forms, especially for the antiaromatic norcorrole.^11^ However, the structural dependency of global (anti)aromaticity for these macrocycles also limits their synthetic versatility. Therefore, we were interested in developing a conjugated macrocyclic ligand platform that derives its redox-active capability from independent moieties, in order to deviate from the reliance on a global (anti)aromatic electronic structure. The combination of independent redox-active groups within an overall conjugated macrocyclic structure should give rise to a unique electronic structure that features greater synthetic flexibility. We envision that the development of such a versatile platform can contribute to new modes of reactivity in catalysis,^3^ development of functional materials (i.e., redox-flow batteries^12^), and replacement of expensive noble-metal driven processes.^5^ Based on the aforementioned *ortho*-substituted heteroaromatic systems as electron donors and conjugated π-systems as electron acceptors, we report the synthesis of the novel conjugated macrocyclic tetra**phen**ylene[b,e,i,m][1,4,8,11]**t**etra**a**za[14]**a**nnulene, *H*_*2*_(R_2_PhenTAA), featuring a diaryl-*o*-phenylenediamine donor and an *o*-diiminophenylene acceptor moiety (Figure 1C). Nickel was employed as the first-row transition metal of choice, as the anticipated square planar Ni(II)-complexes are bereft of (readily accessible) metal-centered redox events.^13^ Additionally, the R-group at the imino group C-terminus was varied to probe the redox properties and/or stability of these Ni complexes. Through a combination of spectroscopic and electrochemical techniques (CV, spectro-electrochemical UV/vis, EPR) and computational methods (DFT), we characterized the redox properties of the various [Ni(R_2_PhenTAA)] complexes and determined whether redox events are either metal- or ligand-centered.

## Results and Discussion

### Synthesis of Ligands and Nickel Complexes

**Scheme 1:**
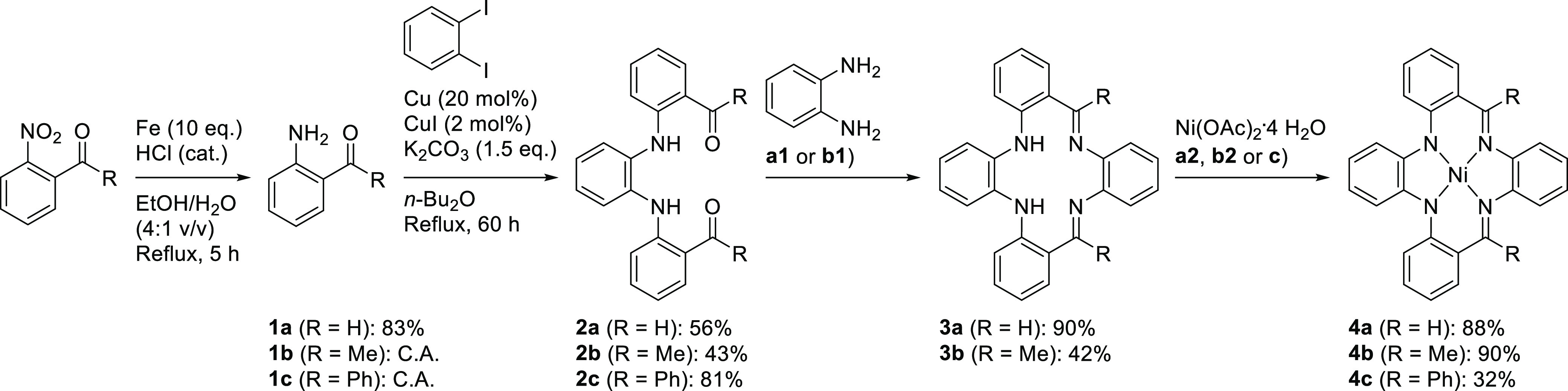
Synthesis of [Ni(R_2_PhenTAA)] Complexes Conditions diverged from **2** onwards. a) For **2a** (condition a1) (1 equiv), Zn(OAc)_2_·2H_2_O (1 equiv), *o*-phenylenediamine (1 equiv), MeOH, reflux, overnight. b) For **2b** (condition b1) (1 equiv), *o* -phenylenediamine (1.1 equiv), NaHCO_3_ (1 equiv), 3 Å MS, toluene, 110 °C, 10 d. c) For **2c** (condition c) (1 equiv), *o*-phenylenediamine (1 equiv), Ni(OAc)_2_·4H_2_O (1 equiv), toluene, reflux (Dean-Stark), 7 d. d) For **3a-b** (condition a2/b2) (1 equiv), Ni(OAc)_2_·4H_2_O (2 equiv), DMF, reflux, overnight. C.A. = commercially available.

[Ni(R_2_PhenTAA)] complexes were synthesized via a gram-scalable divergent synthetic approach (Scheme 1). For the archetypical R = H, the starting point was the commercially available *o*-nitrobenzaldehyde, which was reduced to *o*-aminobenzaldehyde (**1a**) with iron powder according to a literature procedure.^14^ To prevent self-condensation, **1a** was used immediately in the next step. Compounds **2a**–**c** (R = H, Me, Ph, respectively) were synthesized using an Ullmann–Goldberg cross-coupling reaction between *o*-diiodobenzene and **1a**–**c** (**1b**–**c** are commercially available starting materials) via a modified literature procedure.^15^ Condensation of *o*-phenylenediamine with dicarbonyl synthons **2a**–**c** required a different route, depending on the R-group desired. For R = H, condensation of **2a** with *o*-phenylenediamine in combination with a stoichiometric amount of Zn(OAc)_2_·2H_2_O as a Lewis acid afforded [H_2_(H_2_PhenTAA)] (**3a**, 90% yield) in 90% yield as a bright orange-red precipitate. For R = Me, the condensation of **2b** with *o*-phenylenediamine was achieved via reflux in toluene using 3 Å molecular sieves and NaHCO_3_ as a mild base to afford [H_2_[Me_2_PhenTAA)] (**3b**) in 42% yield. Compounds **4a**–**b** were synthesized via metalation of free-base ligands **3a**–**b** with Ni(OAc)_2_·4H_2_O at reflux in DMF, with subsequent crystallization/precipitation affording complexes **4a** and **4b** in 88% and 90% yields, respectively. All attempts to obtain **3c** proved unsuccessful, rationalized by the steric bulk imparted by the additional phenyl group in the benzophenone moiety. Therefore, a direct route toward [Ni(Ph_2_PhenTAA)] was employed, using nickel as a template for square-planar complex formation.^16^ Subsequently, compound **2c**, *o*-phenylenediamine, and Ni(OAc)_2_·4H_2_O were heated at reflux for 7 days in xylene using a Dean–Stark apparatus. [Ni(Ph_2_PhenTAA)] (**4c**) was isolated in 32% yield after purification.

### Characterization of the Free Base Ligands

The new compounds were characterized by ^1^H-,^13^C-, and 2D–NMR spectroscopy, HR–ESI–MS (positive mode), and UV/vis spectroscopy. Additionally, **3a** was analyzed by in situ ATR–FT–IR and melting point analysis (see Supporting Information, page 8).

**Figure 2:**
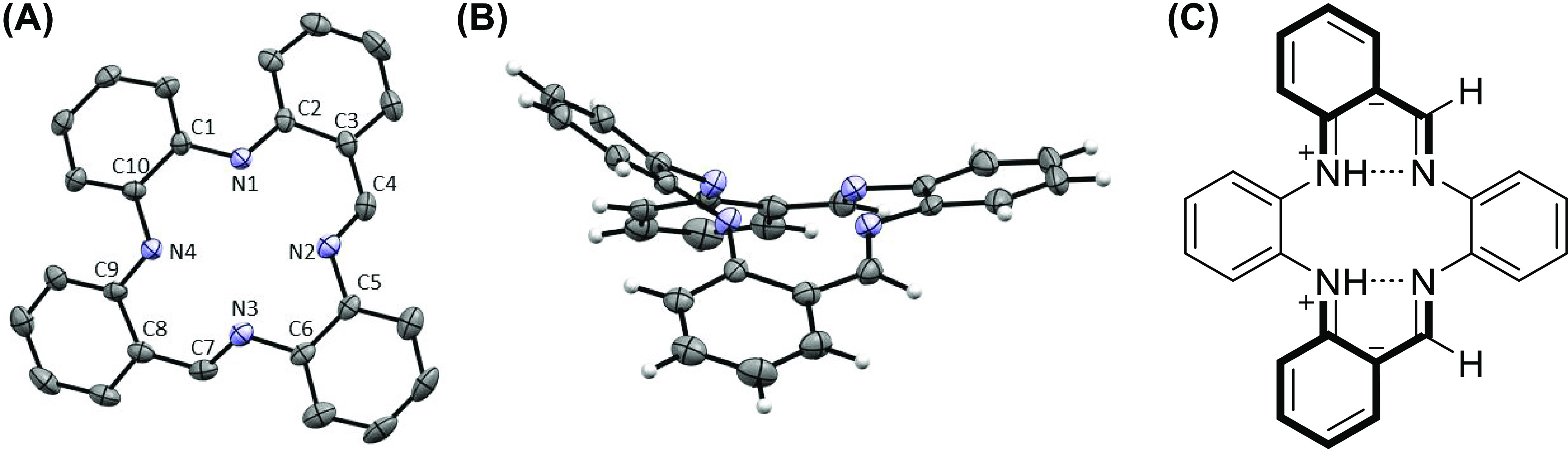
Displacement ellipsoid plots (50% probability) of *H*_*2*_(H_2_PhenTAA) (**3a**) front- (A) and side-views (B). N*H* (A and B) and C*H* (A) protons were omitted. Selected bond lengths (Å): C1–N1 1.409(4), N1–C2 1.369(4), C2–C3 1.435(4), C3–C4 1.434(4), C4–N2 1.284(4), N2–C5 1.412(4), C5–C6 1.402(4), C6–N3 1.412(4), N3–C7 1.284(4), C7–C8 1.434(4), C8–C9 1.435(4), C9–N4 1.369(4), N4–C10 1.409(4), C10–C1 1.427(4). Resonance structure of free-base **3a** that contributes significantly to the overall electronic structure (C).

Single crystal X-ray diffraction (SC–XRD) studies (Figure 2A and B) showed that these macrocyclic structures have a distinct saddle-shaped geometry, akin to the tetramethyltetra[14]azaannulene macrocycle.^17^ Bond length analysis revealed a distinct deviation from the reference standard benzene for the *o*-aminobenzaldimine phenyl rings 2 and 3 (Table S2, Supporting Information). The C–N and C_Ar_–C_imine_ bonds have significant double-bond character, whereas the adjacent C_Ar_–C_Ar_ are lengthened with respect to benzene. This suggests significant delocalization across the *o*-aminobenzaldimine moiety (Figure 2C).

### Characterization of the Nickel Complexes

**Figure 3:**
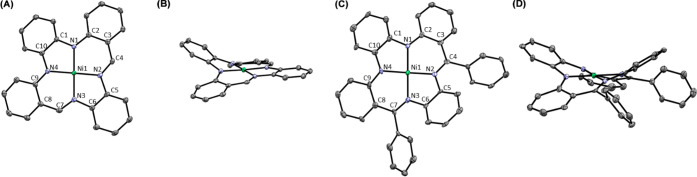
Displacement ellipsoid plots (50% probability) of [Ni(H_2_PhenTAA)] (**4a**) top-view (A) and side-view (B) and [Ni(Ph_2_PhenTAA)] (**4c**) top view (C) and side view (D). Selected bond lengths for **4a** (Å): C1–N1 1.406(2), N1–C2 1.361(2), C2–C3 1.428(2), C3–C4 1.417(2), C4–N2 1.304(2), N2–C5 1.424(2), C5–C6 1.395(2), C6–N3 1.424(2), N3–C7 1.304(2), C7–C8 1.416(2), C8–C9 1.427(2), C9–N4 1.361(2), N4–C10 1.402(2), C10–C1 1.411(2), N1–Ni1 1.864(1), N2–Ni1 1.860(1), N3–Ni1 1.863(1), N4–Ni1 1.865(1). Selected bond lengths for **4c** (Å): C1–N1 1.402(3), N1–C2 1.362(3), C2–C3 1.427(3), C3–C4 1.457(4), C4–N2 1.322(3), N2–C5 1.431(3), C5–C6 1.410(4), C6–N3 1.427(3), N3–C7 1.329(3), C7–C8 1.446(3), C8–C9 1.433(4), C9–N4 1.356(4), N4–C10 1.405(4), C10–C1 1.416(4), N1–Ni1 1.842(2), N2–Ni1 1.874(2), N3–Ni1 1.867(2), N4–Ni1 1.841(2).

Upon metalation of **3a**, the distinct signal of the NH hydrogens at δ = 12.20 ppm in ^1^H NMR disappears, indicative of successful metalation. The imine (δ = 8.04 vs 8.73 ppm for **4a** vs **3a**, respectively) and Ar–H signals (see Supporting Information, pages 60 and 65) experience a significant upfield shift. 2D-NMR and mass spectrometry further support the composition of the expected complex. The diamagnetic nature of the complex (as per its NMR spectra) indicates that the complex does not possess an open-shell character, congruent with a square planar d^8^ geometry.^18^ UV/vis spectroscopy on the intensely dark-purple complex revealed the appearance of two new broad visible wavelength absorptions at 497 and 627 nm. The UV absorption at 282 nm of **3a** experienced a red-shift to 301 nm for **4a**, with the bands at 307 nm and 394 nm disappearing completely for **4a**. In situ ATR–FT–IR spectroscopy on the complex (suspended in C_6_H_6_ solution) reveals a shift of the characteristic C=N and *o*-aminobenzaldimine stretching modes between **3a** (1587 and 1446 cm^–1^) and **4a** (1573 and 1457 cm^–1^). Notably, the disappearance of the free N–H stretching band at 1638 cm^–1^ is consistent with metalation. SC–XRD confirmed the expected square-planar geometry of **4a**, adopting a half-saddle shape, with the *o*-diiminophenylene moiety aligned with the two *o*-aminobenzaldimine moieties (Figure 3). This is in stark contrast to **3a**, which featured a distinct saddle-shaped geometry. The increase in planarity upon metalation rationalizes the 10-fold decrease in solubility of **4a** compared to **3a** (1 vs 10 mg mL^–1^ in C_6_H_6_, respectively). Nickel complexes **4b**–**c** exhibit comparable NMR spectra. These NMR spectra, together with mass spectrometry, confirmed their composition and structure. Interestingly, the *o*-Ar–H hydrogens of the *o*-diiminophenylene moiety in **4c** appear significantly upfield shifted (δ = 5.70 ppm). SC–XRD studies support the explanation that this shift is due to the strong interactions between pendant phenyl rings that are in close proximity, arising from the rigid geometry of the complex (Figure 3). Geometrical differences between **4a** and **4c** are apparent from SC–XRD studies. While **4a** adopts a half-saddle shape and stacks in an antisymmetric fashion, **4c** displays a full saddle-shape due to steric hindrance of the additional phenyl rings that suppress planarity (∠centroid(C1–C10)-metal-centroid (C5–C6) < 180°).

**Figure 4:**
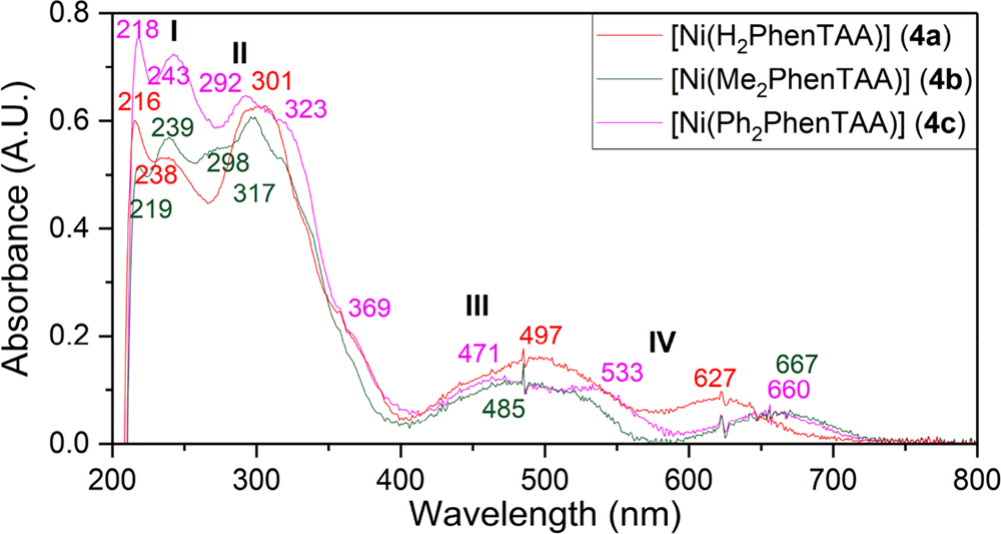
UV/vis spectra of Ni complexes **4a**–**c** in CH_2_Cl_2_ (1.0 mM, **4a**–**c**, 20 °C, air, OTTLE cell (l = 0.2 mm)).

This hindrance also suppresses π–π stacking, leading to a 10-fold solubility increase for **4c** compared to **4a** (10 vs 1 mg mL^–1^ in C_6_H_6_, respectively). Comparison of the UV/vis spectra reveals a strikingly similar pattern for all three nickel complexes. Four main features can be identified (labeled **I**, **II**, **III**, and **IV**) (Figure 4). The largest difference is observed for the longest wavelength band at 627, 667, and 660 nm for **4a**–**c**, respectively. The presence of the additional phenyl rings for **4c** leads to the emergence of additional shoulders at 323 and 369 nm and a visible absorption at 533 nm.

### Computational Investigations

**Scheme 2:**
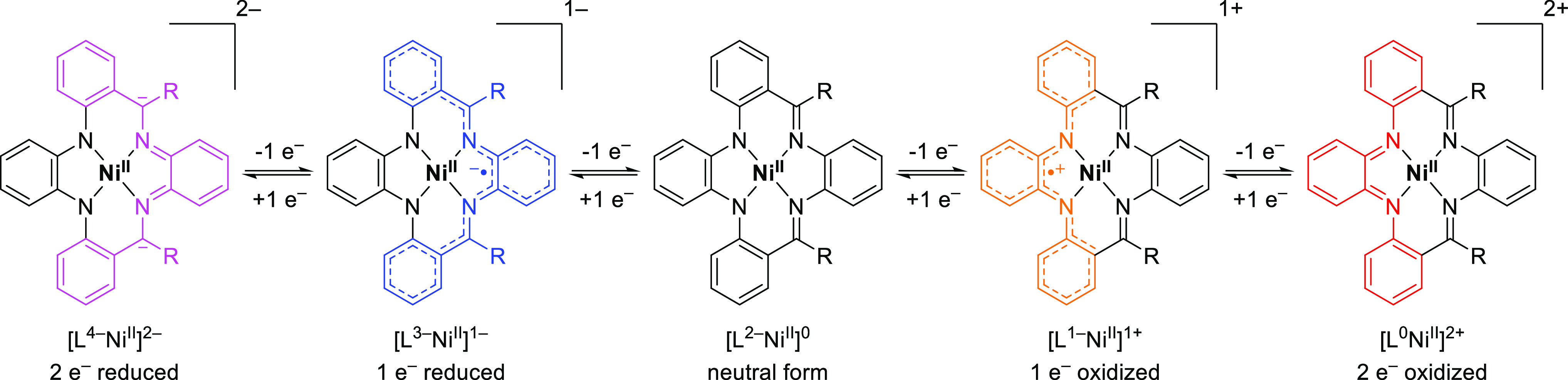
Schematic Representation of the Proposed Five Oxidation States of [Ni(R_2_PhenTAA)] Complexes from Doubly Reduced to Doubly Oxidized (−2 to +2) (R = H, Me, Ph for **4a**, **4b**, and **4c**, Respectively)

The design of the R_2_PhenTAA scaffold is based on the redox-active *o*-diarylphenylenediamine donor and *o*-diiminophenylene acceptor moieties. Consequently, we expect that the overall ligand is also redox-active and can be oxidized twice or reduced twice (Scheme 2). As a first elaboration into this chemistry, we turned to density functional theory calculations to ascertain structural and spin state changes upon oxidation/reduction as well as the spin density and overall electronic structure of the R_2_PhenTAA complexes. Calculations were performed using the Karlsruhe def2-TZVP basis sets using the TURBOMOLE 7.4.1. package (see Supporting Information, pages 77–85 and pages 133–139). To rule out any bias from the functional on the spin state of the complexes, all structures were calculated using the GGA BP86, the meta-GGA M06-L, and the hybrid B3LYP functionals. All three variants (R = H, Me, and Ph or **4a**–**c**) were calculated in five different oxidation states (−2, −1, 0, +1, and +2). For integer spin systems, the closed-shell singlet (CSS), open-shell singlet (OSS), and triplet and quintet spin states were calculated. For noninteger spin states, the doublet, quartet, and sextet spin states were calculated. To include any implicit solvent effects that may be present during experimental conditions, all electronic structures were evaluated using single-point calculations with the conductor-like screening model (COSMO). As solvents, MeTHF (ε = 6.97) and MeCN (ε = 38.8) were chosen as these were the most apolar and polar used in X-band EPR measurements (vide infra). The results are displayed in the Supporting Information, Tables S12–S17. The calculated bond distances, angles, and overall geometry are in excellent agreement with experimental SC–XRD results for the neutral complexes. All three functionals (BP86, M06-L, B3LYP) yielded identical prediction of the ground spin state with the exception of the doubly reduced **4a**^**2**^^–^, for which B3LYP predicts an open-shell singlet (OSS) ground state. For all other complexes, the ground state was always the lowest possible spin state (i.e., closed-shell *S* = 0 or doublet *S* = 1/2) for **4a**–**c** and their five oxidation states (−2 to +2). Spin density plots (Table S44, Supporting Information) reveal a similar pattern for all three nickel complexes, indicating that the overall redox properties arise as a result of the unique R_2_PhenTAA scaffold.

**Figure 5:**
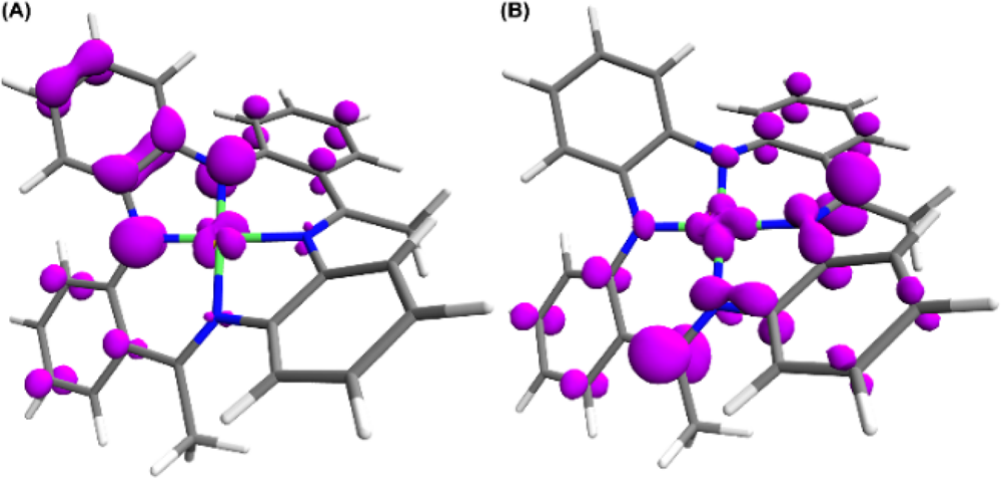
Spin density plots for (a) the singly oxidized (**4b**^**1+**^) and (b) the singly reduced (**4b**^**1–**^) forms of complex [Ni(Me_2_PhenTAA)] (**4b**). α-spin density (purple); β-spin density (yellow). Isosurface value = 0.04. Calculated at the BP86/def2-TZVP level of theory.

As such, for clarity, only the data for [Ni(Me_2_PhenTAA)] is displayed from here on (Figure 5, vide infra). Upon single electron oxidation, significant spin density develops at the diaryl-*o*-phenylenediamine moiety, centered largely on the two nitrogen atoms as well as the central phenyl ring. A small amount of spin density (∼10%) is also present on the filled Ni(*d_xy_*) orbital. Upon single electron reduction, the opposite occurs, and spin density develops on the α-imino carbon and the neighboring phenyl rings. In contrast to the oxidized forms, reduction leads to some spin density in nearly empty Ni(*d_x2-y2_*) orbital (∼13%). Interestingly, the resonance pattern on the *o*-aminobenzaldimine moiety is opposite to that obtained upon oxidation, where the α-spin density follows an alternating pattern. This suggests that the peripheral *o*-aminobenzaldimine phenylene rings have a stabilizing function and delocalize spin density for both the oxidized and reduced forms. Additionally, the reduced **4c**^**1–**^ exhibits spin density on the pendant phenyl rings as well (Table S44, Supporting Information).

### TD-DFT Calculations of Neutral Ni Complexes

**Figure 6:**
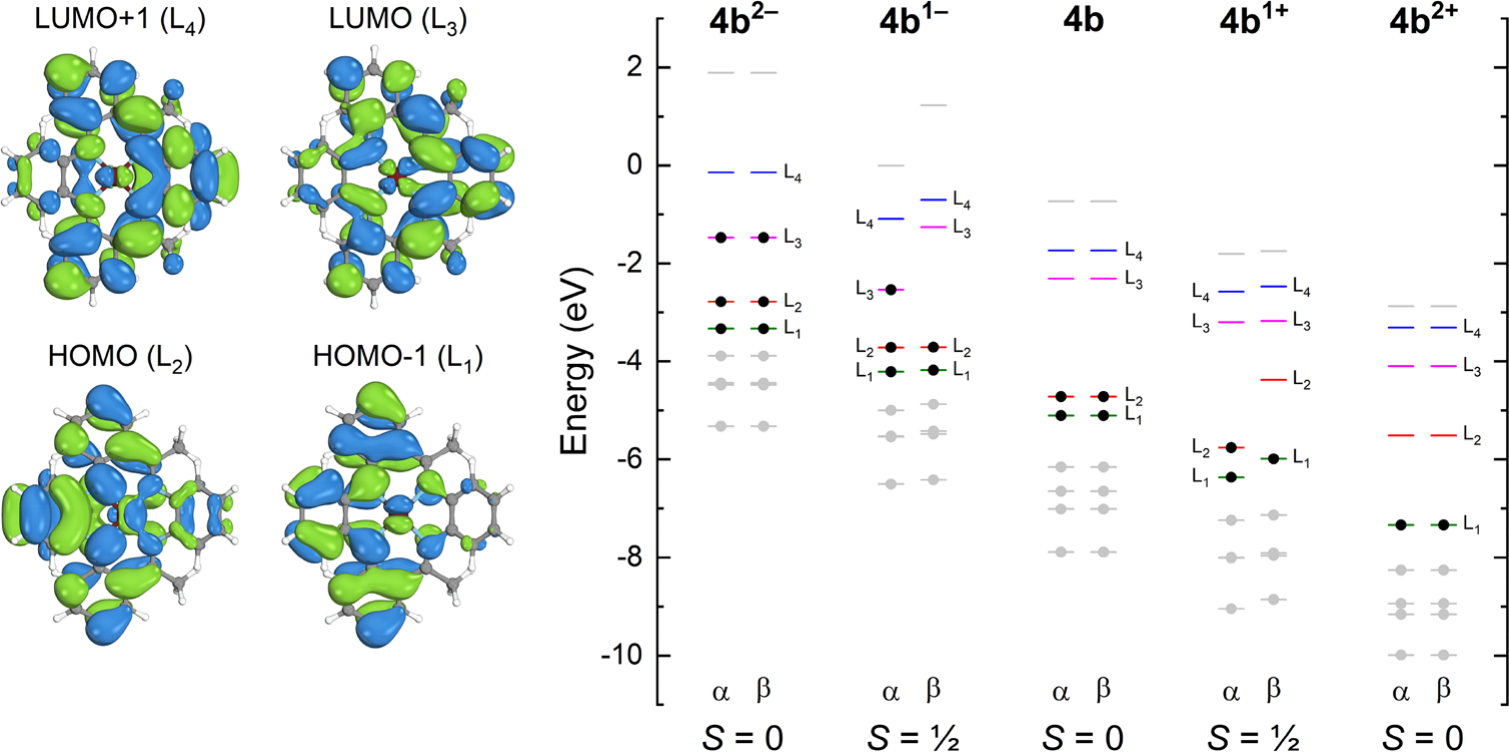
Molecular orbital diagram for the oxidation states of [Ni(Me_2_PhenTAA)] (**4b**) calculated with TD–DFT at the B3LYP/def2-TZVPP/CPCMC (CH_2_Cl_2_) level of theory using 100 roots. Visualized selected Hartree–Fock orbitals (left) for the neutral **4b** form are representative for all other oxidation states. Metal d-orbitals shown in gray (from high- to low-energy: *d*_*x2-y2*_, *d*_*z2*_, *d*_*yz*_ ≈ *d*_*xz*_, *d*_*xy*_) and color-coded selected ligand orbitals (L_1_–L_4_) were assigned via visual inspection and Löwdin population analysis.

Time-dependent density functional theory calculations were performed at the B3LYP/def2-TZVPP level of theory with the Tamm–Damcoff approximation using the ORCA 4.2.1 software package. The CPCMC solvation model was used in accordance with experimentally used solvents for UV/vis–SEC monitoring (*vide infra*) (CH_2_Cl_2_ for oxidized species; THF for reduced species) (see Supporting Information, pages 86–132). A frontier orbital and Löwdin population analysis for **4a**–**c** revealed a significant amount of ligand orbitals in between the metal d-orbitals in the overall MO diagrams (Figures S107–S109, Supporting Information). The HOMO and HOMO–1 are centered on the diamide moiety, whereas the LUMO and LUMO+1 are centered on the α-imino carbon and neighboring phenyl rings. These findings are in line with the DFT-obtained spin density plots. While these four orbitals exhibit some intermixing with metal *d*-orbitals, the overall wave function is dominated by the ligand. Löwdin population analysis revealed that even for the doubly reduced and oxidized complexes, the oxidation state of the metal center is unchanged and retains its initial Ni(II) state (Figure 6). The redox chemistry is centered entirely on the HOMO and LUMO of the neutral complex and changes in the overall wave function of the frontier orbitals is negligible. The relative order of the *d*-orbitals also does not change upon reduction or oxidation, with the exception of the virtually degenerate *d*_*xz*_-*d*_*yz*_ pair. TD–DFT calculations were performed with 100 roots to further elucidate the UV/vis spectra of Ni complexes **4a**–**c**. The obtained spectra of the neutral complexes show that the excitations are dominated by ligand → ligand excitations.

**Figure 7:**
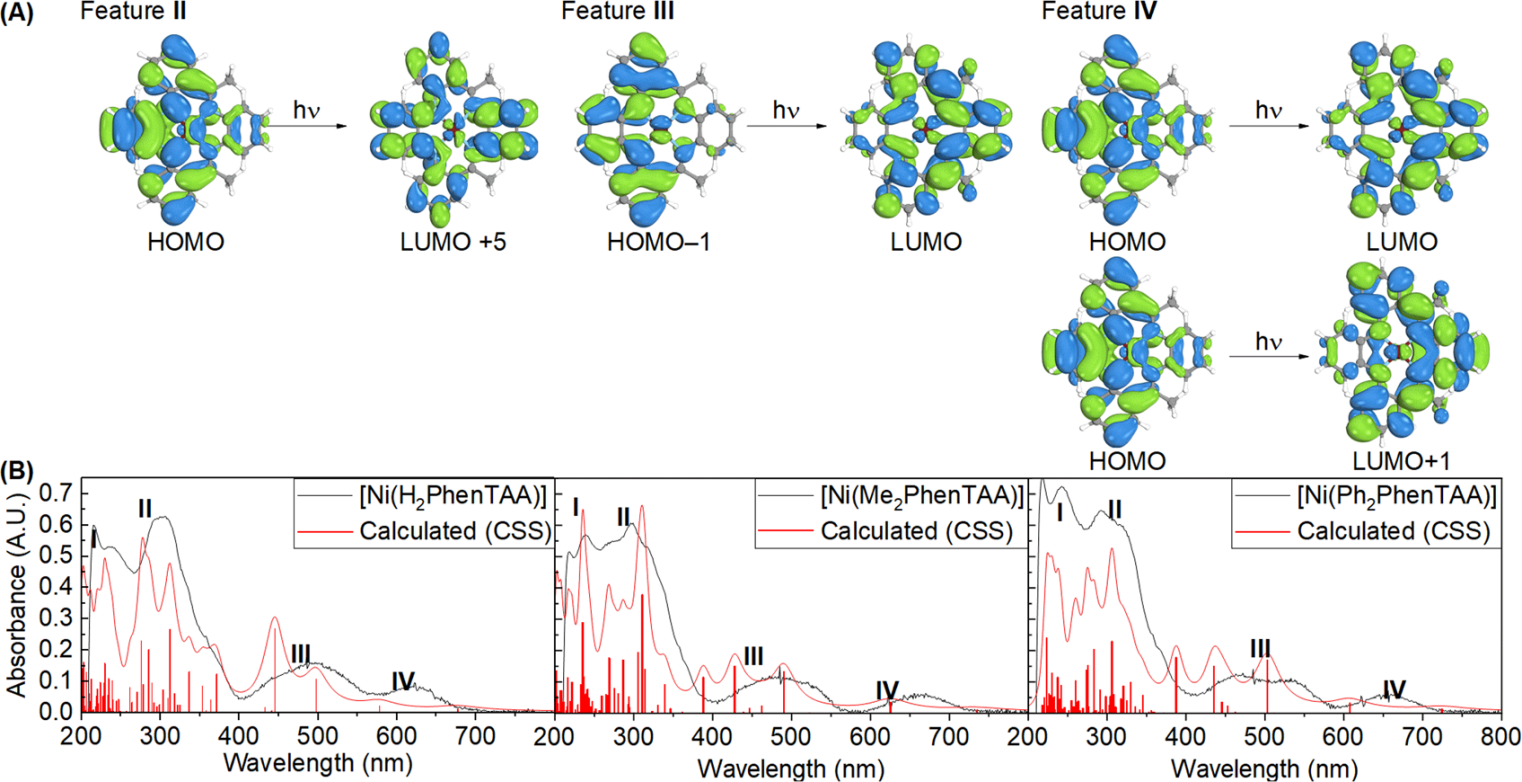
Orbitals involved in the predominant excitations for features **I**–**IV** (**A**). Experimental (black) and simulated (red) spectra for complexes **4a**–**c** with features **I**–**IV** labeled. The relative energy and oscillator strength of each excitation are represented in red bars and were fitted to the experimental spectra (**B**).

Features **II**, **III**, and **IV** correspond to the same excitations for all three Ni complexes (Figure 7), whereas **I** is more affected by R group substitution (see Supporting Information, pages 90–92 (R = H); pages 106–108 (R = Me); pages 123–125 (R = Ph)). Feature **II** corresponds to an excitation from the HOMO to a high-lying orbital with global quinoidal character.

Feature **III** predominantly conforms to the HOMO → LUMO+1 excitation, and feature **IV** conforms to the HOMO → LUMO and HOMO–1 → LUMO excitations. As is evident from the MO scheme in Figure 6, the excitations for features **III–IV** correspond to excitations from the donor diaryl-*o*-phenylenediamine moiety to the acceptor diimino moiety (Figure 7), revealing a unique donor → acceptor relationship for the R_2_PhenTAA macrocycle. These computational findings show much promise for the potential redox-active nature of the R_2_PhenTAA scaffold.

### Electrochemical Studies

**Figure 8:**
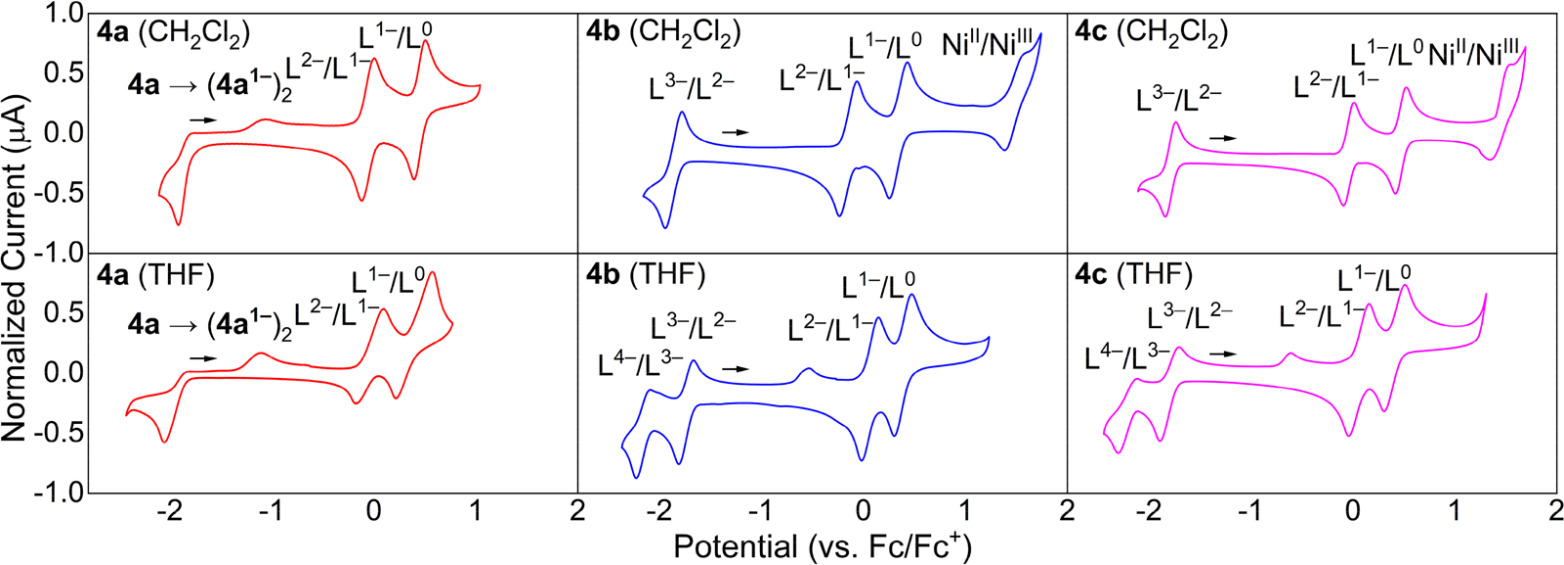
Cyclic voltammograms of complexes **4a**–**c** in CH_2_Cl_2_ and THF. Measurements were performed at 200 mV s^–1^ at room temperature in a 0.1 M TBAPF_6_ solution. WE = glassy carbon, CE = Pt wire; RE = Ag/AgCl (3.5 M KCl). All scans were collected within the accessible solvent windows. Potentials are reported vs Fc/Fc^+^.

Having studied the [Ni(R_2_PhenTAA)] complexes computationally, we sought to corroborate the results experimentally via cyclic voltammetry (CV). To study any solvent effects and have a large voltaic window, measurements were conducted in CH_2_Cl_2_ and THF (Figure 8). The values for every redox event are noted in Table 1 (vs Fc/Fc^+^). The three complexes **4a**–**c** exhibit comparable voltammograms in CH_2_Cl_2_ and THF, and the oxidative stability of CH_2_Cl_2_ and reductive stability of THF allow us to scan an electrochemical window from −2.4 V to +1.8 V (vs Fc/Fc^+^). When scanning to positive voltages, two electrochemically reversible waves are observed. These are assigned to ligand-based redox events.^19^

**Table 1:** All Redox Events for [Ni]-Complexes **4a**–**c** Based on Differential Pulse Voltammetry^b^

Peak type	[Ni(H_2_PhenTAA)]	[Ni(Me_2_PhenTAA)]	[Ni(Ph_2_PhenTAA)]
	**CH**_**2**_**Cl**_**2**_		
2nd reduction	-	–	–
1st reduction	–1.88 V	–1.86 V	–1.84 V
1st oxidation	–0.10 V	–0.15 V	–0.11 V
2nd oxidation	+0.41 V	+0.34 V	+0.40 V
3rd oxidation	–	+1.49 V^a^	+1.63 V
	**THF**		
2nd reduction	–	–2.16 V^a^	–2.25 V
1st reduction	–1.91 V	–1.73 V	–1.87 V
1st oxidation	–0.07 V	+0.08 V	+0.00 V
2nd oxidation	+0.31 V	+0.41 V	+0.34 V
3rd oxidation	–	–	–

a Based on CV due to overlap of solvent oxidation/reduction.

b Voltages reported versus Fc/Fc^+^.

Based on related nickel *o*-phenylenediamine complexes,^13^ these are assigned to the *o*-diiminosemiquinone and benzoiminoquinone forms, respectively (Scheme 2). For **4b**–**c**, a third oxidative wave is detected in CH_2_Cl_2_ at high positive voltages (+1.3–1.6 V). Based on literature reports, this wave is assigned to the Ni(II)/Ni(III) redox couple.^19^ The Ni(II)/Ni(III) wave was absent for **4a** since at these voltages, significant degradation occurred at the working electrode.

**Figure 9:**
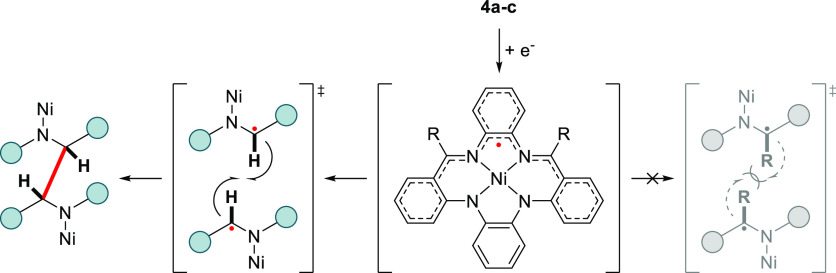
Schematic overview of the reductive dimerization of **4a** at the α-iminocarbon vs steric/electronic stabilization of the radical anionic species for **4b**–**c**.

A different picture arises when scanning to negative voltages. For the archetypical [Ni(H_2_PhenTAA)] (**4a**) complex, an electrochemically irreversible event is detected at −1.9 V, with a large peak separation of 770 mV at a 200 mV s^–1^ scan rate. Increasing the scan rate led to more narrow peak separation, indicative of an EC mechanism, i.e., reversible electron transfer followed by a reversible chemical step.^20^ As shown computationally (vide supra), this is most likely a ligand-centered reduction of the α-iminocarbon. The electrochemically irreversible nature is proposedly due to reversible chemical dimerization of the two ligand radical species (Figure 9), as this is also observed for [Ni(Salphen)] complexes.^21^

Moreover, a metal-centered Ni(II)/Ni(I) redox couple is excluded on the basis of literature reports, as these tend to occur at even more negative voltages.^19,22^ The ketimine based complexes (R = Me/Ph) **4b**–**c** exhibit contrasting electrochemical properties, featuring a fully electrochemically reversible reduction. This behavior is consistent with the hypothesis of dimerization occurring at the α-imino carbon, as the electrogenerated radical experiences enhanced steric and electronic stability at this carbon (Figure 9). Cyclic voltammetry on **4b**–**c** in THF was possible down to −2.4 V vs Fc/Fc^+^, revealing another reduction at −2.16 V (**4b**) and −2.25 V (**4c**). This additional wave is not electrochemically reversible and gives rise to an additional, smaller wave at −0.33 V (**4b**) and −0.47 V (**4c**). At higher scan rates, the cathodic current of this second reduction increases relatively to the anodic current, and an EC mechanism is assigned to this redox event. Based on computational data, this is assigned to a second ligand-based reduction of the remaining imine position to yield a tetraanionic R_2_PhenTAA ligand (Scheme 2).

### Spectroelectrochemistry

**Figure 10:**
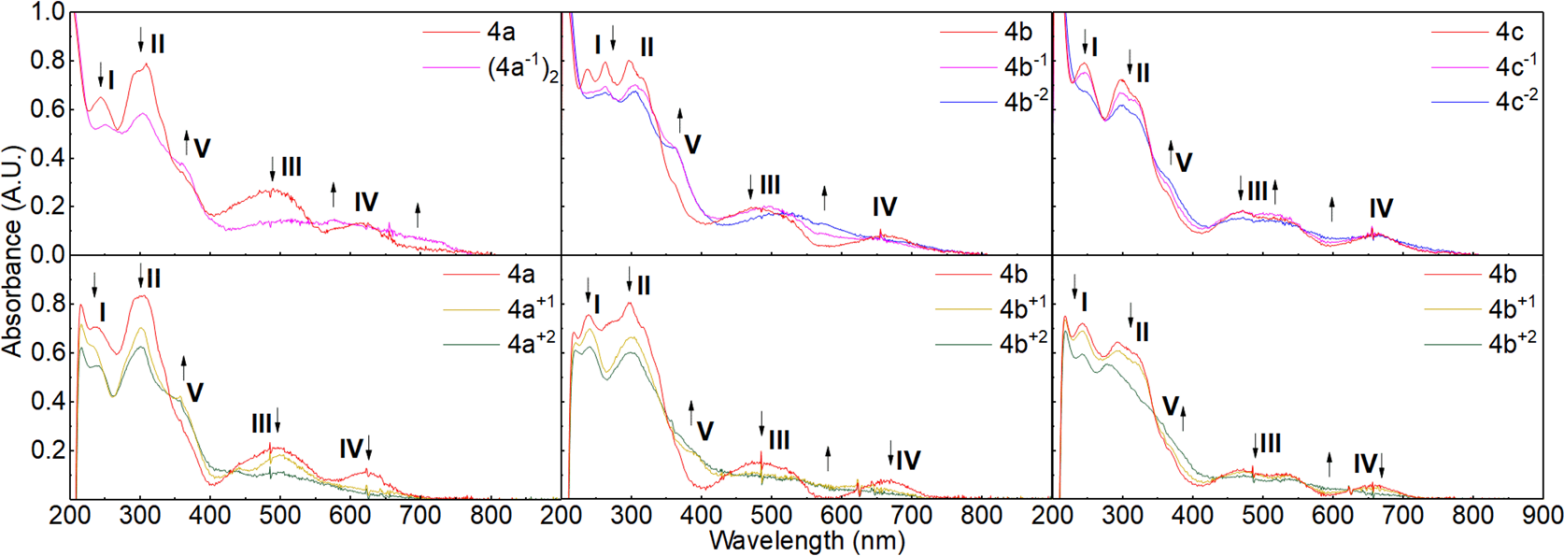
UV/vis spectra obtained via spectroelectrochemical monitoring of reduced (top) Ni complexes **4a**–**c** in THF and oxidized (bottom) in CH_2_Cl_2_ with main features **I**–**IV** labeled. Intermediate species were determined on the basis of isosbestic points.

UV/vis-spectroelectrochemical measurements (UV/vis–SEC) were performed to gather more information on the redox chemistry of nickel complexes **4a**–**c** and to assign the ligand/metal oxidation states. The spectral changes are listed in Table S6 and visualized in Figure 10. At 0 V (vs Ag wire), all species revealed the same spectrum as with the cell turned off in both THF and CH_2_Cl_2_. This confirms that the CV assignments with respect to reduction or oxidation are correct. Overall, the UV/vis–SEC measurements corroborate the results observed with CV with four different detectable species for **4a** (one reduced and two oxidized species) and five for **4b**–**c** (two reduced and two oxidized species). The most discernible spectral changes occur upon the first oxidation or reduction for all three complexes, with the second oxidation or reduction resulting in only minor spectral perturbations. This suggests that departure from the dianonic form of the R_2_PhenTAA macrocycle induces the strongest change in the overall excitations, which applies to both reduction and oxidation of the ligand. This observation is in line with the results of the TD–DFT calculations, which indicate that the electronic excitations are dominated by donation from the diaryl*-o*-phenylenediamine moiety to the acceptor diimino moiety. The first oxidation of the donor moiety (or reduction of the acceptor) is expected to strongly perturb the intramolecular donor → acceptor process, manifested by dramatic changes in the UV/vis spectrum. Additional oxidation or reduction leads to a further departure from the already disturbed donor → acceptor relationship, giving rise to a minor change to the overall spectrum. Reduction of the Ni complexes produces a clear difference for **4a** vs **4b**–**c**, in line with CV measurements. Going from **4a** to **(4a**^**1–**^**)**_**2**_ (see Figure 10), two main features for **4a** (**III** and **IV**) broaden into several large features at 519, 579, and 733 nm with **III**. For **4b**–**c**, features **III** and **IV** only slightly shift to longer wavelengths but do not broaden as much. Therefore, these two features are likely strongly affected by changes on the α-iminocarbon.

In line with with TD–DFT calculations, these features **III** and **IV** match excitations from the donor diaryl*-o*-phenylenediamine moiety to the acceptor diimino moiety and reduction of the diimino moiety is expected to strongly affect such excitations. Upon oxidation, the three complexes all gain a new broad feature at around 360 nm (feature **V**) that is present for both the singly and doubly oxidized complexes. For the defining features **III** and **IV**, a distinct difference between **4a** and **4b**–**c** is again present. For the first oxidation, feature **III** sharpens and is red-shifted for **4a**, whereas for **4b**–**c**, it decreases in intensity but does not shift. Feature **IV** collapses into the baseline for **4a** but is broadened and blue-shifted for **4b**–**c**. For the second oxidation to the benzoiminoquinone state, both features **III** and **IV** collapse almost entirely into the baseline for all three complexes. This spectral difference between **4a** and **4b**–**c** is attributed to the geometric difference of the complexes as per XRD of the neutral complexes and DFT studies of the reduced and oxidized complexes (*vide supra*). The collapse of features **III** and **IV** for all three complexes in their doubly oxidized form can be attributed to the absence of electrons in the HOMO of the neutral parent complexes.

### TD–DFT Calculations of the Oxidized and Reduced Ni Complexes

**Figure 11:**
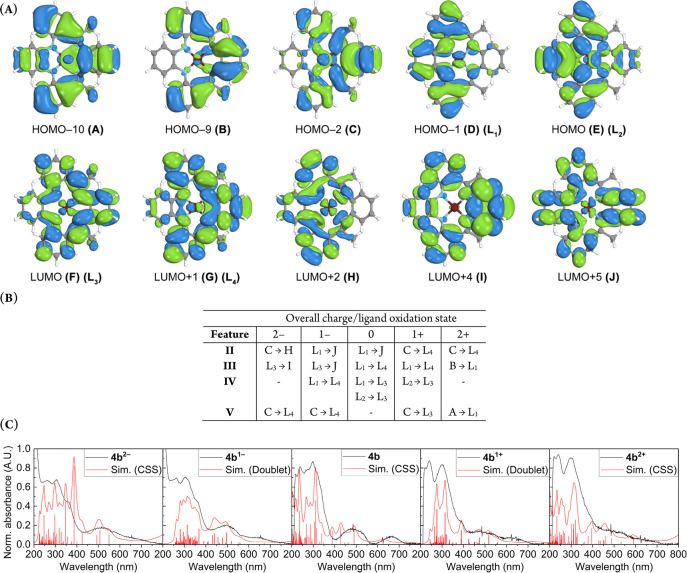
Most relevant orbitals involved in the five main features for [Ni(Me_2_PhenTAA)] (**4b**) and oxidation states (−2, −1, 0, +1, +2). Frontier orbitals are labeled L_1_–L_4_ as in Figure 6 (A). Table with the most dominant transitions of the **4b** redox series per oxidation state and spectral feature (B). Experimental (black) and simulated (red) spectra for **4b** and oxidation states (−2, −1, 0, +1, +2) (C, left to right). The relative energy and oscillator strength of each excitation are represented in red bars and were fitted to the experimental spectra.

To acquire more insight into these spectral changes and correlate them to the proposed computed states, TD–DFT calculations were performed for all five oxidation states and varying spin states using the same parameters as for the neutral complexes (vide supra). For integer spin states, the closed-shell singlet (CSS), triplet, and quintet states were calculated. For noninteger spin states, the doublet, quartet, and sextet spin states were calculated. In all cases, the best match was obtained for the low-spin states, in line with the ground state DFT calculations. For clarity, the **4b** redox series and the orbital transitions are visualized here (Figure 11). The **4a** and **4c** series can be found in the Supporting Information (pages 90–132). Feature **I** cannot be assigned to a single, dominant transition and is composed of several excitations for all three complexes in all oxidation states. These often involve excitation from either a very low-lying orbital to the LUMO/LUMO+1 or from the HOMO/HOMO–1 to a high energy vacant orbital.

The other features are highly dependent on the overall ligand oxidation state but are quite similar for all three Ni complexes with the same oxidation state. In both the singly reduced and neutral form, this changes to a transition of the diaryl-*o*-phenylenediamine site to a global quinoidal structure. In the oxidized forms, feature **II** primarily consists of a π → π* transition of the diimino moiety. Features **III** and **IV**, as with the neutral complexes, correspond largely to excitations within the four frontier orbitals described in Figure 6. Exceptions are the doubly reduced complexes (**4b**^**2–**^–**4c**^**2–**^), because these show excitation from the quinoidal HOMO to higher lying global π* antibonding states. Likewise, the doubly oxidized complexes do not have a defined feature **IV**, and the still present feature **III** now reveals a reversed donor → acceptor relationship, with excitations occurring mainly from the diimino moiety to the benzoiminosemiquinone moiety. Feature **V**, a feature not present for the neutral complexes, but visible for both the reduced and oxidized forms, corresponds mainly to excitations from the acceptor diimino moiety to higher lying π* antibonding states. Changing the oxidation state from −2 to +2 only shifts the starting donor orbital, still centered on the diimino moiety, to a lower energy.

### X-Band EPR Spectroscopy

**Figure 12:**
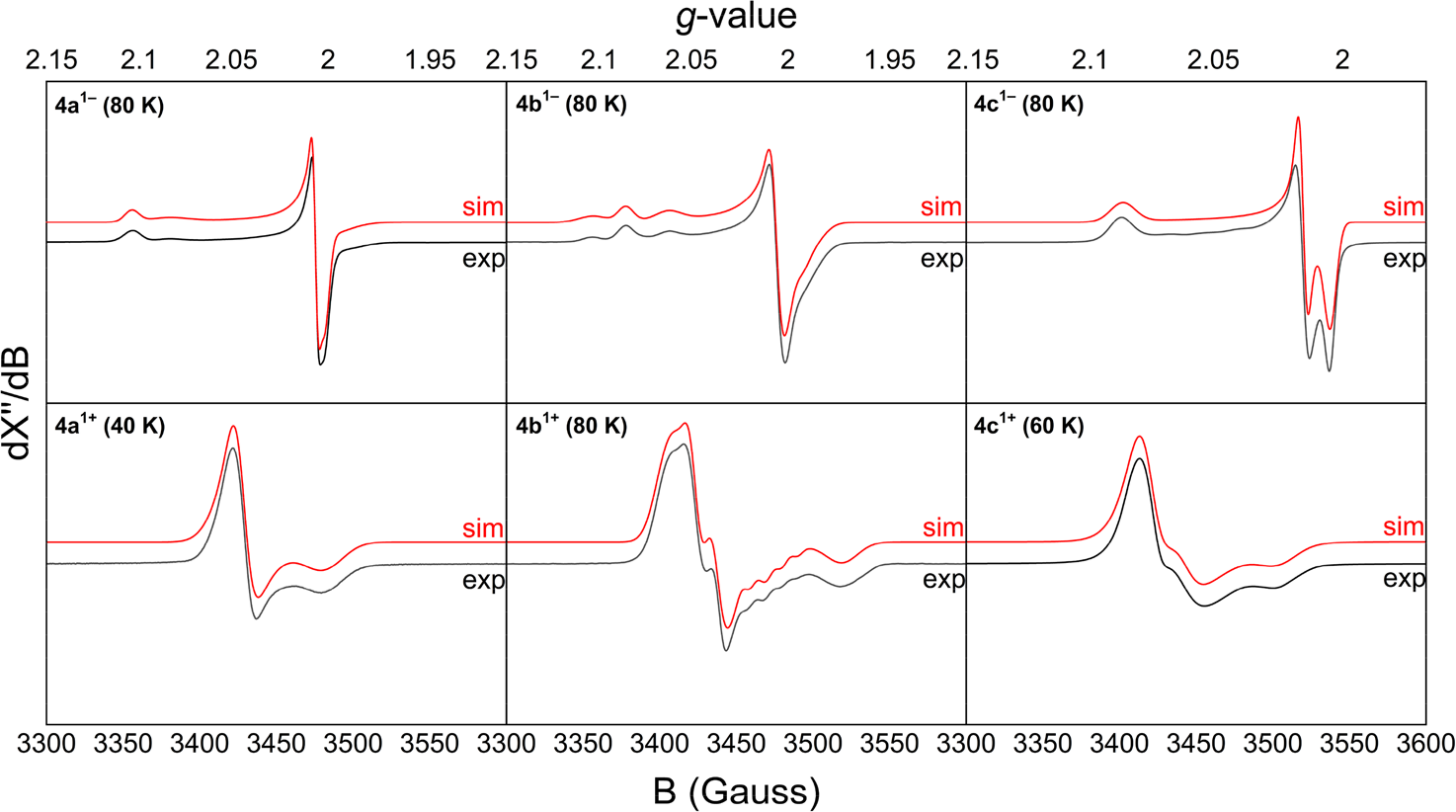
Anisotropic X-band EPR spectra obtained after reduction of Ni complexes **4a**–**c** with sodium anthracenide in 2-MeTHF (top) or oxidation with thianthrenium tetrafluoroborate in CH_2_Cl_2_:MeCN (1:1 v/v) (bottom). For experimental details, see Supporting Information, page 36.

Seeking additional experimental evidence for the ligand-based nature of the redox events for R_2_PhenTAA complexes **4a**–**c**, we turned to X-band EPR spectroscopy. Anisotropic spectra recorded in frozen solutions (40–80K) are shown in Figure 12, and the obtained simulated data are reported in Table 2. Isotropic spectra recorded at RT are shown in the Supporting Information (Figures S35–S40). Chemical reductions were performed using 1 eq. of freshly produced sodium anthracenide solution in 2-MeTHF. Chemical oxidation was achieved using 1 eq. of a solution of freshly prepared thianthrenium tetrafluoroborate (ThiBF_4_) in CH_2_Cl_2_. Reduction of **4a** gave a faint and rapidly diminishing signal at RT in 2-MeTHF with a signal at *g*_iso_ = 2.0026, in line with a transient ligand radical. A follow-up ^1^H NMR measurement of this sample revealed the formation of a new diamagnetic species that does not correspond to the neutral complex **4a**. This conforms to the hypothesis that upon reduction, the radical **4a**^**1–**^ dimerizes into a diamagnetic species. Freeze quenching of **4a**^**1–**^ gave an axial anisotropic spectrum at 80 K void of any hyperfine interactions (HFIs) (*g*_*11*_ = *g*_*22*_ = 2.0043; *g*_*33*_ = 2.1047) (Figure 12). These values correspond to a metastable ligand radical. The *g*-value at 2.10 suggests spin–orbit coupling involving the SOMO interacting with an empty orbital close in energy.

**Table 2:** Simulated *g*-Values of Singly Reduced/Oxidized Complexes **4a**–**c** Derived from X-Band EPR Spectroscopy Measurements at 40–80 K

	**4a**			**4b**			**4c**		
	*g*_11_	*g*_22_	*g*_33_	*g*_11_	*g*_22_	*g*_33_	*g*_11_	*g*_22_	*g*_33_
**Reduction**									
*Species 1*	2.0013	2.0064	2.1043	2.0010	2.0061	2.1040	2.0149	2.0050	2.0868
*Species 2*	2.0074	1.9963	2.0844	1.9947	2.0071	2.0853			
*Species 3*				1.9864	2.0061	2.0625			
**Oxidation**									
*Species 1*	2.0104	2.0140	1.9789	1.9580	2.0044	2.0148	1.9855	2.0133	2.0146
*Species 2*				1.9867	2.0153	2.0243	1.9666	2.0034	2.0036

Reduction of **4b** and **4c** at RT gave a broad and stable signal at *g*_*iso*_ = 2.0223 and *g*_*iso*_ = 2.0380, respectively, in line with ligand radical species. Interestingly, the isotropic spectrum of **4b**^**1**^^–^ features an apparent large hyperfine interaction with a single (apparently *I* = ^3^/_2_) nucleus. Due to all hydrogen atoms occurring at least twice (due to symmetry) and because naturally occurring nickel isotopes do not have a nuclear spin, this could only be simulated by including hyperfine coupling with a single ^23^Na atom. Apparently, sodium ions introduced together with the reducing agent bind to **4b**^**1–**^. Anisotropic spectra were obtained after freeze quenching, and measurement at 80 K revealed a mostly axial spectrum, similar to that of **4a**^**1–**^. However, for **4b**^**1–**^, the feature at high *g*-values corresponds to three different species. As with the isotropic room temperature spectrum, this is attributed to various coordination modes of the sodium counterion. Interestingly, this is not observed for **4c**^**1–**^, which shows a rhombic spectrum at higher *g*-value (*g*_*11*_ = 2.0149; *g*_*22*_ = 2.0050; *g*_*33*_ = 2.0868), void of HFIs. For both **4b** and **4c**, this is in accordance with a ligand radical.

Oxidation of **4a**–**c** with ThiBF_4_ afforded clean, stable isotropic spectra for all three complexes (**4a**: *g*_*iso*_ = 1.9994; **4b**: *g*_*iso*_ = 1.9991 and **4c**: *g*_*iso*_ = 2.0004). Their *g*-values are again in line with ligand radical species. Addition of a CH_2_Cl_2_ solution of ThiBF_4_ to an equimolar solution of **4a**–**c** in MeCN, followed by freeze quenching and subsequent measurement with X-band EPR, revealed a slightly rhombic EPR spectrum for **4a**^**1+**^ (*g*_*11*_ = 2.0104; *g*_*22*_ = 2.0140; *g*_*33*_ = 1.9789) measured at 40 K. As with reduced **4b**^**1–**^, oxidation of **4b** to **4b**^**1+**^ gave a rhombic spectrum that featured several additional small hyperfine interactions. In line with CV measurements in THF (another coordinating solvent), this is attributed to coordination of the now cationic **4b**^**1+**^ with acetonitrile, resulting in several different solvent adducts (Species 1: *g*_*11*_ = 1.9580; *g*_*22*_ = 2.0044; *g*_*33*_ = 2.0148 (weight = 0.50). Species 2: *g*_*11*_ = 1.9867; *g*_*22*_ = 2.0153; *g*_*33*_ = 2.0243 (weight = 0.50) (A^14N1^_11_ = 32.1603 MHz; A^14N1^_22_ = 0 MHz; A^14N1^_33_ = 0 MHz) (A^14N2^_11_ = 25 MHz, A^14N1^_22_ = 0 MHz, A^14N1^_33_ = 0 MHz). A similar coordination effect may be present for **4c**^**1+**^, although a rhombic spectrum void of HFIs was obtained (Species 1: *g*_*11*_ = 1.9855; *g*_*22*_ = 2.0133; *g*_*33*_ = 2.0146 (weight = 0.63). Species 2: *g*_*11*_ = 1.9666; *g*_*22*_ = 2.0034; *g*_*33*_ = 2.0036 (weight = 0.37)). These *g*-values correspond to ligand-centered radicals, analogous to the room-temperature spectra. All the EPR spectra gave signals that correspond to a doublet spin state (*S* = ^1^/_2_). These findings are in line with the UV/vis–SEC measurements and the computational results from DFT and TD–DFT analysis.

## Conclusions

To summarize, we have described the synthesis and characterization of a series of new conjugated macrocycles: R_2_PhenTAA. The imine position has been substituted with either a methyl or phenyl group, and three new [Ni(R_2_PhenTAA)] complexes were synthesized and characterized (R = H, Me, Ph). Electrochemical studies, using a combination of cyclic voltammetry and spectro-electrochemistry, have revealed the extensive redox chemistry of [Ni(R_2_PhenTAA)]. The incorporation of the diaryl-*o*-phenylenediamine moiety gave rise to two ligand-based oxidation states for all three Ni complexes. Substitution of the imine group with a methyl or phenyl both led to stabilization of the first reduction, preventing dimerization. This allowed further access to a second reduction, giving access to a total of five spectroscopically detectable different ligand-based oxidation states for R_2_PhenTAA that are electrochemically stable over a range of +4.2 V. In all cases, the metal remains in its Ni(II) oxidation state. These findings show that combining two different redox-active moieties within a single conjugated macrocycle leads to a unique electronic structure that exhibits a clear donor → acceptor dual nature. However, the fundamental nature of the individual redox moieties is still retained, demonstrating that a conjugated redox-active macrocycle is not fully dependent on a globally delocalized nature. Apart from the redox properties of PhenTAA, the donor → acceptor electronic structure also leads to unique photophysical properties, revolving largely around its four frontier ligand orbitals. We anticipate these results will also lead to further development and insights into other ligand-mediated radical group transfer reactions.

## Experimental Section

### General Considerations

All reactions were carried out under argon using standard Schlenk techniques or under an inert atmosphere in a N_2_-filled glovebox, unless noted otherwise. All chemicals were of commercial grade and used without further purification, unless noted otherwise. All solvents used were predried using either a Solvent Purification System (SPS) from MBraun (MB SPS-800, with standard MBraun drying columns) or were dried and distilled from sodium (PhMe/pentane), sodium/benzophenone (THF/Et_2_O) or CaH_2_ (CH_2_Cl_2_, MeOH). All solvents were further dried/stored on activated 3 Å molecular sieves and degassed by sparging with argon unless mentioned otherwise. Additional information regarding NMR, EPR, HRMS, UV/vis, CV, XRD, and spectroelectrochemical measurements can be found in the Supporting Information.

### Synthesis and Characterization of Ligands

#### Synthesis of 2-Aminobenzaldehyde (**1a**)

Compound **1a** was prepared according to a modified literature procedure.^14^ To a 1 L round-bottom flask was added Fe (55.85 g, 1 mol), 2-nitrobenzaldehyde (15.11 g, 0.1 mol), 300 mL of absolute EtOH, and 75 mL of H_2_O. This mixture was bubbled through with Ar for 10 min after which HCl (0.75 mL; 37%) was added. The mixture was brought to reflux under Ar for 14 h. After cooling down, the mixture was filtered over Celite and washed with EtOH. The filtrate was concentrated in vacuo, subsequently extracted with CH_2_Cl_2_/H_2_O, washed with brine, dried with Na_2_SO_4_, filtered over cotton, and evaporated in vacuo until a yellow oil remained. Next, the crude product was purified via column chromatography (CH_2_Cl_2_). The pure fractions were identified via TLC (**1a***R*_*F*_ = 0.2) and evaporated in vacuo. Yield: 10 g (83%). ^1^H NMR (400 MHz, DMSO-*d*_6_): δ 9.81 (s, 1H; C*H*O), 7.51 (d, ^3^*J*_H,H_ = 7.2 Hz, 1H; *o*-Ar*H*), 7.30 (t, *J* = 7.7 Hz, 1H), 7.10 (s, 2H; *o-*N*H*_2_), 6.76 (d, *J* = 8.5 Hz, 1H), 6.63 (t, *J* = 7.3 Hz, 1H) (Figure S59).

#### General Procedure A for Dicarbonyl Building Blocks **2a**–**2c**

*n*-Bu_2_O (200 mL) was filtered over activated basic alumina. To this was added *o*-diiodobenzene (1 equiv) and amine (2.2 equiv). To a separate 1 L Schlenk flask was added 500 mesh Cu powder (20 mol %), CuI (2 mol %), and K_2_CO_3_ (4 equiv). To this Schlenk flask the *n*-Bu_2_O solution was added, and this mixture was brought to reflux for 6 days. After cooling, the salts were filtered off over a short silica plug and washed with CH_2_Cl_2_ until the effluent was colorless, and the solvent was removed in vacuo. The crude mixture was either recrystallized from CH_2_Cl_2_/pentane or purified via column chromatography (CH_2_Cl_2_/petroleum ether (40–60) gradient).

#### Synthesis of *N*,*N*′-(1,2-Phenylenediamino)-bis(2-aminobenzaldehyde) (**2a**)

The reaction was performed according to general procedure A (see above) on a 37.5 mmol scale. The product was isolated after column chromatography (CH_2_Cl_2_/petroleum ether (40–60) gradient). Gradient: 50% CH_2_Cl_2_/PE (fractions 1–30); 75% CH_2_Cl_2_/PE (fractions 31–50); 100% CH_2_Cl_2_/PE (fractions 51–80); 25% EtOAc/75% CH_2_Cl_2_ (fractions 81–all color eluted). The first fraction is the monocoupled product (*R*_*F*_ = 0.9), the second fraction is compound **2a** (*R*_*F*_ = 0.6), and the final fraction is the 2-aminobenzaldehyde starting material (*R*_*F*_ = 0.2). The pure fractions were identified via TLC and evaporated in vacuo. Yield: 6.64 g (56%). ^1^H NMR (400 MHz, CD_2_Cl_2_): δ 9.84 (s, broad, 2H, N**H**), δ 9.81 (s, 2H, C**H**O), δ 7.54 (dd, ^*3*^*J*_*H,H*_ = 7.7 Hz, ^*4*^*J*_*H,H*_ = 1.6 Hz, 2H, *o*-C**H**), 7.50 (dd, ^*3*^*J*_*H,H*_ = 5.9 Hz, ^*4*^*J*_*H,H*_ = 3.6 Hz, 2H, *m*-C**H**), 7.32 (td, ^*3*^*J*_*H,H*_ = 8.6, ^*4*^*J*_*H,H*_ = 1.7 Hz, 2H, *m*-C**H**), 7.22 (dd, ^*3*^*J*_*H,H*_ = 6.0, ^*3*^*J*_*H,H*_ = 3.5 Hz, 2H, *o*-C**H**), 7.01 (d, ^*3*^*J*_*H,H*_ = 8.5 Hz, 2H, *o*-C**H**), 6.83 (td, ^*3*^*J*_*H,H*_ = 7.9, ^*4*^*J*_*H,H*_ = 0.8 Hz, 2H, *m*-C**H**) (Figure S60). ^13^C NMR (126 MHz, CD_2_Cl_2_): δ 194.61 (2C, **C**HO), 147.93 (2C, *ipso*-**C**), 136.88 (2C, *o*-**C**), 135.83 (2C, *m*-**C**), 134.42 (2C, *ipso*-**C**), 125.83 (2C, *o*-**C**), 125.34 (2C, *m*-**C**), 120.34 (2C, *ipso*-**C**), 117.91 (2C, *m*-**C**), 113.48 (2C, *o*-**C**) (Figure S61). HRMS: *m*/*z* = 299.1190. **2a**^**+**^ (*z* = 1) calc. 317.1290. **[2a**-H_2_O]^+^ (*z* = 1) calc. 299.1184.

#### Synthesis of *N*,*N*′-(1,2-Phenylenediamino)-bis(2-aminoacetophenone) (**2b**)

The reaction was performed according to general procedure A (see above) on a 5 mmol scale. The product was isolated from the crude mixture after recrystallization from CH_2_Cl_2_/pentane. Yield: 735 mg (43%). ^1^H NMR (500 MHz, CD_2_Cl_2_): δ 10.31 (s, broad, 2H, N**H**), 7.77 (dd, ^*3*^*J*_*H,H*_ = 8.1 Hz, ^*4*^*J*_*H,H*_*=* 1.6 Hz, 2H, *o*-C**H**), 7.44 (dd, ^*3*^*J*_*H,H*_ = 5.9, ^*4*^*J*_*H,H*_ = 3.6 Hz, 2H, *m*-C**H**), 7.25 (ddd, ^*3*^*J*_*H,H*_ = 8.6, ^*3*^*J*_*H,H*_ = 7.0, ^*4*^*J*_*H,H*_ = 1.6 Hz, 2H, *m*-C**H**), 7.15 (dd, ^*3*^*J*_*H,H*_ = 6.0, ^*4*^*J*_*H,H*_ = 3.5 Hz, 2H, *o*-C**H**), 7.02 (dd, ^*3*^*J*_*H,H*_ = 8.5, ^*4*^*J*_*H,H*_ = 1.1 Hz, 2H, *o*-C**H**), 6.72 (ddd, ^*3*^*J*_*H,H*_ = 8.1, ^*3*^*J*_*H,H*_ = 7.0, ^*4*^*J*_*H,H*_ = 1.2 Hz, 2H, *m*-C**H**), 2.54 (s, 6H, C**H**_3_) (Figure S65). ^13^C NMR (126 MHz, CD_2_Cl_2_): δ 201.52 (2C, **C**OCH_3_), 148.13 (2C, *ipso*-**C**), 135.16 (2C, *ipso-***C**), 134.78 (2C, *m*-**C**), 132.84 (2C, *m*-**C**), 125.29 (2C, *o*-**C**), 125.15 (2C, *m*-**C**), 120.14 (2C, *ipso*-**C**), 117.23 (2C, *m*-**C**), 114.93 (2C, *o*-**C**), 28.40 (2C, **C**H_3_) (Figure S66). HRMS: *m*/*z* = 345.1608. **2b**^**+**^ (*z* = 1) calc. 345.1603.

#### Synthesis of *N*,*N*′-(1,2-Phenylenediamino)-bis(2-aminobenzophenone) (**2c**)

The reaction was performed according to general procedure A (see above) on a 5 mmol scale. The product was isolated after column chromatography (CH_2_Cl_2_/petroleum ether (40–60) gradient). The pure fractions were identified via TLC and evaporated in vacuo. Yield: 1.91 g (81%). ^1^H NMR (500 MHz, CD_2_Cl_2_): δ 9.88 (s, broad, 2H, N**H**), 7.61 (dt, ^*3*^*J*_*H,H*_ = 7.5 Hz, ^*4*^*J*_*H,H*_ = 1.5 Hz, 4H, *o*-C**H**), 7.55 (tt, ^*3*^*J*_*H,H*_ = 7.4 Hz, ^*4*^*J*_*H,H*_ = 2.0 Hz, 2H, *p*-C**H**), 7.51 (dd, ^*3*^*J*_*H,H*_ = 5.9 Hz, ^*3*^*J*_*H,H*_ = 3.5 Hz, 2H, *o*-C**H**), 7.47 (m, 2H, *o*-C**H**), 7.45 (m, 4H, *m*-C**H**), 7.27 (td, ^*3*^*J*_*H,H*_ = 7.5 Hz, ^*4*^*J*_*H,H*_ = 1.1 Hz, 2H, *m*-C**H**), 7.19 (dt, ^*3*^*J*_*H,H*_ = 9.6 Hz, ^*3*^*J*_*H,H*_ = 3.7 Hz, 2H, *m*-C**H**) 7.13 (dd, ^*3*^*J*_*H,H*_ = 7.8 Hz, ^*4*^*J*_*H,H*_ = 0.6 Hz, 2H, *o*-C**H**) 6.88 (td, ^*3*^*J*_*H,H*_ = 7.4 Hz, ^*4*^*J*_*H,H*_ = 1.1 Hz, 2H, *m*-C**H**) (Figure S70). ^13^C NMR (126 MHz, CD_2_Cl_2_): δ 199.37 (2C, **C**O), 148.21 (2C, *ipso*-**C**), 140.35 (2C, *ipso*-**C**), 135.12 (2C, *o*-**C**), 134.81 (2C, *ipso*-**C**), 134.47 (2C, *o*-**C**), 131.83 (2C, *p*-**C**), 130.04 (4C, *o*-**C**), 128.52 (4C, *m*-**C**), 125.17 (2C, *m*-**C**), 124.61 (2C, *o-***C**), 120.83 (2C, *ipso*-**C**), 117.27 (2C, *m*-**C**), 115.27 (2C, *o*-**C**) (Figure S71). HRMS: *m*/*z* = 468.1984. **2c**^**+**^ (*z* = 1) calc. 468.1838.

#### Synthesis of H_2_ (H_2_PhenTAA) (**3a**)

Compound **2a** (1.00 g, 3.16 mmol), *o*-phenylenediamine (342 mg, 3.16 mmol), and Zn(OAc)_2_·2H_2_O (693.3 mg, 3.16 mmol) were added to a 500 mL Schlenk flask, and to this mixture was added dried and degassed methanol (150 mL). The mixture was stirred at reflux overnight, during which the product precipitated out of the solution. The product was filtered off aerobically, washed with cold methanol, and dried in vacuo. Yield: 1.11 g (90%). ^1^H NMR (400 MHz, CD_2_Cl_2_): δ 12.20 (s, broad, 2H, N**H**), 8.73 (s, 2H, N = C**H**), 7.75 (dt, ^*3*^*J*_*H,H*_ = 9.6 Hz, ^*4*^*J*_*H,H*_ = 3.6 Hz, 2H, *o*-C**H**), 7.60 (d, ^*3*^*J*_*H,H*_ = 8.6 Hz, 2H, *m*-C**H**), 7.45 (dd, ^*3*^*J*_*H,H*_ = 7.8 Hz, ^*4*^*J*_*H,H*_ = 1.5 Hz, 2H, *o*-C**H**), 7.39–7.35 (m, 2H, *o*-C**H**), 7.35–7.33 (m, 2H, *m*-C**H**), 7.30 (td, ^*3*^*J*_*H,H*_ = 7.9 Hz, ^*4*^*J*_*H,H*_ = 1.6 Hz, 2H, *m*-C**H**), 7.08 (dt, ^*3*^*J*_*H,H*_ = 9.6 Hz, ^*3*^*J*_*H,H*_ = 3.5 Hz, 2H, *m*-C**H**), 6.81 (td, ^*3*^*J*_*H,H*_ = 7.4, ^*4*^*J*_*H,H*_ = 0.8 Hz, 2H, *o*-C**H**) (Figure S75). ^13^C NMR (75 MHz, CD_2_Cl_2_): δ 160.78 (2C, N = **C**H), 146.27 (2C, *ipso*-**C**), 144.51 (2C, *ipso*-**C**), 135.47 (2C, *m*-**C**), 133.08 (2C, *ipso*-**C**), 132.46 (2C, *ipso*-**C**), 128.00 (2C, *m*-**C**), 123.14 (2C, *m*-**C**), 121.87 (2C, *o*-**C**), 119.16 (2C, *ipso*-**C**), 117.60 (2C, *o*-**C**), 117.46 (2C, *o*-**C**), 112.57 (2C, *m*-**C**) (Figure S76). ^15^N NMR (41 MHz, CD_2_Cl_2_): 301 (2N, **N**=CH), 99 (2N, Ar_2_**N**H) (Figure S80). UV/vis (nm (ε)): 282 (45333), 307 (36666), 394 (15333). HRMS: *m*/*z* = 389.1768. **3a**^**+**^ (*z* = 1) calc. 389.1766. M.p.: 235 °C.

#### Synthesis of H_2_ (Me_2_PhenTAA) (**3b**)

Compound **2b** (1,75 g, 5,1 mmol), *o*-phenylenediamine (606 mg, 5,6 mmol), NaHCO_3_ (428 mg, 5.1 mmol), and activated 3 Å molecular sieves were added to a 500 mL Schlenk flask. To this mixture was added 150 mL dry and degassed toluene. The mixture was stirred at reflux for 10 days, during which the color gradually changed to dark brown. The salts and molecular sieves were filtered off aerobically over Celite and washed with dichloromethane, and the solvents were removed in vacuo. The residue was recrystallized from CH_2_Cl_2_/pentane at −20 °C for 3 h. The tan-yellow crystalline needles were filtered off and washed with pentane (∼300 mL). Yield: 892 mg (42%). ^1^H NMR (400 MHz, CD_2_Cl_2_): δ 12.07 (s, broad, 2H, N**H**), 7.69 (dd, ^*3*^*J*_*H,H*_ = 6.5 Hz, ^*4*^*J*_*H,H*_ = 1.5 Hz, 2H, *o-*C**H**), 7.67 (dd, ^*3*^*J*_*H,H*_ = 7.0 Hz, ^*3*^*J*_*H,H*_ = 3.6 Hz, 2H, *o*-C**H**), 7.39 (dd, ^*3*^*J*_*H,H*_ = 8.5 Hz, ^*4*^*J*_*H,H*_ = 0.9 Hz, 2H, *o*-C**H**), 7.22 (dd, ^*3*^*J*_*H,H*_ = 6.9 Hz, ^*4*^*J*_*H,H*_ = 1.3 Hz, 2H, *m*-C**H**), 7.18 (dd, ^*3*^*J*_*H,H*_ = 6.0 Hz, ^*4*^*J*_*H,H*_ = 3.3 Hz, 2H, *o*-C**H**), 7.15 (dd, ^*3*^*J*_*H,H*_ = 6.0 Hz, ^*3*^*J*_*H,H*_ = 3.5 Hz, 2H, *m*-C**H**), 6.96 (dt, ^*3*^*J*_*H,H*_ = 9.2 Hz, ^*3*^*J*_*H,H*_ = 3.3 Hz, 2H, *m*-C**H**), 6.73 (dd, ^*3*^*J*_*H,H*_ = 7.6 Hz, ^*4*^*J*_*H,H*_ = 1.2 Hz, 2H, *m*-C**H**), 2.41 (s, 6H, C**H**_3_) (Figure S81). ^13^C NMR (101 MHz, CD_2_Cl_2_): δ 168.66 (2C, N=**C**-CH_3_), 147.39 (2C, *ipso*-**C**), 141.01 (2C, *ipso*-**C**), 135.07 (2C, *ipso*-**C**), 131.99 (2C, *m*-**C**), 131.47 (2C, *m*-**C**), 126.01 (2C, *o*-**C**), 124.91 (2C, *o*-**C**), 124.08 (2C, *m-***C**), 121.62 (2C, *m*-**C**), 119.40 (2C, *ipso*-**C**), 116.70 (2C, *m*-**C**), 112.31 (2C, *o*-**C**), 19.10 (2C, **C**H_3_) (Figure S82). UV/vis (nm (ε)): 233 (50666), 265 (38000), 306 (24666), 365 (13333). HRMS: *m*/*z* = 309.1492. *m*/*z* = 415.1927. **3b**^**+**^ (*z* = 1) calc. 415.1923.

### Synthesis and Characterization of Complexes

#### General Procedure B for the Metalation of Free Bases **3a** and **3b**

Free base ligand (1 equiv) and Ni(OAc)_2_·4H_2_O (2 equiv) were added to a Schlenk flask. To this mixture was added DMF (40–45 mL). The reaction was brought to reflux under Ar for 16 h, after which the reaction was cooled, and the solvent was removed in vacuo. The residue was redissolved in CH_2_Cl_2_, filtered over cotton, and washed three times with water. The organic layer was then washed with brine and subsequently dried with Na_2_SO_4_. The Na_2_SO_4_ was filtered off, and the solution was concentrated in vacuo. The product was obtained after recrystallization from CH_2_Cl_2_/pentane.

#### Synthesis of Ni_2_(H_2_PhenTAA) (**4a**)

The reaction was performed according to general procedure A (see above) on a 0.649 mmol scale. Yield: 255.5 mg (88%). ^1^H NMR (500 MHz, CD_2_Cl_2_): δ 8.01 (s, 2H, N = C**H**), 7.80 (d, ^*3*^*J*_*H,H*_ = 8.9 Hz, 2H, *o*-C**H**), 7.58 (dd, ^*3*^*J*_*H,H*_ = 6.0 Hz, ^*3*^*J*_*H,H*_ = 3.3 Hz, 2H, *m*-C**H**), 7.40 (dd, ^*3*^*J*_*H,H*_ = 6.1 Hz, ^*3*^*J*_*H,H*_ = 3.3 Hz, 2H, *o*-C**H**), 7.32 (d, ^*3*^*J*_*H,H*_ = 7.9 Hz, 2H, *o*-C**H**), 7.16 (dt, ^*3*^*J*_*H,H*_ = 9.2 Hz, ^*4*^*J*_*H,H*_ = 1.2 Hz, 2H, *m*-C**H**), 7.05 (dd, ^*3*^*J*_*H,H*_ = 6.2 Hz, ^*3*^*J*_*H,H*_ = 3.2 Hz, 2H, *m*-C**H**), 6.57 (dd, ^*3*^*J*_*H,H*_ = 5.6 Hz, ^*3*^*J*_*H,H*_ = 3.4 Hz, 2H, *o*-C**H**), 6.55 (t, ^*3*^*J*_*H,H*_ = 6.7 Hz, 2H, *m*-C**H**) (Figure S86). ^13^C NMR (126 MHz, CD_2_Cl_2_) δ 150.31 (2C, N=**C**H), 149.83 (2C, *ipso*-**C**), 147.82 (2C, *ipso*-**C**), 145.17 (2C, *ipso*-**C**), 135.39 (2C, *o*-**C**), 133.19 (2C, *m*-**C**), 127.80 (2C, *m*-**C**), 123.75 (2C, *ipso*-**C**), 120.07 (2C, *o*-**C**), 119.38 (2C, *o*-**C**), 117.33 (2C, *m*-**C**), 116.39 (2C, *m*-**C**), 115.26 (2C, *o*-**C**) (Figure S87). ^15^N NMR (51 MHz, CD_2_Cl_2_): 189 (2N, **N**=CH) (Figure S91). HRMS: *m*/*z* = 444.0895. **4a**^**+**^ (*z* = 1) calc. 444.0885.

#### Synthesis of Ni_2_(Me_2_PhenTAA) (**4b**)

The reaction was performed according to general procedure A (see above) on a 0.48 mmol scale. Yield: 204 mg (90%). ^1^H NMR (400 MHz, CD_2_Cl_2_): δ 7.57–7.55 (m, 2H, *o*-C**H**), 7.55–7.51 (m, 2H, *o* -C**H**), 7.27 (dt, ^*3*^*J*_*H,H*_ = 9.4 Hz, ^*3*^*J*_*H,H*_ = 3.4 Hz, 2H, *m*-C**H**), 6.97 (ddd, ^*3*^*J*_*H,H*_ = 9.2 Hz, ^*3*^*J*_*H,H*_ = 6.6 Hz, ^*4*^*J*_*H,H*_ = 1.6 Hz, 2H, *m*-C**H**), 6.92–6.86 (m, 2H, *m*-C**H**), 6.86–6.80 (m, 2H, *o*-C**H**), 6.50 (t, ^*3*^*J*_*H,H*_ = 6.1 Hz, ^*3*^*J*_*H,H*_ = 3.4 Hz, 2H, *m*-C**H**), 6.36 (t, ^*3*^*J*_*H,H*_ = 7.5 Hz, 2H, *m*-C**H**), 2.52 (s, 6H, C**H**_3_) (Figure S92). ^13^C NMR (101 MHz, CD_2_Cl_2_): δ 162.22 (2C, N = **C**CH_3_), 148.76 (2C, *ipso*-**C**), 148.20 (2C, *ipso*-**C**), 147.75 (2C, *ipso*-**C**), 132.05 (2C, *o*-**C**), 132.02 (2C, *m*-**C**), 126.07 (2C, *m*-**C**), 125.83 (2C, *ipso*-**C**), 123.75 (2C, *o*-**C**), 119.63 (2C, *m*-**C**), 118.36 (2C, *o*-**C**), 118.02 (2C, *o-***C**), 114.75 (2C, *m*-**C**), 20.16 (2C, **C**H_3_) (Figure S93). ^15^N NMR (41 MHz, CD_2_Cl_2_): 187 (2N, **N**=CH) (Figure S97). HRMS: *m*/*z* = 472.1201. **4b**^**+**^ (*z* = 1) calc. 472.1198.

#### Synthesis of Ni_2_(Ph_2_PhenTAA) (**4c**)

Compound **2c** (1000 mg, 2.13 mmol), *o*-phenylenediamine (230.5 mg, 2.13 mmol), and Ni(OAc)_2_·4H_2_O (531 mg, 2.13 mmol) were added to a Schlenk flask. To this was added xylene (30 mL), which was connected with a Dean–Stark trap. The trap was filled with 11.5 mL of xylene to the top, and the apparatus was connected to the reaction flask under Ar. The mixture was refluxed vigorously for 7 days, after which it was cooled down, and the solvent removed in vacuo. The dark black-purple residue was redissolved in CH_2_Cl_2_/petroleum ether (40/60) (1:1 v/v) and passed over a short silica plug. The first fraction is the product (*R*_*F*_ = 0.8 (CH_2_Cl_2_)), the second fraction is compound **2c** (*R*_*F*_ = 0.3 (CH_2_Cl_2_)), and an unidentified greyish spot remains on the baseline. The first fraction was evaporated in vacuo and recrystallized from CH_2_Cl_2_/MeOH. Yield: 403.5 mg (32%). ^1^H NMR (400 MHz, CD_2_Cl_2_): δ 7.67 (d, ^*3*^*J*_*H,H*_ = 8.7 Hz, 1H, *o*-C**H**), 7.56–7.47 (m, 4H, *m*-C**H**), 7.43–7.39 (m, 2H, *p*-C**H**), 7.38 (ddd, ^*3*^*J*_*H,H*_ = 9.4 Hz, ^*3*^*J*_*H,H*_ = 4.0 Hz, ^*3*^*J*_*H,H*_ = 3,9 Hz, 2H, *o*-C**H**), 7.35–7.30 (m, 4H, *o*-C**H**), 6.98 (ddd, ^*3*^*J*_*H,H*_ = 9.1 Hz, ^*3*^*J*_*H,H*_ = 6.6 Hz, ^*4*^*J*_*H,H*_ = 1.3 Hz, 2H, *m*-C**H**), 6.81 (dd, ^*3*^*J*_*H,H*_ = 8.6 Hz, ^*4*^*J*_*H,H*_ = 1.3 Hz, 2H, *o*-C**H**), 6.62 (s, 2H, *m*-C**H**), 6.22 (t, ^*3*^*J*_*H,H*_ = 7.4 Hz, 2H, *m*-C**H**), 6.17 (ddd, ^*3*^*J*_*H,H*_ = 9.7 Hz, ^*3*^*J*_*H,H*_ = 4.2 Hz, ^*3*^*J*_*H,H*_ = 3.8 Hz, 2H, *m*-C**H**), 5.70 (ddd, ^*3*^*J*_*H,H*_ = 9.6 Hz, ^*3*^*J*_*H,H*_ = 4.3 Hz, ^*3*^*J*_*H,H*_ = 3.7 Hz, 2H, *o*-C**H**) (Figure S98). ^13^C NMR (101 MHz, CD_2_Cl_2_): 162.45 (2C, N = **C**Ph), 147.80 (2C, *ipso*-**C**), 137.57 (2C, *ipso*-**C**), 135.60 (2C, *o*-**C**), 132.56 (2C, *m*-**C**), 130.78 (2C, *ipso*-**C**), 130.09 (4C, *o*-**C**), 129.99 (4C, *m*-**C**), 129.66 (2C, *p*-**C**), 129.15 (2C, *ipso*-**C**), 128.08 (2C, *ipso*-**C**), 125.22 (2C, *m*-**C**), 123.44 (2C, *o*-**C**), 120.04 (2C, *m*-**C**), 118.82 (2C, *o*-**C**), 117.52 (2C, *o*-**C**), 114.58 (2C, *m*-**C**) (Figure S99). ^15^N NMR (51 MHz, C_6_D_6_): 185 (2N, **N**=CPh), 90 (2N, Ar_2_**N**) (Figure S105). HRMS: *m*/*z* = 596.1515. **4a**^**+**^ (*z* = 1) calc. 596.1511.

### Computational Studies

DFT geometry optimizations were performed without simplifications on full atomic models using TURBOMOLE 7.4.1^23^ coupled to the PQS Baker optimizer^24^ via the BOpt package.^25^ Unless mentioned otherwise, convergence criteria (scfconv = 7) were used on a m4 grid using Grimme’s version 3 zero-damping dispersion correction.^26^ The multipole accelerated resolution of identity (MARI-J) was used for all TURBOMOLE calculations.^27^ For a description of the functional and basis sets used, see below. All minima, without imaginary frequencies, were characterized by calculating the analytical Hessian matrix. Energy output generated in Hartree units was converted to kcal·mol^–1^ by multiplication with 627.51. Grimme’s dispersion corrections were added to compensate for the underestimation of metal–ligand interactions from uncorrected DFT calculations. EPR parameters were calculated with the ORCA 4.2.1^28^ software package at the B3LYP (ref (29))/ZORA-TZVPP (ref (30)) level using the coordinates from the structures optimized in TURBOMOLE as the input. Graphical representations of structures and visualization of orbitals were generated using IboView v20150427.^31^ Spin density plots were generated using IQMol 2.9.2 (http://iqmol.org/). TD–DFT calculations were performed with the ORCA 4.2.1^28^ software package at the B3LYP (ref (29))/def2-TZVPP (ref (30)) level of theory with the Tamm-Damcoff approximation.^32^ Additionally, the CPCMC solvation model was used with CH_2_Cl_2_ for neutral and oxidized species (ε = 9.08, refractive index 1.424) and THF for reduced species (ε = 7.25, refractive index 1.407). For each computed spectrum, 100 roots were evaluated. For energetic values, spin densities, and xyz coordinates, see the Supporting Information.

## Supporting Information Available

Experimental details; synthetic procedures; EPR, NMR, UV–Vis, *in-situ* ATR–FT–IR and ESI^+^–MS spectroscopic data as well as DFT calculations. Crystallographic details for CCDC 2277309 (**2a**), 2277308 (**2c**), 2277307 (**3a**), 2300376 (**4a**), and 2277310 (**4c**) (PDF)
