# Supplementary material for: PhenTAA: A Redox-Active N4-Macrocyclic Ligand Featuring Donor and Acceptor Moieties
Source: Inorg Chem. 2024 Jan 12;63(4):1974–87. doi: 10.1021/acs.inorgchem.3c03708 (PMC10828995; doi:10.1021/acs.inorgchem.3c03708)
Supplement: Supplementary file 1 — ic3c03708_si_001.pdf [file ic3c03708_si_001.pdf]

Supporting Information belonging to the paper:

PhenTAA: A Redox-Active N_4-Macrocyclic Ligand Featuring Donor and Acceptor Moieties

Roel F.J. Epping[†], Felix J. de Zwart[†], Nicolaas P. van Leest[†], Jarl Ivar van der Vlugt[†,‡], Maxime A. Siegler[§], Simon Mathew[†], Joost N.H. Reek[†] and Bas de Bruin[†]*

[†]Homogeneous, Supramolecular Catalysis and Bio-Inspired Catalysis Group, Van 't Hoff Institute for Molecular Sciences (HIMS), University of Amsterdam, Science Park 904, 1098 XH Amsterdam, The Netherlands

[‡]Current address: Bioinspired Coordination Chemistry & Homogeneous Catalysis Group, Institute of Chemistry, Carl von Ossietzky University Oldenburg, Carl-von-Ossietzky-Strasse 9-11. 26129 Oldenburg, Germany

[§]Department of Chemistry, Johns Hopkins University, Baltimore, Maryland 21218, United States

Table of contents

General considerations S2
Experimental data S5
Synthesis of the complexes S5
X-Ray Diffraction S12
Bond-length & HOMA analysis S14
UV/Vis Spectra S17
in-situ ATR–FT–IR spectra: S18
Electrochemistry S19
Cyclic voltammetry S19
Spectroelectrochemistry S26
EPR Spectra S36
Mass Spectra S45
NMR Spectra S51
Computational data S75
Density Functional Theory Calculations S75
Calculated <s²> values, spin states and energies for optimized structures S75
Time-dependent Density Functional Theory Calculations S84
Mulliken spin density plots S131
XYZ coordinates of all optimized DFT calculated structures S138
References in the Supporting Information S171

1. General Considerations

Chemicals and solvents

All reactions were carried out under argon using standard Schlenk techniques or under an inert atmosphere in a N_2-filled glovebox, unless noted otherwise. All chemicals were of commercial grade and used without further purification, unless noted otherwise. All solvents used were pre-dried using either a Solvent Purification System (SPS) from MBraun (MB SPS-800, with standard MBraun drying columns) or were dried and distilled from sodium (PhMe/pentane), sodium/benzophenone (THF/Et_2O) or CaH_2 (CH_2Cl_2, MeOH). All solvents were further dried/stored on activated 3Å molecular sieves and degassed by sparging with argon unless mentioned otherwise.

NMR spectroscopy

NMR spectra (^1H and ^{13}C) were measured on a Bruker DRX 500, Bruker AMX 400, Bruker DRX 300 or on a Varian Mercury 300 spectrometer at RT unless noted otherwise. Chemical shifts were reported in ppm with respect the solvent peak of $SiMe_4$.[1] Individual peaks are reported as: chemical shift (in ppm) (multiplicity (s: singlet, d: doublet, t: triplet, q: quartet, m: multiplet), integration, coupling constant (in Hz).

EPR spectroscopy

EPR spectra were recorded on a Bruker EMX X-band spectrometer equipped with an ER 4112HV-CF100 He cryostat. Simulations of the EPR spectra were performed by iteration of the anisotropic g-values, hyperfine coupling interactions and line widths using EasySpin,[2] via the cwEPR 3.2 GUI.[3]

UV/Vis spectroscopy

UV/Vis spectra were recorded on a Hewlett Packard 8453 or a double beam Shimadzu UV-2600 spectrometer in a 1.0 cm Teflon screw-cap quartz cuvette under inert atmosphere using the solvent as a background. The cuvette was custom outfitted with an extra 10 mL round bottom flask for mixing purposes.

Electrochemical measurements

Cyclic voltammograms were recorded using a 663 VA stand with a PGSTAT302N potentiostat (Methrohm/Autolab) in a single-cell three electrode system with a glassy carbon working electrode, Pt auxiliary electrode and leak-free Ag/AgCl (3.5 M KCl) reference electrode. All potentials are reported vs. ferrocene/ferrocenium (Fc/Fc^+) by adding Fc to the mixture in an equimolar fashion under argon or nitrogen.

Spectroelectrochemical measurements

Spectroelectrochemical measurements were performed using a standard OTTLE cell[4] (l = 0.2 mm)with a Pt grid working electrode, Pt auxiliary electrode and a Ag wire reference electrode, coupled to an Autolab PGSTAT302N potentiostat and double beam Shimadzu UV-2600 spectrometer.

Cold-Electronspray-ionization MS (CSI–MS)

Mass spectra were collected on a HR–ToF Bruker Daltonik GmbH (Bremen, Germany) Impact II, an ESI–ToF–MS capable of resolution of at least 40000 FWHM. Detection was in positive-ion mode and the source voltage was between 4–6 kV. The sample was introduced with a syringe pump at a flow rate of 18 μl hr^{-1}. The drying gas (N_2) was held between 0°C and –40°C and the spray gas was held

between 10°C and –35°C. The machine was calibrated prior to every experiment via direct infusion of a TFA-Na solution, which provided a *m/z* range of singly charged peaks up to 3500 Da in both ion modes. Software acquisition Compass 2.0 for Otof series. Software processing m-mass and Compass 2.0.

Single-crystal X-ray diffraction

X-ray diffraction data of **2a**, **2c**, **3a** and **4c** were measured on a Bruker D8 Quest Eco diffractometer using graphite monochromated (Triumph) Mo Kα radiation (λ = 0.71073 Å) and a CMOS Photon 100 detector (**2c** & **3a**) or a CPAD Photon III C14 detector (**2a** & **4c**). The sample was cooled with N_2 to 100 K (**2a** & **4c**) or 150 K (**2c** & **3a**) with a Cryostream 700 (Oxford Cryosystems). Intensity data were integrated using the SAINT software.[5] Absorption correction and scaling was executed with SADABS.[6] The structures were solved using intrinsic phasing with the program SHELXT 2018/2.[7] Least-squares refinement was performed with SHELXL-2018/3.[8] All non-hydrogen atoms were refined with anisotropic displacement parameters. The hydrogen atoms were introduced at calculated positions with a riding model. The structure of **4c** contained voids (total solvent accessible vol. = 174 Å^3, 42 e$^-$), containing residual solvent (dichloromethane) within the asymmetric unit that could not be refined reliably. Using the SQUEEZE procedure in PLATON[9] (110423), the 42 electrons per unit cell were assigned to the presence of 0.25 × CH_2Cl_2 (42 e-/molecule) solvent molecules within the asymmetric unit (Z=4).

The resulting CIF files reveal no A-level alerts. The crystal data for all species are summarized as shown in **Table S1.** Crystal structure and determination data of **2a, 2c, 3a, 4a** & **4c.** The X-ray crystallographic data were deposited at the Cambridge Crystallographic Data Centre (CCDC) under the deposition numbers CCDC 2277309 (2a) 2277308 (2c) 2277307 (3a) 2277310 (4c), 2300376 (4a). These data can be obtained free of charge from The Cambridge Crystallographic Data Centre via www.ccdc.cam.ac.uk/structures

DFT calculations

DFT geometry optimizations were performed without simplifications on full atomic models using TURBOMOLE 7.4.1[10] coupled to the PQS Baker optimizer[11] via the BOpt package.[12] Unless mentioned otherwise, convergence criteria (scfconv = 7) were used on a m4 grid using Grimme's version 3 zero-damping dispersion correction.[13] The multipole accelerated resolution of identity (MARI-J) was used for all TURBOMOLE calculations.[14] For a description of the functional and basis sets used, see below. All minima, without imaginary frequencies, were characterized by calculating the analytical Hessian matrix. Energy output generated in Hartree units was converted to kcal·mol^{-1} by multiplication with 627.51. Grimme's dispersion corrections were added to compensate for the underestimation of metal-ligand interactions from uncorrected DFT calculations.

Geometric minima:

To decrease calculation times, the following approach was used:

1) All input structures were generated as protein database files (.pdb).
2) All intermediates of relevance and their geometric variances were pre-optimized at the ri-DFT BP86[15]/def2-SVP[16] level of theory followed by a Hessian matrix calculation at the doublet or triplet spin surface for non-integer and integer spin systems respectively.
3) The structures obtained in step 2, were further optimized at the BP86/def2-TZVP[16] level of theory.
4) The optimized structures were calculated at varying spin states at the BP86/def2-TZVP level of theory. For non-integer spin systems, the additional quartet and sextet spin states were calculated. For integer spin systems, the additional closed-shell singlet, open-shell singlet and quintet spin states were calculated.

In case of broken-symmetry solutions, corrected broken-symmetry energies were estimated from the energy (E_S) of the optimized single-determinant broken symmetry solution and the energy (E_{S+1}) from a separate unrestricted single-point calculation of the high spin-state at the same level of theory. The following approximate correction formula (1) was used:[17]

$$E = \frac{S_{S+1}^2 \cdot E_S - S_S^2 \cdot E_{S+1}}{S_{S+1}^2 - S_S^2}$$

EPR parameters were calculated with the ORCA 4.2.1[18] software package at the B3LYP[19]/ZORA-TZVPP[20] level using the coordinates from the structures optimized in TURBOMOLE as the input. Graphical representations of structures and visualization of orbitals were generated using IboView v20150427.[21] Spin density plots were generated using IQMol 2.9.2 (http://iqmol.org/).

TD–DFT calculations

TD–DFT calculations were performed with the ORCA 4.2.1[18] software package at the B3LYP[19]/def2-TZVPP[20] level of theory with the Tamm-Damcoff approximation[22]. Additionally, the CPCMC solvation model was used with CH_2Cl_2 for neutral and oxidized species (ε = 9.08, refractive index 1.424) and THF for reduced species (ε = 7.25, refractive index 1.407). For each computed spectrum 100 roots were evaluated.

Synthesis of the ligands and complexes

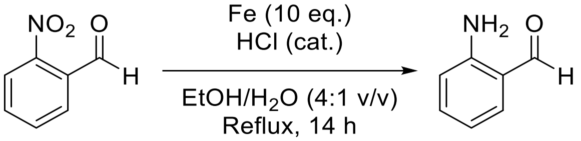

2-aminobenzaldehyde (1a)

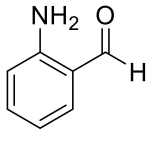

Compound **1a** was prepared according to a modified literature procedure[23]. To a 1 L round-bottom flask was added Fe (55.85 g, 1 mol, 10 eq.), 2-nitrobenzaldehyde (15.11 g, 0.1 mol, 1 eq.), 300 mL absolute EtOH and 75 mL H_2O. This mixture was bubbled through with Ar for 10 min after which HCl (0,75 mL; 37%) was added. The mixture was brought to reflux under Ar for 14 h. After cooling down, the mixture was filtered over Celite until no Fe remained in the mixture and washed with EtOH until effluent was colorless. The filtrate was concentrated in vacuo, subsequently extracted with CH_2Cl_2/H_2O, washed with brine, dried with Na_2SO_4, filtered over cotton and evaporated in vacuo until a yellow oil remained. Next, the crude product was purified via column chromatography (eluent: CH_2Cl_2) until the eluent became colorless. The pure fractions were identified via TLC and evaporated in vacuo (R_F = 0.2). Yield: 10 g (83%). ^{1}H-NMR (400 MHz, DMSO-d_6): δ 9.81 (s, 1H; C*H*O), 7.51 (d, $^3J_{H,H}$ = 7.2 Hz, 1H; *o*-Ar*H*), 7.30 (t, *J* = 7.7 Hz, 1H), 7.10 (s, 2H; *o*-N*H*$_2$), 6.76 (d, *J* = 8.5 Hz, 1H), 6.63 (t, *J* = 7.3 Hz, 1H). The reported data is in accordance with literature.[23]

Notes:

1) The product is unstable over time and polymerizes! Consequently, all of it is immediately used in the synthesis of **2a**.
2) When concentrating the filtrate, don't evaporate all the solvent as the product has an azeotrope with the remaining water.
3) A short silica plug can also be used instead of a column as it will get rid of the majority of impurities. This does not seem to significantly affect the yields of the next synthetic step.

General procedure A towards compounds 2a-c

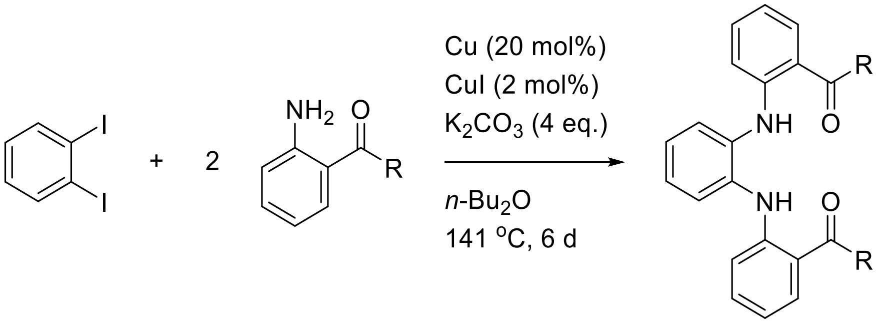

n-Bu_2O (200 mL) was filtered over activated basic alumina and to this was added *o*-diiodobenzene (1 eq.) and amine (2.2 eq.). The solution was subsequently sparged with argon for at least 30 min. To a separate 1 L Schlenk flask was added 500 mesh Cu powder (20 mol%), CuI (2 mol%) and K_2CO_3 (4 eq.) and this was subsequently cycled three times with Ar/vacuum. To this Schlenk flask the *n*-Bu_2O solution was added, and this was brought to reflux for 6 days. After cooling, the salts were filtered off over a short silica plug, washed with CH_2Cl_2 until the effluent was colorless and the solvent was

removed in vacuo. The crude mixture was either recrystallized from CH_2Cl_2/pentane or purified via column chromatography (CH_2Cl_2/petroleum ether (40-60) gradient). The pure fractions were identified via TLC and evaporated in vacuo. TLCs were taken in CH_2Cl_2 and R_F-values are reported as such.

General notes:

1) Small amounts of copper might insert into the product, with very comparable R_F-values and can be identified as bright-red spots on TLC (compared to bright-yellow for the metal-free product). These can be separated through careful column chromatography or strong acidic extraction in concentrated HCl. Do not use concentrated, dry acids like H_2SO_4 as the products will react further.
2) The products have a high affinity for silica and will exhibit significant tailing on the column. Depending on the brand of silica used, between 1–2 liters of solvent/mmol is generally required.
3) After column chromatography, the products tend to be highly viscous liquids. These can easily be recrystallized either from CH_2Cl_2/pentane or hot MeOH. Due to aerobic decomposition at elevated temperatures, hot recrystallizations should be performed under inert conditions.

***N,N'*-(1,2-phenylenediamino)-bis(2-aminobenzaldehyde) (2a)**

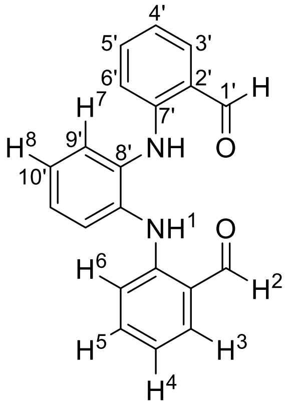

The reaction was performed according to general procedure A (see above) on a 37.5 mmol scale. The product was isolated after column chromatography (CH_2Cl_2/petroleum ether (40–60) gradient). Gradient: 50% CH_2Cl_2/PE (fractions 1–30); 75% CH_2Cl_2/PE (fractions 31–50); 100% CH_2Cl_2/PE (fractions 51–80); 25% EtOAc/75% CH_2Cl_2 (fractions 81–all color eluted). The first fraction is the mono-coupled product (R_F = 0.9), the second fraction compound **2a** (R_F = 0.6) and the final fraction 2-aminobenzaldehyde starting material (R_F = 0.2). The pure fractions were identified via TLC and evaporated in vacuo. Yield: 6.64 g (56%). Crystals suitable for X-ray diffraction were obtained by via layering of a concentrated CH_2Cl_2 solution with pentane. ^{1}H-NMR (400 MHz, CD_2Cl_2): δ 9.84 (s, broad, 2H, *H-1*, N**H**), δ 9.81 (s, 2H, *H-2*, C**H**O), δ 7.54 (dd, $^3J_{H,H}$ = 7.7 Hz, $^4J_{H,H}$ = 1.6 Hz, 2H, *H-3, o*-C**H**), 7.50 (dd, $^3J_{H,H}$ = 5.9 Hz, $^4J_{H,H}$ = 3.6 Hz, 2H, *H-8, m*-C**H**), 7.32 (td, $^3J_{H,H}$ = 8.6, $^4J_{H,H}$ =1.7 Hz, 2H, *H-5, m*-C**H**), 7.22 (dd, $^3J_{H,H}$ = 6.0, $^3J_{H,H}$ = 3.5 Hz, 2H, *H-7, o*-C**H**), 7.01 (d, $^3J_{H,H}$ = 8.5 Hz, 2H, *H-6, o*-C**H**), 6.83 (td, $^3J_{H,H}$ = 7.9, $^4J_{H,H}$ = 0.8 Hz, 2H, *H-4, m*-C**H**). ^{13}C-NMR (126 MHz, CD_2Cl_2): δ 194.61 (2C, *C-1'*, **C**HO), 147.93 (2C, *C-7', ipso*-**C**), 136.88 (2C, *C-3', o*-**C**), 135.83 (2C, *C-5', m*-**C**), 134.42 (2C, *C-8', ipso*-**C**), 125.83 (2C, *C-9', o*-**C**), 125.34(2C, *C-10', m*-**C**), 120.34(2C, *C-2', ipso*-**C**), 117.91(2C, *C-4', m*-**C**), 113.48(2C, *C-6', o*-**C**). HRMS: m/z = 299.1190. **2a**$^+$ (z = 1) calc. 317.1290. [**2a**-H_2O]$^+$ (z = 1) calc. 299.1184 (σ = 2.005895 ppm).

***N,N'*-(1,2-phenylenediamino)-bis(2-aminoacetophenone) (2b)**

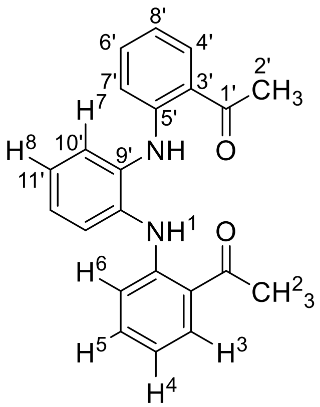

The reaction was performed according to general procedure A (see above) on a 5 mmol scale. The product was isolated after recrystallization from CH_2Cl_2/pentane. Yield: 735 mg (43%). ^{1}H-NMR (500 MHz, CD_2Cl_2): δ 10.31 (s, broad, 2H, *H-1*, N**H**), 7.77 (dd, $^3J_{H,H}$ = 8.1 Hz, $^4J_{H,H}$ = 1.6 Hz, 2H, *H-3*, *o*-C**H**), 7.44 (dd, $^3J_{H,H}$ = 5.9, $^4J_{H,H}$ = 3.6 Hz, 2H, *H-8*, *m*-C**H**), 7.25 (ddd, $^3J_{H,H}$ = 8.6, $^3J_{H,H}$ = 7.0, $^4J_{H,H}$ = 1.6 Hz, 2H, *H-5*, *m*-C**H**), 7.15 (dd, $^3J_{H,H}$ = 6.0, $^4J_{H,H}$ = 3.5 Hz, 2H, *H-7*, *o*-C**H**), 7.02 (dd, $^3J_{H,H}$ = 8.5, $^4J_{H,H}$ = 1.1 Hz, 2H, *H-6*, *o*-C**H**), 6.72 (ddd, $^3J_{H,H}$ = 8.1, $^3J_{H,H}$ = 7.0, $^4J_{H,H}$ = 1.2 Hz, 2H, *H-4*, *m*-C**H**), 2.54 (s, 6H, *H-2*, C**H**$_3$). ^{13}C-NMR (126 MHz, CD_2Cl_2): δ 201.52 (2C, *C-1'*, **C**OCH$_3$), 148.13 (2C, *C-8'*, *ipso*-**C**), 135.16 (2C, *C-9'*, *ipso*-**C**), 134.78 (2C, *C-6'*, *m*-**C**), 132.84 (2C, *C-4'*, *m*-**C**), 125.29 (2C, *C-10'*, *o*-**C**), 125.15 (2C, *C-11'*, *m*-**C**), 120.14 (2C, *C-3'*, *ipso*-**C**), 117.23 (2C, *C-5'*, *m*-**C**), 114.93 (2C, *C-7'*, *o*-**C**), 28.40 (2C, *C-2'*, **C**H$_3$). HRMS: m/z = 345.1608. **2b**$^+$ (z = 1) calc. 345.1603 (σ = 1.448602 ppm).

Notes:

1) Since all starting materials and the *mono*-coupled byproduct are liquid at room temperature, the easier synthetic step is recrystallization. However, for maximum yield, column chromatography can be used to extract the rest from the filtrate.

***N,N'*-(1,2-phenylenediamino)-bis(2-aminobenzophenone) (2c)**

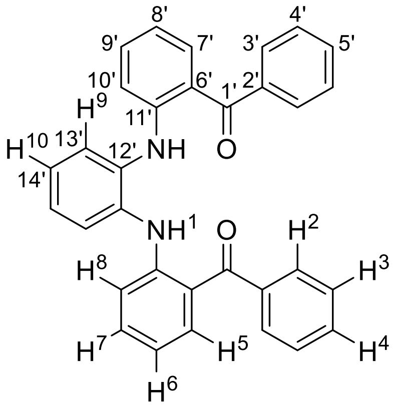

The reaction was performed according to general procedure A (see above) on a 5 mmol scale. The product was isolated after column chromatography (CH_2Cl_2/petroleum ether (40–60) gradient) (R_F = 0.3 (CH_2Cl_2)). The pure fractions were identified via TLC and evaporated in vacuo. Yield: 1.91 g (81%). Crystals suitable for X-ray diffraction were obtained by slow cooling of a hot, concentrated solution of **2c** in MeOH under inert atmosphere ^{1}H-NMR (500 MHz, CD_2Cl_2): δ 9.88 (s, broad, 2H, *H-1*, N**H**), 7.61 (dt, $^3J_{H,H}$ = 7.5 Hz, $^4J_{H,H}$ = 1.5 Hz, 4H, *H-2*, *o*-C**H**), 7.55 (tt, $^3J_{H,H}$ = 7.4 Hz, $^4J_{H,H}$ = 2.0 Hz, 2H, *H-4*, *p*-C**H**), 7.51 (dd, $^3J_{H,H}$ = 5.9 Hz, $^3J_{H,H}$ = 3.5 Hz, 2H, *H-9*, *o*-C**H**), 7.47 (m, 2H, *H-5*, *o*-C**H**), 7.45 (m, 4H, *H-3*, *m*-C**H**), 7.27 (td, $^3J_{H,H}$ = 7.5 Hz, $^4J_{H,H}$ = 1.1 Hz, 2H, *H-7*, *m*-C**H**), 7.19 (dt, $^3J_{H,H}$ = 9.6 Hz, $^3J_{H,H}$ = 3.7 Hz, 2H, *H-10*, *m*-C**H**) 7.13 (dd, $^3J_{H,H}$ = 7.8 Hz, $^4J_{H,H}$ = 0.6 Hz, 2H, *H-8*, *o*-C**H**) 6.88 (td, $^3J_{H,H}$ = 7.4 Hz, $^4J_{H,H}$ = 1.1 Hz, 2H, *H-6*, *m*-C**H**). ^{13}C-NMR (126 MHz, CD_2Cl_2): δ 199.37 (2C, *C-1'*, **C**O), 148.21 (2C, *C-11'*, *ipso*-**C**), 140.35 (2C, *C-2'*, *ipso*-**C**), 135.12 (2C, *C-7'*, *o*-**C**), 134.81 (2C, *C-6'*, *ipso*-**C**), 134.47 (2C, *C-9'*, *o*-**C**), 131.83 (2C, *C-5'*, *p*-**C**), 130.04 (4C, *C-3'*, *o*-**C**), 128.52 (4C, *C-4'*, *m*-**C**), 125.17 (2C, *C-14'*, *m*-**C**), 124.61 (2C, *C-13'*, *o*-**C**), 120.83 (2C, *C-12'*, *ipso*-**C**), 117.27 (2C, *C-8'*, *m*-**C**), 115.27 (2C, *C-10'*, *o*-**C**). HRMS: m/z = 468.1984. **2c**$^+$ (z = 1) calc. 468.1838.

$H_2(H_2PhenTAA)$ (3a)

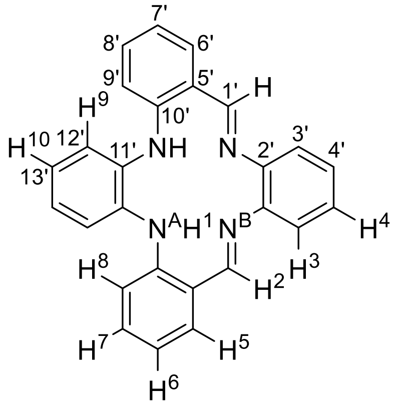

Compound **2a** (1.00 g, 3.16 mmol, 1 eq.), *o*-phenylene diamine (342 mg, 3.16 mmol, 1 eq.), $Zn(OAc)_2 \cdot 2\ H_2O$ (693.3 mg, 3.16 mmol, 1 eq.) were added to a flame-dried 500 mL Schlenk flask and subsequently cycled three times with Ar/vacuum. To a separate Schlenk flask, methanol (150 mL) was filtered over activated basic alumina and added, sparged with Ar for at least 30 min and subsequently added to the initial Schlenk flask. The mixture was stirred at reflux overnight under Ar, during which orange powder precipitated out of solution. The product was filtered off aerobically, washed with cold methanol and dried in vacuo. Yield: 1.11 g (90%). Crystals suitable for X-ray diffraction were obtained by via layering of a concentrated CH_2Cl_2 solution with cyclopentane. ^{1}H-NMR (400 MHz, CD_2Cl_2): δ 12.20 (s, broad, 2H, *H-1*, N**H**), 8.73 (s, 2H, *H-2*, N=C**H**), 7.75 (dt, $^3J_{H,H}$ = 9.6 Hz, $^4J_{H,H}$ = 3.6 Hz, 2H, *H-9*, *o*-C**H**), 7.60 (d, $^3J_{H,H}$ = 8.6 Hz, 2H, *H-6*, *m*-C**H**), 7.45 (dd, $^3J_{H,H}$ = 7.8 Hz, $^4J_{H,H}$ = 1.5 Hz, 2H, *H-5*, *o*-C**H**), 7.39 – 7.35 (m, 2H, *H-3*, *o*-C**H**), 7.35 – 7.33 (m, 2H, *H-4*, *m*-C**H**), 7.30 (td, $^3J_{H,H}$ = 7.9 Hz, $^4J_{H,H}$ = 1.6 Hz, 2H, *H-7*, *m*-C**H**), 7.08 (dt, $^3J_{H,H}$ = 9.6 Hz, $^3J_{H,H}$ = 3.5 Hz, 2H, *H-10*, *m*-C**H**), 6.81 (td, $^3J_{H,H}$ = 7.4, $^4J_{H,H}$ = 0.8 Hz, 2H, *H-8*, *o*-C**H**). ^{13}C-NMR (75 MHz, CD_2Cl_2): δ 160.78 (2C, *C-1'*, N=CH), 146.27 (2C, *C-10'*, *ipso*-**C**), 144.51 (2C, *C-2'*, *ipso*-**C**), 135.47 (2C, *C-6'*, *m*-**C**), 133.08 (2C, *C-11'*, *ipso*-**C**), 132.46 (2C, *C-8'*, *ipso*-**C**), 128.00 (2C, *C-4'*, *m*-**C**), 123.14 (2C, *C-13'*, *m*-**C**), 121.87 (2C, *C-12'*, *o*-**C**), 119.16 (2C, *C-5'*, *ipso*-**C**), 117.60 (2C, *C-9'*, *o*-**C**), 117.46 (2C, *C-3'*, *o*-**C**), 112.57 (2C, *C-7'*, *m*-**C**). ^{15}N-NMR (41 MHz, CD_2Cl_2): 301 (2N, *N-B*, **N**=CH), 99 (2N, *N-A*, Ar_2**N**H). UV/Vis (nm (ε)): 282 (45333), 307 (36666), 394 (15333). HRMS: *m/z* = 389.1768. **3a**$^+$ (*z* = 1) calc. 389.1766 (σ = 0.513906 ppm). M.p.: 235 °C.

Notes:

1) The methanol needs to be slightly wet (~200-2000 ppm H_2O), otherwise the product might not crash out and the yield/purity will be much lower. If the methanol is too wet, the reaction will be unfavorable and yield is considerably lower. In scaling up or down, the amount of methanol:**2a** is lower as scale increases and vice versa.

2) The reaction is performed under inert conditions to prevent oxidation of the starting aldehyde or *ortho*-phenylene diamine at elevated temperatures. However, the product can be handled in air without any difficulties.

3) If the precipitate is quite fine, the powder can be excessively static when dry.

$H_2(Me_2PhenTAA)$ (3b)

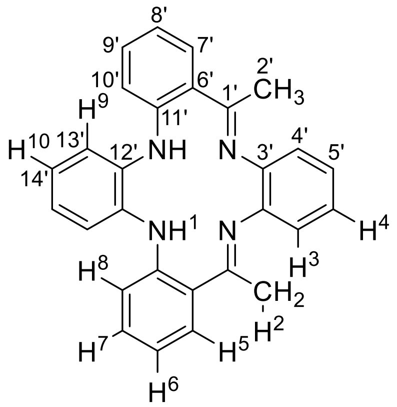

Compound **2b** (1,75 g, 5,1 mmol, 1 eq.), *o*-phenylene diamine (606 mg, 5,6 mmol, 1.1 eq.), $NaHCO_3$ (428 mg, 5.1 mmol, 1 eq.) and activated 3Å molecular sieves were added to a flame-dried 500 mL Schlenk flask and subsequently cycled three times with Ar/vacuum. To this was added 150 mL dry and degassed toluene (150 mL). The mixture was stirred at reflux for 10 days under Ar, during which the color gradually changed to dark brown. The salts and molecular sieves were filtered off aerobically over Celite, washed with dichloromethane and the solvents were removed in vacuo. The residue was recrystallized from CH_2Cl_2/pentane at –20 °C for 3 h. The tan-yellow crystalline needles were filtered off and washed excessively with pentane (~300 mL). Yield: 892 mg (42%). ^{1}H-NMR (400 MHz, CD_2Cl_2): δ 12.07 (s, broad, 2H, *H-1*, N**H**), 7.69 (dd, $^3J_{H,H}$ = 6.5 Hz, $^4J_{H,H}$ = 1.5 Hz, 2H, *H-5*, *o*-C**H**), 7.67 (dd, $^3J_{H,H}$ = 7.0 Hz, $^3J_{H,H}$ = 3.6 Hz, 2H, *H-9*, *o*-C**H**), 7.39 (dd, $^3J_{H,H}$ = 8.5 Hz, $^4J_{H,H}$ = 0.9 Hz, 2H, *H-8*, *o*-C**H**), 7.22 (dd, $^3J_{H,H}$ = 6.9 Hz, $^4J_{H,H}$ = 1.3 Hz, 2H, *H-7*, *m*-C**H**), 7.18 (dd, $^3J_{H,H}$ = 6.0 Hz, $^4J_{H,H}$ = 3.3 Hz, 2H, *H-3*, *o*-C**H**), 7.15 (dd, $^3J_{H,H}$ = 6.0 Hz, $^3J_{H,H}$ = 3.5 Hz, 2H, *H-10*, *m*-C**H**), 6.96 (dt, $^3J_{H,H}$ = 9.2 Hz, $^3J_{H,H}$ = 3.3 Hz, 2H, *H-4*, *m*-C**H**), 6.73 (dd, $^3J_{H,H}$ = 7.6 Hz, $^4J_{H,H}$ = 1.2 Hz, 2H, *H-6*, *m*-C**H**), 2.41 (s, 6H, *H-2*, C**H**$_3$). ^{13}C-NMR (101 MHz, CD_2Cl_2): δ 168.66 (2C, *C-1'*, N=**C**-CH_3), 147.39 (2C, *C-11'*, *ipso*-**C**), 141.01 (2C, *C-3'*, *ipso*-**C**), 135.07 (2C, *C-12'*, *ipso*-**C**), 131.99 (2C, *C-9'*, *m*-**C**), 131.47 (2C, *C-7'*, *m*-**C**), 126.01 (2C, *C-13'*, *o*-**C**), 124.91 (2C, *C-4'*, *o*-**C**), 124.08 (2C, *C-14'*, *m*-**C**), 121.62 (2C, *C-5'*, *m*-**C**), 119.40 (2C, *C-6'*, *ipso*-**C**), 116.70 (2C, *C-8'*, *m*-**C**), 112.31 (2C, *C-10'*, *o*-**C**), 19.10 (2C, *C-2'*, **C**H_3). UV/Vis (nm (ε)): 233 (50666), 265 (38000), 306 (24666), 365 (13333). HRMS: *m/z* = 309.1492. *m/z* = 415.1927. **3b**$^+$ (*z* = 1) calc. 415.1923 (σ = 0.963409 ppm).

***Note*:**

1) Harsher dehydrative procedures were tried to speed up reaction time, but always led to either decomposition or condensation of *C-1'* onto *C-13'*.

[Ni(H_2PhenTAA)] (4a)

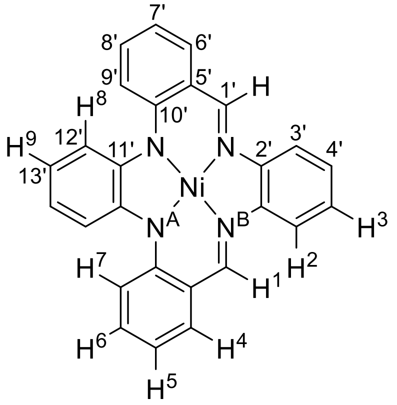

Compound **3a** (252 mg, 0.649 mmol, 1 eq.) and Ni(OAc)$_2$ • 4 H_2O (322.84 mg, 1.300 mmol, 2 eq.) were added to a Schlenk flask and cycled three times with Ar/vacuum. To this was added dry and degassed DMF (45 mL). The reaction was brought to reflux under Ar for 16 h, after which the reaction was cooled, and the solvent removed in vacuo. The residue was redissolved in CH_2Cl_2, filtered over cotton and washed three times with water. The organic layer was then washed with brine and subsequently dried with Na_2SO_4. The Na_2SO_4 was filtered off and the solution was concentrated in vacuo. The product was obtained after recrystallization from CH_2Cl_2/pentane. Yield: 255.5 mg (88%). Crystals suitable for X-ray diffraction were obtained by layering a concentrated CH_2Cl_2 solution with cyclopentane. ^{1}H-NMR (500 MHz, CD_2Cl_2): δ 8.01 (s, 2H, *H-1*, N=C**H**), 7.80 (d, $^3J_{H,H}$ = 8.9 Hz, 2H, *H-7*, *o*-C**H**), 7.58 (dd, $^3J_{H,H}$ = 6.0 Hz, $^3J_{H,H}$ = 3.3 Hz, 2H, *H-9*, *m*-C**H**), 7.40 (dd, $^3J_{H,H}$ = 6.1 Hz, $^3J_{H,H}$ = 3.3 Hz, 2H, *H-2*, *o*-C**H**), 7.32 (d, $^3J_{H,H}$ = 7.9 Hz, 2H, *H-4*, *o*-C**H**), 7.16 (dt, $^3J_{H,H}$ = 9.2 Hz, $^4J_{H,H}$ = 1.2 Hz, 2H, *H-6*, *m*-C**H**), 7.05 (dd, $^3J_{H,H}$ = 6.2 Hz, $^3J_{H,H}$ = 3.2 Hz, 2H, *H-3*, *m*-C**H**), 6.57 (dd, $^3J_{H,H}$ = 5.6 Hz, $^3J_{H,H}$ = 3.4 Hz, 2H, *H-8*, *o*-C**H**), 6.55 (t, $^3J_{H,H}$ = 6.7 Hz, 2H, *H-5*, *m*-C**H**). ^{13}C-NMR (126 MHz, CD_2Cl_2) δ 150.31 (2C, *C-1'*, N=**C**H), 149.83 (2C, *C-10'*, *ipso*-**C**), 147.82 (2C, *C-11'*, *ipso*-**C**), 145.17 (2C, *C-2'*, *ipso*-**C**), 135.39 (2C, *C-6'*, *o*-**C**), 133.19 (2C, *C-8'*, *m*-**C**), 127.80 (2C, *C-4'*, *m*-**C**), 123.75 (2C, *C-5'*, *ipso*-**C**), 120.07 (2C, *C-12'*, *o*-**C**), 119.38 (2C, *C-9'*, *o*-**C**), 117.33 (2C, *C-13'*, *m*-**C**), 116.39 (2C, *C-7'*, *m*-**C**), 115.26 (2C, *C-3'*, *o*-**C**). ^{15}N-NMR (51 MHz, CD_2Cl_2): 189 (2N, *N-B*, **N**=CH). HRMS: *m/z* = 444.0895. **4a**$^+$ (*z* = 1) calc. 444.0885 (σ = 2.251803 ppm).

Notes:

1) Older bottles of DMF can cause overreduction of the Ni(OAc)$_2$ • 4 H_2O to metallic Ni(0) and can impact the yield and purity of the complex. It is advisable to use a freshly produced batch or distill the DMF beforehand
2) The compound can also be recrystallized easily from CH_2Cl_2/MeOH.
3) Although the reaction is performed under inert conditions, the complex appears stable to air and water up to a temperature of at least 100 °C.

[Ni(Me_2PhenTAA)] (4b)

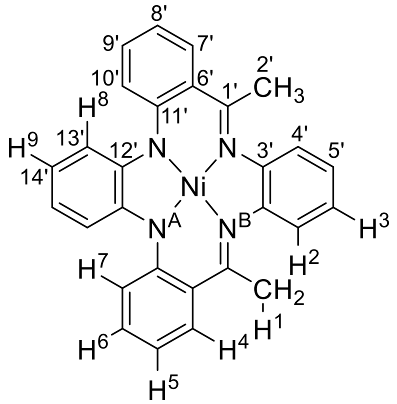

Compound **3b** (200 mg, 0.48 mmol, 1 eq.) and Ni(OAc)$_2$ • 4 H_2O (239 mg, 0.96 mmol, 2 eq.) were added to a Schlenk and cycled three times with Ar/vacuum. To this was added dry and degassed DMF (40 mL). The reaction was brought to reflux under Ar for 16 h, after which the reaction was cooled, and the solvent removed in vacuo. The residue was redissolved in CH_2Cl_2, filtered over cotton and washed three times with water. The organic layer was then washed with brine and subsequently dried with Na_2SO_4. The Na_2SO_4 was filtered off and the solution was concentrated in vacuo. The product was obtained after recrystallization from CH_2Cl_2/pentane. Yield: 204 mg (90%). ^{1}H-NMR (400 MHz, CD_2Cl_2): δ 7.57 – 7.55 (m, 2H, *H-7*, *o*-C**H**), 7.55 – 7.51 (m, 2H, *H-4*, *o*-C**H**), 7.27 (dt, $^3J_{H,H}$ = 9.4 Hz, $^3J_{H,H}$ = 3.4 Hz, 2H, *H-8*, *m*-C**H**), 6.97 (ddd, $^3J_{H,H}$ = 9.2 Hz, $^3J_{H,H}$ = 6.6 Hz, $^4J_{H,H}$ = 1.6 Hz, 2H,

H-6, *m*-C**H**), 6.92 – 6.86 (m, 2H, *H-3*, *m*-C**H**), 6.86 – 6.80 (m, 2H, *H-2*, *o*-C**H**), 6.50 (t, $^{3}J_{H,H}$ = 6.1 Hz, $^{3}J_{H,H}$ = 3.4 Hz, 2H, *H-9*, *m*-C**H**), 6.36 (t, $^{3}J_{H,H}$ = 7.5 Hz, 2H, *H-5*, *m*-C**H**), 2.52 (s, 6H, *H-1*, C**H**$_3$). ^{13}C-NMR (101 MHz, CD_2Cl_2): δ 162.22 (2C, *C-1'*, N=**C**CH$_3$), 148.76 (2C, *C-11'*, *ipso*-**C**), 148.20 (2C, *C-12'*, *ipso*-**C**), 147.75 (2C, *C-3'*, *ipso*-**C**), 132.05 (2C, *C-7'*, *o*-**C**), 132.02 (2C, *C-9'*, *m*-**C**), 126.07 (2C, *C-5'*, *m*-**C**), 125.83 (2C, *C-6'*, *ipso*-**C**), 123.75 (2C, *C-4'*, *o*-**C**), 119.63 (2C, *C-14'*, *m*-**C**), 118.36 (2C, *C-13'*, *o*-**C**), 118.02 (2C, *C-10'*, *o*-**C**), 114.75 (2C, *C-8'*, *m*-**C**), 20.16 (2C, *C-2'*, **C**H$_3$). ^{15}N-NMR (41 MHz, CD_2Cl_2): 187 (2N, *N-B*, **N**=CH). HRMS: *m/z* = 472.1201. **4b**$^+$ (z = 1) calc. 472.1198 (σ = 0.635432 ppm).

[Ni(Ph$_2$PhenTAA)] (4c)

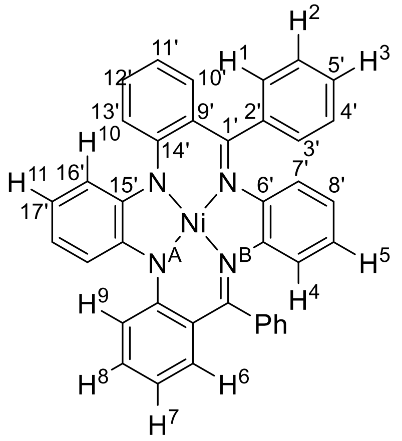

Compound **2c** (1000 mg, 2.13 mmol, 1 eq.), *o*-phenylene diamine (230.5 mg, 2.13 mmol, 1 eq.) and Ni(OAc)$_2$ • 4 H_2O (531 mg, 2.13 mmol, 1 eq.) were added to a flame-dried 50 mL Schlenk flask. Separately, a reflux condenser was connected to a Dean-Stark trap and closed off with a small round-bottom flask. Both setups were cycled three times with Ar/vacuum (vacuum ≥ 2 min.), after which was added 30 mL xylene to the reaction flask. The Dean-Stark trap was filled with 11.5 mL xylene to the top and the apparatus was connected to the reaction flask under Ar. The mixture was refluxed vigorously for 7 days, after which it was cooled down and the solvent removed in vacuo. The dark black-purple residue was redissolved in CH_2Cl_2/petroleum ether (40/60) (1:1 v/v) and passed over a short silica plug. The first fraction is the product (R_F = 0.8 (CH_2Cl_2)), the second fraction is compound **2c** (R_F = 0.3 (CH_2Cl_2)) and an unidentified greyish spot remains on the baseline. The first fraction was evaporated in vacuo and recrystallized from CH_2Cl_2/MeOH. Yield: 403.5 mg (32%). Crystals suitable for X-ray diffraction were obtained by via layering of a concentrated CH_2Cl_2 solution with MeOH. ^{1}H-NMR (400 MHz, CD_2Cl_2): δ 7.67 (d, $^{3}J_{H,H}$ = 8.7 Hz, 1H, *H-9*, *o*-C**H**), 7.56 – 7.47 (m, 4H, *H-2*, *m*-C**H**), 7.43 – 7.39 (m, 2H, *H-3*, *p*-C**H**), 7.38 (ddd, $^{3}J_{H,H}$ = 9.4 Hz, $^{3}J_{H,H}$ = 4.0 Hz, $^{3}J_{H,H}$ = 3,9 Hz, 2H, *H-10*, *o*-C**H**), 7.35 – 7.30 (m, 4H, *H-1*, *o*-C**H**), 6.98 (ddd, $^{3}J_{H,H}$ = 9.1 Hz, $^{3}J_{H,H}$ = 6.6 Hz, $^{4}J_{H,H}$ = 1.3 Hz, 2H, *H-8*, *m*-C**H**), 6.81 (dd, $^{3}J_{H,H}$ = 8.6 Hz, $^{4}J_{H,H}$ = 1.3 Hz, 2H, *H-6*, *o*-C**H**), 6.62 (s, 2H, *H-11*, *m*-C**H**), 6.22 (t, $^{3}J_{H,H}$ = 7.4 Hz, 2H, *H-7*, *m*-C**H**), 6.17 (ddd, $^{3}J_{H,H}$ = 9.7 Hz, $^{3}J_{H,H}$ = 4.2 Hz, $^{3}J_{H,H}$ = 3.8 Hz, 2H, *H-5*, *m*-C**H**), 5.70 (ddd, $^{3}J_{H,H}$ = 9.6 Hz, $^{3}J_{H,H}$ = 4.3 Hz, $^{3}J_{H,H}$ = 3.7 Hz, 2H, *H-4*, *o*-C**H**). ^{13}C-NMR (101 MHz, CD_2Cl_2): 162.45 (2C, *C-1'*, N=**C**Ph), 147.80 (2C, *C-14'*, *ipso*-**C**), 137.57 (2C, *C-6'*, *ipso*-**C**), 135.60 (2C, *C-10'*, *o*-**C**), 132.56 (2C, *C-12'*, *m*-**C**), 130.78 (2C, *C-2'*, *ipso*-**C**), 130.09 (4C, *C-3'*, *o*-**C**), 129.99 (4C, *C-4'*, *m*-**C**), 129.66 (2C, *C-5'*, *p*-**C**), 129.15 (2C, *C-15'*, *ipso*-**C**), 128.08 (2C, *C-9'*, *ipso*-**C**), 125.22 (2C, *C-8'*, *m*-**C**), 123.44 (2C, *C-7'*, *o*-**C**), 120.04 (2C, *C-17'*, *m*-**C**), 118.82 (2C, *C-16'*, *o*-**C**), 117.52 (2C, *C-13'*, *o*-**C**), 114.58 (2C, *C-11'*, *m*-**C**). ^{15}N-NMR (51 MHz, C_6D_6): 185 (2N, *N-B*, **N**=CPh), 90 (2N, *N-A*, Ar$_2$**N**). HRMS: *m/z* = 596.1515. **4a**$^+$ (z = 1) calc. 596.1511 (σ = 0.670971 ppm).

Notes:

1) While the complex can be purified via column chromatography, it decomposes slowly on silica and should be passed over the silica as soon as possible.
2) Due to π–π stacking, signal broadening can be significant when measuring ^{1}H-NMR in CD_2Cl_2. This is not a problem when C_6D_6 is used.

X-ray Diffraction

N,N'-(1,2-phenylenediamino)-bis(2-aminobenzaldehyde) (2a):

(A) **(B)**

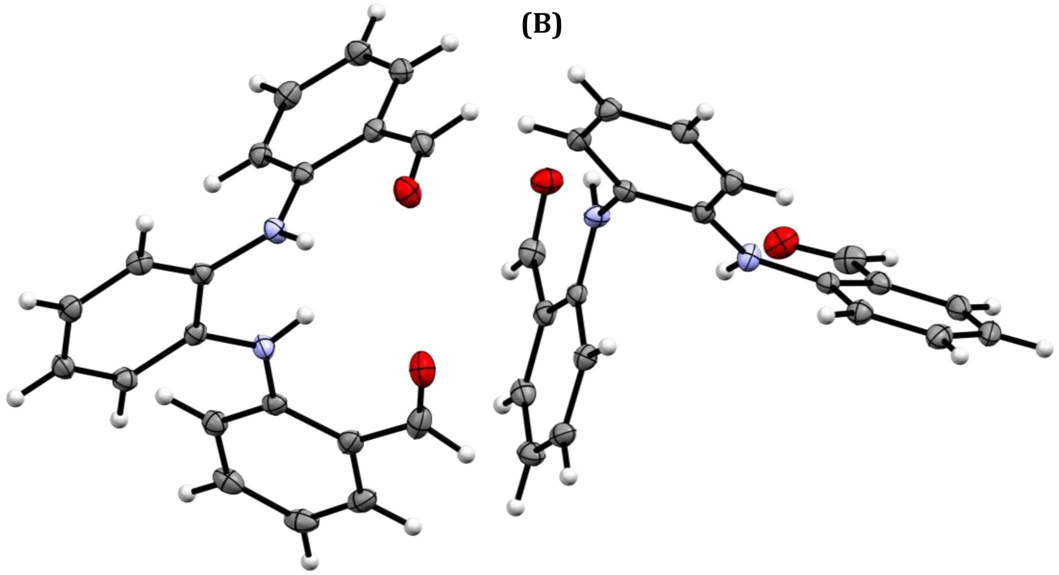

Figure S1. (A) 50% thermal ellipsoid probability plot (top view) of the SC–XRD measurement of **2a** **(B)** Side view.

Table S1. Crystal structure and determination data of **2a**, **2c**, **3a**, **4a** & **4c**.

Compound	**2a**	**2c**	**3a**	**4a**	**4c**
CCDC number	2277309	2277308	2277307	2300376	2277310
Empirical formula	$C_{20}H_{16}N_2O_2$	$C_{32}H_{24}N_2O_2$	$C_{26}H_{20}N_4$	$C_{80}H_{58}Cl_4N_{12}Ni_3$	$C_{38.25}H_{26.50}Cl_{0.50}N_4Ni$
Formula weight	316.35	468.53	388.46	1505.31	618.57
Temperature [K]	100(2)	150(2)	150(2)	110(2)	100(2)
Crystal system	monoclinic	monoclinic	orthorhombic	monoclinic	monoclinic
Space group (number)	$P2_1/n$ (14)	$P2_1/c$ (14)	$Pnma$ (62)	$P2_1/c$ (14)	$P2_1/c$ (14)
a [Å]	12.8286(8)	10.6873(5)	7.9492(6)	18.4838(3)	9.4354(7)
b [Å]	7.7173(5)	28.2800(12)	18.7658(13)	26.4251(4)	23.4870(17)
c [Å]	15.8011(11)	8.2652(4)	12.7592(10)	13.2538(2)	13.1194(10)
α [°]	90	90	90	90	90
β [°]	101.144(3)	100.2670(10)	90	97.9616(15)	104.843(3)
γ [°]	90	90	90	90	90
Volume [Å^3]	1534.85(18)	2458.0(2)	1903.3(2)	6411.24(17)	2810.4(4)
Z	4	4	4	4	4
ρ_{calc} [gcm^{-3}]	1.369	1.266	1.356	1.560	1.462
μ [mm^{-1}]	0.090	0.079	0.082	1.099	0.775
F(000)	664	984	816	3096	1282
Crystal size [mm^3]	0.226×0.186×0.096	0.642×0.363×0.204	0.520×0.237×0.232	0.28×0.10×0.06	0.394×0.102×0.050
Crystal colour	yellow	yellow	orange	dark-red	violet
Crystal shape	plate	block	block	block	block
Radiation	MoK_α (λ=0.71073 Å)	MoK_α (λ=0.71073 Å)	MoK_α (λ=0.71073 Å)	MoK_α (λ=0.71073 Å)	MoK_α (λ=0.71073 Å)
2θ range [°]	4.55 to 54.97	5.21 to 57.38	6.39 to 53.49	4.34 to 55.00	4.73 to 55.05
	(0.77 Å)	(0.74 Å)	(0.79 Å)	(0.77 Å)	(0.77 Å)
Index ranges	−16 ≤ h ≤ 16	−14 ≤ h ≤ 14	−8 ≤ h ≤ 10	−24 ≤ h ≤ 24	−12 ≤ h ≤ 12
	−10 ≤ k ≤ 10	−38 ≤ k ≤ 38	−23 ≤ k ≤ 20	−34 ≤ k ≤ 34	−30 ≤ k ≤ 30
	−20 ≤ l ≤ 20	−11 ≤ l ≤ 11	−14 ≤ l ≤ 16	−17 ≤ l ≤ 14	−17 ≤ l ≤ 17
Reflections collected	213560	64649	8163	97507	325152
Independent reflections	3489	6337	2063	14715	6459
	R_{int} = 0.0702	R_{int} = 0.0289	R_{int} = 0.0405	R_{int} = 0.0313	R_{int} = 0.0877
	R_{sigma} = 0.0175	R_{sigma} = 0.0168	R_{sigma} = 0.0456	R_{sigma} = 0.0206	R_{sigma} = 0.0166
Completeness to θ = 25.242°	99.9 %	99.8 %	99.1 %	99.9 %	99.9 %
Data/Restraints/Parameters	3489/0/223	6337/0/331	2063/0/139	14715/75/920	6459/0/388
Goodness-of-fit on F^2	1.148	1.067	1.148	1.035	1.149
Final R indexes [I≥2σ(I)]	R_1 = 0.0467	R_1 = 0.0481	R_1 = 0.0829	R_1 = 0.0276	R_1 = 0.0480
	wR_2 = 0.0977	wR_2 = 0.1153	wR_2 = 0.1723	wR_2 = 0.0669	wR_2 = 0.1282
Final R indexes [all data]	R_1 = 0.0535	R_1 = 0.0577	R_1 = 0.1009	R_1 = 0.0338	R_1 = 0.0564
	wR_2 = 0.1025	wR_2 = 0.1214	wR_2 = 0.1810	wR_2 = 0.0703	wR_2 = 0.1340
Largest peak/hole [eÅ^{-3}]	0.28/-0.23	0.29/-0.23	0.41/-0.24	0.36/-0.32	0.74/-0.69

N,N'-(1,2-phenylenediamino)-bis(2-aminobenzophenone) (2c):

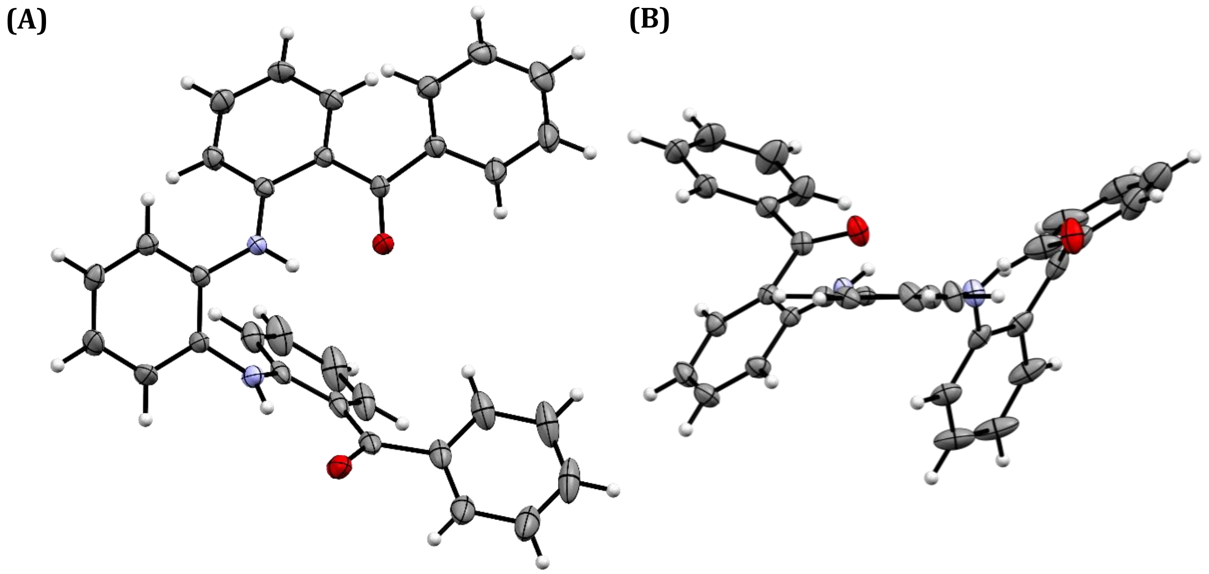

Figure S2. (A) 50% thermal ellipsoid probability plot (top view) of the SC–XRD measurement of **2c (B)** Side view.

$H_2(H_2PhenTAA)$ (3a):

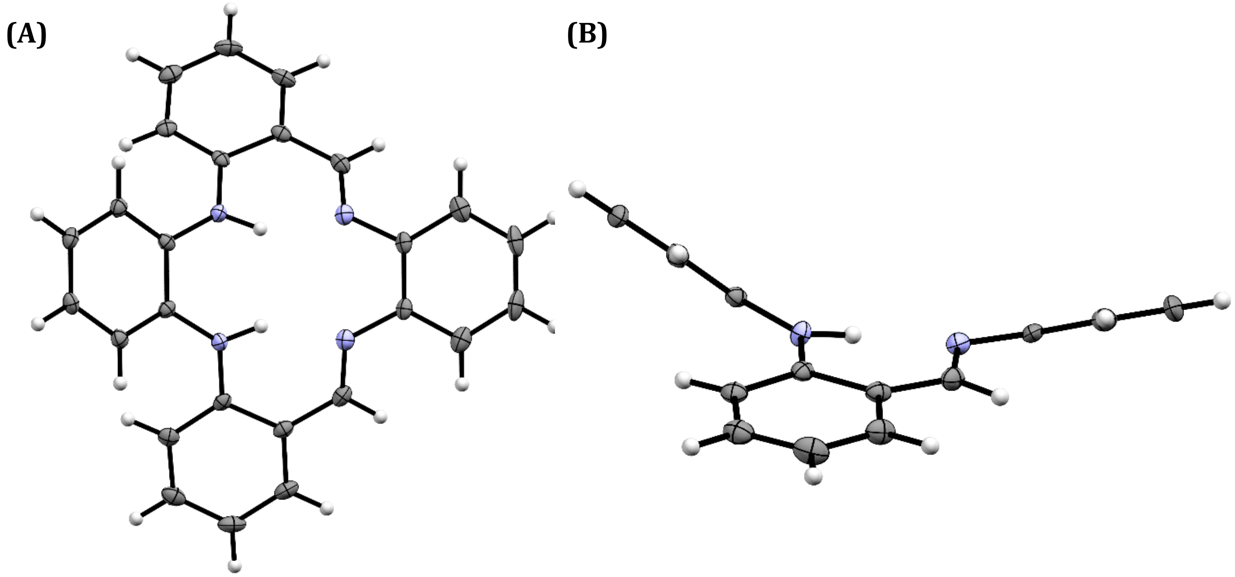

Figure S3. (A) 50% thermal ellipsoid probability plot (top view) of the SC–XRD measurement of **3a (B)** Side view.

[Ni(H_2PhenTAA)] (4a):

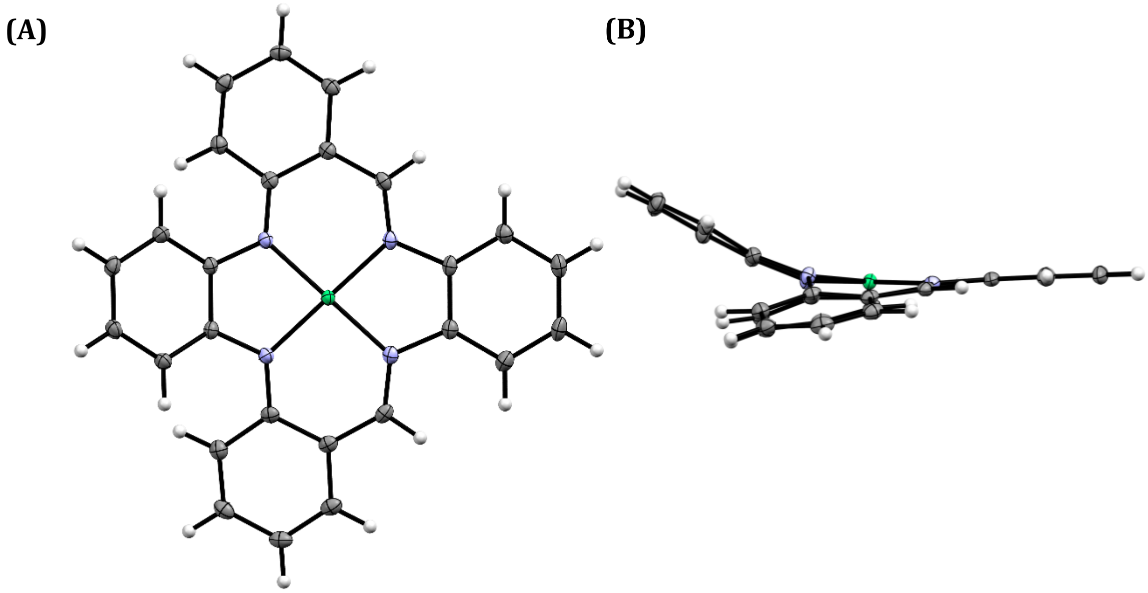

Figure S4. (A) 50% thermal ellipsoid probability plot (top view) of the SC–XRD measurement of **4a (B)** Side view.

[Ni(Ph_2PhenTAA)] (4c):

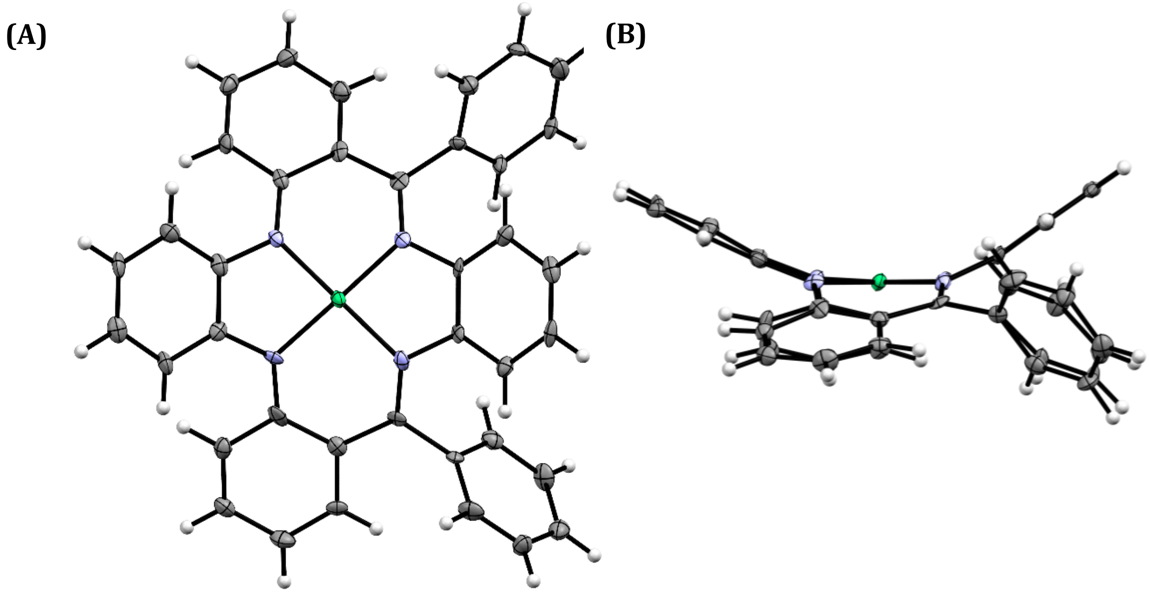

Figure S5. (A) 50% thermal ellipsoid probability plot (top view) of the SC–XRD measurement of **4c (B)** Side view.

Bond-length analysis

Cyclization of **2a-c** to free base **3a-b** and nickel complexes **4a-c** results in a fully conjugated macrocycle. To investigate any delocalization or aromatization effects, a bond-length analysis was performed as well as use of the harmonic oscillator model for aromaticity (HOMA) (Table S2). The numbering used here is different from the single-crystal XRD structures.

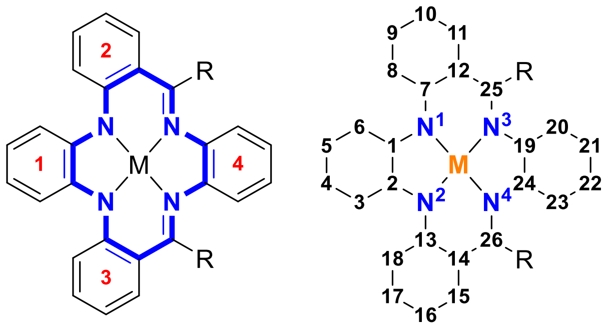

Scheme S1. Numbering scheme for the bond-length analysis in Table S2. The numbering here is different from the aforementioned single-crystal XRD structures.

Table S2. Selected bond-lengths of molecules **2a**, **2c**, **3a**, **4a** & **4c**. Data ordered per "macrocyclic" benzene ring and exocyclic bond types (with respect to benzene rings). **X** = O (**2a**, **2c**) or N (**3a**, **4a** & **4c**).

Bond	2a	2c	3a	4a	4c
Ring 1					
C1–C2	1.410(2)	1.402(2)	1.427(4)	1.411(2)	1.416(4)
C2–C3	1.395(2)	1.390(2)	1.383(4)	1.404(2)	1.394(3)
C3–C4	1.385(2)	1.384(2)	1.390(4)	1.386(2)	1.385(4)
C4–C5	1.384(2)	1.383(2)	1.382(5)	1.392(2)	1.396(4)
C5–C6	1.383(2)	1.385(2)	1.390(4)	1.389(2)	1.391(3)
C6–C1	1.388(2)	1.396(2)	1.383(4)	1.399(2)	1.391(4)
Ring 2					
C7–C8	1.400 (2)	1.406(2)	1.394(4)	1.426(2)	1.433(4)
C8–C9	1.377(2)	1.382(2)	1.374(4)	1.372(2)	1.367(4)
C9–C10	1.392(2)	1.393(2)	1.384(5)	1.407(2)	1.403(3)
C10–C11	1.372(2)	1.379(2)	1.388(5)	1.366(2)	1.379(4)
C11–C12	1.397(2)	1.403(2)	1.402(4)	1.418(2)	1.414(4)
C12–C7	1.418(2)	1.425(2)	1.435(4)	1.428(2)	1.427(3)
Ring 3					
C13–C14	1.414(2)	1.423(2)	1.394(4)	1.427(2)	1.433(4)
C14–C15	1.397(2)	1.405(2)	1.374(4)	1.419(2)	1.424(4)
C15–C16	1.374(2)	1.372(2)	1.384(5)	1.362(2)	1.371(3)
C16–C17	1.385(2)	1.388(2)	1.388(5)	1.407(2)	1.398(4)
C17–C18	1.382(2)	1.379(2)	1.402(4)	1.366(2)	1.381(4)
C18–C13	1.399(2)	1.423(2)	1.435(4)	1.426(2)	1.425(3)
Ring 4					
C19–C20	-	-	1.403(5)	1.396(2)	1.396(4)
C20–C21	-	-	1.374(5)	1.387(2)	1.389(4)
C21–C22	-	-	1.366(5)	1.385(2)	1.383(4)
C22–C23	-	-	1.374(5)	1.386(2)	1.388(4)
C23–C24	-	-	1.403(5)	1.393(2)	1.403(4)
C24–C19	-	-	1.402(4)	1.395(2)	1.410(4)
C-C/X bonds					
C1–N1	1.420(2)	1.399(1)	1.409(4)	1.406(2)	1.402(3)
N1–C7	1.373(2)	1.377(2)	1.369(4)	1.361(2)	1.362(3)
C2–N2	1.395(2)	1.416(2)	1.409(4)	1.402(2)	1.405(4)
N2–C13	1.380(2)	1.373(2)	1.369(4)	1.361(2)	1.356(4)
C12–C25	1.453(2)	1.477(2)	1.434(4)	1.417(2)	1.457(4)
C14–C26	1.457(2)	1.476(2)	1.434(4)	1.416(2)	1.446(3)
C25–X3	1.219(2)	1.230(2)	1.284(4)	1.304(2)	1.322(3)
C26–X4	1.219(2)	1.231(2)	1.284(4)	1.304(2)	1.329(3)
N3–C19	-	-	1.412(4)	1.424(2)	1.413(3)
N4–C24	-	-	1.412(4)	1.424(2)	1.427(3)
N-Ni bonds					
N1–Ni	-	-	-	1.864(1)	1.842(2)
N2–Ni	-	-	-	1.865(1)	1.841(2)
N3–Ni	-	-	-	1.860(1)	1.874(2)
N4–Ni	-	-	-	1.863(1)	1.867(2)

Table S3. HOMA values for the four benzene rings (red; **1**–**4**) and the macrocycle (blue; 5) based on the bond-length analysis in Table S2. DFT-calculated values (BP86/def2-TZVP/disp3) are displayed between parentheses.

Ring	2a	2c	3a	4a	4c
**1**	0.97 (0.93)	0.99 (0.93)	0.93 (0.91)	0.96 (0.92)	0.96 (0.92)
**2**	0.93 (0.90)	0.91 (0.90)	0.88 (0.81)	0.78 (0.71)	0.78 (0.68)
**3**	0.94 (0.84)	0.87 (0.80)	0.88 (0.81)	0.76 (0.71)	0.78 (0.68)
**4**	-	-	0.93 (0.90)	0.99 (0.95)	0.96 (0.93)
**Macrocycle**	-	-	0.40 (0.41)	0.56 (0.58)	0.54 (0.50)

UV/Vis spectra:

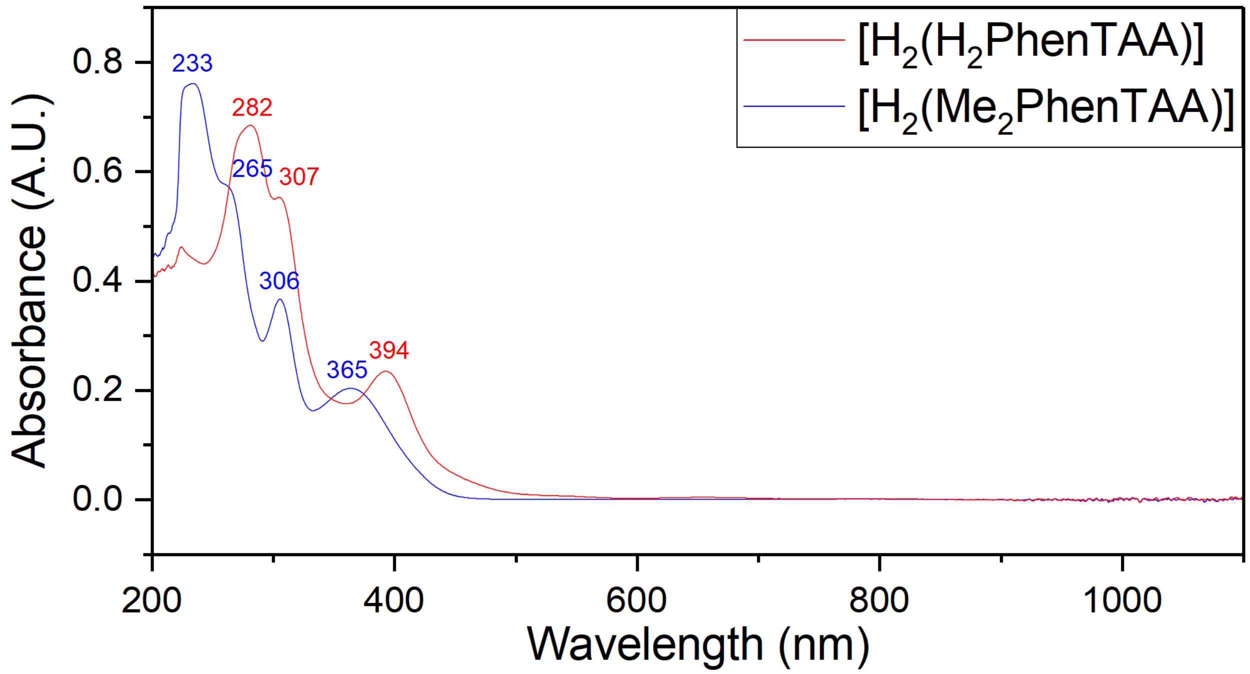

Figure S6. UV/Vis spectra of compounds [$H_2(H_2$PhenTAA)] (**3a)** (red) and [$H_2(Me_2$PhenTAA)] (**3b)** (blue) in CH_2Cl_2 at 15 μM.

In-situ ATR–FT–IR spectra:

(A) (B)

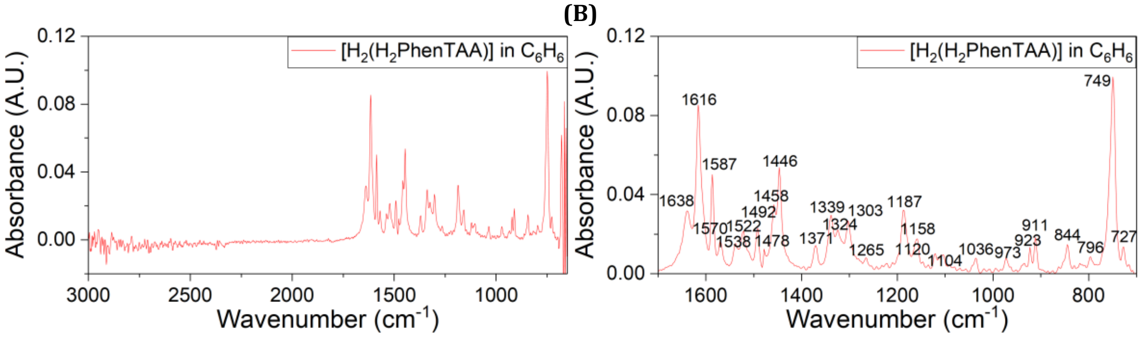

Figure S7. in-situ ATR–FT–IR spectrum of [H_2(H_2PhenTAA)] in C_6H_6 (A). Zoomed-in spectrum with labelled peaks (B).

(A) (B)

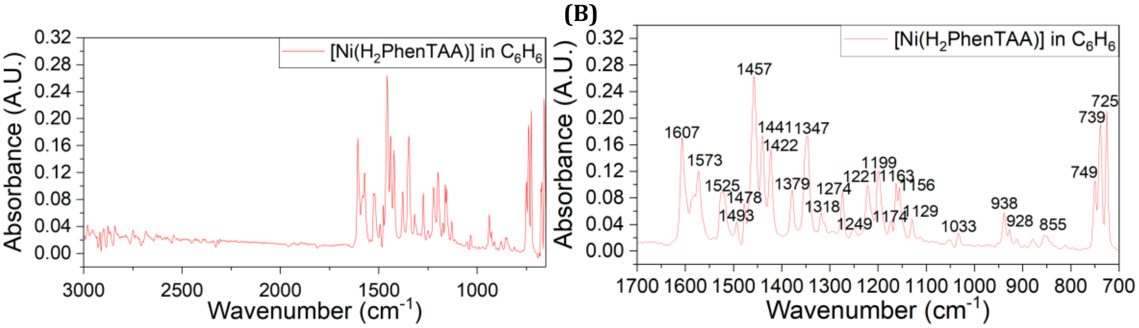

Figure S8. in-situ ATR–FT–IR spectrum of [Ni(H_2PhenTAA)] in C_6H_6 (A). Zoomed-in spectrum with labelled peaks (B).

(A) (B)

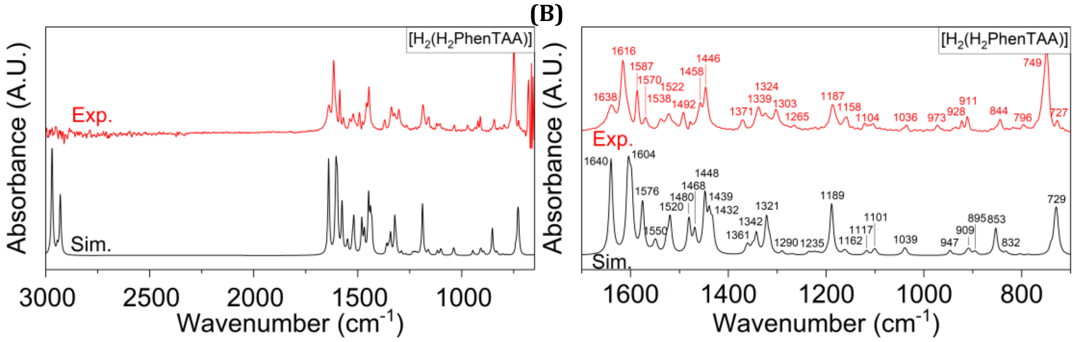

Figure S9. in-situ ATR–FT–IR spectra of [H_2(H_2PhenTAA)] (red) with the DFT-simulated spectrum below (black) (A). Zoomed-in spectrum (B).

(A) (B)

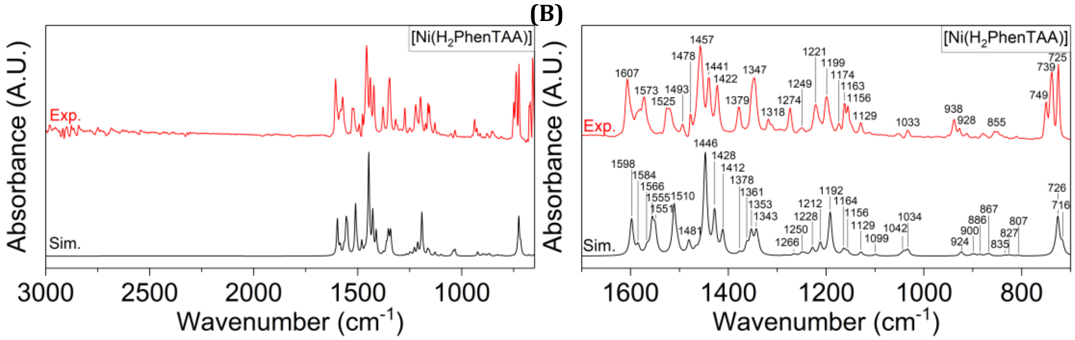

Figure S10. in-situ ATR–FT–IR spectra of [Ni(H_2PhenTAA)] (red) with the DFT-simulated spectrum below (black) (A). Zoomed-in spectrum (B)

Table S4. Experimental vs. calculated IR spectroscopy data of [H_2(H_2PhenTAA)] (**3a**) and [Ni(H_2PhenTAA)] (**4a**) in cm^{-1}.

**H_2(H_2PhenTAA) (3a) (Exp.)**	**H_2(H_2PhenTAA) (calc.)**	**Ni(H_2PhenTAA) (4a) (Exp.)**	**Ni(H_2PhenTAA) (calc.)**
1638	1640	1607	1598
1616	1604	1573	1584
1587	1576	1525	1566
1570	1550	1493	1555
1538	1520	1478	1551
1522	1480	1457	1510
1492	1468	1441	1481
1478	1448	1422	1446
1458	1439	1379	1428
1446	1432	1347	1412
1371	1361	1318	1378
1339	1342	1274	1361
1324	1321	1249	1353
1303	1290	1221	1343
1265	1235	1199	1266
1187	1189	1174	1250
1158	1162	1163	1228
1120	1117	1156	1212
1104	1101	1129	1192
1036	1039	1033	1164
973	947	938	1156
923	909	928	1129
911	895	855	1099
844	853	749	1042
796	832	739	1034
749	729	725	924
727			900
			886
			867
			835
			827
			807
			726
			716

Electrochemistry

Cyclic voltammetry

General procedure: To a flame dried Schlenk flask was added [Ni(R_2PhenTAA)] (10 μmol), which was cycled three times with Ar/vacuum (vacuum ≥ 2 min.), after which 10 mL of 100 mM $TBAPF_6$ in the desired solvent (dry and degassed) was added. An oven-dried electrochemical cell was supplied with a glassy carbon working electrode, Pt counter electrode and a leak-free Ag/AgCl (3.5 M KCl) reference electrode. To this setup was added 4 mL of 1 mM NiR_2PhenTAA solution, closed with two J-Young valve taps and reconnected a N_2-line and the potentiostat for measurement. Electrochemical windows were based on blank measurements of the same electrolyte solution. Voltages were determined based on differential pulse voltammograms measured with and without added ferrocene.

Table S5. List of all redox events for [Ni]-complexes **4a–c** based on differential pulse voltammogram. Voltages reported vs. Fc/Fc^+.

Peak type	[Ni(H_2PhenTAA)]	[Ni(Me_2PhenTAA)]	[Ni(Ph_2PhenTAA)]
	CH_2Cl_2		
2nd reduction	-	–	–
1st reduction	−1.88 V	−1.86 V	−1.84 V
1st oxidation	−0.10 V	−0.15 V	−0.11 V
2nd oxidation	+0.41 V	+0.34 V	+0.40 V
3rd oxidation	–	+1.49 V[a]	+1.63 V
	THF		
2nd reduction	–	−2.16 V[a]	−2.25 V
1st reduction	−1.91 V	−1.73 V	−1.87 V
1st oxidation	−0.07 V	+0.08 V	+0.00 V
2nd oxidation	+0.31 V	+0.41 V	+0.34 V
3rd oxidation	–	–	–

[a] = Based on CV due to overlap of solvent oxidation/reduction.

[Ni(H_2PhenTAA)]

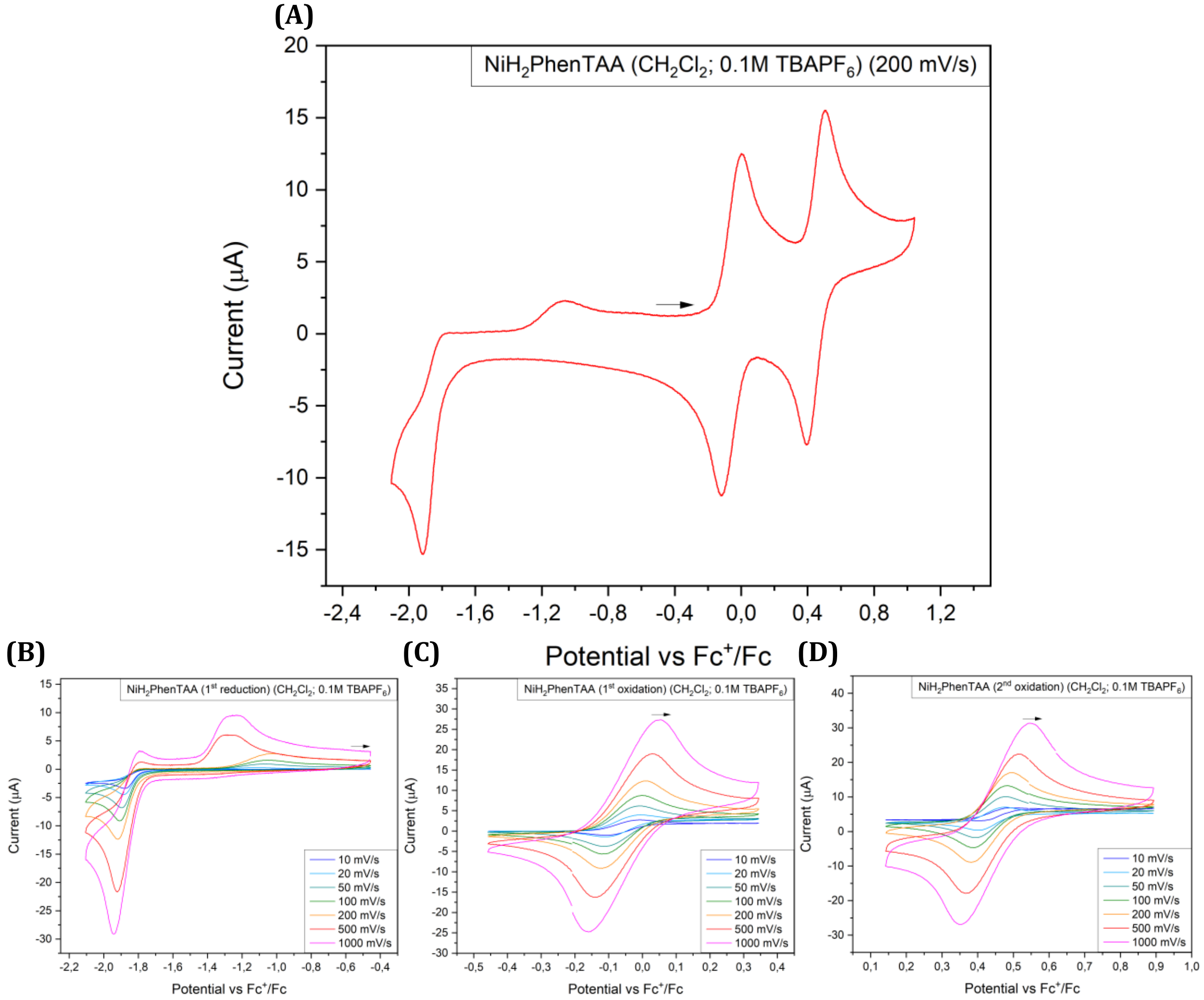

Figure S11. A. Cyclic voltammogram of all three redox events of 4a in CH_2Cl_2 at 200 mV/s (A). Scan rate dependency of the 1st reduction at −1.88 V (B), the 1st oxidation at −0.10 V (C) and the 2nd oxidation at +0.41 V (D). All voltages are reported vs. Fc/Fc^+.

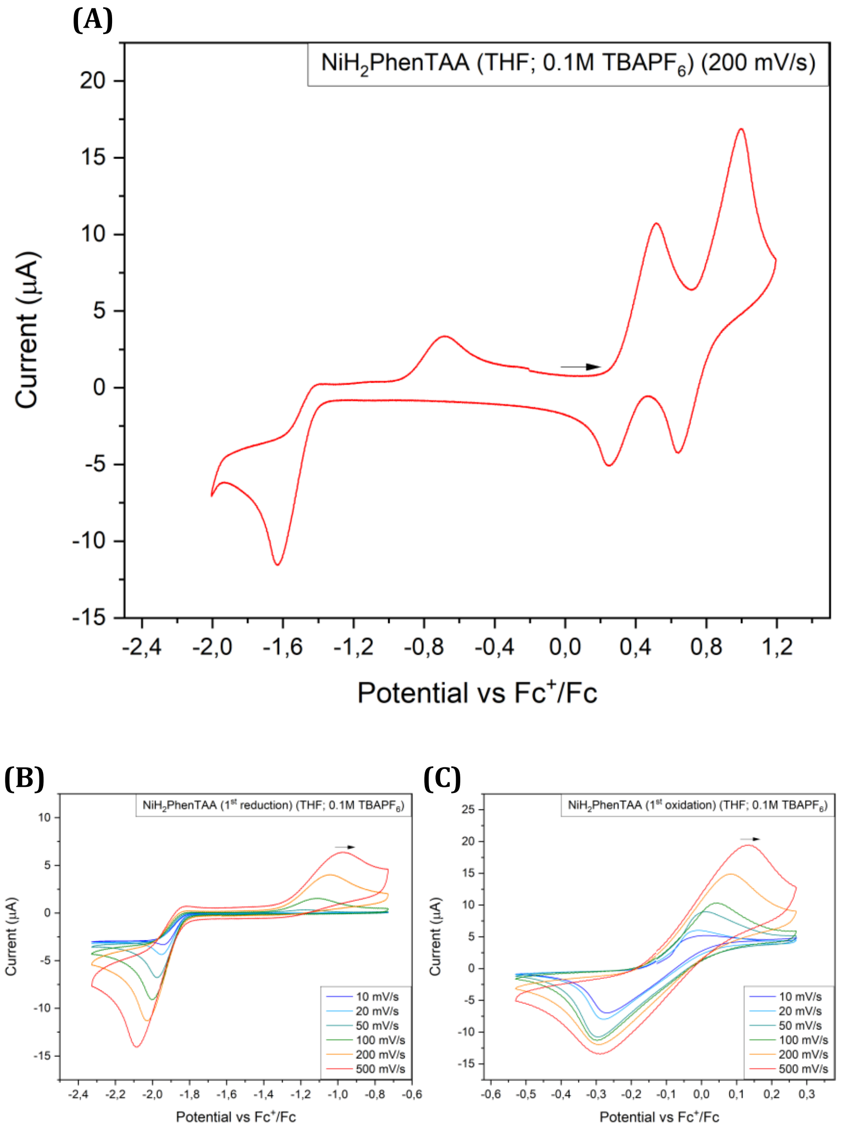

Figure S12. Cyclic voltammogram of all three redox events of **4a** in THF at 200 mV/s (**A**). Scan rate dependency of the 1st reduction at –1.91 V (**B**) and the 1st oxidation at –0.07 V (**C**). All voltages are reported vs. Fc/Fc^+.

[Ni(Me$_2$PhenTAA)]

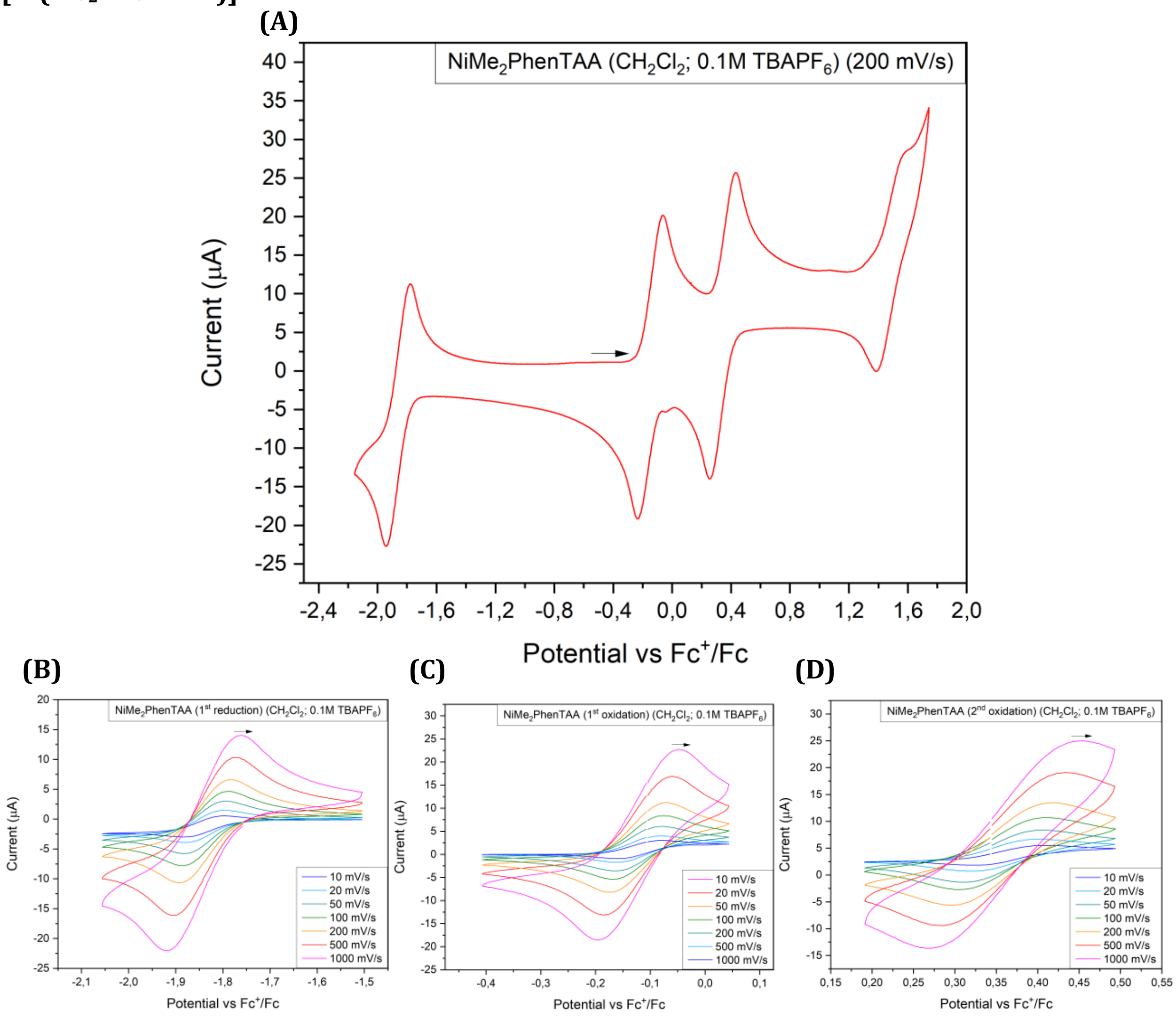

Figure S13. Cyclic voltammogram of all four redox events of **4b** in CH_2Cl_2 at 200 mV/s (**A**). Scan rate dependency of the 1st reduction at −1.86 V (**B**), the 1st oxidation at −0.15 V (**C**) and the 2nd oxidation at +0.34 V (**D**). All voltages are reported vs. Fc/Fc$^+$.

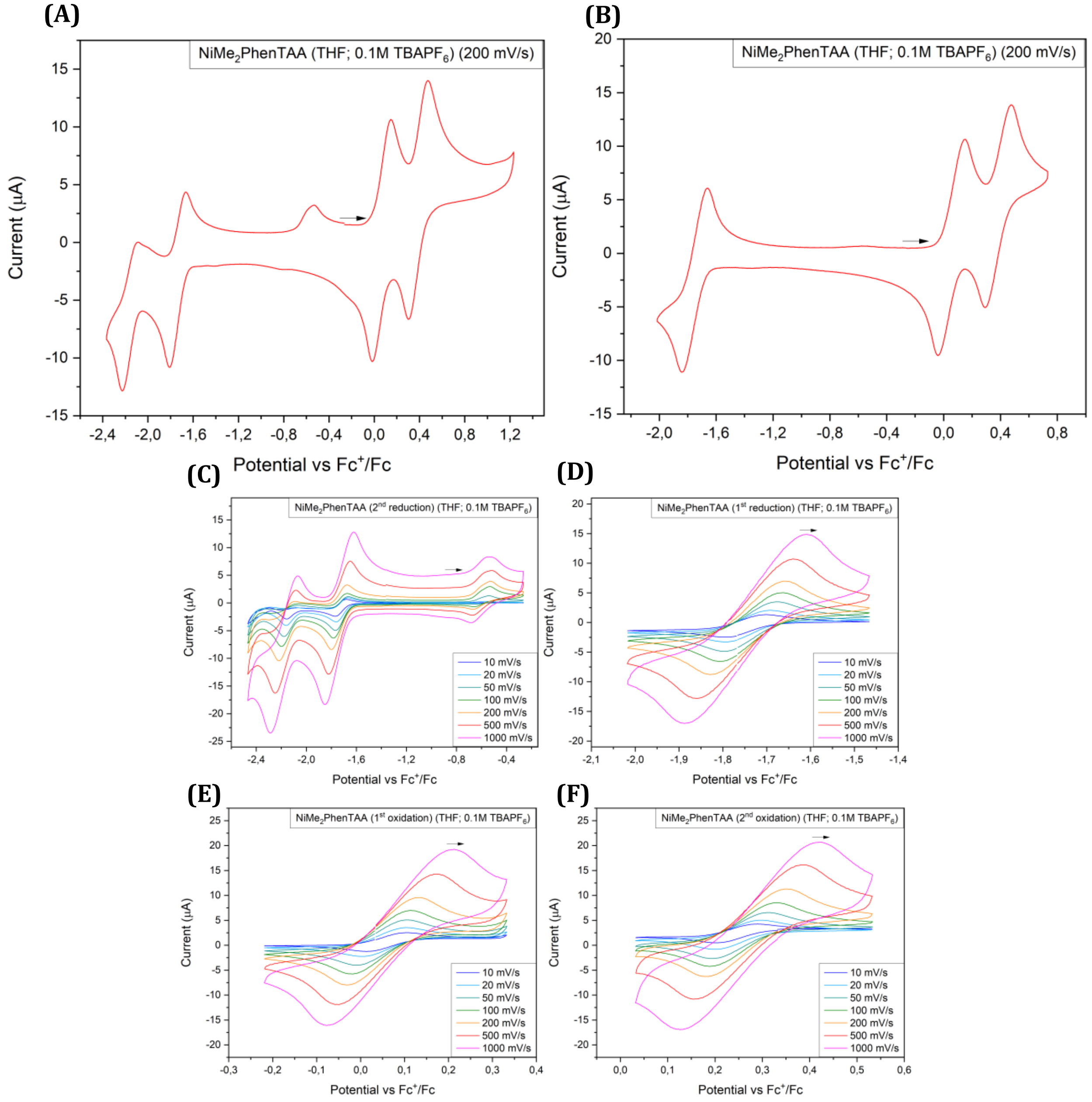

Figure S14. Cyclic voltammogram of the three reversible redox events (**A**) and all four redox events (**B**) of **4b** in THF at 200 mV/s. Scan rate dependency of the 2nd and 1st reduction at −2.16 V and −1.73 V resp. (**C**), the isolated 1st reduction at −1.73 V (**D**), the 1st oxidation at +0.08 V (**E**) and the 2nd oxidation at +0.41 V (**F**). All voltages are reported vs. Fc/Fc^{+}.

[$NiPh_2PhenTAA$]

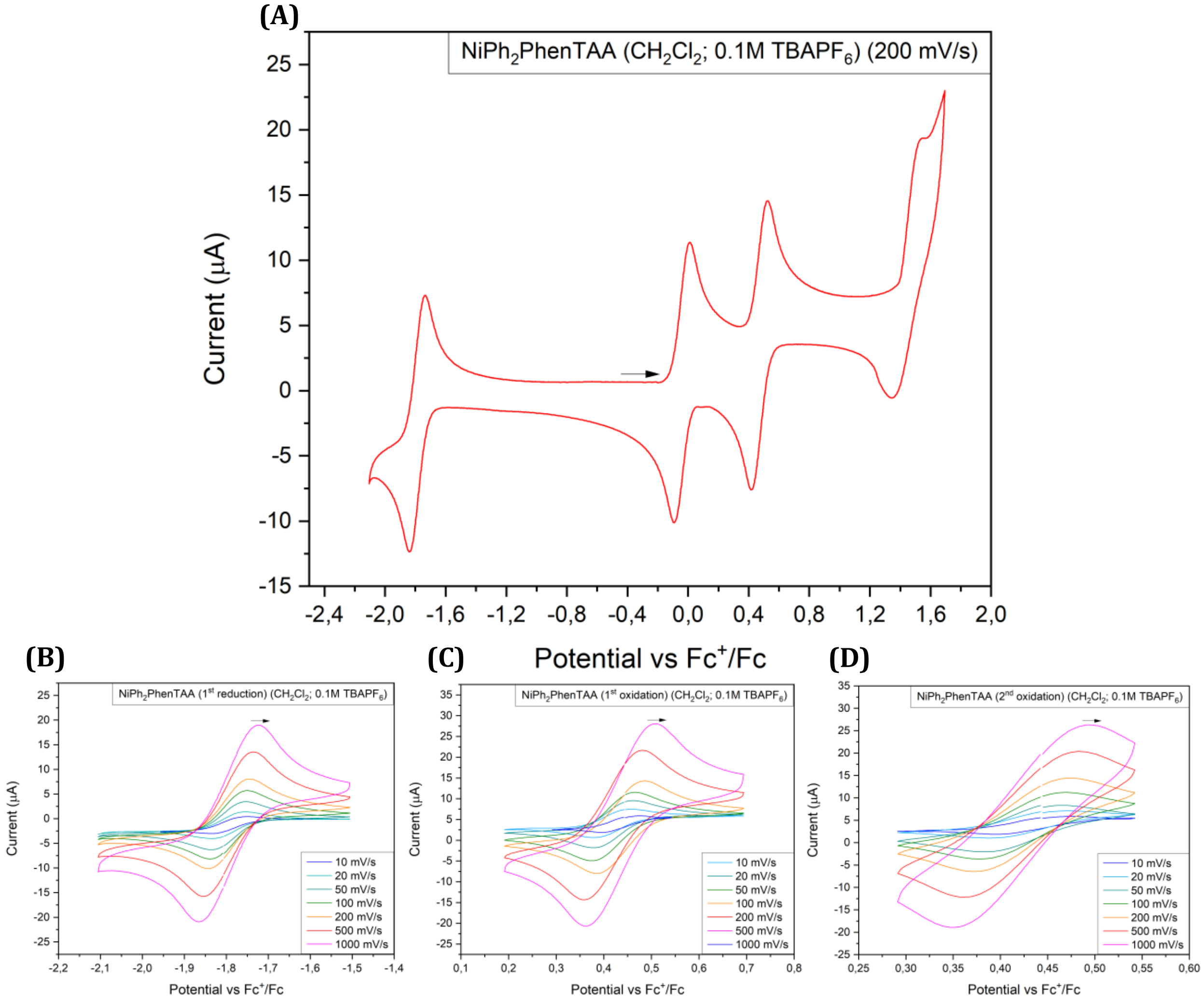

Figure S15. Cyclic voltammogram of all four redox events of **4c** in CH_2Cl_2 at 200 mV/s (**A**). Scan rate dependency of the 1st reduction at −1.84 V (**B**), the 1st oxidation at −0.11 V (**C**) and the 2nd oxidation at +0.40 V (**D**). All voltages are reported vs. Fc/Fc^+.

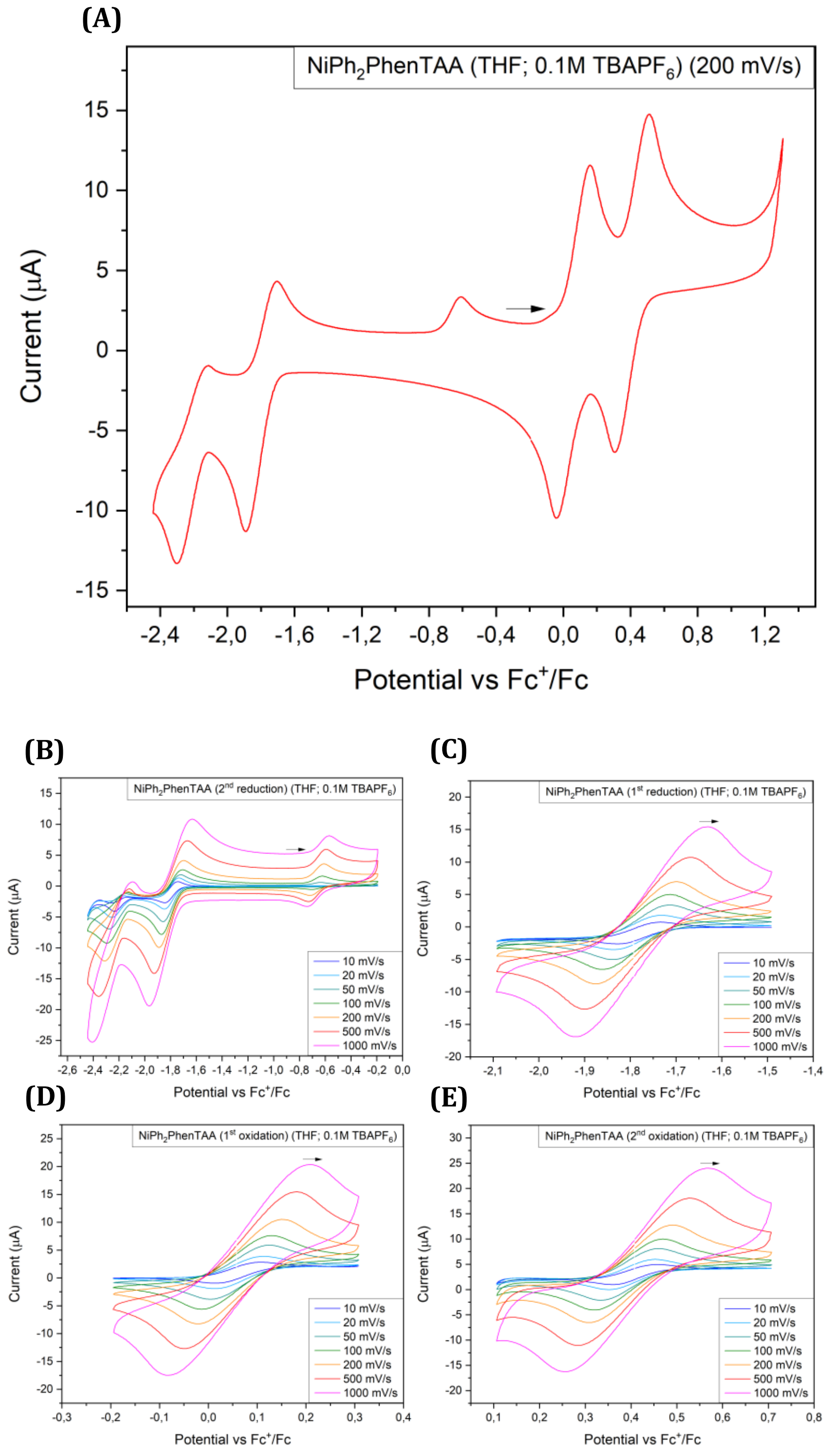

Figure S16. Cyclic voltammogram of all four redox events of **4b** in THF at 200 mV/s (**A**). Scan rate dependency of the 2nd and 1st reduction at –2.25 V and –1.87 V resp. (**B**), the isolated 1st reduction at –1.87 V (**C**), the 1st oxidation at +0.00 V (**D**) and the 2nd oxidation at +0.34 V (**E**). All voltages are reported vs. Fc/Fc$^+$.

Electrochemistry

Spectroelectrochemistry

General procedure: To a flame dried Schlenk flask was added [Ni(R_2PhenTAA)] (5.0 μmol), which was cycled three times with Ar/vacuum (vacuum ≥ 2 min.), after which 5 mL of 100 mM $TBAPF_6$ in the desired solvent (dry and degassed) was added to acquire a 1 mM solution of [Ni] complex. A standard OTTLE cell (l = 0.2 mm) with a Pt working and auxiliary electrode and a Ag wire reference electrode was purged with Ar via a syringe in an Ar bucket. To the OTTLE cell was added a small amount of [Ni] solution. The cell was inspected for gas bubbles, subsequently sealed and transported to the UV/Vis spectrometer. The background spectrum as well as the electrochemical windows were based on blank measurements of the same electrolyte solution.

Table S6. UV/Vis spectral data for all detected species during spectroelectrochemical monitoring of **4a-c** in both CH_2Cl_2 and THF.

**[Ni(H_2PhenTAA)] (4a)**											
2eOX (CH_2Cl_2)		1eOX (CH_2Cl_2)		0eNeutral (CH_2Cl_2)		0eNeutral (THF)		1eRED (THF)		2eRED (THF)	
nm	ε	nm	ε	nm	ε	nm	ε	nm	ε	nm	ε
217	23400	216	26900	216	30000	245	16300	252	13550	-	-
244	20650	236	23200	238	26500	294	18750	305	14600	-	-
301	23600	301	26400	301	31300	309	19750	364	9150	-	-
358	15250	358	15950	497	8050	333	13600	519	3550	-	-
439	4500	440	5000	627	4150	495	6650	579	3650	-	-
502	4500	502	6850			620	3350	733	1250	-	-
**[Ni(Me_2PhenTAA)] (4b)**											
2eOX (CH_2Cl_2)		1eOX (CH_2Cl_2)		0eNeutral (CH_2Cl_2)		0eNeutral (THF)		1eRED (THF)		2eRED (THF)	
nm	ε	nm	ε	nm	ε	nm	ε	nm	ε	nm	ε
220	23050	221	24150	218	25800	237	38300	263	34750	265	33500
241	23550	241	26300	239	28450	262	39900	306	35200	305	33750
300	22650	301	25000	298	30200	297	40150	364	22100	363	22500
485	3500	359	11100	317	26500	317	36600	497	10250	524	8650
		398	6600	485	5600	334	28950	657	3550	671	3150
		485	3900	667	3000	363	14700				
		633	2000			482	9650				
						664	4050				
**[Ni(Ph_2PhenTAA)] (4c)**											
2eOX (CH_2Cl_2)		1eOX (CH_2Cl_2)		0eNeutral (CH_2Cl_2)		0eNeutral (THF)		1eRED (THF)		2eRED (THF)	
nm	ε	nm	ε	nm	ε	nm	ε	nm	ε	nm	ε
218	34650	218	37000	218	37750	246	39600	246	37700	249	33750
243	29900	244	34600	243	36150	299	36150	298	33500	298	30850
279	27800	293	30450	292	32350	323	31700	321	31400	321	28250
476	5150	322	27750	323	29250	364	12850	365	14650	366	15750
537	4500	359	13700	369	9900	470	9300	470	9250	464	7700
		476	5700	471	6200	535	7350	525	8450	527	7150
		535	4950	533	5250	665	4600	664	4500	665	4150
		660	2400	660	2950						

[Ni(H_2PhenTAA)]:

Oxidation (CH_2Cl_2)

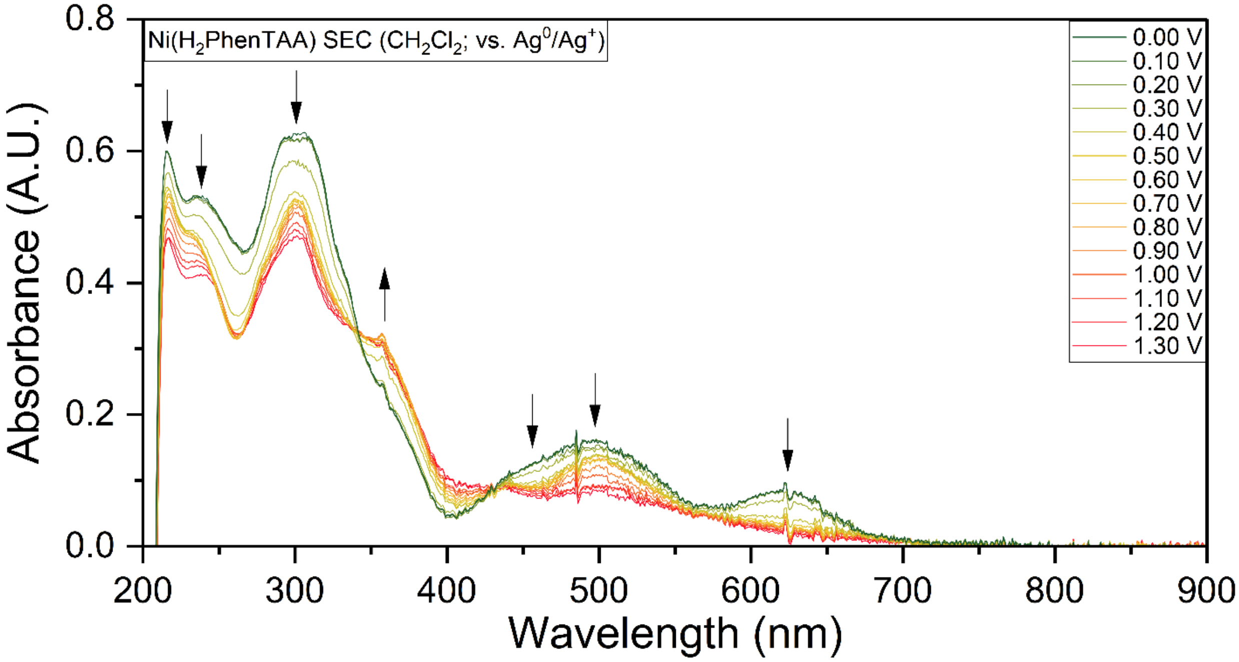

Figure S17. UV/Vis spectro-electrochemical monitoring of **4a** (1.0 mM) in CH_2Cl_2 over a voltage range of 0.00 V to +1.30 V. UV/Vis Measurements were conducted at intervals of 20 seconds at a scan rate of 0.5 mV/s.

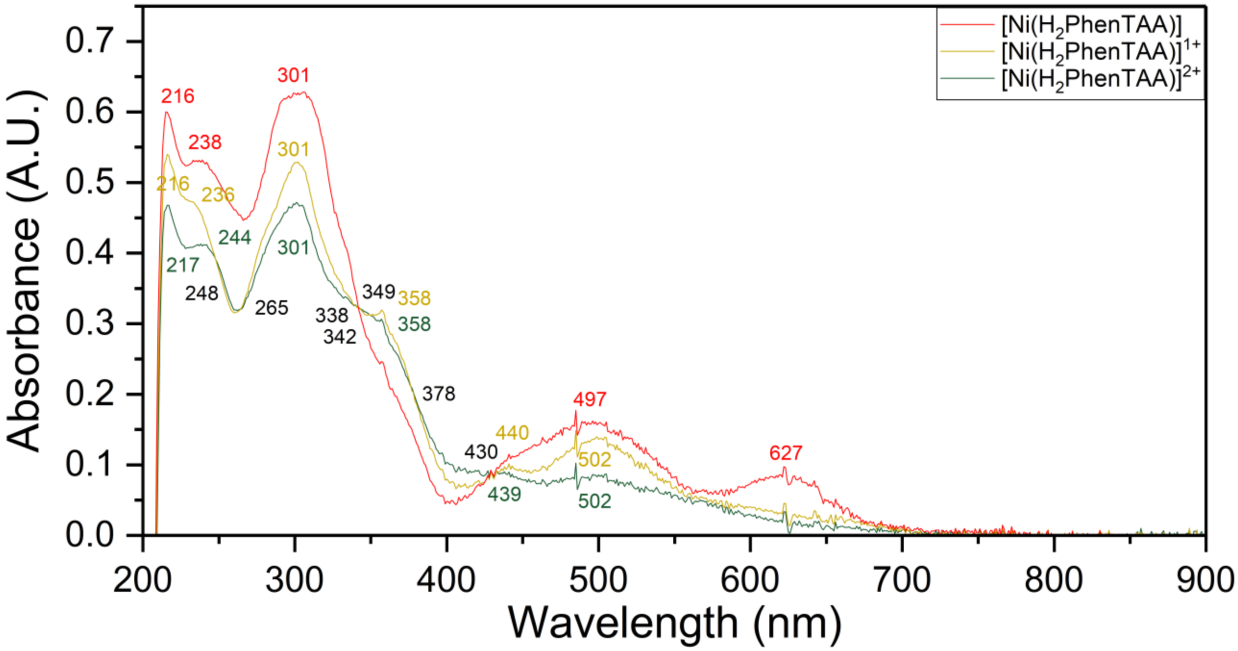

Figure S18. Isolated UV/Vis spectra of the neutral species **4a** (red), 1e oxidized species **4a⁺** (yellow) and 2e oxidized species **4a²⁺** (green). Isosbestic points (black) are included based on intersections between subsequent species (**4a** → **4a⁺** → **4a²⁺**).

Reduction (THF)

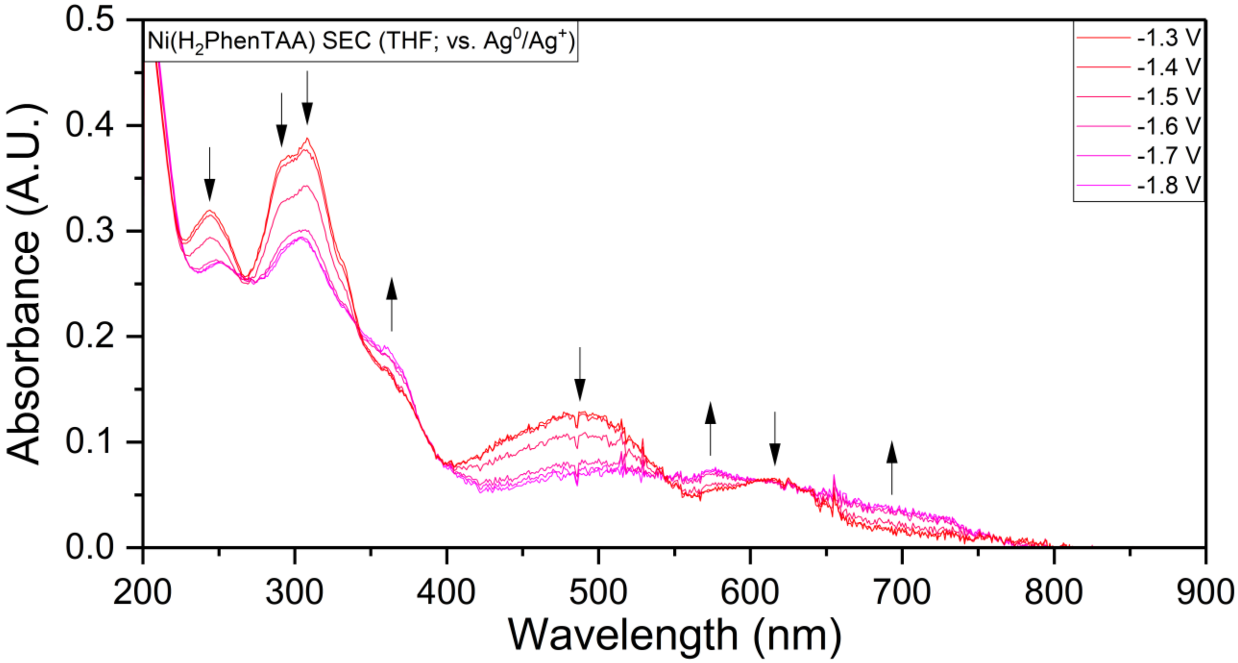

Figure S19. UV/Vis spectro-electrochemical monitoring of **4a** (0.5 mM) in THF over a voltage range of –1.30 V to –1.80 V. UV/Vis Measurements were conducted at intervals of 20 seconds at a scan rate of 0.5 mV/s.

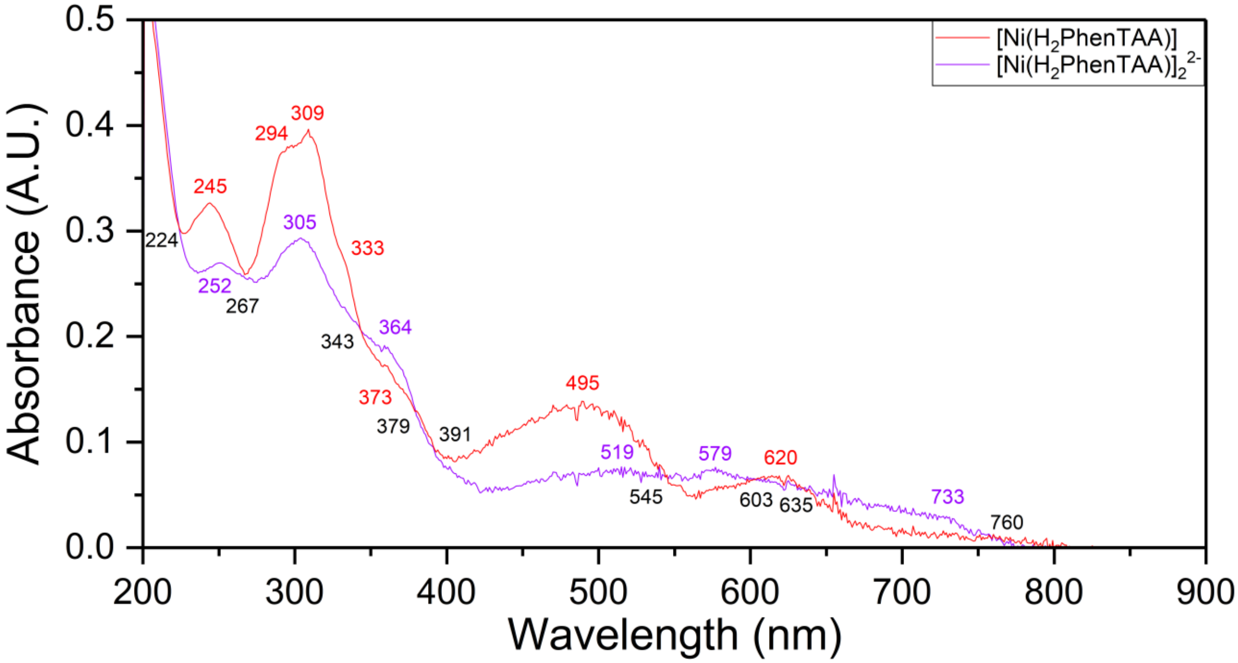

Figure S20. Isolated UV/Vis spectra of the neutral **4a** (red) and 1e reduced species **4a**⁻ (magenta), with isosbestic points included (black).

[Ni(Me$_2$PhenTAA)]:

Oxidation (CH_2Cl_2)

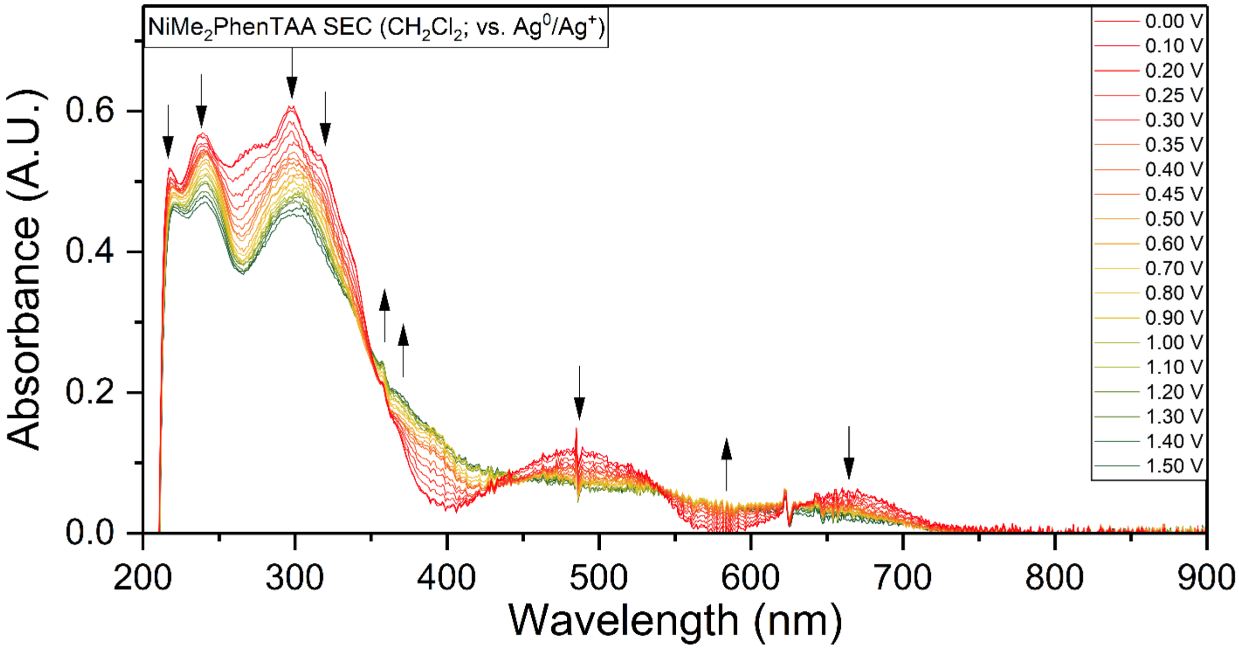

Figure S21. UV/Vis spectroelectrochemical monitoring of **4b** (1.0 mM) in CH_2Cl_2 over a voltage range of 0.00 V to +1.50 V. UV/Vis Measurements were conducted at intervals of 20 seconds at a scan rate of 0.5 mV s^{-1}.

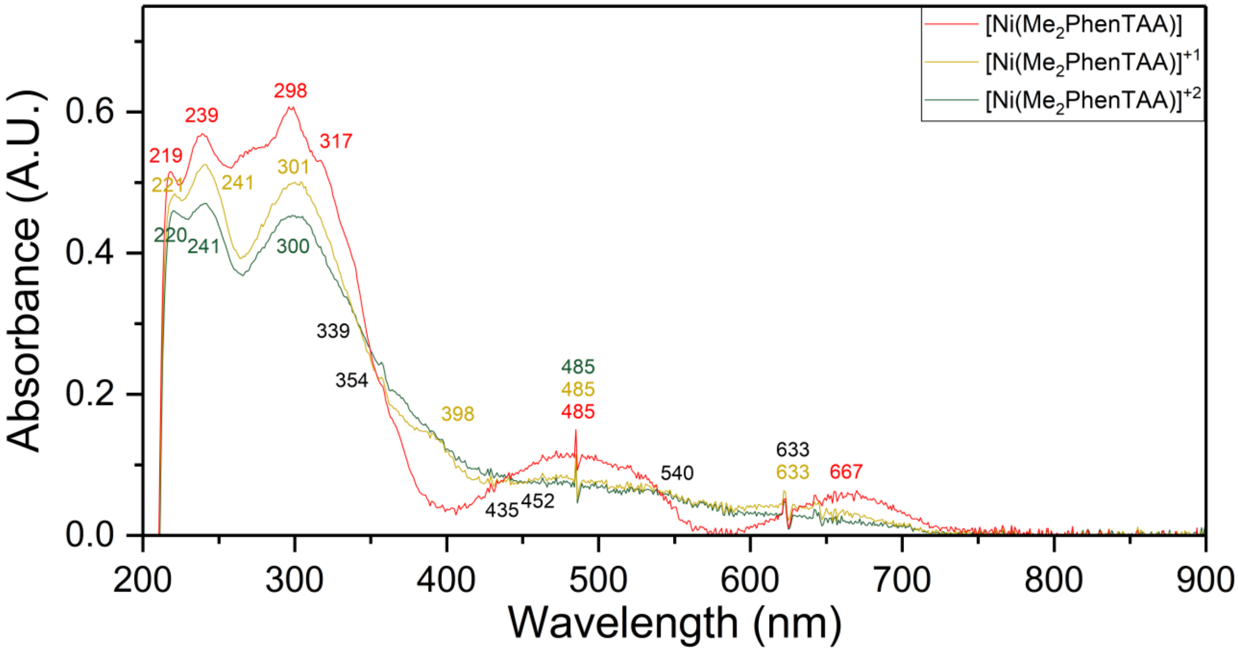

Figure S22. Isolated UV/Vis spectra of the neutral species **4b** (red), 1e oxidized species **4b$^+$** (yellow) and 2e oxidized species **4b^{2+}** (green). Isosbestic points (black) are included based on intersections between subsequent species (**4b** $\rightarrow$ **4b$^+$** $\rightarrow$ **4b^{2+}**).

Reduction (THF)

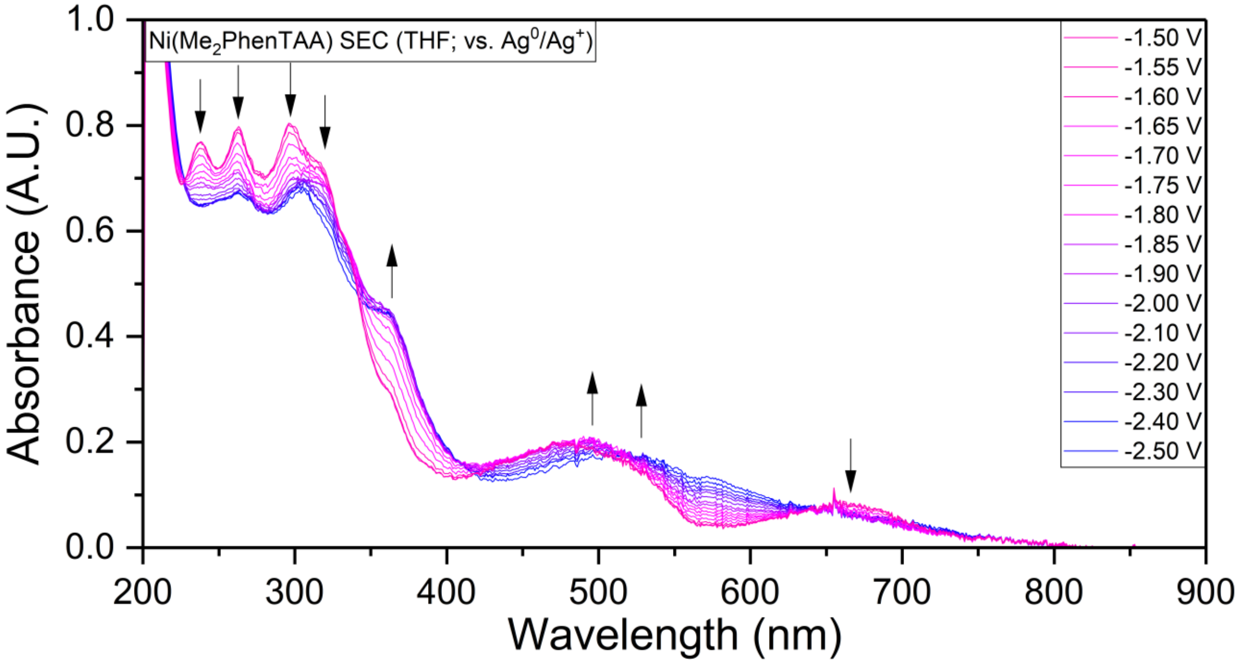

Figure S23. UV/Vis spectroelectrochemical monitoring of **4b** (1.0 mM) in THF over a voltage range of −1.50 V to −2.50 V. UV/Vis Measurements were conducted at intervals of 10 or 20 seconds at a scan rate of 0.5 mV/s.

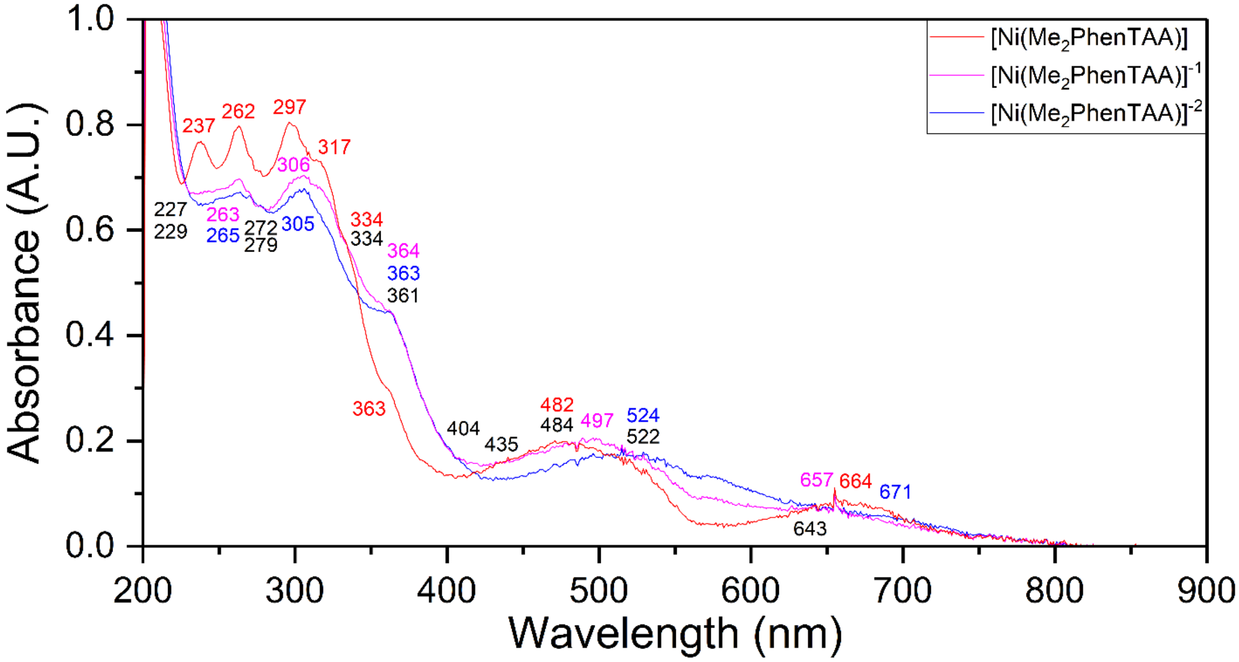

Figure S24. Isolated UV/Vis spectra of the neutral species **4b** (red), 1e reduced species **4b**⁻ (magenta) and 2e reduced species **4b**²⁻ (blue). Isosbestic points (black) are included based on intersections between subsequent species (**4b** → **4b**⁻ → **4b**²⁻).

[Ni(Ph_2PhenTAA)]:

Oxidation (CH_2Cl_2)

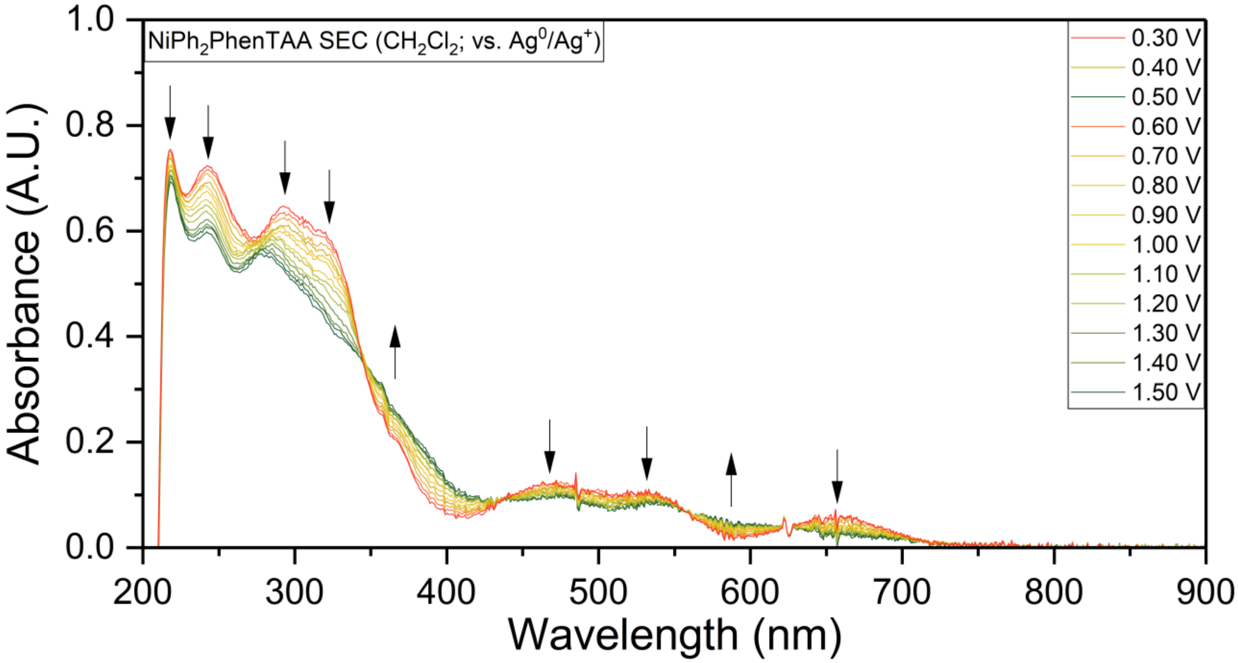

Figure S25. UV/Vis spectro-electrochemical monitoring of **4c** (1.0 mM) in CH_2Cl_2 over a voltage range of 0.30 V to +1.50 V. UV/Vis Measurements were conducted at intervals of 20 seconds at a scan rate of 0.5 mV/s.

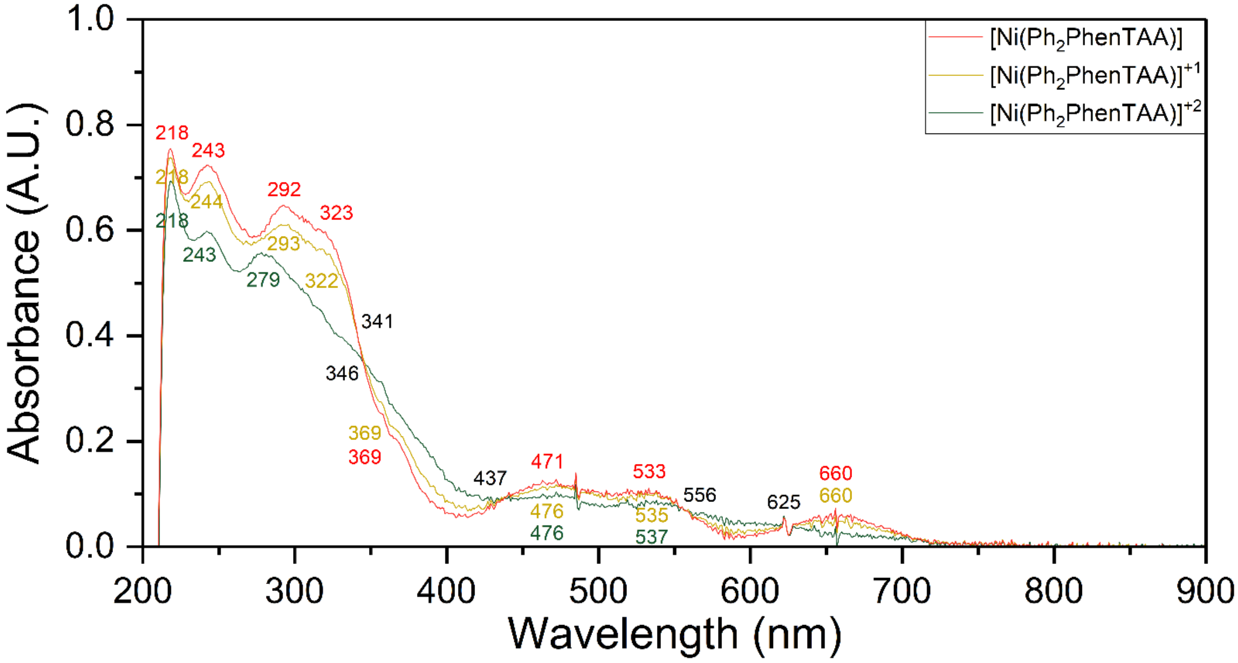

Figure S26. Isolated UV/Vis spectra of the neutral species **4c** (red), 1e oxidized species **4c**$^+$ (yellow) and 2e oxidized species **4c**$^{2+}$ (green). Isosbestic points (black) are included based on intersections between subsequent species (**4c** $\rightarrow$ **4c**$^+$ $\rightarrow$ **4c**$^{2+}$).

Reduction (THF)

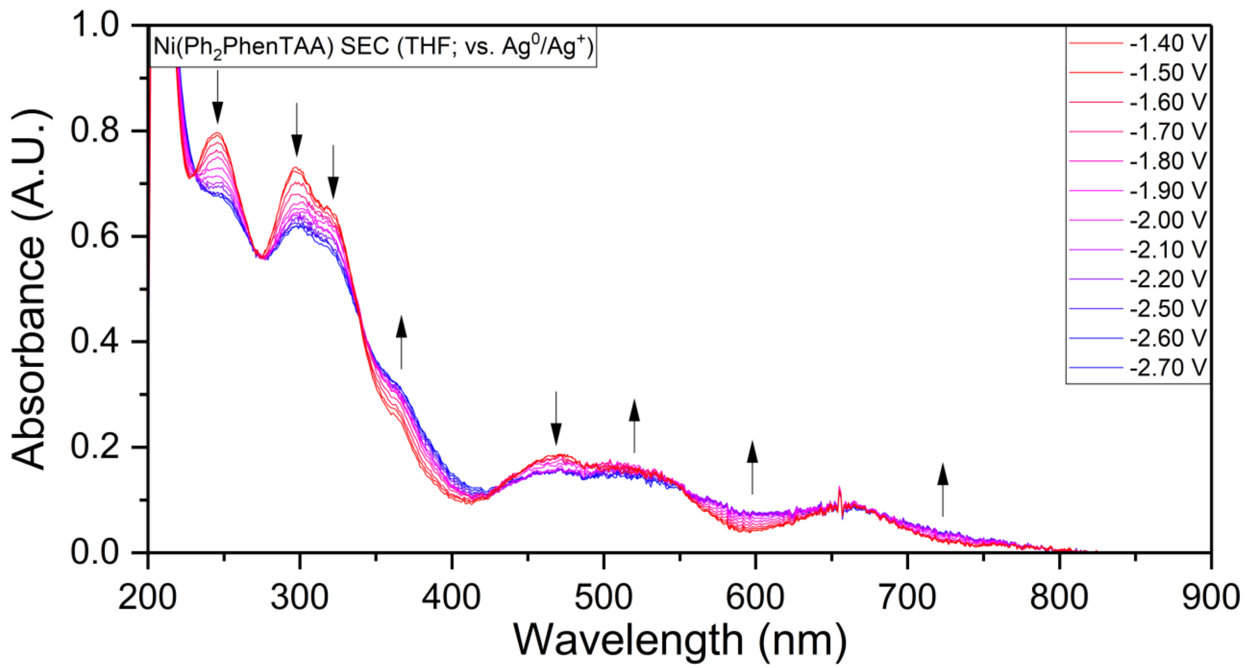

Figure S27. UV/Vis spectroelectrochemical monitoring of **4c** (1.0 mM) in THF over a voltage range of −1.40 V to −2.70 V. UV/Vis Measurements were conducted at intervals of 10 or 20 seconds at a scan rate of 0.5 mV/s.

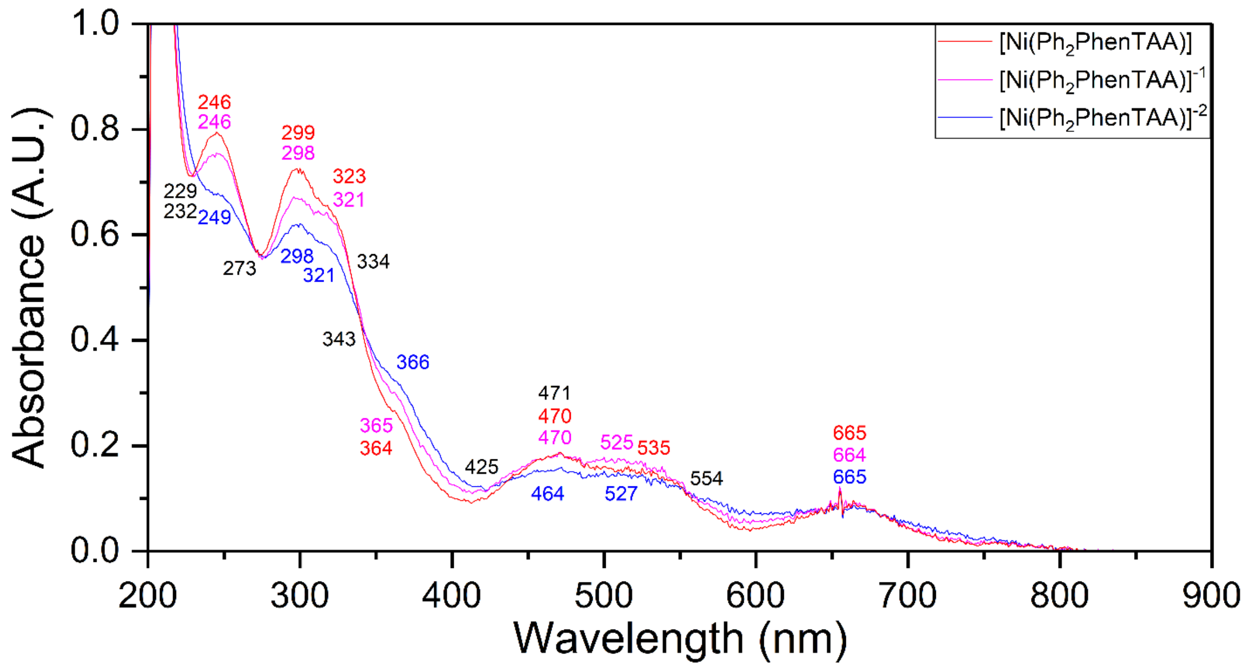

Figure S28. Isolated UV/Vis spectra of the neutral species **4c** (red), 1e reduced species **4c**⁻ (magenta) and 2e reduced species $\mathbf{4c}^{2-}$ (blue). Isosbestic points (black) are included based on intersections between subsequent species (**4c** → **4c**⁻ → $\mathbf{4c}^{2-}$).

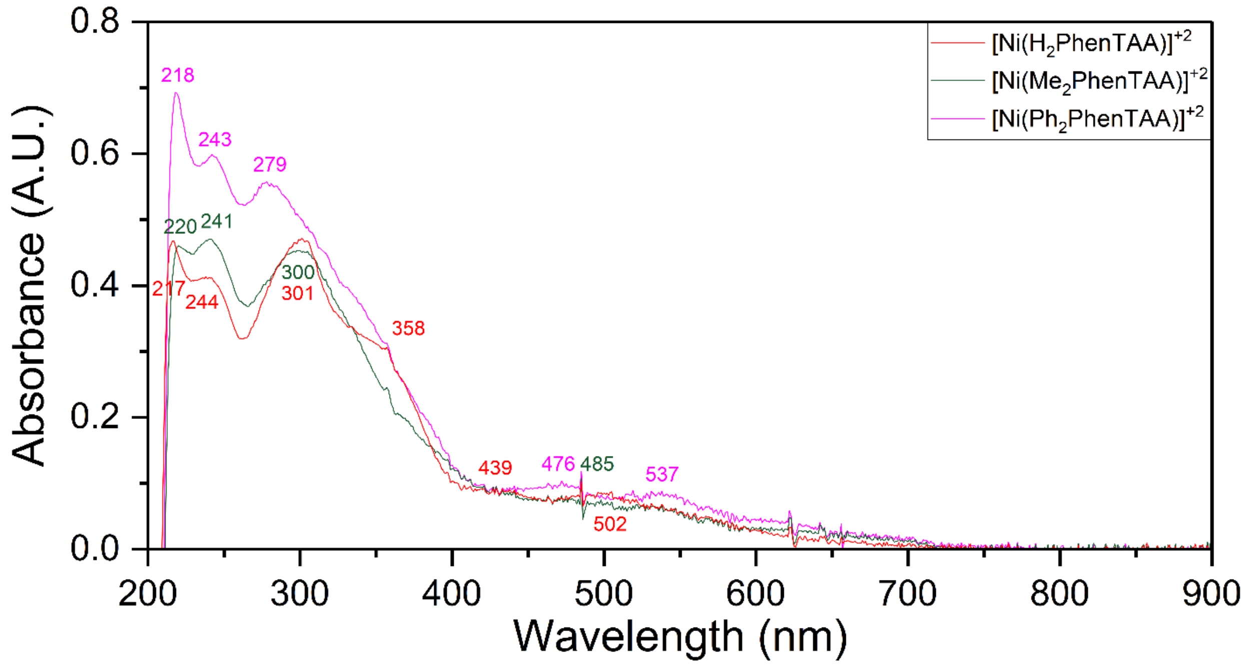

Figure S29. Isolated UV/Vis spectra of the 2e oxidized **[Ni]$^{2+}$** complexes **4a-c** measured via spectro-electrochemistry in CH_2Cl_2.

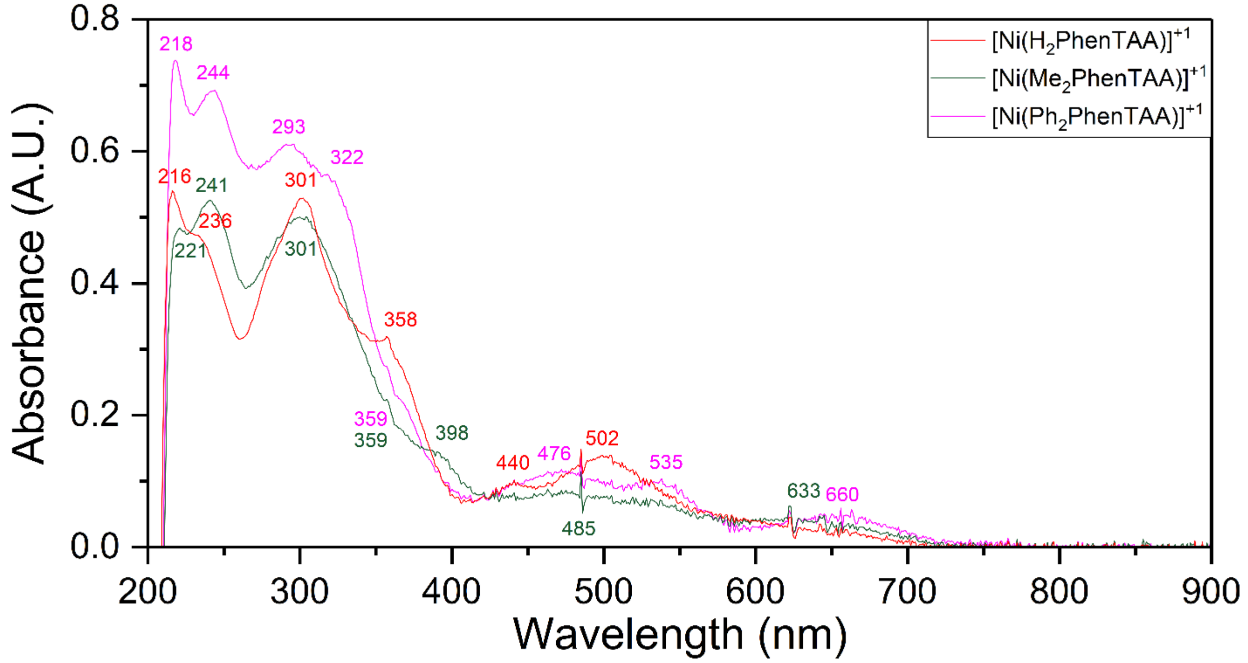

Figure S30. Isolated UV/Vis spectra of the 1e oxidized **[Ni]$^{+}$** complexes **4a-c** measured via spectro-electrochemistry in CH_2Cl_2.

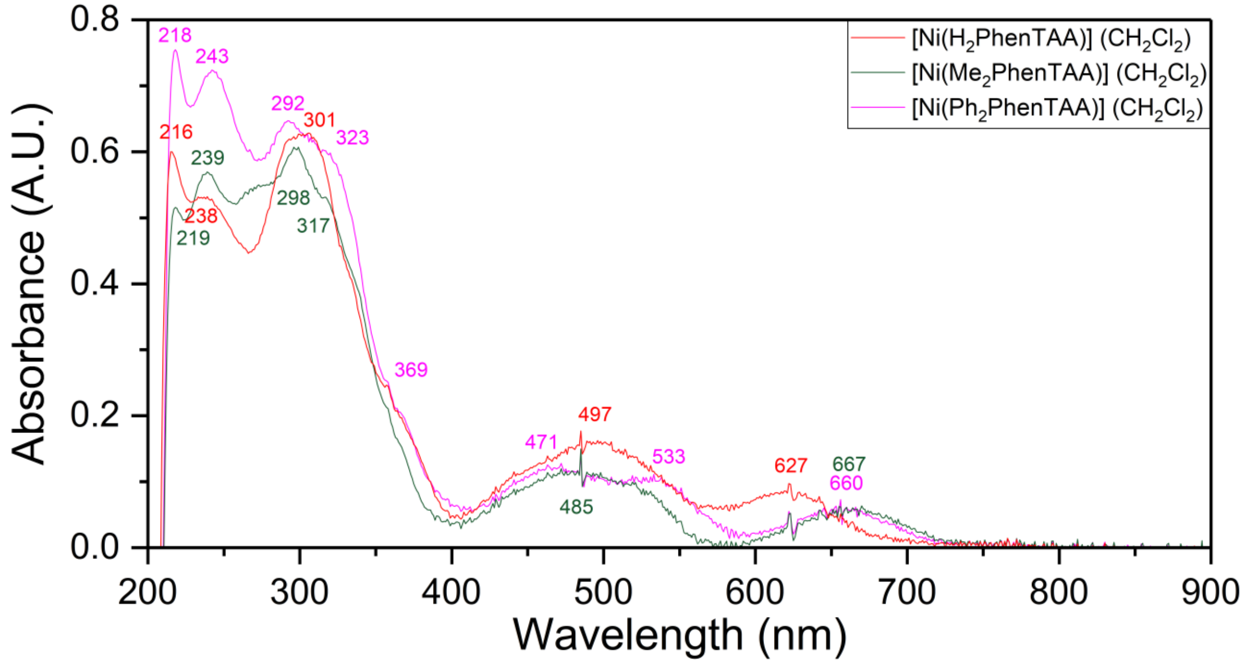

Figure S31. Isolated UV/Vis spectra of the neutral **[Ni]^0** complexes **4a-c** measured in the OTTLE cell in CH_2Cl_2.

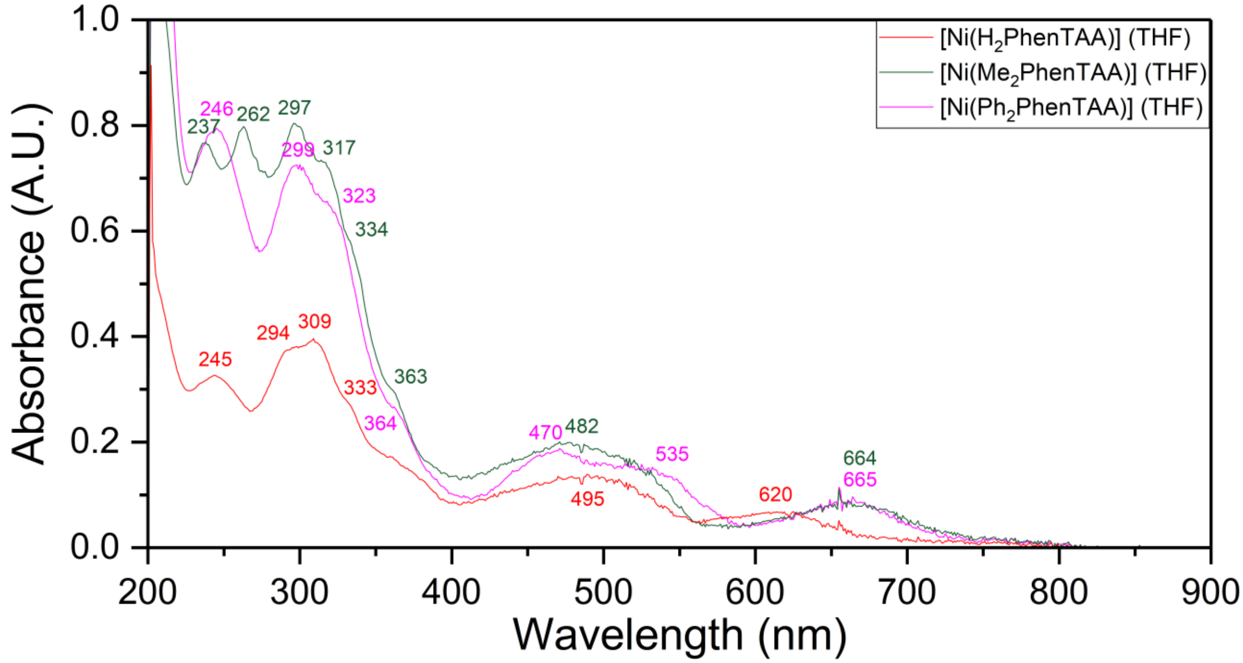

Figure S32. Isolated UV/Vis spectra of the neutral **[Ni]^0** complexes **4a-c** measured in the OTTLE cell in THF.

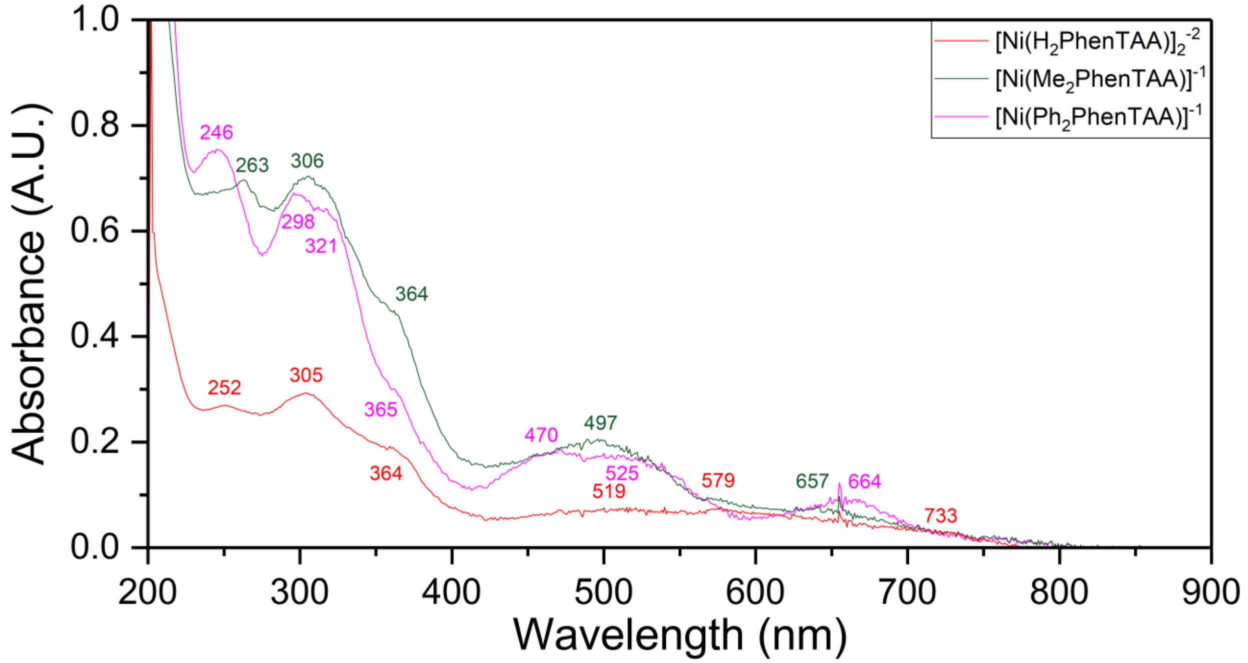

Figure S33. Isolated UV/Vis spectra of the 1e reduced **[Ni]⁻** complexes **4b-c** and dimer **[Ni]$_2$$^{2-}$** measured via spectro-electrochemistry in THF.

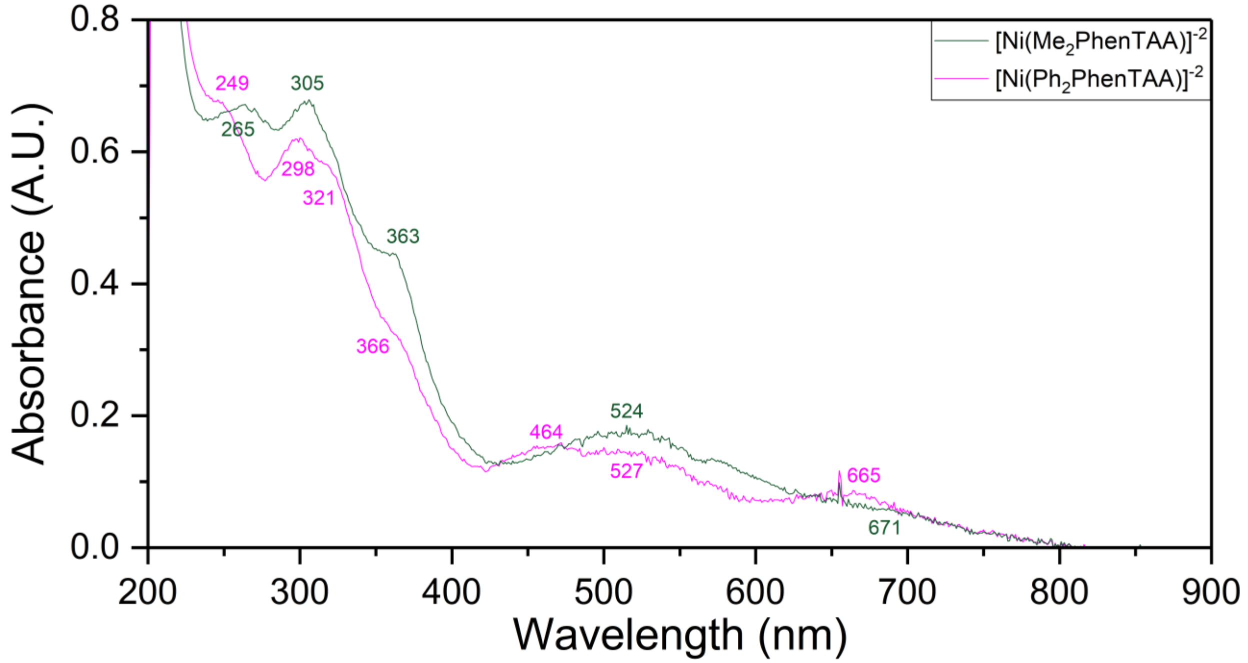

Figure S34. Isolated UV/Vis spectra of the 2e reduced **[Ni]$^{2-}$** complexes **4b-c** measured via spectro-electrochemistry in THF.

EPR studies

Standard procedure for sample preparation (anisotropic measurements)

A quartz EPR tube as outfitted with the EPR sample holder to facilitate sample preparation and was subsequently purged with Ar for at least 15 min in a septum-sealed tube holder together with an EPR-tube cap. The reactions were carried out in separate Schlenk flasks containing 22.5 μmol of [Ni(R_2PhenTAA)] (R = H (10 mg); R = Me (10.6 mg); R = Ph (13.4 mg)). For oxidation reactions, 2 mL of an 11.25 mM stock solution of thianthrenium tetrafluoroborate ($ThiBF_4$) in CH_2Cl_2 was added to the reaction Schlenk flask and was stirred for 5 min. To the quartz EPR tube was added 200 μL of the reaction mixture followed by 200 μL of MeCN. For reduction reactions, 1 mL of a 22.5 mM stock solution of sodium anthracenide in 2-Me-THF was added. The reaction was stirred for 1 min and 400 μL of the reaction mixture was added to the EPR tube. The septum was removed afterwards (under a flow of Ar), the EPR tube was closed with the cap and the sample was flash frozen in liq. N_2. The cap was secured with parafilm and the tube was kept in liquid nitrogen until measured.

Standard procedure for sample preparation (isotropic measurements)

A separate Schlenk flask containing a glass capillary sealed on one side was cycled three times with Ar/vacuum. The reactions were carried out in Schlenk flasks containing 22.5 μmol of [Ni(R_2PhenTAA)] (R = H (10 mg); R = Me (10.6 mg); R = Ph (13.4 mg)). For oxidation reactions, 2 mL of an 11.25 mM stock solution of thianthrenium tetrafluoroborate ($ThiBF_4$) in CH_2Cl_2 was added to the reaction Schlenk flask and was stirred for 5 min. For reduction reactions, 1 mL of a 22.5 mM stock solution of sodium anthracenide in 2-Me-THF was added and the reaction was stirred for 1 min. The reaction mixture was carefully added to the glass capillary via syringe, closed off with vacuum grease and sealed with a burner. The sealed capillary was added to a quartz EPR tube and measured as soon as possible.

Table S7. Experimental EPR parameters of reduced and oxidized complexes **4a–c** derived from spectral simulations,[a],[b] compared to DFT-calculated EPR values. All computed values were obtained with Orca 4.2.1 (B3LYP, ZORA-def2-TZVPP).

	**4a**				**4b**				**4c**			
	g_{11}	g_{22}	g_{33}	g_{iso}	g_{11}	g_{22}	g_{33}	g_{iso}	g_{11}	g_{22}	g_{33}	g_{iso}
**Reduction**												
		**80 K**		**RT**		**80 K**		**RT**		**80 K**		**RT**
Species 1	2.0013	2.0064	2.1043	2.0026	2.0010	2.0061	2.1040	2.0220	2.0149	2.005	2.0868	2.038
Species 2	2.0074	1.9963	2.0844		1.9947	2.0071	2.0853					
Species 3					1.9864	2.0061	2.0625					
A^X								33.5 (^{23}Na)				
Calculated	2.0005	2.0070	2.0400	2.0158	2.0069	2.0154	2.0806	2.0343	2.0032	2.0129	2.0647	2.0269
**Oxidation**												
		**40 K**		**RT**		**80 K**		**RT**		**60 K**		**RT**
Species 1	2.0104	2.0140	1.9789	1.9994	1.9580	2.0044	2.0148	1.9991	1.9855	2.0133	2.0146	2.0004
Species 2					1.9867	2.0153	2.0243		1.9666	2.0034	2.0036	
A^{X_1}[c]					32.1603	0	0					
A^{X_2}[c]					25	0	0					
Calculated	1.9815	2.0048	2.0095	1.9986	1.9668	2.0085	2.0157	1.9970	1.9753	2.0107	2.0647	2.0269

[a] *Spectral simulations performed with Easyspin,[2] using the cwEPR plugin.[3]*
[b] *Hyperfine couplings in MHz.*
[c] *Hyperfine couplings correspond to species 2 for **4b^{+1}** measured at 80 K.*

Isotropic spectra

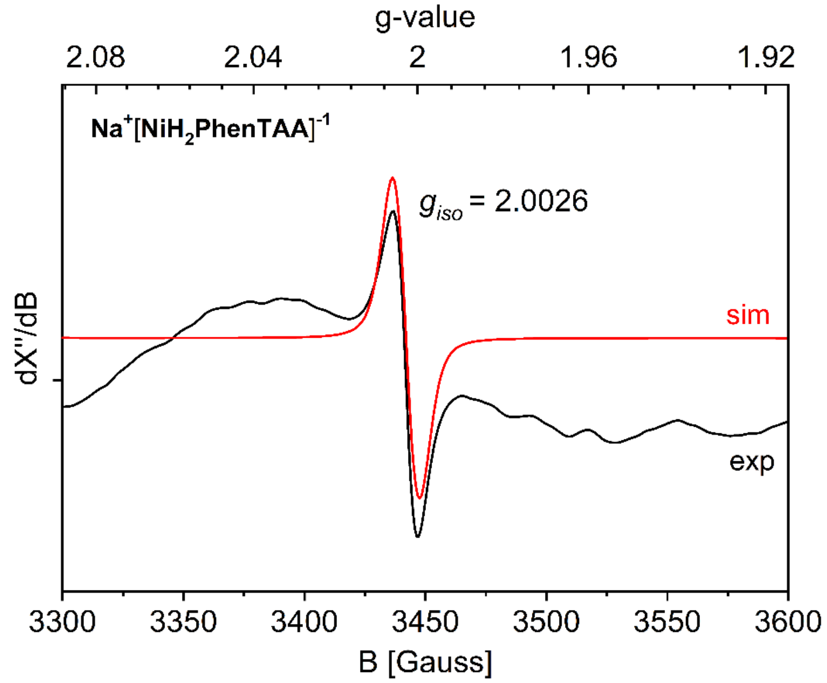

Figure S35. X-band EPR spectrum measured at 293 K of Na[Ni(H_2PhenTAA)], obtained by reduction of [Ni(H_2PhenTAA)] with 1 eq. of sodium anthracenide . Microwave freq. 9.647224 GHz, Mod. Amp. = 8 Gauss, microwave power 6.325 mW. Simulation parameters (garlic): g_{iso} = 2.0026. Note that due to the high modulation amplitude required for signal detection, there is significant baseline distortion.

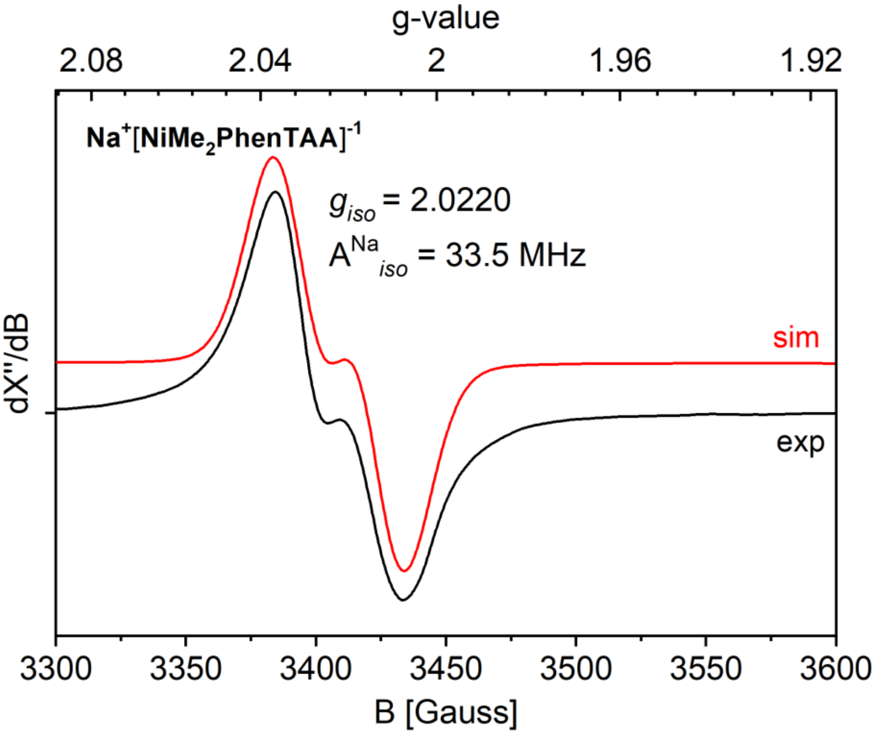

Figure S36. X-band EPR spectrum measured at 293 K of Na[Ni(Me_2PhenTAA)], obtained by reduction of [Ni(Me_2PhenTAA)] with 1 eq. of sodium anthracenide . Microwave freq. 9.646857 GHz, Mod. Amp. = 4 Gauss, microwave power 6.325 mW. Simulation parameters (chili): g_{11} = g_{22} = g_{33} = 2.0220; $A^{23Na}{}_{11}$ = $A^{23Na}{}_{22}$ = A^{23Na} = 33.5 MHz; logtcorr = –16.5.

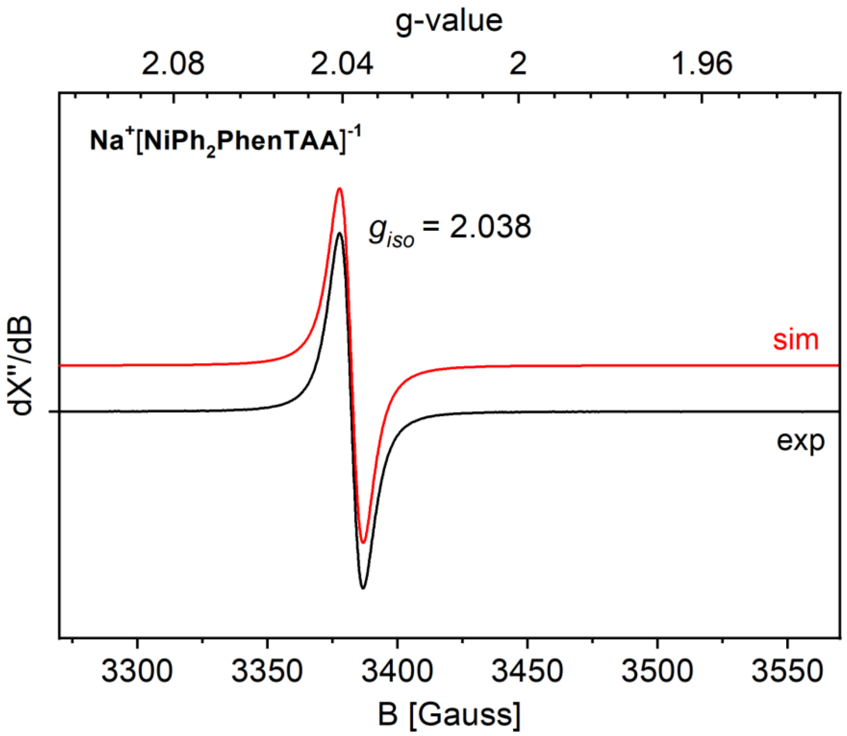

Figure S37. X-band EPR spectrum measured at 293 K of Na[Ni(Ph_2PhenTAA)], obtained by reduction of [Ni(Ph_2PhenTAA)] with 1 eq. of sodium anthracenide . Microwave freq. 9.647637 GHz, Mod. Amp. = 1 Gauss, microwave power 0.6325 mW. Simulation parameters (garlic): g_{iso} = 2.038.

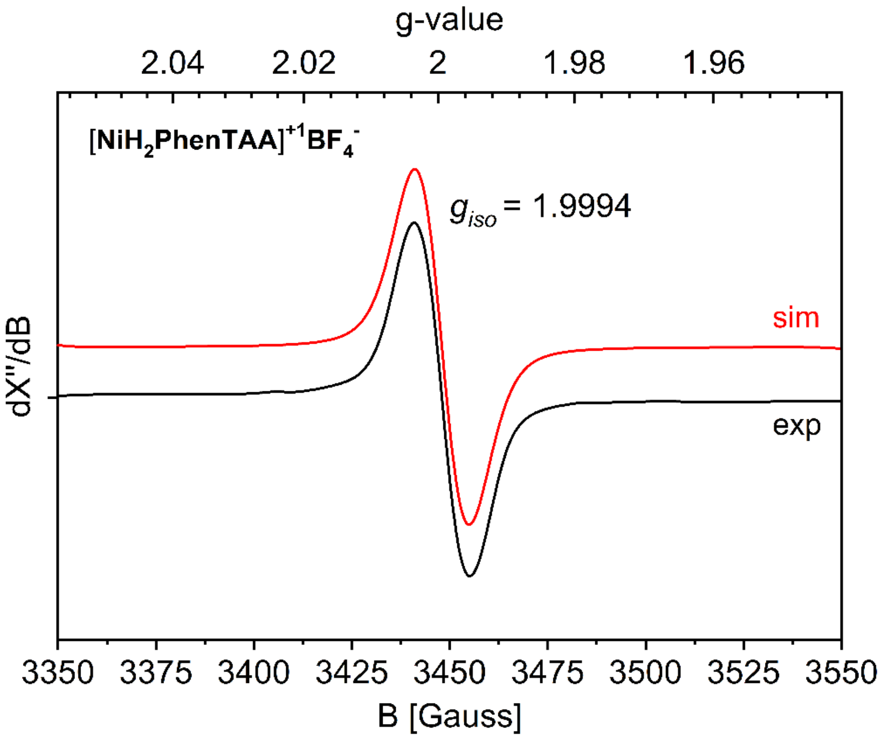

Figure S38. X-band EPR spectrum measured at 293 K of [Ni(H_2PhenTAA)]BF_4, obtained by oxidation of [Ni(H_2PhenTAA)] with 1 eq. of ThiBF_4. Microwave freq. 9.648911 GHz, Mod. Amp. = 4 Gauss, microwave power 6.325 mW. Simulation parameters (garlic): g_{iso} = 1.9994.

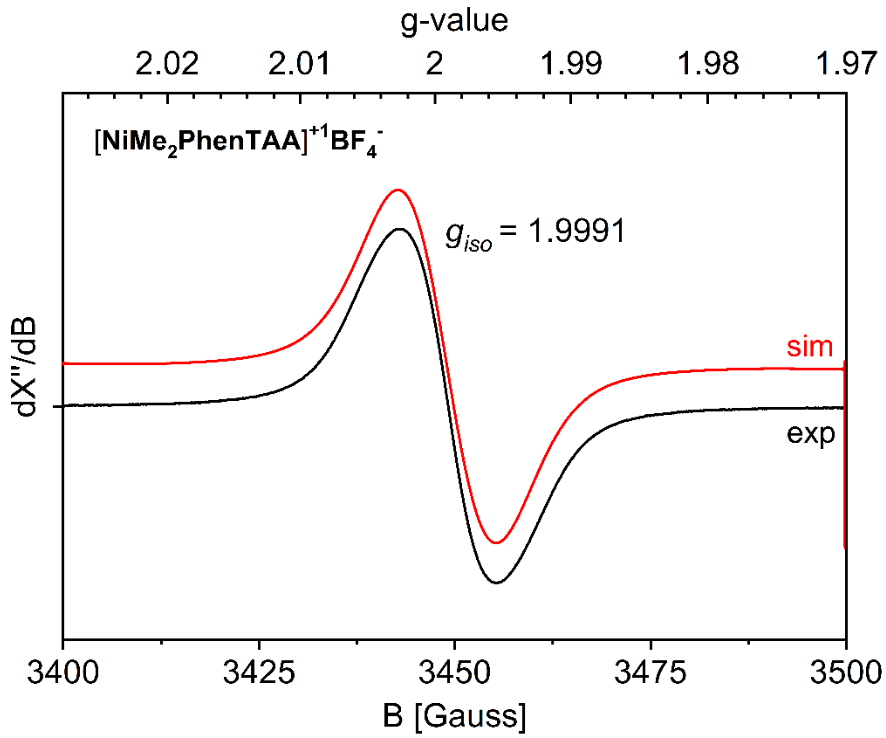

Figure S39. X-band EPR spectrum measured at 293 K of [Ni(Me$_2$PhenTAA)]BF$_4$, obtained by oxidation of [Ni(Me$_2$PhenTAA)] with 1 eq. of ThiBF$_4$. Microwave freq. 9.650816 GHz, Mod. Amp. = 0.1 Gauss, microwave power 6.325 mW. Simulation parameters (garlic): g_{iso} = 1.9991.

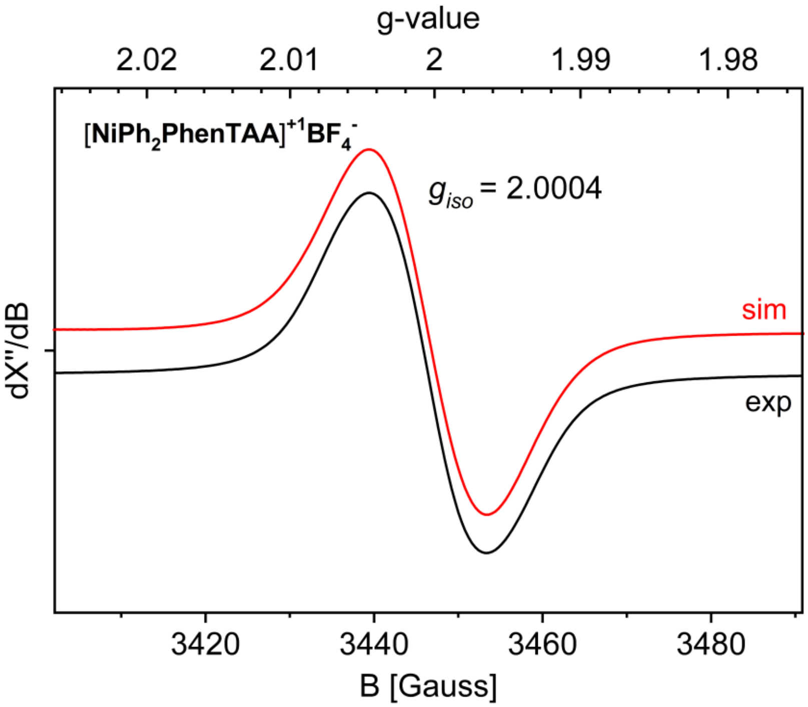

Figure S40. X-band EPR spectrum measured at 293 K of [Ni(Ph$_2$PhenTAA)]BF$_4$, obtained by oxidation of [Ni(Ph$_2$PhenTAA)] with 1 eq. of ThiBF$_4$. Microwave freq. 9.649524 GHz, Mod. Amp. = 4 Gauss, microwave power 6.325 mW. Simulation parameters (garlic): g_{iso} = 2.0004.

Anisotropic spectra

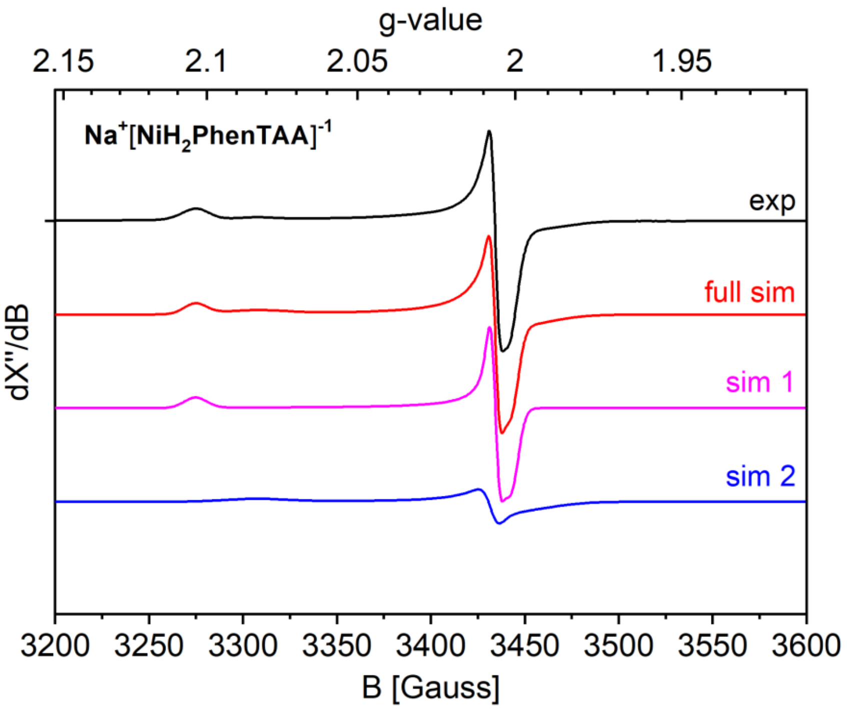

Figure S41. X-band EPR spectrum measured at 80 K of Na[Ni(H_2PhenTAA)], obtained by reduction of [Ni(H_2PhenTAA)] with 1 eq. of sodium anthracenide. Microwave freq. 9.644348 GHz, Mod. Amp. = 4 Gauss, microwave power 2.000 mW. Simulated parameters with separated simulations visualized (pepper): Species 1: g_{11} = 2.0013; g_{22} = 2.0064; g_{33} = 2.1043 (weight = 0.57). Species 2: g_{11} = 2.0074; g_{22} = 1.9963; g_{33} = 2.0844 (weight = 0.43).

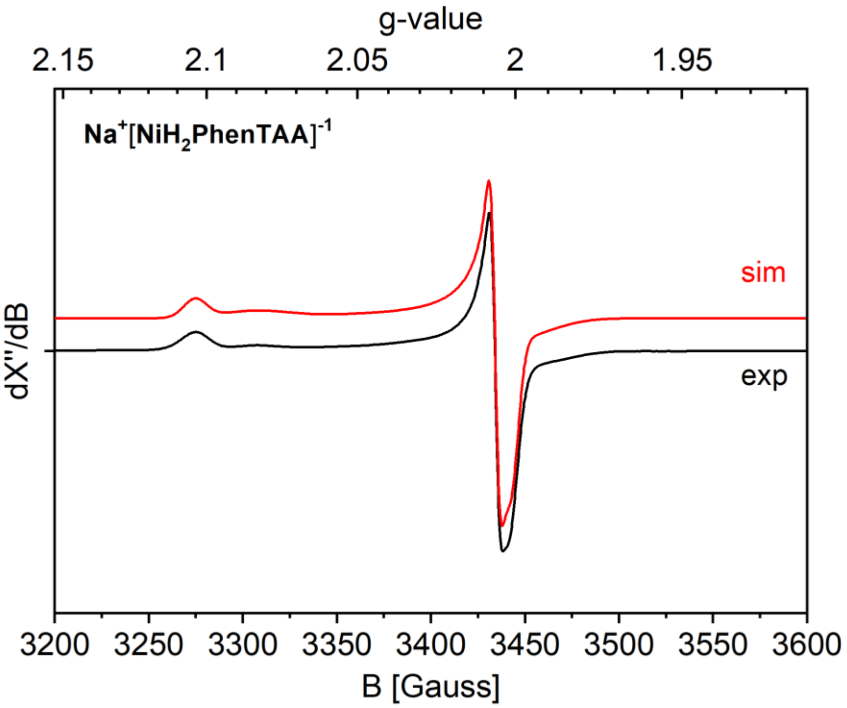

Figure S42. X-band EPR spectrum measured at 80 K of Na[Ni(H_2PhenTAA)], obtained by reduction of [Ni(H_2PhenTAA)] with 1 eq. of sodium anthracenide. Full simulation only (see Figure S41 for details).

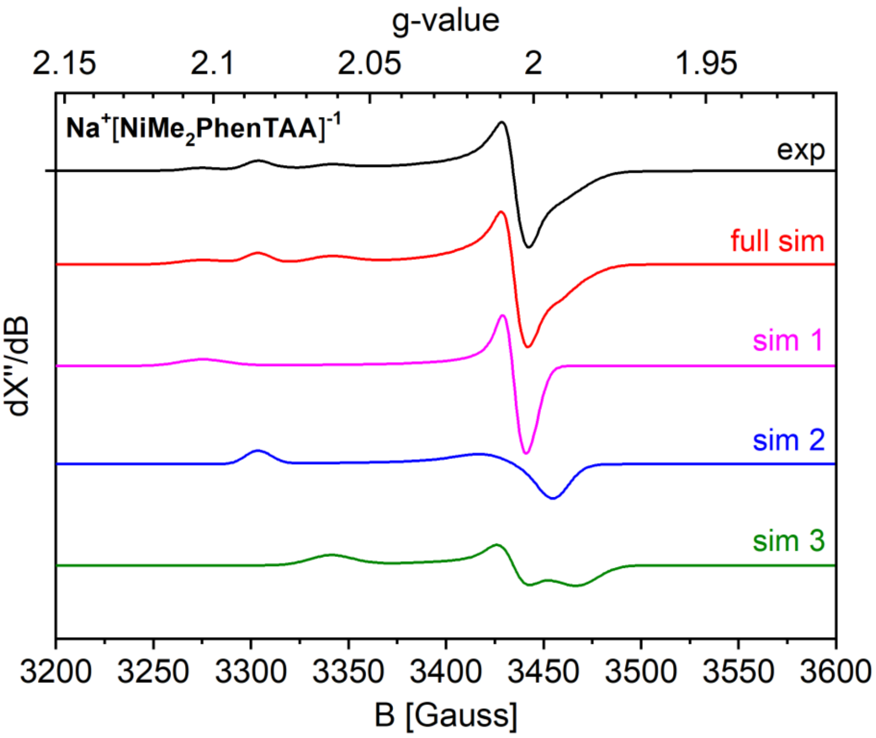

Figure S43. X-band EPR spectrum measured at 80 K of Na[Ni(Me$_2$PhenTAA)], obtained by reduction of [Ni(Me$_2$PhenTAA)] with 1 eq. of sodium anthracenide. Microwave freq. 9.643138 GHz, Mod. Amp. = 8 Gauss, microwave power 2.000 mW. Simulated parameters with separated simulations visualized (pepper): Species 1: g_{11} = 2.001; g_{22} = 2.0061; g_{33} = 2.104 (weight = 0.34). Species 2: g_{11} = 1.9947; g_{22} = 2.0071; g_{33} = 2.0853 (weight = 0.37). Species 3: g_{11} = 1.9864; g_{22} = 2.0061; g_{33} = 2.0625 (weight = 0.29).

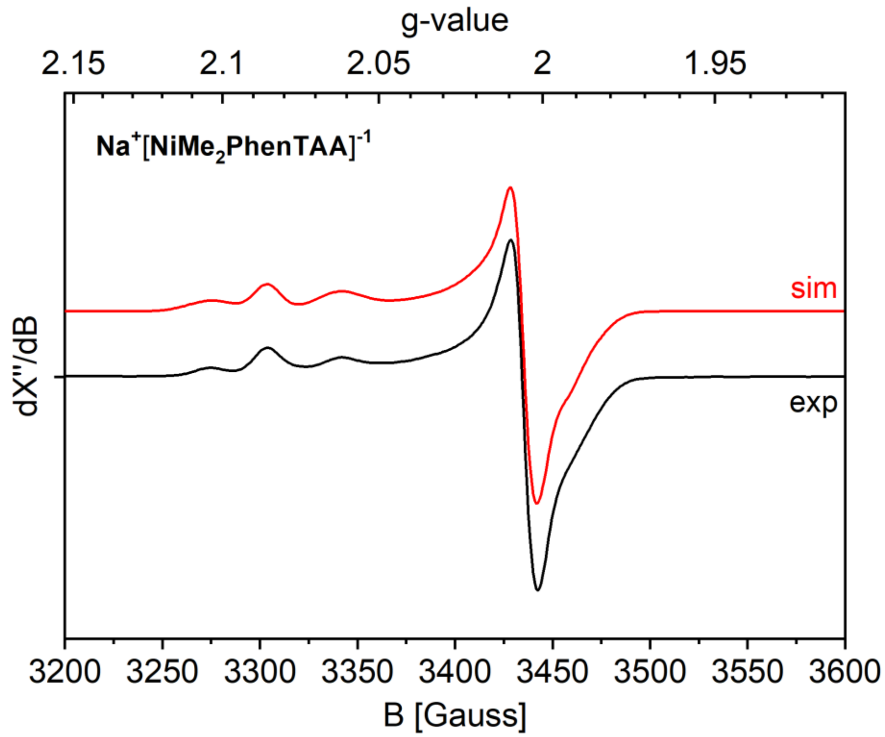

Figure S44. X-band EPR spectrum measured at 80 K of Na[Ni(Me$_2$PhenTAA)], obtained by reduction of [Ni(Me$_2$PhenTAA)] with 1 eq. of sodium anthracenide. Full simulation only (see Figure S43 for details).

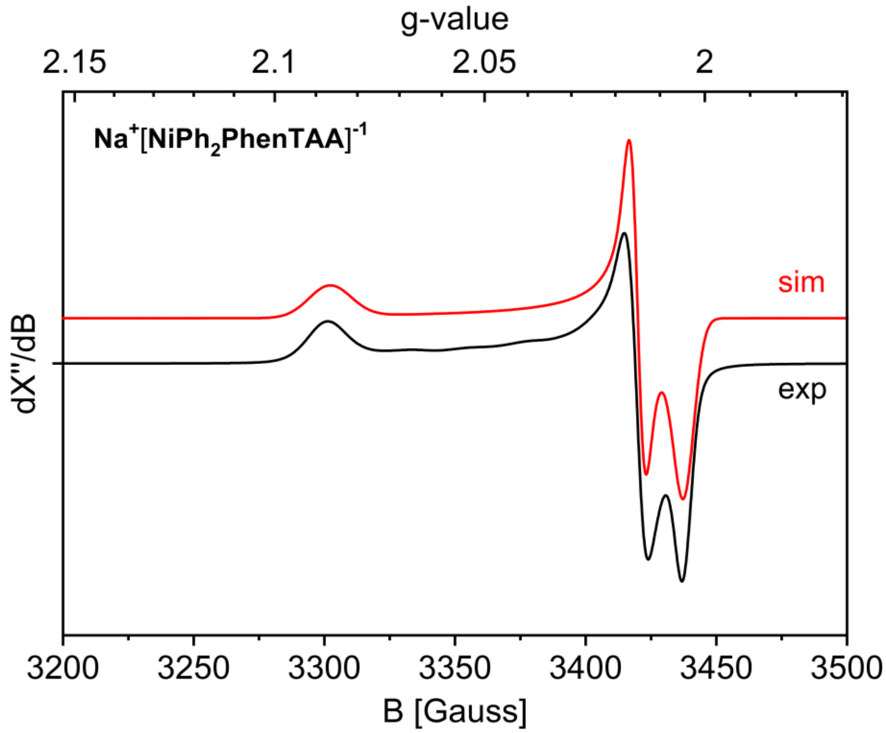

Figure S45. X-band EPR spectrum measured at 80 K of Na[Ni(Me$_2$PhenTAA)], obtained by reduction of [Ni(Me$_2$PhenTAA)] with 1 eq. of sodium anthracenide. Microwave freq. 9.644217 GHz, Mod. Amp. = 2 Gauss, microwave power 0.6325 mW. Simulation parameters (pepper):g_{11} = 2.0149; g_{22} = 2.005; g_{33} = 2.0868.

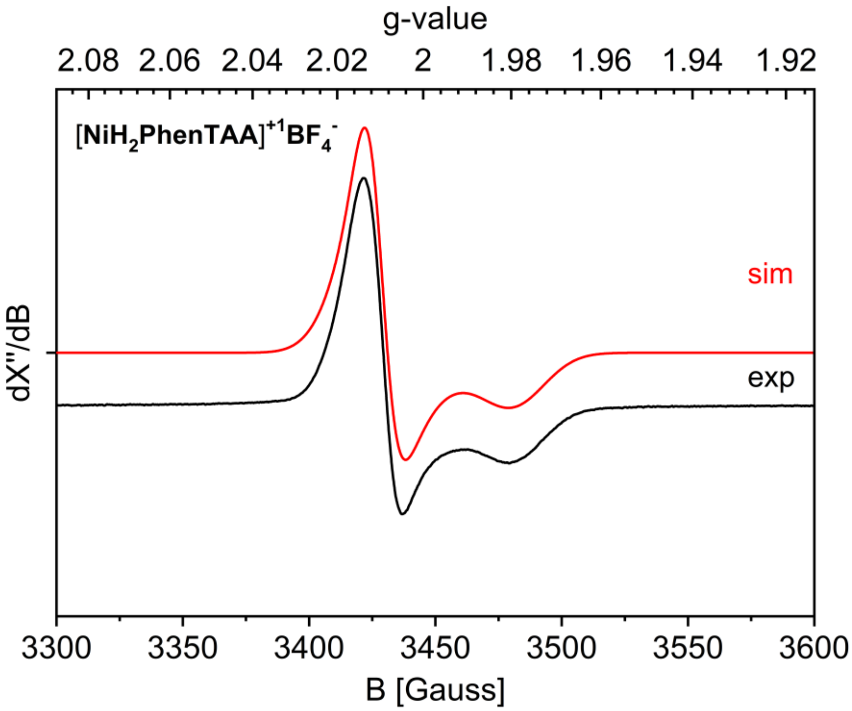

Figure S46. X-band EPR spectrum measured at 40 K of [Ni(H$_2$PhenTAA)]BF$_4$, obtained by oxidation of [Ni(H$_2$PhenTAA)] with 1 eq. of ThiBF$_4$. Microwave freq. 9.643109 GHz, Mod. Amp. = 1 Gauss, microwave power 2.000 mW. Simulation parameters (pepper):g_{11} = 2.0104; g_{22} = 2.014; g_{33} = 1.9789.

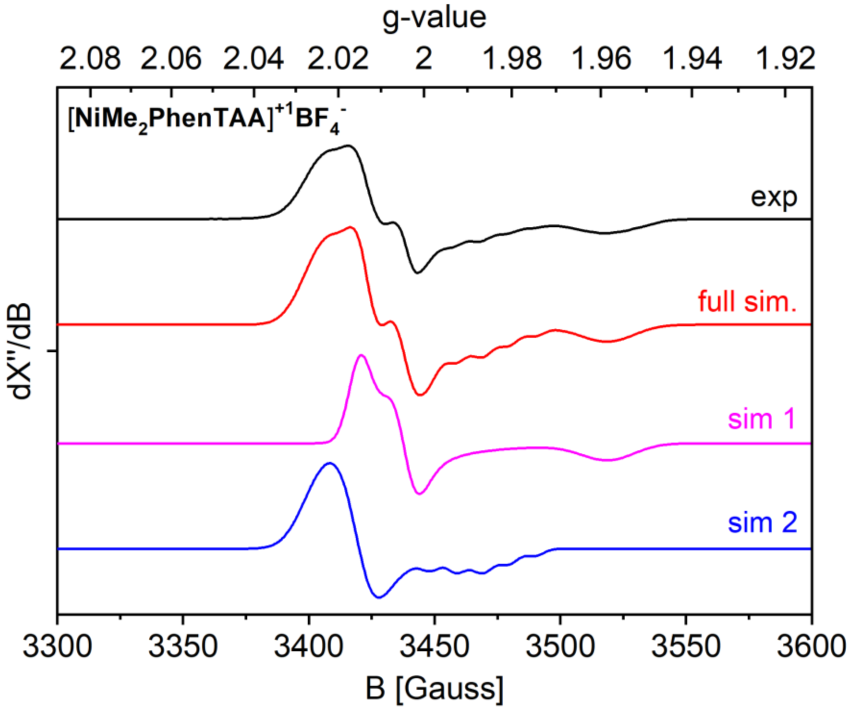

Figure S47. X-band EPR spectrum measured at 80 K of [Ni(Me$_2$PhenTAA)]BF$_4$, obtained by oxidation of [Ni(Me$_2$PhenTAA)] with 1 eq. of ThiBF$_4$. Microwave freq. 9.645636 GHz, Mod. Amp. = 4 Gauss, microwave power 2.000 mW. Simulated parameters with separated simulations visualized (pepper): Species 1: g_{11} = 1.958; g_{22} = 2.0044; g_{33} = 2.0148 (weight = 0.50). Species 2: g_{11} =1.9867; g_{22} = 2.0153; g_{33} = 2.0243. $A^{14N1}{}_{11}$ = 32.1603 MHz; $A^{14N1}{}_{22}$ = 0; $A^{14N1}{}_{33}$ = 0. $A^{14N2}{}_{11}$ = 25; $A^{14N2}{}_{22}$ = 0; $A^{14N2}{}_{33}$ = 0 (weight = 0.50).

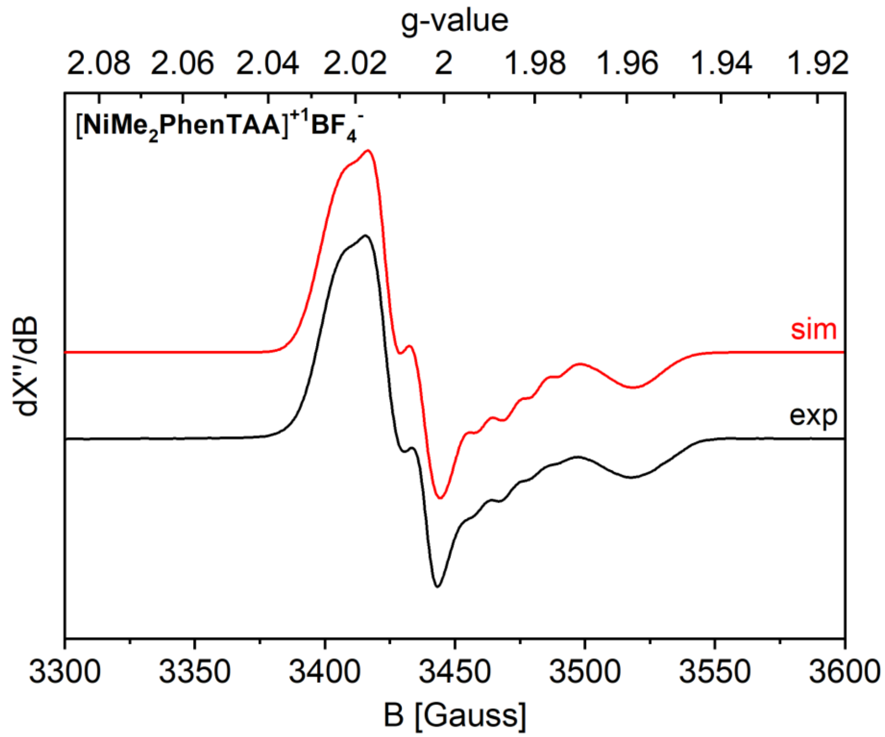

Figure S48. X-band EPR spectrum measured at 80 K of [Ni(Me_2PhenTAA)]BF_4, obtained by oxidation of [Ni(Me_2PhenTAA)] with 1 eq. of ThiBF_4. Full simulation only (see Figure S47 for details).

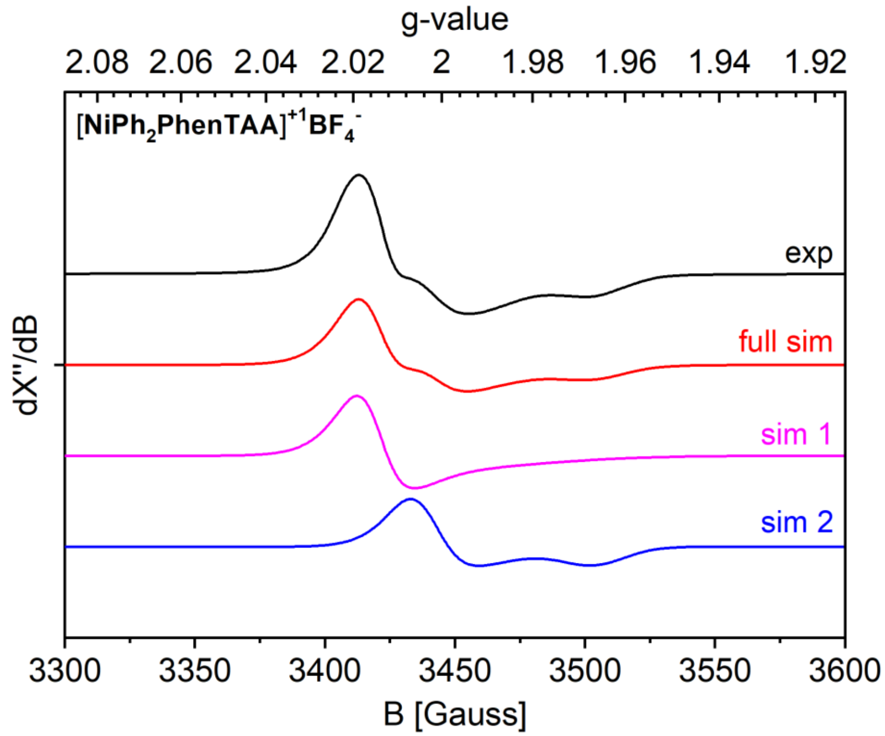

Figure S49. X-band EPR spectrum measured at 60 K of [Ni(Ph_2PhenTAA)]BF_4, obtained by oxidation of [Ni(Ph_2PhenTAA)] with 1 eq. of ThiBF_4. Microwave freq. 9.643381 GHz, Mod. Amp. = 4 Gauss, microwave power 2.000 mW. Simulated parameters with separated simulations visualized (pepper): Species 1: g_{11} = 1.9855; g_{22} = 2.0133; g_{33} = 2.0146 (weight = 0.63). Species 2: g_{11} =1.9666; g_{22} = 2.0034; g_{33} = 2.0036 (weight = 0.37).

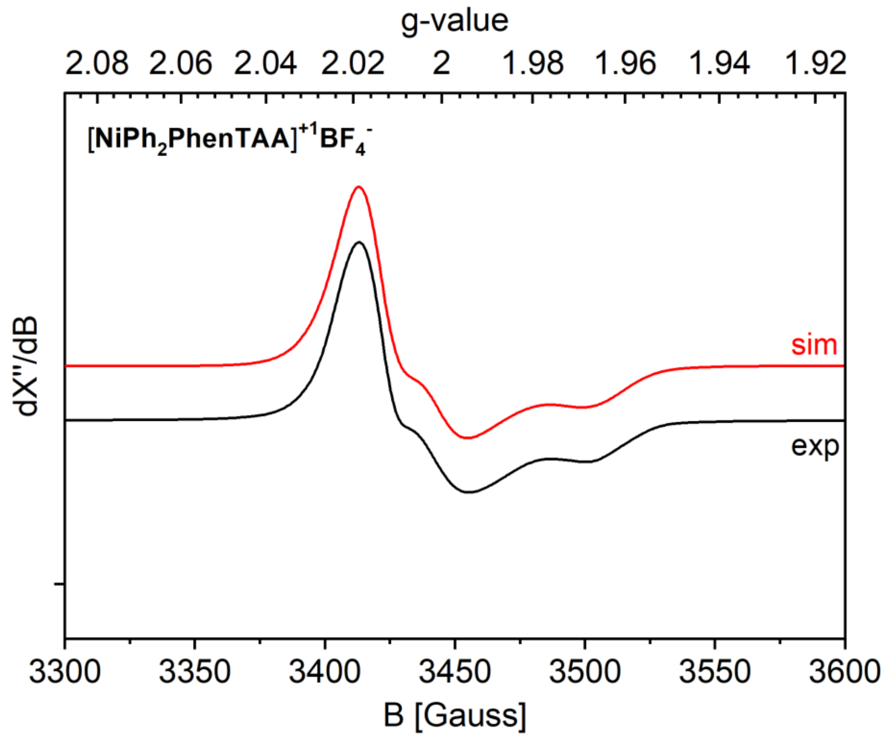

Figure S50. X-band EPR spectrum measured at 60 K of [Ni(Ph_2PhenTAA)]BF_4, obtained by oxidation of [Ni(Ph_2PhenTAA)] with 1 eq. of $ThiBF_4$. Full simulation only (see Figure S49 for details).

Mass spectra (ESI⁺–MS):

***N,N'*-(1,2-phenylenediamino)-bis(2-aminobenzaldehyde) (2a):**

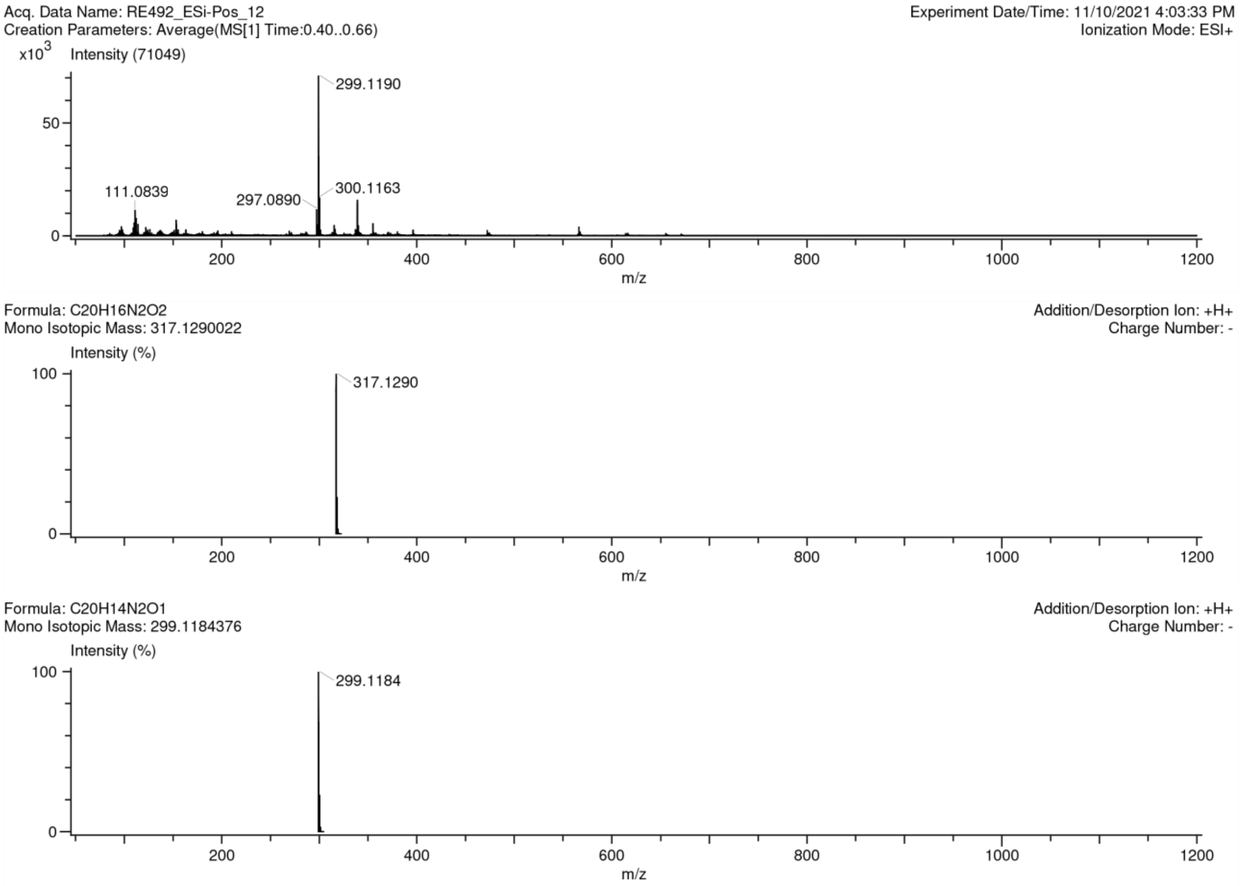

Figure S51. ESI–MS measurement (positive mode) of **2a** (top) and simulation of $C_{20}H_{16}N_2O_2$ *m/z* = 317.1290 Da (middle) and $C_{20}H_{14}N_2O_1$ *m/z* = 299.1184 Da (bottom). (σ = 2.005895 ppm).

N,N'-(1,2-phenylenediamino)-bis(2-aminoacetophenone) (2b):

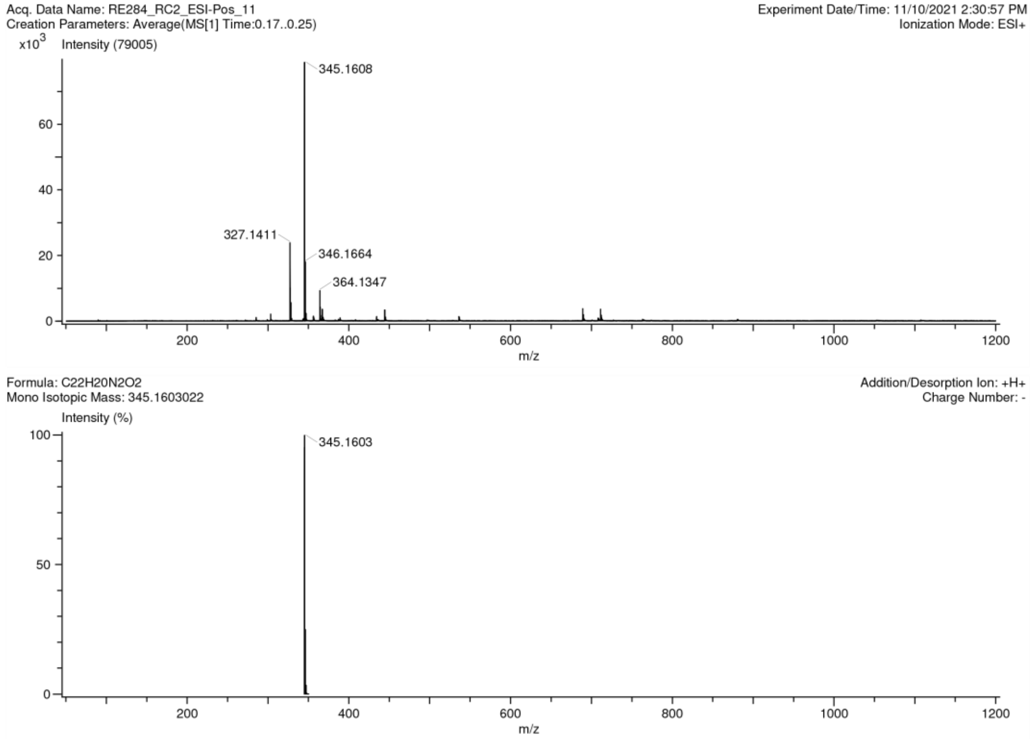

Figure S52. ESI–MS measurement (positive mode) of **2b** (top) and simulation of $C_{22}H_{20}N_2O_2$ *m/z* = 345.1603 Da. (σ = 1.448602 ppm).

N,N'-(1,2-phenylenediamino)-bis(2-aminobenzophenone) (2c):

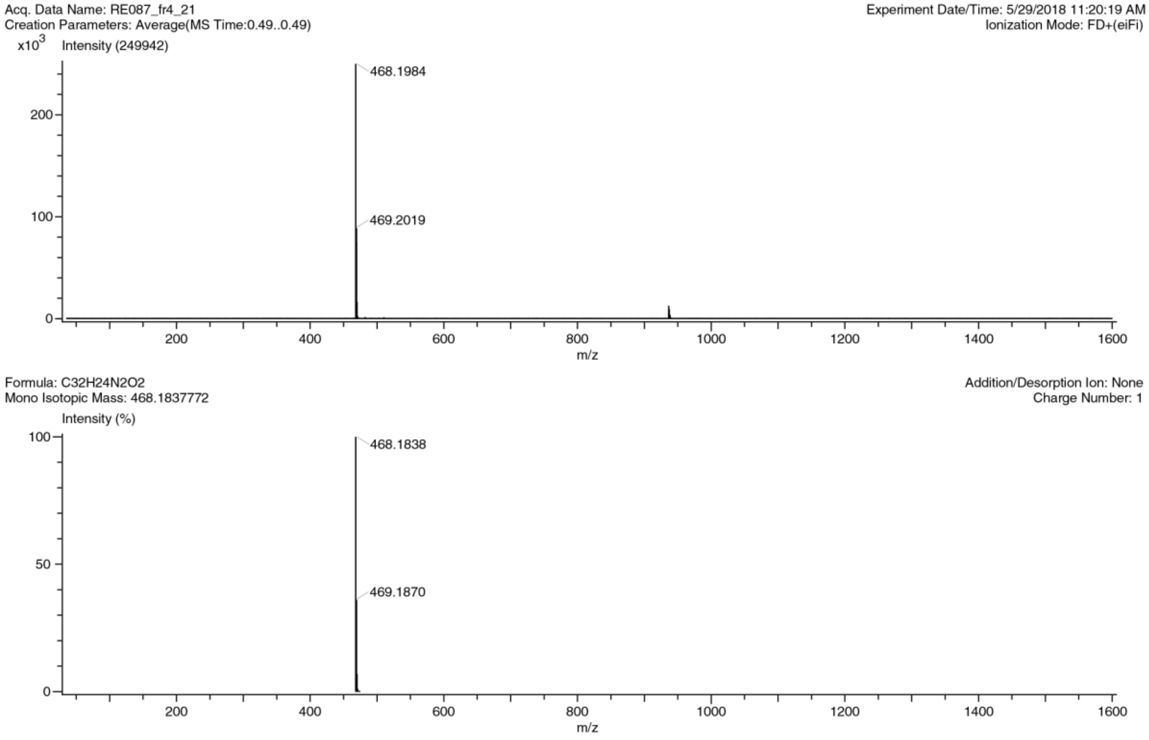

Figure S53. ESI–MS measurement (positive mode) of **2c** (top) and simulation of $C_{32}H_{24}N_2O_2$ *m/z* = 469.1838 Da.

$H_2(H_2PhenTAA)$ (3a):

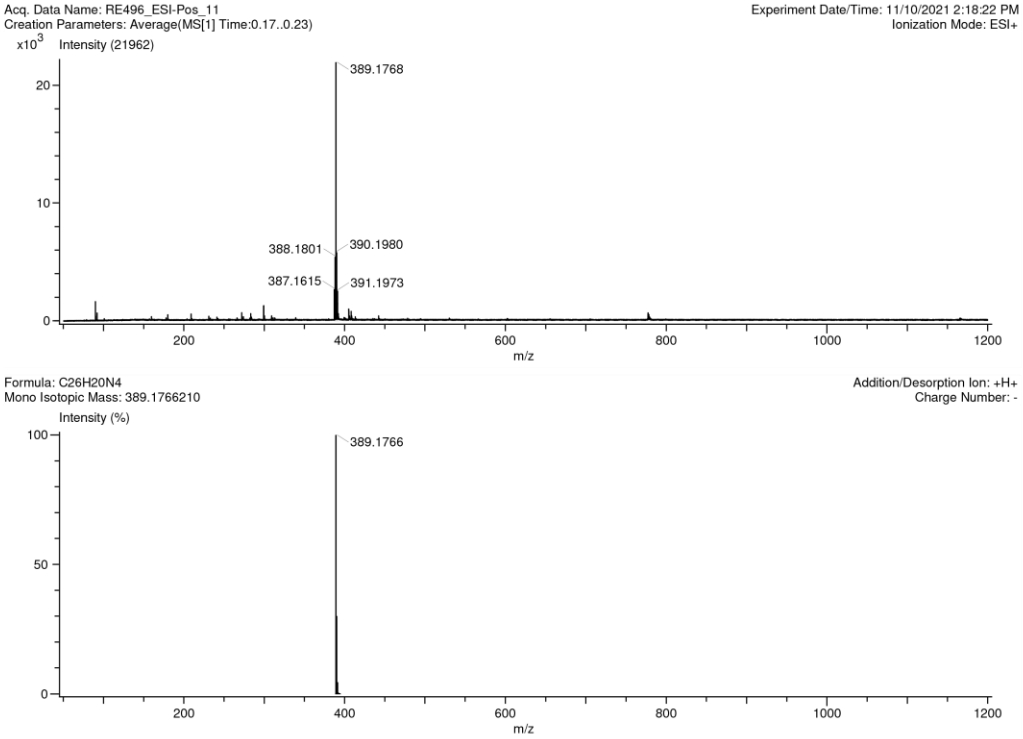

Figure S54. ESI–MS measurement (positive mode) of **3a** (top) and simulation of $C_{26}H_{20}N_4$ *m/z* = 389.1766 Da. (σ = 0.513906 ppm).

$H_2(Me_2PhenTAA)$ (3b)

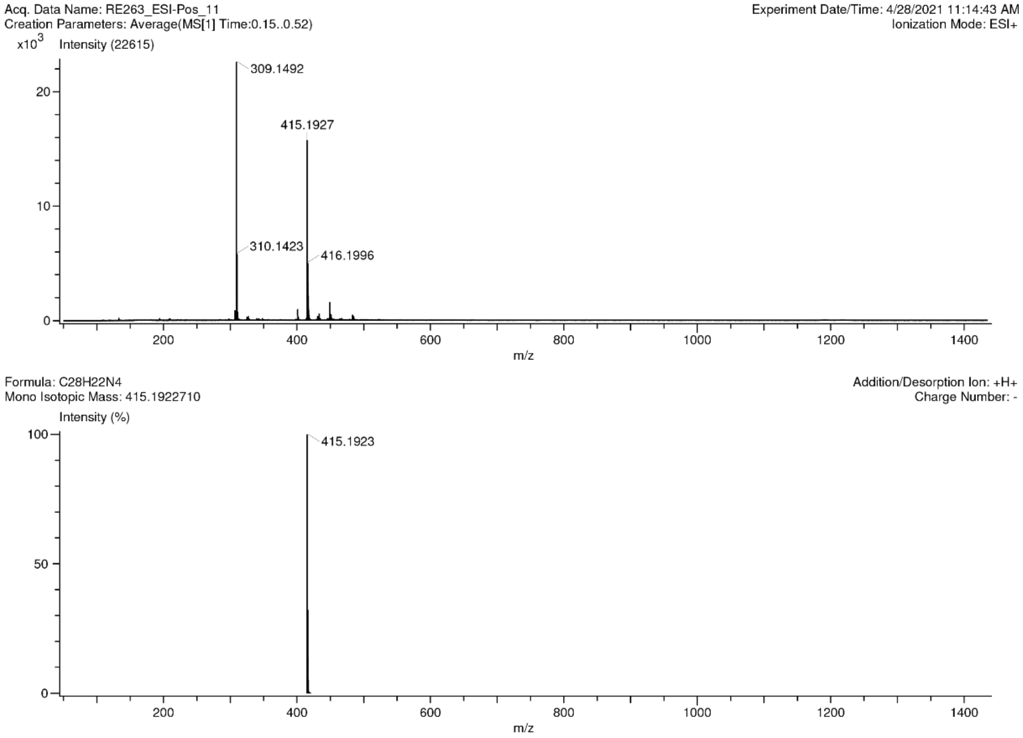

Figure S55. ESI–MS measurement (positive mode) of **3b** (top) and simulation of $C_{28}H_{22}N_4$ *m/z* = 415.1923 Da. (σ = 0.963409 ppm).

Ni(H_2PhenTAA) (4a):

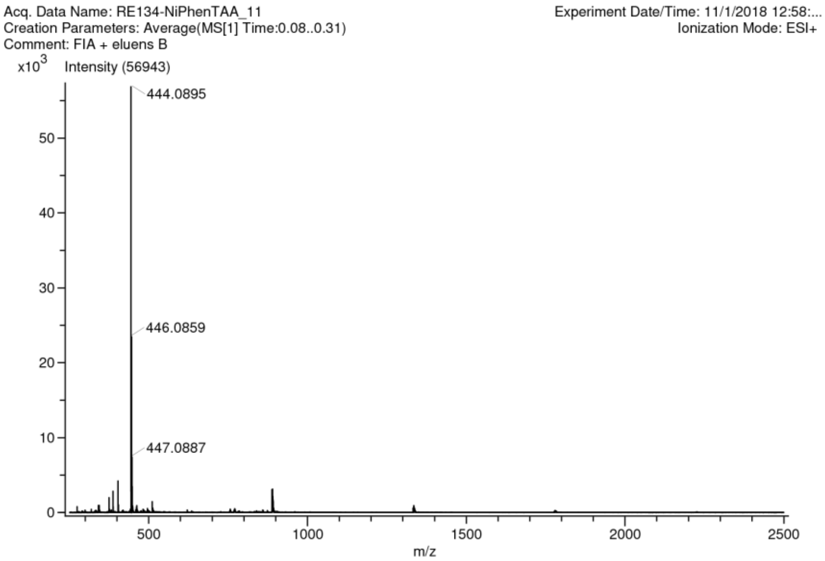

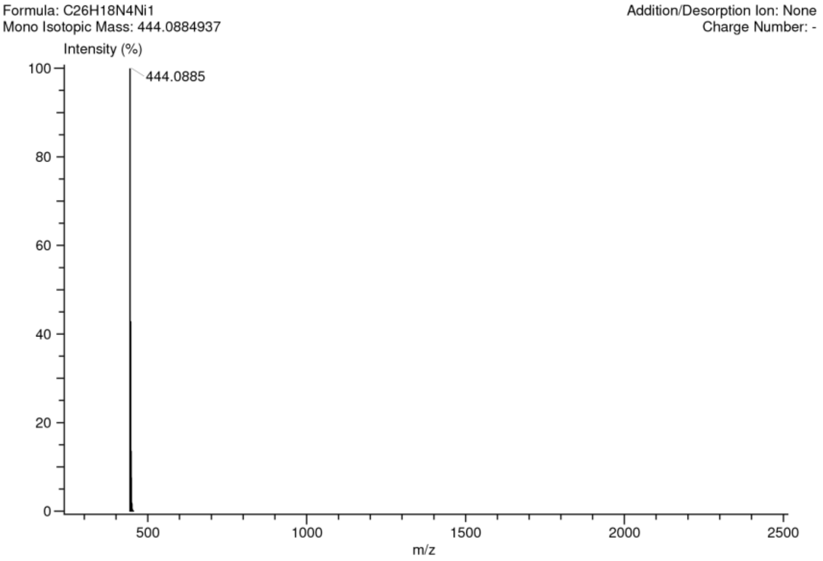

Figure S56. ESI–MS measurement (positive mode) of **4a** (top) and simulation of $C_{26}H_{18}N_4Ni$ *m/z* = 444.0885 Da. *(σ =* 2.251803 ppm).

Ni(Me_2PhenTAA) (4b):

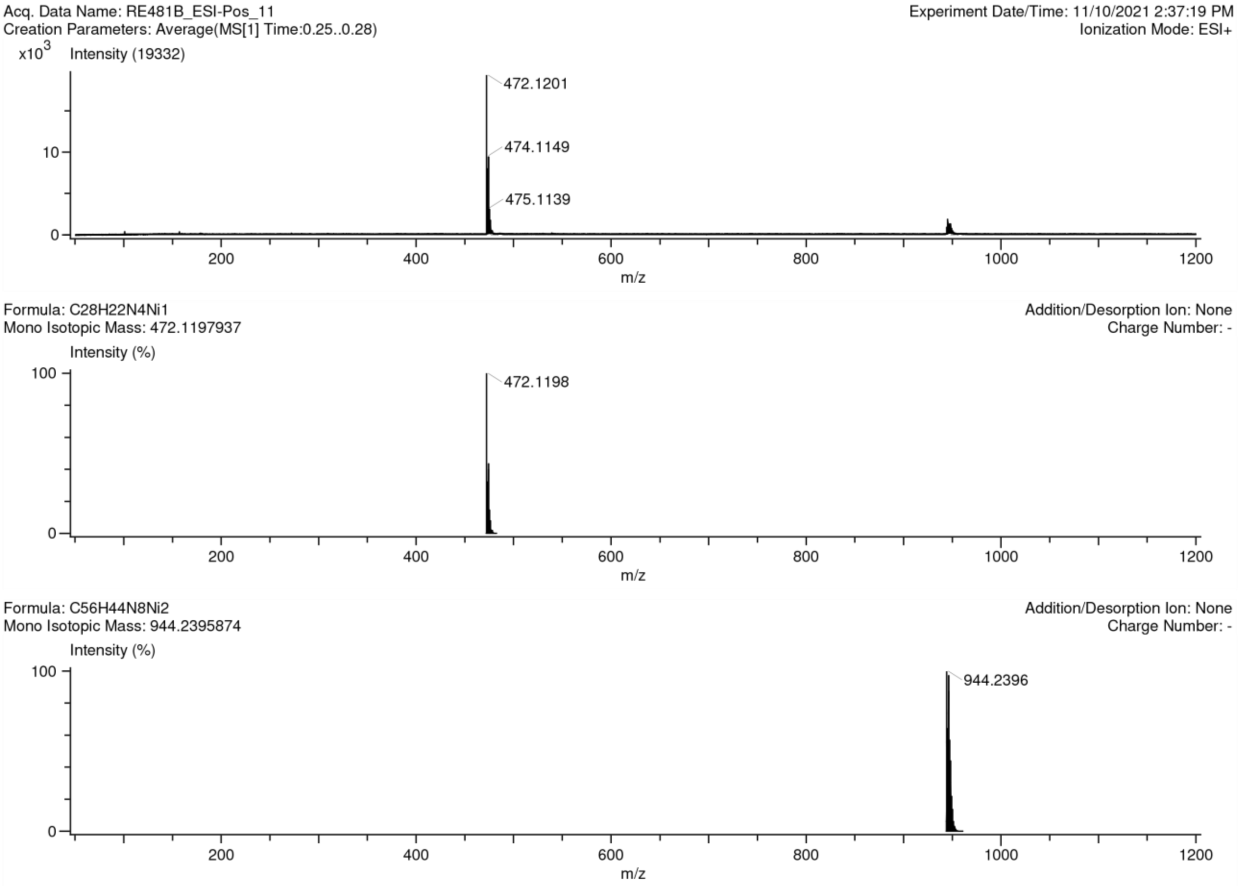

Figure S57. ESI–MS measurement (positive mode) of **4b** (top) and simulation of $C_{28}H_{22}N_4Ni$ *m/z* = 444.0885 Da (middle) and $C_{56}H_{44}N_8Ni_2$ *m/z* = 944.2396 (bottom). *(* σ = 0.635432 ppm for 472.1201 *m/z).*

Ni(Ph$_2$PhenTAA) (4c):

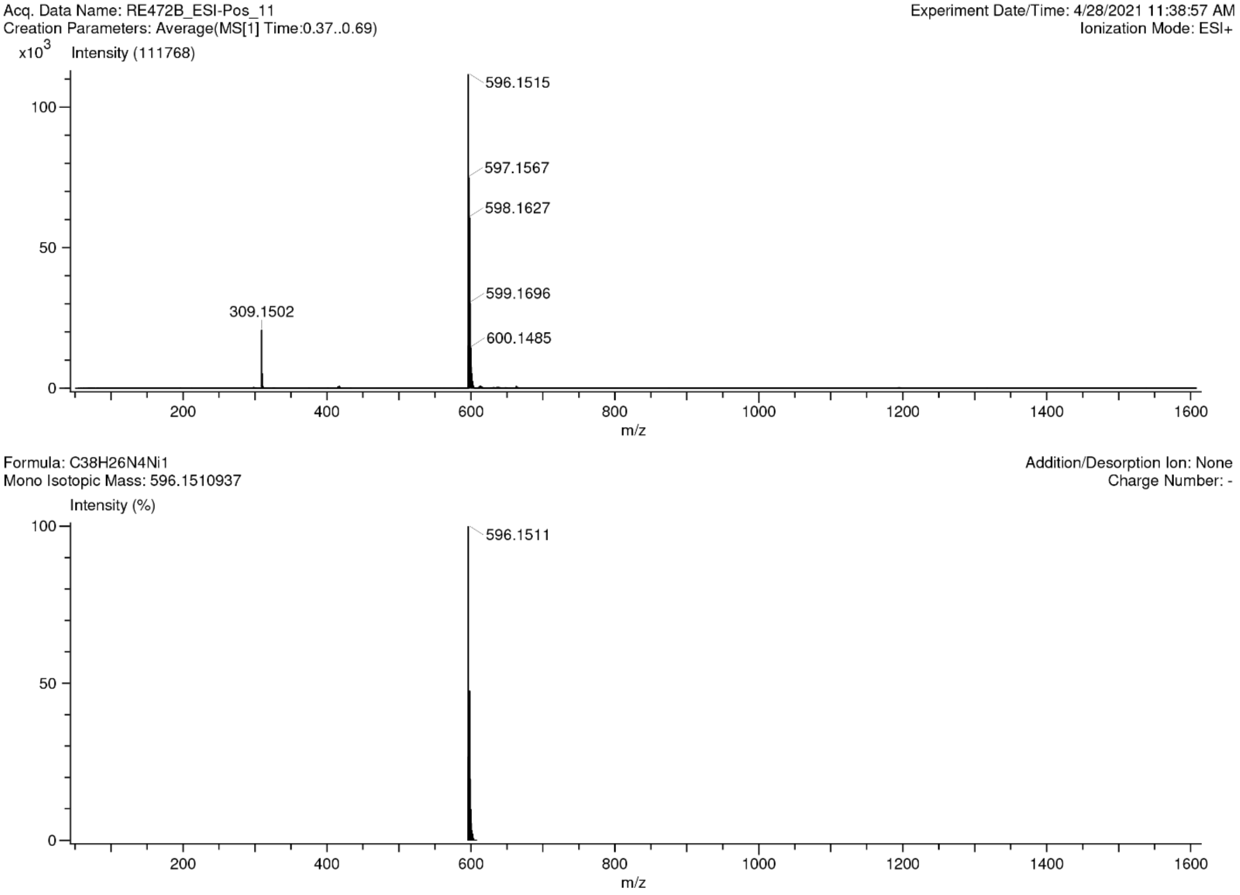

Figure S58. ESI–MS measurement (positive mode) of **4c** (top) and simulation of $C_{38}H_{26}N_4Ni$ *m/z* = 596.1511 Da. (σ = 0.670971 ppm).

Copies of the NMR spectra (Characterization):

2-aminobenzaldehyde (1a)

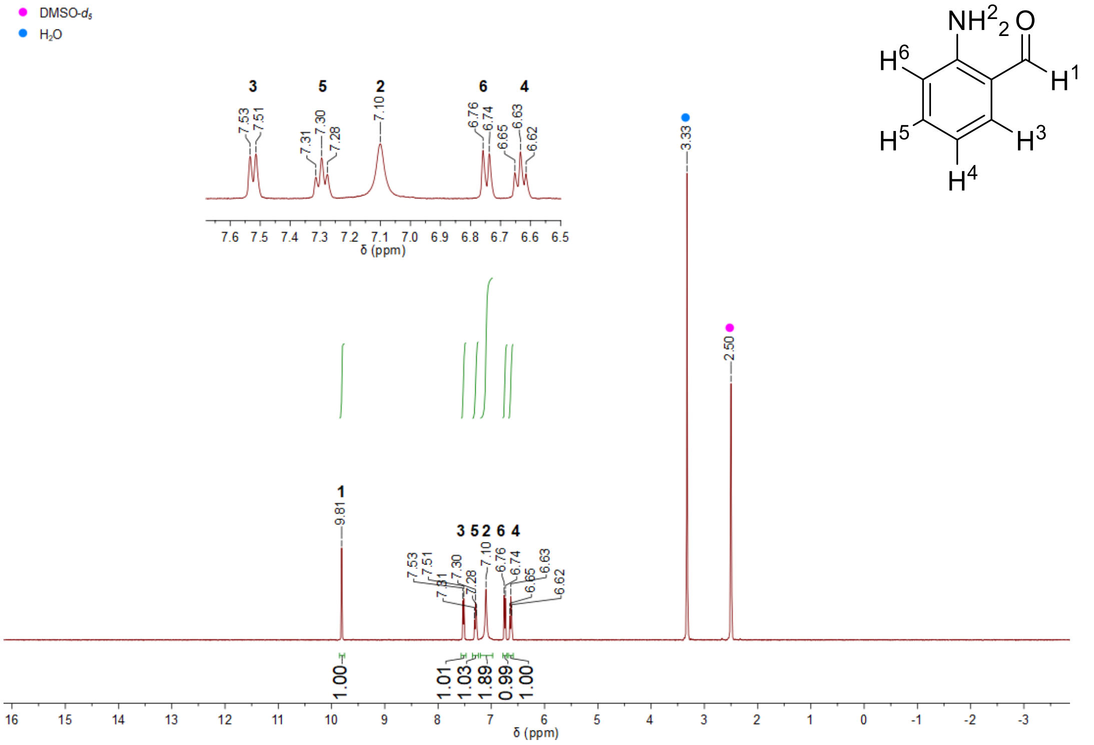

Figure S59. ^{1}H-NMR spectrum of **1a** in DMSO-d_6 (400 MHz).

N,N'-(1,2-phenylenediamino)-bis(2-aminobenzaldehyde) (2a)

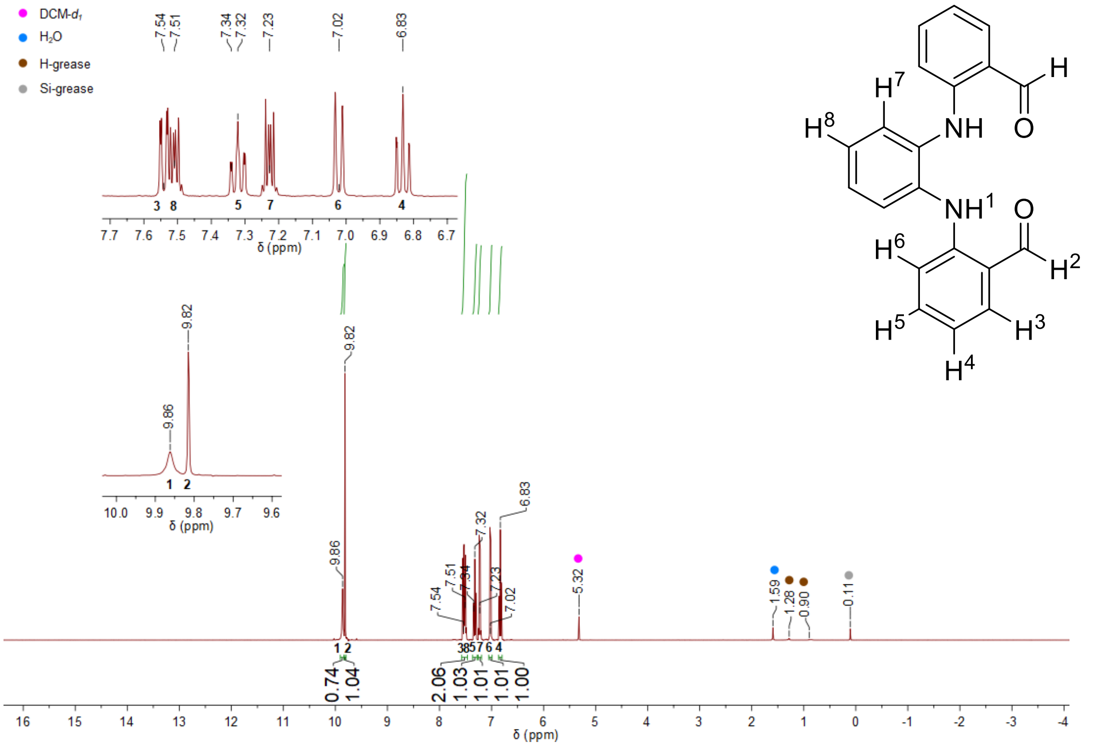

Figure S60. ^{1}H-NMR spectrum of **2a** in CD_2Cl_2 (400 MHz).

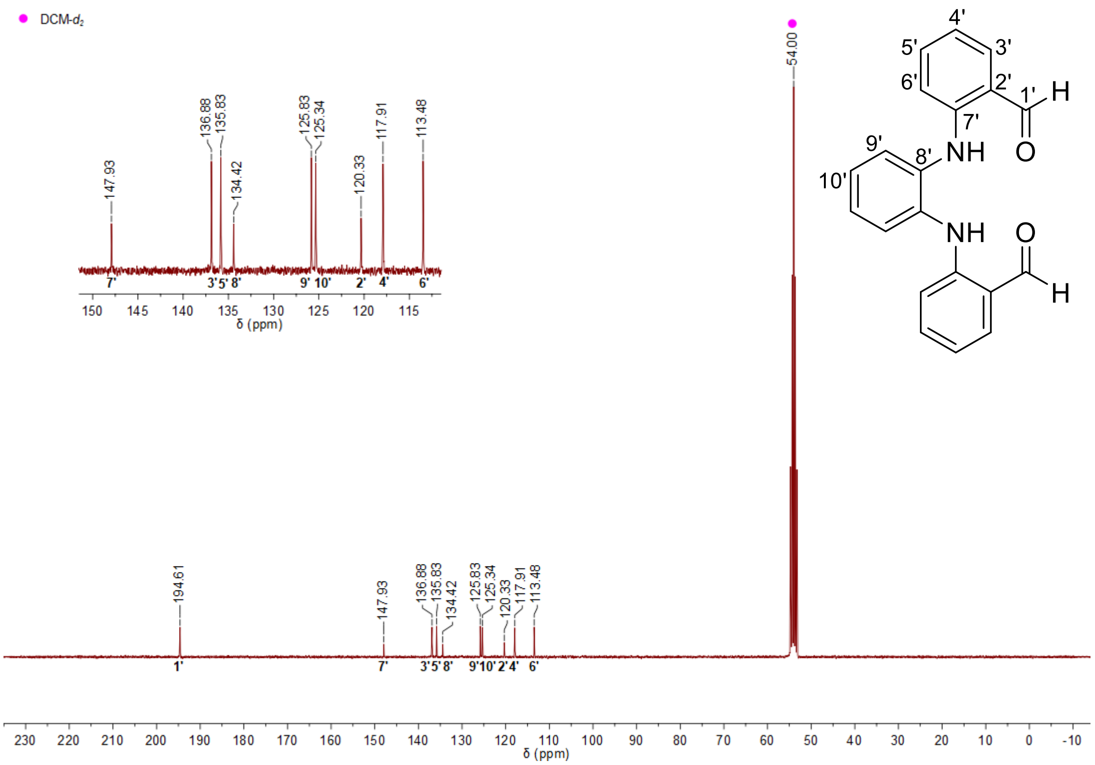

Figure S61. ^{13}C-NMR spectrum of **2a** in CD_2Cl_2 (126 MHz).

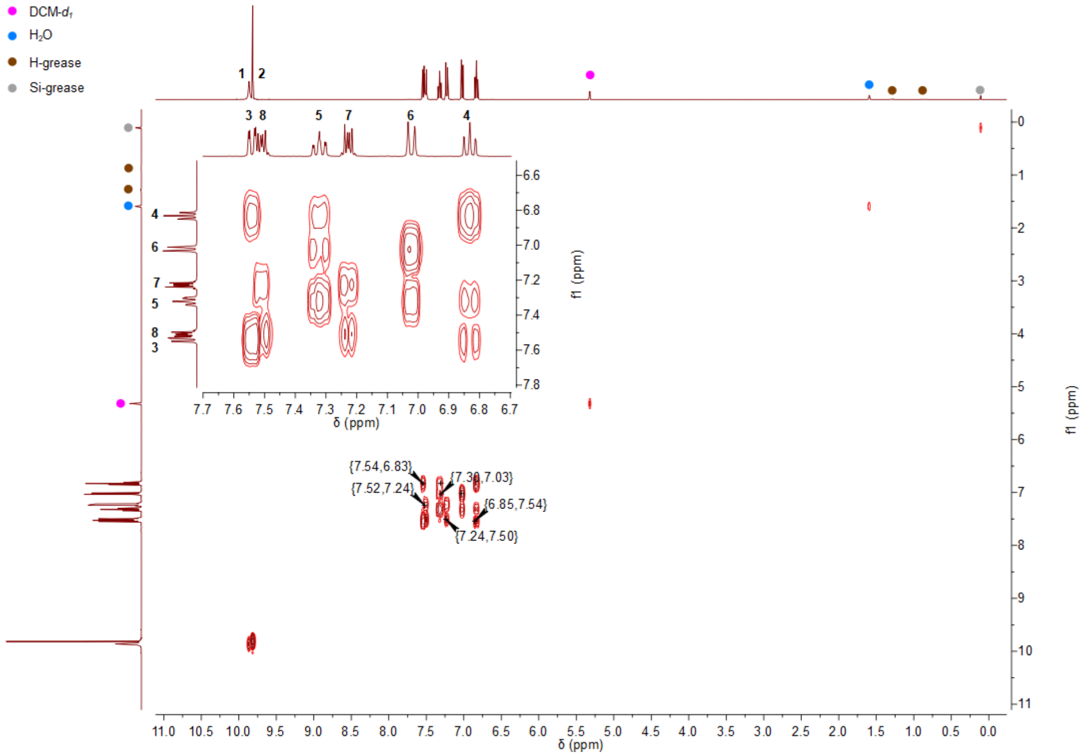

Figure S62. $^1H^1H$-COSY NMR spectrum of **2a** in CD_2Cl_2. Only cross peaks are assigned (400 MHz).

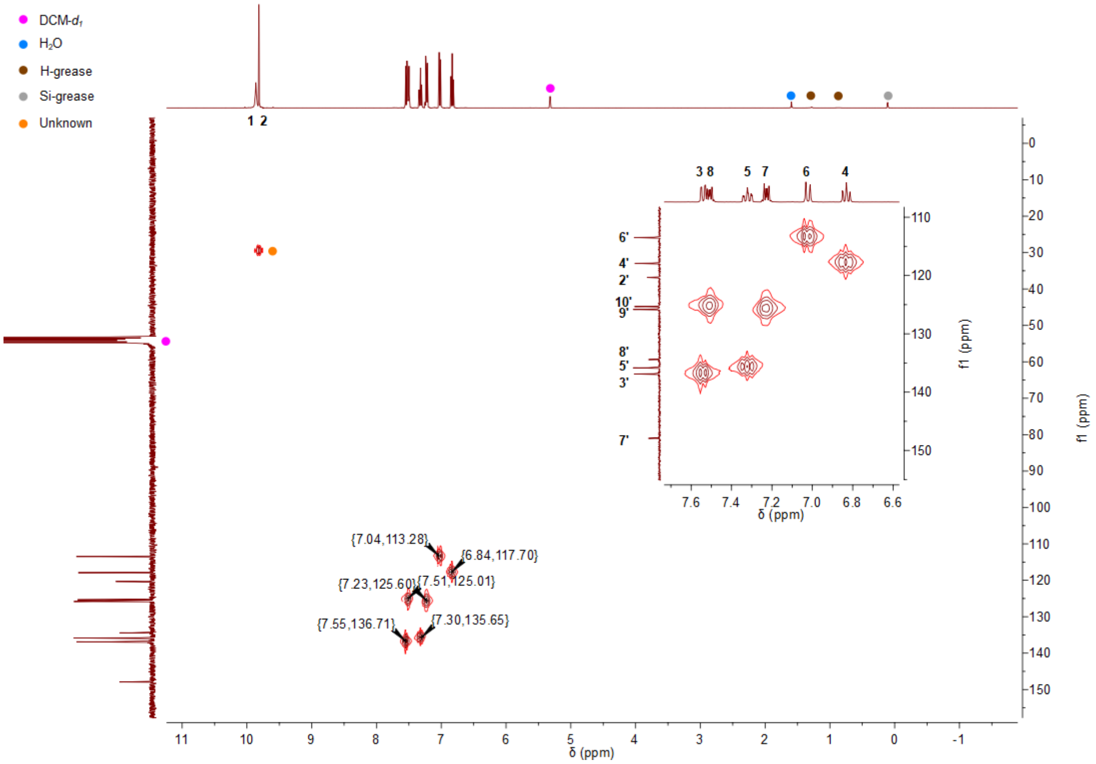

Figure S63. $^{1}H^{13}C$-HSQC NMR spectrum of **2a** in CD_2Cl_2 (400 MHz for ^{1}H, 101 MHz for ^{13}C).

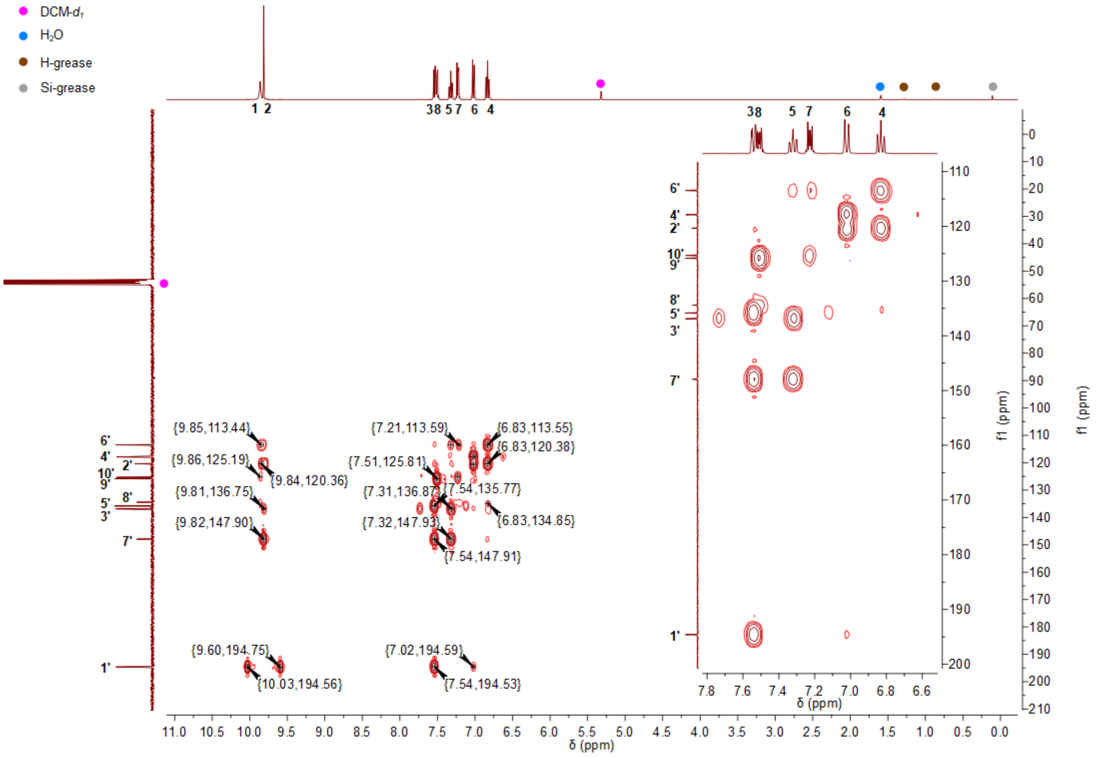

Figure S64. $^{1}H^{13}C$-HMBC NMR spectrum of **2a** in CD_2Cl_2 (400 MHz for ^{1}H, 101 MHz for ^{13}C).

N,N'-(1,2-phenylenediamino)-bis(2-aminoacetophenone) (2b)

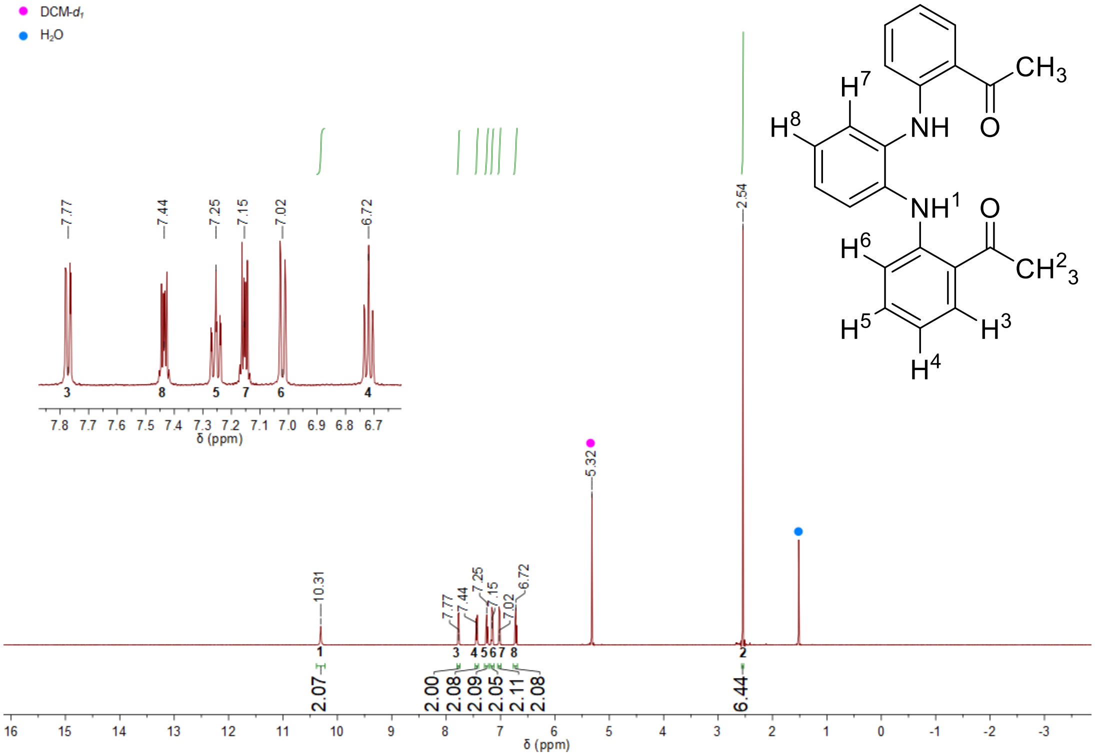

Figure S65. ^{1}H-NMR spectrum of **2b** in CD_2Cl_2 (500 MHz).

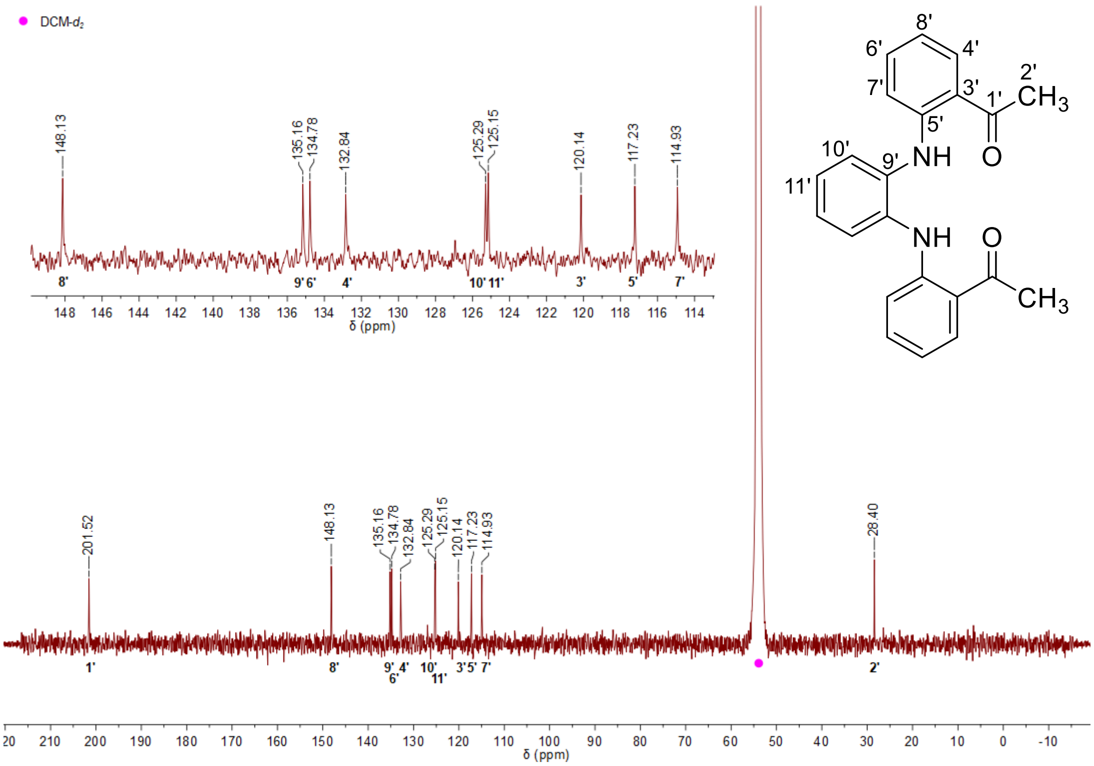

Figure S66. ^{13}C-NMR spectrum of **2b** in CD_2Cl_2 (126 MHz).

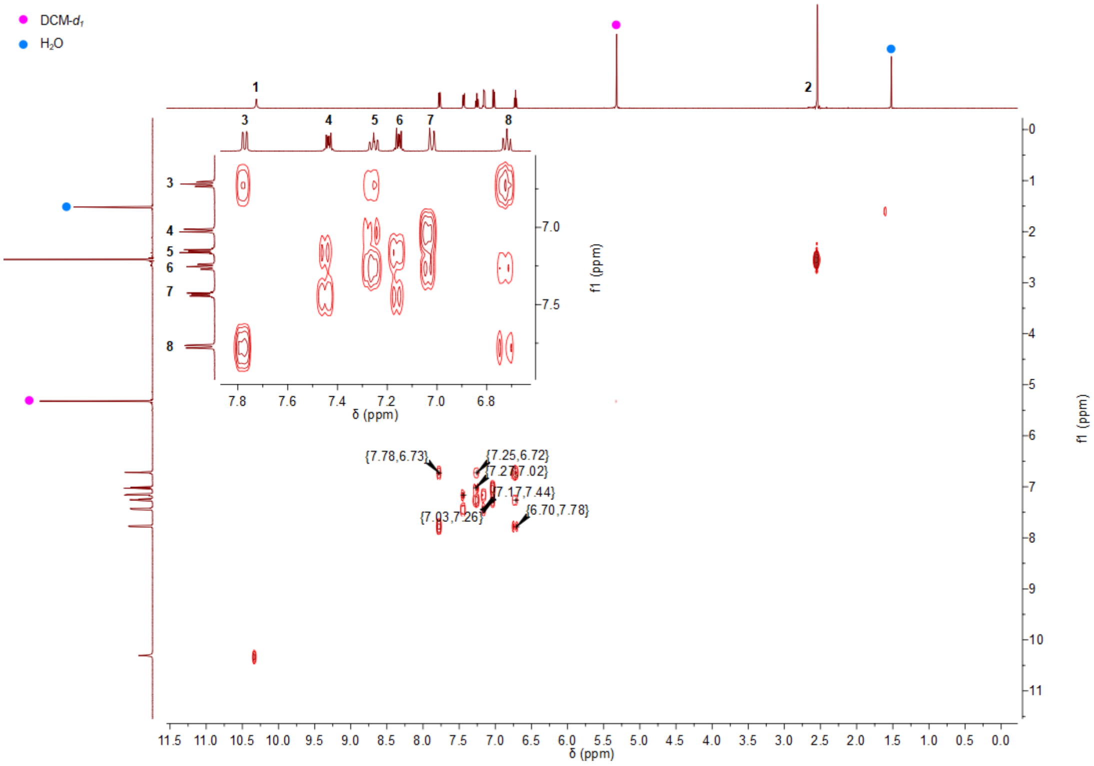

Figure S67. $^1H^1H$-COSY NMR spectrum of **2b** in CD_2Cl_2. Only cross peaks are assigned (400 MHz).

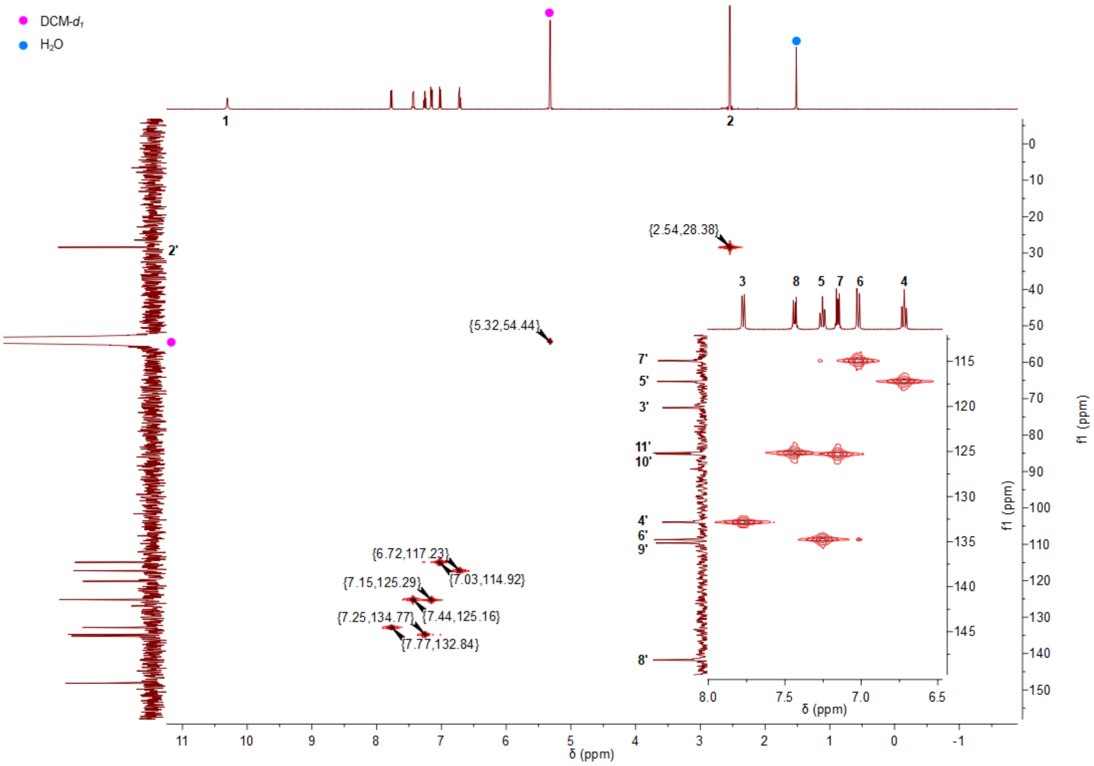

Figure S68. $^1H^{13}C$-HSQC NMR spectrum of **2b** in CD_2Cl_2 (500 MHz for ^1H, 126 MHz for ^{13}C).

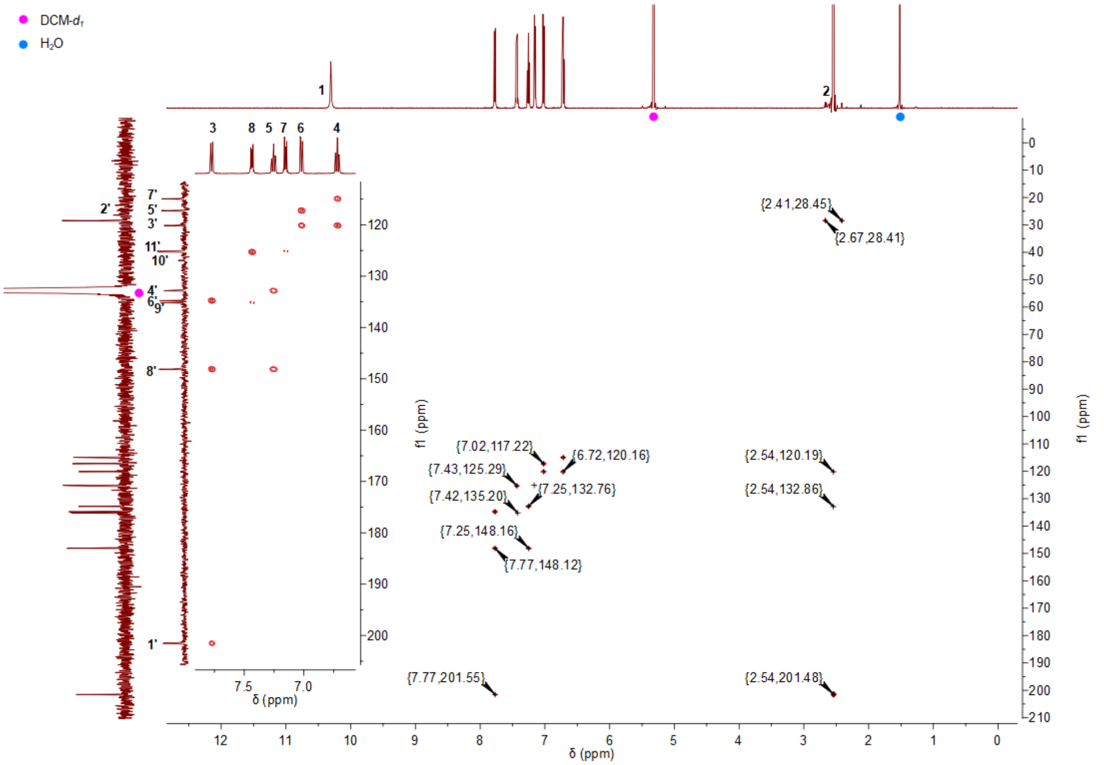

Figure S69. $^{1}H^{13}C$-HMBC NMR spectrum of **2b** in CD_2Cl_2 (500 MHz for ^{1}H, 126 MHz for ^{13}C).

N,N'-(1,2-phenylenediamino)-bis(2-aminobenzophenone) (2c)

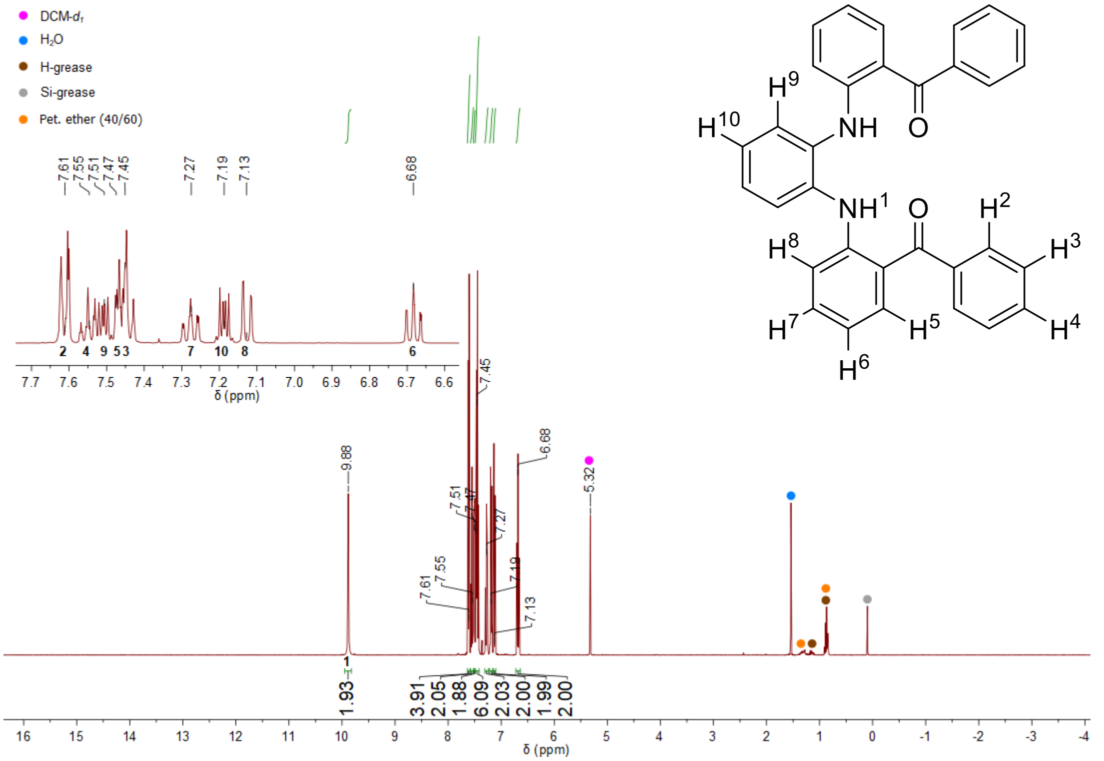

Figure S70. ^{1}H-NMR spectrum of **2c** in CD_2Cl_2 (400 MHz).

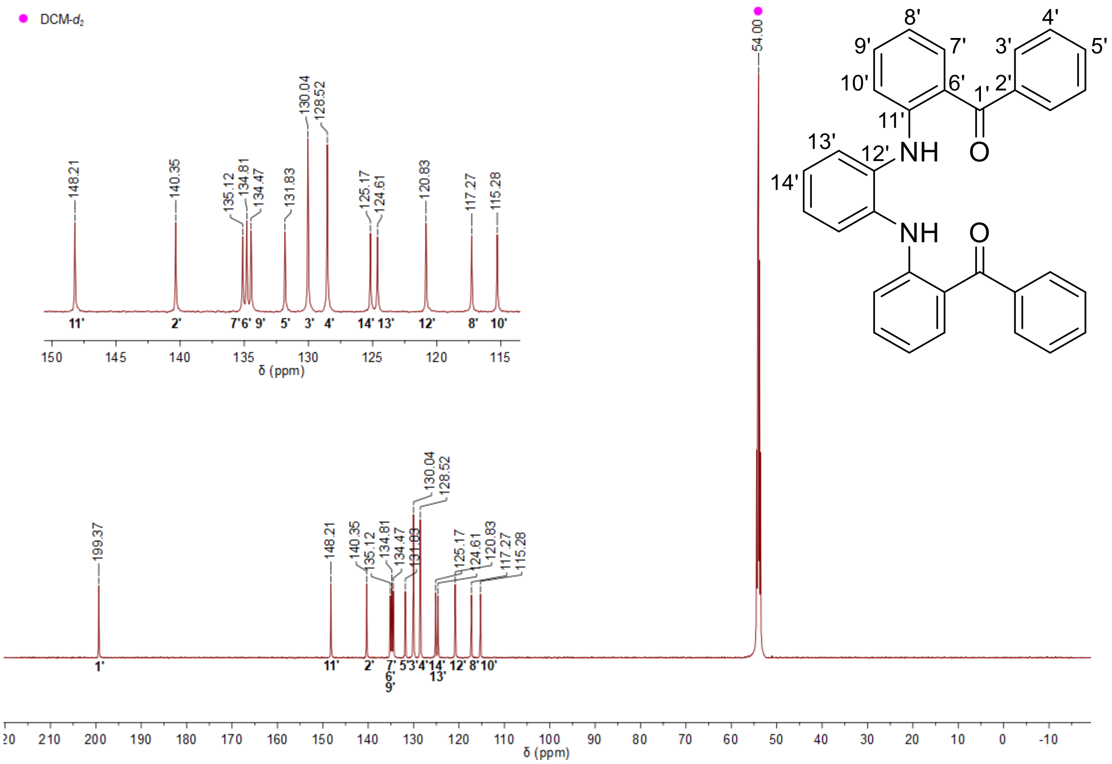

Figure S71. ^{13}C-NMR spectrum of **2c** in CD_2Cl_2 (126 MHz).

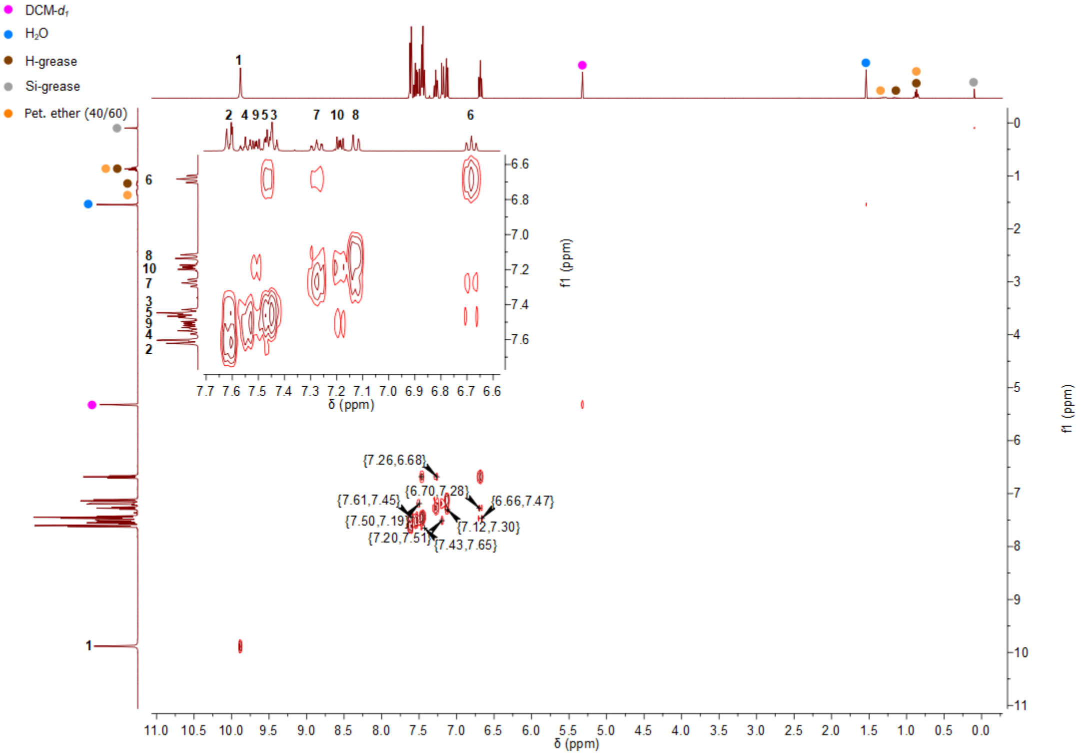

Figure S72. ^{1}H–^{1}H COSY NMR spectrum of **2c** in CD_2Cl_2. Only cross peaks are assigned (400 MHz).

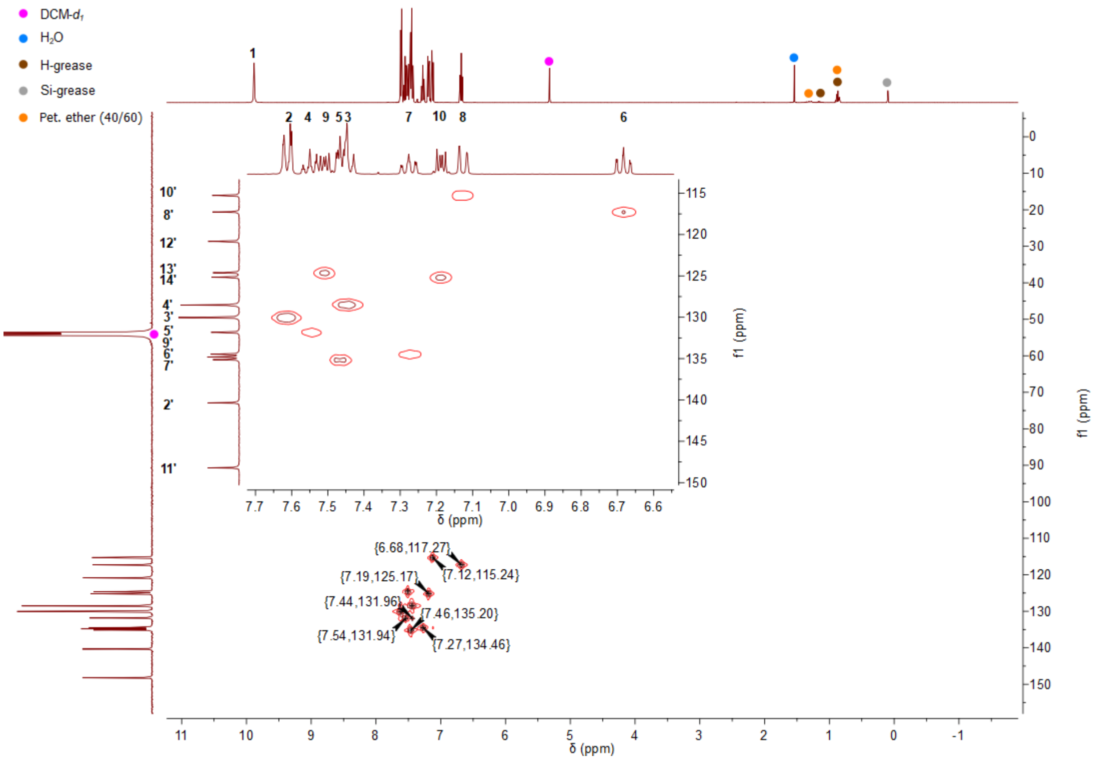

Figure S73. $^{1}H^{13}C$-HSQC NMR spectrum of **2c** in CD_2Cl_2 (500 MHz for ^{1}H, 126 MHz for ^{13}C)

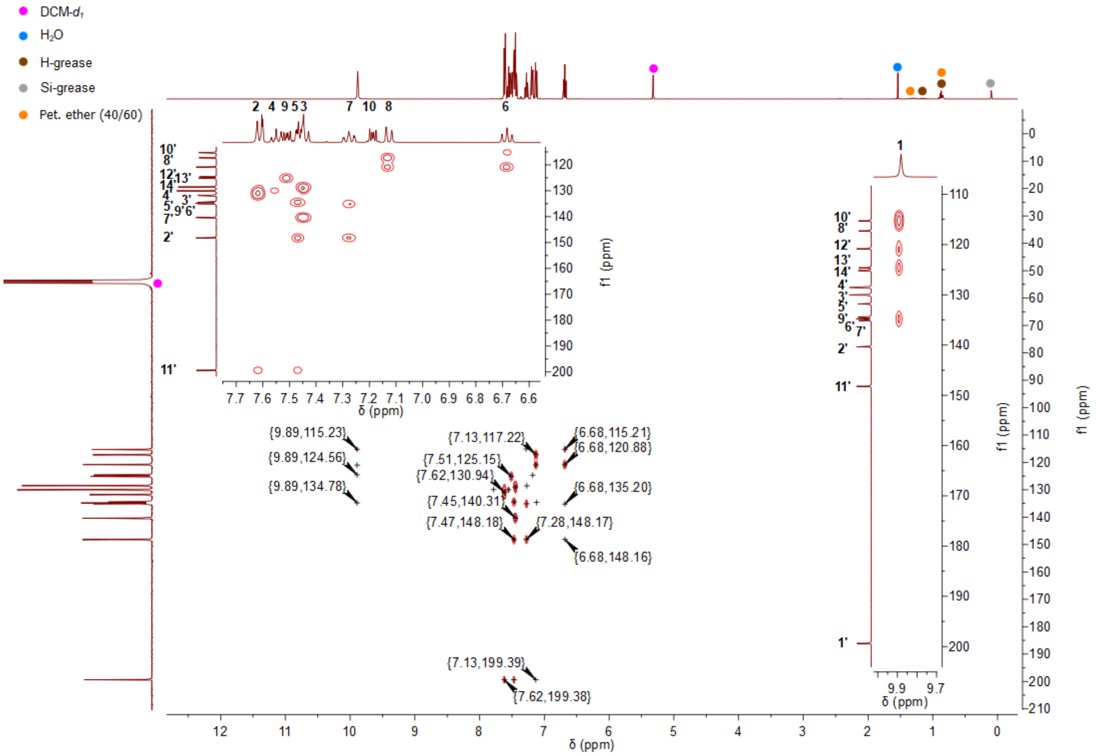

Figure S74. $^{1}H^{13}C$-HMBC NMR spectrum of **2c** in CD_2Cl_2 (500 MHz for ^{1}H, 126 MHz for ^{13}C).

$H_2(H_2PhenTAA)$ (3a)

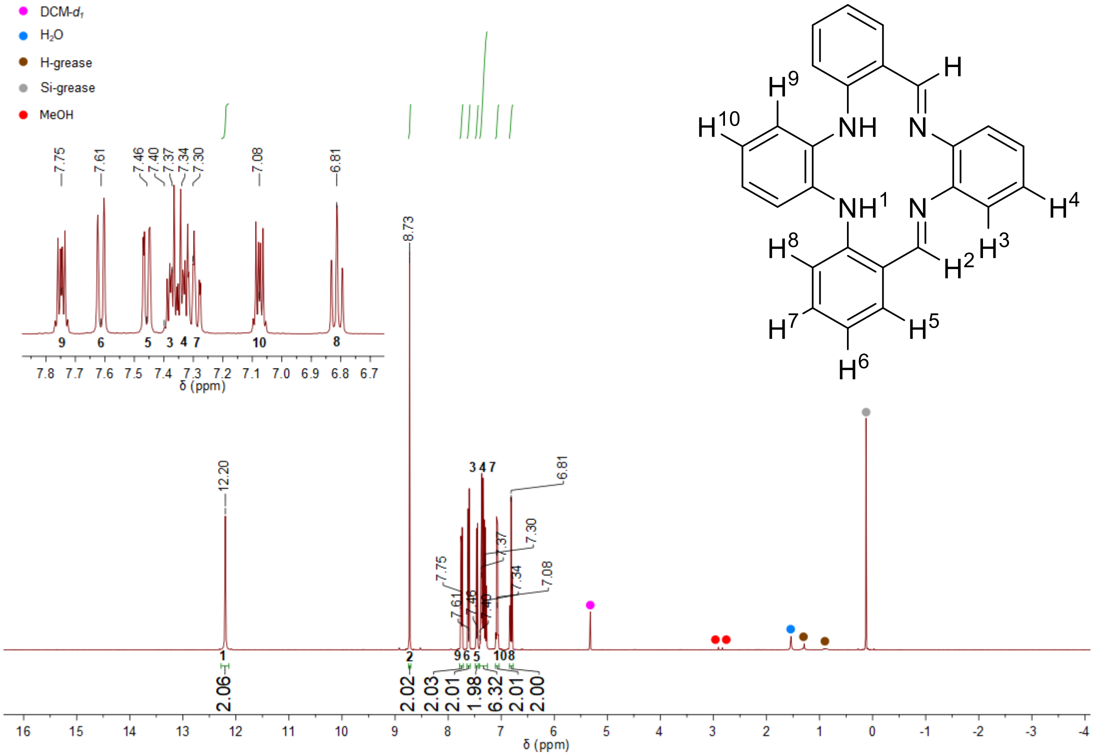

Figure S75. ^{1}H-NMR spectrum of **3a** in CD_2Cl_2 (400 MHz).

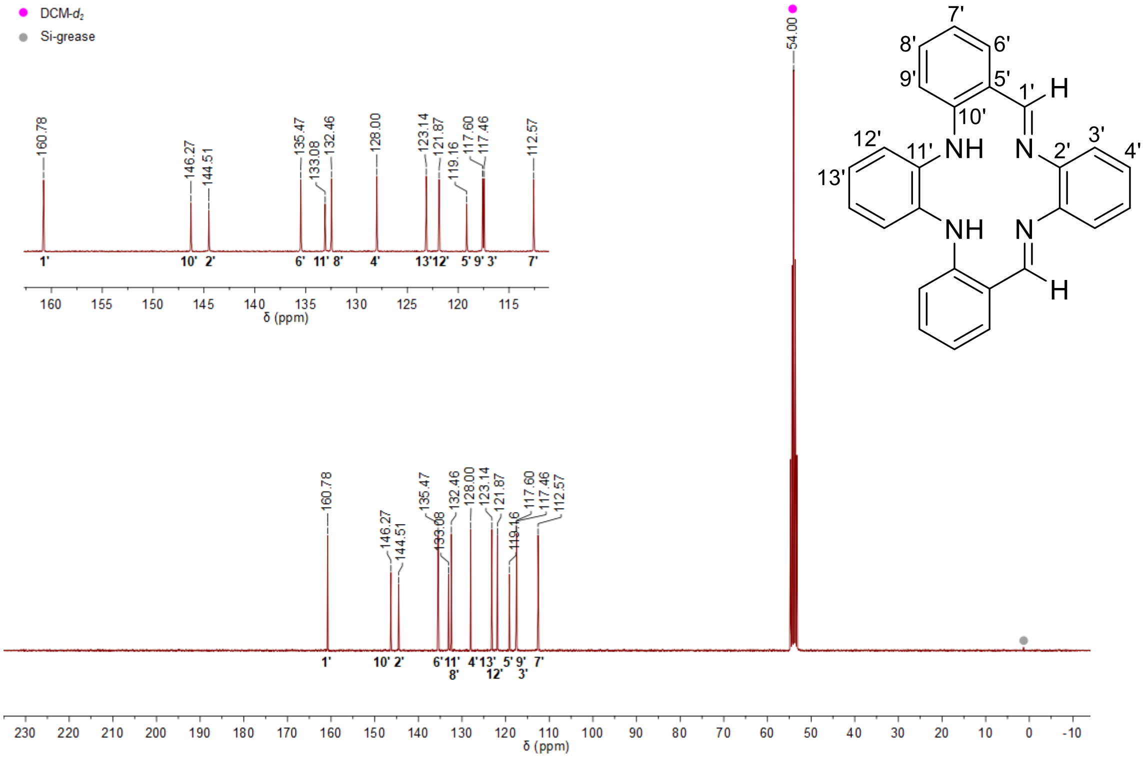

Figure S76. ^{13}C-NMR spectrum of **3a** in CD_2Cl_2 (75 MHz).

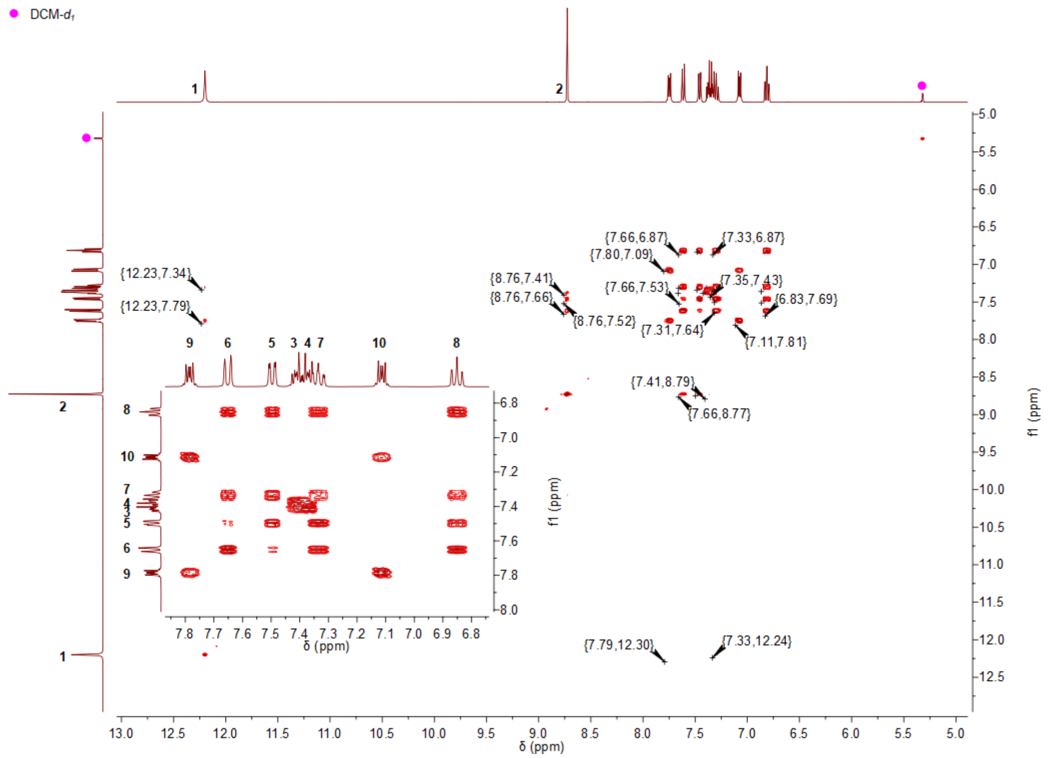

Figure S77. $^1H^1H$-COSY NMR spectrum of **3a** in CD_2Cl_2 (400 MHz).

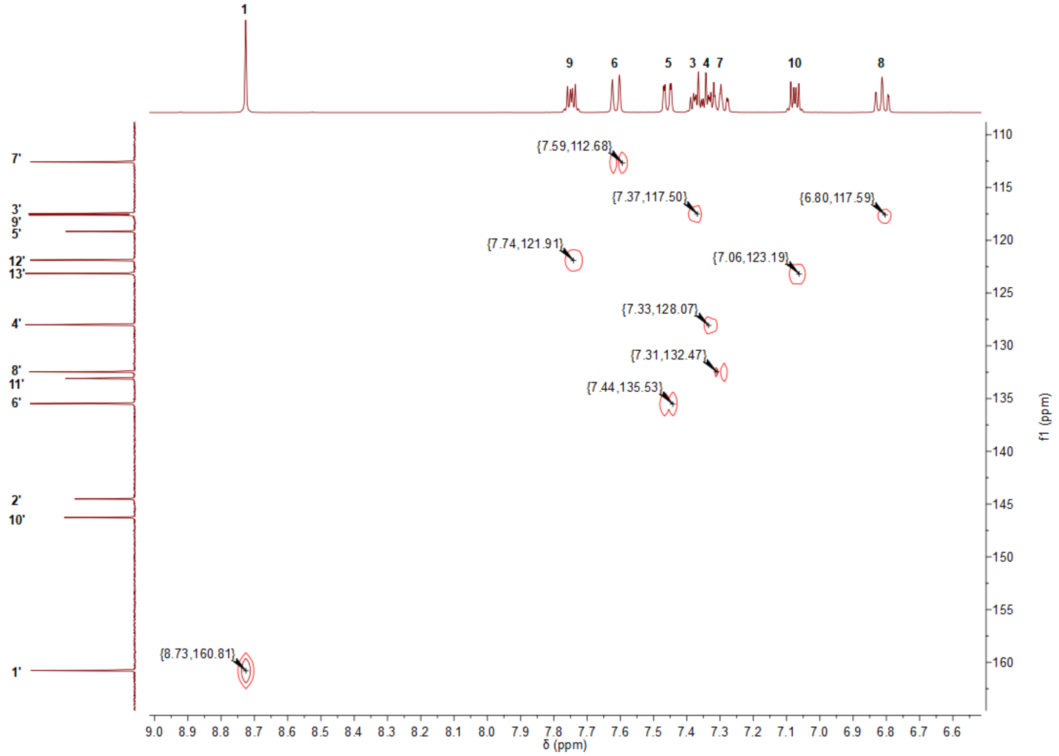

Figure S78. $^1H^{13}C$-HSQC NMR spectrum of **3a** in CD_2Cl_2 (400 MHz for ^1H, 101 MHz for ^{13}C)

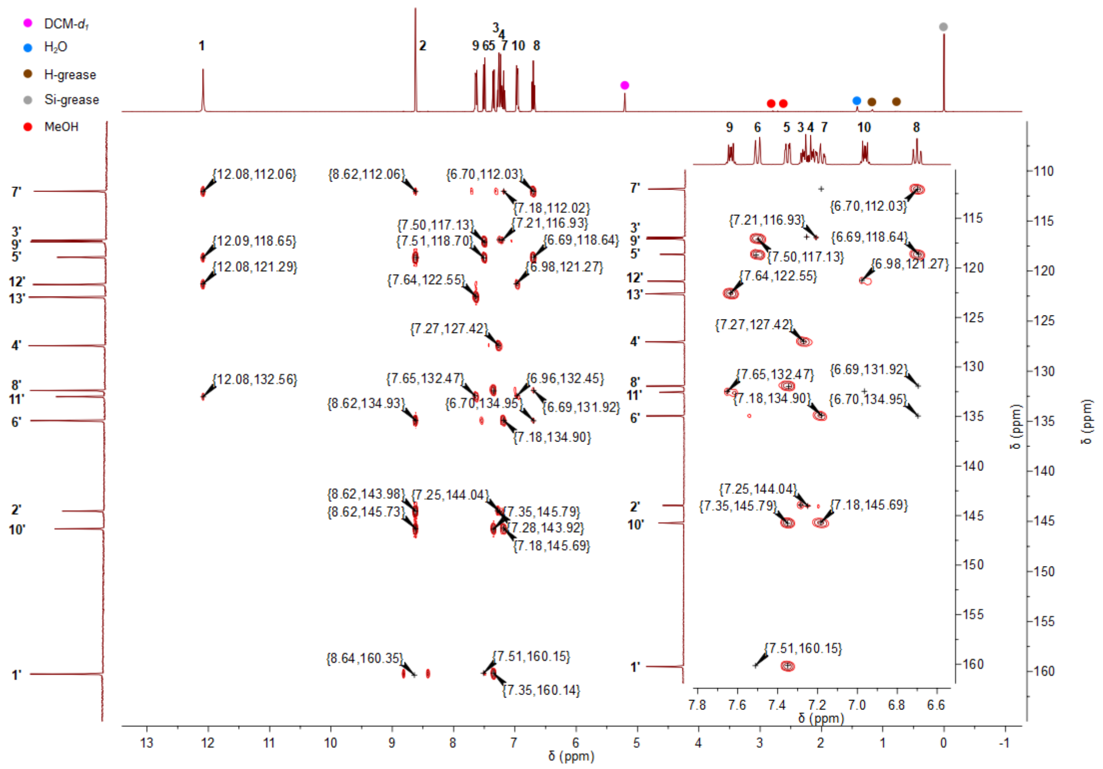

Figure S79. $^{1}H^{13}C$-HMBC NMR spectrum of **3a** in CD_2Cl_2 (400 MHz for ^{1}H, 101 MHz for ^{13}C).

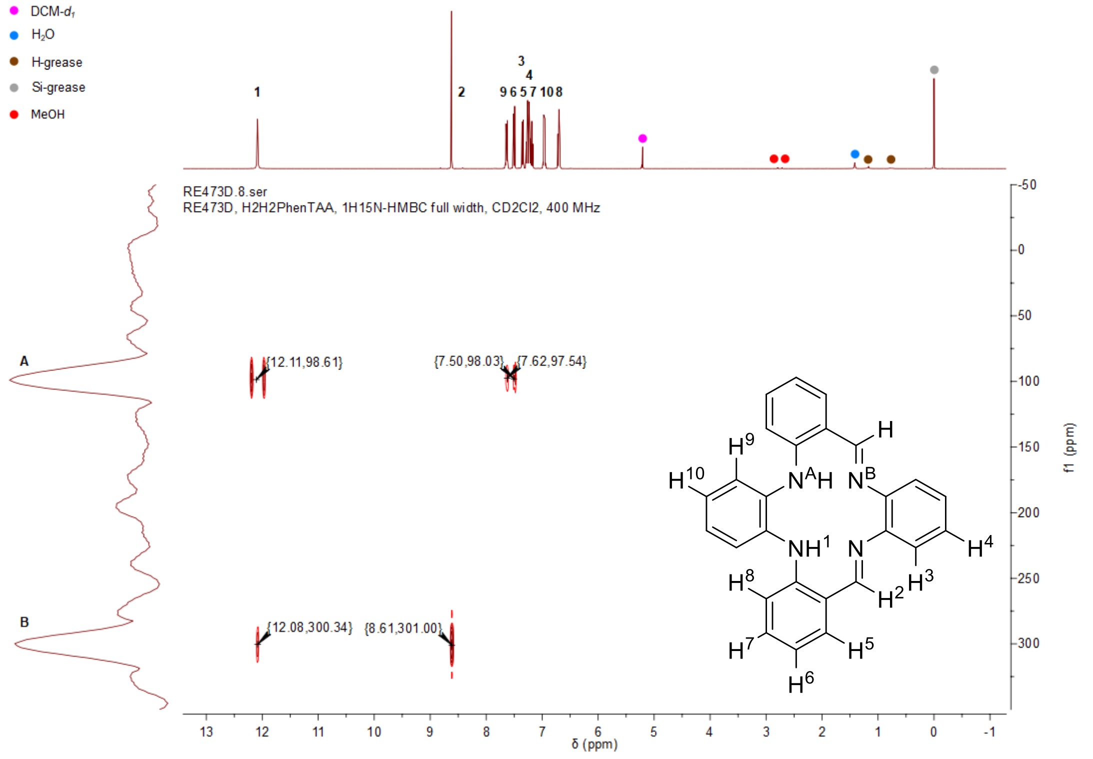

Figure S80. $^{1}H^{15}N$-HMBC NMR spectrum of **3a** in CD_2Cl_2 (400 MHz for ^{1}H, 41 MHz for ^{15}N).

$H_2(Me_2PhenTAA)$ (3b)

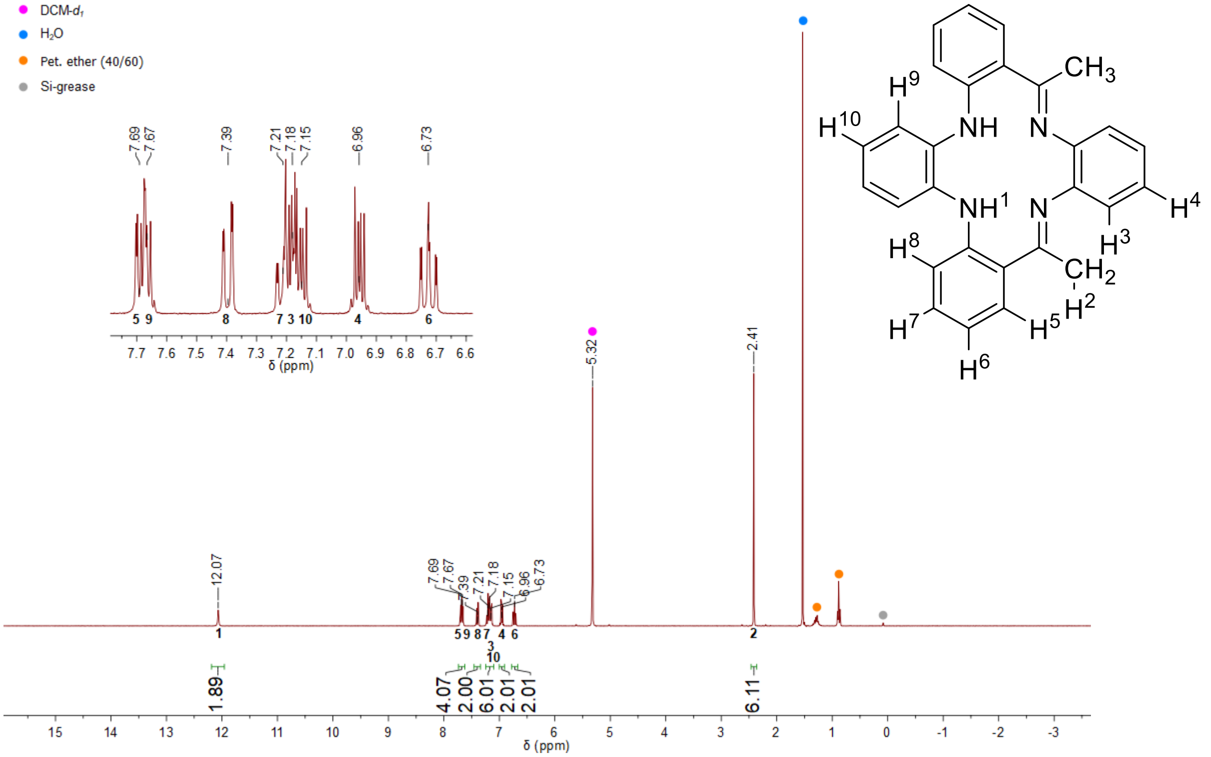

Figure S81. ^{1}H-NMR spectrum of **3b** in CD_2Cl_2 (300 MHz).

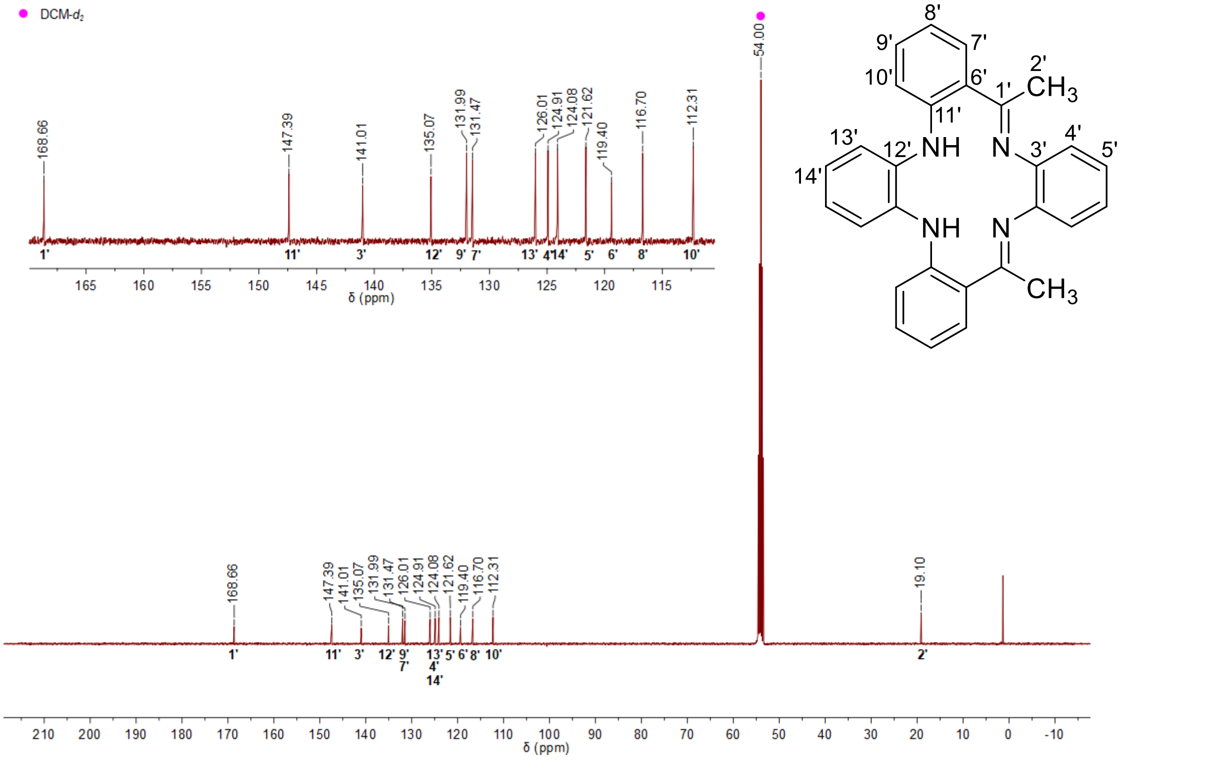

Figure S82. ^{13}C-NMR spectrum of **3b** in CD_2Cl_2 (101 MHz).

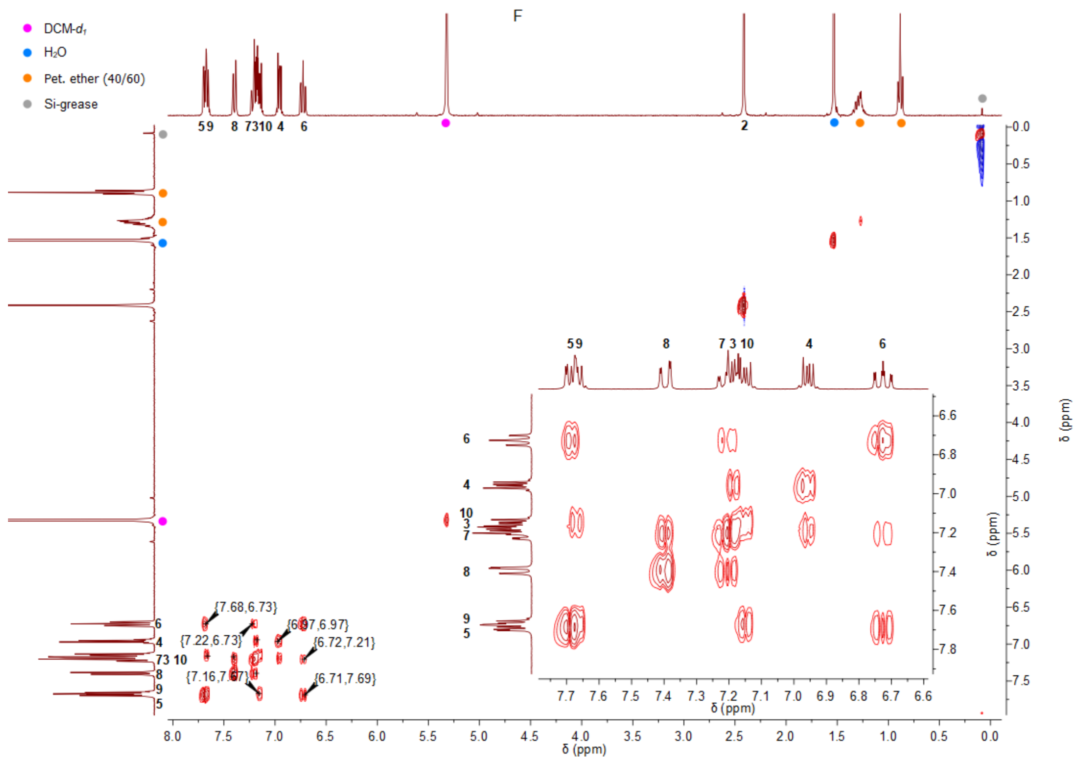

Figure S83. $^1H^1H$-COSY NMR spectrum of **3b** in CD_2Cl_2. Only cross peaks are assigned (400 MHz).

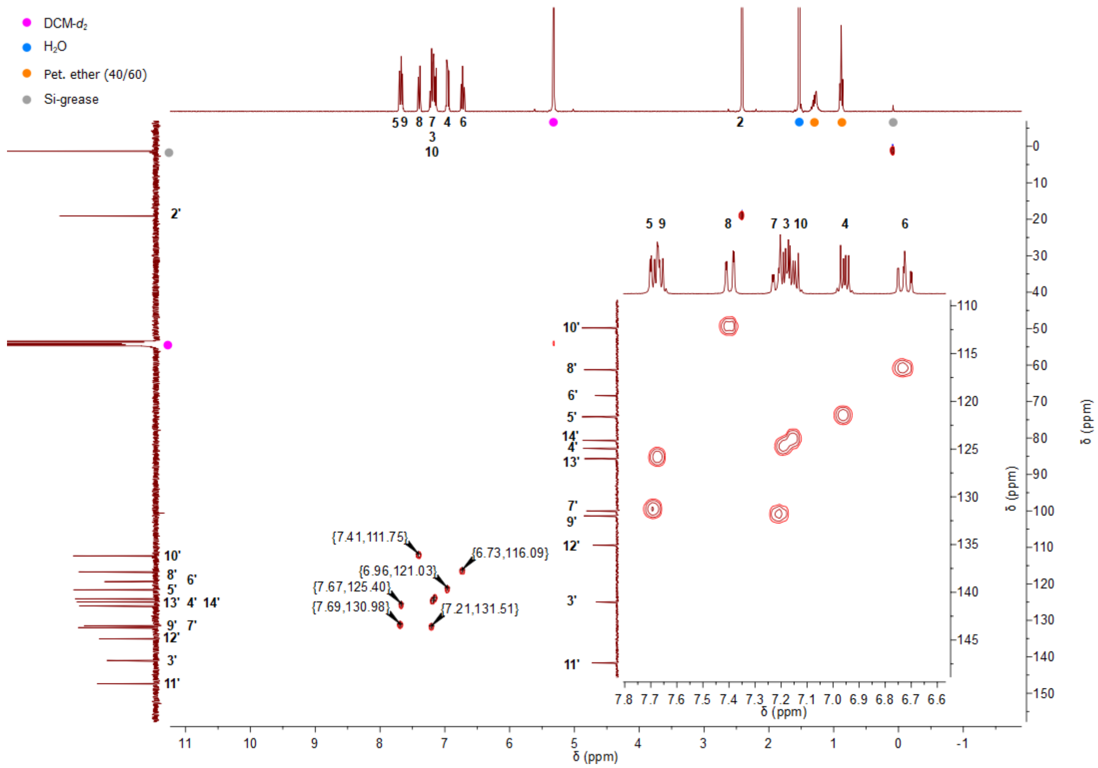

Figure S84. $^1H^{13}C$-HSQC NMR spectrum of **3b** in CD_2Cl_2 (400 MHz for ^1H, 101 MHz for ^{13}C).

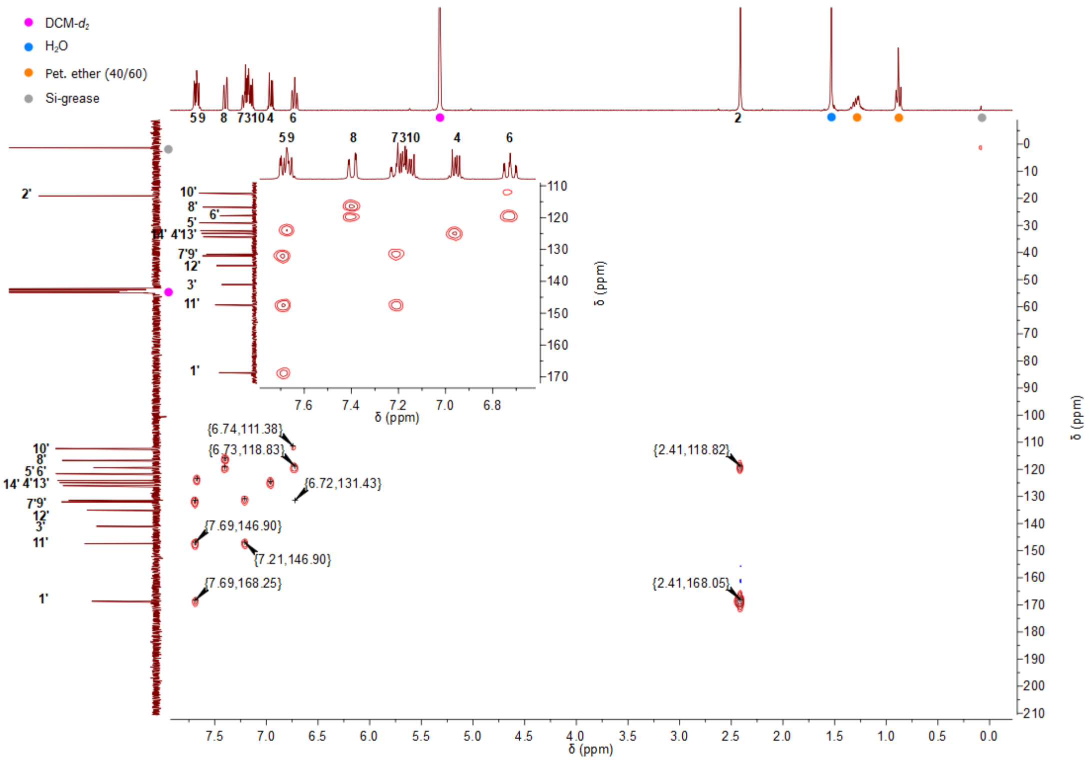

Figure S85. $^{1}H^{13}C$-HMBC NMR spectrum of **3b** in CD_2Cl_2 (400 MHz for ^{1}H, 101 MHz for ^{13}C).

[Ni(H2PhenTAA)] (4a)

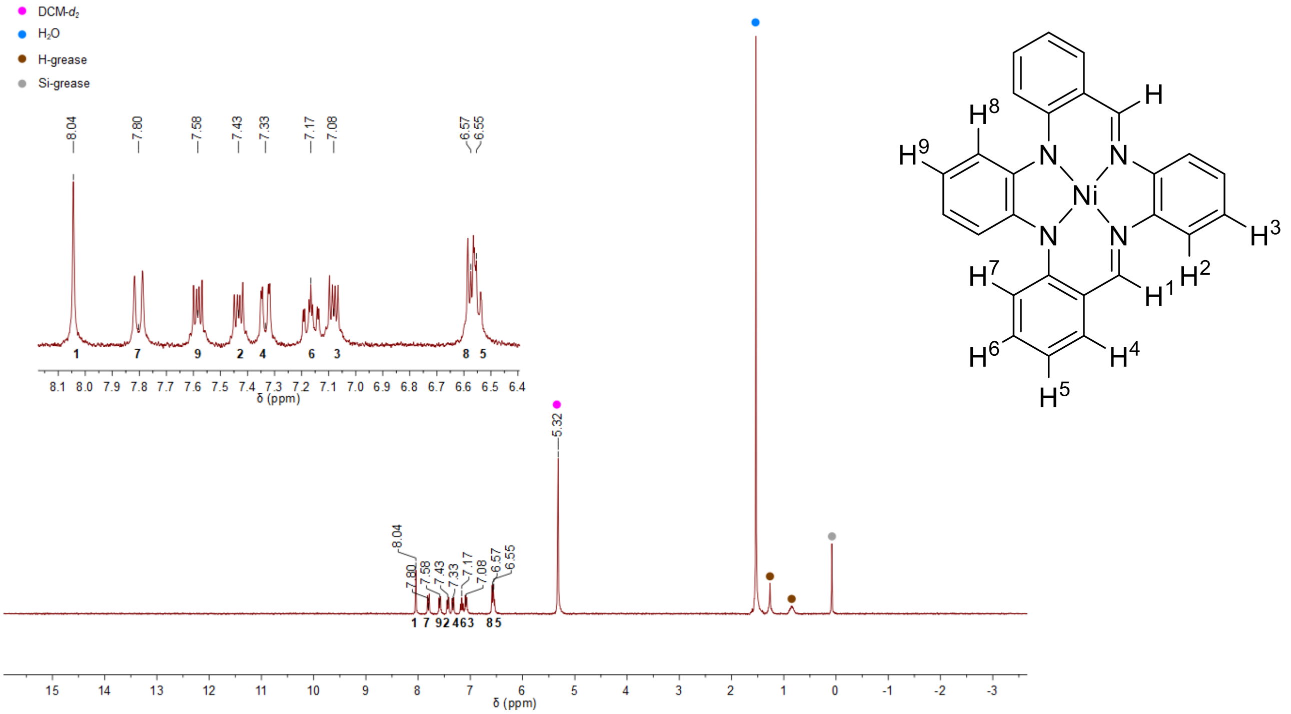

Figure S86. ^{1}H-NMR spectrum of **4a** in CD_2Cl_2 (300 MHz).

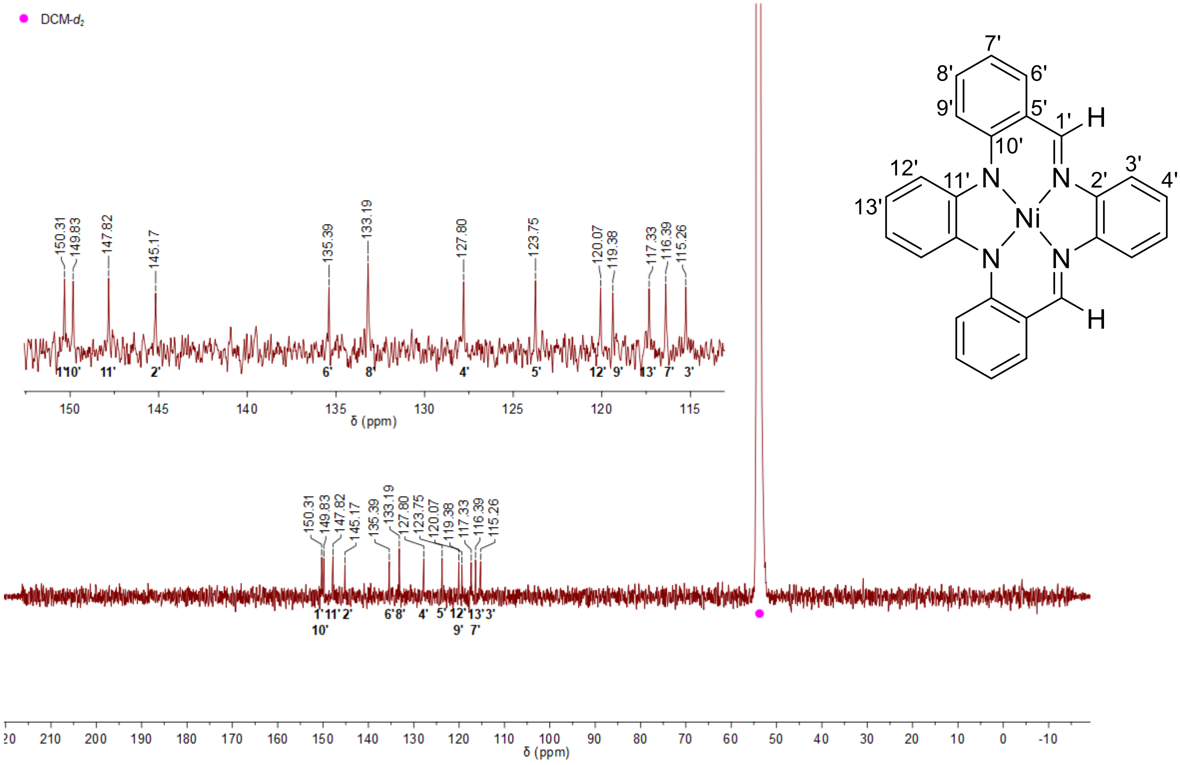

Figure S87. ^{13}C-NMR spectrum of **4a** in CD_2Cl_2 (126 MHz).

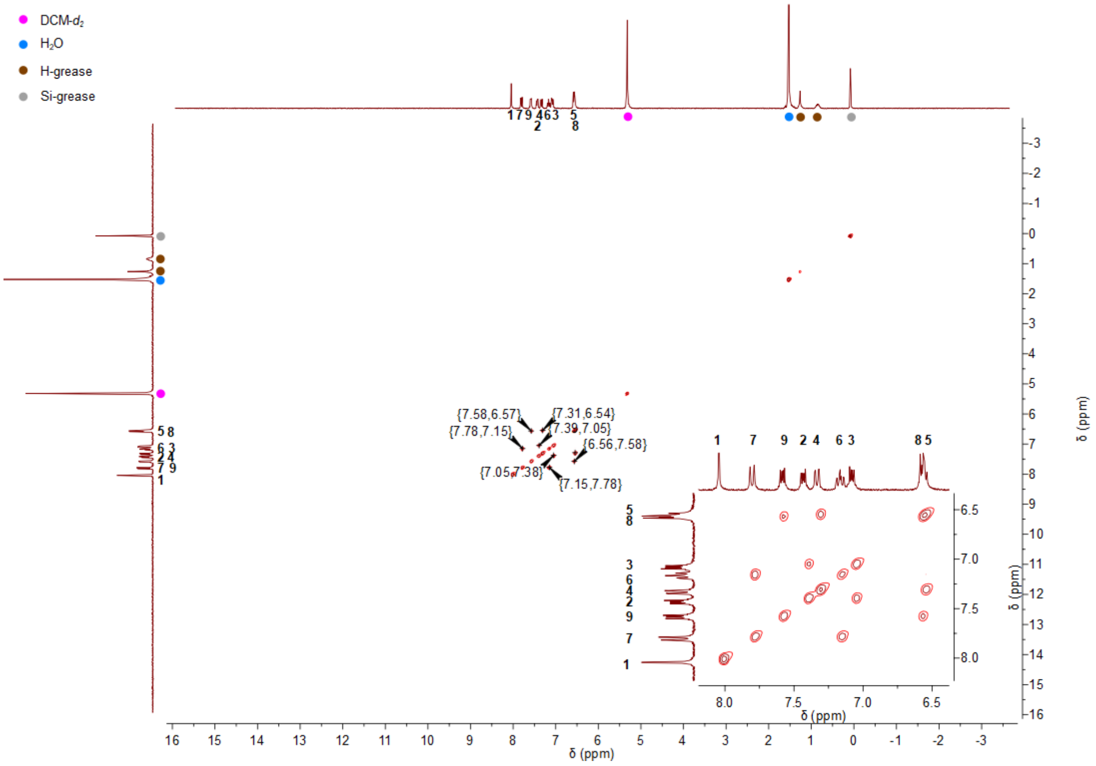

Figure S88. $^1H^1H$-COSY NMR spectrum of **4a** in CD_2Cl_2. Only cross peaks are assigned (500 MHz).

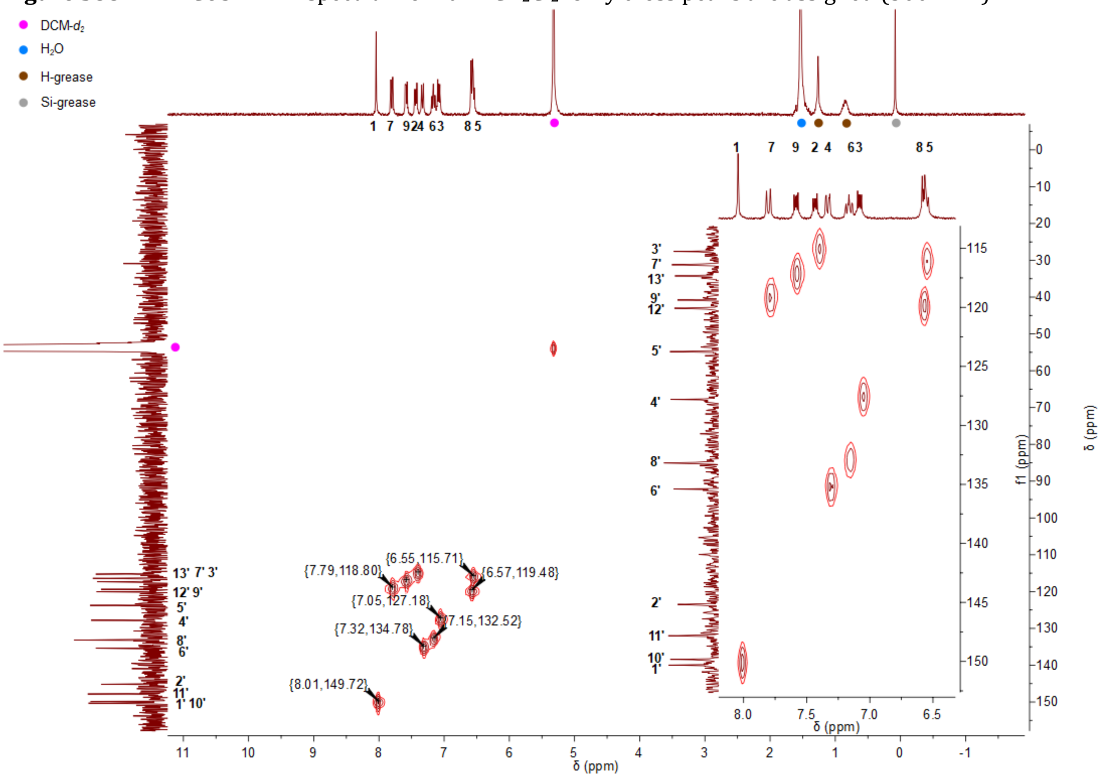

Figure S89. $^1H^{13}C$-HSQC NMR spectrum of **4a** in CD_2Cl_2 (500 MHz for ^1H, 126 MHz for ^{13}C).

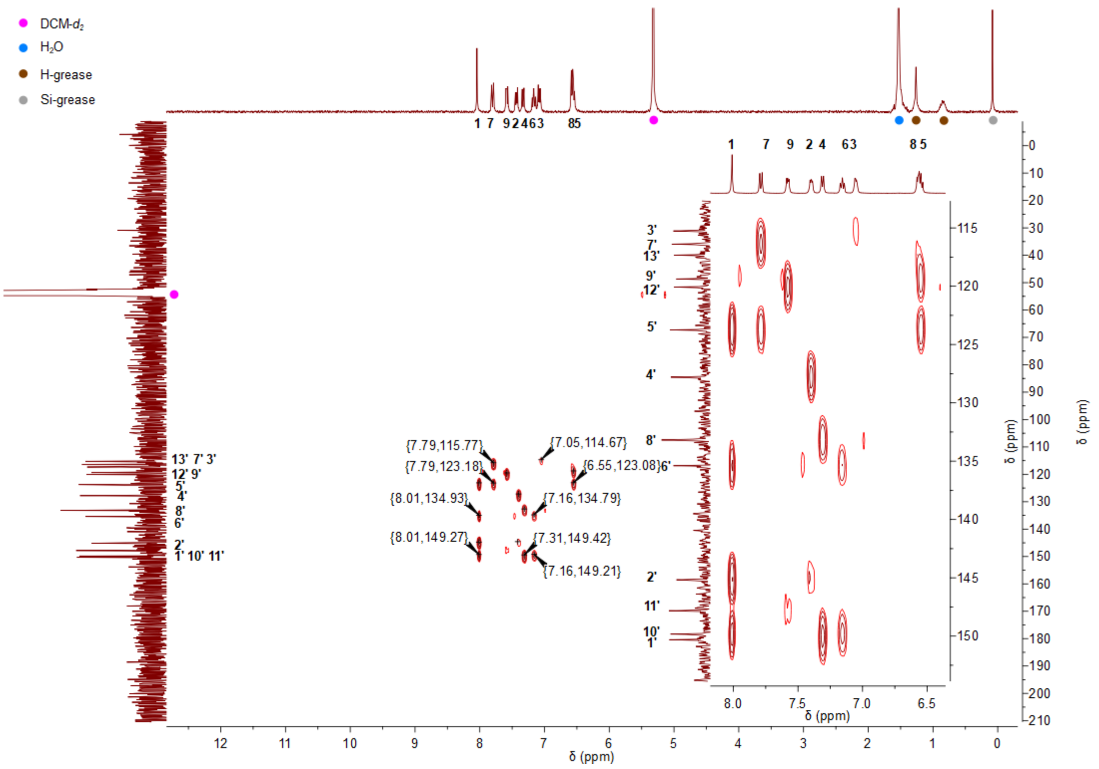

Figure S90. $^{1}H^{13}C$-HMBC NMR spectrum of **4a** in CD_2Cl_2 (500 MHz for ^{1}H, 126 MHz for ^{13}C).

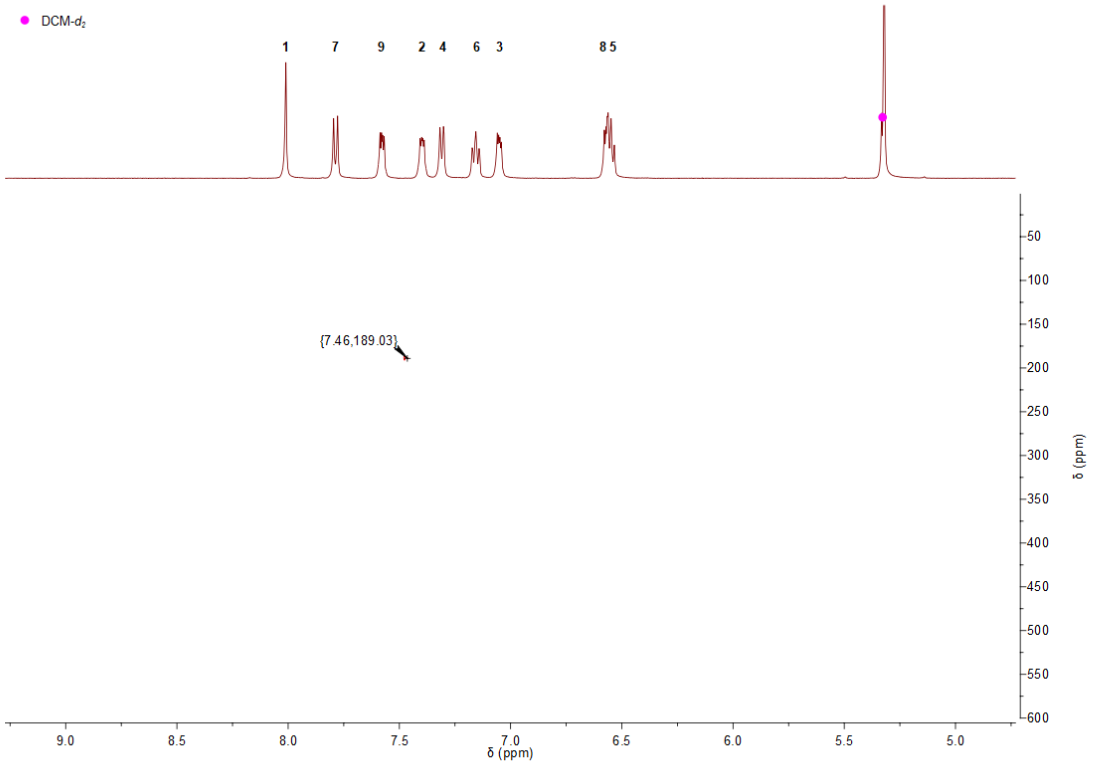

Figure S91. $^{1}H^{15}N$-HMBC NMR spectrum of **4a** in CD_2Cl_2 (500 MHz for ^{1}H, 51 MHz for ^{15}N).

[Ni(Me2PhenTAA)] (4b)

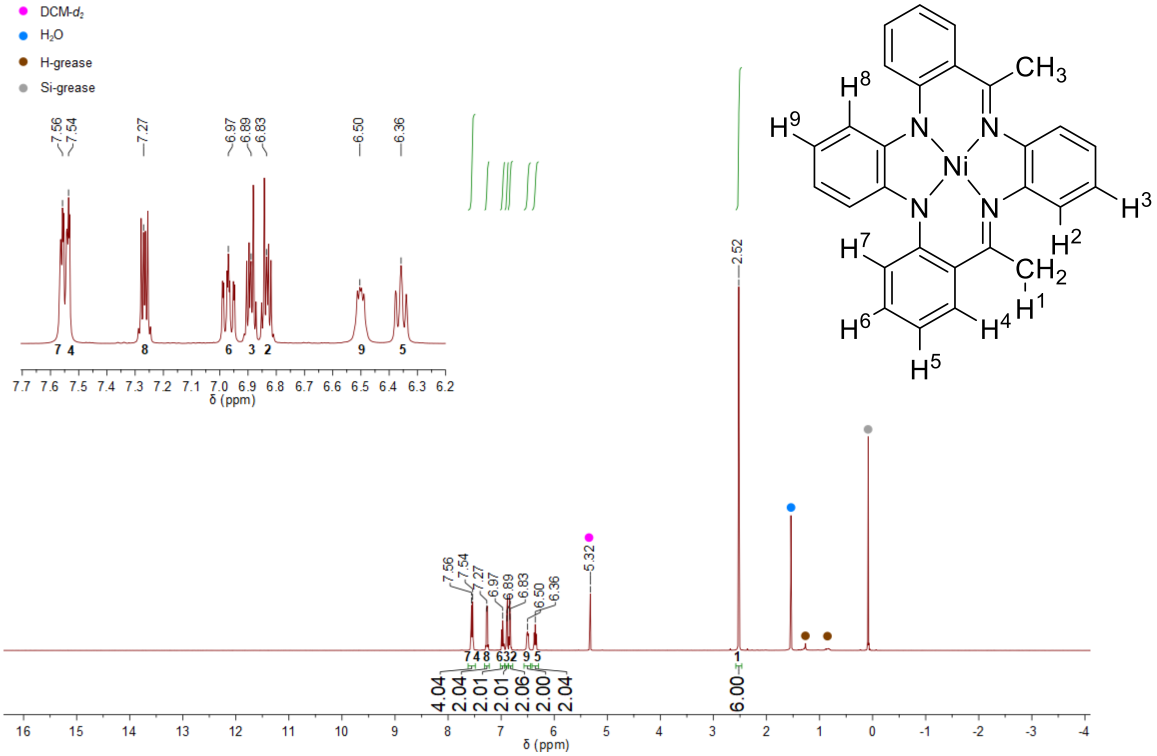

Figure S92. ^{1}H-NMR spectrum of **4b** in CD_2Cl_2 (400 MHz).

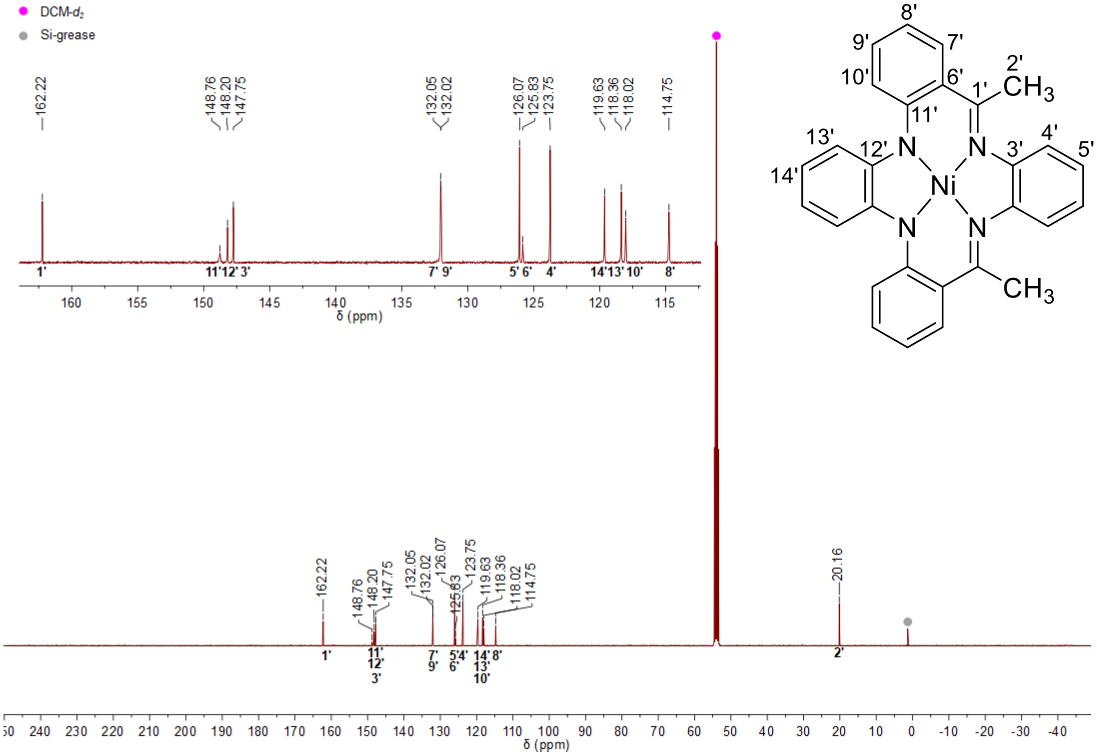

Figure S93. ^{13}C-NMR spectrum of **4b** in CD_2Cl_2 (101 MHz).

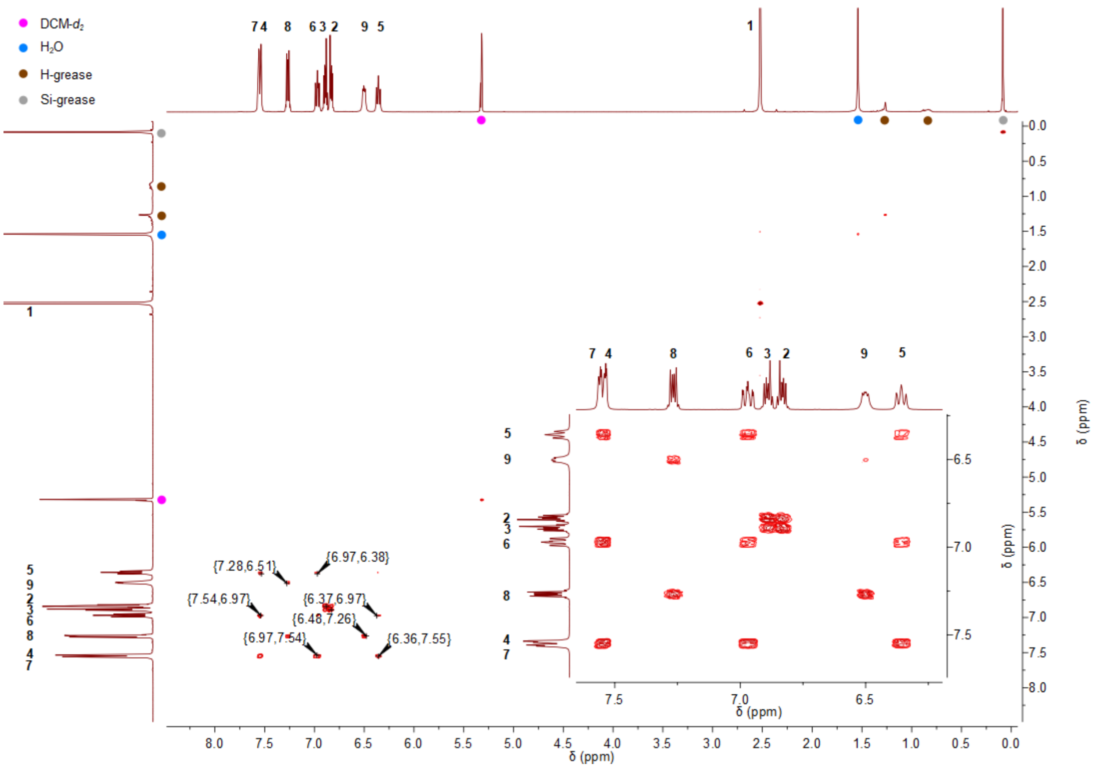

Figure S94. $^1H^1H$-COSY NMR spectrum of **4b** in CD_2Cl_2 (400 MHz).

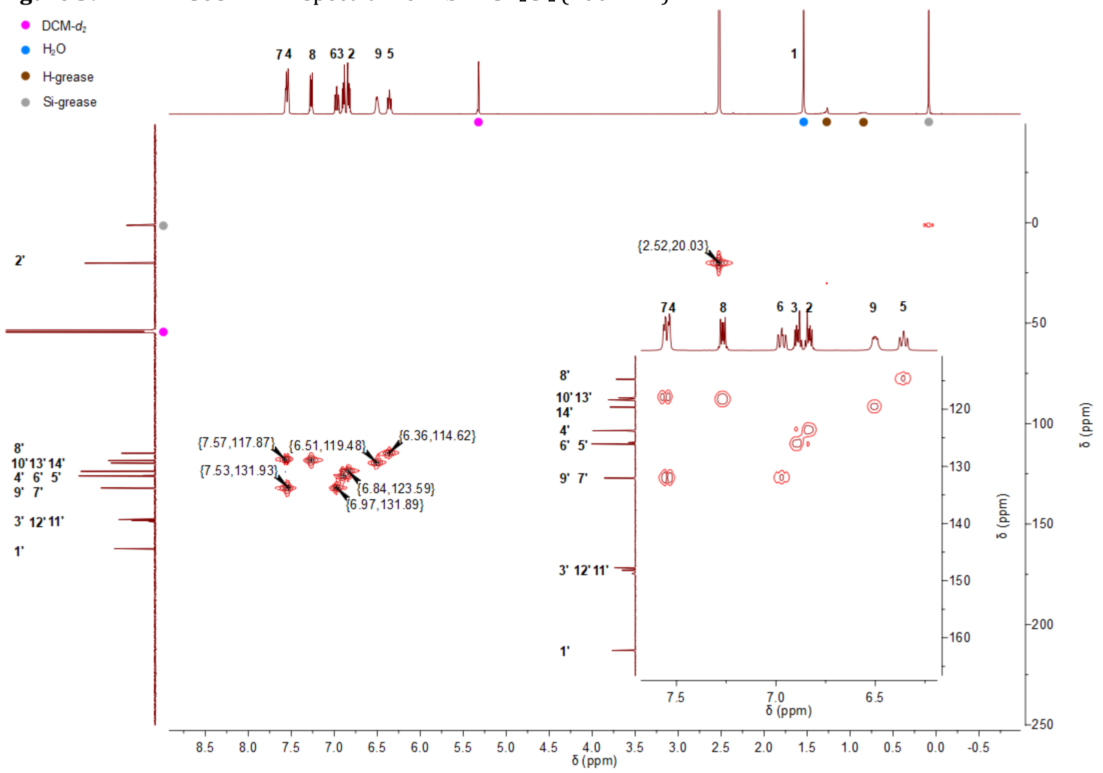

Figure S95. $^1H^{13}C$-HSQC NMR spectrum of **4b** in CD_2Cl_2 (400 MHz for ^1H, 101 MHz for ^{13}C).

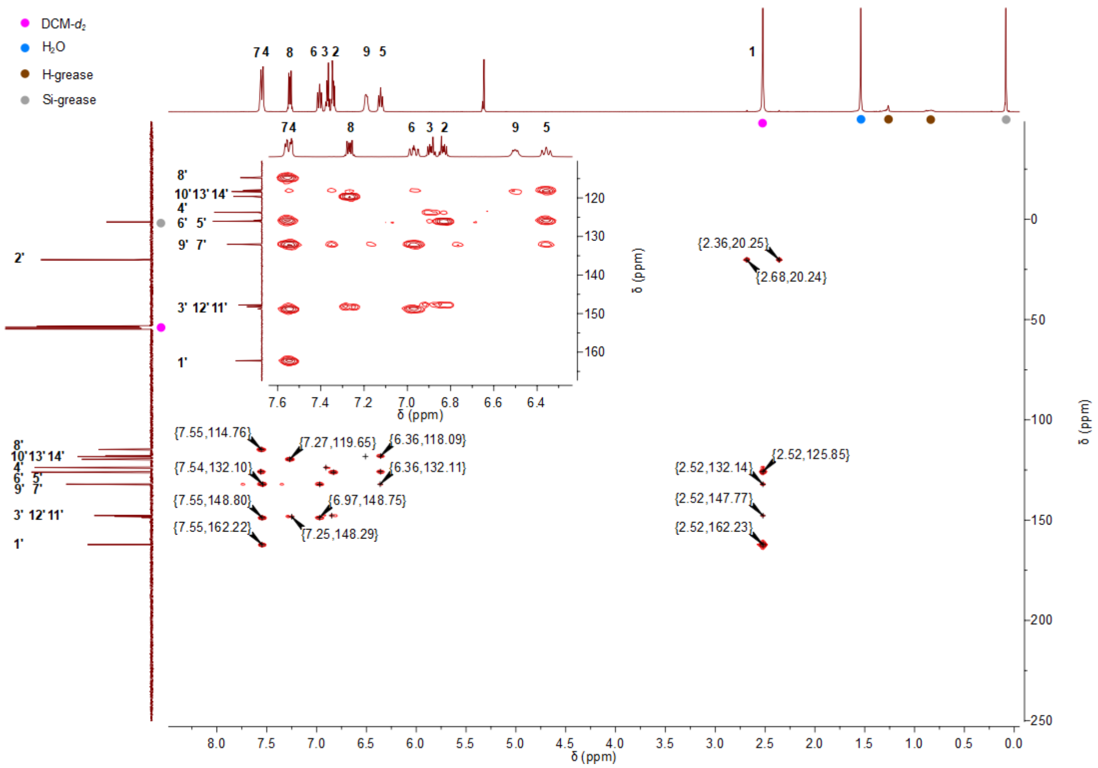

Figure S96. $^{1}H^{13}C$-HMBC NMR spectrum of **4b** in CD_2Cl_2 (400 MHz for ^{1}H, 101 MHz for ^{13}C).

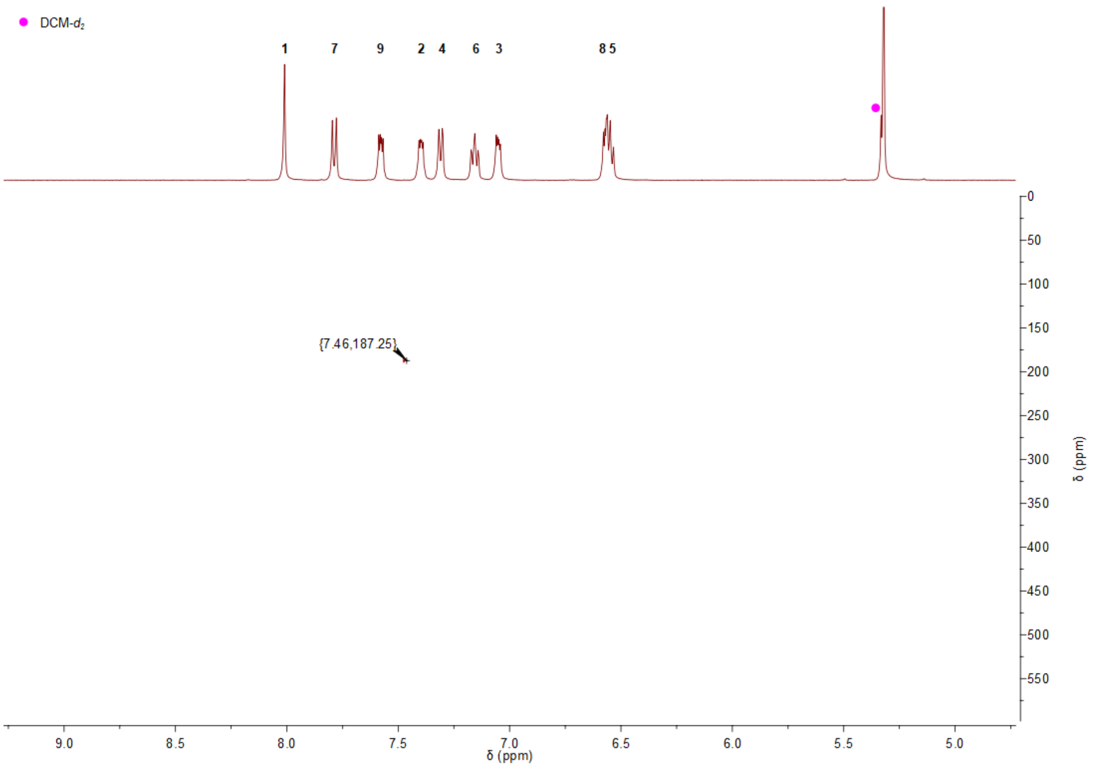

Figure S97. $^{1}H^{15}N$-HMBC NMR spectrum of **4b** in CD_2Cl_2 (400 MHz for ^{1}H, 41 MHz for ^{15}N).

[Ni(Ph2PhenTAA)] (4c)

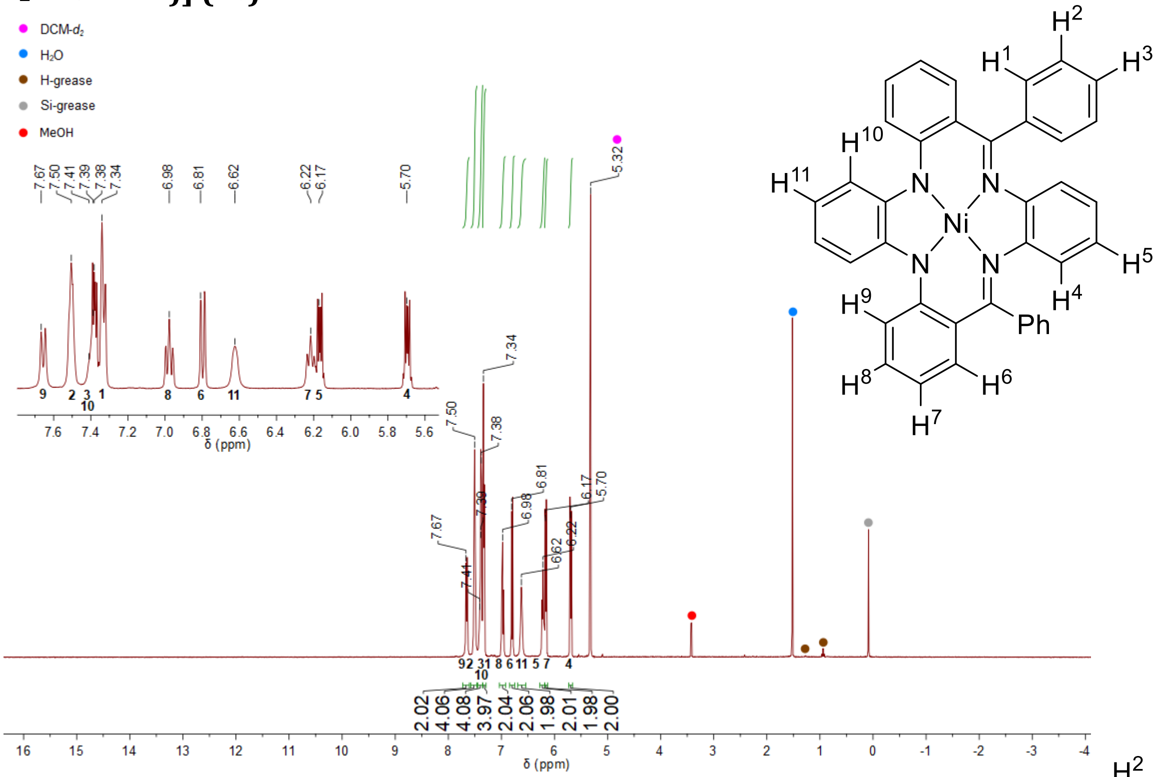

Figure S98. ^{1}H-NMR spectrum of **4c** in CD_2Cl_2 (400 MHz).

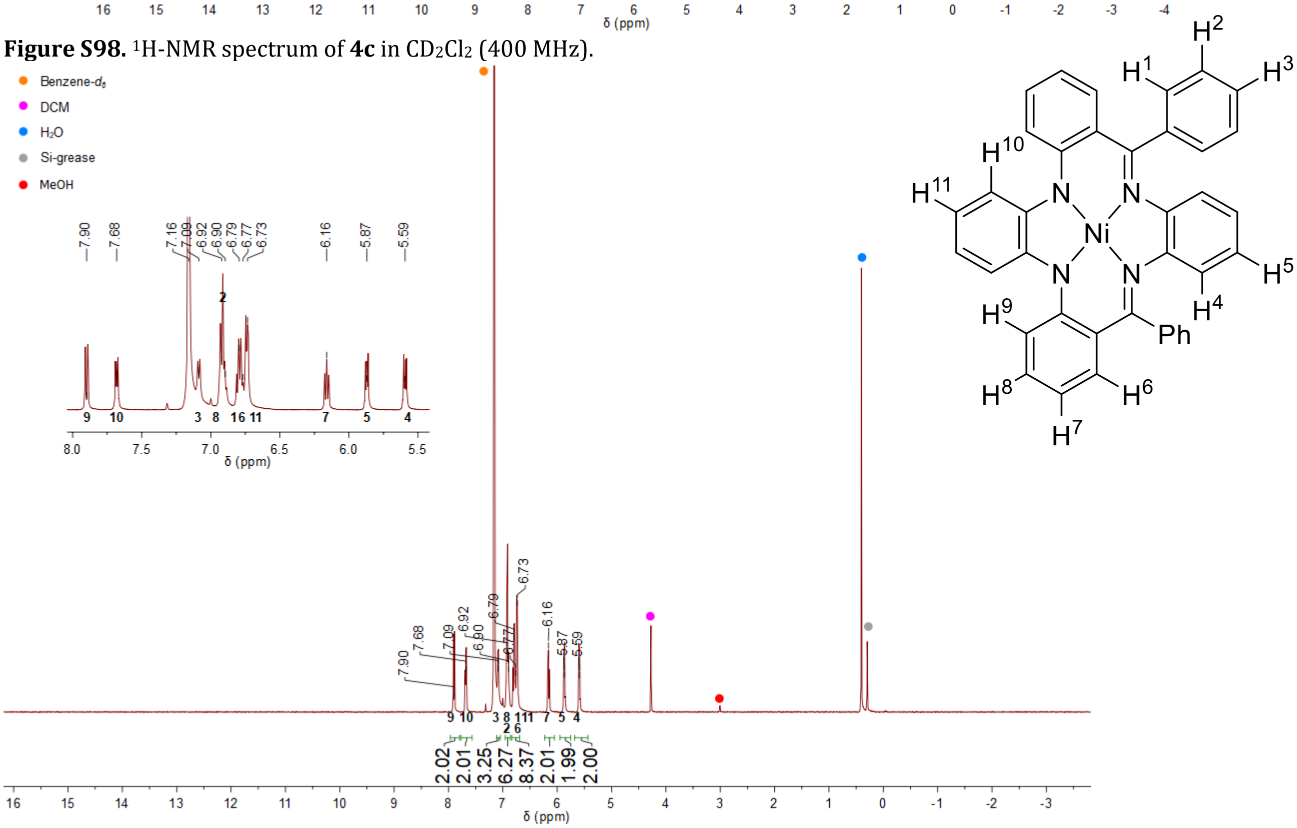

Figure S99. ^{1}H-NMR spectrum of **4c** in C_6D_6 (500 MHz).

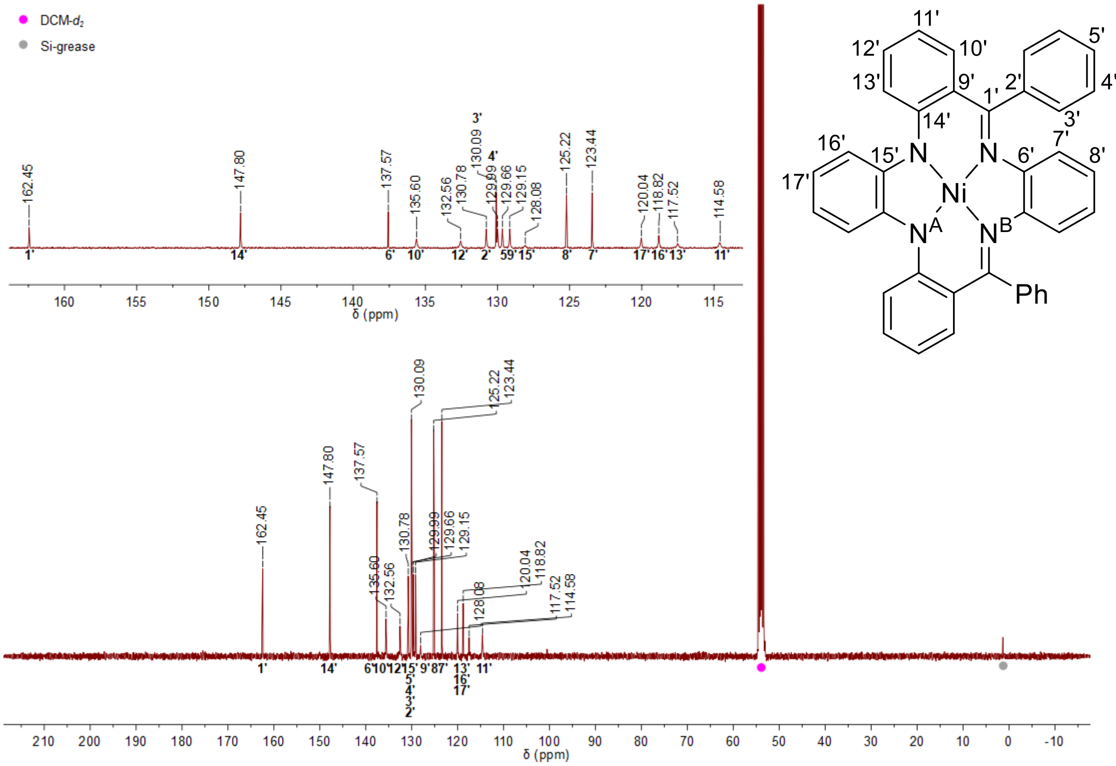

Figure S100. ^{13}C-NMR spectrum of **4c** in CD_2Cl_2 (101 MHz).

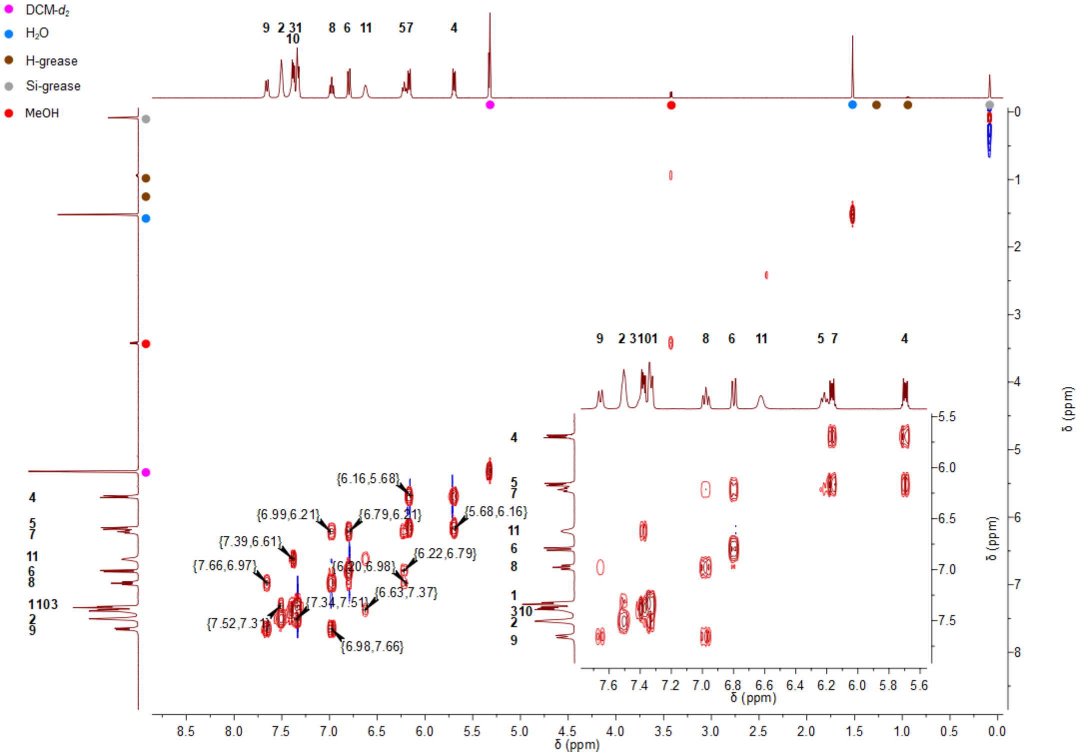

Figure S101. ^{1}H^{1}H-COSY NMR spectrum of **4c** in CD_2Cl_2 (400 MHz).

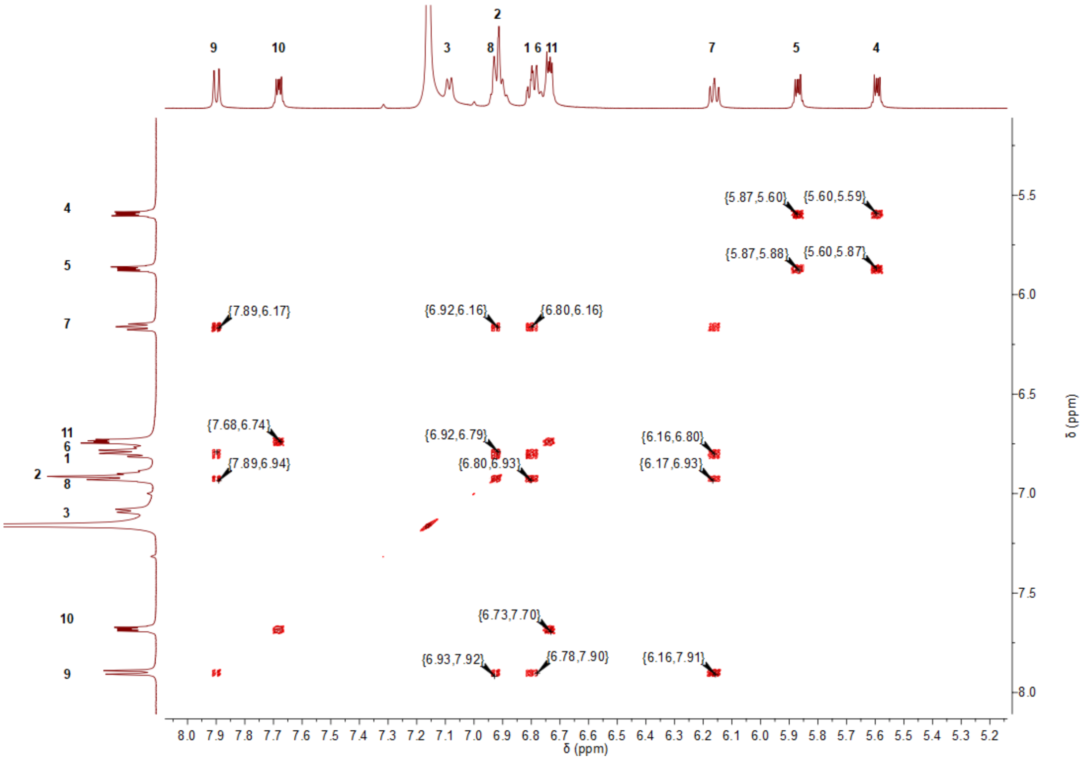

Figure S102. $^1H^1H$-COSY NMR spectrum of **4c** in C_6D_6 (500 MHz).

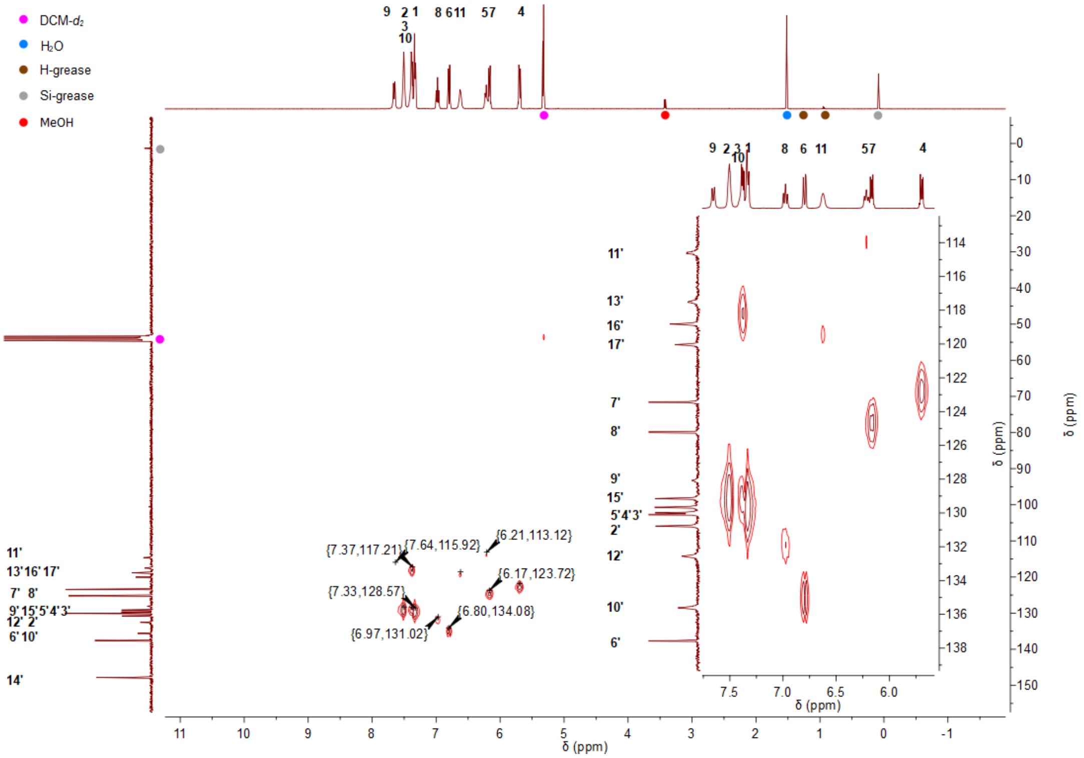

Figure S103. $^1H^{13}C$-HSQC NMR spectrum of **4c** in CD_2Cl_2 (400 MHz for ^1H, 101 MHz for ^{13}C).

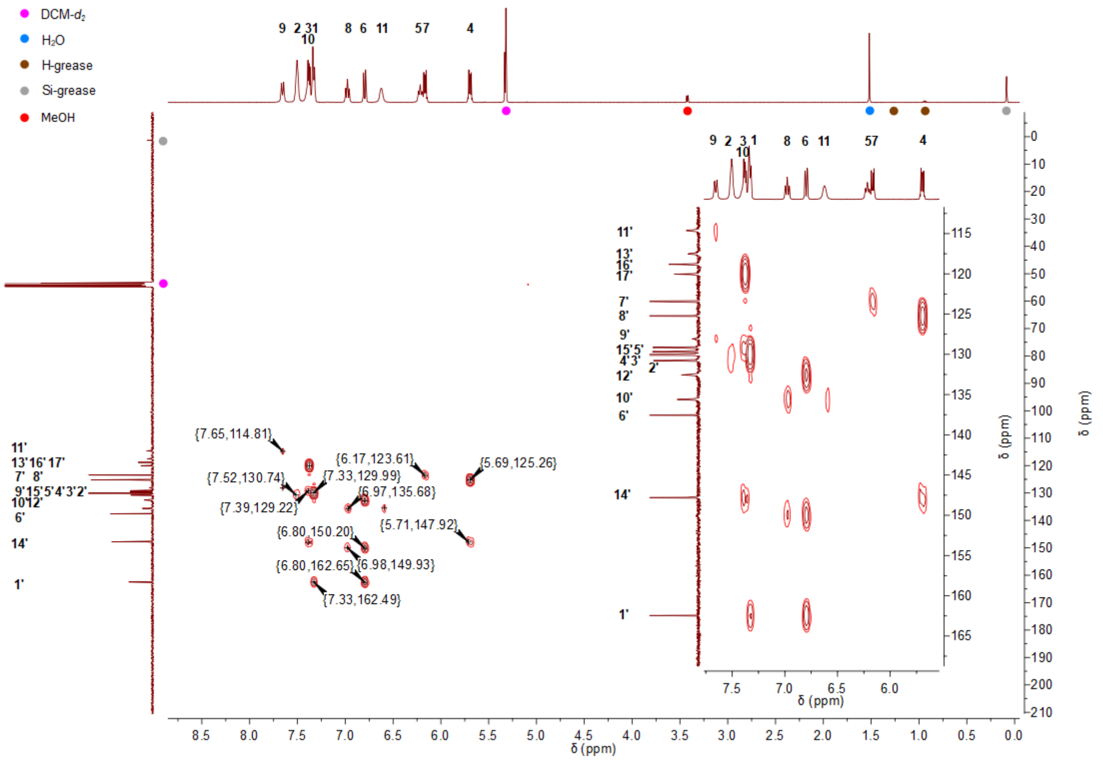

Figure S104. $^{1}H^{13}C$-HMBC NMR spectrum of **4c** in CD_2Cl_2 (400 MHz for ^{1}H, 101 MHz for ^{13}C).

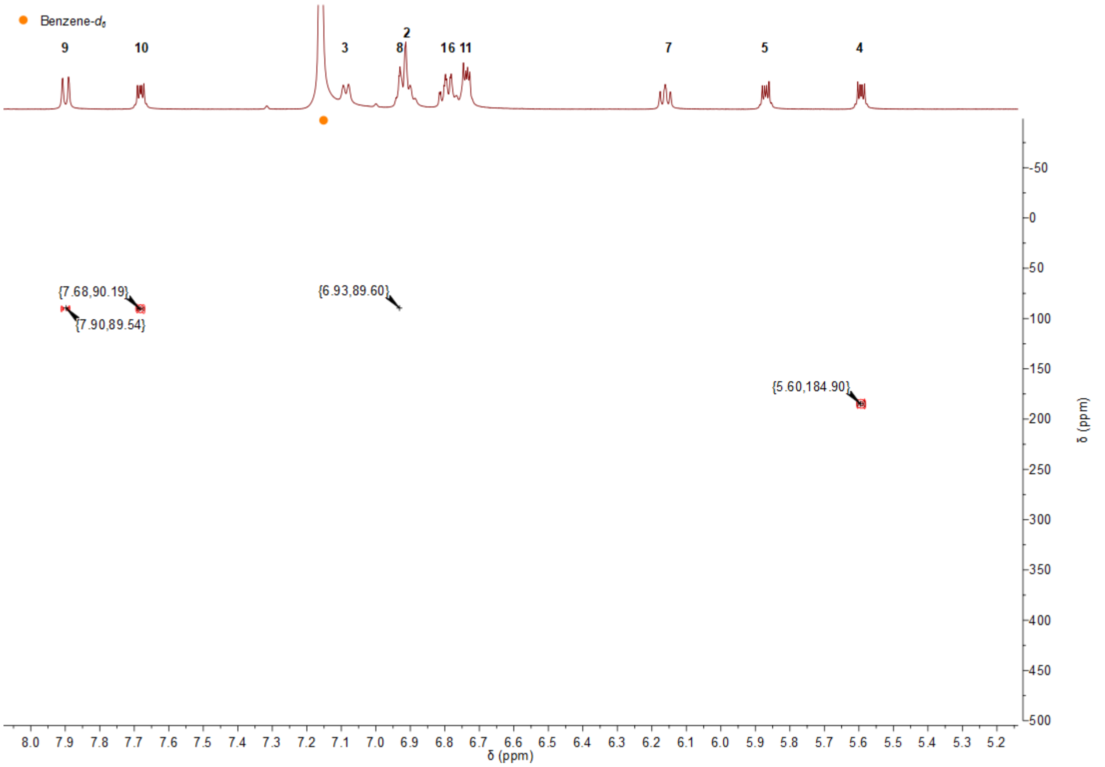

Figure S105. $^{1}H^{15}N$-HMBC NMR spectrum of **4c** in C_6D_6 (500 MHz for ^{1}H, 51 MHz for ^{15}N).

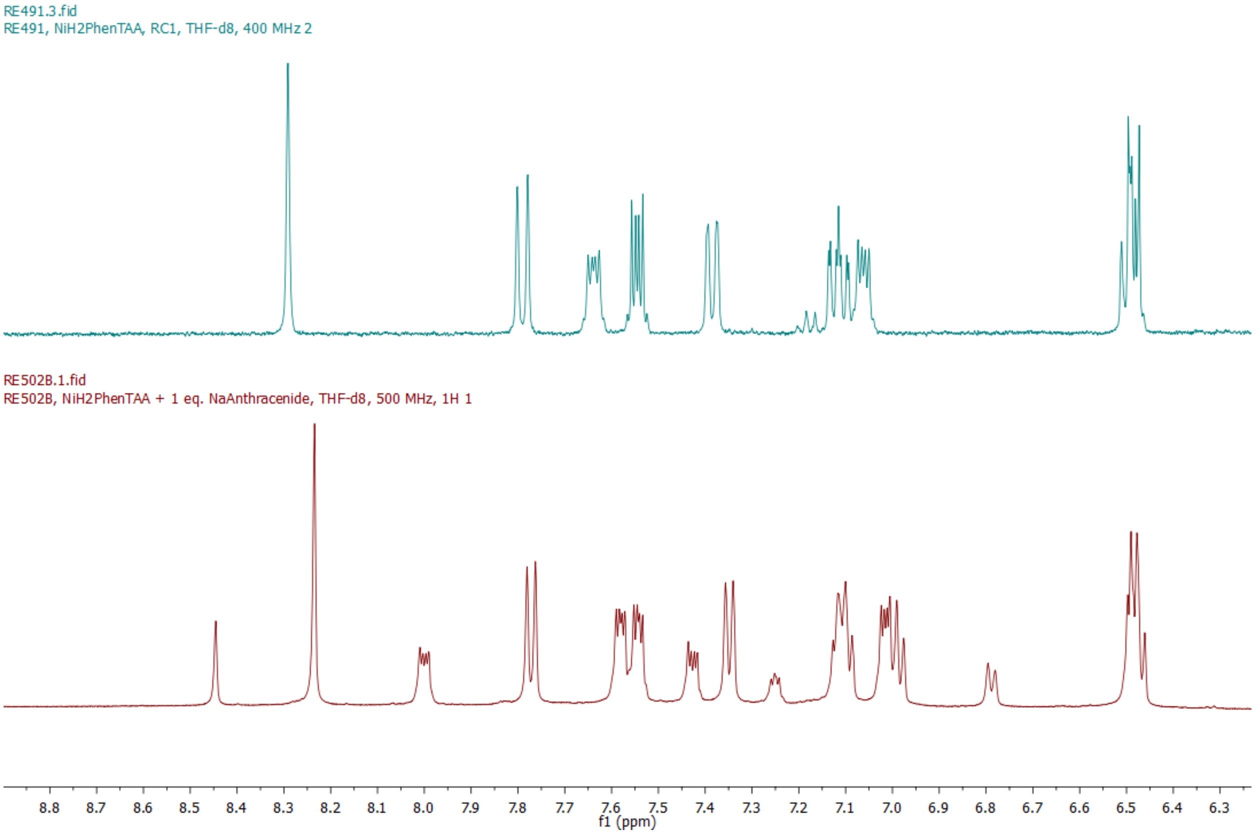

Figure S106. Comparison of **4a** in THF-d$_8$ (top) vs. the reaction mixture of **4a** + 1 eq. sodium anthracenide in THF-d$_8$ (300 MHz).

Table S8. Comparison of the ^{15}N-NMR chemical shift values obtained from ^{1}H^{15}N-HMBC measurements.

**Species**	^{15}N chemical shift (Ar$_2$***N***H) (δ ppm)	^{15}N chemical shift (***N***=CH) (δ ppm)
**3a**	99	301
**4a**	-	189
**4b**	-	187
**4c**	90	185

Density Functional Calculations

Table S9. Calculated $\langle s^2 \rangle$ values, spin states and energies (in Hartree) of [Ni(H_2PhenTAA)].

Charge	Spin state	$\langle s^2 \rangle$	SCF (Hartree)	ZPE correction (Hartree)	Enthalpy correction (Hartree)	Entropy correction (Hartree)	$\Delta G°_{298K}$ (Hartree)	Relative energies (kcal·mol^{-1})
**[Ni(H_2PhenTAA)]**								
**BP86/def2-TZVP/disp3**								
-2	**CSS**	**-**	**-2729.91016**	**0.35751**	**0.38258**	**0.30661**	**-2729.60355**	**0.0**
	Triplet	2.0087	-2729.89713	0.35661	0.38194	0.30522	-2729.59191	7.3
	Quintet	6.0139	-2729.84398	0.35350	0.37967	0.30037	-2729.54361	37.6
-1	**Doublet**	**0.7581**	**-2729.96760**	**0.36267**	**0.38714**	**0.31194**	**-2729.65567**	**0.0**
	Quartet	3.7588	-2729.91649	0.36004	0.38514	0.30831	-2729.60818	29.8
	Sextet	8.7614	-2729.86045	0.35817	0.38360	0.30576	-2729.55469	63.4
0	**CSS**	**-**	**-2729.90547**	**0.36741**	**0.39143**	**0.31686**	**-2729.58861**	**0.0**
	Triplet	2.0053	-2729.87203	0.36528	0.38965	0.31435	-2729.55767	19.4
	Quintet	6.0107	-2729.81520	0.36251	0.38757	0.31019	-2729.50501	52.5
1	**Doublet**	**0.7531**	**-2729.68933**	**0.36892**	**0.39292**	**0.31813**	**-2729.37120**	**0.0**
	Quartet	3.7660	-2729.64867	0.36674	0.39113	0.31553	-2729.33314	23.9
	Sextet	8.7644	-2729.57380	0.36280	0.38800	0.30996	-2729.26384	67.4
2	**CSS**	**-**	**-2729.34766**	**0.36979**	**0.39381**	**0.31900**	**-2729.02865**	**0.0**
	Triplet	2.0115	-2729.33917	0.36908	0.39306	0.31832	-2729.02084	4.9
	Quintet	6.0132	-2729.28225	0.36614	0.39077	0.31452	-2728.96773	38.2
3	**Doublet**	**0.75800**	**-2728.86714**	**0.36851**	**0.39265**	**0.31762**	**-2728.54951**	**0.0**
	Quartet	3.75810	-2728.84639	0.36756	0.39184	0.31640	-2728.53	12.2
	Sextet	8.76510	-2728.78778	0.36502	0.38994	0.31298	-2728.4748	46.9
**B3-LYP/def2-TZVP/disp3**								
-2	CSS	-	-2728.70556	0.36763	0.39189	0.31739	-2728.38816	4.4
	**OSS[(a)]**	**0.7888**	**-2728.70879**	**0.36717**	**0.39153**	**0.31679**	**-2728.39199**	**0.0**
	Triplet	2.0290	-2728.70435	0.36707	0.39152	0.31640	-2728.38796	4.5
	Quintet	6.0333	-2728.66691	0.36425	0.38946	0.31206	-2728.35485	25.3
-1	**Doublet**	**0.7781**	**-2728.77902**	**0.37249**	**0.39621**	**0.32235**	**-2728.45667**	**0.0**
	Quartet	3.7782	-2728.73771	0.36929	0.39390	0.31744	-2728.42027	22.8
	Sextet	8.7780	-2728.68687	0.36901	0.39366	0.31715	-2728.36972	54.6
0	**CSS**	**-**	**-2728.73253**	**0.37822**	**0.40144**	**0.32839**	**-2728.40415**	**0.0**
	Triplet	2.0224	-2728.68781	0.37522	0.39886	0.32499	-2728.36283	25.9
	Quintet	6.0235	-2728.64720	0.37241	0.39685	0.32050	-2728.32670	48.6
1	**Doublet**	**0.7596**	**-2728.52178**	**0.37978**	**0.40300**	**0.32968**	**-2728.19210**	**0.0**
	Quartet	3.7765	-2728.48652	0.37767	0.40145	0.32657	-2728.15996	20.2
	Sextet	8.7845	-2728.40564	0.37254	0.39710	0.32033	-2728.08531	67.0
2	**CSS**	**-**	**-2728.18227**	**0.38088**	**0.40413**	**0.33077**	**-2727.85150**	**0.0**
	Triplet	2.0400	-2728.17232	0.37957	0.40278	0.32954	-2727.84278	5.5
	Quintet	6.0491	-2728.13487	0.37679	0.40078	0.32553	-2727.80935	26.4
3	**Doublet**	**0.80450**	**-2727.69747**	**0.37907**	**0.40247**	**0.32888**	**-2727.36859**	**0.0**
	Quartet	3.77950	-2727.68002	0.37784	0.40135	0.32737	-2727.35265	10.0
	Sextet	8.78550	-2727.64614	0.37530	0.39951	0.32383	-2727.32231	29.0
**M06-L/def2-TZVP/disp3**								
-2	**CSS**	**-**	**-2729.27686**	**0.37139**	**0.39524**	**0.32168**	**-2728.95517**	**0.0**
	Triplet	2.0191	-2729.26729	0.37029	0.39443	0.32007	-2728.94721	5.0
	Quintet	6.0260	-2729.22693	0.36806	0.39274	0.31675	-2728.91018	28.2
-1	**Doublet**	**0.7662**	**-2729.34763**	**0.37601**	**0.39941**	**0.32636**	**-2729.02128**	**0.0**
	Quartet	3.7718	-2729.30376	0.37342	0.39748	0.32258	-2728.98119	25.2
	Sextet	8.7729	-2729.25167	0.37215	0.39637	0.32117	-2728.93050	57.0
0	**CSS**	**-**	**-2729.29642**	**0.38053**	**0.40357**	**0.33101**	**-2728.96541**	**0.0**
	Triplet	2.0133	-2729.25883	0.37760	0.40114	0.32754	-2728.93129	21.4
	Quintet	6.0156	-2729.21416	0.37603	0.39988	0.32538	-2728.88878	48.1
1	**Doublet**	**0.7564**	**-2729.08650**	**0.38148**	**0.40455**	**0.33175**	**-2728.75475**	**0.0**
	Quartet	3.7796	-2729.05071	0.37919	0.40280	0.32860	-2728.72211	20.5
	Sextet	8.7772	-2728.97608	0.37524	0.39933	0.32406	-2728.65202	64.5
2	**CSS**	**-**	**-2728.74870**	**0.38193**	**0.40506**	**0.33220**	**-2728.41650**	**0.0**
	Triplet	2.0228	-2728.74024	0.38101	0.40409	0.33129	-2728.40895	4.7
	Quintet	6.0358	-2728.69920	0.37918	0.40275	0.32872	-2728.37048	28.9
3	**Doublet**	**0.76690**	**-2728.26974**	**0.37995**	**0.40324**	**0.33005**	**-2727.93969**	**0.0**
	Quartet	3.76730	-2728.25029	0.37911	0.40248	0.32900	-2727.92129	11.5
	Sextet	8.77740	-2728.21727	0.37613	0.40021	0.32474	-2727.89253	29.6

[(a)] Energy corrected according to formula 1 in general considerations. For all other integer spin states, the open-shell singlet converged to the closed-shell singlet.

Table S10. Calculated <s^2> values, spin states and energies (in Hartree) of [Ni(Me$_2$PhenTAA)].

Charge	Spin state	<s^2>	SCF (Hartree)	ZPE correction (Hartree)	Enthalpy correction (Hartree)	Entropy correction (Hartree)	ΔG°$_{298K}$ (Hartree)	Relative energies (kcal·mol^{-1})
**[Ni(Me$_2$PhenTAA)]**								
**BP86/def2-TZVP/disp3**								
-2	**CSS**	**-**	**-2808.56253**	**0.41218**	**0.43997**	**0.35881**	**-2808.20372**	**0.0**
	Triplet	2.0082	-2808.54381	0.41108	0.43917	0.3572	-2808.18662	10.7
	Quintet	6.0141	-2808.48579	0.40879	0.43744	0.3537	-2808.1321	44.9
-1	**Doublet**	**0.7569**	**-2808.61574**	**0.41742**	**0.44467**	**0.36416**	**-2808.25158**	**0.0**
	Quartet	3.7591	-2808.56790	0.41490	0.44177	0.44271	-2808.2071	27.9
	Sextet	8.7620	-2808.50320	0.41297	0.44109	0.35811	-2808.14509	66.8
0	**CSS**	**-**	**-2808.55189**	**0.42207**	**0.44890**	**0.36911**	**-2808.18277**	**0.0**
	Triplet	2.0058	-2808.52575	0.42010	0.44726	0.36661	-2808.15914	14.8
	Quintet	6.0126	-2808.46235	0.41668	0.44447	0.36207	-2808.10028	51.8
1	**Doublet**	**0.7535**	**-2808.34297**	**0.42355**	**0.45030**	**0.37046**	**-2807.97251**	**0.0**
	Quartet	3.7655	-2808.30719	0.42159	0.44869	0.36794	-2807.93925	20.9
	Sextet	8.7661	-2808.23309	0.41789	0.44577	0.3631	-2807.87	64.3
2	**CSS**	**-**	**-2808.00922**	**0.42440**	**0.45112**	**0.37134**	**-2807.63788**	**0.0**
	Triplet	2.0125	-2808.00106	0.42367	0.45040	0.37056	-2807.63049	4.6
	Quintet	6.0128	-2807.95080	0.42079	0.44821	0.36658	-2807.58422	33.7
3	**Doublet**	**0.7625**	**-2807.53743**	**0.42304**	**0.44991**	**0.36982**	**-2807.16762**	**0.7625**
	Quartet	3.7596	-2807.51840	0.42176	0.44884	0.36824	-2807.15015	3.7596
	Sextet	8.7663	-2807.46326	0.41927	0.44703	0.36457	-2807.09869	8.7663
**B3-LYP/def2-TZVP/disp3**								
-2	**CSS**	**-**	**-2807.29490**	**0.42395**	**0.45080**	**0.37139**	**-2806.92352**	**0.0**
	Triplet	2.0264	-2807.28801	0.42316	0.45028	0.37008	-2806.91792	3.5
	Quintet	6.0317	-2807.24504	0.42137	0.44897	0.36718	-2806.87787	28.6
-1	**Doublet**	**0.7746**	**-2807.36574**	**0.42900**	**0.45540**	**0.37645**	**-2806.98929**	**0.0**
	Quartet	3.7746	-2807.31329	0.42707	0.45387	0.37398	-2806.93931	31.4
	Sextet	8.7806	-2807.26764	0.42554	0.45278	0.37138	-2806.89626	58.4
0	**CSS**	**-**	**-2807.31767**	**0.43442**	**0.46036**	**0.38223**	**-2806.93544**	**0.0**
	Triplet	2.0164	-2807.28199	0.43223	0.45875	0.37888	-2806.90311	20.3
	Quintet	6.0281	-2807.22954	0.43003	0.45679	0.37628	-2806.85325	51.6
1	**Doublet**	**0.7604**	**-2807.11253**	**0.43599**	**0.46184**	**0.38369**	**-2806.72884**	**0.0**
	Quartet	3.7801	-2807.07781	0.43426	0.46057	0.38094	-2806.69687	20.1
	Sextet	8.7989	-2807.00000	0.43029	0.45739	0.37589	-2806.62423	65.6
2	**CSS**	**-**	**-2806.77975**	**0.43710**	**0.46294**	**0.38486**	**-2806.39489**	**0.0**
	Triplet	2.0409	-2806.77215	0.43576	0.46165	0.38338	-2806.38878	3.8
	Quintet	6.0521	-2806.73375	0.43388	0.46024	0.38063	-2806.35312	26.2
3	**Doublet**	**0.8072**	**-2806.30418**	**0.43535**	**0.46136**	**0.38289**	**-2805.92129**	**0.8072**
	Quartet	3.7812	-2806.28894	0.43368	0.45990	0.38094	-2805.90800	3.7812
	Sextet	8.7884	-2806.25391	0.43191	0.45863	0.37828	-2805.87563	8.7884
**M06-L/def2-TZVP/disp3**								
-2	**CSS**	**-**	**-2807.90991**	**0.42851**	**0.45487**	**0.37661**	**-2807.53331**	**0.0**
	Triplet	2.0185	-2807.89492	0.42748	0.45412	0.37512	-2807.5198	8.5
	Quintet	6.0275	-2807.84948	0.42588	0.45295	0.37246	-2807.47702	35.3
-1	**Doublet**	**0.7651**	**-2807.97688**	**0.43339**	**0.45934**	**0.38156**	**-2807.59532**	**0.0**
	Quartet	3.7776	-2807.92754	0.43074	0.45734	0.37763	-2807.5499	28.5
	Sextet	8.7789	-2807.87502	0.42918	0.45608	0.37562	-2807.4994	60.2
0	**CSS**	**-**	**-2807.92394**	**0.43765**	**0.46330**	**0.38603**	**-2807.53792**	**0.0**
	Triplet	2.0164	-2807.89420	0.43508	0.46118	0.38271	-2807.51149	16.6
	Quintet	6.0255	-2807.83852	0.43306	0.45953	0.37983	-2807.45869	49.7
1	**Doublet**	**0.7572**	**-2807.72067**	**0.43866**	**0.46427**	**0.3869**	**-2807.33377**	**0.0**
	Quartet	3.7828	-2807.68819	0.43661	0.46266	0.38405	-2807.30414	18.6
	Sextet	8.7867	-2807.61171	0.43308	0.45970	0.37975	-2807.23195	63.9
2	**CSS**	**-**	**-2807.39030**	**0.43916**	**0.46479**	**0.38741**	**-2807.00289**	**0.0**
	Triplet	2.0242	-2807.38291	0.43820	0.46384	0.38638	-2806.99653	4.0
	Quintet	6.0380	-2807.34290	0.43657	0.46261	0.38405	-2806.95886	27.6
3	**Doublet**	**0.7746**	**-2806.92004**	**0.43719**	**0.46299**	**0.38524**	**-2806.53481**	**0.7746**
	Quartet	3.7694	-2806.90269	0.43604	0.46195	0.38396	-2806.51873	3.7694
	Sextet	8.7807	-2806.86603	0.43455	0.46089	0.38173	-2806.48430	8.7807

Table S11. Calculated <s^2> values, spin states and energies (in Hartree) of [Ni(Ph_2PhenTAA)].

Charge	Spin state	<s^2>	SCF (Hartree)	ZPE correction (Hartree)	Enthalpy correction (Hartree)	Entropy correction (Hartree)	ΔG°$_{298K}$ (Hartree)	Relative energies (kcal·mol^{-1})
**[Ni(Ph_2PhenTAA)]**								
**BP86/def2-TZVP/disp3**								
-2	**CSS**	**-**	**-3192.22536**	**0.51440**	**0.54908**	**0.45113**	**-3191.77423**	**0.0**
	Triplet	2.0072	-3192.21149	0.51383	0.54859	0.45043	-3191.76106	8.3
	Quintet	6.0129	-3192.15456	0.51120	0.54665	0.44638	-3191.70818	41.4
-1	**Doublet**	**0.7565**	**-3192.26544**	**0.51956**	**0.55378**	**0.45639**	**-3191.80905**	**0.0**
	Quartet	3.7581	-3192.22011	0.51750	0.55209	0.45365	-3191.76646	26.7
	Sextet	8.7620	-3192.15730	0.51505	0.55015	0.44956	-3191.70775	63.6
0	**CSS**	**-**	**-3192.19946**	**0.52411**	**0.55796**	**0.46097**	**-3191.73848**	**0.0**
	Triplet	2.0051	-3192.17037	0.52225	0.55639	0.45868	-3191.71169	16.8
	Quintet	6.0129	-3192.10757	0.51917	0.55382	0.45448	-3191.65309	53.6
1	**Doublet**	**0.7533**	**-3191.99354**	**0.52575**	**0.55952**	**0.46249**	**-3191.53104**	**0.0**
	Quartet	3.7662	-3191.95263	0.52368	0.55785	0.45960	-3191.49302	23.9
	Sextet	8.7666	-3191.88098	0.52083	0.55539	0.45628	-3191.42471	66.7
2	**CSS**	**-**	**-3191.66855**	**0.52671**	**0.56044**	**0.46371**	**-3191.20485**	**0.0**
	Triplet	2.0125	-3191.65981	0.52582	0.55957	0.46263	-3191.19718	4.8
	Quintet	6.0134	-3191.60798	0.52344	0.55769	0.45951	-3191.14846	35.4
3	**Doublet**	**0.7541**	**-3191.21329**	**0.52406**	**0.55814**	**0.46068**	**-3190.75261**	**0.0**
	Quartet	3.7641	-3191.20300	0.52452	0.55843	0.46147	-3190.74153	7.0
	Sextet	8.7680	-3191.14454	0.52152	0.55620	0.45673	-3190.68781	40.7
**B3-LYP/def2-TZVP/disp3**								
-2	**CSS**	**-**	**-3190.70211**	**0.52884**	**0.56241**	**0.46650**	**-3190.23561**	**0.0**
	Triplet	2.0242	-3190.69908	0.52870	0.56227	0.46632	-3190.23276	1.8
	Quintet	6.0261	-3190.65498	0.52738	0.56134	0.46146	-3190.19082	28.1
-1	**Doublet**	**0.7748**	**-3190.76053**	**0.53377**	**0.56695**	**0.47130**	**-3190.28923**	**0.0**
	Quartet	3.7753	-3190.71053	0.53246	0.56585	0.46981	-3190.24072	30.4
	Sextet	8.7816	-3190.66661	0.53083	0.56464	0.46735	-3190.19926	56.5
0	**CSS**	**-**	**-3190.71079**	**0.53919**	**0.57194**	**0.47711**	**-3190.23368**	**0.0**
	Triplet	2.0210	-3190.66845	0.53660	0.56972	0.47367	-3190.19478	24.4
	Quintet	6.0256	-3190.62149	0.53495	0.56858	0.47086	-3190.15063	52.1
1	**Doublet**	**0.7605**	**-3190.50860**	**0.54092**	**0.57356**	**0.47888**	**-3190.02972**	**0.0**
	Quartet	3.7793	-3190.46985	0.53838	0.57176	0.47432	-3189.99553	21.5
	Sextet	8.7935	-3190.39401	0.53569	0.56928	0.47182	-3189.92219	67.5
2	**CSS**	**-**	**-3190.18367**	**0.54215**	**0.57476**	**0.48024**	**-3189.70343**	**0.0**
	Triplet	2.0430	-3190.17583	0.54075	0.57344	0.47857	-3189.69726	3.9
	Quintet	6.0519	-3190.13469	0.53882	0.57203	0.47557	-3189.65912	27.8
3	**Doublet**	**0.7601**	**-3189.72727**	**0.54065**	**0.57340**	**0.47885**	**-3189.24841**	**0.0**
	Quartet	3.7895	-3189.71677	0.53939	0.57217	0.47748	-3189.23929	5.7
	Sextet	8.7956	-3189.67835	0.53765	0.57093	0.47480	-3189.20354	28.2
**M06-L/def2-TZVP/disp3**								
-2	CSS	**-**	**-3191.48127**	**0.53385**	**0.56692**	**0.47261**	**-3191.00866**	**0.0**
	Triplet	2.0166	-3191.47096	0.53328	0.56645	0.47155	-3190.99941	5.8
	Quintet	6.0225	-3191.42571	0.53172	0.56529	0.46945	-3190.95626	32.9
-1	Doublet	**0.7649**	**-3191.53367**	**0.53815**	**0.57101**	**0.4764**	**-3191.05727**	**0.0**
	Quartet	3.7708	-3191.48663	0.53584	0.56913	0.47360	-3191.01304	27.8
	Sextet	8.7785	-3191.43842	0.53485	0.56830	0.47249	-3190.96593	57.3
0	CSS	**-**	**-3191.47794**	**0.54261**	**0.57515**	**0.48096**	**-3190.99698**	**0.0**
	Triplet	2.0137	-3191.44499	0.54000	0.57296	0.47779	-3190.96720	18.7
	Quintet	6.0240	-3191.39163	0.53822	0.57162	0.47467	-3190.91697	50.2
1	Doublet	**0.7567**	**-3191.27735**	**0.54366**	**0.57619**	**0.48183**	**-3190.79552**	**0.0**
	Quartet	3.7834	-3191.23998	0.54133	0.57436	0.47860	-3190.76138	21.4
	Sextet	8.7857	-3191.16823	0.53883	0.57211	0.47577	-3190.69246	64.7
2	CSS	**-**	**-3190.95534**	**0.54423**	**0.57676**	**0.48267**	**-3190.47267**	**0.0**
	Triplet	2.0248	-3190.94753	0.54331	0.57586	0.48142	-3190.46611	4.1
	Quintet	6.0328	-3190.89946	0.54098	0.57411	0.47804	-3190.42142	32.2
3	**Doublet**	0.7578	-3190.50345	0.54142	0.57423	0.48005	-3190.02341	0.0
	Quartet	3.7732	-3190.50177	0.54137	0.57404	0.48010	-3190.02168	1.1
	Sextet	8.7853	-3190.45262	0.53972	0.57286	0.47759	-3189.97503	30.4

Single-point COSMO calculations (2-MeTHF)

Table S12. Calculated <s²> values, spin states and energies (in Hartree) of [Ni(H_2PhenTAA)] (single-point COSMO (2-MeTHF)).

Charge	Spin state	$\langle s^2 \rangle$	SCF (Hartree)	ZPE correction (Hartree)	Enthalpy correction (Hartree)	Entropy correction (Hartree)	$\Delta G°_{298K}$ (Hartree)	Relative energies (kcal·mol^{-1})
**[Ni(H_2PhenTAA)] (COSMO 2-MeTHF (ε = 6.97)**								
**BP86/def2-TZVP/disp3**								
-2	**CSS**	**-**	**-2730.08753**	**0.35751**	**0.38258**	**0.30661**	**-2729.78092**	**0.0**
	Triplet	2.0091	-2730.07385	0.35661	0.38194	0.30522	-2729.76863	7.7
	Quintet	6.0146	-2730.01939	0.35350	0.37967	0.30037	-2729.71902	38.8
-1	**Doublet**	**0.7577**	**-2730.02224**	**0.36267**	**0.38714**	**0.31194**	**-2729.71030**	**0.0**
	Quartet	3.7589	-2729.96999	0.36004	0.38514	0.30831	-2729.66168	30.5
	Sextet	8.7618	-2729.91364	0.35817	0.38360	0.30576	-2729.60788	64.3
0	**CSS**	**-**	**-2729.91806**	**0.36741**	**0.39143**	**0.31686**	**-2729.60120**	**0.0**
	Triplet	2.0053	-2729.88314	0.36528	0.38965	0.31435	-2729.56879	20.3
	Quintet	6.0107	-2729.82592	0.36251	0.38757	0.31019	-2729.51573	53.6
1	**Doublet**	**0.7531**	**-2729.73953**	**0.36892**	**0.39292**	**0.31813**	**-2729.42140**	**0.0**
	Quartet	3.7659	-2729.69797	0.36674	0.39113	0.31553	-2729.38244	24.4
	Sextet	8.7644	-2729.62279	0.36280	0.38800	0.30996	-2729.31283	68.1
2	**CSS**	**-**	**-2729.51829**	**0.36979**	**0.39381**	**0.31900**	**-2729.19929**	**0.0**
	Triplet	2.0116	-2729.50947	0.36908	0.39306	0.31832	-2729.19115	5.1
	Quintet	6.0133	-2729.45121	0.36614	0.39077	0.31452	-2729.13669	39.3
3	**Doublet**	**0.7573**	**-2729.23942**	**0.36851**	**0.39265**	**0.31762**	**-2728.92180**	**0.0**
	Quartet	3.7579	-2729.21720	0.36756	0.39184	0.31640	-2728.90080	13.2
	Sextet	8.7650	-2729.15811	0.36502	0.38994	0.31298	-2728.84513	48.1
**B3-LYP/def2-TZVP/disp3**								
-2	CSS	-	-2728.88226	0.36763	0.39189	0.31739	-2728.56487	4.4
	**OSS**	**0.7677**	**-2728.88533**	**0.36717**	**0.39153**	**0.31679**	**-2728.57181**	**0.0**
	Triplet	2.0297	-2728.88060	0.36707	0.39152	0.31640	-2728.56420	4.8
	Quintet	6.0344	-2728.84096	0.36425	0.38946	0.31206	-2728.52890	26.9
-1	**Doublet**	**0.7771**	**-2728.83413**	**0.37249**	**0.39621**	**0.32235**	**-2728.51178**	**0.0**
	Quartet	3.7783	-2728.79148	0.36929	0.39390	0.31744	-2728.47404	23.7
	Sextet	8.7785	-2728.73988	0.36901	0.39366	0.31715	-2728.42273	55.9
0	**CSS**	**-**	**-2728.74519**	**0.37822**	**0.40144**	**0.32839**	**-2728.41680**	**0.0**
	Triplet	2.0222	-2728.69858	0.37522	0.39886	0.32499	-2728.37359	27.1
	Quintet	6.0229	-2728.65789	0.37241	0.39685	0.32050	-2728.33739	49.8
1	**Doublet**	**0.7596**	**-2728.57245**	**0.37978**	**0.40300**	**0.32968**	**-2728.24277**	**0.0**
	Quartet	3.7747	-2728.53704	0.37767	0.40145	0.32657	-2728.21047	20.3
	Sextet	8.7838	-2728.45537	0.37254	0.39710	0.32033	-2728.13504	67.6
2	**CSS**	**-**	**-2728.35494**	**0.38088**	**0.40413**	**0.33077**	**-2728.02417**	**0.0**
	Triplet	2.0399	-2728.34392	0.37957	0.40278	0.32954	-2728.01438	6.1
	Quintet	6.0501	-2728.30759	0.37679	0.40078	0.32553	-2727.98206	26.4
3	**Doublet**	**0.8064**	**-2728.07263**	**0.37907**	**0.40247**	**0.32888**	**-2727.74375**	**0.0**
	Quartet	3.7789	-2728.05267	0.37784	0.40135	0.32737	-2727.72530	11.6
	Sextet	8.7853	-2728.01937	0.37530	0.39951	0.32383	-2727.69554	30.3
**M06-L/def2-TZVP/disp3**								
-2	**CSS**	**-**	**-2729.45182**	**0.37139**	**0.39524**	**0.32168**	**-2729.13014**	**0.0**
	Triplet	2.0198	-2729.44170	0.37029	0.39443	0.32007	-2729.12163	5.3
	Quintet	6.0280	-2729.39923	0.36806	0.39274	0.31675	-2729.08248	29.9
-1	**Doublet**	**0.7581**	**-2729.12830**	**0.37601**	**0.39941**	**0.32636**	**-2728.80194**	**0.0**
	Quartet	3.7779	-2729.09318	0.37342	0.39748	0.32258	-2728.77060	19.7
	Sextet	8.7694	-2729.02239	0.37215	0.39637	0.32117	-2728.70122	63.2
0	**CSS**	**-**	**-2729.30618**	**0.38053**	**0.40357**	**0.33101**	**-2728.97517**	**0.0**
	Triplet	2.0137	-2729.26704	0.37760	0.40114	0.32754	-2728.93950	22.4
	Quintet	6.0156	-2729.22232	0.37603	0.39988	0.32538	-2728.89694	49.1
1	**Doublet**	**0.7564**	**-2729.13453**	**0.38148**	**0.40455**	**0.33175**	**-2728.80278**	**0.0**
	Quartet	3.7783	-2729.09827	0.37919	0.40280	0.32860	-2728.76967	20.8
	Sextet	8.7768	-2729.02312	0.37524	0.39933	0.32406	-2728.69906	65.1
2	**CSS**	**-**	**-2728.91871**	**0.38193**	**0.40506**	**0.33220**	**-2728.58651**	**0.0**
	Triplet	2.0231	-2728.90986	0.38101	0.40409	0.33129	-2728.57857	5.0
	Quintet	6.0370	-2728.87001	0.37918	0.40275	0.32872	-2728.54129	28.4
3	**Doublet**	**0.7657**	**-2728.64325**	**0.37995**	**0.40324**	**0.33005**	**-2728.31320**	**0.0**
	Quartet	3.7673	-2728.62205	0.37911	0.40248	0.32900	-2728.29305	12.6
	Sextet	8.7782	-2728.58505	0.37613	0.40021	0.32474	-2728.26031	33.2

Table S13. Calculated <s^2> values, spin states and energies (in Hartree) of [Ni(Me$_2$PhenTAA)] (single-point COSMO (2-MeTHF)).

[Ni(Me$_2$PhenTAA)] (COSMO 2-MeTHF (ε = 6.97)								
**BP86/def2-TZVP/disp3**								
**Charge**	**Spin state**	**<s^2>**	**SCF (Hartree)**	**ZPE correction (Hartree)**	**Enthalpy correction (Hartree)**	**Entropy correction (Hartree)**	**ΔG°$_{298K}$ (Hartree)**	**Relative energies (kcal·mol^{-1})**
-2	**CSS**	**-**	**-2808.73804**	**0.41218**	**0.43997**	**0.35881**	**-2808.37923**	**0.0**
	Triplet	2.0085	-2808.71927	0.41108	0.43917	0.35720	-2808.36207	10.8
	Quintet	6.0146	-2808.65970	0.40879	0.43744	0.35370	-2808.30600	46.0
-1	**Doublet**	**0.7565**	**-2808.67078**	**0.41742**	**0.44467**	**0.36416**	**-2808.30662**	**0.0**
	Quartet	3.7593	-2808.62122	0.41490	0.44271	0.36080	-2808.26042	29.0
	Sextet	8.7623	-2808.55564	0.41297	0.44109	0.35811	-2808.19753	68.5
0	**CSS**	**-**	**-2808.56515**	**0.42207**	**0.44890**	**0.36911**	**-2808.19604**	**0.0**
	Triplet	2.0059	-2808.53717	0.42010	0.44726	0.36661	-2808.17056	16.0
	Quintet	6.0126	-2808.47321	0.41668	0.44447	0.36207	-2808.11114	53.3
1	**Doublet**	**0.7535**	**-2808.39198**	**0.42355**	**0.45030**	**0.37046**	**-2808.02152**	**0.0**
	Quartet	3.7654	-2808.35542	0.42159	0.44869	0.36794	-2807.98748	21.4
	Sextet	8.7662	-2808.28109	0.41789	0.44577	0.36310	-2807.91799	65.0
2	**CSS**	**-**	**-2808.17540**	**0.42440**	**0.45112**	**0.37134**	**-2807.80406**	**0.0**
	Triplet	2.0126	-2808.16700	0.42367	0.45040	0.37056	-2807.79644	4.8
	Quintet	6.0130	-2808.11612	0.42079	0.44821	0.36658	-2807.74954	34.2
3	**Doublet**	**0.7603**	**-2807.90124**	**0.42304**	**0.44991**	**0.36982**	**-2807.53142**	**0.0**
	Quartet	3.7598	-2807.88131	0.42176	0.44884	0.36824	-2807.51307	11.5
	Sextet	8.7665	-2807.82623	0.41927	0.44703	0.36457	-2807.46166	43.8
**B3-LYP/def2-TZVP/disp3**								
-2	**CSS**	**-**	**-2807.46958**	**0.42395**	**0.45080**	**0.37139**	**-2807.09819**	**0.0**
	Triplet	2.0272	-2807.46283	0.42316	0.45028	0.37008	-2807.09275	3.4
	Quintet	6.0328	-2807.41753	0.42137	0.44897	0.36718	-2807.05035	30.0
-1	**Doublet**	**0.7736**	**-2807.42022**	**0.42900**	**0.45540**	**0.37645**	**-2807.04377**	**0.0**
	Quartet	3.7745	-2807.36597	0.42707	0.45387	0.37398	-2806.99199	32.5
	Sextet	8.7812	-2807.31944	0.42554	0.45278	0.37138	-2806.94806	60.1
0	**CSS**	**-**	**-2807.33104**	**0.43442**	**0.46036**	**0.38223**	**-2806.94881**	**0.0**
	Triplet	2.0159	-2807.29454	0.43223	0.45875	0.37888	-2806.91566	20.8
	Quintet	6.0277	-2807.24062	0.43003	0.45679	0.37628	-2806.86434	53.0
1	**Doublet**	**0.7606**	**-2807.16181**	**0.43599**	**0.46184**	**0.38369**	**-2806.77812**	**0.0**
	Quartet	3.7791	-2807.12684	0.43426	0.46057	0.38094	-2806.74590	20.2
	Sextet	8.7992	-2807.05091	0.43029	0.45739	0.37589	-2806.67502	64.7
2	**CSS**	**-**	**-2806.94770**	**0.43710**	**0.46294**	**0.38486**	**-2806.56284**	**0.0**
	Triplet	2.0412	-2806.93921	0.43576	0.46165	0.38338	-2806.55583	4.4
	Quintet	6.0529	-2806.90240	0.43388	0.46024	0.38063	-2806.52177	25.8
3	**Doublet**	**0.8093**	**-2806.67104**	**0.43535**	**0.46136**	**0.38289**	**-2806.28815**	**0.0**
	Quartet	3.7819	-2806.65382	0.43368	0.45990	0.38094	-2806.27288	9.6
	Sextet	8.7895	-2806.62036	0.43191	0.45863	0.37828	-2806.24208	28.9
**M06-L/def2-TZVP/disp3**								
-2	**CSS**	**-**	**-2808.08311**	**0.42851**	**0.45487**	**0.37661**	**-2807.70650**	**0.0**
	Triplet	2.0191	-2808.06822	0.42748	0.45412	0.37512	-2807.69310	8.4
	Quintet	6.0288	-2808.02058	0.42588	0.45295	0.37246	-2807.64812	36.6
-1	**Doublet**	**0.7642**	**-2808.02909**	**0.43339**	**0.45934**	**0.38156**	**-2807.64753**	**0.0**
	Quartet	3.7769	-2807.97869	0.43074	0.45734	0.37763	-2807.60106	29.2
	Sextet	8.7792	-2807.92470	0.42918	0.45608	0.37562	-2807.54908	61.8
0	**CSS**	**-**	**-2807.93458**	**0.43765**	**0.46330**	**0.38603**	**-2807.54855**	**0.0**
	Triplet	2.0173	-2807.90298	0.43508	0.46118	0.38271	-2807.52027	17.7
	Quintet	6.0258	-2807.84692	0.43306	0.45953	0.37983	-2807.46709	51.1

1	**Doublet**	**0.7572**	**-2807.76767**	**0.43866**	**0.46427**	**0.38690**	**-2807.38077**	**0.0**
	Quartet	3.7824	-2807.73467	0.43661	0.46266	0.38405	-2807.35062	18.9
	Sextet	8.7865	-2807.65830	0.43308	0.45970	0.37975	-2807.27855	64.1
2	**CSS**	**-**	**-2807.55608**	**0.43916**	**0.46479**	**0.38741**	**-2807.16867**	**0.0**
	Triplet	2.0246	-2807.54835	0.43820	0.46384	0.38638	-2807.16197	4.2
	Quintet	6.0390	-2807.50975	0.43657	0.46261	0.38405	-2807.12570	27.0
3	**Doublet**	**0.7697**	**-2807.28541**	**0.43719**	**0.46299**	**0.38524**	**-2806.90017**	**0.0**
	Quartet	3.7700	-2807.26683	0.43604	0.46195	0.38396	-2806.88287	10.9
	Sextet	8.7816	-2807.23152	0.43455	0.46089	0.38173	-2806.84979	31.6

Table S14. Calculated <s²> values, spin states and energies (in Hartree) of [Ni(Ph_2PhenTAA)] (single-point COSMO (2-MeTHF)).

Charge	Spin state	<s²>	SCF (Hartree)	ZPE correction (Hartree)	Enthalpy correction (Hartree)	Entropy correction (Hartree)	ΔG°298K (Hartree)	Relative energies (kcal·mol⁻¹)
**[Ni(Ph_2PhenTAA)] (COSMO 2-MeTHF (ε = 6.97)**								
**BP86/def2-TZVP/disp3**								
-2	**CSS**	**-**	**-3192.39201**	**0.51440**	**0.54908**	**0.45113**	**-3191.94088**	**0.0**
	Triplet	2.0077	-3192.37708	0.51383	0.54859	0.45043	-3191.92665	8.9
	Quintet	6.0135	-3192.31876	0.51120	0.54665	0.44638	-3191.87238	43.0
-1	**Doublet**	**0.7564**	**-3192.32070**	**0.51956**	**0.55378**	**0.45639**	**-3191.86431**	**0.0**
	Quartet	3.7583	-3192.27312	0.51750	0.55209	0.45365	-3191.81947	28.1
	Sextet	8.7622	-3192.20962	0.51505	0.55015	0.44956	-3191.76006	65.4
0	**CSS**	**-**	**-3192.21418**	**0.52411**	**0.55796**	**0.46097**	**-3191.75321**	**0.0**
	Triplet	2.0052	-3192.18372	0.52225	0.55639	0.45868	-3191.72504	17.7
	Quintet	6.0129	-3192.12097	0.51917	0.55382	0.45448	-3191.66649	54.4
1	**Doublet**	**0.7533**	**-3192.03989**	**0.52575**	**0.55952**	**0.46249**	**-3191.57740**	**0.0**
	Quartet	3.7659	-3191.99921	0.52368	0.55785	0.45960	-3191.53961	23.7
	Sextet	8.7667	-3191.92791	0.52083	0.55539	0.45628	-3191.47163	66.4
2	**CSS**	**-**	**-3191.82347**	**0.52671**	**0.56044**	**0.46371**	**-3191.35976**	**0.0**
	Triplet	2.0126	-3191.81461	0.52582	0.55957	0.46263	-3191.35198	4.9
	Quintet	6.0133	-3191.76151	0.52344	0.55769	0.45951	-3191.30200	36.2
3	**Doublet**	**0.7566**	**-3191.55008**	**0.52406**	**0.55814**	**0.46068**	**-3191.08940**	**0.0**
	Quartet	3.7610	-3191.53630	0.52452	0.55843	0.46147	-3191.07483	9.1
	Sextet	8.7676	-3191.47686	0.52152	0.55620	0.45673	-3191.02013	43.5
**B3-LYP/def2-TZVP/disp3**								
-2	**CSS**	**-**	**-3190.86876**	**0.52884**	**0.56241**	**0.46650**	**-3190.40226**	**0.0**
	Triplet	2.0259	-3190.86489	0.52870	0.56227	0.46632	-3190.39857	2.3
	Quintet	6.0279	-3190.81863	0.52738	0.56134	0.46146	-3190.35717	28.3
-1	**Doublet**	**0.7742**	**-3190.81566**	**0.53377**	**0.56695**	**0.47130**	**-3190.34436**	**0.0**
	Quartet	3.7749	-3190.76345	0.53246	0.56585	0.46981	-3190.29364	31.8
	Sextet	8.7821	-3190.71895	0.53083	0.56464	0.46735	-3190.25160	58.2
0	**CSS**	**-**	**-3190.72558**	**0.53919**	**0.57194**	**0.47711**	**-3190.24847**	**0.0**
	Triplet	2.0210	-3190.68155	0.53660	0.56972	0.47367	-3190.20788	25.5
	Quintet	6.0253	-3190.63472	0.53495	0.56858	0.47086	-3190.16386	53.1
1	**Doublet**	**0.7605**	**-3190.55520**	**0.54092**	**0.57356**	**0.47888**	**-3190.07632**	**0.0**
	Quartet	3.7787	-3190.51656	0.53838	0.57176	0.47432	-3190.04224	21.4
	Sextet	8.7927	-3190.44151	0.53569	0.56928	0.47182	-3189.96969	66.9
2	**CSS**	**-**	**-3190.34090**	**0.54215**	**0.57476**	**0.48024**	**-3189.86066**	**0.0**
	Triplet	2.0432	-3190.33215	0.54075	0.57344	0.47857	-3189.85358	4.4
	Quintet	6.0528	-3190.29300	0.53882	0.57203	0.47557	-3189.81743	27.1
3	**Doublet**	**0.7579**	**-3190.06432**	**0.54065**	**0.57340**	**0.47885**	**-3189.58547**	**0.0**
	Quartet	3.7870	-3190.04861	0.53939	0.57217	0.47748	-3189.57113	9.0
	Sextet	8.7932	-3190.01577	0.53765	0.57093	0.47480	-3189.54097	27.9
**M06-L/def2-TZVP/disp3**								
-2	**CSS**	**-**	**-3191.64475**	**0.53385**	**0.56692**	**0.47261**	**-3191.17214**	**0.0**
	Triplet	2.0177	-3191.63369	0.53328	0.56645	0.47155	-3191.16214	6.3
	Quintet	6.0246	-3191.58574	0.53172	0.56529	0.46945	-3191.11629	35.0
-1	**Doublet**	**0.7644**	**-3191.58527**	**0.53815**	**0.57101**	**0.4764**	**-3191.10887**	**0.0**
	Quartet	3.7709	-3191.53614	0.53584	0.56913	0.47360	-3191.06254	29.1
	Sextet	8.7789	-3191.48736	0.53485	0.56830	0.47249	-3191.01487	59.0
0	**CSS**	**-**	**-3191.48936**	**0.54261**	**0.57515**	**0.48096**	**-3191.00840**	**0.0**
	Triplet	2.0140	-3191.45495	0.54000	0.57296	0.47779	-3190.97716	19.6
	Quintet	6.0243	-3191.40144	0.53822	0.57162	0.47467	-3190.92677	51.2
1	**Doublet**	**0.7568**	**-3191.32117**	**0.54366**	**0.57619**	**0.48183**	**-3190.83934**	**0.0**
	Quartet	3.7829	-3191.28400	0.54133	0.57436	0.47860	-3190.80540	21.3
	Sextet	8.7854	-3191.21261	0.53883	0.57211	0.47577	-3190.73684	64.3
2	**CSS**	**-**	**-3191.10955**	**0.54423**	**0.57676**	**0.48267**	**-3190.62688**	**0.0**
	Triplet	2.0250	-3191.10150	0.54331	0.57586	0.48142	-3190.62008	4.3
	Quintet	6.0338	-3191.05315	0.54098	0.57411	0.47804	-3190.57511	32.5
3	**Doublet**	**0.7562**	**-3190.83821**	**0.54142**	**0.57423**	**0.48005**	**-3190.35816**	**0.0**
	Quartet	3.7725	-3190.82757	0.54137	0.57404	0.48010	-3190.34747	6.7
	Sextet	8.7844	-3190.78830	0.53972	0.57286	0.47759	-3190.31071	29.8

Single-point COSMO calculations (MeCN)

Table S15. Calculated <s²> values, spin states and energies (in Hartree) of [Ni(H_2PhenTAA)] (single-point COSMO (MeCN)).

Charge	Spin state	<s²>	SCF (Hartree)	ZPE correction (Hartree)	Enthalpy correction (Hartree)	Entropy correction (Hartree)	ΔG°$_{298K}$ (Hartree)	Relative energies (kcal·mol^{-1})
**[Ni(H_2PhenTAA)] (COSMO MeCN (ε = 38.8)**								
**BP86/def2-TZVP/disp3**								
-2	**CSS**	**-**	**-2730.12628**	**0.35751**	**0.38258**	**0.30661**	**-2729.81967**	**0.0**
	Triplet	2.0093	-2730.11239	0.35661	0.38194	0.30522	-2729.80717	7.8
	Quintet	6.0148	-2730.05766	0.35350	0.37967	0.30037	-2729.75729	39.1
-1	**Doublet**	**0.7576**	**-2730.03472**	**0.36267**	**0.38714**	**0.31194**	**-2729.72278**	**0.0**
	Quartet	3.7590	-2729.98211	0.36004	0.38514	0.30831	-2729.67380	30.7
	Sextet	8.7619	-2729.92571	0.35817	0.38360	0.30576	-2729.61995	64.5
0	**CSS**	**-**	**-2729.92129**	**0.36741**	**0.39143**	**0.31686**	**-2729.60443**	**0.0**
	Triplet	2.0053	-2729.88589	0.36528	0.38965	0.31435	-2729.57154	20.6
	Quintet	6.0108	-2729.82857	0.36251	0.38757	0.31019	-2729.51838	54.0
1	**Doublet**	**0.7530**	**-2729.75004**	**0.36892**	**0.39292**	**0.31813**	**-2729.43191**	**0.0**
	Quartet	3.7658	-2729.70824	0.36674	0.39113	0.31553	-2729.39271	24.6
	Sextet	8.7644	-2729.63297	0.36280	0.38800	0.30996	-2729.32301	68.3
2	**CSS**	**-**	**-2729.55357**	**0.36979**	**0.39381**	**0.31900**	**-2729.23457**	**0.0**
	Triplet	2.0115	-2729.54466	0.36908	0.39306	0.31832	-2729.22634	5.2
	Quintet	6.0133	-2729.48608	0.36614	0.39077	0.31452	-2729.17156	39.5
3	**Doublet**	**0.7571**	**-2729.31649**	**0.36851**	**0.39265**	**0.31762**	**-2728.99887**	**0.0**
	Quartet	3.7579	-2729.29377	0.36756	0.39184	0.31640	-2728.97737	13.5
	Sextet	8.7649	-2729.23478	0.36502	0.38994	0.31298	-2728.92180	48.4
**B3-LYP/def2-TZVP/disp3**								
-2	CSS	-	-2728.92069	0.36763	0.39189	0.31739	-2728.60330	4.4
	**OSS[(a)]**	**0.7618**	**-2728.92372**	**0.36717**	**0.39153**	**0.31679**	**-2728.61024**	**0.0**
	Triplet	2.0300	-2728.91889	0.36707	0.39152	0.31640	-2728.60249	4.9
	Quintet	6.0348	-2728.87859	0.36425	0.38946	0.31206	-2728.56653	27.4
-1	**Doublet**	**0.7769**	**-2728.84638**	**0.37249**	**0.39621**	**0.32235**	**-2728.52403**	**0.0**
	Quartet	3.7782	-2728.80331	0.36929	0.39390	0.31744	-2728.48587	23.9
	Sextet	8.7787	-2728.75158	0.36901	0.39366	0.31715	-2728.43443	56.2
0	**CSS**	**-**	**-2728.74841**	**0.37822**	**0.40144**	**0.32839**	**-2728.42002**	**0.0**
	Triplet	2.0222	-2728.70121	0.37522	0.39886	0.32499	-2728.37622	27.5
	Quintet	6.0228	-2728.66051	0.37241	0.39685	0.32050	-2728.34001	50.2
1	**Doublet**	**0.7596**	**-2728.57245**	**0.37978**	**0.40300**	**0.32968**	**-2728.24277**	**0.0**
	Quartet	3.7743	-2728.54764	0.37767	0.40145	0.32657	-2728.22107	13.6
	Sextet	8.7838	-2728.46577	0.37254	0.39710	0.32033	-2728.14544	61.1
2	**CSS**	**-**	**-2728.39074**	**0.38088**	**0.40413**	**0.33077**	**-2728.05997**	**0.0**
	Triplet	2.0401	-2728.37942	0.37957	0.40278	0.32954	-2728.04988	6.3
	Quintet	6.0505	-2728.34348	0.37679	0.40078	0.32553	-2728.01795	26.4
3	**Doublet**	**0.8069**	**-2728.15047**	**0.37907**	**0.40247**	**0.32888**	**-2727.82159**	**0.0**
	Quartet	3.7790	-2728.12966	0.37784	0.40135	0.32737	-2727.80229	12.1
	Sextet	8.7853	-2728.09669	0.37530	0.39951	0.32383	-2727.77286	30.6
**M06-L/def2-TZVP/disp3**								
-2	**CSS**	**-**	**-2729.48963**	**0.37139**	**0.39524**	**0.32168**	**-2729.16795**	**0.0**
	Triplet	2.0201	-2729.47933	0.37029	0.39443	0.32007	-2729.15926	5.5
	Quintet	6.0288	-2729.43624	0.36806	0.39274	0.31675	-2729.11949	30.4
-1	**Doublet**	**0.7581**	**-2729.13827**	**0.37601**	**0.39941**	**0.32636**	**-2728.81191**	**0.0**
	Quartet	3.7767	-2729.10314	0.37342	0.39748	0.32258	-2728.78056	19.7
	Sextet	8.7694	-2729.03266	0.37215	0.39637	0.32117	-2728.71149	63.0
0	**CSS**	**-**	**-2729.30868**	**0.38053**	**0.40357**	**0.33101**	**-2728.97767**	**0.0**
	Triplet	2.0137	-2729.26704	0.37760	0.40114	0.32754	-2728.93950	24.0
	Quintet	6.0156	-2729.22423	0.37603	0.39988	0.32538	-2728.89885	49.5
1	**Doublet**	**0.7564**	**-2729.14456**	**0.38148**	**0.40455**	**0.33175**	**-2728.81281**	**0.0**
	Quartet	3.7778	-2729.10821	0.37919	0.40280	0.32860	-2728.77961	20.8
	Sextet	8.7767	-2729.03290	0.37524	0.39933	0.32406	-2728.70884	65.2
2	**CSS**	**-**	**-2728.95394**	**0.38193**	**0.40506**	**0.33220**	**-2728.62174**	**0.0**
	Triplet	2.0231	-2728.94498	0.38101	0.40409	0.33129	-2728.61369	5.1
	Quintet	6.0373	-2728.90555	0.37918	0.40275	0.32872	-2728.57683	28.2
3	**Doublet**	**0.7656**	**-2728.72074**	**0.37995**	**0.40324**	**0.33005**	**-2728.39069**	**0.0**
	Quartet	3.7673	-2728.69895	0.37911	0.40248	0.32900	-2728.36995	13.0
	Sextet	8.7784	-2728.66229	0.37613	0.40021	0.32474	-2728.33755	33.3

Table S16. Calculated <s^2> values, spin states and energies (in Hartree) of [Ni(Me_2PhenTAA)] (single-point COSMO (MeCN)).

**Charge**	**Spin state**	**<s^2>**	**SCF (Hartree)**	**ZPE correction (Hartree)**	**Enthalpy correction (Hartree)**	**Entropy correction (Hartree)**	**ΔG°$_{298K}$ (Hartree)**	**Relative energies (kcal·mol^{-1})**
**[Ni(Me_2PhenTAA)] (COSMO MeCN (ε = 38.8)**								
**BP86/def2-TZVP/disp3**								
-2	**CSS**	**-**	**-2808.77655**	**0.41218**	**0.43997**	**0.35881**	**-2808.41774**	**0.0**
	Triplet	2.0087	-2808.75774	0.41108	0.43917	0.35720	-2808.40054	10.8
	Quintet	6.0149	-2808.69774	0.40879	0.43744	0.35370	-2808.34404	46.2
-1	**Doublet**	**0.7563**	**-2808.68346**	**0.41742**	**0.44467**	**0.36416**	**-2808.31930**	**0.0**
	Quartet	3.7594	-2808.63333	0.41490	0.44271	0.36080	-2808.27253	29.3
	Sextet	8.7624	-2808.56750	0.41297	0.44109	0.35811	-2808.20939	69.0
0	**CSS**	**-**	**-2808.56858**	**0.42207**	**0.44890**	**0.36911**	**-2808.19947**	**0.0**
	Triplet	2.0060	-2808.54001	0.42010	0.44726	0.36661	-2808.17340	16.4
	Quintet	6.0126	-2808.47591	0.41668	0.44447	0.36207	-2808.11384	53.7
1	**Doublet**	**0.7535**	**-2808.40222**	**0.42355**	**0.45030**	**0.37046**	**-2808.03176**	**0.0**
	Quartet	3.7653	-2808.36544	0.42159	0.44869	0.36794	-2807.99750	21.5
	Sextet	8.7662	-2808.29107	0.41789	0.44577	0.36310	-2807.92797	65.1
2	**CSS**	**-**	**-2808.20966**	**0.42440**	**0.45112**	**0.37134**	**-2807.83832**	**0.0**
	Triplet	2.0127	-2808.20120	0.42367	0.45040	0.37056	-2807.83064	4.8
	Quintet	6.0130	-2808.15026	0.42079	0.44821	0.36658	-2807.78368	34.3
3	**Doublet**	**0.7598**	**-2807.97644**	**0.42304**	**0.44991**	**0.36982**	**-2807.60662**	**0.0**
	Quartet	3.7599	-2807.95622	0.42176	0.44884	0.36824	-2807.58798	11.7
	Sextet	8.7665	-2807.90133	0.41927	0.44703	0.36457	-2807.53676	43.8
**B3-LYP/def2-TZVP/disp3**								
-2	**CSS**	**-**	**-2807.50773**	**0.42395**	**0.45080**	**0.37139**	**-2807.13634**	**0.0**
	Triplet	2.0274	-2807.50100	0.42316	0.45028	0.37008	-2807.13092	3.4
	Quintet	6.0332	-2807.45496	0.42137	0.44897	0.36718	-2807.08778	30.5
-1	**Doublet**	**0.7734**	**-2807.43269**	**0.42900**	**0.45540**	**0.37645**	**-2807.05624**	**0.0**
	Quartet	3.7746	-2807.37788	0.42707	0.45387	0.37398	-2807.00390	32.8
	Sextet	8.7814	-2807.33101	0.42554	0.45278	0.37138	-2806.95963	60.6
0	**CSS**	**-**	**-2807.33446**	**0.43442**	**0.46036**	**0.38223**	**-2806.95223**	**0.0**
	Triplet	2.0159	-2807.29454	0.43223	0.45875	0.37888	-2806.91566	22.9
	Quintet	6.0277	-2807.24336	0.43003	0.45679	0.37628	-2806.86708	53.4
1	**Doublet**	**0.7606**	**-2807.17209**	**0.43599**	**0.46184**	**0.38369**	**-2806.78840**	**0.0**
	Quartet	3.7788	-2807.13707	0.43426	0.46057	0.38094	-2806.75613	20.2
	Sextet	8.7993	-2807.06173	0.43029	0.45739	0.37589	-2806.68584	64.4
2	**CSS**	**-**	**-2806.98242**	**0.43710**	**0.46294**	**0.38486**	**-2806.59756**	**0.0**
	Triplet	2.0415	-2806.97367	0.43576	0.46165	0.38338	-2806.59029	4.6
	Quintet	6.0533	-2806.93739	0.43388	0.46024	0.38063	-2806.55676	25.6
3	**Doublet**	**0.8102**	**-2806.74705**	**0.43535**	**0.46136**	**0.38289**	**-2806.36416**	**0.0**
	Quartet	3.7824	-2806.72918	0.43368	0.45990	0.38094	-2806.34824	10.0
	Sextet	8.7899	-2806.69632	0.43191	0.45863	0.37828	-2806.31804	28.9
**M06-L/def2-TZVP/disp3**								
-2	**CSS**	**-**	**-2808.12071**	**0.42851**	**0.45487**	**0.37661**	**-2807.74410**	**0.0**
	Triplet	2.0194	-2808.10583	0.42748	0.45412	0.37512	-2807.73071	8.4
	Quintet	6.0293	-2808.05747	0.42588	0.45295	0.37246	-2807.68501	37.1
-1	**Doublet**	**0.7640**	**-2808.04092**	**0.43339**	**0.45934**	**0.38156**	**-2807.65936**	**0.0**
	Quartet	3.7767	-2807.99017	0.43074	0.45734	0.37763	-2807.61254	29.4
	Sextet	8.7794	-2807.93566	0.42918	0.45608	0.37562	-2807.56004	62.3
0	**CSS**	**-**	**-2807.93733**	**0.43765**	**0.46330**	**0.38603**	**-2807.55130**	**0.0**
	Triplet	2.0174	-2807.90517	0.43508	0.46118	0.38271	-2807.52246	18.1
	Quintet	6.0259	-2807.84900	0.43306	0.45953	0.37983	-2807.46917	51.5
1	**Doublet**	**0.7573**	**-2807.77746**	**0.43866**	**0.46427**	**0.38690**	**-2807.39056**	**0.0**
	Quartet	3.7821	-2807.74434	0.43661	0.46266	0.38405	-2807.36029	19.0
	Sextet	8.7865	-2807.66804	0.43308	0.45970	0.37975	-2807.28829	64.2
2	**CSS**	**-**	**-2807.59035**	**0.43916**	**0.46479**	**0.38741**	**-2807.20294**	**0.0**
	Triplet	2.0248	-2807.58252	0.43820	0.46384	0.38638	-2807.19614	4.3
	Quintet	6.0393	-2807.54439	0.43657	0.46261	0.38405	-2807.16034	26.7
3	**Doublet**	**0.7689**	**-2807.36112**	**0.43719**	**0.46299**	**0.38524**	**-2806.97588**	**0.0**
	Quartet	3.7702	-2807.34214	0.43604	0.46195	0.38396	-2806.95818	11.1
	Sextet	8.7819	-2807.30739	0.43455	0.46089	0.38173	-2806.92566	31.5

Table S17. Calculated <s²> values, spin states and energies (in Hartree) of [Ni(Ph_2PhenTAA)] (single-point COSMO (MeCN)).

Charge	Spin state	<s²>	SCF (Hartree)	ZPE correction (Hartree)	Enthalpy correction (Hartree)	Entropy correction (Hartree)	ΔG°$_{298K}$ (Hartree)	Relative energies (kcal·mol^{-1})
**[Ni(Ph_2PhenTAA)] (COSMO MeCN (ε = 38.8)**								
**BP86/def2-TZVP/disp3**								
-2	**CSS**	**-**	**-3192.42903**	**0.51440**	**0.54908**	**0.45113**	**-3191.97790**	**0.0**
	Triplet	2.0079	-3192.41387	0.51383	0.54859	0.45043	-3191.96344	9.1
	Quintet	6.0138	-3192.35527	0.51120	0.54665	0.44638	-3191.90889	43.3
-1	**Doublet**	**0.7564**	**-3192.33352**	**0.51956**	**0.55378**	**0.45639**	**-3191.87713**	**0.0**
	Quartet	3.7584	-3192.28529	0.51750	0.55209	0.45365	-3191.83164	28.5
	Sextet	8.7623	-3192.22160	0.51505	0.55015	0.44956	-3191.77204	65.9
0	**CSS**	**-**	**-3192.21779**	**0.52411**	**0.55796**	**0.46097**	**-3191.75682**	**0.0**
	Triplet	2.0052	-3192.18692	0.52225	0.55639	0.45868	-3191.72824	17.9
	Quintet	6.0129	-3192.12425	0.51917	0.55382	0.45448	-3191.66977	54.6
1	**Doublet**	**0.7533**	**-3192.04953**	**0.52575**	**0.55952**	**0.46249**	**-3191.58704**	**0.0**
	Quartet	3.7658	-3192.00896	0.52368	0.55785	0.45960	-3191.54936	23.6
	Sextet	8.7667	-3191.93776	0.52083	0.55539	0.45628	-3191.48148	66.2
2	**CSS**	**-**	**-3191.85574**	**0.52671**	**0.56044**	**0.46371**	**-3191.39203**	**0.0**
	Triplet	2.0126	-3191.84683	0.52582	0.55957	0.46263	-3191.38420	4.9
	Quintet	6.0133	-3191.79335	0.52344	0.55769	0.45951	-3191.33384	36.5
3	**Doublet**	**0.7572**	**-3191.62097**	**0.52406**	**0.55814**	**0.46068**	**-3191.16029**	**0.0**
	Quartet	3.7609	-3191.60559	0.52452	0.55843	0.46147	-3191.14412	10.1
	Sextet	8.7676	-3191.54615	0.52152	0.55620	0.45673	-3191.08942	44.5
**B3-LYP/def2-TZVP/disp3**								
-2	**CSS**	**-**	**-3190.90566**	**0.52884**	**0.56241**	**0.46650**	**-3190.43916**	**0.0**
	Triplet	2.0263	-3190.90164	0.52870	0.56227	0.46632	-3190.43532	2.4
	Quintet	6.0285	-3190.85464	0.52738	0.56134	0.46146	-3190.39318	28.9
-1	**Doublet**	**0.7740**	**-3190.82836**	**0.53377**	**0.56695**	**0.47130**	**-3190.35706**	**0.0**
	Quartet	3.7749	-3190.77555	0.53246	0.56585	0.46981	-3190.30574	32.2
	Sextet	8.7823	-3190.73079	0.53083	0.56464	0.46735	-3190.26344	58.7
0	**CSS**	**-**	**-3190.72918**	**0.53919**	**0.57194**	**0.47711**	**-3190.25207**	**0.0**
	Triplet	2.0210	-3190.68467	0.53660	0.56972	0.47367	-3190.21100	25.8
	Quintet	6.0252	-3190.63789	0.53495	0.56858	0.47086	-3190.16703	53.4
1	**Doublet**	**0.7604**	**-3190.56489**	**0.54092**	**0.57356**	**0.47888**	**-3190.08601**	**0.0**
	Quartet	3.7860	-3190.52631	0.53838	0.57176	0.47432	-3190.05199	21.3
	Sextet	8.7927	-3190.45152	0.53569	0.56928	0.47182	-3189.97970	66.7
2	**CSS**	**-**	**-3190.37378**	**0.54215**	**0.57476**	**0.48024**	**-3189.89354**	**0.0**
	Triplet	2.0433	-3190.36473	0.54075	0.57344	0.47857	-3189.88616	4.6
	Quintet	6.0530	-3190.32622	0.53882	0.57203	0.47557	-3189.85065	26.9
3	**Doublet**	**0.7579**	**-3190.13460**	**0.54065**	**0.57340**	**0.47885**	**-3189.65575**	**0.0**
	Quartet	3.7867	-3190.11835	0.53939	0.57217	0.47748	-3189.64087	9.3
	Sextet	8.7928	-3190.08629	0.53765	0.57093	0.47480	-3189.61149	27.8
**M06-L/def2-TZVP/disp3**								
-2	**CSS**	**-**	**-3191.68069**	**0.53385**	**0.56692**	**0.47261**	**-3191.20808**	**0.0**
	Triplet	2.0180	-3191.66948	0.53328	0.56645	0.47155	-3191.19793	6.4
	Quintet	6.0254	-3191.62069	0.53172	0.56529	0.46945	-3191.15124	35.7
-1	**Doublet**	**0.7643**	**-3191.59706**	**0.53815**	**0.57101**	**0.4764**	**-3191.12066**	**0.0**
	Quartet	3.7710	-3191.54731	0.53584	0.56913	0.47360	-3191.07371	29.5
	Sextet	8.7791	-3191.49830	0.53485	0.56830	0.47249	-3191.02581	59.5
0	**CSS**	**-**	**-3191.49215**	**0.54261**	**0.57515**	**0.48096**	**-3191.01119**	**0.0**
	Triplet	2.0141	-3191.45731	0.54000	0.57296	0.47779	-3190.97952	19.9
	Quintet	6.0244	-3191.40379	0.53822	0.57162	0.47467	-3190.92912	51.5
1	**Doublet**	**0.7567**	**-3191.33026**	**0.54366**	**0.57619**	**0.48183**	**-3190.84843**	**0.0**
	Quartet	3.7826	-3191.29319	0.54133	0.57436	0.47860	-3190.81459	21.2
	Sextet	8.7854	-3191.22192	0.53883	0.57211	0.47577	-3190.74615	64.2
2	**CSS**	**-**	**-3191.14177**	**0.54423**	**0.57676**	**0.48267**	**-3190.65910**	**0.0**
	Triplet	2.0248	-3191.13363	0.54331	0.57586	0.48142	-3190.65221	4.3
	Quintet	6.0342	-3191.08532	0.54098	0.57411	0.47804	-3190.60728	32.5
3	**Doublet**	**0.7564**	**-3190.90808**	**0.54142**	**0.57423**	**0.48005**	**-3190.42803**	**0.0**
	Quartet	3.7724	-3190.89721	0.54137	0.57404	0.48010	-3190.41711	6.9
	Sextet	8.7843	-3190.85853	0.53972	0.57286	0.47759	-3190.38094	29.5

Time-dependent Density Functional Theory Calculations

TD–DFT calculations were performed according to the procedure described in the general considerations. All spin states were calculated at the B3LYP/def2-TZVPP level of theory and compared with experimental UV/Vis spectra for accuracy. In line with experimental findings and DFT calculations, all experimental spectra matched best with the low-spin TD–DFT calculated spectra. Additionally, the most relevant excitation states were analyzed and the orbital pair with the greatest contribution is highlighted. MO schemes for the neutral, archetypical [Ni(R_2PhenTAA)] complexes are shown as well (vide infra).

As shown in Figure S107–Figure S109, the frontier orbitals of [Ni(R_2PhenTAA)] complexes are dominated by ligand-based orbitals and many are heavily correlated with the metal d-orbitals of the central Ni atom. In all complexes, the HOMO and LUMO are ligand based. The LUMO and LUMO+1 are centered on the imine moieties in combination with the adjacent phenyl rings. Interestingly, the LUMO exhibits a quinoidal structure for the *o*-diiminophenylene ring as opposed to a more localized orbital on the benzene ring. In contrast, the HOMO and HOMO-1 are centered on the diaryl-*o*-phenylene diamine moiety. This is in accordance with the experimentally found redox-active nature of the ligand where upon reduction, the imine moieties are reduced and upon oxidation, the *o*-phenylene diamine moiety is oxidized (vide supra). The separation of the donor- and acceptor moieties is further underscored by the separation of these two parts in the deeper lying orbitals (<HOMO-1). Furthermore, the flanking phenyl rings clearly play a role in delocalizing the electron density in the respective donor/acceptor moieties as both are conjugated to either the diaryl-*o*-phenylene diamine- or the diiminophenylene moiety.

The metal d-orbitals are arranged in a fairly expected manner for a square planar N_4-ligand.[24] The highest, most antibonding orbital is the Ni(d_{x2-y2}) and the least antibonding is the Ni(d_{xy}). Due to the strong π-donating properties of the ligand, the Ni(d_{z2}) is the second highest in energy. Since the ligand is asymmetric, the Ni(d_{xz}) and Ni(d_{yz}) are not degenerate with the latter being the lower in energy.

For the [Ni(H_2PhenTAA)] (**4a**) and [Ni(Me_2PhenTAA)] (**4b**) complexes, there is an clear additional asymmetry for the σ-bonding orbitals of the nitrogen donors. The N(amide)$_2$-Ni(d_{x2-y2}) bonding orbital is at a much lower energy than the N(imino)$_2$-Ni(d_{x2-y2}) orbital. The negative charge of the diaryl-*o*-phenylene diamine moiety makes it a stronger donor than the neutral imine nitrogen atoms. However, for [Ni(Ph_2PhenTAA)] (**4c**), this is not the case. Both bonding orbitals are still split due to the asymmetry of the ligand, but are comparable in energy for this complex. The addition of an extra substituent on the imine carbon changes the geometry from a half-saddle to a fully saddled complex, yet this does not change the bonding situation for [Ni(Me_2PhenTAA)] (**4b**). Therefore, this effect must be due to delocalization of the additional phenyl rings that stabilize the negative charge away from the diaryl-*o*-phenylene diamine moiety.

[Ni(H_2PhenTAA)] (4a) (CSS):

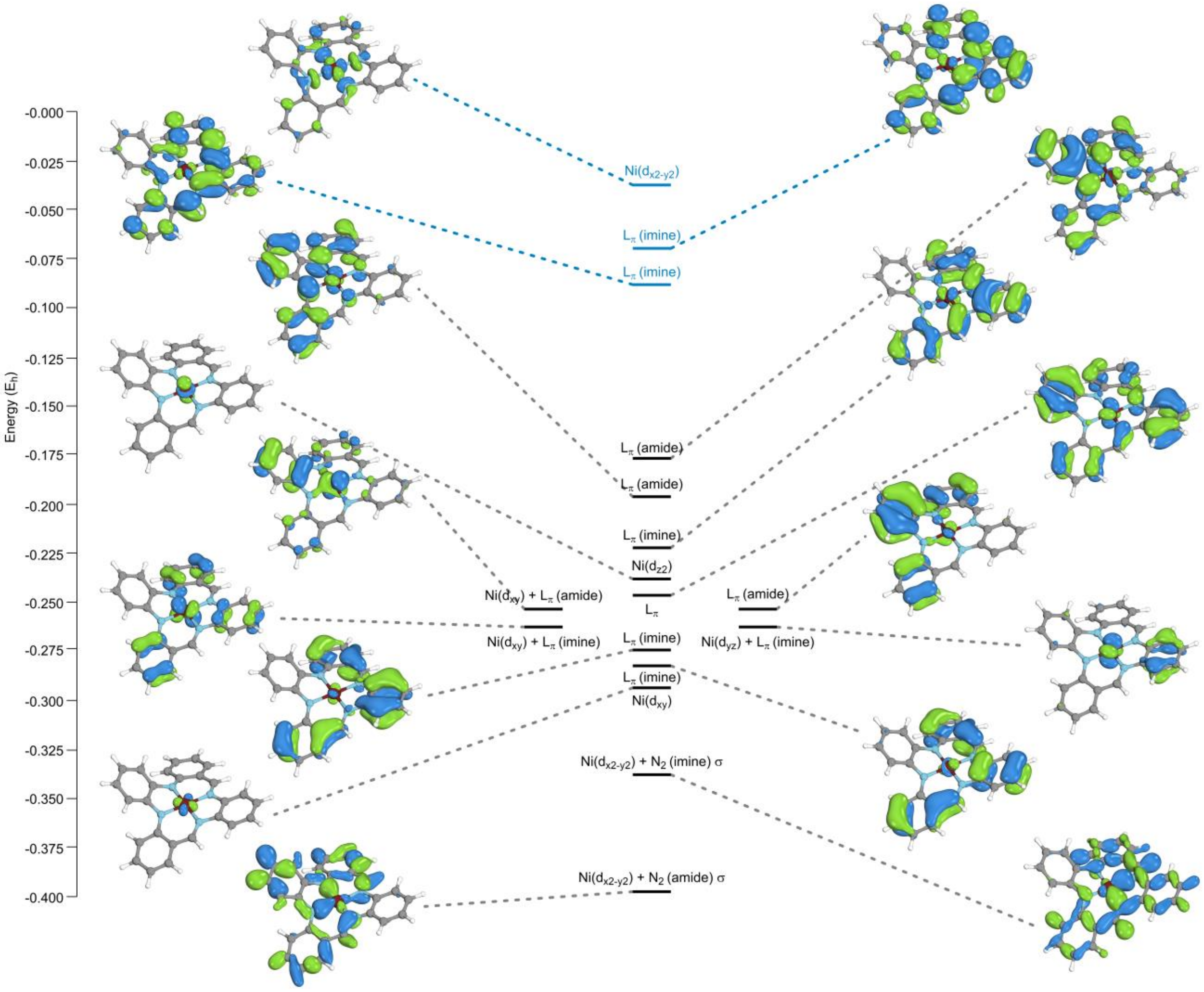

Figure S107. MO diagram of the frontier orbitals of [Ni(H_2PhenTAA)] (**4a**)at the B3LYP/def2-TZVPP/CPCMC (CH_2Cl_2) level of theory (TD–DFT). Filled orbitals displayed in black; empty orbitals in blue.

[Ni(Me$_2$PhenTAA)] (4b) (CSS):

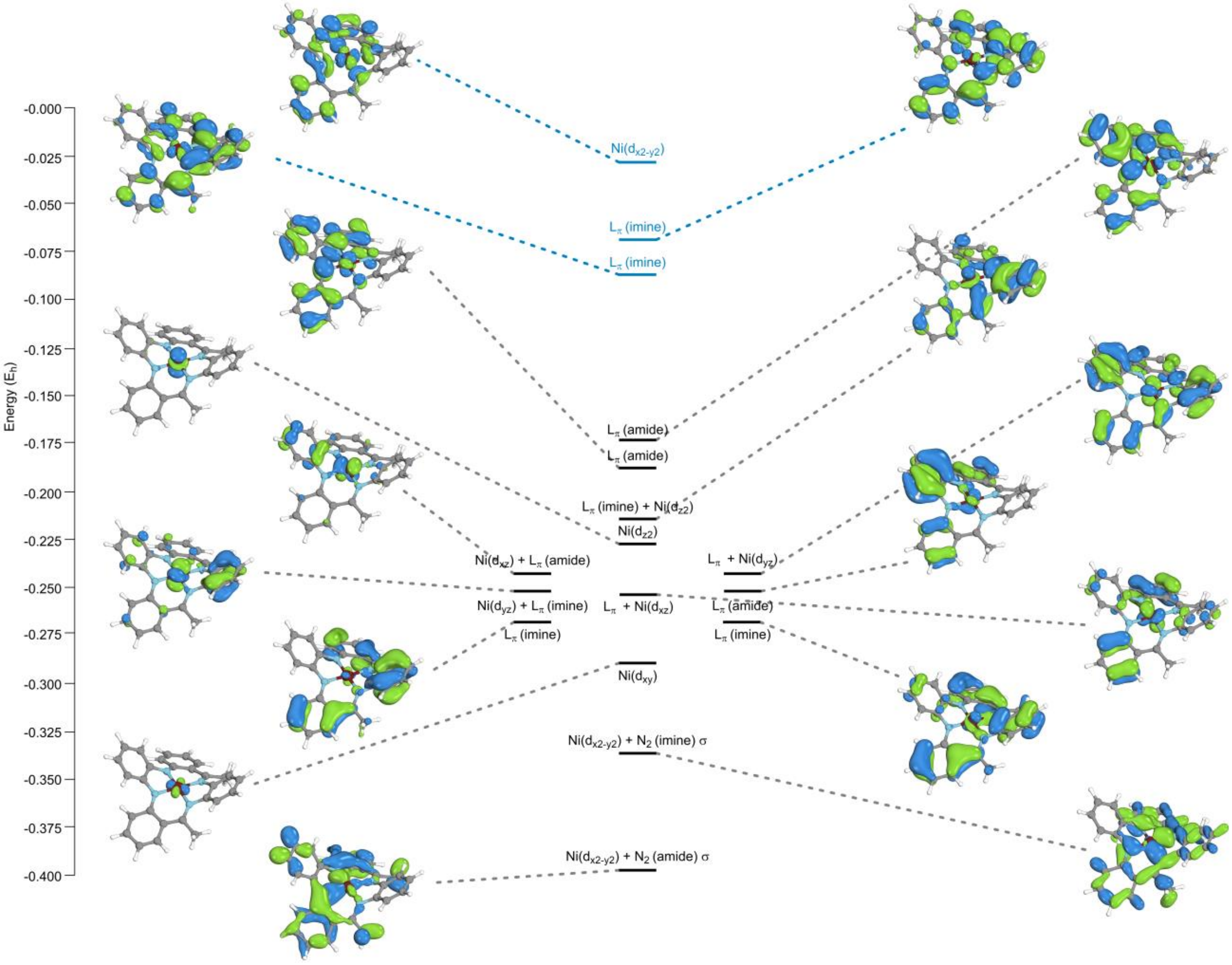

Figure S108. MO diagram of the frontier orbitals of [Ni(Me$_2$PhenTAA)] (**4b**)at the B3LYP/def2-TZVPP/CPCMC (CH_2Cl_2) level of theory (TD–DFT). Filled orbitals displayed in black; empty orbitals in blue.

[Ni(Ph_2PhenTAA)] (4c) (CSS):

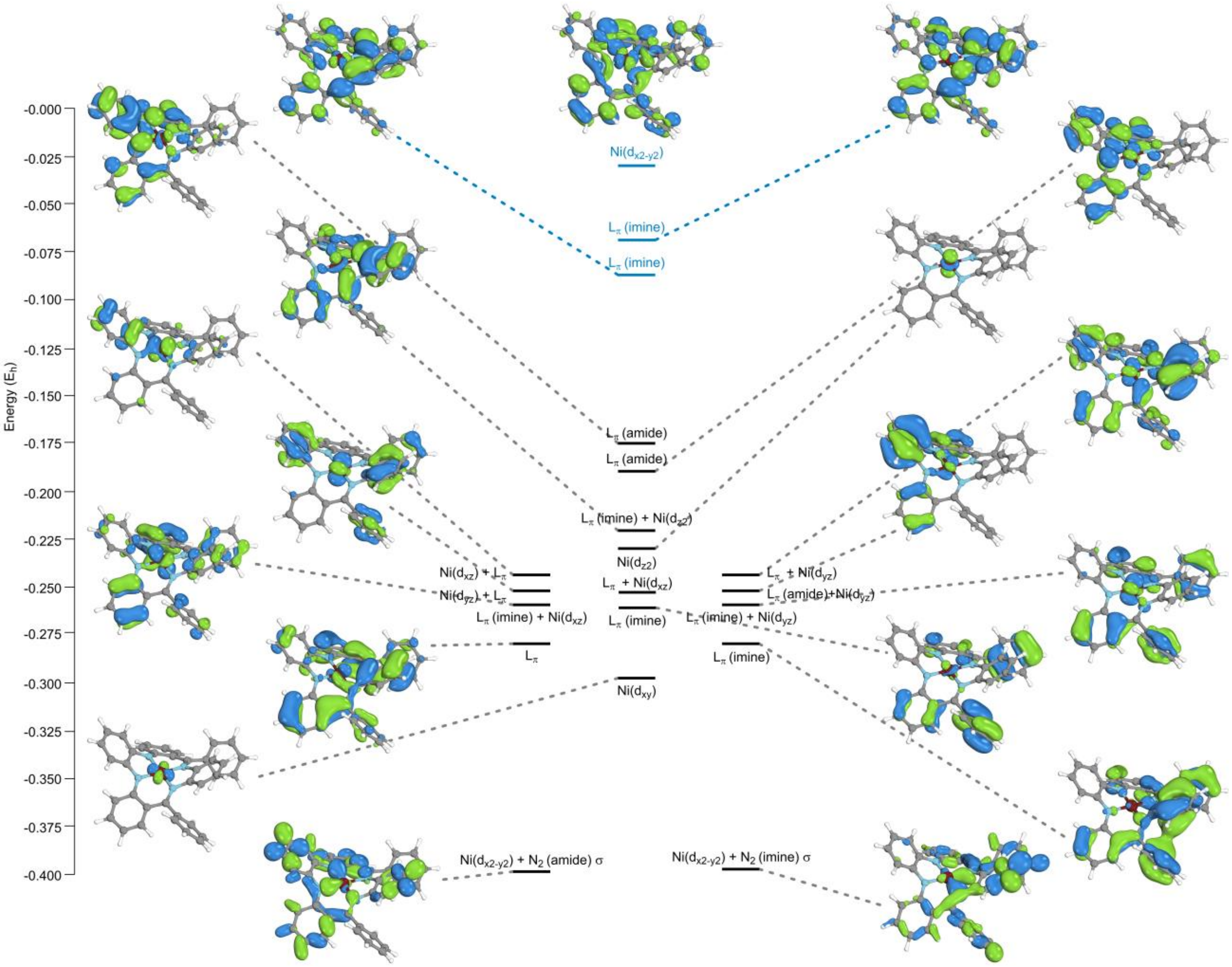

Figure S109. MO diagram of the frontier orbitals of [Ni(Ph_2PhenTAA)] (**4c**)at the B3LYP/def2-TZVPP/CPCMC (CH_2Cl_2) level of theory (TD–DFT). Filled orbitals displayed in black; empty orbitals in blue.

[Ni(H_2PhenTAA)] (4a) (CSS)

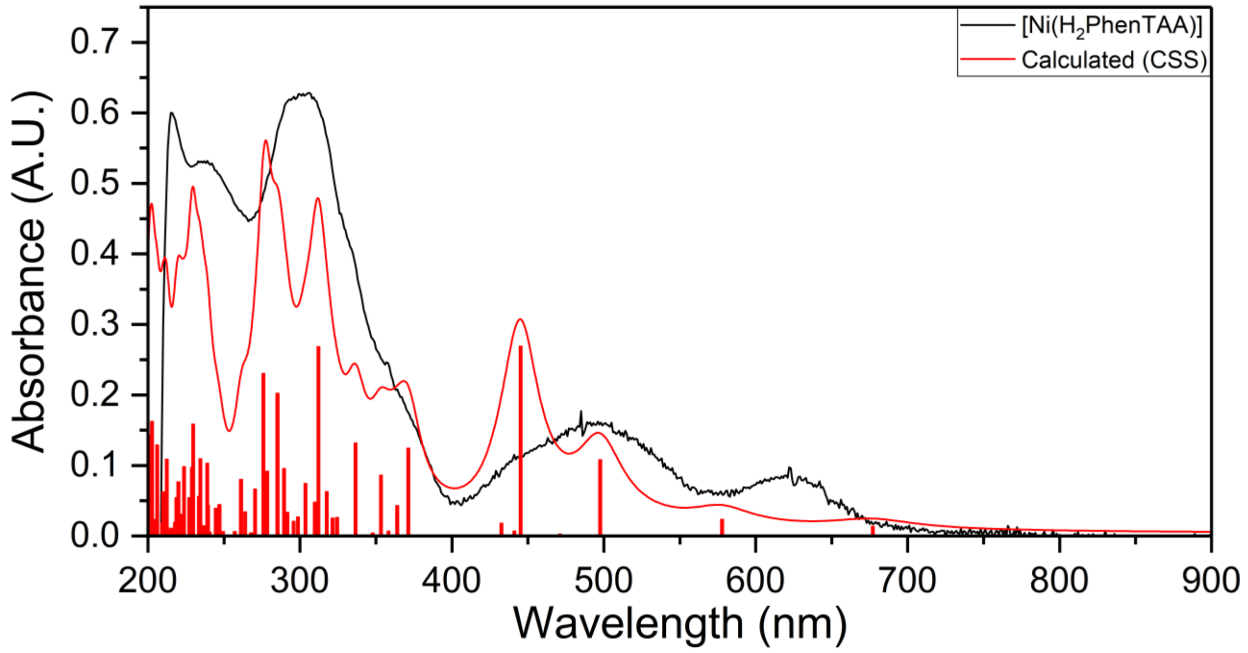

Figure S110. Experimental UV/Vis spectrum of [Ni(H_2PhenTAA)] (**4a**) in CH_2Cl_2 (black) and calculated spectrum (red; Lorentzian peak FWHM: 1500 cm^{-1}). Individual excitations illustrated in red bars.

Analysis of the most relevant excitations and their corresponding orbital pairs (

Table S19; vide infra) reveals that the UV/Vis spectrum is dominated entirely by ligand → ligand* excitations. This is due to the extensive π-conjugation of the macrocycle. However, it should be noted that in all of the involved orbitals, metal d–orbitals are mixed with the ligand's π-orbitals as shown in the MO diagrams (Figure S107–Figure S109). Nevertheless, the majority of electron density is centered on the ligand. Excitation occurs mostly from the diaryl-*o*-phenylene diamine moiety to the diimine moiety of the PhenTAA macrocycle, further illustrating the respective donating- and acceptor properties of the ligand.

Table S18. Selected states for the TD–DFT calculated absorptions of [Ni(H_2PhenTAA)] (**4a**) at the B3LYP/def2-TZVPP CPCMC (CH_2Cl_2) level of theory.

State	Energy (cm^{-1})	Wavelength (nm)	fosc	T2 (au**2)	TX (au)	TY (au)	TZ (au)
1	14771.3	677.0	0.018545582	0.41333	0.43424	0.22709	0.41617
2	17305.8	577.8	0.032046712	0.60963	-0.32449	-0.42380	0.56985
3	20094.8	497.6	0.144671395	2.37014	0.69036	0.77900	-1.13433
6	22462.8	445.2	0.359139650	5.26350	-1.07109	-1.09405	1.70860
18	32036.7	312.1	0.358289256	3.68182	-1.29895	-0.67785	-1.23898
54	43532.8	229.7	0.212127093	1.60419	0.48517	0.73428	-0.91084

Table S19. Most contributing orbital pairs to the selected states listed in Table S18 and their respective fractions.

State	Donating orbital → Accepting orbital	Fraction of total state	Donating orbital	Accepting orbital
1	HOMO → LUMO	0.95	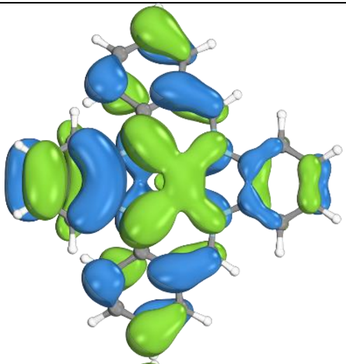	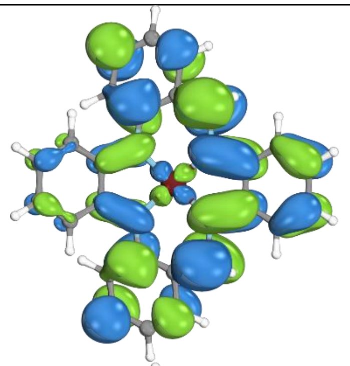
2	HOMO-1 → LUMO	0.67	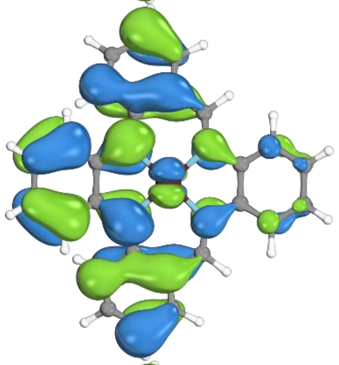	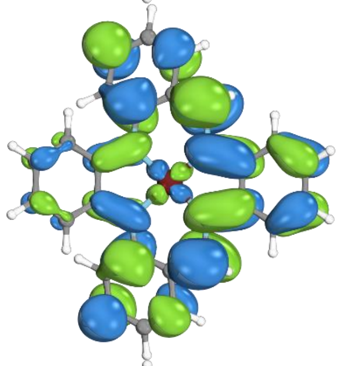
3	HOMO → LUMO+1	0.67	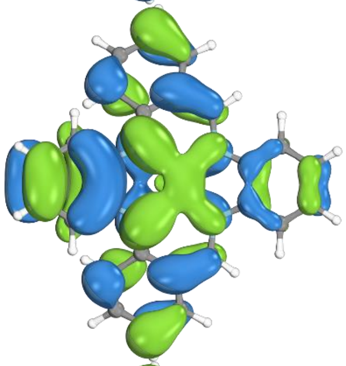	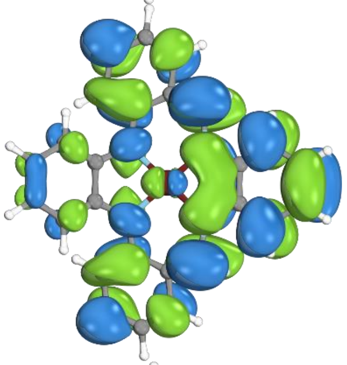
6	HOMO-1 → LUMO	0.29	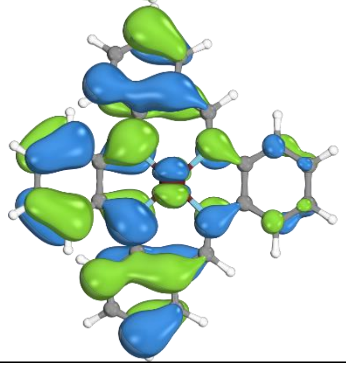	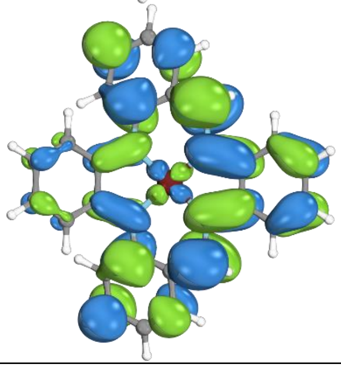

	HOMO-1 → LUMO+2	0.24	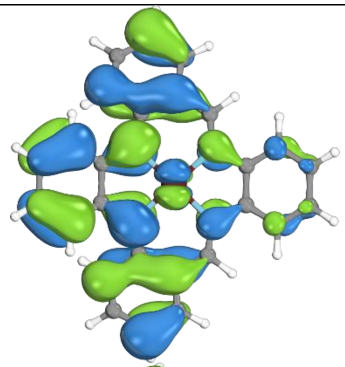	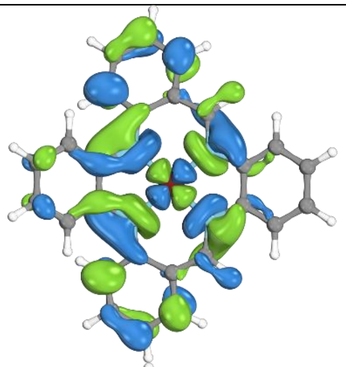
	HOMO → LUMO+1	0.24	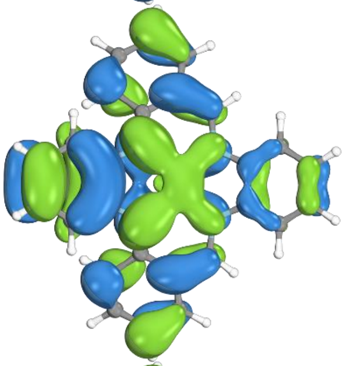	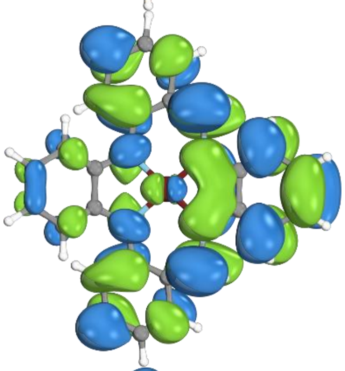
18	HOMO → LUMO+5	0.70	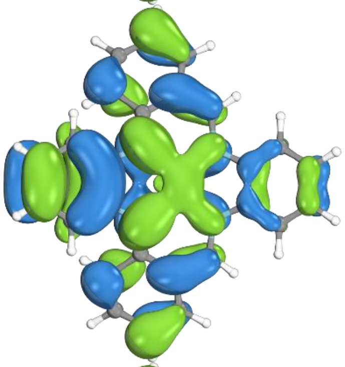	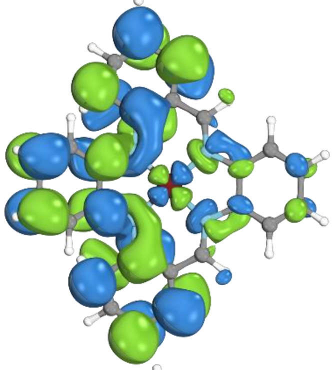
54	HOMO-1 → LUMO+8	0.71	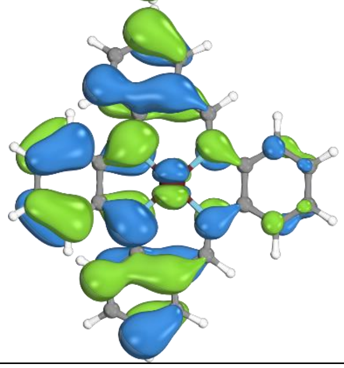	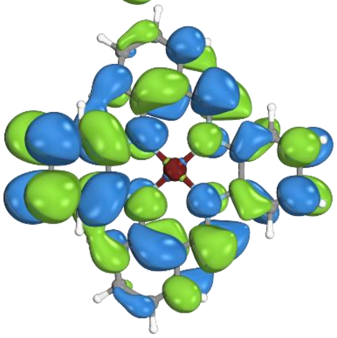

[Ni(H_2PhenTAA)]$^+$ (Doublet):

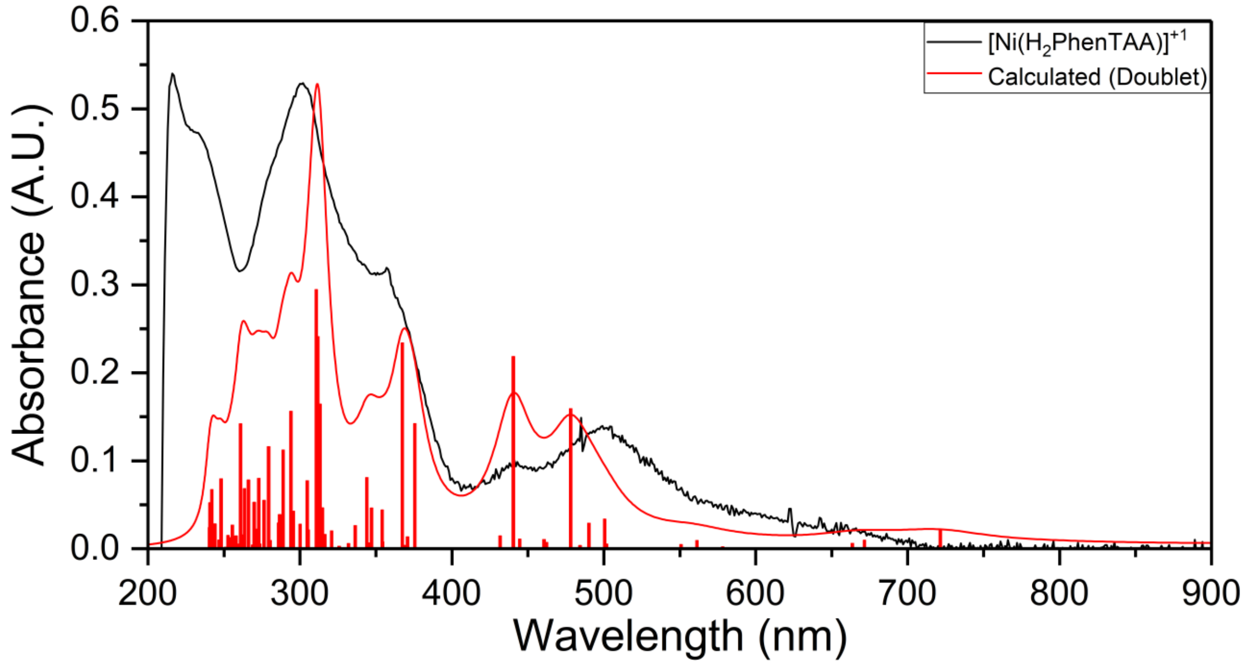

Figure S111. Experimental UV/Vis spectrum of **[**Ni(H_2PhenTAA)]$^+$ (4a^{+1}) in CH_2Cl_2 measured via spectro-electrochemistry (black) and calculated spectrum (red; Lorentzian peak FWHM: 1500 cm^{-1}). Individual excitations illustrated in red bars.

The major excitations assigned for [Ni(H_2PhenTAA)]$^+$ (**4a^{+1}**) are remarkably similar to the neutral analogue (vide infra) and are almost entirely ligand-based as well. The SOMO is centered on the donor moiety of the macrocycle, in accordance with spin density plots (vide infra) and accounts for the transition around 500 nm (SOMO → LUMO). Interestingly, the shoulder around 350-375 nm is partially built up from a $d_{xy} \rightarrow d_{x2-y2}$ transition where in the neutral complex, such a transition was absent for the major excitations.

Table S20. Selected states for the TD–DFT calculated absorptions of [Ni(H_2PhenTAA)]$^+$ **(4a^{+1})** at the B3LYP/def2-TZVPP CPCMC (CH_2Cl_2) level of theory.

State	Energy (cm^{-1})	Wavelength (nm)	fosc	T2 (au**2)	TX (au)	TY (au)	TZ (au)
16	20913.4	478.2	0.159228614	2.50653	0.59577	0.93062	-1.13381
21	22708.6	440.4	0.218466769	3.16717	0.74124	0.96386	-1.29950
26	26626.4	375.6	0.142552535	1.76254	0.89665	0.46916	0.85933
49	32183.4	310.7	0.294569773	3.01323	1.21649	-0.04828	-1.23736

Table S21. Most contributing orbital pairs to the selected states listed in Table S20 and their respective fractions. For brevity, the corresponding β-orbital pairs were omitted and their fractional contributions added to the α-orbital pairs.

State	Donating orbital → Accepting orbital	Fraction of total state	Donating orbital	Accepting orbital
16	HOMO (α) → LUMO(α)	0.58	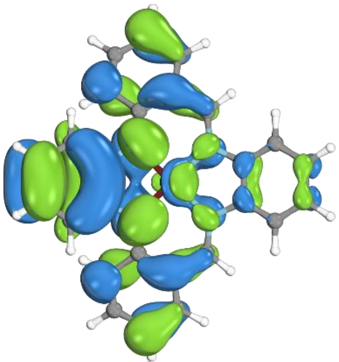	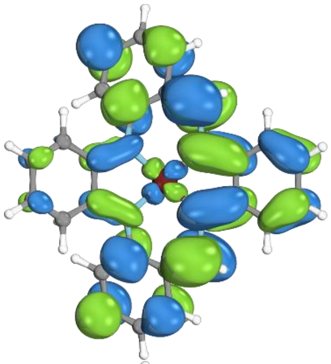
21	HOMO-1 (α) → LUMO (α)	0.53	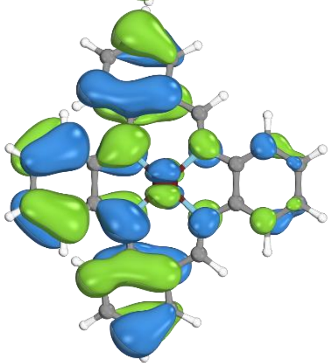	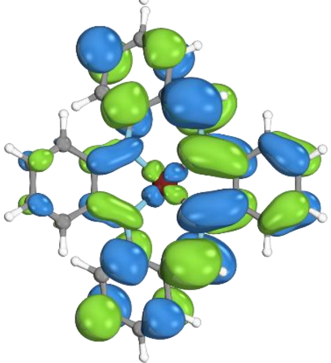
26	HOMO-1 (α) → LUMO (α)	0.41	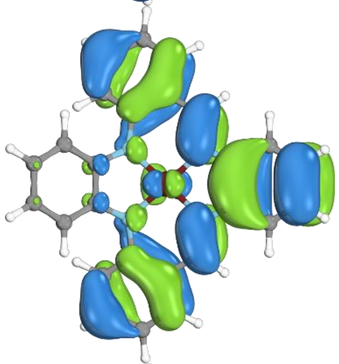	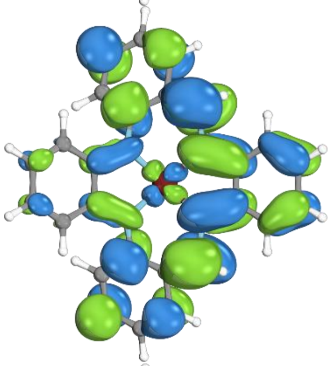
	HOMO-11 (α) → LUMO+2 (α)	0.18	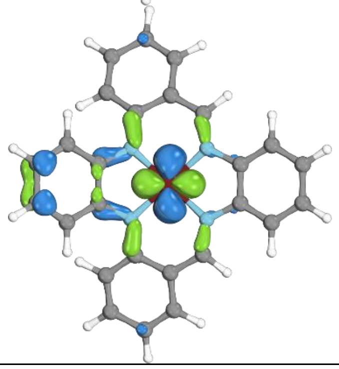	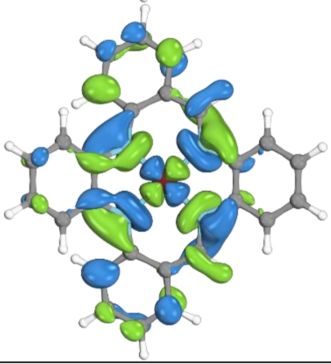

49	HOMO-2 (α) → LUMO+1 (α)	0.31	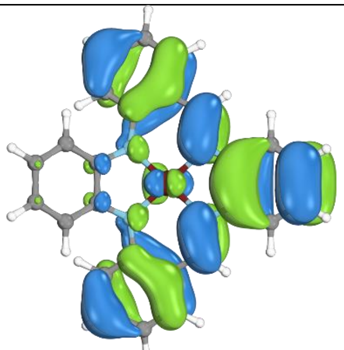	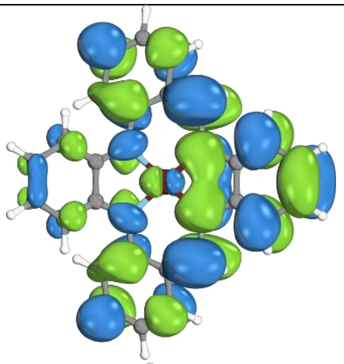
	HOMO-4 (α) → LUMO (α)	0.23	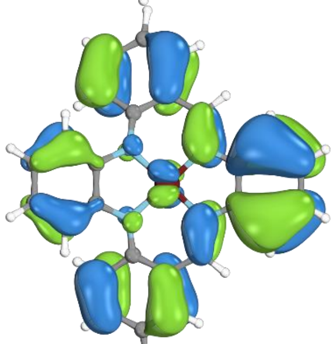	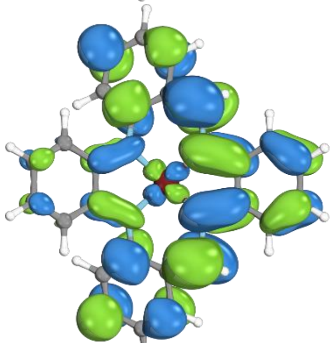
	HOMO-11 (β) → LUMO (β)	0.13	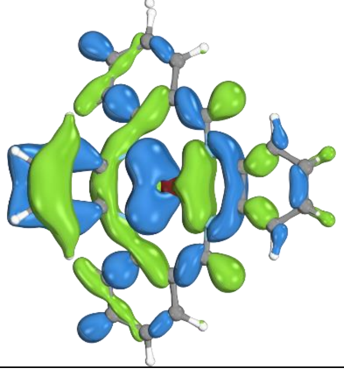	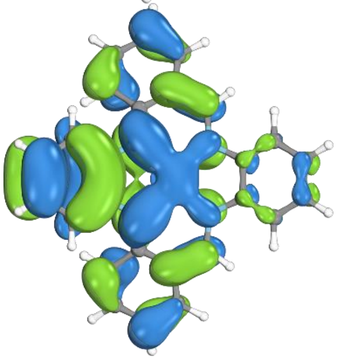

[Ni(H_2PhenTAA)]$^{2+}$ (CSS):

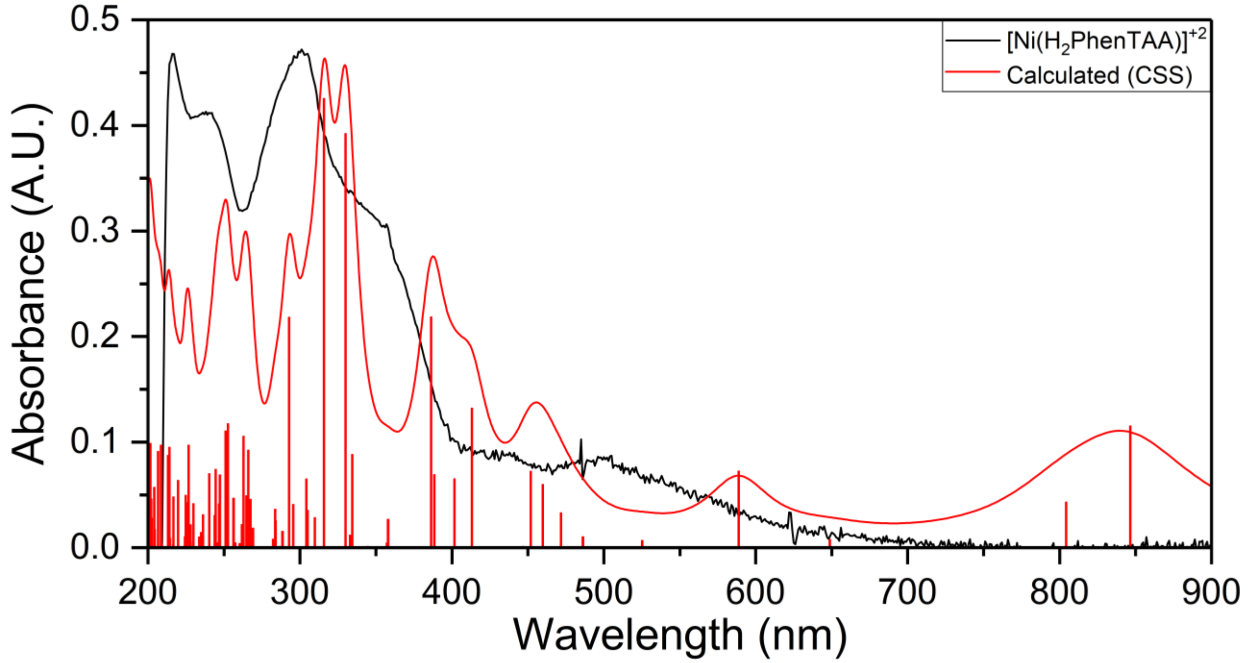

Figure S112. Experimental UV/Vis spectrum of [Ni(H_2PhenTAA)]$^{2+}$ (**4a^{+2}**) in CH_2Cl_2 measured via spectro-electrochemistry (black) and calculated spectrum (red; Lorentzian peak FWHM: 1500 cm^{-1}). Individual excitations illustrated in red bars.

Although the match between experimental data and the calculated spectrum were poorer than for the previous oxidation states, several relevant states were selected. In contrast to the previous oxidation states, state 4 is an excitation from the acceptor diimine moiety to the “donor” diaryl-*o*-phenylene diamine moiety. This is due to the *o*-phenylene diamine moiety being fully oxidized to the *o*-diiminoquinone state, which is now an acceptor moiety as well. Interestingly, the other three states are remarkably similar to the previous oxidation states and are relatively unaffected by oxidation state changes. However, the similarity of the UV/Vis spectra of **4a^{+1}** and **4a^{+2}** and the large difference in theoretical spectra could mean that the oxidation of **4a^{+1}** $\rightarrow$ **4a^{+2}** has not fully taken place yet within the allowed solvent window.

Table S22. Selected states for the TD–DFT calculated absorptions of [Ni(H_2PhenTAA)]$^{2+}$ (**4a^{+2}**) at the B3LYP/def2-TZVPP CPCMC (CH_2Cl_2) level of theory.

State	Energy (cm^{-1})	Wavelength (nm)	fosc	T2 (au**2)	TX (au)	TY (au)	TZ (au)
4	16983.6	588.8	0.087212032	1.69053	0.87803	0.45916	0.84189
10	22129.6	451.9	0.086989364	1.29410	-0.02593	-0.98883	0.56183
16	25884.4	386.3	0.262421303	3.33762	1.23436	0.64634	1.18161
23	31663.0	315.8	0.510886336	5.31188	1.49571	0.30749	-1.72632

Table S23. Most contributing orbital pairs to the selected states listed in Table S22 and their respective fractions.

State	Donating orbital → Accepting orbital	Fraction of total state	Donating orbital	Accepting orbital
4	HOMO-2 → LUMO	0.93	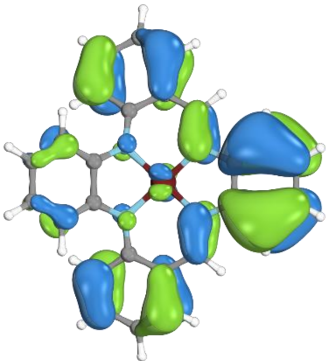	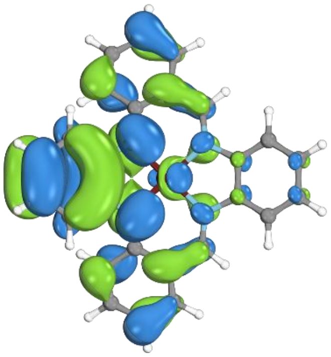
10	HOMO-4 → LUMO	0.64	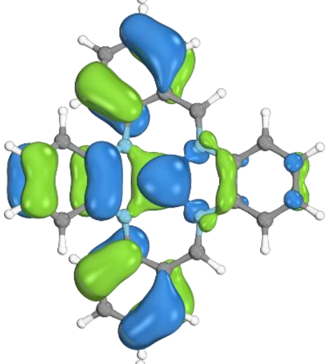	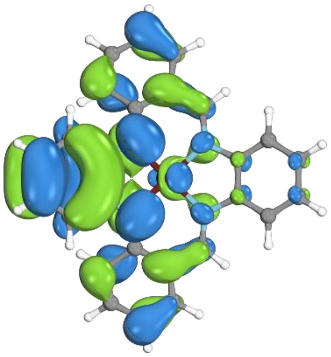
16	HOMO-1 → LUMO+1	0.48	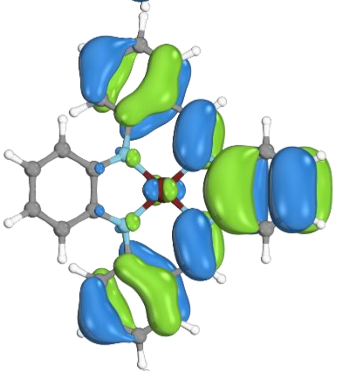	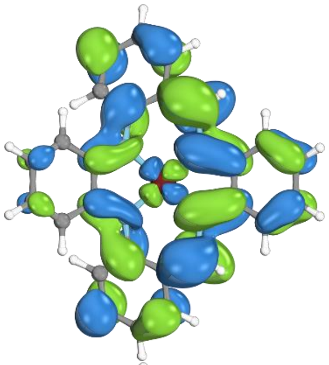
	HOMO-10 → LUMO+3	0.19	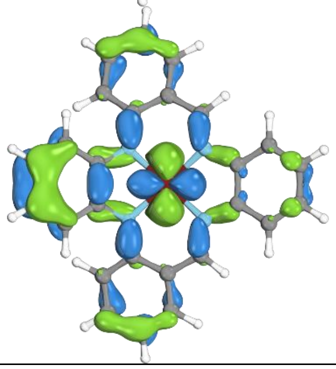	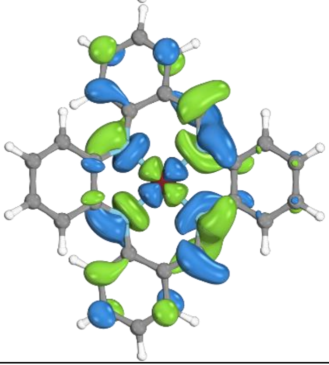

23	HOMO-1 → LUMO+2	0.50	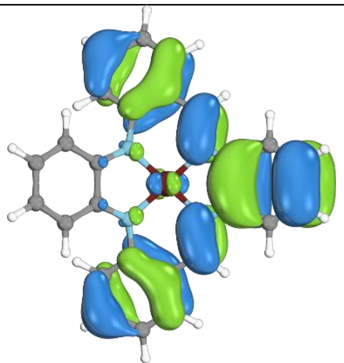	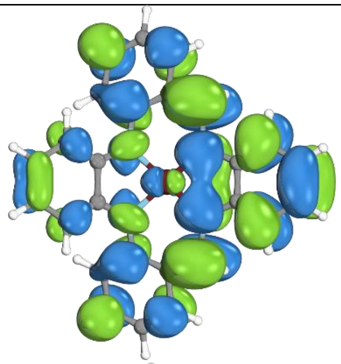
	HOMO-2 → LUMO+3	0.20	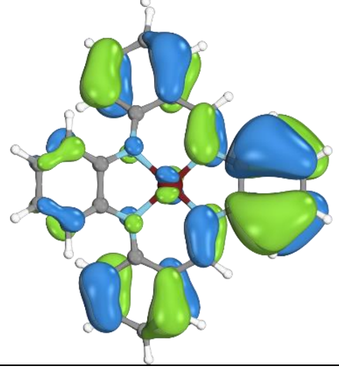	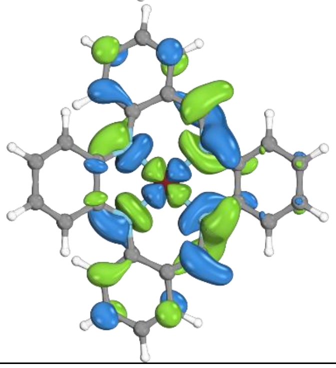

[Ni(Me2PhenTAA)]2– (CSS):

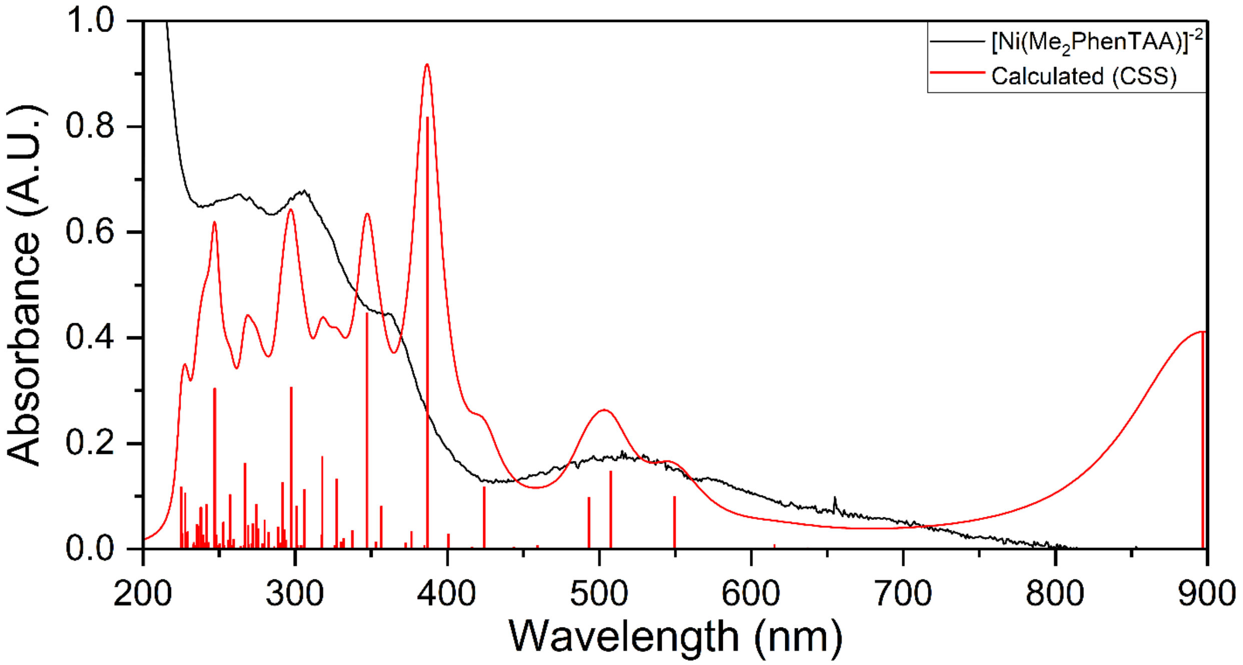

Figure S113. Experimental UV/Vis spectrum of $[Ni(Me_2PhenTAA)]^{2-}$ (**4b**$^{-2}$) in THF measured via spectro-electrochemistry (black) and calculated spectrum (red; Lorentzian peak FWHM: 1500 cm^{-1}). Individual excitations illustrated in red bars.

The UV/Vis spectrum for the doubly reduced $[Ni(Me_2PhenTAA)]^{2-}$ (**4b**$^{-2}$) species is entirely dominated by ligand → ligand excitations as seen in Table S25. These orbital pairs are predominantly π-orbitals, yet there are a few high energy σ*-orbitals involved as well (123 → 134). The absence of the strong absorption at 900 nm in the experimental UV/Vis spectrum is due to limitations of the spectrometer at this wavelength. The theoretical spectrum matches reasonably well with the experimental spectrum, barring the strong absorption at 386.9 nm.

Table S24. Selected states for the TD–DFT calculated absorptions of $[Ni(Me_2PhenTAA)]^{2-}$ (**4b**$^{-2}$) at the B3LYP/def2-TZVPP CPCMC (THF) level of theory.

State	Energy (cm^{-1})	Wavelength (nm)	fosc	T2 (au**2)	TX (au)	TY (au)	TZ (au)
5	19707.1	507.4	0.162560983	2.71562	-1.16531	-0.33180	-1.11695
15	25847.6	386.9	0.900507181	11.46947	-2.23974	-0.50927	2.48871
22	28811.5	347.1	0.492950013	5.63266	1.67999	0.47638	1.60728
36	33626.4	297.4	0.337341911	3.30268	1.28700	0.36402	1.23036
72	40477.5	247.1	0.334914981	2.72393	1.14284	0.32569	1.14533

Table S25. Most contributing orbital pairs to the selected states listed in Table S24 and their respective fractions.

State	Donating orbital → Accepting orbital	Fraction of total state	Donating orbital	Accepting orbital
5	HOMO → LUMO+4	0.90	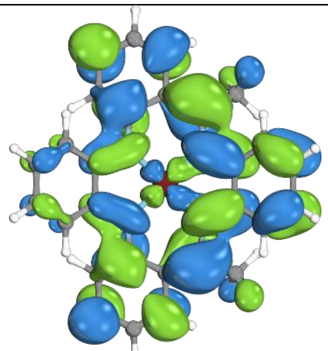	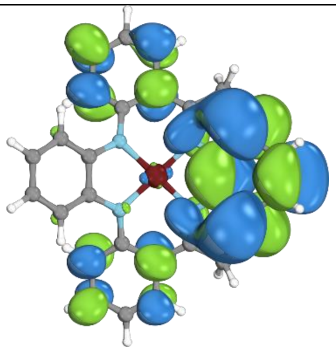
15	HOMO-3 → LUMO	0.44	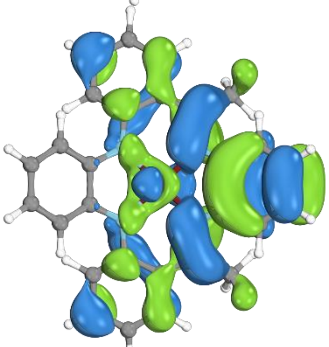	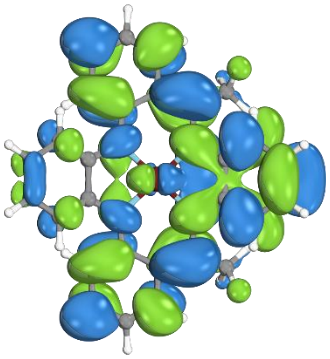
	HOMO → LUMO+3	0.26	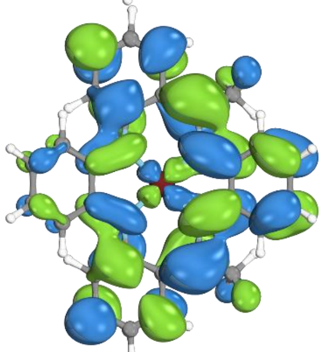	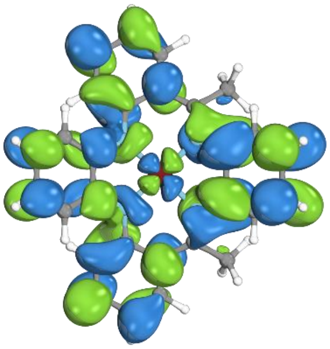
22	HOMO-1 → LUMO+1	0.36	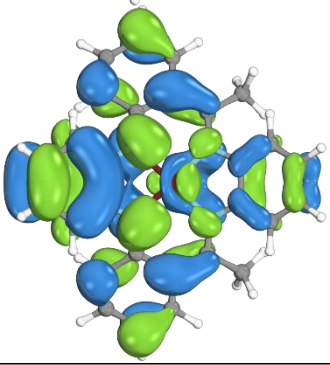	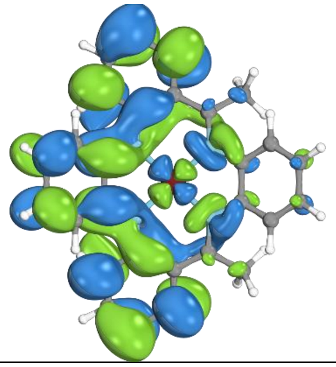

	HOMO-1 → LUMO+5	0.11	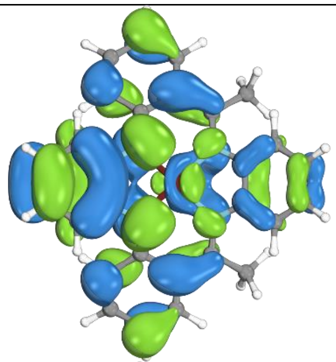	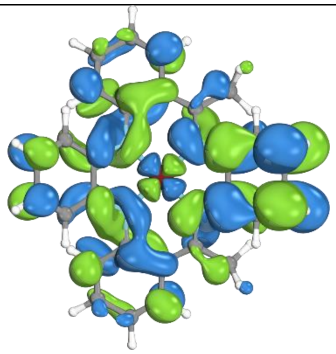
	HOMO → LUMO+10	0.13	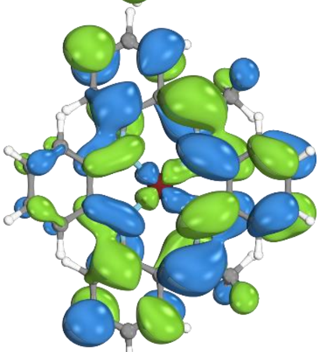	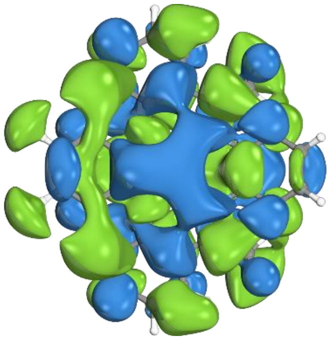
36	HOMO-3 → LUMO+1	0.35	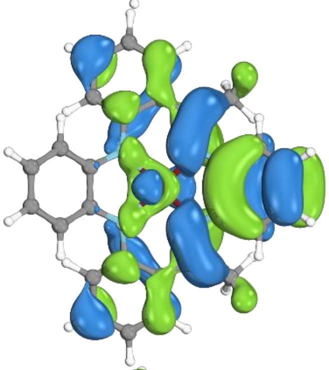	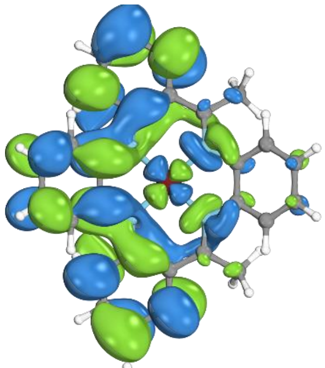
	HOMO-1 → LUMO+5	0.19	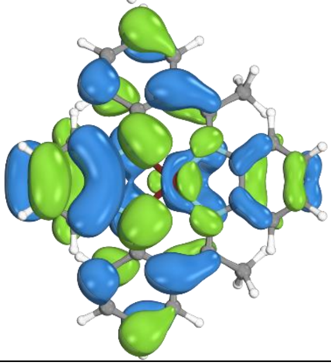	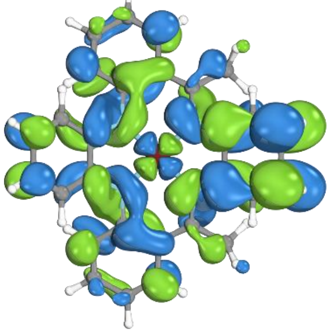

72	HOMO-2 → LUMO+7	0.76	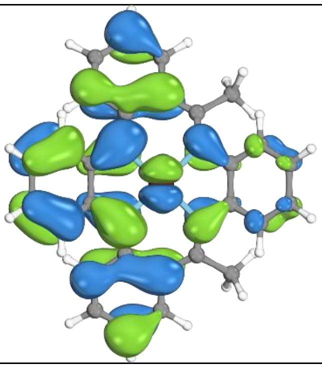	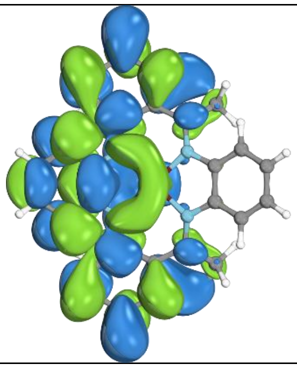

[Ni(Me$_2$PhenTAA)]$^-$ (Doublet):

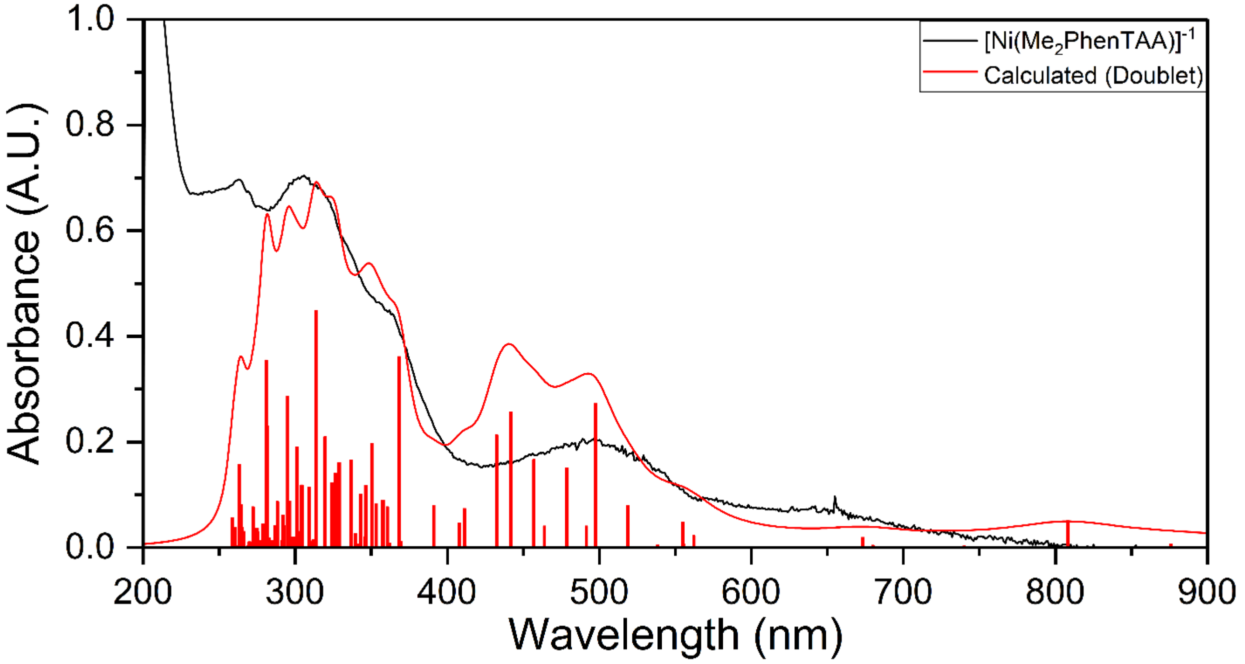

Figure S114. Experimental UV/Vis spectrum of [Ni(Me$_2$PhenTAA)]$^-$ (**4b^{-1}**) in THF measured via spectro-electrochemistry (black) and calculated spectrum (red; Lorentzian peak FWHM: 1500 cm^{-1}). Individual excitations illustrated in red bars.

The theoretical spectrum is a good match for the experimental UV/Vis spectrum with all major excitations accounted for. The spectrum is dominated by ligand → ligand excitations (typical for PhenTAA complexes), with a prominent MLCT (d_{z2} → L) excitation as the exception (119 → 124).

Table S26. Selected states for the TD–DFT calculated absorptions of [Ni(Me$_2$PhenTAA)]$^-$ (**4b^{-1}**) at the B3LYP/def2-TZVPP CPCMC (THF) level of theory.

State	Energy (cm^{-1})	Wavelength (nm)	fosc	T2 (au**2)	TX (au)	TY (au)	TZ (au)
7	14851.7	673.3	0.009888917	0.21920	-0.25482	-0.21080	0.33141
14	20104.6	497.4	0.136523261	2.23557	-0.74061	-0.80650	1.01815
21	22627.1	441.9	0.128604218	1.87112	0.80291	0.50988	-0.98310
30	27141.3	368.4	0.180908865	2.19434	0.98731	0.19106	-1.08768
50	31896.4	313.5	0.224748924	2.31970	1.07623	0.30846	1.03261

Table S27. Most contributing orbital pairs to the selected states listed in Table S26 and their respective fractions.

State	Donating orbital → Accepting orbital	Fraction of total state	Donating orbital	Accepting orbital
7	HOMO-1 (α) → LUMO (α)	0.72	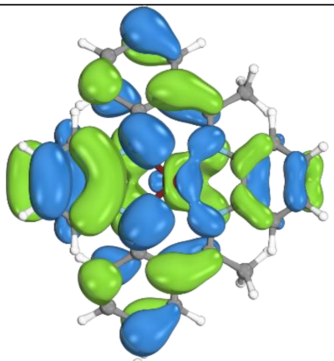	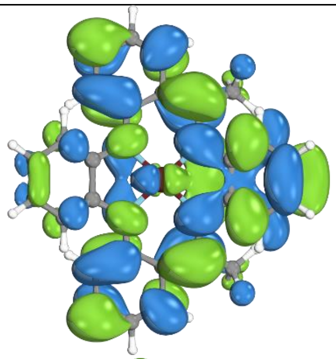
14	HOMO (α) → LUMO+3 (α)	0.29	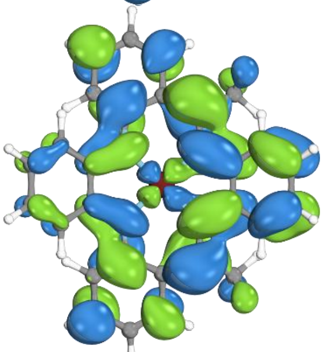	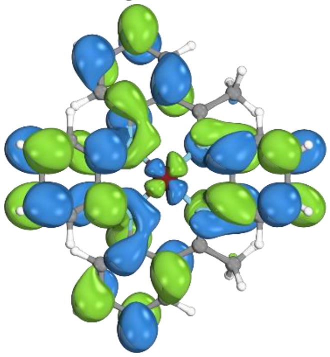
	HOMO (α) → LUMO+5 (α)	0.19	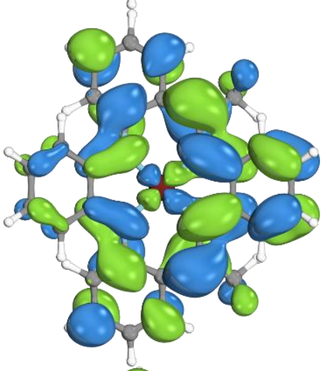	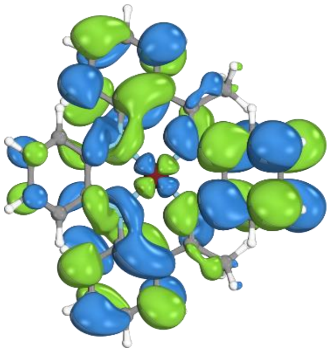
	HOMO (β)→ LUMO+1 (β)	0.31	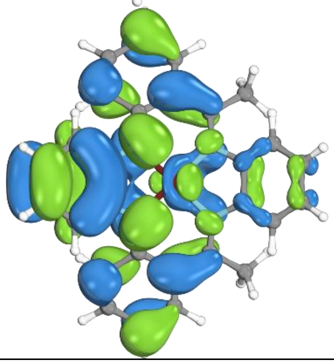	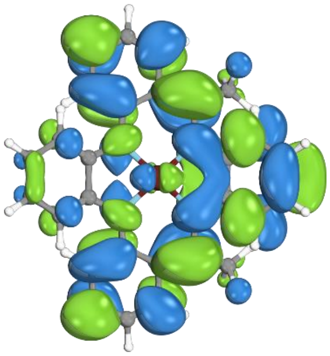

21	HOMO-4 (α) → LUMO (α)	0.37	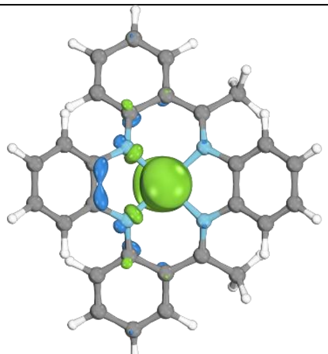	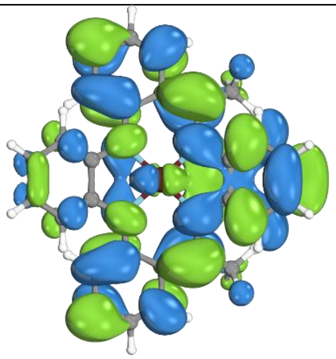
	HOMO-1 (β) → LUMO (β)	0.11	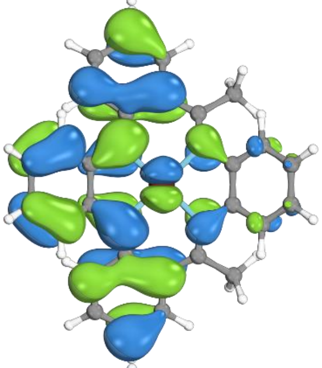	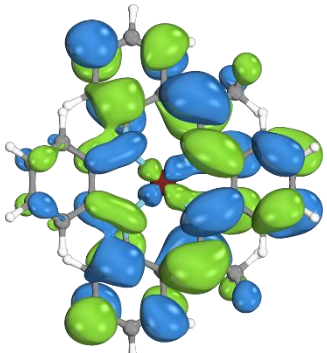
	HOMO-4 (β) → LUMO (β)	0.08	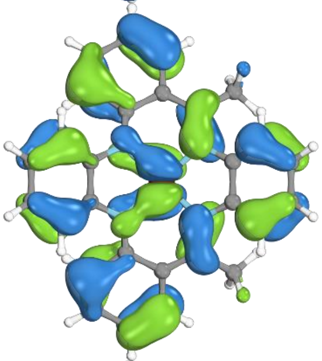	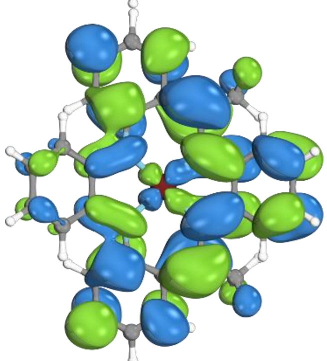
30	HOMO-2 (β)→ LUMO +1 (β)	0.38	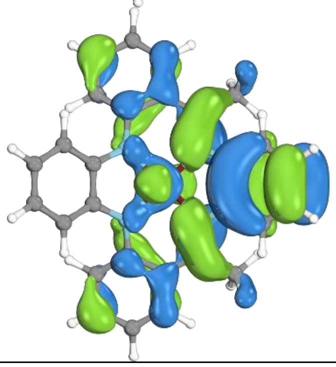	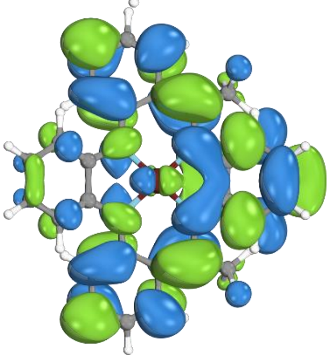

50	HOMO-4 (β) → LUMO (β)	0.15	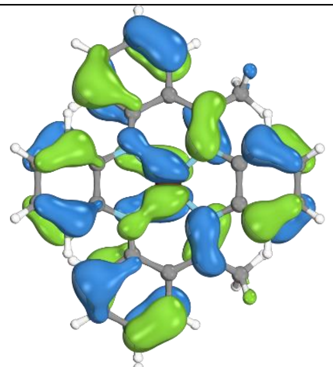	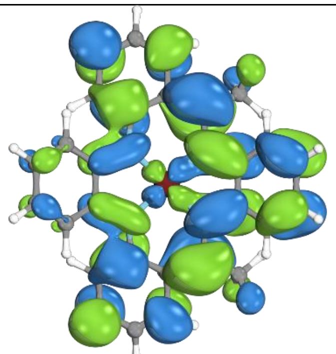
	HOMO-3 (β) → LUMO +1 (β)	0.15	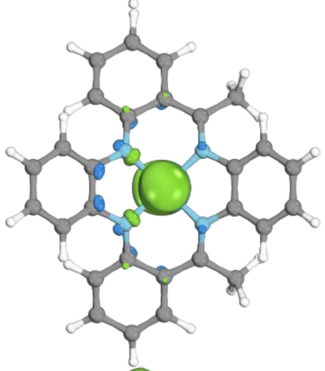	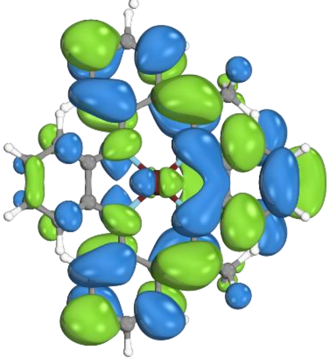
	HOMO (β) → LUMO+5 (β)	0.28	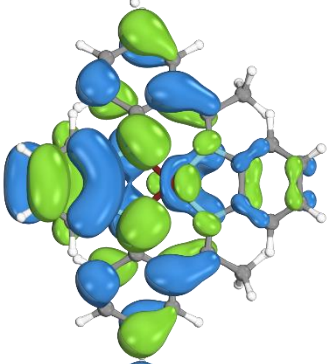	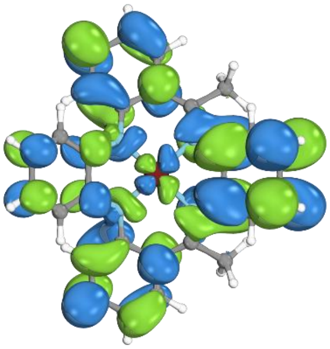
	HOMO-1 (α) → LUMO+3 (α)	0.15	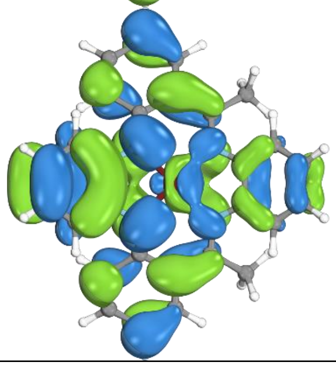	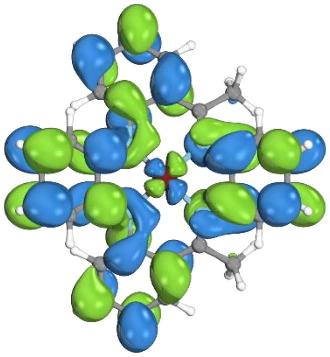

	HOMO-4 (β)→ LUMO+1 (β)	0.09	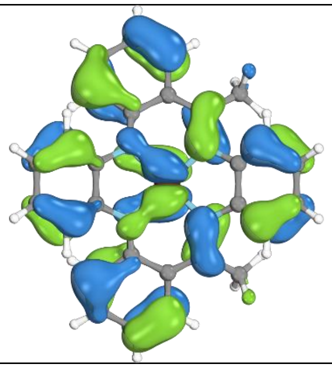	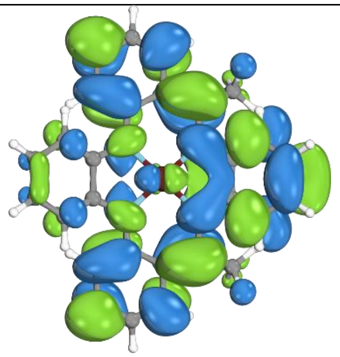

[Ni(Me$_2$PhenTAA)] (CSS):

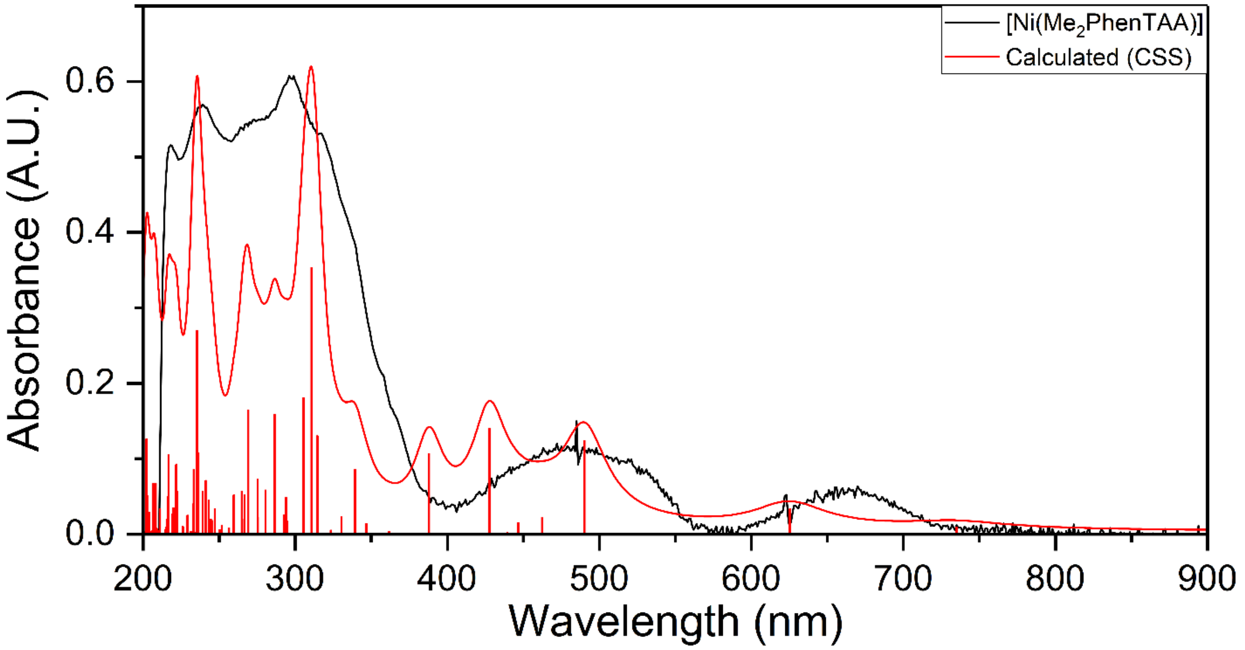

Figure S115. Experimental UV/Vis spectrum of [Ni(Me$_2$PhenTAA)] **(4b)** in CH_2Cl_2 measured in an OTTLE cell (black) and calculated spectrum (red; Lorentzian peak FWHM: 1500 cm^{-1}). Individual excitations illustrated in red bars.

A good match was obtained for the calculated spectrum with the experimental UV/Vis spectrum. The selected excitations correspond to the four main peaks observed in the experimental spectrum. They largely correlate with excitation from the donor diaryl-*o*-phenylene diamine moiety to the diimine acceptor moiety. State 51 also features excitation to a Rydberg state (a high energy s-like orbital) (121 → 132) from the donor moiety. The Rydberg state is centered around the acceptor moiety.

Table S28. Selected states for the TD–DFT calculated absorptions of [Ni(Me$_2$PhenTAA)] (**4b)** at the B3LYP/def2-TZVPP CPCMC (CH_2Cl_2) level of theory.

State	Energy (cm^{-1})	Wavelength (nm)	fosc	T2 (au**2)	TX (au)	TY (au)	TZ (au)
1	13606.2	735.0	0.003792945	0.32948	0.40600	0.11565	0.38894
2	15992.7	625.3	0.006851234	0.98676	-0.53193	-0.47091	0.69430
4	20399.8	490.2	0.177460745	2.86386	-0.91966	-0.77715	1.18917
19	32170.1	310.8	0.504805798	5.16592	1.60712	0.46123	1.53959
51	42501.8	235.3	0.385955086	2.98955	1.22449	0.34854	1.16990

Table S29. Most contributing orbital pairs to the selected states listed in Table S28 and their respective fractions.

State	Donating orbital → Accepting orbital	Fraction of total state	Donating orbital	Accepting orbital
1	HOMO → LUMO	0.92	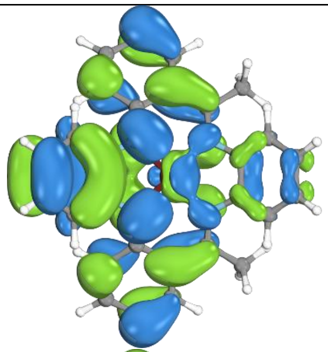	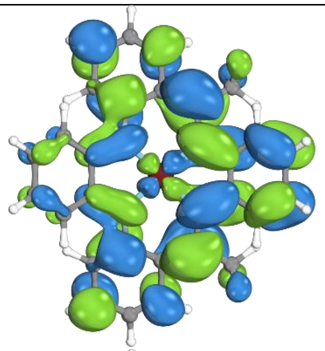
2	HOMO-1 → LUMO	0.77	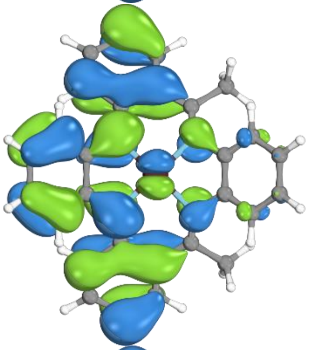	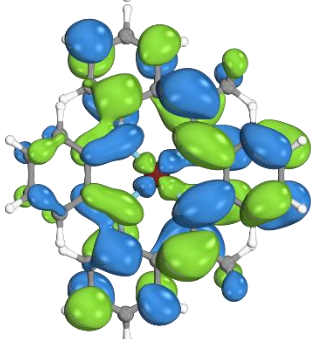
4	HOMO → LUMO+1	0.86	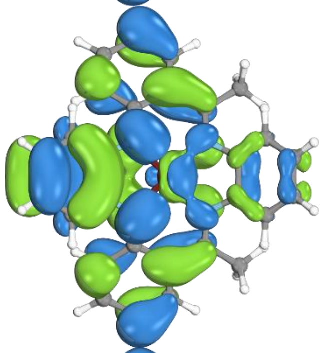	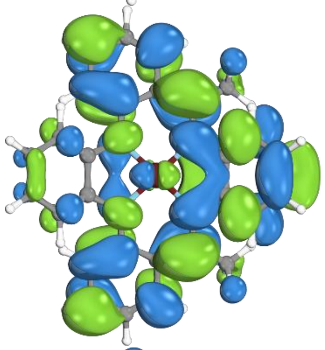
19	HOMO → LUMO+5	0.52	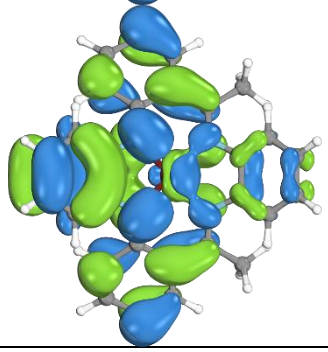	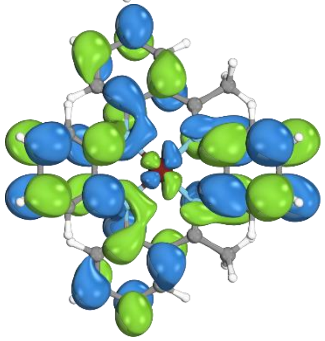

51	HOMO → LUMO+11	0.47	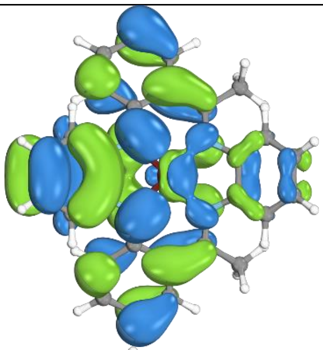	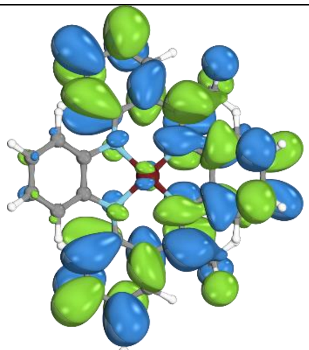
	HOMO-12 → LUMO	0.12	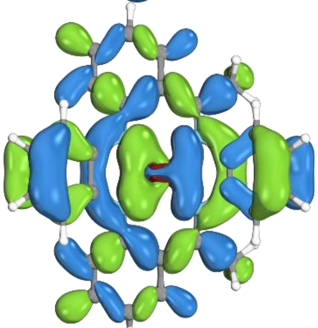	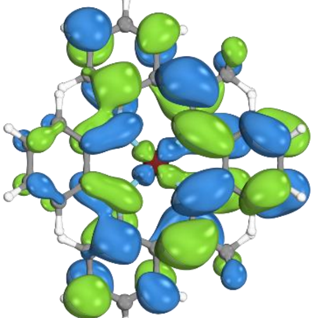
	HOMO-1 → LUMO+9	0.10	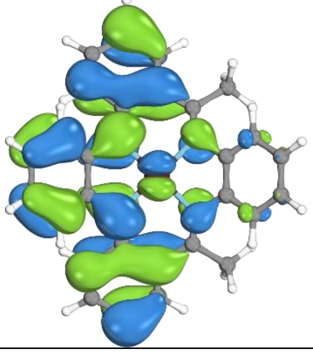	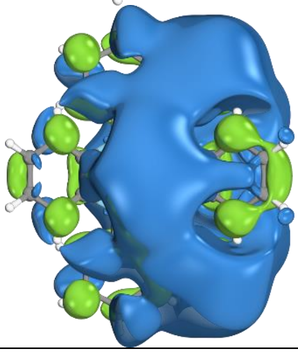

[Ni(Me₂PhenTAA)]⁺ (Doublet):

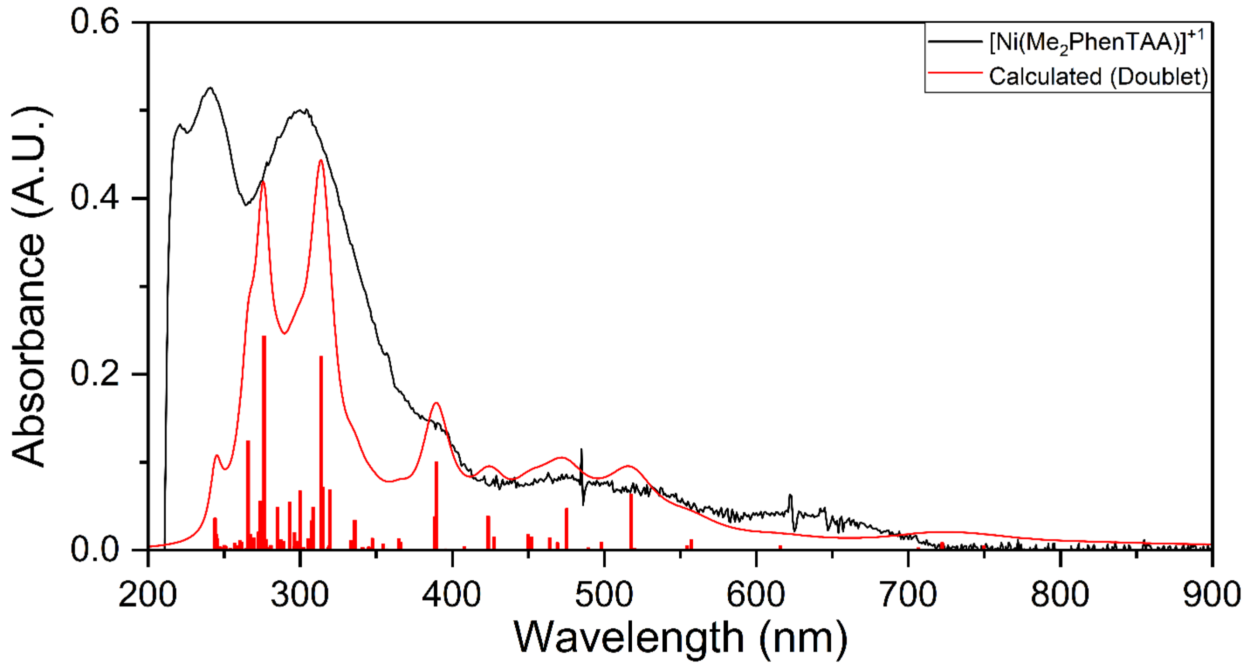

Figure S116. Experimental UV/Vis spectrum of $[Ni(Me_2PhenTAA)]^+$ (**4b^{1+}**) in CH_2Cl_2 measured via spectroelectrochemistry (black) and calculated spectrum (red; Lorentzian peak FWHM: 1500 cm^{-1}). Individual excitations illustrated in red bars.

The excitations shown in Table S31 reveal a similar pattern as for $[Ni(H_2PhenTAA)]^+$ (**4b^{1+}**), featuring solely ligand → ligand excitations that are centered around excitations from the donor moiety to the PhenTAA acceptor moiety. The theoretical spectrum matches the experimental spectrum well.

Table S30. Selected states for the TD–DFT calculated absorptions of $[Ni(Me_2PhenTAA)]^+$ (**4b^{1+}**) at the B3LYP/def2-TZVPP CPCMC (CH_2Cl_2) level of theory.

State	Energy (cm^{-1})	Wavelength (nm)	fosc	T2 (au**2)	TX (au)	TY (au)	TZ (au)
8	16240.5	615.7	0.008278750	0.16782	0.24867	0.13138	-0.29786
13	19320.7	517.6	0.106220054	1.80992	-0.60707	-0.81002	0.88614
16	21039.6	475.3	0.079018571	1.23642	0.60787	0.49936	-0.78585
26	25662.3	389.7	0.168013874	2.15539	-1.03926	-0.29656	-0.99367
47	31871.7	313.8	0.367014237	3.79099	1.37399	-0.48213	-1.29255
68	36207.5	276.2	0.406336971	3.69457	1.36115	0.38740	1.30068

Table S31. Most contributing orbital pairs to the selected states listed in Table S30 and their respective fractions. For brevity, the corresponding β-orbital pairs were omitted and their fractional contributions added to the α-orbital pairs.

State	Donating orbital → Accepting orbital	Fraction of total state	Donating orbital	Accepting orbital
8	HOMO-1 (α) → LUMO (α)	0.60	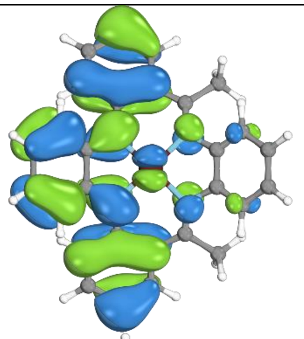	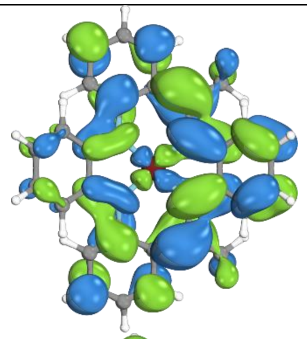
	HOMO-4 (β) → LUMO (β)	0.12	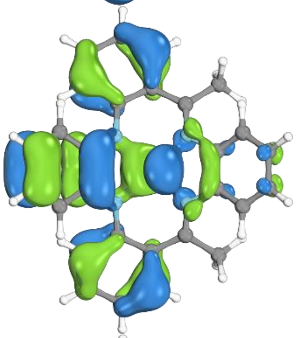	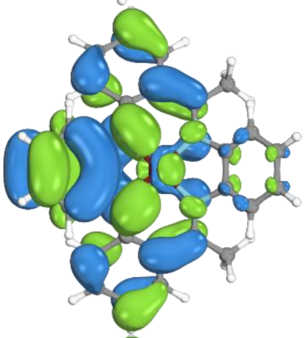
13	HOMO-4 (β) → LUMO (β)	0.33	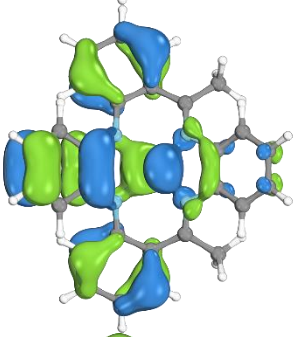	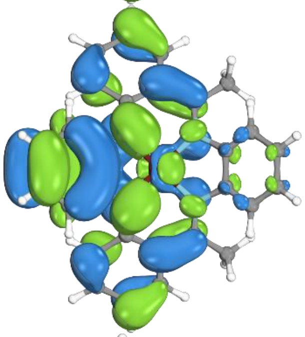
	HOMO (α) → LUMO (α)	0.31	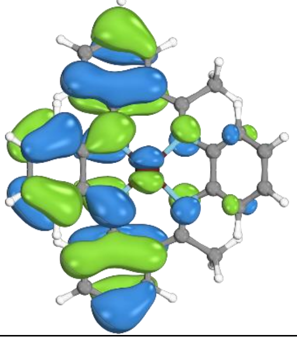	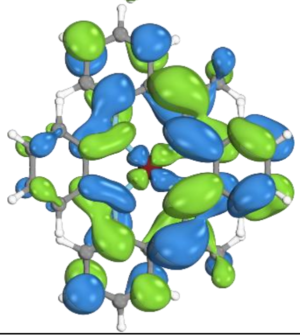

16	HOMO (α) → LUMO+1 (α)	0.67	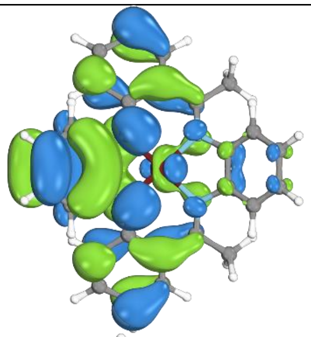	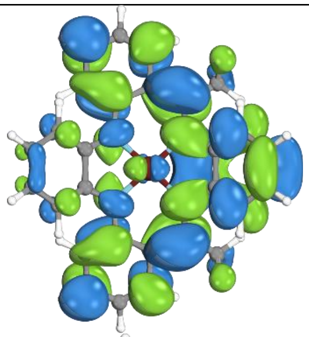
26	HOMO-1 (α) → LUMO (α)	0.46	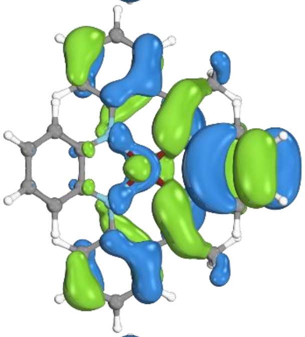	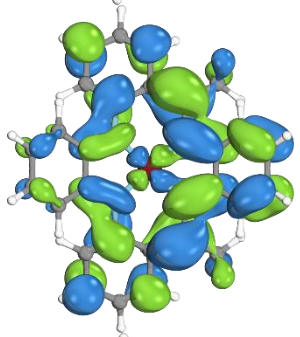
	HOMO (α) → LUMO+2 (α)	0.11	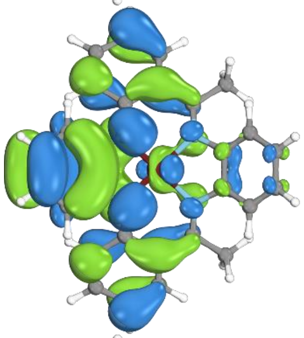	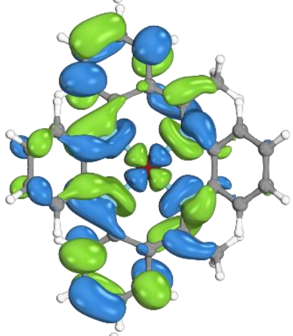
47	HOMO-2 (α)→ LUMO+1 (α)	0.33	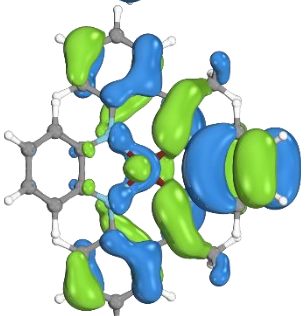	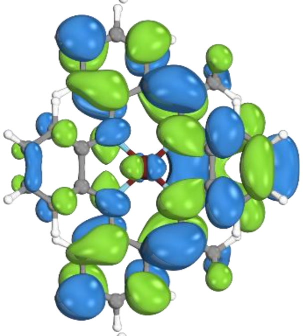
	HOMO-4 (α) → LUMO (α)	0.23	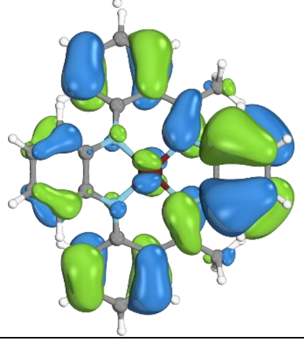	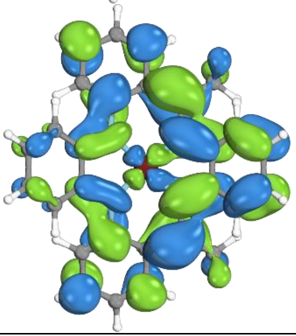

	HOMO (β) → LUMO+5 (β)	0.11	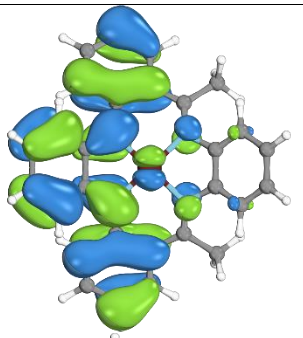	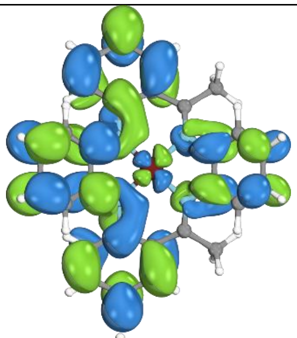
68	HOMO-2 (α) → LUMO+2 (α)	0.24	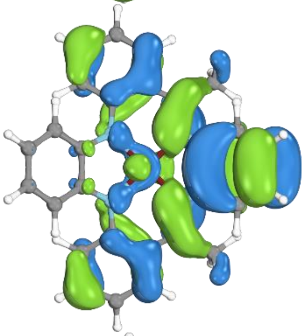	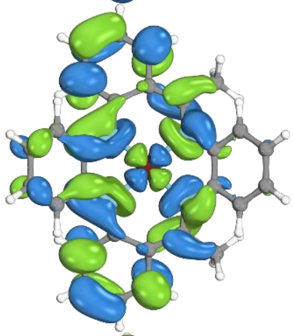
	HOMO-14 (β) → LUMO (β)	0.18	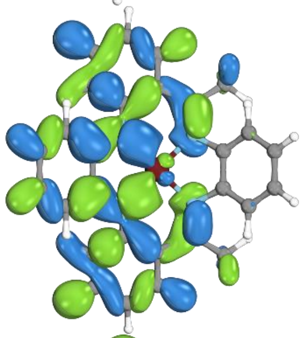	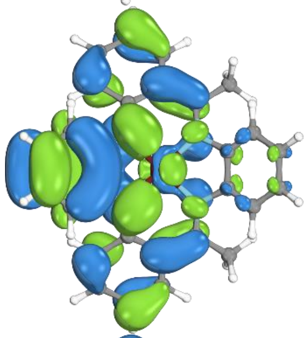
	HOMO-1 (α)→ LUMO+3 (α)	0.10	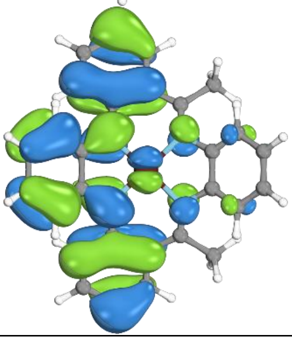	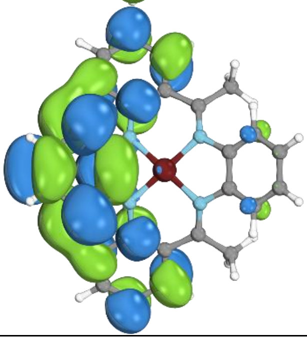

[Ni(Me$_2$PhenTAA)]$^{2+}$ (CSS):

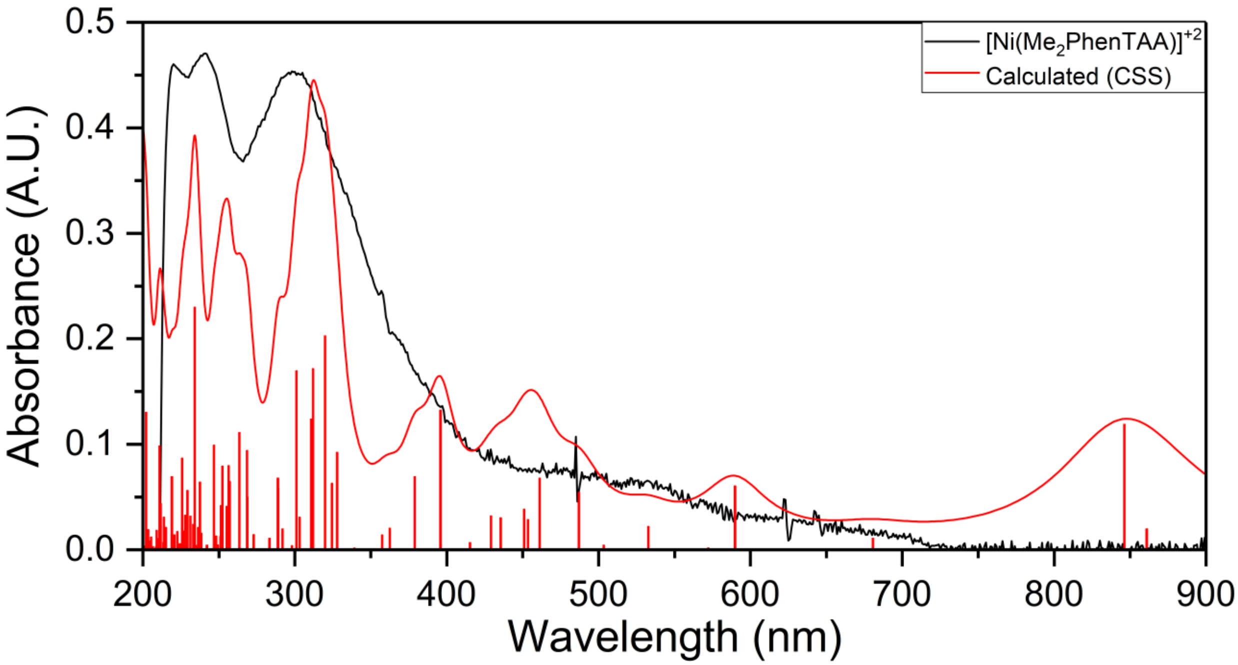

Figure S117. Experimental UV/Vis spectrum of [Ni(Me$_2$PhenTAA)]$^{2+}$ (**4b^{2+}**) in CH_2Cl_2 measured via spectro-electrochemistry (black) and calculated spectrum (red; Lorentzian peak FWHM: 1500 cm^{-1}). Individual excitations illustrated in red bars. The peak at 850 nm could not be accurately measured by the spectrometer.

The calculated spectrum for [Ni(Me$_2$PhenTAA)]$^{2+}$ (**4b^{2+}**) matches well with experimental data. When analyzing the orbital pairs, one can see that the donor-acceptor relation observed in more reduced PhenTAA species is reversed. The oxidized *o*-diiminoquinone now occupies the role of the acceptor moiety, whereas electrons are now excited from the diiminophenylene moiety. Furthermore, several prominent LMCT excitations are now present where the electron-rich d$_8$-metal donates electrons to the highly electron deficient *o*-diiminoquinone moiety.

Table S32. Selected states for the TD–DFT calculated absorptions of [Ni(Me$_2$PhenTAA)]$^{2+}$ (**4b^{2+}**) at the B3LYP/def2-TZVPP CPCMC (CH_2Cl_2) level of theory.

State	Energy (cm^{-1})	Wavelength (nm)	fosc	T2 (au**2)	TX (au)	TY (au)	TZ (au)
4	16953.8	589.8	0.085114456	1.65277	-0.90915	-0.25924	-0.87121
9	21683.2	461.2	0.095615171	1.45171	0.85170	0.24437	0.81645
15	25259.7	395.9	0.185443219	2.41690	-0.49874	-1.18727	0.87095
22	31258.6	319.9	0.284136565	2.99249	-1.21580	0.52849	1.11132
57	42715.3	234.1	0.322064318	2.48219	-1.11490	-0.31750	-1.06695

Table S33. Most contributing orbital pairs to the selected states listed in Table S32 and their respective fractions.

State	Donating orbital → Accepting orbital	Fraction of total state	Donating orbital	Accepting orbital
4	HOMO-2 → LUMO	0.93	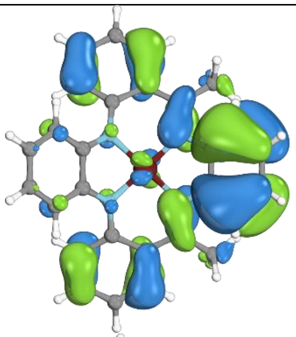	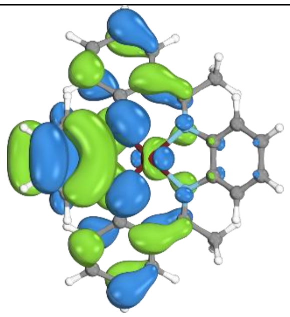
9	HOMO-5 → LUMO	0.41	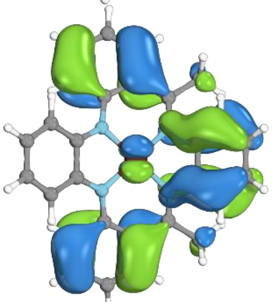	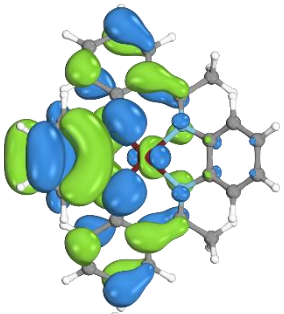
	HOMO-1 → LUMO+1	0.14	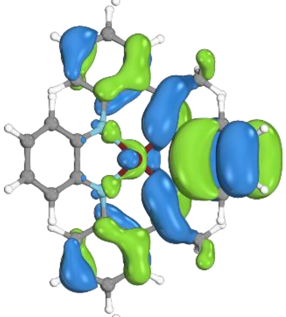	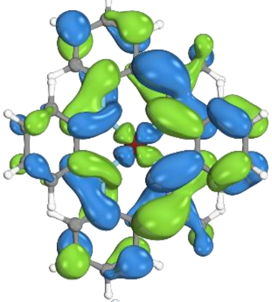
	HOMO-8 → LUMO	0.11	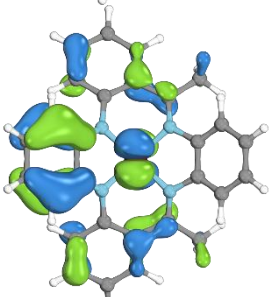	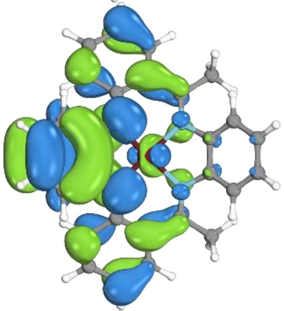
15	HOMO-9 → LUMO	0.63	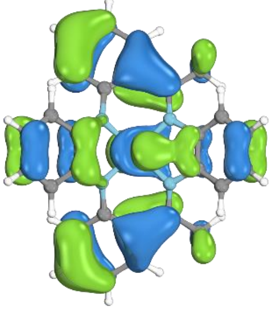	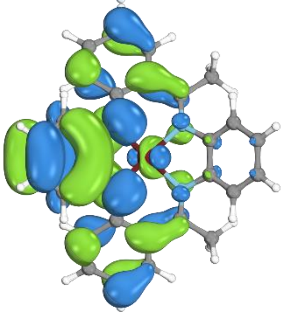

22	HOMO-1 → LUMO+2	0.58	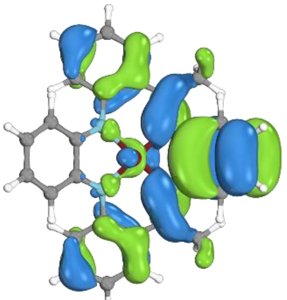	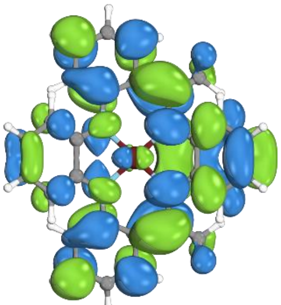
	HOMO → LUMO+3	0.19	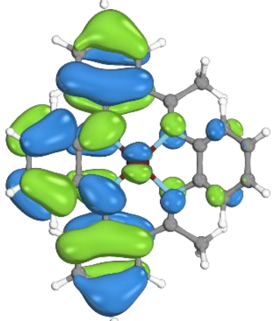	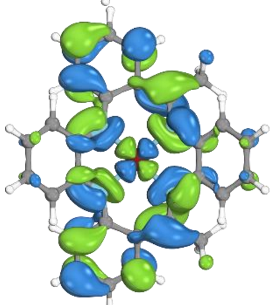
57	HOMO-11 → LUMO+1	0.35	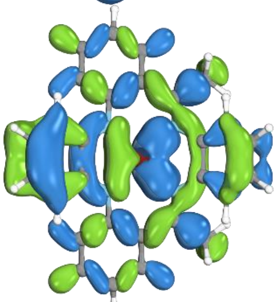	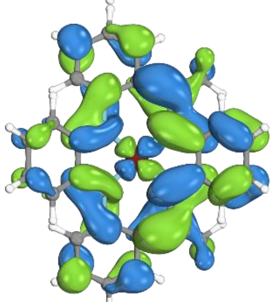
	HOMO-10 → LUMO+1	0.20	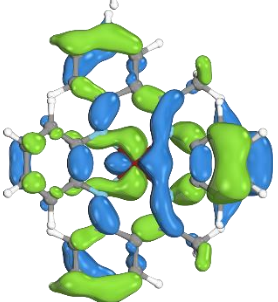	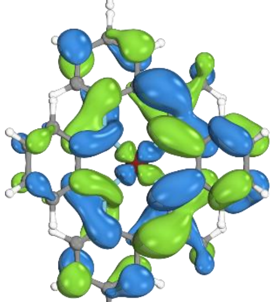
	HOMO-3 → LUMO+5	0.16	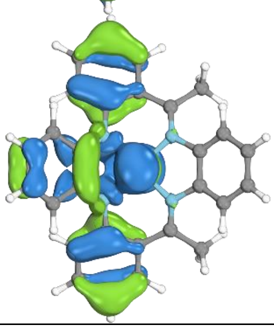	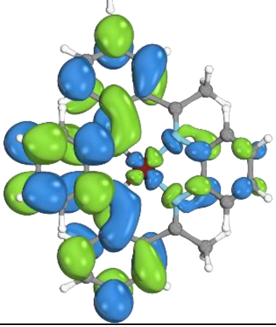

[Ni(Ph₂PhenTAA)]²⁻ (CSS):

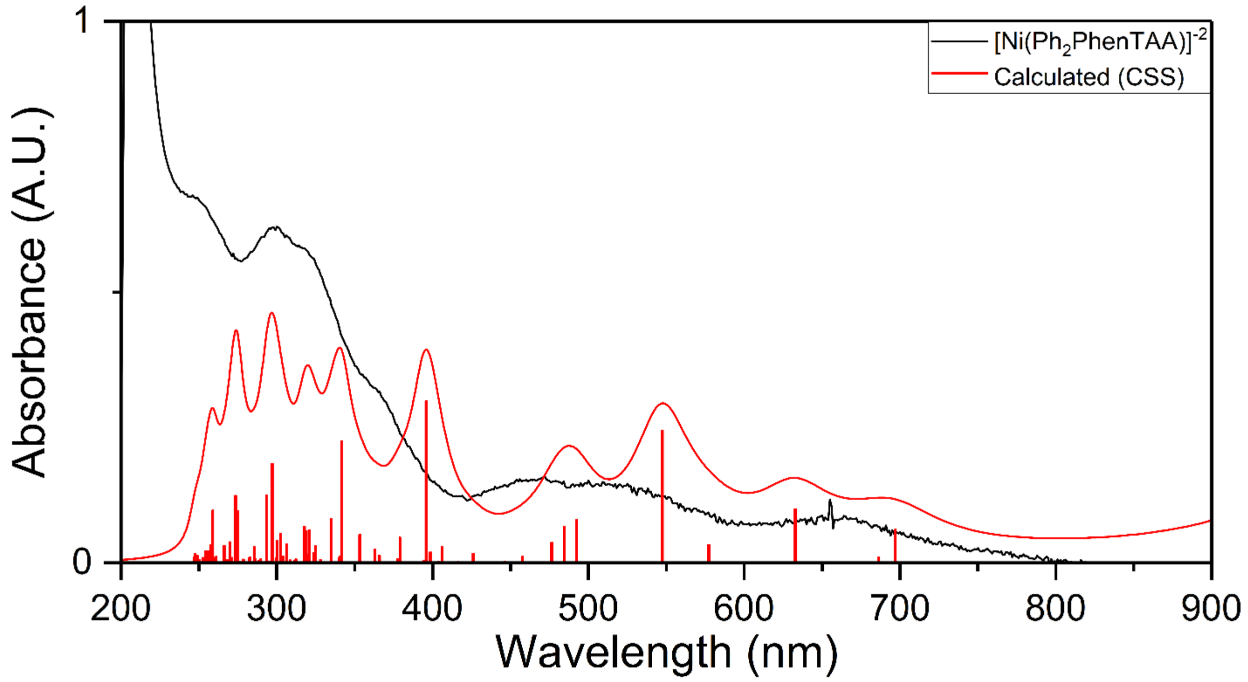

Figure S118. Experimental UV/Vis spectrum of $[Ni(Ph_2PhenTAA)]^{2-}$ (**4c**$^{-2}$) in THF measured via spectro-electrochemistry (black) and calculated spectrum (red; Lorentzian peak FWHM: 1500 cm^{-1}). Individual excitations illustrated in red bars.

Due to the additional phenyl ligands, excitations from the central PhenTAA moiety to the peripheral phenyl rings are predominant throughout the UV/Vis spectrum. Furthermore, the highly reduced nature of the complex and its globally delocalized electronic structure results in a shift from the usual donor↔acceptor excitations. Both the donating and accepting orbital feature molecular orbitals that are delocalized throughout the PhenTAA macrocycle.

Table S34. Selected states for the TD–DFT calculated absorptions of $[Ni(Ph_2PhenTAA)]^{2-}$ (**4c**$^{-2}$) at the B3LYP/def2-TZVPP CPCMC (THF) level of theory.

State	Energy (cm^{-1})	Wavelength (nm)	fosc	T2 (au**2)	TX (au)	TY (au)	TZ (au)
5	15804.7	632.7	0.149835852	3.12108	-0.84716	0.13078	1.54476
8	18266.8	547.4	0.367085690	6.61576	-1.22853	0.22821	2.24820
10	20305.9	492.5	0.120379798	1.95167	-0.54667	0.48447	1.19085
20	25270.0	395.7	0.449173259	5.85174	-1.28209	-1.19771	1.66537
30	29279.5	341.5	0.337613319	3.79604	1.63941	-0.46281	0.94561
54	33674.7	297.0	0.276402177	2.70217	1.38426	-0.38851	0.79691

Table S35. Most contributing orbital pairs to the selected states listed in **Table S34.** and their respective fractions.

State	Donating orbital → Accepting orbital	Fraction of total state	Donating orbital	Accepting orbital
5	HOMO → LUMO+2	0.41	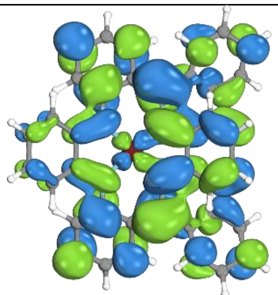	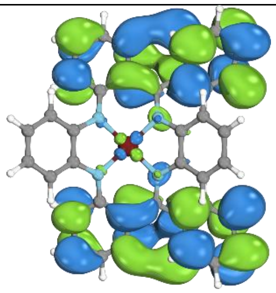
	HOMO → LUMO+3	0.43	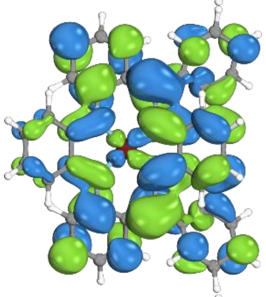	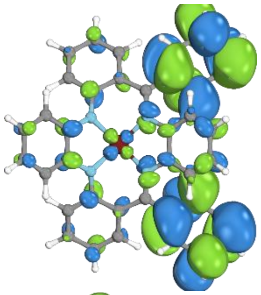
8	HOMO → LUMO+5	0.41	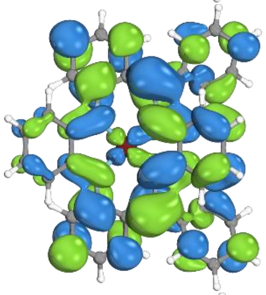	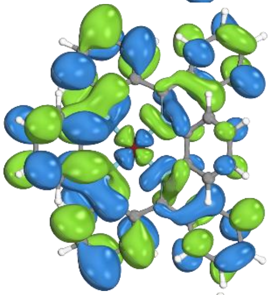
	HOMO-1 → LUMO	0.30	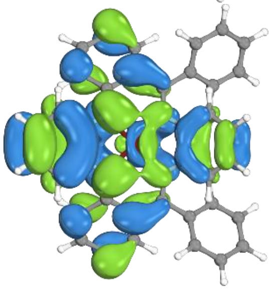	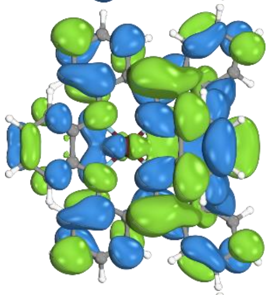
10	HOMO-3 → LUMO	0.42	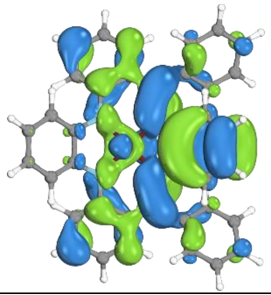	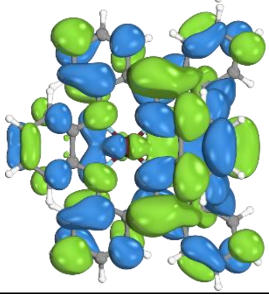

	HOMO → LUMO+8	0.30	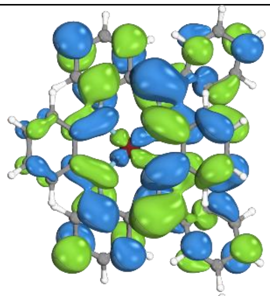	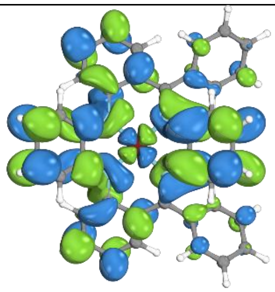
20	HOMO → LUMO+9	0.42	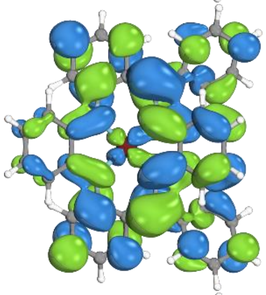	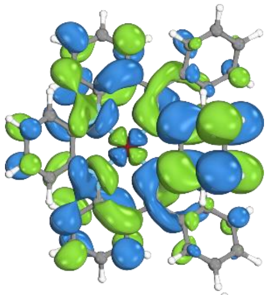
	HOMO → LUMO+8	0.20	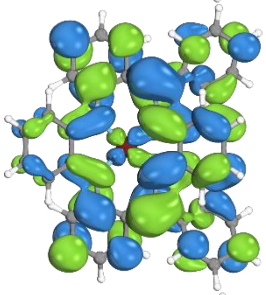	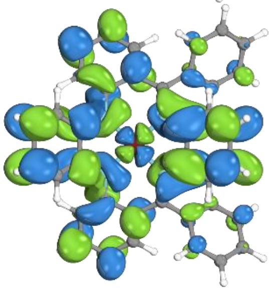
30	HOMO-1 → LUMO+5	0.33	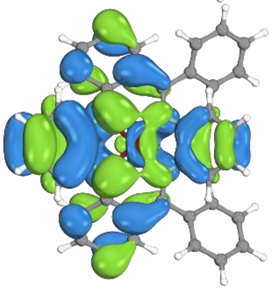	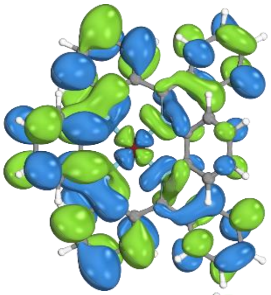
	HOMO-3 → LUMO+2	0.10	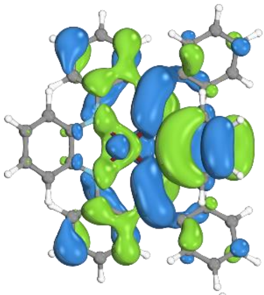	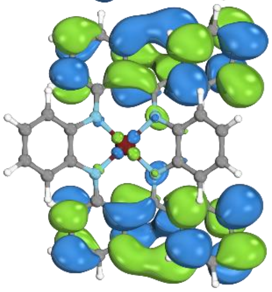
	HOMO-1 → LUMO+8	0.10	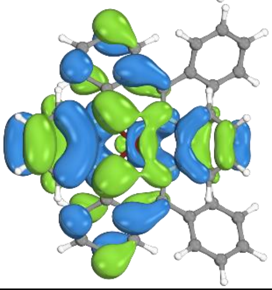	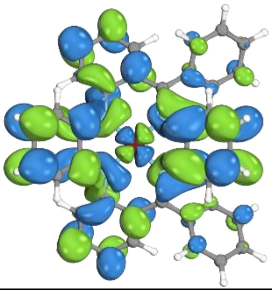

54	HOMO-3 → LUMO+5	0.46	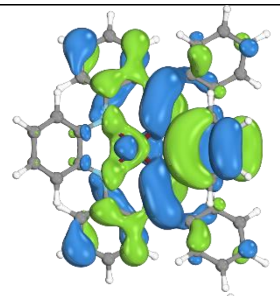	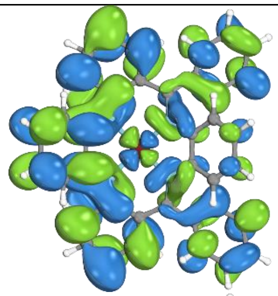
	HOMO-1 → LUMO+8	0.12	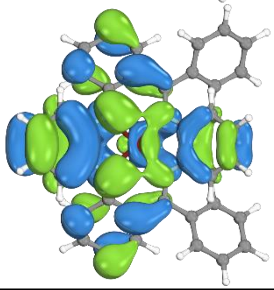	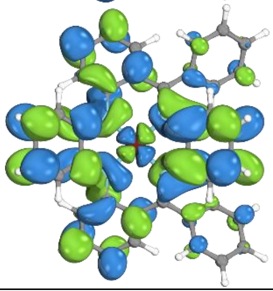

[Ni(Ph_2PhenTAA)]⁻ (Doublet):

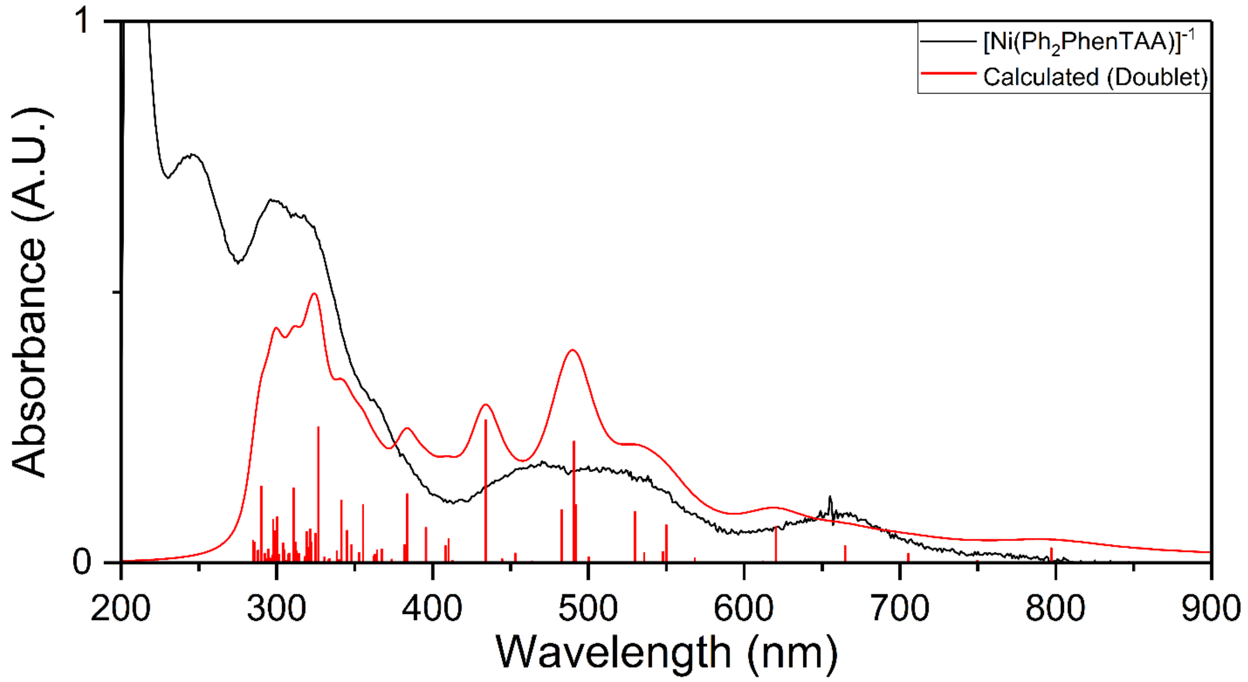

Figure S119. Experimental UV/Vis spectrum of [Ni(Ph_2PhenTAA)]⁻ (**4c**$^{-1}$) in THF measured via spectro-electrochemistry (black) and calculated spectrum (red; Lorentzian peak FWHM: 1500 cm^{-1}). Individual excitations illustrated in red bars.

The theoretically spectrum is in good agreement with experimental data and features a mix of the usual donor ↔ acceptor excitations and the globally delocalized structures seen for the doubly reduced [Ni(Ph_2PhenTAA)]$^{2-}$ (**4c**$^{-2}$), being intermediate between the neutral and doubly reduced forms. Additionally, a MLCT excited state from the Ni(d_{xz}) orbital to a globally delocalized ligand orbital is present as well.

Table S36. Selected states for the TD–DFT calculated absorptions of [Ni(Ph_2PhenTAA)]⁻ (4c^{-1}) at the B3LYP/def2-TZVPP CPCMC (THF) level of theory.

State	Energy (cm^{-1})	Wavelength (nm)	fosc	T2 (au**2)	TX (au)	TY (au)	TZ (au)
9	16117.6	620.4	0.054100968	1.10505	-0.51263	0.05325	0.91620
16	18873.9	529.8	0.076062382	1.32674	0.55716	-0.09351	-1.00377
19	20379.4	490.7	0.179641150	2.90195	0.89214	-0.06614	-1.44971
26	23044.0	434.0	0.211862987	3.02672	0.91448	0.29992	-1.44931
59	30620.6	326.6	0.201714576	2.16870	-1.24089	0.34823	-0.71248

Table S37. Most contributing orbital pairs to the selected states listed in **Table S36** and their respective fractions.

State	Donating orbital → Accepting orbital	Fraction of total state	Donating orbital	Accepting orbital
9	HOMO (α) → LUMO+3 (α)	0.66	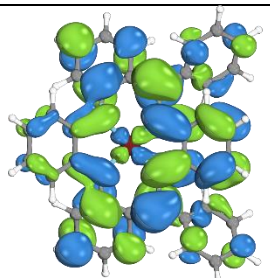	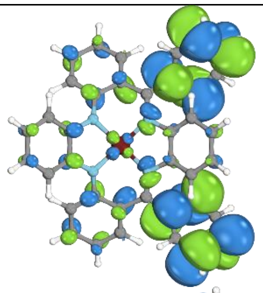
16	HOMO (β) → LUMO+1 (β)	0.65	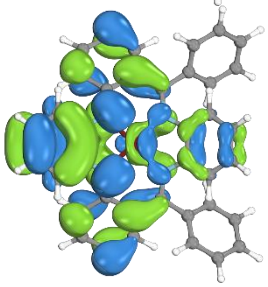	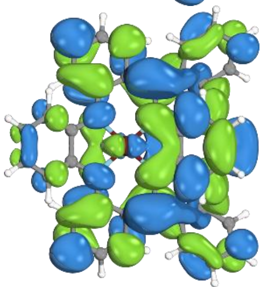
19	HOMO (α) → LUMO+7 (α)	0.58	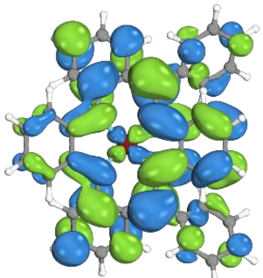	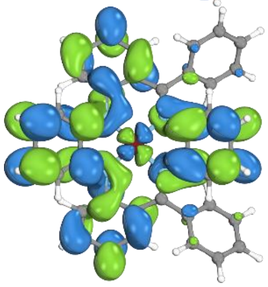
26	HOMO (α) → LUMO+9 (α)	0.50	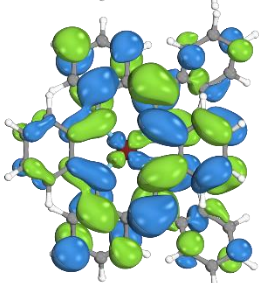	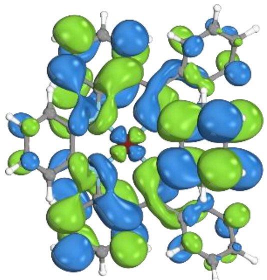
59	HOMO-5 (β) → LUMO (β)	0.11	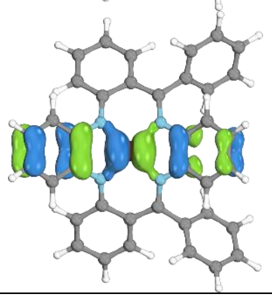	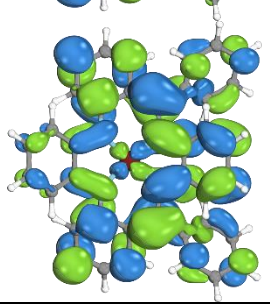

	HOMO (β) → LUMO+6 (β)	0.13	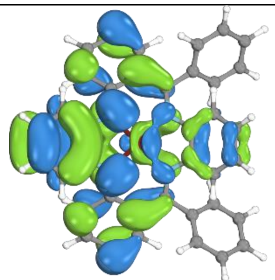	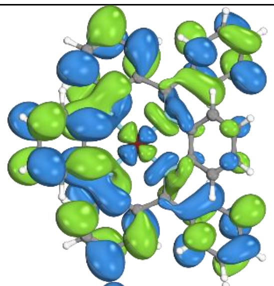
	HOMO-1 (α) → LUMO+7 (α)	0.06	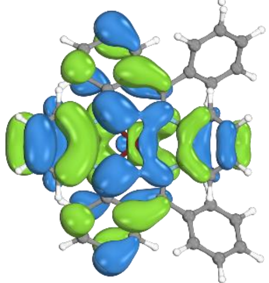	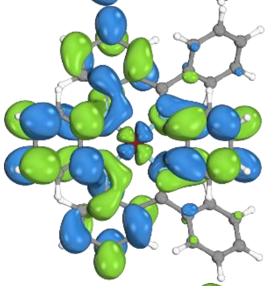
	HOMO-2 (β) → LUMO+3 (β)	0.05	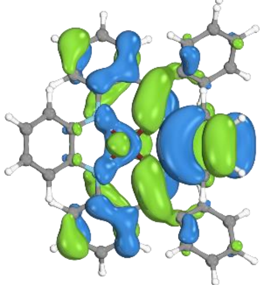	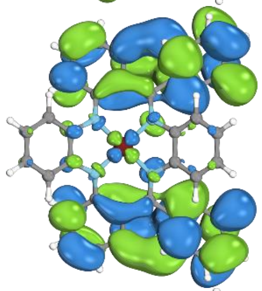
	HOMO (β) → LUMO+10 (β)	0.06	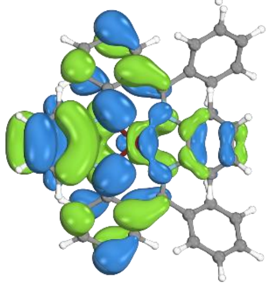	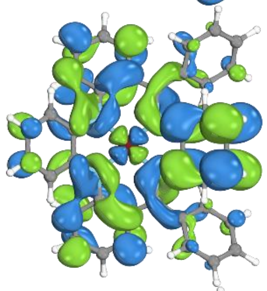
	HOMO-2 (β) → LUMO+9 (β)	0.04	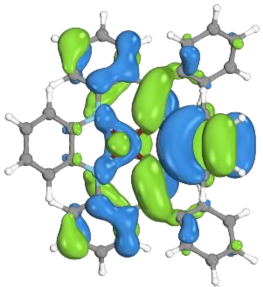	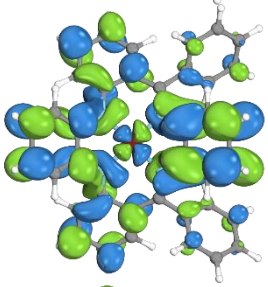
	HOMO-5 (β) → LUMO+6 (β)	0.04	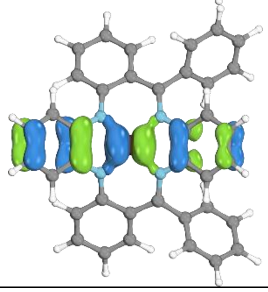	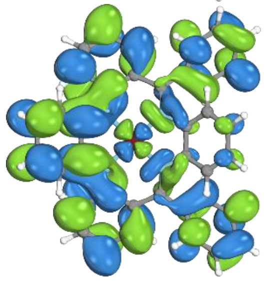

[Ni(Ph$_2$PhenTAA)] (CSS):

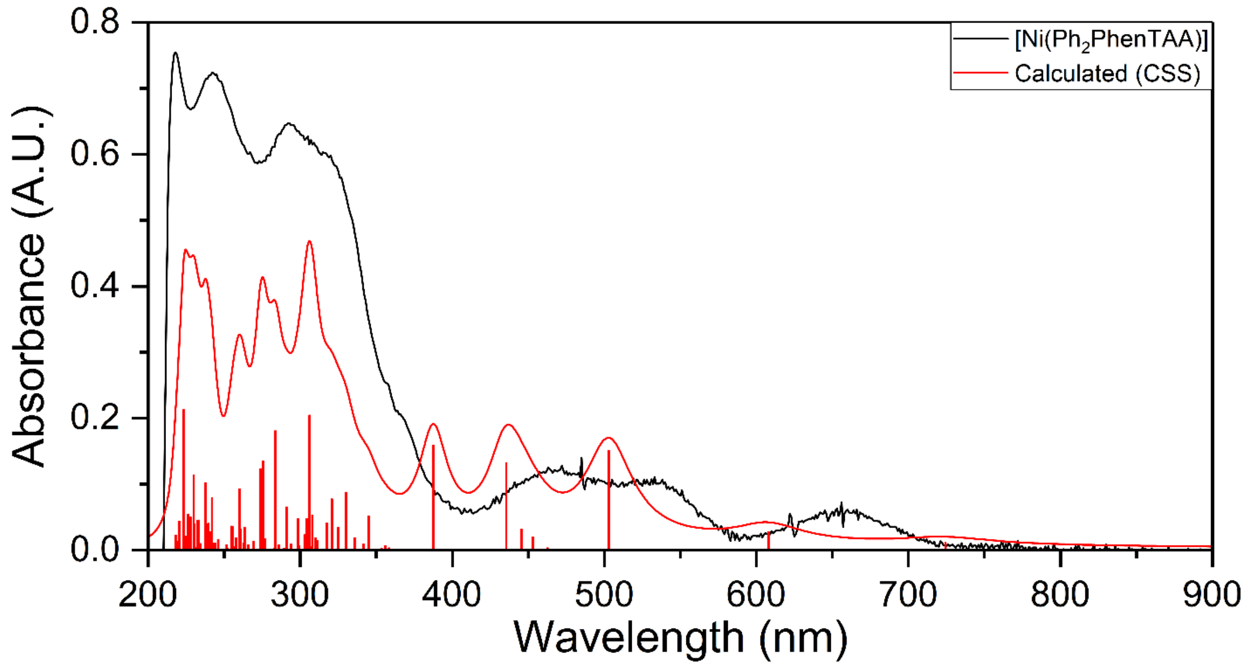

Figure S120. Experimental UV/Vis spectrum of [Ni(Ph$_2$PhenTAA)] (**4c**) in CH_2Cl_2 measured in an OTTLE cell (black) and calculated spectrum (red; Lorentzian peak FWHM: 1500 cm^{-1}). Individual excitations illustrated in red bars.

The neutral [Ni(Ph$_2$PhenTAA)] (**4c**) complex features similar excitations as for the other neutral PhenTAA complexes, revolving mainly around donor ↔ acceptor excitations. Due to the additional phenyl rings, additional delocalization is clearly observed for the acceptor moiety.

Table S38. Selected states for the TD–DFT calculated absorptions of [Ni(Ph$_2$PhenTAA)] (**4c**) at the B3LYP/def2-TZVPP CPCMC (CH_2Cl_2) level of theory.

State	Energy (cm^{-1})	Wavelength (nm)	fosc	T2 (au**2)	TX (au)	TY (au)	TZ (au)
2	16450.4	607.9	0.042119585	0.84291	0.44410	-0.05550	-0.80163
4	19877.2	503.1	0.226858577	3.75730	-0.96411	0.00806	1.68159
8	22969.3	435.4	0.199007973	2.85233	-0.83035	0.02739	1.47040
9	25802.3	387.6	0.238388314	3.04160	-1.46962	0.41366	-0.84303
28	32670.0	306.1	0.307166667	3.09528	-1.48408	0.42016	-0.84631
94	44757.8	223.4	0.319848210	2.35261	0.77472	-0.00945	-1.32376

Table S39. Most contributing orbital pairs to the selected states listed in **Table S38** and their respective fractions.

State	Donating orbital → Accepting orbital	Fraction of total state	Donating orbital	Accepting orbital
2	HOMO-1 → LUMO	0.78	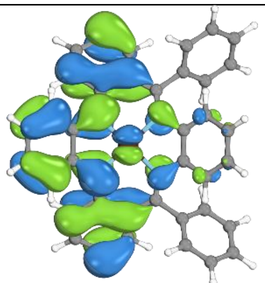	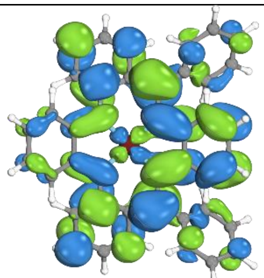
4	HOMO → LUMO+1	0.86	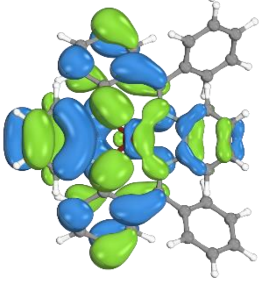	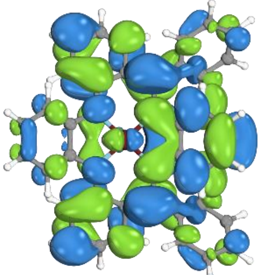
8	HOMO-1 → LUMO	0.20	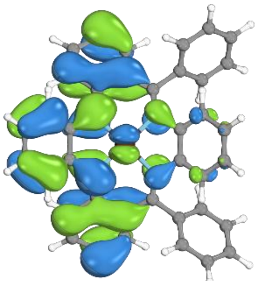	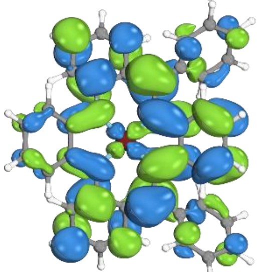
	HOMO-1 → LUMO+2	0.20	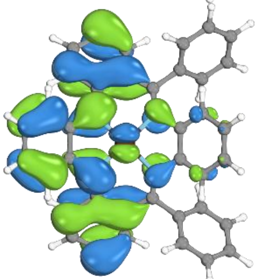	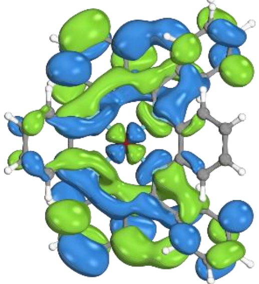
	HOMO → LUMO+1	0.08	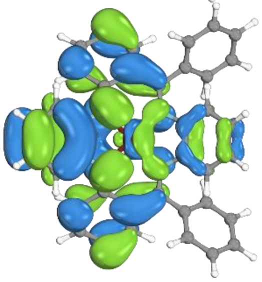	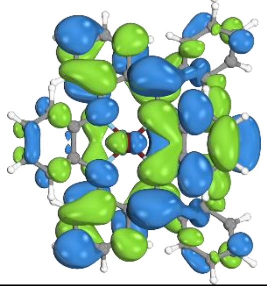

	HOMO-7 → LUMO+2	0.06	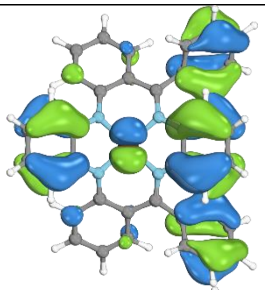	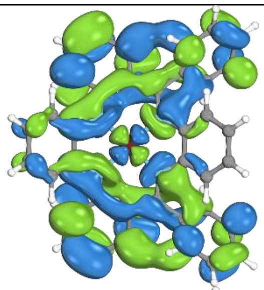
9	HOMO-2 → LUMO	0.58	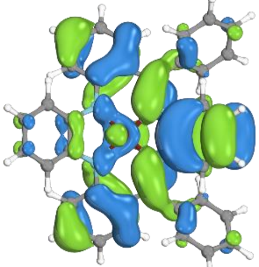	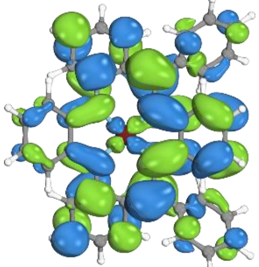
28	HOMO → LUMO+9	0.68	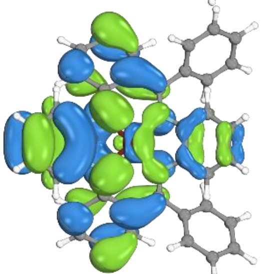	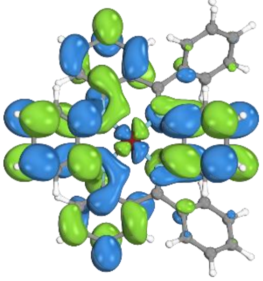
94	HOMO-5 → LUMO+4	0.42	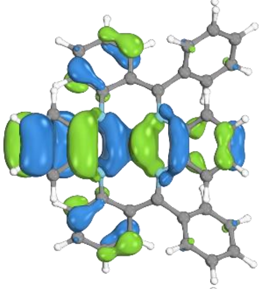	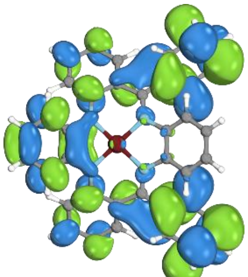
	HOMO-1 → LUMO+13	0.19	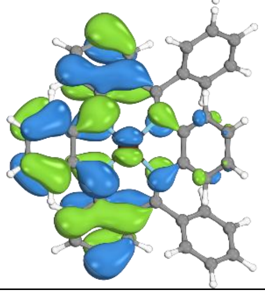	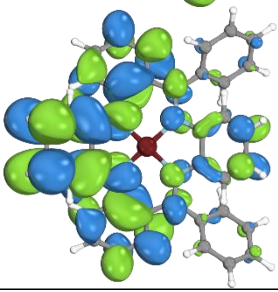

[Ni(Ph₂PhenTAA)]⁺ (Doublet):

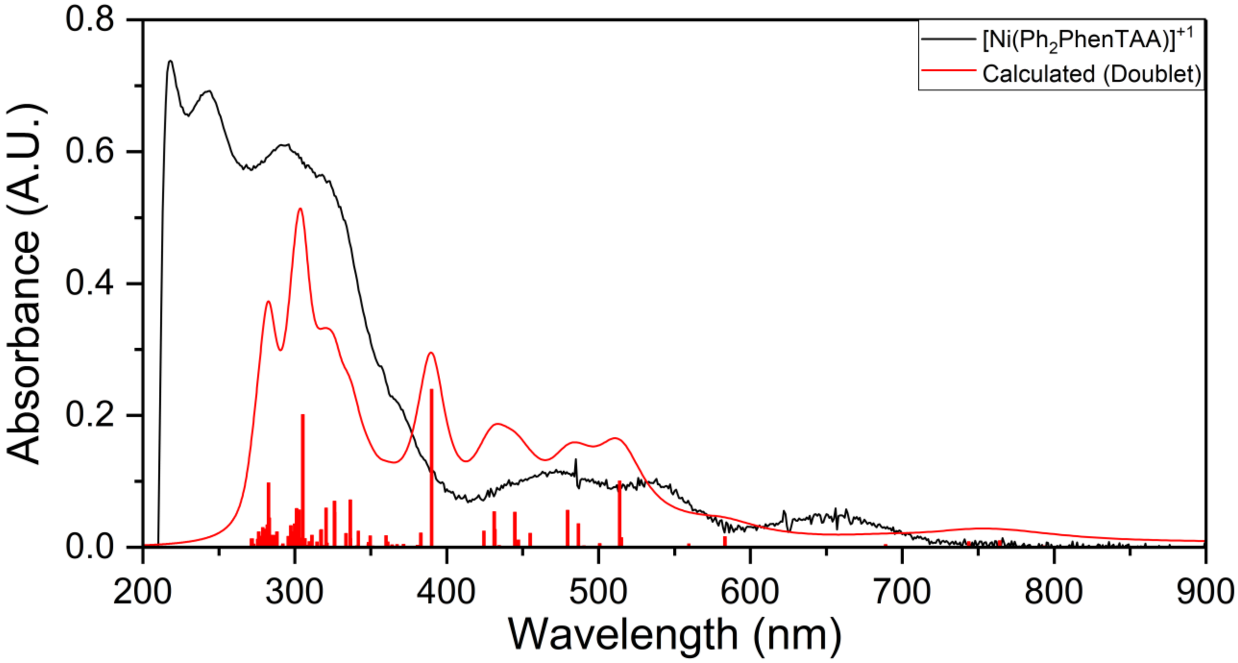

Figure S121. Experimental UV/Vis spectrum of $[Ni(Ph_2PhenTAA)]^+$ (**4c⁺**) in CH_2Cl_2 measured via spectro-electrochemistry (black) and calculated spectrum (red; Lorentzian peak FWHM: 1500 cm^{-1}). Individual excitations illustrated in red bars.

In line with previous PhenTAA complexes, the singly oxidized $[Ni(Ph_2PhenTAA)]^+$ (**4c⁺**) features mostly donor ↔ acceptor excitations as well. The theoretical spectrum is in reasonable agreement with the experimental data.

Table S40. Selected states for the TD–DFT calculated absorptions of $[Ni(Ph_2PhenTAA)]^+$ (**4c⁺**) at the B3LYP/def2-TZVPP CPCMC (CH_2Cl_2) level of theory.

State	Energy (cm^{-1})	Wavelength (nm)	fosc	T2 (au**2)	TX (au)	TY (au)	TZ (au)
7	14513.5	689.0	0.005682589	0.12890	0.30277	-0.08471	0.17336
13	19457.7	513.9	0.126159213	2.13454	0.71777	-0.27912	-1.24155
18	20851.6	479.6	0.070294600	1.10983	-0.36872	0.46547	0.87018
29	25636.6	390.1	0.299595032	3.84724	-1.65281	0.46503	-0.94826
64	32760.5	305.2	0.251841786	2.53077	-0.84366	-0.68678	1.16075

Table S41. Most contributing orbital pairs to the selected states listed in Table S40 and their respective fractions.

State	Donating orbital → Accepting orbital	Fraction of total state	Donating orbital	Accepting orbital
7	HOMO (α) → LUMO (α)	0.49	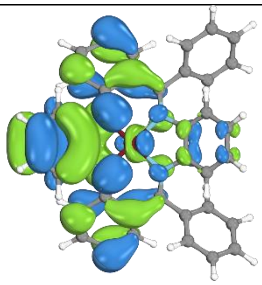	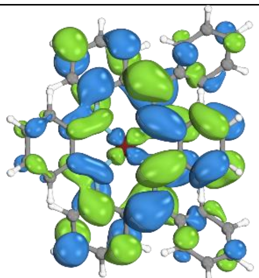
13	HOMO-1 (α) → LUMO (α)	0.31	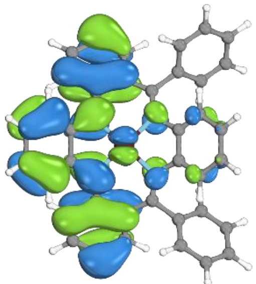	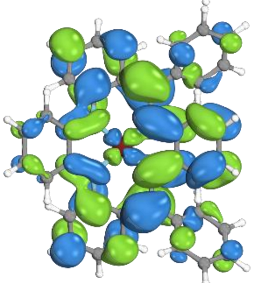
	HOMO-4 (β) → LUMO (β)	0.20	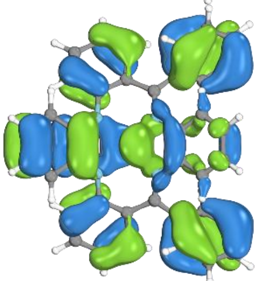	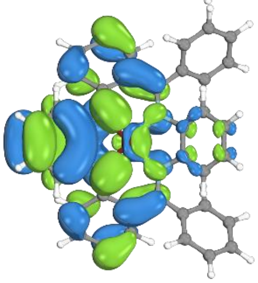
18	HOMO (α) → LUMO+1 (α)	0.30	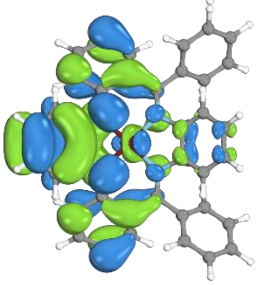	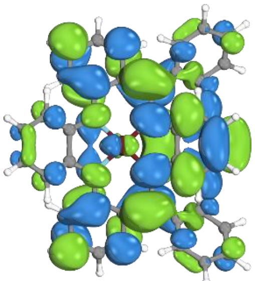
	HOMO-10 (β) → LUMO (β)	0.29	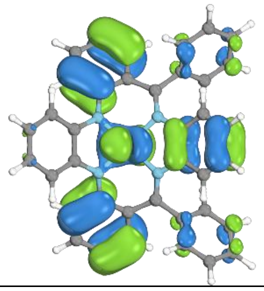	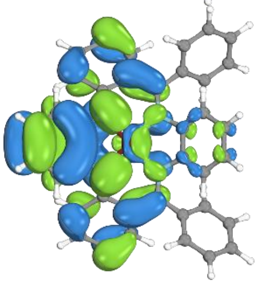

29	HOMO-2 (α) → LUMO (α) + HOMO-1 (β) → LUMO+1 (β)	0.25 (0.24)	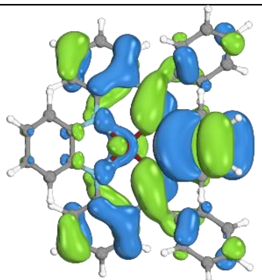	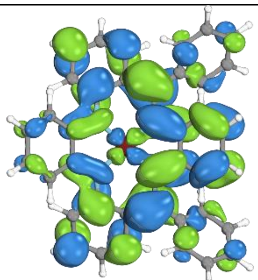
64	HOMO-8 (β) → LUMO+1 (β)	0.33	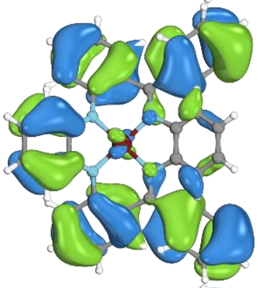	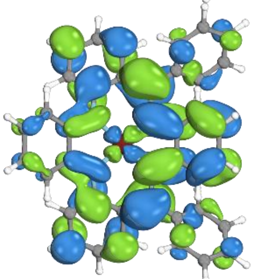
	HOMO-8 (α) → LUMO (α)	0.30	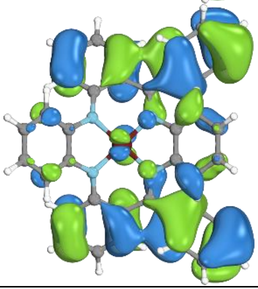	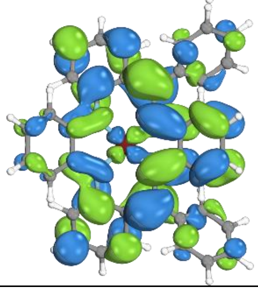

[Ni(Ph$_2$PhenTAA)]$^{2+}$ (CSS):

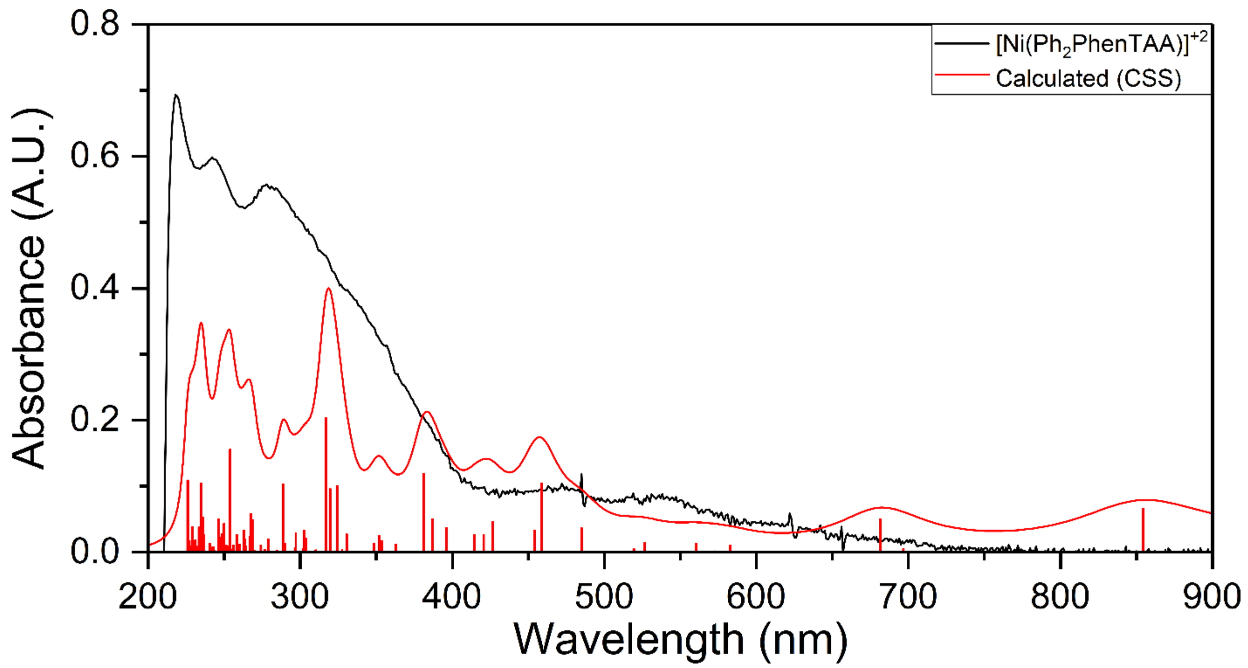

Figure S122. Experimental UV/Vis spectrum of [Ni(Ph$_2$PhenTAA)]$^{2+}$ (**4c^{2+}**) in CH_2Cl_2 measured via spectro-electrochemistry (black) and calculated spectrum (red; Lorentzian peak FWHM: 1500 cm^{-1}). Individual excitations illustrated in red bars.

Like the other two doubly oxidized PhenTAA complexes, the donor ↔ acceptor relation is inversed with the diaryl-*o*-phenylene diamine moiety now serving as an additional acceptor group. Furthermore, the spectrum features several $\sigma \rightarrow \pi$ excitations that were not so prevalent in the other PhenTAA complexes. Although the experimental spectrum is highly broadened, the theoretical spectrum is a reasonable match.

Table S42. Selected states for the TD–DFT calculated absorptions of [Ni(Ph$_2$PhenTAA)]$^{2+}$ (**4c^{2+}**) at the B3LYP/def2-TZVPP CPCMC (CH_2Cl_2) level of theory.

State	Energy (cm^{-1})	Wavelength (nm)	fosc	T2 (au**2)	TX (au)	TY (au)	TZ (au)
4	14674.6	681.4	0.095146773	2.13453	1.23094	-0.34699	0.70633
9	18990.7	526.6	0.028765803	0.49867	0.59716	-0.16707	0.33786
13	21800.4	458.7	0.196145148	2.96202	-1.44863	0.40448	-0.83659
21	26227.8	381.3	0.224664455	2.82000	-1.41486	0.39665	-0.81292
33	31560.6	316.9	0.382007882	3.98477	1.06428	0.50005	-1.61308
64	39385.8	253.9	0.292495676	2.44487	0.28953	-1.11926	-1.05276

Table S43. Most contributing orbital pairs to the selected states listed in Table S42 and their respective fractions.

State	Donating orbital → Accepting orbital	Fraction of total state	Donating orbital	Accepting orbital
4	HOMO-2 → LUMO	0.91	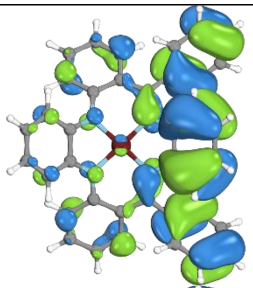	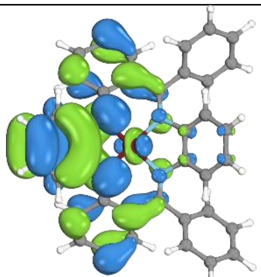
9	HOMO-7 → LUMO	0.67	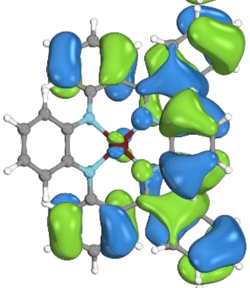	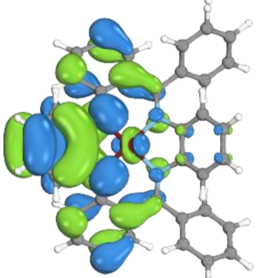
13	HOMO-9 → LUMO	0.03	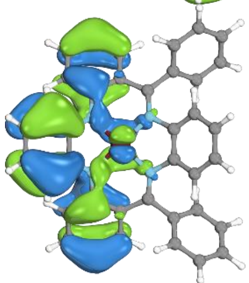	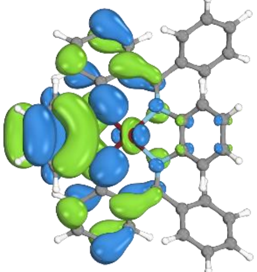
	HOMO-1 → LUMO+1	0.23	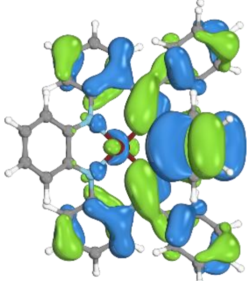	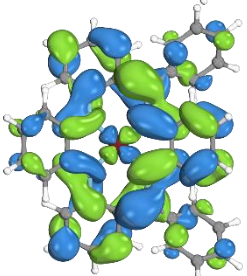
21	HOMO-14 → LUMO+3	0.25	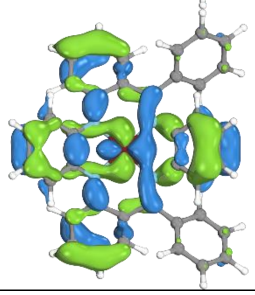	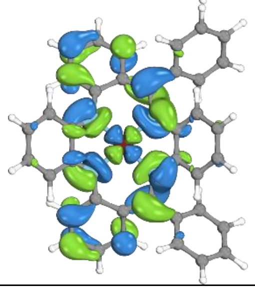

	HOMO-14 → LUMO+1	0.19	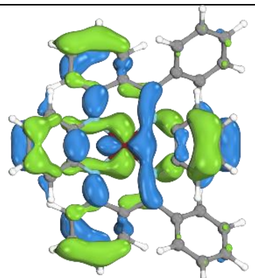	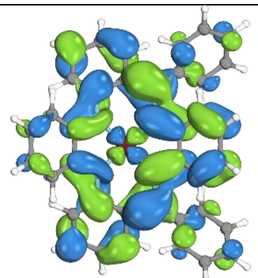
	HOMO-1 → LUMO+1	0.17	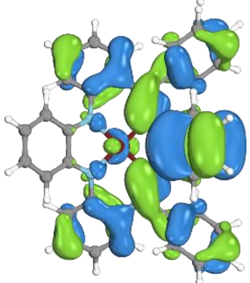	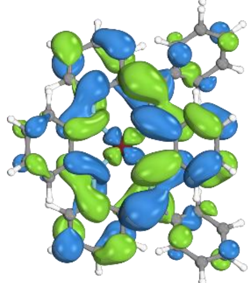
33	HOMO-15 → LUMO	0.49	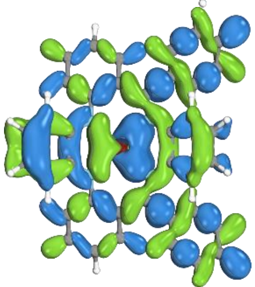	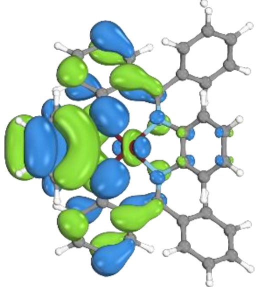
	HOMO-7 → LUMO+1	0.20	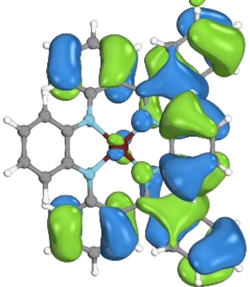	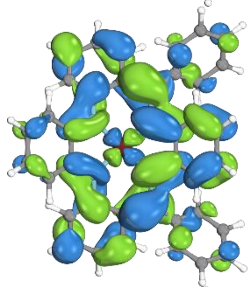
64	HOMO → LUMO+5	0.35	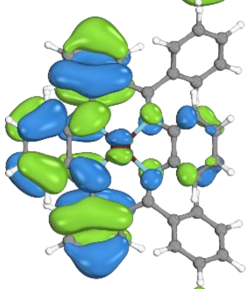	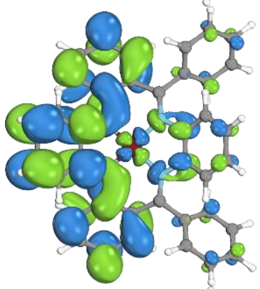
	HOMO-24 → LUMO	0.17	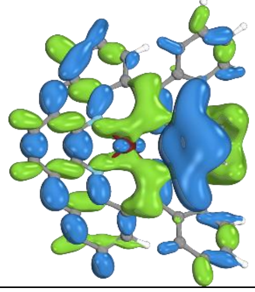	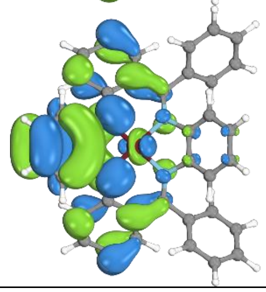

	HOMO-21 → LUMO	0.15	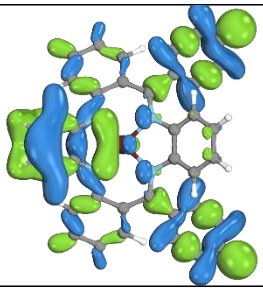	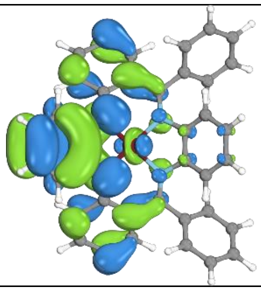

Mulliken spin density plots

Table S44. Mulliken spin density plots for all complexes in six oxidation states and all relevant spin states.

Ligand: +1.90 Nickel: +0.17	**[Ni(H$_2$PhenTAA)]$^{2-}$ (Triplet)** 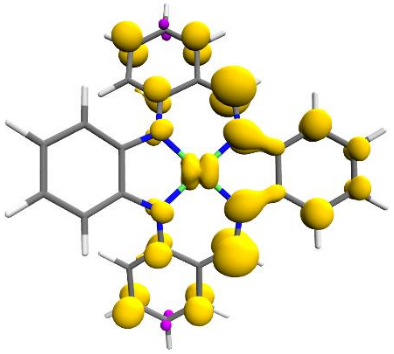	Ligand: +2.89 Nickel: +1.17	**[Ni(H$_2$PhenTAA)]$^{2-}$ (Quintet)** 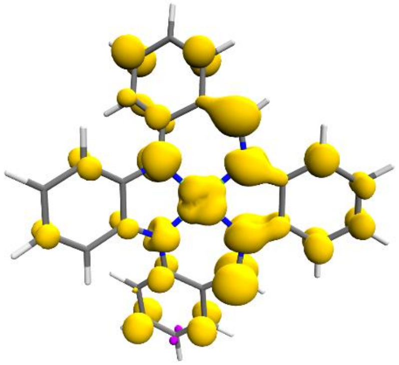
Ligand: +0.94 Nickel: +0.09	**[Ni(H$_2$PhenTAA)]$^{-}$ (Doublet)** 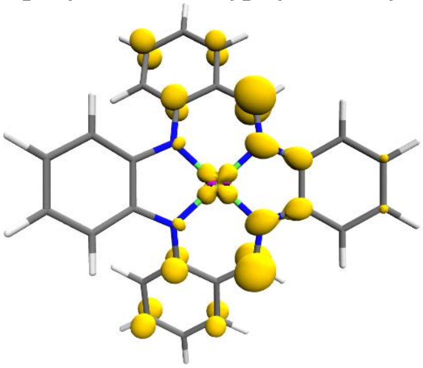	Ligand: +2.66 Nickel: +0.43	**[Ni(H$_2$PhenTAA)]$^{-}$ (Quartet)** 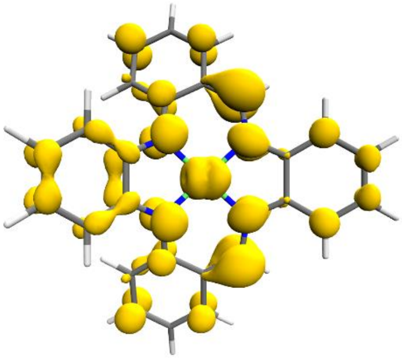
Ligand: +3.47 Nickel: +1.39	**[Ni(H$_2$PhenTAA)]$^{-}$ (Sextet)** 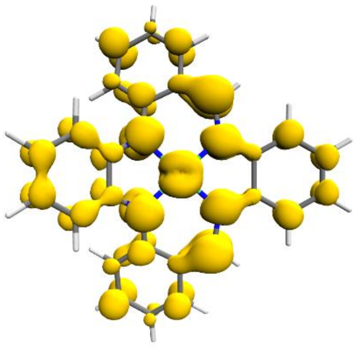	Ligand: +1.73 Nickel: +0.36	**[Ni(H$_2$PhenTAA)] (Triplet)** 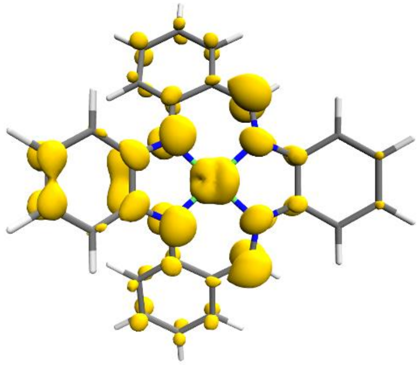

Ligand:<br>+3.16<br>Nickel:<br>+0.93	**[Ni(H_2PhenTAA)] (Quintet)**<br>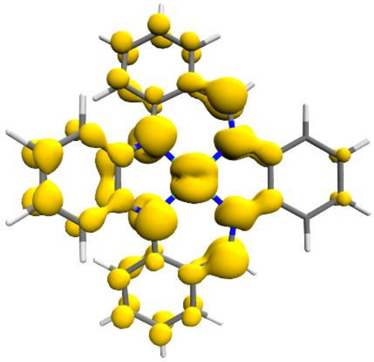	Ligand:<br>+0.91<br>Nickel:<br>+0.10	**[Ni(H_2PhenTAA)]$^{+}$ (Doublet)**<br>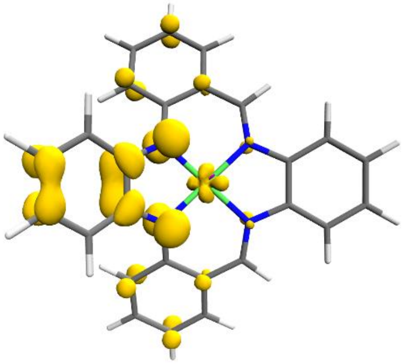
Ligand:<br>+2.10<br>Nickel:<br>+0.94	**[Ni(H_2PhenTAA)]$^{+}$ (Quartet)**<br>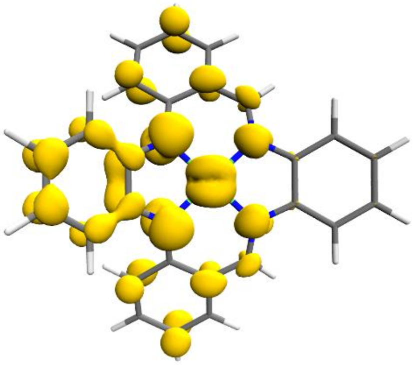	Ligand:<br>+4.13<br>Nickel:<br>+0.96	**[Ni(H_2PhenTAA)]$^{+}$ (Sextet)**<br>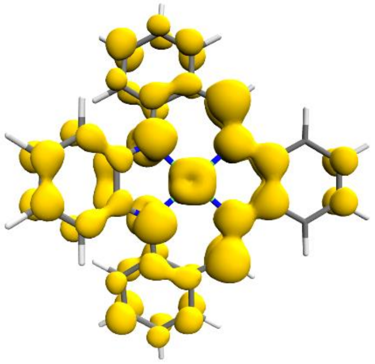
Ligand:<br>+1.75<br>Nickel:<br>+0.28	**[Ni(H_2PhenTAA)]$^{2+}$ (Triplet)**<br>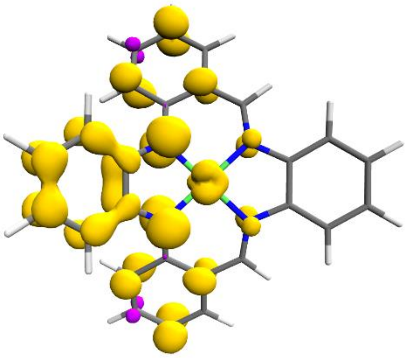	Ligand:<br>+3.20<br>Nickel:<br>+0.90	**[Ni(H_2PhenTAA)]$^{2+}$ (Quintet)**<br>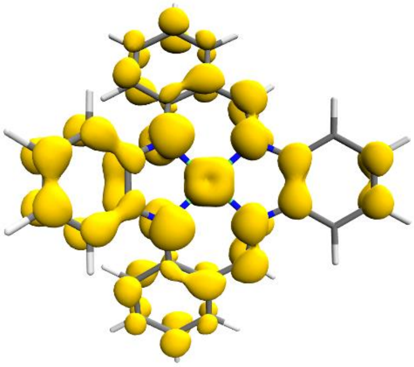
Ligand:<br>+0.91<br>Nickel:<br>+0.15	**[Ni(H_2PhenTAA)]$^{3+}$ (Doublet)**<br>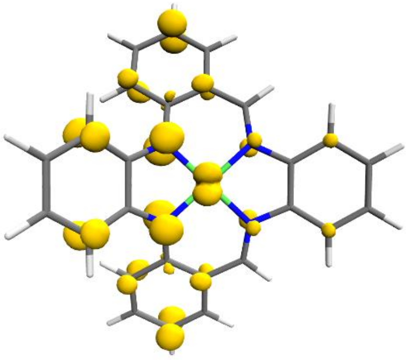	Ligand:<br>+2.74<br>Nickel:<br>+0.27	**[Ni(H_2PhenTAA)]$^{3+}$ (Quartet)**<br>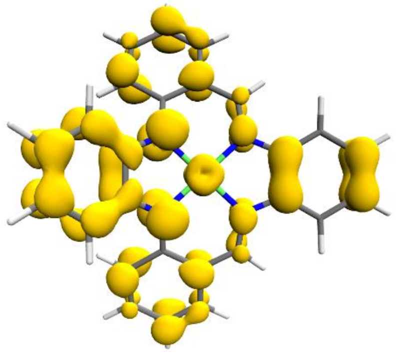

Ligand:<br>+3.91<br>Nickel:<br>+1.17	**[Ni(H_2PhenTAA)]$^{3+}$ (Sextet)**<br>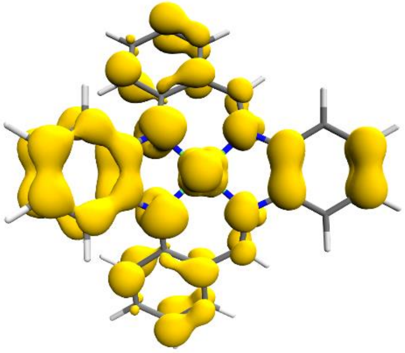	:	

Ligand:<br>+1.85<br>Nickel:<br>+0.19	**[Ni(Me_2PhenTAA)]$^{2-}$ (Triplet)**<br>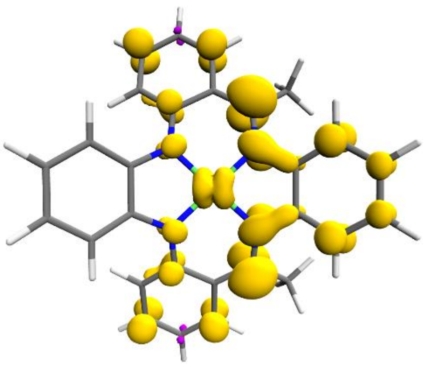	Ligand:<br>+2.84<br>Nickel:<br>+1.20	**[Ni(Me_2PhenTAA)]$^{2-}$ (Quintet)**<br>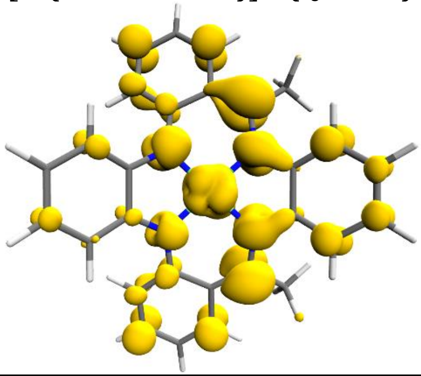
Ligand:<br>+0.86<br>Nickel:<br>+0.12	**[Ni(Me_2PhenTAA)]$^{-}$ (Doublet)**<br>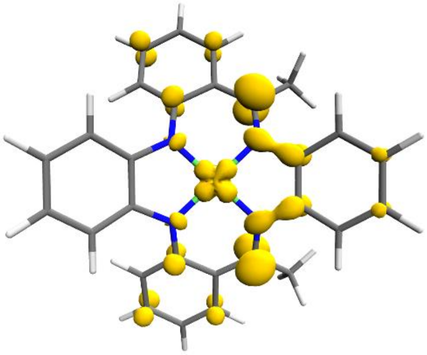	Ligand:<br>+2.59<br>Nickel:<br>+0.49	**[Ni(Me_2PhenTAA)]$^{-}$ (Quartet)**<br>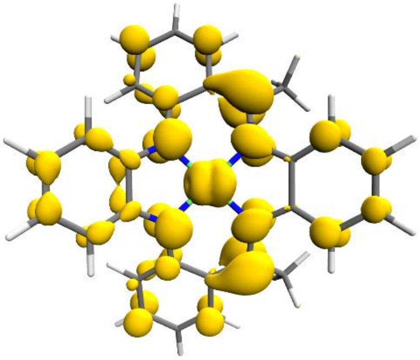
Ligand:<br>+3.69<br>Nickel:<br>+1.39	**[Ni(Me_2PhenTAA)]$^{-}$ (Sextet)**<br>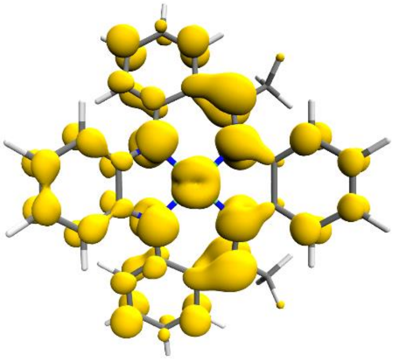	Ligand:<br>+1.60<br>Nickel:<br>+0.40	**[Ni(Me_2PhenTAA)] (Triplet)**<br>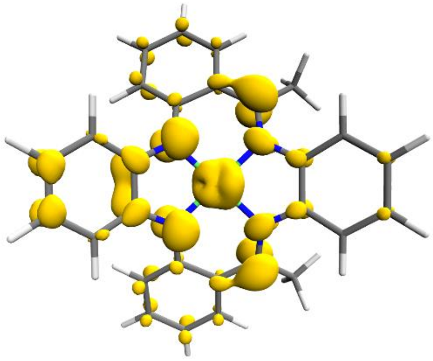

Ligand:<br>+3.12<br>Nickel:<br>+0.97	**$[Ni(Me_2PhenTAA)]$ (Quintet)**<br>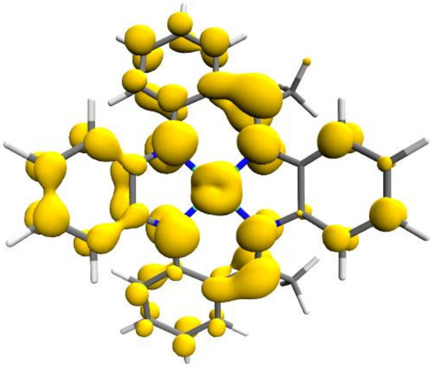	Ligand:<br>+0.90<br>Nickel:<br>+0.10	**$[Ni(Me_2PhenTAA)]^{+}$ (Doublet)**<br>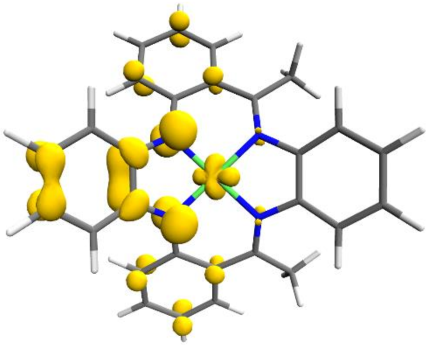
Ligand:<br>+2.12<br>Nickel:<br>+0.93	**$[Ni(Me_2PhenTA)]^{+}$ (Quartet)**<br>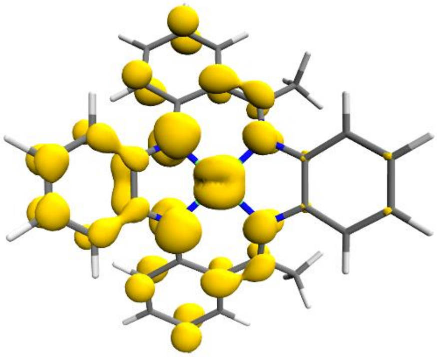	Ligand:<br>+4.10<br>Nickel:<br>+0.98	**$[Ni(Me_2PhenTAA)]^{+}$ (Sextet)**<br>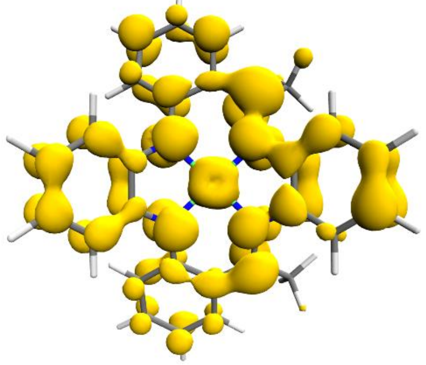
Ligand:<br>+1.74<br>Nickel:<br>+0.27	**$[Ni(Me_2PhenTAA)]^{2+}$ (Triplet)**<br>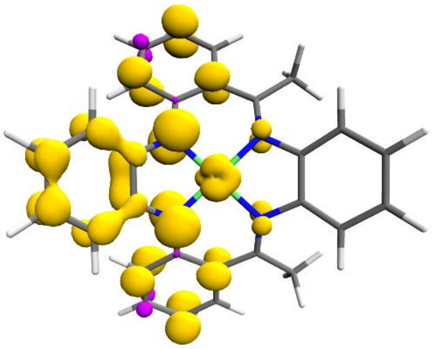	Ligand:<br>+3.13<br>Nickel:<br>+0.91	**$[Ni(Me_2PhenTAA)]^{2+}$ (Quintet)**<br>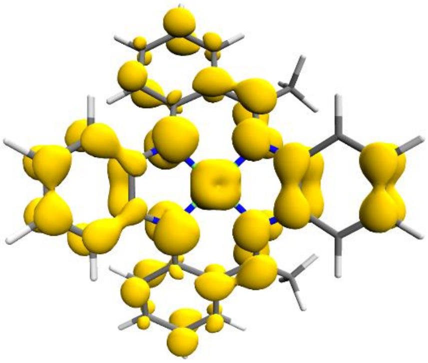
Ligand:<br>+0.92<br>Nickel:<br>+0.14	**$[Ni(Me_2PhenTAA)]^{3+}$ (Doublet)**<br>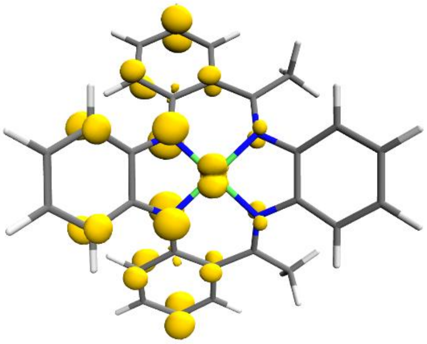	Ligand:<br>+2.78<br>Nickel:<br>+0.28	**$[Ni(Me_2PhenTAA)]^{3+}$ (Quartet)**<br>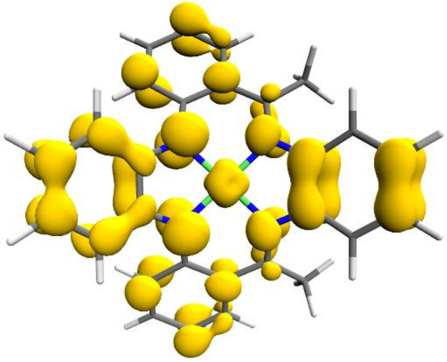

Ligand:<br>+3.67<br>Nickel:<br>+1.36	**$[Ni(Me_2PhenTAA)]^{3+}$ (Sextet)**<br>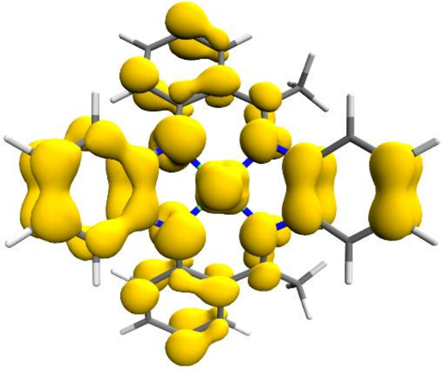		

Ligand: +1.90<br>Nickel: +0.17	**$[Ni(Ph_2PhenTAA)]^{2-}$ (Triplet)**<br>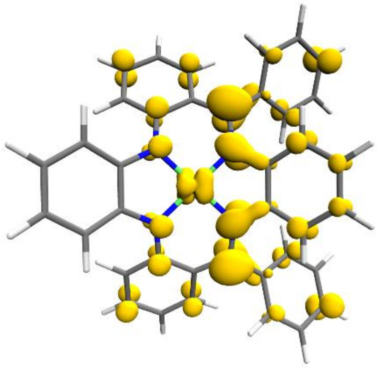	Ligand: +2.89<br>Nickel: +1.17	**$[Ni(Ph_2PhenTAA)]^{2-}$ (Quintet)**<br>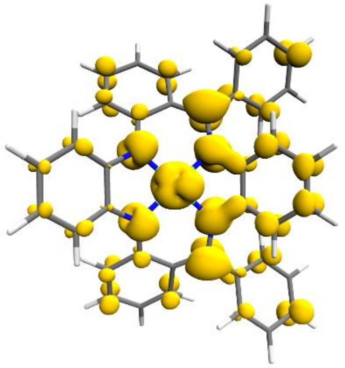
Ligand: +0.94<br>Nickel: +0.09	**$[Ni(Ph_2PhenTAA)]^{-}$ (Doublet)**<br>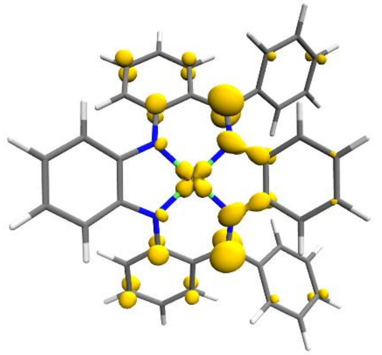	Ligand: +2.66<br>Nickel: +0.43	**$[Ni(Ph_2PhenTAA)]^{-}$ (Quartet)**<br>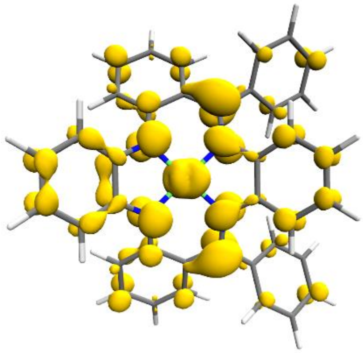
Ligand: +3.74<br>Nickel: +1.39	**$[Ni(Ph_2PhenTAA)]^{-}$ (Sextet)**<br>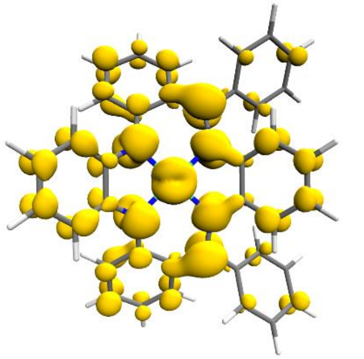	Ligand: +1.73<br>Nickel: +0.36	**$[Ni(Ph_2PhenTAA)]$ (Triplet)**<br>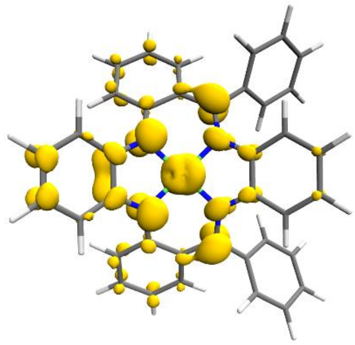

Ligand: +3.16<br>Nickel: +0.93	**[Ni(Ph_2PhenTAA)] (Quintet)**<br>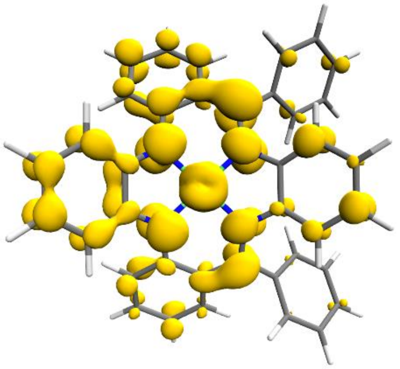	Ligand: +0.91<br>Nickel: +0.10	**[Ni(Ph_2PhenTAA)]$^+$ (Doublet)**<br>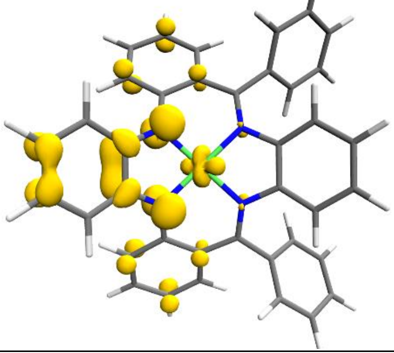
Ligand: +2.10<br>Nickel: +0.94	**[Ni(Ph_2PhenTA)]$^+$ (Quartet)**<br>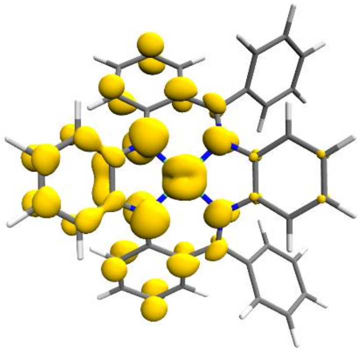	Ligand: +4.13<br>Nickel: +0.96	**[Ni(Ph_2PhenTAA)]$^+$ (Sextet)**<br>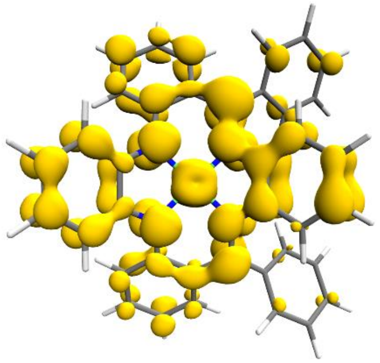
Ligand: +1.75<br>Nickel: +0.28	**[Ni(Ph_2PhenTAA)]$^{2+}$ (Triplet)**<br>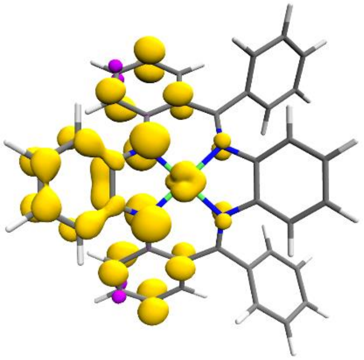	Ligand: +3.20<br>Nickel: +0.90	**[Ni(Ph_2PhenTAA)]$^{2+}$ (Quintet)**<br>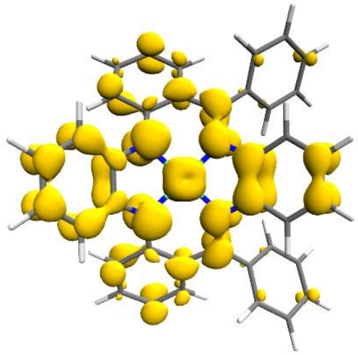
Ligand: +0.91<br>Nickel: +0.15	**[Ni(Ph_2PhenTAA)]$^{3+}$ (Doublet)**<br>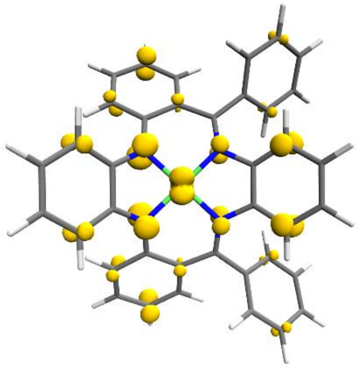	Ligand: +2.74<br>Nickel: +0.27	**[Ni(Ph_2PhenTAA)]$^{3+}$ (Quartet)**<br>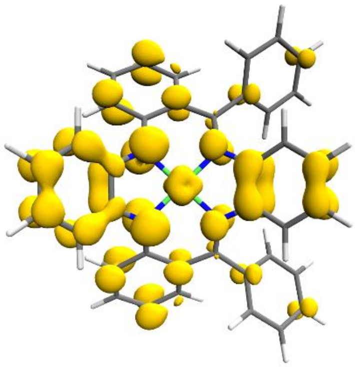

Ligand: +3.91<br>Nickel: +1.17	**[Ni(Ph_2PhenTAA)]$^{3+}$ (Sextet)**<br>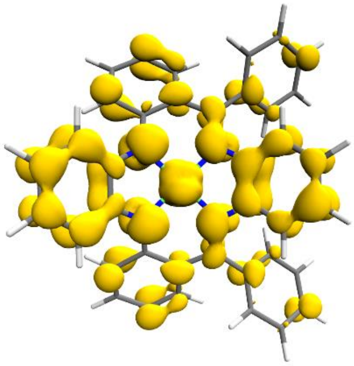		

List of xyz coordinates for all reported structures:

BP86/def2-TZVP/disp3:
$NiH_2PhenTAA$:
$NiH_2PhenTAA$_2eRED_CSS:
49 atoms
C -0.8380184 2.0860283 1.9735762
C 0.1101266 2.5824450 2.8832392
C 0.3901827 1.8733633 4.0548019
C -0.2565604 0.6529322 4.3497087
C -1.2324377 0.1422949 3.4140391
C -1.4992658 0.8841922 2.2421441
N -1.9092769 -0.9814466 3.8510895
N -0.1616452 -0.0674391 5.5261105
C 0.9341943 -0.0693498 6.3544556
C -2.5366024 -1.8849841 3.0282864
C -3.5584262 -2.7764170 3.5742310
C -4.2256346 -3.6477806 2.6560240
C -3.8986084 -3.7315986 1.3134768
C -2.8367194 -2.9432019 0.8129786
C -2.1755521 -2.0635061 1.6679093
C 2.2404352 0.2465872 5.8996593
C 3.3499267 0.2936352 6.7415649
C 3.1957458 -0.0198672 8.1118759
C 1.9533008 -0.4150610 8.5772477
C 0.7929596 -0.4999528 7.7441793
C -3.8748566 -2.8722124 4.9450119
C -0.3875999 -1.0476920 8.2868065
N -1.5341705 -1.2667825 7.6070287
N -3.2762475 -2.1785542 5.9375928
C -3.6644272 -2.2442085 7.2504485
C -2.6845865 -1.7314096 8.1893633
C -2.9990538 -1.7249755 9.5668271
C -4.2208226 -2.2134746 10.0281875
C -5.1764269 -2.7134439 9.1125835
C -4.8994189 -2.7192185 7.7460295
Ni -1.6847091 -1.1851390 5.7268296
H -0.3246017 -1.4285419 9.3150563
H -4.5865637 -3.6585541 5.2307329
H -1.0805310 2.6448455 1.0650888
H 0.6161354 3.5333801 2.6931216
H 1.0880398 2.2861941 4.7836375
H -2.2715858 0.5274669 1.5602352
H -5.0205876 -4.2853468 3.0586188
H -4.4459494 -4.4129614 0.6553007
H -2.5184679 -3.0318098 -0.2294097
H -1.3368040 -1.4802876 1.2859981
H 2.3693395 0.4584018 4.8375901
H 4.3314920 0.5520454 6.3347892
H 4.0485371 0.0316185 8.7953480
H 1.8361002 -0.6979829 9.6292789
H -2.2827599 -1.2992507 10.2727709
H -4.4492947 -2.1823991 11.0973043
H -6.1460477 -3.0699537 9.4716294
H -5.6572553 -3.0646271 7.0396021

$NiH_2PhenTAA$_2eRED_Triplet:
49 atoms
C -0.7658653 2.0446261 1.9251494
C 0.1786467 2.5392305 2.8314454
C 0.4432037 1.8390150 4.0165529
C -0.2239737 0.6347661 4.3215814
C -1.1961651 0.1259386 3.3893111
C -1.4444805 0.8505546 2.2052866
N -1.8834578 -0.9923834 3.8422974
N -0.1465876 -0.0837415 5.5072737
C 0.9388031 -0.0661313 6.3541371
C -2.5373585 -1.8846805 3.0226708
C -3.5830849 -2.7387305 3.5729717
C -4.2877591 -3.5911444 2.6680526
C -3.9754857 -3.6889353 1.3191609
C -2.9030973 -2.9306433 0.8097747
C -2.2048215 -2.0643101 1.6575760
C 2.2472558 0.2643278 5.9238408
C 3.3409344 0.3357580 6.7931176
C 3.1636517 0.0462450 8.1603830
C 1.9102442 -0.3480811 8.6076467
C 0.7738160 -0.4591218 7.7484104
C -3.9158416 -2.7912199 4.9451818
C -0.4300965 -0.9672513 8.2857225
N -1.5417483 -1.2703809 7.5811696
N -3.2524995 -2.1659673 5.9416805
C -3.6557895 -2.2600409 7.2642783
C -2.6884682 -1.7536234 8.1912827
C -2.9657782 -1.7905938 9.5740975
C -4.1843742 -2.3283000 10.0389249
C -5.1277131 -2.8221192 9.1349681
C -4.8713689 -2.7880592 7.7481248
Ni -1.6735903 -1.1820403 5.7135717
H -0.4144590 -1.2411784 9.3477179
H -4.7122613 -3.4900553 5.2288180
H -0.9898488 2.5886534 1.0030019
H 0.7052631 3.4765129 2.6297320
H 1.1436834 2.2524412 4.7417419
H -2.2135365 0.4946753 1.5200972
H -5.0960252 -4.2035832 3.0818077
H -4.5467408 -4.3574717 0.6685717
H -2.6031269 -3.0213691 -0.2379110
H -1.3587532 -1.5031166 1.2609232
H 2.4001590 0.4626016 4.8629789
H 4.3266241 0.6032338 6.4023091
H 4.0016129 0.1153810 8.8600885
H 1.7701410 -0.6105970 9.6617129
H -2.2503266 -1.3746541 10.2843465
H -4.3899901 -2.3466069 11.1132953
H -6.0762863 -3.2291553 9.4974787
H -5.6282792 -3.1426736 7.0476473

$NiH_2PhenTAA$_2eRED_Quintet:
49 atoms
C -0.5770190 1.9103713 1.8433297
C 0.3332171 2.4265072 2.7734692
C 0.5597623 1.7328398 3.9692737
C -0.0936865 0.5186301 4.2611697
C -1.0476926 -0.0223040 3.2954583
C -1.2545175 0.7142456 2.1017040
N -1.7381391 -1.1329206 3.7108363
N -0.0297501 -0.1675323 5.4660176
C 0.9963277 -0.0264329 6.3945866
C -2.5592957 -1.9126590 2.9225712
C -3.6856734 -2.6274687 3.5299229
C -4.5352149 -3.3623381 2.6441239
C -4.2954217 -3.5010760 1.2819542
C -3.1553297 -2.8979108 0.7245064
C -2.3210196 -2.1242508 1.5443024
C 2.3156660 0.2945440 6.0141376
C 3.3574087 0.4867808 6.9370571
C 3.0785361 0.3689715 8.3077864
C 1.8033762 -0.0071917 8.7169846
C 0.7356247 -0.2894093 7.8091500
C -4.0098549 -2.6849861 4.9173365
C -0.4666040 -0.8222277 8.3566255
N -1.4949843 -1.3263002 7.6512383
N -3.2787837 -2.2034691 5.9454867
C -3.6147019 -2.3617522 7.2744149
C -2.6275720 -1.8756762 8.2214595
C -2.8869787 -2.0066387 9.6035027
C -4.0792152 -2.5939592 10.0692024
C -5.0313822 -3.0581395 9.1572164
C -4.8010873 -2.9469010 7.7729326
Ni -1.5647103 -1.2818644 5.6853290
H -0.4965684 -0.8720621 9.4554220
H -4.9165399 -3.2636703 5.1501532
H -0.7742594 2.4456584 0.9091276
H 0.8558990 3.3676215 2.5818373
H 1.2235112 2.1595150 4.7214501
H -2.0000908 0.3589902 1.3912508
H -5.4025153 -3.8629037 3.0878031
H -4.9814603 -4.0847999 0.6609544
H -2.9139008 -3.0238395 -0.3346184
H -1.4250147 -1.6763299 1.1133509
H 2.5257703 0.3889818 4.9483643
H 4.3608753 0.7378540 6.5845332
H 3.8575080 0.5538025 9.0534218
H 1.6011932 -0.1444515 9.7844676
H -2.1542970 -1.6410046 10.3250054
H -4.2585872 -2.6766362 11.1453107
H -5.9634657 -3.5066852 9.5148350
H -5.5643418 -3.2935221 7.0742448

$NiH_2PhenTAA$_1eRED_Doublet:
49 atoms
C -0.7910200 2.0592857 1.9414589
C 0.1547229 2.5547895 2.8489875
C 0.4175764 1.8629302 4.0325042
C -0.2437279 0.6541567 4.3251543
C -1.2100731 0.1480797 3.3983248
C -1.4721272 0.8729445 2.2192485
N -1.9049454 -0.9674422 3.8566253
N -0.1730252 -0.0608023 5.5172275

C 0.9147565 -0.0678479 6.3458132
C -2.5309533 -1.8709513 3.0432461
C -3.5519606 -2.7516042 3.5791727
C -4.2295649 -3.6296861 2.6855931
C -3.9129348 -3.7182362 1.3437189
C -2.8527079 -2.9306352 0.8433727
C -2.1845230 -2.0443695 1.6736023
C 2.2253433 0.2631750 5.8998825
C 3.3159434 0.2966734 6.7547132
C 3.1618445 -0.0174095 8.1231024
C 1.9186451 -0.4135809 8.5772132
C 0.7773627 -0.4866100 7.7281749
C -3.8884474 -2.8176272 4.9522448
C -0.4211689 -1.0035038 8.2751016
N -1.5371679 -1.2598495 7.5886252
N -3.2634101 -2.1637158 5.9340906
C -3.6557212 -2.2568118 7.2645745
C -2.6902634 -1.7512397 8.1898746
C -2.9808269 -1.7644340 9.5665226
C -4.1923131 -2.2779681 10.0270676
C -5.1420000 -2.7752719 9.1169487
C -4.8783566 -2.7580309 7.7480951
Ni -1.6975974 -1.1499031 5.7212202
H -0.3939046 -1.2831905 9.3347586
H -4.6792984 -3.5249678 5.2279058
H -1.0236647 2.6128377 1.0294912
H 0.6669699 3.4987292 2.6519150
H 1.1040834 2.2857195 4.7650053
H -2.2532005 0.5270468 1.5432355
H -5.0178047 -4.2643245 3.1007785
H -4.4528605 -4.4057928 0.6905321
H -2.5396878 -3.0287938 -0.1984938
H -1.3466419 -1.4714236 1.2789205
H 2.3701059 0.4739365 4.8412516
H 4.3011280 0.5501453 6.3568757
H 4.0140500 0.0230458 8.8035565
H 1.7897783 -0.7036825 9.6239835
H -2.2678500 -1.3439819 10.2764708
H -4.4106689 -2.2706375 11.0965915
H -6.0996298 -3.1549672 9.4780427
H -5.6372624 -3.1082022 7.0476775

NiH$_2$PhenTAA_1eRED_Quartet:
49 atoms
C -0.8908532 2.0838051 2.0262643
C 0.0612384 2.5818688 2.9397325
C 0.3724052 1.8722730 4.0938220
C -0.2601331 0.6355675 4.3693036
C -1.2420468 0.1224242 3.4281073
C -1.5344509 0.8749762 2.2645562
N -1.8341899 -1.0402968 3.8333500
N -0.1111569 -0.1409281 5.4836876
C 0.9794188 -0.1030709 6.3431969
C -2.5077155 -1.9259256 3.0021328
C -3.5401910 -2.7748950 3.5590861
C -4.2267820 -3.6380251 2.6590048
C -3.9024625 -3.7294036 1.3126282
C -2.8412222 -2.9647391 0.8001913
C -2.1552671 -2.0912650 1.6493364
C 2.2717962 0.2237516 5.8911548
C 3.3710684 0.2857044 6.7529437
C 3.1948570 -0.0145329 8.1139379
C 1.9457939 -0.4074027 8.5741936
C 0.8071702 -0.5011647 7.7247789
C -3.8733531 -2.8361301 4.9405993
C -0.4004968 -1.0200022 8.2681349
N -1.5250584 -1.2668307 7.5776070
N -3.2515361 -2.1671973 5.9243688
C -3.6605136 -2.2226289 7.2508822
C -2.6945722 -1.7184087 8.1758180
C -2.9902842 -1.7053199 9.5508513
C -4.2232396 -2.1999700 10.0092114
C -5.1670922 -2.6927427 9.1053452
C -4.8950467 -2.6998455 7.7265574
Ni -1.6063012 -1.2650132 5.6882743
H -0.3664511 -1.3329322 9.3185546
H -4.6377061 -3.5694028 5.2246477
H -1.1449318 2.6621904 1.1358071
H 0.5415629 3.5446019 2.7540872
H 1.0720403 2.2828689 4.8205736
H -2.3039377 0.5173297 1.5816809
H -5.0302164 -4.2594036 3.0636053
H -4.4596776 -4.4077404 0.6631889
H -2.5458467 -3.0494093 -0.2468373
H -1.3105006 -1.5199664 1.2641795
H 2.4097914 0.4246104 4.8286351
H 4.3527431 0.5602612 6.3635932
H 4.0374545 0.0410166 8.8063070
H 1.8182765 -0.6740570 9.6267989
H -2.2734387 -1.2885027 10.2583915
H -4.4445526 -2.1847388 11.0784203
H -6.1288359 -3.0639012 9.4654744
H -5.6505566 -3.0514572 7.0238334

NiH$_2$PhenTAA_1eRED_Sextet:
49 atoms
C -0.6980970 1.9284019 1.9148161
C 0.2489515 2.4243330 2.8234301
C 0.5357838 1.7236042 3.9943550
C -0.1010629 0.4958150 4.2839959
C -1.0861132 -0.0198426 3.3393614
C -1.3620979 0.7298630 2.1736162
N -1.7501502 -1.1498335 3.7545382
N 0.0168987 -0.2250490 5.4487004
C 1.0399180 -0.0660824 6.3774098
C -2.5486225 -1.9436629 2.9380570
C -3.6802138 -2.6504966 3.5266762
C -4.5122109 -3.3693927 2.6144841
C -4.2191555 -3.5030633 1.2652258
C -3.0585357 -2.9145307 0.7335572
C -2.2523033 -2.1410713 1.5763112
C 2.3507200 0.2668210 5.9875231
C 3.3863679 0.4567651 6.9093857
C 3.1009379 0.3263949 8.2795907
C 1.8316680 -0.0501169 8.6937192
C 0.7636846 -0.3251945 7.7855523
C -4.0210300 -2.7014055 4.9064334
C -0.4482945 -0.8317041 8.3305077
N -1.5001645 -1.3007817 7.6252736
N -3.2727469 -2.2286770 5.9264598
C -3.6167351 -2.3503134 7.2616248
C -2.6396047 -1.8386874 8.1981240
C -2.9067137 -1.9295574 9.5782171
C -4.1000354 -2.5041551 10.0463450
C -5.0429393 -2.9978887 9.1426230
C -4.8032771 -2.9225674 7.7605185
Ni -1.5699606 -1.3073130 5.6739262
H -0.4757513 -0.8993326 9.4251141
H -4.9482642 -3.2397636 5.1387242
H -0.9374993 2.4917202 1.0107705
H 0.7492180 3.3751023 2.6291294
H 1.2237657 2.1438951 4.7262081
H -2.1414706 0.3819203 1.4974894
H -5.3984492 -3.8636665 3.0217342
H -4.8840943 -4.0866143 0.6242272
H -2.7870686 -3.0505940 -0.3139724
H -1.3418863 -1.6893943 1.1809761
H 2.5603682 0.3517438 4.9207658
H 4.3910360 0.7037910 6.5642166
H 3.8818297 0.4991747 9.0237949
H 1.6340079 -0.1837642 9.7607896
H -2.1872980 -1.5351530 10.2960962
H -4.2882513 -2.5560421 11.1209258
H -5.9741256 -3.4386934 9.5051553
H -5.5559326 -3.2989404 7.0674961

NiH$_2$PhenTAA_0eNeutral_CSS:
49 atoms
C -0.7669183 2.0423968 1.9272053
C 0.1775835 2.5364234 2.8320918
C 0.4374476 1.8509778 4.0200030
C -0.2251161 0.6452533 4.3018354
C -1.1841537 0.1433955 3.3827919
C -1.4554382 0.8606811 2.2062684
N -1.8817474 -0.9772901 3.8559491
N -0.1652889 -0.0787337 5.5013319
C 0.9087477 -0.0768600 6.3358841
C -2.5184040 -1.8700588 3.0512550
C -3.5413047 -2.7414009 3.5827140
C -4.2399806 -3.6196329 2.7091153
C -3.9329089 -3.7137828 1.3694233
C -2.8761002 -2.9236649 0.8636211
C -2.1986184 -2.0307329 1.6695850
C 2.2217096 0.2809831 5.9055958
C 3.2932368 0.3026209 6.7757285
C 3.1353324 -0.0164869 8.1433124
C 1.8919650 -0.4112504 8.5860421
C 0.7701765 -0.4850564 7.7150730
C -3.8923134 -2.7695970 4.9536925
C -0.4460182 -0.9654310 8.2569453
N -1.5244219 -1.2711183 7.5632023
N -3.2343916 -2.1669212 5.9240532
C -3.6451280 -2.2674900 7.2736422
C -2.6923460 -1.7681147 8.1869617

C -2.9608791 -1.7943738
9.5606854
C -4.1698365 -2.3201623
10.0205284
C -5.1139980 -2.8152423
9.1154641
C -4.8567759 -2.7883441
7.7433730
Ni -1.6933874 -1.1387425
5.7109562
H -0.4653266 -1.1276228
9.3395829
H -4.7632168 -3.3774392
5.2200644
H -1.0004287 2.5977271
1.0181177
H 0.6869505 3.4803013
2.6345941
H 1.1137521 2.2829796
4.7555243
H -2.2456563 0.5256003
1.5366405
H -5.0252269 -4.2482855
3.1355904
H -4.4705696 -4.4043148
0.7203379
H -2.5718617 -3.0304063 -
0.1792458
H -1.3649349 -1.4646747
1.2592324
H 2.3834811 0.4948944
4.8511416
H 4.2828579 0.5534637
6.3894675
H 3.9853765 0.0186987
8.8239479
H 1.7502337 -0.7029225
9.6293504
H -2.2487350 -1.3838948
10.2755041
H -4.3824244 -2.3246019
11.0898902
H -6.0651615 -3.2069789
9.4768532
H -5.6148325 -3.1487675
7.0490756

NiH_2PhenTAA_0eNeutral_Triplet:
49 atoms
C -0.8887030 2.0868149
2.0178121
C 0.0663895 2.5868967
2.9339840
C 0.3676886 1.8907532
4.0915852
C -0.2699110 0.6563381
4.3669745
C -1.2502530 0.1432355
3.4270042
C -1.5420030 0.8909867
2.2599094
N -1.8573815 -1.0106084
3.8372273
N -0.1311479 -0.1073931
5.4920184
C 0.9575347 -0.0945614
6.3359095
C -2.5060316 -1.9067000
3.0164564
C -3.5134657 -2.7800959
3.5656093
C -4.1817715 -3.6676005
2.6844522
C -3.8639026 -3.7534417
1.3392120
C -2.8206217 -2.9608349
0.8279084
C -2.1546317 -2.0667060
1.6545319
C 2.2553768 0.2410023
5.8809522
C 3.3466378 0.2663062
6.7382138
C 3.1867474 -0.0646713
8.0958175
C 1.9418679 -0.4643145
8.5525936
C 0.8062830 -0.5205279
7.7052078
C -3.8523866 -2.8494412
4.9516358
C -0.4020947 -1.0449959
8.2577759
N -1.5254535 -1.2538347
7.5892501
N -3.2663440 -2.1645897
5.9211352
C -3.6605429 -2.2250555
7.2548928
C -2.6972180 -1.7210694
8.1778895
C -2.9986802 -1.6978823
9.5524324
C -4.2198627 -2.1825558
10.0048689
C -5.1706891 -2.6799922
9.0938907
C -4.8997843 -2.6924130
7.7310295
Ni -1.6512950 -1.1989690
5.6995553
H -0.3570012 -1.3563832
9.3078816
H -4.6182915 -3.5846670
5.2246629
H -1.1429217 2.6691166
1.1314559
H 0.5408365 3.5508547
2.7467248
H 1.0546522 2.3117387
4.8240128
H -2.3224432 0.5437635
1.5845938
H -4.9629550 -4.3097122
3.0975080
H -4.3980221 -4.4492704
0.6920327
H -2.5200711 -3.0541940 -
0.2163835
H -1.3204894 -1.4870689
1.2612870
H 2.3954666 0.4578938
4.8227880
H 4.3305118 0.5308547
6.3488478
H 4.0380386 -0.0356361
8.7760895
H 1.8189852 -0.7624746
9.5962420
H -2.2874303 -1.2719202
10.2600315
H -4.4516583 -2.1517002
11.0699131
H -6.1368823 -3.0332833
9.4553428
H -5.6596755 -3.0359914
7.0292338

NiH_2PhenTAA_0eNeutral_Quintet:
49 atoms
C -0.6831546 1.9308760
1.8948969
C 0.2647954 2.4285242
2.8052277
C 0.5345007 1.7439216
3.9848457
C -0.1146391 0.5253154
4.2783130
C -1.0957899 0.0105997
3.3367492
C -1.3628968 0.7481116
2.1630746
N -1.7864769 -1.1016294
3.7678553
N -0.0213363 -0.1760963
5.4612200
C 1.0091470 -0.0512782
6.3713481
C -2.5519688 -1.9161908
2.9575331
C -3.6667697 -2.6438693
3.5312289
C -4.4733933 -3.3970538
2.6395458
C -4.1760159 -3.5313374
1.2907989
C -3.0283550 -2.9150872
0.7670580
C -2.2431824 -2.1172008
1.5921793
C 2.3254967 0.2766878
5.9716686
C 3.3595368 0.4293656
6.8890585
C 3.0964986 0.2720013
8.2592511
C 1.8275957 -0.1038914
8.6765874
C 0.7584170 -0.3301883
7.7716255
C -4.0245328 -2.6909332
4.9177089
C -0.4621852 -0.8303305
8.3306422
N -1.5068188 -1.2797852
7.6416682
N -3.2975284 -2.2173370
5.9253264
C -3.6236161 -2.3407546
7.2661852
C -2.6487963 -1.8303943
8.2005815
C -2.9140823 -1.9304066
9.5832347
C -4.0881385 -2.5056856
10.0450105
C -5.0398150 -3.0043346
9.1327018
C -4.8077703 -2.9221744
7.7681563
Ni -1.6185554 -1.2301651
5.6827817
H -0.4782762 -0.8896484
9.4259195
H -4.9575597 -3.2249750
5.1359695
H -0.9166961 2.4922324
0.9895819
H 0.7666276 3.3761013
2.6062740
H 1.2122827 2.1747776
4.7196891
H -2.1499338 0.4101606
1.4911821
H -5.3438195 -3.9133286
3.0503393
H -4.8169442 -4.1382478
0.6506301
H -2.7459515 -3.0623447 -
0.2755698
H -1.3369271 -1.6613050
1.1952330
H 2.5336557 0.3697939
4.9063915
H 4.3657634 0.6624280
6.5401921
H 3.8902865 0.4144037
8.9933227
H 1.6396235 -0.2662083
9.7402320
H -2.1949310 -1.5393338
10.3029153
H -4.2770138 -2.5653667
11.1173910
H -5.9652113 -3.4500668
9.4990111
H -5.5601454 -3.3023514
7.0772325

NiH_2PhenTAA_1eOX_Doublet:
49 atoms
C -0.8383812 2.0454916
1.9880198
C 0.1169492 2.5453601
2.9039822
C 0.4028916 1.8640967
4.0738062
C -0.2536714 0.6434535
4.3507443
C -1.2285506 0.1333695
3.4162123
C -1.5110106 0.8626861
2.2388274
N -1.8419678 -1.0180937
3.8438542
N -0.1319153 -0.1233324
5.4830783
C 0.9515780 -0.1013391
6.3310594
C -2.4991922 -1.9065531
3.0239917
C -3.5248541 -2.7537408
3.5616941
C -4.2267141 -3.6246826
2.6957485
C -3.9082724 -3.7232941
1.3512892
C -2.8516183 -2.9496824
0.8431489
C -2.1647067 -2.0602240
1.6571214
C 2.2494294 0.2481881
5.8869204
C 3.3255987 0.2809995
6.7623421
C 3.1579966 -0.0267457
8.1226740
C 1.9074080 -0.4150678
8.5742760
C 0.7959593 -0.4927806
7.7026771
C -3.8841896 -2.7731796
4.9441922
C -0.4351718 -0.9677985
8.2498186
N -1.5046995 -1.2844041
7.5638496
N -3.2271504 -2.1863508
5.9129957
C -3.6419963 -2.2559362
7.2648129
C -2.6897438 -1.7570479
8.1775303
C -2.9656579 -1.7597223
9.5509841
C -4.1805904 -2.2660736
10.0047828
C -5.1270785 -2.7620979
9.0975878
C -4.8654253 -2.7551863
7.7301438
Ni -1.6551649 -1.1877194
5.6976107
H -0.4557144 -1.1085651
9.3351380
H -4.7675926 -3.3657623
5.2023951
H -1.0836490 2.6246077
1.0978869
H 0.5972972 3.5042152
2.7096021
H 1.0821871 2.2936129
4.8075520
H -2.2977466 0.5252450
1.5668116
H -5.0230397 -4.2439816
3.1123866
H -4.4512478 -4.4109898
0.7044676
H -2.5522382 -3.0555037 -
0.2000712
H -1.3225835 -1.5013699
1.2531856
H 2.4095520 0.4497764
4.8293985
H 4.3145794 0.5352199
6.3796753
H 4.0043404 0.0124011
8.8070008

H 1.7690009 -0.6896823 9.6215094
H -2.2562760 -1.3484665 10.2676253
H -4.4023127 -2.2560782 11.0715930
H -6.0837927 -3.1372428 9.4599486
H -5.6258518 -3.1140282 7.0381185

NiH$_2$PhenTAA_1eOX_Quartet:
49 atoms
C -0.8509667 2.0955155 1.9816603
C 0.0969948 2.5918473 2.8907060
C 0.3961155 1.8901606 4.0559572
C -0.2303890 0.6580447 4.3194319
C -1.2059044 0.1473201 3.3841264
C -1.5083490 0.8930549 2.2297402
N -1.8665693 -0.9930885 3.8117124
N -0.1179602 -0.0776799 5.4881344
C 0.9581377 -0.0713547 6.3336040
C -2.5146963 -1.8886686 3.0050719
C -3.5220527 -2.7609398 3.5698142
C -4.1951019 -3.6514986 2.7033658
C -3.8828076 -3.7462089 1.3550534
C -2.8402160 -2.9621674 0.8300670
C -2.1694927 -2.0594909 1.6377516
C 2.2653841 0.2611471 5.8880666
C 3.3438140 0.2731483 6.7563625
C 3.1679001 -0.0579964 8.1116605
C 1.9164740 -0.4546016 8.5600862
C 0.7926390 -0.5036140 7.7048582
C -3.8626976 -2.8414310 4.9627505
C -0.4167018 -1.0385823 8.2651925
N -1.5414762 -1.2241878 7.6147664
N -3.3036190 -2.1468285 5.9258240
C -3.6734203 -2.2373689 7.2773684
C -2.7122469 -1.7340456 8.1985581
C -3.0025245 -1.7262641 9.5710386
C -4.2187465 -2.2287429 10.0251863
C -5.1661875 -2.7249260 9.1171898
C -4.9023954 -2.7211594 7.7503112
Ni -1.6843274 -1.1285475 5.6958059
H -0.3484811 -1.3684602 9.3096733
H -4.6129671 -3.5988567 5.2230580
H -1.1015554 2.6702891 1.0902610
H 0.5797933 3.5506581 2.7025994
H 1.0754174 2.3181027 4.7911526
H -2.2953818 0.5533080 1.5586590
H -4.9740882 -4.2919181 3.1210510
H -4.4202442 -4.4466364 0.7167186
H -2.5466483 -3.0713697 -0.2141270
H -1.3327118 -1.4916770 1.2343355
H 2.4202336 0.4725544 4.8313456
H 4.3346310 0.5287252 6.3803044
H 4.0134320 -0.0352708 8.7982988
H 1.7888838 -0.7546685 9.6017797
H -2.2930513 -1.3019853 10.2810244
H -4.4428748 -2.2150287 11.0915826
H -6.1246103 -3.0957319 9.4798806
H -5.6633870 -3.0668794 7.0511800

NiH$_2$PhenTAA_1eOX_Sextet:
49 atoms
C -0.6743512 1.9281679 1.8877265
C 0.2730314 2.4254899 2.7974710
C 0.5395334 1.7459355 3.9825666
C -0.1134290 0.5310068 4.2730389
C -1.0938126 0.0167311 3.3322264
C -1.3615477 0.7482353 2.1573503
N -1.7802776 -1.0972638 3.7651813
N -0.0206084 -0.1745471 5.4534755
C 1.0052062 -0.0538184 6.3670100
C -2.5473141 -1.9143334 2.9614235
C -3.6798836 -2.6251078 3.5353873
C -4.5063655 -3.3638389 2.6428578
C -4.2011698 -3.5120508 1.2995632
C -3.0340846 -2.9237669 0.7837939
C -2.2331192 -2.1328623 1.6041090
C 2.3232988 0.2553744 5.9717817
C 3.3483109 0.4191897 6.9005561
C 3.0773978 0.2953181 8.2739669
C 1.8063269 -0.0651949 8.6908909
C 0.7450422 -0.3123667 7.7751982
C -4.0351513 -2.6770802 4.9099713
C -0.4622069 -0.8128964 8.3323913
N -1.5048062 -1.3063541 7.6293752
N -3.2740108 -2.2321577 5.9337778
C -3.6132447 -2.3644558 7.2519513
C -2.6239987 -1.8470043 8.2001763
C -2.8902714 -1.9442639 9.5878463
C -4.0620387 -2.5194141 10.0342292
C -5.0218709 -3.0220524 9.1140605
C -4.8034396 -2.9454606 7.7541275
Ni -1.5753939 -1.2955169 5.6732788
H -0.4976952 -0.8491532 9.4256332
H -4.9800365 -3.1814761 5.1344340
H -0.9029577 2.4878444 0.9808906
H 0.7774319 3.3701281 2.5947023
H 1.2161490 2.1810513 4.7158258
H -2.1493683 0.4146707 1.4843419
H -5.3909865 -3.8560562 3.0501598
H -4.8484790 -4.1064509 0.6555341
H -2.7519539 -3.0805002 -0.2571162
H -1.3195910 -1.6955991 1.2036588
H 2.5440120 0.3325125 4.9079488
H 4.3573910 0.6450789 6.5564566
H 3.8679975 0.4523234 9.0073687
H 1.6089236 -0.2016880 9.7555464
H -2.1746540 -1.5604982 10.3132909
H -4.2579030 -2.5880500 11.1041547
H -5.9414540 -3.4700343 9.4900688
H -5.5575772 -3.3317447 7.0703405

NiH$_2$PhenTAA_2eOX_CSS:
49 atoms
C -0.9010402 2.0495930 2.0365948
C 0.0668825 2.5560471 2.9647170
C 0.3700952 1.8845650 4.1227110
C -0.2842212 0.6512917 4.4006082
C -1.2788197 0.1310876 3.4473257
C -1.5665960 0.8713783 2.2659296
N -1.8251408 -1.0364683 3.8351206
N -0.1137714 -0.1418124 5.4749932
C 0.9785841 -0.1174894 6.3256281
C -2.4845270 -1.9290084 3.0069052
C -3.4991583 -2.7793089 3.5532156
C -4.1912540 -3.6487663 2.6882208
C -3.8610373 -3.7441007 1.3381087
C -2.8145842 -2.9662589 0.8258075
C -2.1380355 -2.0684295 1.6453120
C 2.2659397 0.2356193 5.8657007
C 3.3517139 0.2600829 6.7351413
C 3.1834547 -0.0584084 8.0889982
C 1.9286509 -0.4471401 8.5529664
C 0.8179767 -0.5210077 7.6903104
C -3.8707305 -2.7930682 4.9418839
C -0.4221923 -0.9891370 8.2464785
N -1.4944473 -1.2976273 7.5694352
N -3.2252987 -2.2033718 5.9108554
C -3.6475650 -2.2404745 7.2598974
C -2.6931920 -1.7409761 8.1744056
C -2.9765927 -1.7190352 9.5469468
C -4.2007926 -2.2035637 9.9947311
C -5.1500855 -2.7004596 9.0851152
C -4.8827894 -2.7166969 7.7204597
Ni -1.6299968 -1.2235164 5.6907527
H -0.4385393 -1.1232026 9.3325840
H -4.7579764 -3.3825133 5.1934082
H -1.1538185 2.6495172 1.1620379
H 0.5229954 3.5269924 2.7699936
H 1.0443485 2.3183399 4.8590414
H -2.3581248 0.5383617 1.5968467
H -4.9898344 -4.2725490 3.0925442
H -4.3971310 -4.4393865 0.6929897
H -2.5142136 -3.0710078 -0.2168476
H -1.2943183 -1.5059147 1.2481170
H 2.4186362 0.4365592 4.8063028
H 4.3396344 0.5152912 6.3514514
H 4.0324377 -0.0288413 8.7711696
H 1.8029882 -0.7189785 9.6020914
H -2.2699737 -1.3062670 10.2653484
H -4.4331631 -2.1754044 11.0590093
H -6.1143010 -3.0553371 9.4481609
H -5.6460755 -3.0731984 7.0304745

NiH$_2$PhenTAA_2eOX_Triplet:
49 atoms
C -0.8736635 2.0834194 2.0130709
C 0.0730899 2.5791644 2.9208024
C 0.3725929 1.8796645 4.0932006
C -0.2611628 0.6531434 4.3428609
C -1.2267031 0.1475331 3.4171573
C -1.5381705 0.8791349 2.2612148
N -1.8587379 -1.0126941 3.8604361
N -0.1504408 -0.1181647 5.4982585
C 0.9444724 -0.1232112 6.3180617
C -2.4774003 -1.9139817 3.0383342
C -3.4969451 -2.7797888 3.5731817
C -4.1755410 -3.6513581 2.7012406

C -3.8360809 -3.7387979 1.3530797
C -2.7857256 -2.9531029 0.8494588
C -2.1202503 -2.0555967 1.6706645
C 2.2391339 0.2257211 5.8487200
C 3.3274207 0.2450290 6.7078290
C 3.1703463 -0.0741960 8.0671365
C 1.9201043 -0.4631414 8.5426045
C 0.8009636 -0.5313166 7.6921404
C -3.8727690 -2.7928472 4.9543378
C -0.4333943 -0.9933377 8.2505872
N -1.5176861 -1.2722616 7.5747118
N -3.2434099 -2.1758607 5.9205351
C -3.6614929 -2.2365380 7.2752044
C -2.7097912 -1.7380034 8.1874740
C -2.9854671 -1.7278668 9.5592392
C -4.2070554 -2.2262036 10.0103980
C -5.1534351 -2.7220958 9.1032477
C -4.8896053 -2.7254777 7.7341010
Ni -1.6892557 -1.1470477 5.7112382
H -0.4421579 -1.1578923 9.3323931
H -4.7411315 -3.4066867 5.2124668
H -1.1278096 2.6613513 1.1247154
H 0.5513683 3.5406281 2.7346617
H 1.0456723 2.3134447 4.8305520
H -2.3305370 0.5456135 1.5934538
H -4.9725062 -4.2820894 3.0979535
H -4.3640189 -4.4340526 0.7011804
H -2.4759417 -3.0595608 -0.1903295
H -1.2745447 -1.4916923 1.2808969
H 2.3846701 0.4239018 4.7882352
H 4.3144867 0.4929408 6.3170512
H 4.0261352 -0.0462006 8.7408679
H 1.8046815 -0.7379373 9.5921558
H -2.2788590 -1.3146173 10.2775321
H -4.4326476 -2.2091910 11.0763202
H -6.1131147 -3.0896952 9.4655362
H -5.6526851 -3.0821841 7.0438295

NiH_2PhenTAA_2eOX_Quintet:
49 atoms
C -0.8998635 2.1133252 2.0237335
C 0.0481197 2.6089770 2.9323130
C 0.3660357 1.8951307 4.0887906
C -0.2410761 0.6505305 4.3349286
C -1.2175255 0.1402139 3.3993971
C -1.5428697 0.8971794 2.2594834
N -1.8504902 -1.0199358 3.8105241
N -0.1021002 -0.1065532 5.4852256
C 0.9713205 -0.0882221 6.3330433
C -2.5061169 -1.9066228 3.0011170
C -3.5201487 -2.7878971 3.5649460
C -4.2079520 -3.6707980 2.6891582
C -3.8982154 -3.7530078 1.3423105
C -2.8493876 -2.9670048 0.8249980
C -2.1644586 -2.0720399 1.6374884
C 2.2724316 0.2487892 5.8891095
C 3.3497779 0.2767998 6.7659852
C 3.1806151 -0.0486717 8.1264759
C 1.9367511 -0.4559317 8.5777583
C 0.8100922 -0.5231127 7.7142040
C -3.8451652 -2.8882425 4.9438940
C -0.3761173 -1.0741581 8.2676663
N -1.5048724 -1.2990580 7.5978307
N -3.2507700 -2.2121542 5.9251765
C -3.6532626 -2.2571916 7.2494621
C -2.6764915 -1.7462785 8.1852470
C -2.9887093 -1.6849408 9.5557752
C -4.2226415 -2.1422439 9.9928048
C -5.1797078 -2.6428943 9.0759019
C -4.9081497 -2.6889226 7.7169069
Ni -1.5955990 -1.2690390 5.6794556
H -0.3153250 -1.4032199 9.3112784
H -4.5954748 -3.6413002 5.2103000
H -1.1637741 2.6975826 1.1423441
H 0.5169923 3.5764535 2.7533021
H 1.0440297 2.3261126 4.8236826
H -2.3318398 0.5611831 1.5884984
H -4.9909533 -4.3088207 3.1021441
H -4.4387481 -4.4425116 0.6949639
H -2.5641544 -3.0636286 -0.2230640
H -1.3265243 -1.5079575 1.2307649
H 2.4323903 0.4578358 4.8325009
H 4.3372036 0.5480932 6.3911069
H 4.0281760 -0.0110851 8.8097618
H 1.8114908 -0.7496525 9.6212104
H -2.2833112 -1.2611081 10.2693318
H -4.4678296 -2.1025729 11.0541491
H -6.1480432 -2.9814715 9.4444549
H -5.6727578 -3.0339569 7.0221588

NiH_2PhenTAA_3eOX_Doublet:
49 atoms
C -0.9038606 2.0753756 2.0360608
C 0.0540469 2.5770395 2.9545785
C 0.3616111 1.8879909 4.1197877
C -0.2788079 0.6561239 4.3796709
C -1.2618137 0.1412859 3.4371395
C -1.5662959 0.8783296 2.2712106
N -1.8484345 -1.0277597 3.8454442
N -0.1293034 -0.1274570 5.4937533
C 0.9617161 -0.1321946 6.3120908
C -2.4662804 -1.9262990 3.0263926
C -3.4736630 -2.8105532 3.5746173
C -4.1516508 -3.6742941 2.7004350
C -3.8171507 -3.7463763 1.3434885
C -2.7774223 -2.9476255 0.8283206
C -2.1172242 -2.0496403 1.6488085
C 2.2555376 0.2392292 5.8399397
C 3.3439248 0.2552964 6.6947177
C 3.1840651 -0.0845752 8.0524548
C 1.9339509 -0.4916285 8.5320059
C 0.8164220 -0.5660502 7.6861337
C -3.8586153 -2.8223332 4.9562145
C -0.4196254 -1.0233505 8.2519727
N -1.5127866 -1.2878607 7.5832425
N -3.2431634 -2.1935716 5.9247506
C -3.6683646 -2.2269434 7.2705125
C -2.7107426 -1.7256851 8.1883234
C -2.9934504 -1.6987900 9.5608554
C -4.2243279 -2.1793030 10.0071065
C -5.1745281 -2.6770015 9.0963886
C -4.9058051 -2.7002086 7.7280528
Ni -1.6786657 -1.1668059 5.7109816
H -0.4217288 -1.1898312 9.3336802
H -4.7255595 -3.4405252 5.2093322
H -1.1652140 2.6709879 1.1601122
H 0.5123299 3.5495756 2.7686730
H 1.0280020 2.3297413 4.8594892
H -2.3650309 0.5527939 1.6059792
H -4.9456762 -4.3144880 3.0882136
H -4.3422687 -4.4467503 0.6926719
H -2.4737173 -3.0539505 -0.2138182
H -1.2697714 -1.4854285 1.2610949
H 2.3992205 0.4361969 4.7781746
H 4.3323778 0.5076141 6.3088345
H 4.0416597 -0.0624709 8.7261656
H 1.8312348 -0.7712518 9.5817735
H -2.2893484 -1.2858670 10.2821048
H -4.4588120 -2.1484467 11.0715374
H -6.1403198 -3.0291986 9.4599114
H -5.6716692 -3.0570648 7.0406180

NiH_2PhenTAA_3eOX_Quartet:
49 atoms
C -0.8879754 2.1005439 2.0175440
C 0.0599276 2.5967842 2.9263990
C 0.3638149 1.8944711 4.0993136
C -0.2656338 0.6640080 4.3452686
C -1.2344875 0.1568198 3.4164213
C -1.5513542 0.8918804 2.2631037
N -1.8541916 -1.0091607 3.8537552
N -0.1458954 -0.1149757 5.4914439
C 0.9469095 -0.1208381 6.3175898
C -2.4781543 -1.9130195 3.0349479
C -3.5036785 -2.7859687 3.5743292
C -4.1929586 -3.6650214 2.6973305
C -3.8546875 -3.7572732 1.3523958
C -2.8003328 -2.9705520 0.8531141
C -2.1250175 -2.0650542 1.6750714
C 2.2393601 0.2188088 5.8578275
C 3.3311901 0.2375219 6.7291572
C 3.1780222 -0.0784412 8.0917648
C 1.9284684 -0.4631421 8.5634058
C 0.8023428 -0.5334322 7.7009197
C -3.8671405 -2.8187524 4.9409423
C -0.4087678 -1.0096058 8.2551726
N -1.5089827 -1.2954647 7.5677215
N -3.2257863 -2.1940135 5.9223421
C -3.6576714 -2.2457863 7.2505879
C -2.6834351 -1.7357639 8.1842953
C -2.9680369 -1.7089653 9.5570777
C -4.1938500 -2.1949585 9.9951754
C -5.1539889 -2.6976845 9.0750130
C -4.8955254 -2.7180754 7.7098965

Ni -1.6632907 -1.1790081 5.7014323
H -0.4137277 -1.1993474 9.3325836
H -4.7192881 -3.4513243 5.2065714
H -1.1453224 2.6818969 1.1315624
H 0.5343192 3.5612304 2.7420052
H 1.0352617 2.3339954 4.8355427
H -2.3456641 0.5641070 1.5939428
H -4.9910626 -4.2931657 3.0970776
H -4.3821636 -4.4512682 0.6979113
H -2.4934852 -3.0725281 -0.1892018
H -1.2807507 -1.5065354 1.2731706
H 2.3980037 0.4190629 4.7990831
H 4.3180418 0.4915503 6.3385378
H 4.0354454 -0.0482234 8.7641719
H 1.8090314 -0.7367416 9.6132877
H -2.2687454 -1.2927053 10.2805529
H -4.4336532 -2.1743076 11.0591275
H -6.1149937 -3.0546187 9.4478151
H -5.6654387 -3.0709580 7.0254984

$NiH_2PhenTAA\_3eOX\_Sextet$:
49 atoms
C -0.9207329 2.1476987 2.0223754
C 0.0302744 2.6455549 2.9341551
C 0.3590802 1.9244730 4.0856452
C -0.2229638 0.6651840 4.3193911
C -1.2086116 0.1491237 3.3743972
C -1.5541591 0.9228669 2.2512844
N -1.8411098 -1.0151625 3.7684708
N -0.0675067 -0.0864691 5.4691198
C 0.9935407 -0.0930386 6.3224732
C -2.4914506 -1.9167426 2.9821570
C -3.5028780 -2.8008817 3.5639741
C -4.1840095 -3.6928513 2.6978390
C -3.8809750 -3.7847690 1.3399279
C -2.8467853 -2.9938651 0.8063416
C -2.1614308 -2.0874275 1.6111722
C 2.3025506 0.2489747 5.8896195
C 3.3789948 0.2641262 6.7728858
C 3.1996987 -0.0803884 8.1252862
C 1.9421482 -0.4885587 8.5683379
C 0.8186328 -0.5404309 7.7053819
C -3.8558688 -2.8903381 4.9447106
C -0.3766823 -1.0710579 8.2786403
N -1.5124933 -1.2897649 7.6291580
N -3.2827377 -2.2157233 5.9327205
C -3.6773561 -2.2563351 7.2587711
C -2.6870956 -1.7384288 8.2076873
C -3.0003715 -1.6807935 9.5767641
C -4.2340524 -2.1414543 10.0113371
C -5.1974208 -2.6452346 9.0882349
C -4.9294489 -2.6895271 7.7283475
Ni -1.6306522 -1.1980580 5.6778818
H -0.3066675 -1.3773225 9.3292612
H -4.6227601 -3.6335142 5.1937109
H -1.1852898 2.7339189 1.1415964
H 0.4971879 3.6146849 2.7546120
H 1.0340661 2.3588613 4.8224593
H -2.3461350 0.5891677 1.5814422
H -4.9629387 -4.3360036 3.1116917
H -4.4228500 -4.4857184 0.7046447
H -2.5705837 -3.0945522 -0.2444204
H -1.3284340 -1.5219723 1.1942931
H 2.4699009 0.4667522 4.8351347
H 4.3698704 0.5378074 6.4070936
H 4.0423858 -0.0571154 8.8165826
H 1.8193532 -0.7892623 9.6105399
H -2.2979880 -1.2583938 10.2946574
H -4.4813947 -2.1082569 11.0732659
H -6.1634651 -2.9878376 9.4615206
H -5.6973851 -3.0359445 7.0374456

$NiMe_2PhenTAA$:
$NiMe_2PhenTAA\_2eRED\_CSS$:
55 atoms
C -0.3716581 2.2337696 2.1844887
C 0.6212163 2.5169767 3.1352131
C 0.7692394 1.6934739 4.2556346
C -0.0581223 0.5671802 4.4572090
C -1.0813899 0.2754106 3.4773850
C -1.2104821 1.1287968 2.3599749
N -1.9301333 -0.7583710 3.8302417
N -0.1004889 -0.2367206 5.5822232
C 0.9559611 -0.4917850 6.4139181
C -2.6558958 -1.5211674 2.9560313
C -3.7842348 -2.3295884 3.4289702
C -4.5332999 -3.0212147 2.4220088
C -4.1875034 -3.0415291 1.0779966
C -3.0215355 -2.3707211 0.6586315
C -2.2838116 -1.6473342 1.5907922
C 2.3000113 -0.3406649 5.9792306
C 3.4006650 -0.5403030 6.8069173
C 3.1915287 -0.9392605 8.1419304
C 1.8979848 -1.1895186 8.5784634
C 0.7347440 -1.0425671 7.7547856
C -4.1428501 -2.4997018 4.8008835
C -0.5310353 -1.4721212 8.2576831
N -1.6868682 -1.2896310 7.5599801
N -3.5243938 -1.8123623 5.8014248
C -3.9843212 -1.6887771 7.0871169
C -2.9537791 -1.3954796 8.0733462
C -3.3513698 -1.1220562 9.4047740
C -4.6916551 -1.1426683 9.7831939
C -5.6923681 -1.4276380 8.8255483
C -5.3377375 -1.6876130 7.5039364
Ni -1.7867789 -1.0947288 5.6896083
C -0.5572047 -2.3355192 9.5029989
C -5.0909519 -3.6248184 5.1647460
H -0.5102174 2.8849164 1.3165592
H 1.2672785 3.3918980 3.0186134
H 1.5060070 1.9459596 5.0187570
H -2.0146753 0.9417503 1.6478491
H -5.4423886 -3.5448110 2.7224439
H -4.8160502 -3.5770386 0.3600809
H -2.6871059 -2.4207132 -0.3816143
H -1.3656297 -1.1520571 1.2735300
H 2.4617042 -0.0604785 4.9379125
H 4.4115468 -0.3970858 6.4144018
H 4.0331136 -1.0558967 8.8313317
H 1.7604350 -1.4941680 9.6171652
H -2.5915392 -0.8280523 10.1296623
H -4.9681265 -0.8946222 10.8117023
H -6.7484636 -1.4017029 9.1080154
H -6.1176868 -1.8320584 6.7556169
H -1.4954856 -2.9055425 9.5406839
H 0.2842680 -3.0492127 9.4958223
H -0.4870659 -1.7733244 10.4549427
H -4.9632227 -3.8913367 6.2228238
H -6.1637245 -3.3873529 5.0228543
H -4.8824523 -4.5188179 4.5525591

$NiMe_2PhenTAA\_2eRED\_OSS$:
55 atoms
C -0.3760313 2.2357346 2.1884931
C 0.6167179 2.5190262 3.1392795
C 0.7667326 1.6937508 4.2581619
C -0.0586010 0.5656941 4.4581551
C -1.0818331 0.2738548 3.4782479
C -1.2128252 1.1289299 2.3623574
N -1.9289193 -0.7617146 3.8299860
N -0.0993303 -0.2400997 5.5819082
C 0.9571865 -0.4940117 6.4138423
C -2.6552690 -1.5235662 2.9554449
C -3.7826213 -2.3333500 3.4284482
C -4.5308525 -3.0257867 2.4215986
C -4.1864749 -3.0437661 1.0771662
C -3.0226119 -2.3699149 0.6571908
C -2.2849090 -1.6467574 1.5894610
C 2.3010674 -0.3389450 5.9800471
C 3.4017656 -0.5381006 6.8077402
C 3.1929651 -0.9407676 8.1416443
C 1.8997447 -1.1941552 8.5773629
C 0.7363755 -1.0464847 7.7541299
C -4.1416261 -2.5029543 4.8003687
C -0.5295651 -1.4754189 8.2572450
N -1.6851121 -1.2937874 7.5590385
N -3.5224325 -1.8163021 5.8008291
C -3.9829684 -1.6897176 7.0860762
C -2.9523693 -1.3966302 8.0722595
C -3.3499750 -1.1195504 9.4028727
C -4.6905911 -1.1361599 9.7804930
C -5.6913434 -1.4209128 8.8229398
C -5.3365932 -1.6846991 7.5020574
Ni -1.7847148 -1.0994469 5.6888019
C -0.5568517 -2.3363808 9.5043836
C -5.0928041 -3.6255220 5.1643922
H -0.5161399 2.8881714 1.3217939
H 1.2611862 3.3952856 3.0239637
H 1.5032393 1.9463697 5.0214843
H -2.0171111 0.9419067 1.6503407
H -5.4382152 -3.5519288 2.7223516
H -4.8145246 -3.5798397 0.3594456
H -2.6898748 -2.4170614 -0.3837582
H -1.3680948 -1.1493958 1.2716820

H 2.4626510 -0.0561529
4.9394499
H 4.4124817 -0.3912342
6.4160854
H 4.0345224 -1.0579042
8.8308542
H 1.7626642 -1.5016958
9.6152402
H -2.5899111 -0.8253701
10.1274506
H -4.9670961 -0.8850311
10.8082266
H -6.7475313 -1.3916685
9.1046753
H -6.1164471 -1.8286517
6.7535353
H -1.4968508 -2.9035739
9.5449360
H 0.2823366 -3.0528403
9.4972716
H -0.4829306 -1.7719738
10.4550047
H -4.9691817 -3.8897460
6.2235858
H -6.1646801 -3.3862071
5.0177451
H -4.8838222 -4.5215459
4.5552126

NiMe$_2$PhenTAA_2eRED_Triplet:
55 atoms
C -0.3991479 2.2505117
2.2118377
C 0.5901037 2.5329803
3.1594770
C 0.7530028 1.6943146
4.2712742
C -0.0624762 0.5601851
4.4614115
C -1.0826825 0.2684815
3.4834083
C -1.2249751 1.1293687
2.3762166
N -1.9109763 -0.7906734
3.8306888
N -0.0920913 -0.2714793
5.5731501
C 0.9664637 -0.5116075
6.4135515
C -2.6491267 -1.5431327
2.9512412
C -3.7602872 -2.3714090
3.4262313
C -4.5018846 -3.0786880
2.4253980
C -4.1804922 -3.0693679
1.0720025
C -3.0525440 -2.3516248
0.6426077
C -2.3131552 -1.6210141
1.5751523
C 2.3070281 -0.3023501
5.9993935
C 3.4102566 -0.5077499
6.8304067
C 3.2051650 -0.9638969
8.1423026
C 1.9112375 -1.2516982
8.5641696
C 0.7492208 -1.0866508
7.7432128
C -4.1285341 -2.5269700
4.8007332
C -0.5229885 -1.5023014
8.2510747
N -1.6744128 -1.3641286
7.5326514
N -3.4776726 -1.8756951
5.8072490
C -3.9739520 -1.6895206
7.0869204
C -2.9533163 -1.3994408
8.0634239
C -3.3383594 -1.0885504
9.3841451

C -4.6968019 -1.0865645
9.7591169
C -5.6850139 -1.3675629
8.8136594
C -5.3277018 -1.6541917
7.4809215
Ni -1.7582613 -1.1411752
5.6735623
C -0.5551279 -2.3261910
9.5236099
C -5.1152197 -3.6200427
5.1617893
H -0.5446257 2.9054829
1.3479008
H 1.2310945 3.4124123
3.0488182
H 1.4907442 1.9469388
5.0326786
H -2.0272838 0.9423429
1.6627128
H -5.3826530 -3.6428255
2.7343825
H -4.8006186 -3.6219132
0.3599173
H -2.7416947 -2.3658991 -
0.4060588
H -1.4166632 -1.0969496
1.2447453
H 2.4728874 0.0144549
4.9701118
H 4.4189237 -0.3220432
6.4501087
H 4.0461406 -1.0998913
8.8285690
H 1.7772722 -1.6044050
9.5871776
H -2.5776701 -0.7977862
10.1081547
H -4.9709358 -0.8387729
10.7885846
H -6.7418643 -1.3424515
9.0943068
H -6.1061106 -1.8010733
6.7326808
H -1.5201586 -2.8377763
9.6226160
H 0.2388729 -3.0926387
9.4999110
H -0.3999289 -1.7373091
10.4480538
H -5.0642912 -3.8403564
6.2348228
H -6.1686345 -3.3743209
4.9270583
H -4.8760794 -4.5453839
4.6097571

NiMe$_2$PhenTAA_2eRED_Quintet:
55 atoms
C -0.4060486 2.2014347
2.2591332
C 0.5547697 2.5032846
3.2340998
C 0.7427248 1.6315746
4.3125328
C -0.0032016 0.4414666
4.4436283
C -1.0074678 0.1221987
3.4270324
C -1.1733147 1.0374339
2.3574743
N -1.7686854 -0.9848453
3.6953217
N 0.0160786 -0.4199992
5.5242055
C 1.0496418 -0.5395107
6.4447733
C -2.6487313 -1.6063963
2.8311387
C -3.7932313 -2.3611008
3.3571676
C -4.6255011 -2.9894924
2.3701609
C -4.3661723 -2.9703175
0.9998827

C -3.2290408 -2.3119098
0.5221073
C -2.4047152 -1.6410169
1.4396284
C 2.3834632 -0.2849455
6.0617293
C 3.4807364 -0.4282981
6.9296735
C 3.2413100 -0.8221352
8.2472656
C 1.9399821 -1.1244802
8.6532941
C 0.7939914 -1.0595605
7.7911821
C -4.1466607 -2.5391421
4.7467908
C -0.4751505 -1.5092669
8.3104791
N -1.6212092 -1.4845297
7.5749547
N -3.4839194 -1.9380713
5.7803194
C -3.9436723 -1.7618061
7.0657273
C -2.9103287 -1.4938220
8.0622468
C -3.3191688 -1.1572584
9.3739852
C -4.6762922 -1.1243642
9.7427622
C -5.6628494 -1.3982396
8.7929820
C -5.2978757 -1.6999541
7.4688990
Ni -1.6221087 -1.3680512
5.6030284
C -0.4734922 -2.2226499
9.6514182
C -5.1866440 -3.5960229
5.0759990
H -0.5731373 2.8845044
1.4205017
H 1.1454577 3.4212353
3.1677590
H 1.4476015 1.8927513
5.1024756
H -1.9570827 0.8475636
1.6244543
H -5.5293186 -3.5004175
2.7006470
H -5.0534783 -3.4739380
0.3131608
H -2.9743490 -2.3173521 -
0.5413675
H -1.4931425 -1.1631680
1.0802467
H 2.5612775 0.0008053
5.0251023
H 4.4933366 -0.2260854
6.5707866
H 4.0624337 -0.9036441
8.9662158
H 1.7950288 -1.4221370
9.6910385
H -2.5656404 -0.8579278
10.1016013
H -4.9513515 -0.8506154
10.7656609
H -6.7219707 -1.3467088
9.0626636
H -6.0764677 -1.8215167
6.7160888
H -1.4188050 -2.7599649
9.7934372
H 0.3476906 -2.9595570
9.6885755
H -0.3369452 -1.5555305
10.5251676
H -5.1374312 -3.8518066
6.1415973
H -6.2299227 -3.2922197
4.8608921
H -4.9989997 -4.5124769
4.4912712

NiMe$_2$PhenTAA_1eRED_Doublet:
55 atoms
C -0.4032993 2.2667576
2.2104555
C 0.5873451 2.5491492
3.1589960
C 0.7409757 1.7257055
4.2768628
C -0.0786096 0.5960487
4.4611165
C -1.0934792 0.3069175
3.4895510
C -1.2399270 1.1610713
2.3801988
N -1.9374683 -0.7409775
3.8490986
N -0.1235805 -0.2238833
5.5860275
C 0.9334742 -0.5084583
6.3977073
C -2.6357953 -1.5254122
2.9806656
C -3.7202644 -2.3836534
3.4416756
C -4.4327290 -3.1192522
2.4489113
C -4.1032292 -3.1092220
1.1049173
C -2.9816156 -2.3673143
0.6865758
C -2.2764160 -1.6085036
1.6032149
C 2.2769096 -0.3113698
5.9621565
C 3.3728005 -0.5569796
6.7696411
C 3.1849222 -1.0327735
8.0818382
C 1.9000453 -1.3151916
8.5112455
C 0.7378906 -1.1140619
7.7091927
C -4.1084454 -2.5305778
4.8192287
C -0.5399679 -1.5149498
8.2346489
N -1.6794355 -1.3146869
7.5481095
N -3.5058015 -1.8346281
5.8001724
C -3.9804438 -1.6969377
7.0998037
C -2.9636094 -1.4074252
8.0729073
C -3.3492017 -1.0947073
9.3909249
C -4.6934891 -1.0907879
9.7624081
C -5.6871056 -1.3737937
8.8115970
C -5.3322216 -1.6594395
7.4934005
Ni -1.7978843 -1.0610627
5.6910936
C -0.5780741 -2.3168774
9.5202925
C -5.1123885 -3.6071931
5.1807293
H -0.5487152 2.9252254
1.3516136
H 1.2227392 3.4301930
3.0478352
H 1.4679131 1.9871027
5.0452229
H -2.0516898 0.9838469
1.6754434
H -5.2936206 -3.7116227
2.7562036
H -4.6932697 -3.6853268
0.3900267
H -2.6532147 -2.3971198 -
0.3549791
H -1.3880665 -1.0698039
1.2774898

H 2.4343037 0.0191509
4.9367481
H 4.3791675 -0.3935370
6.3770874
H 4.0351993 -1.1981813
8.7457447
H 1.7755032 -1.6979491
9.5233710
H -2.5910651 -0.7898770
10.1108515
H -4.9699707 -0.8220595
10.7835701
H -6.7420342 -1.3268564
9.0878923
H -6.1105704 -1.7922124
6.7434093
H -1.5403773 -2.8314055
9.6221667
H 0.2152863 -3.0785334
9.5113241
H -0.4297918 -1.7055204
10.4265669
H -5.0619621 -3.8334861
6.2519311
H -6.1555366 -3.3346482
4.9466644
H -4.8881091 -4.5309090
4.6274518

NiMe$_2$PhenTAA_1eRED_Quartet:
55 atoms
C -0.4909745 2.2737554
2.2943787
C 0.5053555 2.5575789
3.2482709
C 0.7068965 1.7102139
4.3333675
C -0.0826068 0.5473196
4.4943403
C -1.1114488 0.2543020
3.5093439
C -1.2896500 1.1415288
2.4219300
N -1.8608140 -0.8504713
3.8138703
N -0.0585925 -0.3372033
5.5392588
C 1.0022227 -0.5473517
6.4061123
C -2.6332239 -1.5826835
2.9260496
C -3.7391075 -2.3891527
3.4154153
C -4.4824734 -3.0982701
2.4264635
C -4.1587355 -3.0907360
1.0738116
C -3.0359722 -2.3807191
0.6323532
C -2.2895323 -1.6522764
1.5616039
C 2.3293750 -0.3371553
5.9828927
C 3.4287400 -0.5394988
6.8205975
C 3.2095910 -0.9914683
8.1272593
C 1.9156858 -1.2752679
8.5513486
C 0.7646730 -1.1062490
7.7267182
C -4.1027047 -2.5384744
4.8033994
C -0.5225421 -1.5188913
8.2303852
N -1.6642941 -1.3250215
7.5257542
N -3.4811951 -1.8423828
5.7867053
C -3.9678932 -1.6413748
7.0714958
C -2.9523972 -1.3521146
8.0434485
C -3.3329305 -1.0217789
9.3570020
C -4.6880296 -1.0049965
9.7248764
C -5.6758467 -1.2865269
8.7794898
C -5.3205117 -1.5881555
7.4548531
Ni -1.6983298 -1.2300249
5.6374369
C -0.5657650 -2.3499274
9.4952435
C -5.0785374 -3.6345401
5.1760072
H -0.6581793 2.9588213
1.4607276
H 1.1092340 3.4622558
3.1528621
H 1.4423068 1.9615424
5.0965758
H -2.0937275 0.9543856
1.7114125
H -5.3599786 -3.6637512
2.7371287
H -4.7771705 -3.6487789
0.3675376
H -2.7385115 -2.3927731 -
0.4176625
H -1.3907295 -1.1298457
1.2349152
H 2.4922711 -0.0247481
4.9516867
H 4.4381990 -0.3489267
6.4520356
H 4.0464985 -1.1344425
8.8141629
H 1.7802654 -1.6289198
9.5725623
H -2.5700975 -0.7381483
10.0809327
H -4.9638423 -0.7464108
10.7492125
H -6.7309820 -1.2500901
9.0580033
H -6.0965011 -1.7431187
6.7063565
H -1.5400962 -2.8413324
9.5963796
H 0.2116221 -3.1299352
9.4659337
H -0.3959056 -1.7618822
10.4143922
H -5.0342117 -3.8357944
6.2523779
H -6.1273173 -3.3929869
4.9284637
H -4.8295777 -4.5651054
4.6415197

NiMe$_2$PhenTAA_1eRED_Sextet:
55 atoms
C -0.4651692 2.1961595
2.2946747
C 0.5264285 2.4789867
3.2440882
C 0.7522914 1.6058070
4.3086562
C 0.0078617 0.4137908
4.4429204
C -1.0240856 0.1195362
3.4549075
C -1.2364638 1.0386666
2.4046641
N -1.7917974 -0.9888341
3.7375289
N 0.0540837 -0.4624591
5.5048501
C 1.0846138 -0.5593026
6.4313921
C -2.6536131 -1.6247260
2.8526768
C -3.7993128 -2.3768928
3.3674023
C -4.6227708 -2.9790013
2.3596831
C -4.3236733 -2.9568622
1.0012269
C -3.1664129 -2.3201275
0.5408438
C -2.3615805 -1.6568093
1.4763867
C 2.4124926 -0.2957134
6.0469127
C 3.4993140 -0.4204604
6.9221764
C 3.2411714 -0.8019320
8.2429848
C 1.9459272 -1.1084155
8.6475802
C 0.8073425 -1.0648530
7.7771131
C -4.1652234 -2.5605031
4.7473426
C -0.4623533 -1.5063936
8.2914510
N -1.6152416 -1.4472623
7.5577134
N -3.4771707 -1.9775145
5.7757588
C -3.9380094 -1.7756745
7.0676258
C -2.9103048 -1.4829597
8.0511371
C -3.3084698 -1.1382180
9.3576093
C -4.6630518 -1.1059629
9.7275940
C -5.6493574 -1.3871005
8.7837910
C -5.2878515 -1.7022582
7.4636256
Ni -1.6478934 -1.3312564
5.6150532
C -0.4743041 -2.2267082
9.6253666
C -5.2392325 -3.5821299
5.0652393
H -0.6561959 2.8940454
1.4770603
H 1.1109575 3.3980697
3.1690967
H 1.4756305 1.8634969
5.0807949
H -2.0507506 0.8577964
1.7046590
H -5.5392609 -3.4803107
2.6647514
H -4.9985071 -3.4527528
0.2992031
H -2.8895016 -2.3346569 -
0.5140299
H -1.4375975 -1.1841370
1.1438080
H 2.5952876 -0.0337276
5.0049347
H 4.5141430 -0.2243825
6.5738695
H 4.0563330 -0.8736650
8.9672012
H 1.7976367 -1.3916060
9.6878140
H -2.5533864 -0.8349124
10.0805792
H -4.9358268 -0.8279384
10.7478286
H -6.7060711 -1.3325813
9.0539255
H -6.0686474 -1.8367365
6.7172948
H -1.4096063 -2.7855307
9.7457389
H 0.3580329 -2.9467919
9.6751663
H -0.3722152 -1.5604047
10.5014540
H -5.1944375 -3.8616938
6.1241561
H -6.2687629 -3.2373221
4.8575054
H -5.0834374 -4.4948742
4.4682119

NiMe$_2$PhenTAA_0eNeutral_CSS:
55 atoms
C -0.4942047 2.3344732
2.2882415
C 0.4940093 2.6161663
3.2345584
C 0.6735715 1.7748516
4.3361513
C -0.1192156 0.6277120
4.4865713
C -1.1264633 0.3405337
3.5219745
C -1.3102034 1.2094146
2.4364646
N -1.9365205 -0.7508610
3.8730793
N -0.1437045 -0.2396219
5.5900791
C 0.9169560 -0.5484927
6.3786446
C -2.6063107 -1.5518785
3.0058375
C -3.6289550 -2.4809455
3.4579275
C -4.2832482 -3.2770107
2.4746338
C -3.9674154 -3.2319479
1.1330344
C -2.9245515 -2.3829277
0.7121725
C -2.2763358 -1.5692409
1.6155828
C 2.2539429 -0.2794555
5.9519926
C 3.3528488 -0.5964195
6.7203745
C 3.1931220 -1.1943643
7.9862964
C 1.9204400 -1.5112980
8.4124259
C 0.7498992 -1.2339949
7.6496456
C -4.0632621 -2.5849304
4.8298189
C -0.5349476 -1.5791970
8.2078011
N -1.6586863 -1.3759092
7.5266094
N -3.4670508 -1.8916863
5.7952075
C -3.9703718 -1.7022969
7.1033186
C -2.9658736 -1.4154882
8.0650804
C -3.3384445 -1.0333375
9.3586897
C -4.6902249 -0.9730596
9.7149885
C -5.6762275 -1.2548127
8.7709808
C -5.3188725 -1.5991415
7.4626811
Ni -1.7963938 -1.0663798
5.6913199
C -0.5857831 -2.2675338
9.5552461
C -5.1572660 -3.5717679
5.1778798
H -0.6617886 3.0120576
1.4502824
H 1.1040198 3.5153548
3.1412015
H 1.3914868 2.0423959
5.1098588
H -2.1286930 1.0390761
1.7388986
H -5.0750230 -3.9546985
2.7860749
H -4.4973301 -3.8626807
0.4198618
H -2.6058831 -2.3809999 -
0.3318404
H -1.4406291 -0.9602227
1.2790638

H 2.4015695 0.1340708
4.9571474
H 4.3524928 -0.4007630
6.3279927
H 4.0573930 -1.4287682
8.6070720
H 1.8164148 -1.9935179
9.3818850
H -2.5811204 -0.7177603
10.0727779
H -4.9664003 -0.6580308
10.7215246
H -6.7305542 -1.1621184
9.0325187
H -6.0941483 -1.7215021
6.7096850
H -1.5677340 -2.7140708
9.7355086
H 0.1542518 -3.0778077
9.5769829
H -0.3521058 -1.5885081
10.3886529
H -5.1996868 -3.7517523
6.2557504
H -6.1507807 -3.2423393
4.8392708
H -4.9480080 -4.5325657
4.6905202

$NiMe_2PhenTAA$_0eNeutral_OSS:
55 atoms
C -0.4934131 2.3321068
2.2871776
C 0.4941807 2.6146040
3.2338864
C 0.6736685 1.7736715
4.3357785
C -0.1184295 0.6260225
4.4860786
C -1.1252176 0.3382131
3.5212202
C -1.3089119 1.2066944
2.4353844
N -1.9352098 -0.7531982
3.8724756
N -0.1427972 -0.2412977
5.5896835
C 0.9176509 -0.5484591
6.3792273
C -2.6060303 -1.5534045
3.0052971
C -3.6299353 -2.4810772
3.4573913
C -4.2860837 -3.2754100
2.4738485
C -3.9709271 -3.2299940
1.1320793
C -2.9263898 -2.3828883
0.7112991
C -2.2761256 -1.5710689
1.6149684
C 2.2546583 -0.2774863
5.9538512
C 3.3532979 -0.5916282
6.7237660
C 3.1933208 -1.1891547
7.9897530
C 1.9208059 -1.5086663
8.4143288
C 0.7505645 -1.2338148
7.6502209
C -4.0641311 -2.5846890
4.8293770
C -0.5341544 -1.5805086
8.2076044
N -1.6578762 -1.3776191
7.5261456
N -3.4667291 -1.8924866
5.7948586
C -3.9698093 -1.7028017
7.1030582
C -2.9649546 -1.4170019
8.0647533
C -3.3370151 -1.0351801
9.3586391
C -4.6886581 -0.9740455
9.7152362
C -5.6750794 -1.2545277
8.7712305
C -5.3182073 -1.5985409
7.4627188
Ni -1.7954345 -1.0683748
5.6908275
C -0.5848638 -2.2699939
9.5545700
C -5.1598950 -3.5698603
5.1771310
H -0.6609648 3.0093369
1.4489252
H 1.1037319 3.5141211
3.1406186
H 1.3910312 2.0419225
5.1097423
H -2.1270554 1.0358168
1.7375348
H -5.0789925 -3.9518204
2.7851612
H -4.5025347 -3.8591566
0.4187194
H -2.6080860 -2.3810479 -
0.3328448
H -1.4392181 -0.9635693
1.2785525
H 2.4026492 0.1356769
4.9589758
H 4.3530258 -0.3940921
6.3325279
H 4.0573368 -1.4212593
8.6117014
H 1.8165882 -1.9906209
9.3837747
H -2.5793296 -0.7204165
10.0727367
H -4.9643683 -0.6592265
10.7219860
H -6.7293137 -1.1610833
9.0329707
H -6.0937897 -1.7198621
6.7098576
H -1.5664558 -2.7177615
9.7339338
H 0.1560669 -3.0794803
9.5758946
H -0.3524036 -1.5914869
10.3888031
H -5.2028745 -3.7505260
6.2549424
H -6.1528098 -3.2387129
4.8382609
H -4.9521022 -4.5308854
4.6893592

$NiMe_2PhenTAA$_0eNeutral_Triplet:
55 atoms
C -0.4790007 2.2846812
2.2754210
C 0.5201626 2.5694470
3.2320932
C 0.7081106 1.7404258
4.3265836
C -0.0935684 0.5867680
4.4927955
C -1.1211565 0.2939771
3.5089280
C -1.2917507 1.1705183
2.4118184
N -1.8883581 -0.7963366
3.8243173
N -0.0840185 -0.2822725
5.5518190
C 0.9731487 -0.5401687
6.3898781
C -2.6195508 -1.5634892
2.9506329
C -3.6970395 -2.4033622
3.4304945
C -4.4154959 -3.1411768
2.4534684
C -4.0922943 -3.1309003
1.1048061
C -2.9832498 -2.3910737
0.6688221
C -2.2656518 -1.6326338
1.5797401
C 2.3063350 -0.3303195
5.9564240
C 3.3966449 -0.5736096
6.7761900
C 3.1912330 -1.0559539
8.0771957
C 1.9019427 -1.3416560
8.5010081
C 0.7542124 -1.1356792
7.6914270
C -4.0878953 -2.5409362
4.8249787
C -0.5425389 -1.5316191
8.2184468
N -1.6695363 -1.2890366
7.5468587
N -3.5118257 -1.8135101
5.7836195
C -3.9761180 -1.6568155
7.0871118
C -2.9629404 -1.3683401
8.0567835
C -3.3446468 -1.0409075
9.3726960
C -4.6867758 -1.0235962
9.7356845
C -5.6809229 -1.3068121
8.7842708
C -5.3301535 -1.6064336
7.4725680
Ni -1.7535870 -1.1149937
5.6609052
C -0.5848779 -2.3357894
9.4991530
C -5.0866774 -3.6168485
5.1906207
H -0.6405448 2.9695594
1.4421639
H 1.1235922 3.4722976
3.1312765
H 1.4340932 2.0014803
5.0952228
H -2.1037133 0.9933092
1.7080889
H -5.2704228 -3.7374989
2.7655875
H -4.6848916 -3.7133399
0.3989611
H -2.6747440 -2.4162040 -
0.3771617
H -1.3789332 -1.0932612
1.2502664
H 2.4671415 0.0022880
4.9320971
H 4.4059488 -0.3989596
6.4010926
H 4.0346451 -1.2290063
8.7460101
H 1.7738791 -1.7308976
9.5089768
H -2.5852209 -0.7408013
10.0925706
H -4.9663126 -0.7471937
10.7527859
H -6.7341885 -1.2508990
9.0609290
H -6.1069567 -1.7439799
6.7224734
H -1.5578051 -2.8258158
9.6133401
H 0.1894155 -3.1150016
9.4795028
H -0.4096042 -1.7244457
10.3983998
H -5.0551476 -3.8209835
6.2663104
H -6.1230254 -3.3498397
4.9300774
H -4.8473637 -4.5483533
4.6594684

$NiMe_2PhenTAA$_0eNeutral_Quintet:
55 atoms
C -0.4659187 2.2609321
2.2727048
C 0.5254267 2.5444283
3.2223329
C 0.7290219 1.6950204
4.3097885
C -0.0446489 0.5267014
4.4538931
C -1.0725299 0.2386039
3.4761647
C -1.2665342 1.1249931
2.4008026
N -1.8619900 -0.8511319
3.7959011
N -0.0273185 -0.3487642
5.5214007
C 1.0156297 -0.5359387
6.4023002
C -2.6394800 -1.5786306
2.9190088
C -3.7492229 -2.3730557
3.4147516
C -4.4986508 -3.0783138
2.4388508
C -4.1762049 -3.0803017
1.0862886
C -3.0438885 -2.3889982
0.6378281
C -2.2909293 -1.6608478
1.5514666
C 2.3488602 -0.3037079
5.9980704
C 3.4300471 -0.4952044
6.8529051
C 3.1843126 -0.9401873
8.1601721
C 1.8913100 -1.2382262
8.5693505
C 0.7520798 -1.0991867
7.7231195
C -4.1182259 -2.5209865
4.8136863
C -0.5255590 -1.5401668
8.2135884
N -1.6801709 -1.3330293
7.5133740
N -3.5388093 -1.7771887
5.7639876
C -3.9951148 -1.6279991
7.0844270
C -2.9674001 -1.3664501
8.0425510
C -3.3282410 -1.0522419
9.3641381
C -4.6777784 -1.0298484
9.7433717
C -5.6771451 -1.2850499
8.8066294
C -5.3372723 -1.5682751
7.4728746
Ni -1.7243286 -1.1689573
5.6364680
C -0.5656602 -2.4042831
9.4539046
C -5.0736247 -3.6314195
5.1907103
H -0.6343465 2.9443897
1.4398471
H 1.1278181 3.4485061
3.1279400
H 1.4542462 1.9542895
5.0790381
H -2.0784981 0.9453354
1.6982147
H -5.3773385 -3.6380472
2.7535576
H -4.7981574 -3.6374759
0.3847765
H -2.7486807 -2.4194159 -
0.4112214
H -1.3845854 -1.1556931
1.2202899

H 2.5270626 -0.0035107 4.9664199
H 4.4454218 -0.3067694 6.5048599
H 4.0105662 -1.0718244 8.8603819
H 1.7472130 -1.5844332 9.5906957
H -2.5564160 -0.7955790 10.0873319
H -4.9404288 -0.7927044 10.7749384
H -6.7273538 -1.2505310 9.0975855
H -6.1212565 -1.7101889 6.7305652
H -1.5256192 -2.9272337 9.5267813
H 0.2323042 -3.1608014 9.4201243
H -0.4298946 -1.8329400 10.3878855
H -5.0256280 -3.8283169 6.2671474
H -6.1218054 -3.3977573 4.9431180
H -4.8126642 -4.5575868 4.6589106

NiMe2PhenTAA_1eOX_Doublet:
55 atoms
C -0.5743766 2.3483598 2.3562249
C 0.4241268 2.6334430 3.3128029
C 0.6293979 1.7954017 4.3978356
C -0.1592146 0.6346552 4.5419436
C -1.1814342 0.3428859 3.5627465
C -1.3750311 1.2232071 2.4776358
N -1.9122180 -0.7838741 3.8676238
N -0.1291823 -0.2751254 5.5753649
C 0.9458526 -0.5797020 6.3720434
C -2.5905630 -1.5877320 2.9862016
C -3.6020153 -2.5097781 3.4465397
C -4.2451798 -3.3102669 2.4715207
C -3.9181072 -3.2647958 1.1252511
C -2.8904180 -2.4126505 0.6995437
C -2.2465054 -1.5932137 1.6105745
C 2.2654001 -0.3066486 5.9304747
C 3.3732092 -0.6279239 6.6957563
C 3.2097670 -1.2354777 7.9478640
C 1.9328340 -1.5518490 8.3847239
C 0.7690668 -1.2650132 7.6306770
C -4.0547080 -2.6007335 4.8317286
C -0.5320350 -1.5975682 8.2036462
N -1.6462270 -1.3848355 7.5271582
N -3.4653206 -1.9032119 5.7858551
C -3.9648311 -1.6836024 7.0930797
C -2.9610484 -1.3974576 8.0538533
C -3.3298107 -0.9925746 9.3422128
C -4.6797978 -0.9105377 9.6883180
C -5.6678201 -1.1923560 8.7427349
C -5.3146495 -1.5585887 7.4426752
Ni -1.7702609 -1.1099958 5.6770047
C -0.5787629 -2.2515674 9.5630386
C -5.1714053 -3.5590600 5.1670637
H -0.7510814 3.0470731 1.5385592
H 1.0082422 3.5493527 3.2240363
H 1.3491402 2.0614603 5.1699651
H -2.1912659 1.0507997 1.7783609
H -5.0330311 -3.9942711 2.7764257
H -4.4394305 -3.9054963 0.4152572
H -2.5767983 -2.4051470 -0.3447752
H -1.4122742 -0.9781906 1.2798348
H 2.4058665 0.1118570 4.9361728
H 4.3715483 -0.4248041 6.3070427
H 4.0737929 -1.4823943 8.5633601
H 1.8368004 -2.0399535 9.3512470
H -2.5727546 -0.6779335 10.0565565
H -4.9577341 -0.5773797 10.6879364
H -6.7205609 -1.0802866 9.0008985
H -6.0902866 -1.6811043 6.6905110
H -1.5638291 -2.6803580 9.7659453
H 0.1511398 -3.0704274 9.5957078
H -0.3238493 -1.5534535 10.3735049
H -5.2416957 -3.7281523 6.2449227
H -6.1476795 -3.2107688 4.7999206
H -4.9709908 -4.5282343 4.6928920

NiMe2PhenTAA_1eOX_Quartet:
55 atoms
C -0.4728153 2.3145216 2.2669759
C 0.5197973 2.5973730 3.2173756
C 0.7151379 1.7561953 4.3114851
C -0.0700708 0.5994358 4.4578232
C -1.0913966 0.3085282 3.4800740
C -1.2818478 1.1872564 2.3995621
N -1.8985883 -0.7705018 3.8135627
N -0.0809418 -0.2528969 5.5534732
C 0.9697165 -0.5315440 6.3804047
C -2.6131194 -1.5518498 2.9508224
C -3.6849234 -2.3993483 3.4424254
C -4.3901158 -3.1508564 2.4749967
C -4.0592038 -3.1498293 1.1252532
C -2.9566189 -2.4064869 0.6790999
C -2.2508657 -1.6286244 1.5780521
C 2.3068970 -0.3305255 5.9409454
C 3.3912410 -0.5983785 6.7556687
C 3.1770289 -1.0886426 8.0522711
C 1.8840436 -1.3638478 8.4810092
C 0.7416163 -1.1387838 7.6795995
C -4.0862067 -2.5376822 4.8438496
C -0.5616037 -1.5341121 8.2174837
N -1.6825058 -1.2511318 7.5698709
N -3.5447002 -1.7813596 5.7875709
C -3.9968943 -1.6489044 7.1103686
C -2.9875335 -1.3614215 8.0764078
C -3.3630821 -1.0363828 9.3905820
C -4.7072816 -1.0196633 9.7529329
C -5.6986455 -1.3022022 8.8041608
C -5.3486052 -1.6021370 7.4904029
Ni -1.7888549 -1.0213920 5.6698467
C -0.5958434 -2.3582782 9.4834334
C -5.0647713 -3.6305657 5.2060582
H -0.6377278 2.9979800 1.4341754
H 1.1232139 3.4997603 3.1202732
H 1.4362406 2.0210853 5.0830564
H -2.0977312 1.0142582 1.6996962
H -5.2373562 -3.7573336 2.7859711
H -4.6416364 -3.7487561 0.4255325
H -2.6478118 -2.4461812 -0.3655381
H -1.3669404 -1.0872599 1.2454346
H 2.4702031 0.0056170 4.9185065
H 4.4034294 -0.4375625 6.3844125
H 4.0192408 -1.2815110 8.7163713
H 1.7599598 -1.7642792 9.4843144
H -2.6037523 -0.7423896 10.1127144
H -4.9863140 -0.7512936 10.7716512
H -6.7511807 -1.2543524 9.0826428
H -6.1257788 -1.7460523 6.7422424
H -1.5741712 -2.8331449 9.6097665
H 0.1641797 -3.1492437 9.4410308
H -0.3948460 -1.7563643 10.3826564
H -5.0488519 -3.8226836 6.2837651
H -6.0986011 -3.3797739 4.9237672
H -4.8002107 -4.5624803 4.6897086

NiMe2PhenTAA_1eOX_Sextet:
55 atoms
C -0.5763221 2.2526457 2.3787984
C 0.3834865 2.5527524 3.3525601
C 0.6150518 1.6781844 4.4178038
C -0.0917500 0.4661002 4.5034603
C -1.0931388 0.1650014 3.5054739
C -1.3236034 1.0748626 2.4593991
N -1.8442091 -0.9613537 3.7838775
N -0.0245023 -0.4550935 5.5338141
C 1.0135181 -0.5645779 6.4438354
C -2.6350000 -1.6547806 2.9012384
C -3.7710002 -2.4283977 3.3804795
C -4.5435458 -3.0970168 2.3966966
C -4.2202733 -3.0788747 1.0477150
C -3.0649854 -2.4092975 0.6125865
C -2.2873988 -1.7207433 1.5298948
C 2.3326697 -0.2659724 6.0494332
C 3.4122835 -0.3766094 6.9243173
C 3.1857768 -0.8121111 8.2354389
C 1.9074291 -1.1831712 8.6349141
C 0.7784133 -1.1249680 7.7698344
C -4.1384757 -2.5880205 4.7697626
C -0.4576615 -1.6734861 8.2349629
N -1.6210288 -1.5821785 7.4733805
N -3.4863982 -1.9014568 5.7309790
C -3.9362517 -1.6194466 7.0067743
C -2.8760590 -1.3937922 7.9723949
C -3.2088984 -0.9727261 9.2802177
C -4.5440137 -0.8160009 9.6327883
C -5.5700890 -1.0384954 8.6936089
C -5.2720549 -1.4253119 7.3854674
Ni -1.6390416 -1.4283187 5.5992991
C -0.4930386 -2.5746692 9.4418129
C -5.1436625 -3.6419726 5.1621407
H -0.7679324 2.9545482 1.5674736
H 0.9387979 3.4890356 3.2990515
H 1.3134560 1.9533260 5.2059453
H -2.1209967 0.8846345 1.7430702
H -5.4341496 -3.6416670 2.7030232
H -4.8523436 -3.6058761 0.3334000
H -2.7746141 -2.4321047 -0.4376524
H -1.3675364 -1.2359722 1.2060565

H 2.5101381 0.0242783 5.0149168
H 4.4165333 -0.1273109 6.5835974
H 4.0120387 -0.8833371 8.9427670
H 1.7682170 -1.5299862 9.6569724
H -2.4178001 -0.7613127 9.9968087
H -4.8000326 -0.5062058 10.6459133
H -6.6100802 -0.8961031 8.9864492
H -6.0682993 -1.5453831 6.6531000
H -1.4501733 -3.1032402 9.5033981
H 0.3100903 -3.3248745 9.3958446
H -0.3601679 -2.0170750 10.3838778
H -5.1222651 -3.8165425 6.2426548
H -6.1707811 -3.3530065 4.8885345
H -4.9263247 -4.5925284 4.6556370

NiMe$_2$PhenTAA_2eOX_CSS:
55 atoms
C -0.6267864 2.3418289 2.3992229
C 0.3846679 2.6306377 3.3681794
C 0.6052219 1.8040951 4.4444886
C -0.1858406 0.6330413 4.5912297
C -1.2282672 0.3355198 3.5927436
C -1.4223469 1.2252632 2.5022219
N -1.8945998 -0.8087784 3.8577288
N -0.1125629 -0.3003859 5.5643891
C 0.9685658 -0.5967128 6.3703780
C -2.5840507 -1.6095253 2.9690166
C -3.5868522 -2.5313690 3.4394567
C -4.2253951 -3.3266277 2.4675709
C -3.8909162 -3.2772196 1.1152930
C -2.8717478 -2.4241610 0.6827547
C -2.2309988 -1.6029800 1.5991133
C 2.2791882 -0.3165671 5.9174564
C 3.3926102 -0.6387226 6.6797915
C 3.2228917 -1.2514785 7.9245001
C 1.9413470 -1.5712281 8.3699355
C 0.7820846 -1.2871612 7.6214538
C -4.0520914 -2.6156842 4.8338022
C -0.5296312 -1.6127946 8.2053388
N -1.6377613 -1.3962148 7.5281904
N -3.4631263 -1.9161883 5.7810050
C -3.9610347 -1.6672475 7.0833372
C -2.9562422 -1.3809800 8.0450136
C -3.3217823 -0.9640392 9.3309562
C -4.6717303 -0.8694587 9.6697770
C -5.6616052 -1.1516105 8.7224621
C -5.3119492 -1.5311932 7.4263975
Ni -1.7482290 -1.1492291 5.6657202
C -0.5723147 -2.2412989 9.5724066
C -5.1836260 -3.5535771 5.1589026
H -0.8062879 3.0580092 1.5970503
H 0.9488884 3.5591699 3.2785384
H 1.3198484 2.0757843 5.2197623
H -2.2432277 1.0585707 1.8066044
H -5.0142983 -4.0126188 2.7643960
H -4.4085434 -3.9238654 0.4074732
H -2.5611301 -2.4160259 -0.3619833
H -1.3923517 -0.9900520 1.2736757
H 2.4151727 0.0972606 4.9199002
H 4.3911629 -0.4339124 6.2937920
H 4.0865218 -1.5054116 8.5382851
H 1.8536355 -2.0586953 9.3373241
H -2.5662661 -0.6515616 10.0476799
H -4.9514750 -0.5257970 10.6652968
H -6.7133832 -1.0280970 8.9792120
H -6.0892814 -1.6556341 6.6764909
H -1.5581738 -2.6602328 9.7906867
H 0.1525495 -3.0648212 9.6142127
H -0.3050835 -1.5300141 10.3676363
H -5.2716617 -3.7174581 6.2361096
H -6.1489324 -3.1923670 4.7749071
H -4.9907717 -4.5281826 4.6917159

NiMe$_2$PhenTAA_2eOX_OSS:
55 atoms
C -0.6262592 2.3418064 2.3986827
C 0.3851963 2.6306079 3.3676284
C 0.6054586 1.8042887 4.4441724
C -0.1859124 0.6334763 4.5911475
C -1.2283205 0.3359671 3.5926863
C -1.4221161 1.2254680 2.5019239
N -1.8948521 -0.8082142 3.8578417
N -0.1128348 -0.2998238 5.5644781
C 0.9683132 -0.5964036 6.3703257
C -2.5840876 -1.6091610 2.9691708
C -3.5869083 -2.5309973 3.4396022
C -4.2252659 -3.3264354 2.4677413
C -3.8905022 -3.2773091 1.1155297
C -2.8712353 -2.4243388 0.6830340
C -2.2307491 -1.6029013 1.5993338
C 2.2789533 -0.3166156 5.9172170
C 3.3923938 -0.6390886 6.6793796
C 3.2226906 -1.2517218 7.9241556
C 1.9411286 -1.5711271 8.3697740
C 0.7818378 -1.2868472 7.6214126
C -4.0522037 -2.6152949 4.8339252
C -0.5298604 -1.6124522 8.2053455
N -1.6380265 -1.3956852 7.5283080
N -3.4633962 -1.9156477 5.7811193
C -3.9612743 -1.6670006 7.0835101
C -2.9564790 -1.3807425 8.0451895
C -3.3220222 -0.9642736 9.3312782
C -4.6719627 -0.8702333 9.6702532
C -5.6618401 -1.1523727 8.7229341
C -5.3121927 -1.5314049 7.4267131
Ni -1.7485846 -1.1484927 5.6658688
C -0.5724732 -2.2412811 9.5722648
C -5.1835105 -3.5534563 5.1590385
H -0.8055197 3.0578127 1.5963056
H 0.9496681 3.5589628 3.2777838
H 1.3201189 2.0759745 5.2194169
H -2.2429738 1.0587758 1.8062799
H -5.0142166 -4.0123820 2.7645406
H -4.4079639 -3.9241190 0.4077395
H -2.5603557 -2.4164621 -0.3616232
H -1.3920667 -0.9900014 1.2739379
H 2.4149081 0.0971641 4.9196364
H 4.3909436 -0.4346058 6.2932086
H 4.0863397 -1.5058514 8.5378356
H 1.8534300 -2.0585344 9.3371945
H -2.5665306 -0.6517992 10.0480301
H -4.9517188 -0.5270077 10.6659143
H -6.7136279 -1.0292843 8.9798255
H -6.0895509 -1.6558316 6.6768308
H -1.5583159 -2.6602857 9.7904853
H 0.1524090 -3.0647947 9.6138489
H -0.3052381 -1.5301800 10.3676579
H -5.2714699 -3.7173853 6.2362442
H -6.1489178 -3.1924557 4.7751021
H -4.9904536 -4.5280014 4.6918191

NiMe$_2$PhenTAA_2eOX_Triplet:
55 atoms
C -0.5905105 2.3813191 2.3712299
C 0.4004884 2.6640952 3.3204292
C 0.6180264 1.8077843 4.4046576
C -0.1531658 0.6442867 4.5219832
C -1.1648872 0.3551885 3.5523767
C -1.3852371 1.2361209 2.4857671
N -1.9214831 -0.7742124 3.8798170
N -0.1434126 -0.2673569 5.5822013
C 0.9339942 -0.5960794 6.3514846
C -2.5663745 -1.5938068 3.0004941
C -3.5852127 -2.5153043 3.4610523
C -4.2131403 -3.3121374 2.4859465
C -3.8594047 -3.2721066 1.1374835
C -2.8229603 -2.4298595 0.7131483
C -2.1911073 -1.6066460 1.6277123
C 2.2528798 -0.3391738 5.8823836
C 3.3650971 -0.6657940 6.6371347
C 3.2000905 -1.2612424 7.8948927
C 1.9195633 -1.5662384 8.3556897
C 0.7568807 -1.2793186 7.6168142
C -4.0545189 -2.5968542 4.8475587
C -0.5480892 -1.5988093 8.2034424
N -1.6608256 -1.3596789 7.5371094
N -3.4820463 -1.8784891 5.7938361
C -3.9823893 -1.6630816 7.1069984
C -2.9801360 -1.3776509 8.0663273
C -3.3429719 -0.9799693 9.3569097
C -4.6950160 -0.9046433 9.7024045
C -5.6830179 -1.1862417 8.7567716
C -5.3322966 -1.5468502 7.4529214
Ni -1.8030823 -1.0498172 5.6933701
C -0.5888966 -2.2576166 9.5568752
C -5.1645364 -3.5590947 5.1783662
H -0.7735332 3.0791432 1.5543999
H 0.9842600 3.5806242 3.2379500
H 1.3365579 2.0744947 5.1779086
H -2.2029909 1.0646450 1.7877689
H -5.0113712 -3.9906710 2.7753212
H -4.3734307 -3.9172002 0.4256146
H -2.4963414 -2.4365623 -0.3267338
H -1.3448934 -1.0007833 1.3101169

H 2.3833800 0.0637853
4.8801095
H 4.3632371 -0.4802871
6.2403952
H 4.0670834 -1.5131847
8.5047669
H 1.8368276 -2.0419760
9.3294731
H -2.5877757 -0.6705961
10.0754747
H -4.9738847 -0.5802290
10.7045497
H -6.7356779 -1.0824900
9.0183696
H -6.1103787 -1.6746065
6.7041823
H -1.5735686 -2.6847065
9.7648523
H 0.1394767 -3.0783053
9.5838456
H -0.3285020 -1.5607581
10.3669853
H -5.2400742 -3.7279460
6.2558349
H -6.1400016 -3.2129745
4.8064130
H -4.9586997 -4.5281374
4.7058120

$NiMe_2$PhenTAA_2eOX_Quintet:
55 atoms
C -0.5318407 2.3093363
2.3309413
C 0.4600502 2.5920302
3.2807872
C 0.6747343 1.7350245
4.3622969
C -0.0859909 0.5597677
4.4864455
C -1.1073391 0.2687731
3.5084858
C -1.3242540 1.1654155
2.4481848
N -1.8857295 -0.8365360
3.8165556
N -0.0651685 -0.3178937
5.5596588
C 0.9844896 -0.5505829
6.4030451
C -2.6292722 -1.5798284
2.9436447
C -3.7137755 -2.4280707
3.4246404
C -4.4461930 -3.1473607
2.4470953
C -4.1301482 -3.1138119
1.0956919
C -3.0161403 -2.3778818
0.6598392
C -2.2804187 -1.6351379
1.5693810
C 2.3169524 -0.3258036
5.9701950
C 3.4015343 -0.5499317
6.8030355
C 3.1952220 -1.0271097
8.1078067
C 1.9099782 -1.3368344
8.5315164
C 0.7669414 -1.1519491
7.7137871
C -4.0852616 -2.6060469
4.8135671
C -0.5129558 -1.5888533
8.2329807
N -1.6464507 -1.3589629
7.5473395
N -3.4922292 -1.8843871
5.7806445
C -3.9625868 -1.6430948
7.0535192
C -2.9346565 -1.3503411
8.0373759
C -3.3117533 -0.9628384
9.3399868
C -4.6539793 -0.8915289
9.6716112
C -5.6573698 -1.1774976
8.7112872
C -5.3210754 -1.5353731
7.4168954
Ni -1.7155195 -1.2237269
5.6534918
C -0.5669103 -2.4144695
9.4916868
C -5.0563490 -3.6927698
5.1945792
H -0.7129098 3.0047593
1.5116255
H 1.0470950 3.5063714
3.1970735
H 1.3919510 2.0053602
5.1356577
H -2.1432839 0.9979848
1.7505643
H -5.2991609 -3.7482949
2.7537638
H -4.7282074 -3.6823698
0.3842258
H -2.7214918 -2.3943660 -
0.3896730
H -1.3868301 -1.1096758
1.2369696
H 2.4844418 -0.0072253
4.9427834
H 4.4117462 -0.3625834
6.4384267
H 4.0401141 -1.1844005
8.7775146
H 1.7885614 -1.7292952
9.5384809
H -2.5521823 -0.6804937
10.0662106
H -4.9454158 -0.5860537
10.6764099
H -6.7071539 -1.0882679
8.9903246
H -6.0987679 -1.6911727
6.6719277
H -1.5478326 -2.8835453
9.6148248
H 0.1905196 -3.2085642
9.4611428
H -0.3660836 -1.8054949
10.3869797
H -5.0387457 -3.8781011
6.2729125
H -6.0885384 -3.4343439
4.9105236
H -4.8023593 -4.6299519
4.6823313

$NiMe_2$PhenTAA_3eOX_Doublet:
55 atoms
C -0.6119624 2.3613907
2.3882544
C 0.3896382 2.6473123
3.3476782
C 0.6143313 1.8013810
4.4276442
C -0.1656955 0.6343250
4.5563855
C -1.1952863 0.3404862
3.5702184
C -1.4055766 1.2248904
2.4929275
N -1.9075189 -0.7996545
3.8629004
N -0.1199253 -0.2899165
5.5744942
C 0.9506540 -0.6028223
6.3522437
C -2.5649333 -1.6050047
2.9864466
C -3.5786582 -2.5379001
3.4589953
C -4.2134907 -3.3205119
2.4831518
C -3.8693751 -3.2636258
1.1264262
C -2.8396159 -2.4133171
0.6896428
C -2.2013461 -1.5993782
1.6035869
C 2.2739969 -0.3235995
5.8882172
C 3.3829956 -0.6404618
6.6464519
C 3.2083283 -1.2483575
7.9011156
C 1.9246095 -1.5730795
8.3582795
C 0.7652128 -1.3008942
7.6169562
C -4.0542619 -2.6198703
4.8475179
C -0.5416341 -1.6195705
8.2097475
N -1.6532548 -1.3794963
7.5391586
N -3.4781872 -1.8996554
5.7922491
C -3.9781844 -1.6504629
7.0924061
C -2.9696902 -1.3633999
8.0577524
C -3.3320559 -0.9593508
9.3486459
C -4.6847913 -0.8738228
9.6869862
C -5.6768506 -1.1566244
8.7375402
C -5.3301879 -1.5287459
7.4363627
Ni -1.7905213 -1.0781893
5.6887754
C -0.5820146 -2.2612465
9.5684162
C -5.1736083 -3.5687407
5.1731804
H -0.7985409 3.0736687
1.5833149
H 0.9576942 3.5750084
3.2656411
H 1.3271324 2.0771361
5.2038908
H -2.2286750 1.0623093
1.7981307
H -5.0108017 -4.0031643
2.7664215
H -4.3863268 -3.9094034
0.4154404
H -2.5239648 -2.4190892 -
0.3541024
H -1.3484133 -1.0013913
1.2865484
H 2.4062305 0.0697318
4.8815665
H 4.3845879 -0.4504926
6.2594020
H 4.0746831 -1.5007624
8.5138716
H 1.8489126 -2.0511843
9.3317988
H -2.5790291 -0.6544230
10.0715268
H -4.9669483 -0.5421849
10.6865178
H -6.7290495 -1.0446398
9.0001942
H -6.1112440 -1.6611796
6.6914128
H -1.5673512 -2.6822931
9.7861985
H 0.1415877 -3.0870996
9.6025911
H -0.3133624 -1.5567420
10.3701435
H -5.2602394 -3.7350377
6.2503815
H -6.1434301 -3.2160769
4.7906149
H -4.9725912 -4.5427768
4.7067393

$NiMe_2$PhenTAA_3eOX_Quartet:
55 atoms
C -0.6069009 2.3702003
2.3907275
C 0.3848784 2.6533401
3.3409333
C 0.6077201 1.7919966
4.4231571
C -0.1524134 0.6188098
4.5325367
C -1.1679341 0.3290099
3.5597786
C -1.3982911 1.2194490
2.5013993
N -1.9103952 -0.8102112
3.8715100
N -0.1253490 -0.3010498
5.5810558
C 0.9474709 -0.5940588
6.3746855
C -2.5891315 -1.6019955
2.9888120
C -3.6208396 -2.5223071
3.4471338
C -4.2791589 -3.2992436
2.4636339
C -3.9477940 -3.2391721
1.1117367
C -2.8995813 -2.4065208
0.6929973
C -2.2323990 -1.6080275
1.6158176
C 2.2646255 -0.3258527
5.9212473
C 3.3778166 -0.6177529
6.7022165
C 3.2155492 -1.1994087
7.9682407
C 1.9374114 -1.5296173
8.4136816
C 0.7721967 -1.2713221
7.6521677
C -4.0586770 -2.6409624
4.8229929
C -0.5120913 -1.6310670
8.2177313
N -1.6406224 -1.4138406
7.5280336
N -3.4579011 -1.9315266
5.7884907
C -3.9637042 -1.6467098
7.0570430
C -2.9369858 -1.3541535
8.0397827
C -3.3021529 -0.9277404
9.3253202
C -4.6513213 -0.8293555
9.6496733
C -5.6539813 -1.1152360
8.6900523
C -5.3169624 -1.5020520
7.3970137
Ni -1.7663218 -1.1446755
5.6832208
C -0.5778585 -2.3253372
9.5488414
C -5.1366939 -3.6232120
5.1853617
H -0.7962365 3.0731208
1.5789420
H 0.9621544 3.5751317
3.2636826
H 1.3215583 2.0672189
5.1983425
H -2.2200549 1.0563746
1.8054649
H -5.0805410 -3.9741851
2.7552589
H -4.4818441 -3.8608401
0.3930806
H -2.5877328 -2.3972521 -
0.3523885
H -1.3746710 -1.0220769
1.2902167

H 2.4083748 0.0576433 4.9126497
H 4.3766617 -0.4121869 6.3146152
H 4.0824638 -1.4225961 8.5901544
H 1.8516043 -2.0017052 9.3897328
H -2.5479390 -0.6271463 10.0491832
H -4.9428107 -0.4899770 10.6444637
H -6.7038495 -0.9921982 8.9590875
H -6.0976612 -1.6390567 6.6521084
H -1.5681160 -2.7462786 9.7422072
H 0.1415932 -3.1555144 9.5666977
H -0.3116015 -1.6482785 10.3755407
H -5.1997945 -3.7811454 6.2652945
H -6.1226691 -3.3017484 4.8147952
H -4.9190955 -4.5957012 4.7228431

NiMe₂PhenTAA_3eOX_Sextet:
55 atoms
C -0.5948328 2.3751595 2.3709831
C 0.4024593 2.6597606 3.3263734
C 0.6396445 1.7886339 4.3923774
C -0.0872708 0.5893412 4.4913885
C -1.1202983 0.2947013 3.5019931
C -1.3680527 1.2158705 2.4692787
N -1.8823881 -0.8245723 3.7814685
N -0.0350390 -0.2977734 5.5506080
C 1.0037753 -0.5741557 6.3808435
C -2.6009211 -1.6012914 2.9298191
C -3.6838757 -2.4609153 3.4237409
C -4.3875576 -3.2031790 2.4506244
C -4.0595913 -3.1830521 1.0924807
C -2.9595468 -2.4292473 0.6469419
C -2.2414900 -1.6650738 1.5519457
C 2.3420885 -0.3587307 5.9399965
C 3.4284928 -0.6091346 6.7619678
C 3.2180996 -1.1101784 8.0587254
C 1.9225798 -1.4063015 8.4906413
C 0.7771887 -1.1904869 7.6940246
C -4.0962324 -2.6153312 4.8164211
C -0.5129928 -1.5952186 8.2462635
N -1.6474662 -1.3484019 7.5795575
N -3.5260502 -1.8831341 5.7814276
C -3.9824141 -1.6334528 7.0593960
C -2.9429627 -1.3374766 8.0542917
C -3.3232215 -0.9367520 9.3508428
C -4.6664159 -0.8596719 9.6796423
C -5.6740733 -1.1468128 8.7152330
C -5.3388637 -1.5109855 7.4217029
Ni -1.7261160 -1.1738124 5.6498547
C -0.5596950 -2.3885133 9.5227108
C -5.0937445 -3.6790161 5.1830304
H -0.7797500 3.0775560 1.5574588
H 0.9816203 3.5802399 3.2449361
H 1.3602474 2.0555289 5.1649316
H -2.1850059 1.0440919 1.7689961
H -5.2330081 -3.8189268 2.7493430
H -4.6437543 -3.7755271 0.3876634
H -2.6660259 -2.4501606 -0.4033740
H -1.3573960 -1.1217724 1.2197629
H 2.5042425 -0.0205805 4.9170076
H 4.4407089 -0.4249815 6.3996528
H 4.0634799 -1.2955953 8.7220476
H 1.8131047 -1.8129454 9.4935664
H -2.5663979 -0.6524919 10.0795218
H -4.9605264 -0.5519524 10.6838379
H -6.7239086 -1.0545654 8.9961838
H -6.1194552 -1.6648118 6.6790459
H -1.5487762 -2.8272301 9.6851986
H 0.1726697 -3.2064946 9.4954998
H -0.3149249 -1.7614309 10.3953937
H -5.1222066 -3.8451919 6.2641178
H -6.1094728 -3.4097640 4.8504927
H -4.8366807 -4.6317900 4.7011192

NiPh₂PhenTAA:
NiPh₂PhenTAA_2eRED_CSS:
69 atoms
C -0.5692398 -3.0088912 0.7445007
C 0.6140190 -3.3407759 1.4234523
C 0.8000777 -2.9298588 2.7462678
C -0.1817137 -2.1744003 3.4217213
C -1.3985181 -1.8326340 2.7232535
C -1.5619572 -2.2671992 1.3908167
N -2.3443613 -1.2066459 3.5135653
N -0.1792951 -1.8155559 4.7567357
C 0.9344657 -1.5613770 5.5154230
C -3.3291387 -0.3630504 3.0670830
C -4.4882510 -0.0559351 3.9009203
C -5.3892590 0.9249247 3.3849563
C -5.2448523 1.5389940 2.1516828
C -4.1210787 1.2366059 1.3571209
C -3.1897403 0.3244528 1.8292207
C 2.1588007 -1.1767776 4.9010471
C 3.2982387 -0.8462831 5.6184297
C 3.2430939 -0.8462540 7.0265274
C 2.0538614 -1.1688476 7.6593279
C 0.8639494 -1.5621898 6.9741509
C -4.6980681 -0.6032150 5.2221036
C -0.3433684 -1.8307769 7.7222056
N -1.5494520 -1.9636861 7.0923144
N -3.7321230 -1.3482445 5.8393958
C -3.9139121 -2.1748892 6.9301612
C -2.6927754 -2.5195633 7.6308210
C -2.7618948 -3.4404562 8.7024263
C -3.9719574 -4.0052834 9.0890501
C -5.1601735 -3.6695626 8.4076007
C -5.1267816 -2.7724545 7.3460714
Ni -1.9333764 -1.4617532 5.3288789
C -0.3267818 -1.7841420 9.1981337
C 0.5774975 -2.5443795 9.9809615
C 0.5560172 -2.5017498 11.3745936
C -0.3744262 -1.7018341 12.0521965
C -1.2905209 -0.9527116 11.2941168
C -1.2686891 -0.9954858 9.9043462
C -5.8883372 -0.2150780 6.0052716
C -5.7517378 0.2717628 7.3288197
C -6.8570410 0.6201418 8.0972719
C -8.1593306 0.4944173 7.5843038
C -8.3188873 0.0000751 6.2825901
C -7.2102161 -0.3491671 5.5123960
H -0.7317304 -3.3497306 -0.2815813
H 1.3829108 -3.9428028 0.9317547
H 1.6960758 -3.2329285 3.2889973
H -2.5018005 -2.0552650 0.8800309
H -6.2272864 1.2298200 4.0100579
H -5.9811383 2.2775135 1.8235509
H -3.9572515 1.7386804 0.3996104
H -2.2821852 0.1424135 1.2538799
H 2.1738831 -1.1066243 3.8134361
H 4.2098600 -0.5523890 5.0908497
H 4.1140294 -0.5590841 7.6217632
H 2.0179384 -1.0908592 8.7450213
H -1.8448969 -3.7309475 9.2118289
H -3.9906813 -4.7306958 9.9062675
H -6.1087519 -4.1320248 8.6918270
H -6.0430169 -2.5452743 6.8041665
H 1.2904491 -3.1930131 9.4688035
H 1.2664918 -3.1117813 11.9402547
H -0.3984583 -1.6745608 13.1437970
H -2.0302096 -0.3249509 11.7981363
H -1.9972546 -0.4230303 9.3287083
H -4.7474996 0.3573376 7.7459037
H -6.7051608 0.9982193 9.1116370
H -9.0271965 0.7593771 8.1920554
H -9.3223479 -0.1283260 5.8662513
H -7.3525374 -0.7583391 4.5107582

NiPh₂PhenTAA_2eRED_OSS:
69 atoms
C -0.5721318 -3.0143168 0.7475512
C 0.6110521 -3.3462023 1.4266100
C 0.7979337 -2.9334422 2.7487656
C -0.1830423 -2.1762170 3.4233548
C -1.3997621 -1.8344858 2.7248005
C -1.5640289 -2.2707778 1.3930578
N -2.3447346 -1.2065297 3.5146044
N -0.1799821 -1.8152569 4.7578034
C 0.9340046 -1.5598331 5.5157035
C -3.3285971 -0.3621719 3.0675786
C -4.4871327 -0.0531064 3.9014392
C -5.3864921 0.9293492 3.3856155
C -5.2414998 1.5425995 2.1519879
C -4.1187478 1.2375137 1.3569026
C -3.1887225 0.3239887 1.8290326
C 2.1582879 -1.1765656 4.9004295
C 3.2974616 -0.8436668 5.6171261
C 3.2421035 -0.8398259 7.0251572
C 2.0530729 -1.1619052 7.6586587
C 0.8637075 -1.5581865 6.9743593
C -4.6979880 -0.6002502 5.2225871
C -0.3430443 -1.8275419 7.7232209
N -1.5495277 -1.9599621 7.0939413
N -3.7321918 -1.3447438 5.8407579
C -3.9139673 -2.1717356 6.9313160
C -2.6927792 -2.5164658 7.6319941

C -2.7618006 -3.4380241
8.7030111
C -3.9718195 -4.0033076
9.0892361
C -5.1600280 -3.6674113
8.4079495
C -5.1267401 -2.7697204
7.3468661
Ni -1.9334955 -1.4593479
5.3300792
C -0.3244567 -1.7839932
9.1991010
C 0.5827785 -2.5443676
9.9783965
C 0.5634993 -2.5058130
11.3720848
C -0.3676770 -1.7100250
12.0534879
C -1.2864891 -0.9607047
11.2989949
C -1.2668017 -0.9992930
9.9090905
C -5.8899667 -0.2134978
6.0037519
C -5.7568677 0.2718123
7.3283608
C -6.8643320 0.6182525
8.0946985
C -8.1653438 0.4921364
7.5784887
C -8.3214315 -0.0005803
6.2755643
C -7.2106536 -0.3478961
5.5075136
H -0.7352387 -3.3565758 -
0.2779600
H 1.3792443 -3.9496420
0.9355560
H 1.6938714 -3.2363942
3.2916739
H -2.5038404 -2.0587087
0.8822571
H -6.2236094 1.2361377
4.0111042
H -5.9763874 2.2826204
1.8240151
H -3.9545674 1.7386257
0.3989415
H -2.2817820 0.1399006
1.2532841
H 2.1734802 -1.1091960
3.8126705
H 4.2090236 -0.5507319
5.0889117
H 4.1126461 -0.5500927
7.6196843
H 2.0168477 -1.0812434
8.7441395
H -1.8447766 -3.7287906
9.2121986
H -3.9904351 -4.7292353
9.9059956
H -6.1085364 -4.1302322
8.6918269
H -6.0429700 -2.5425863
6.8049486
H 1.2964190 -3.1897399
9.4632315
H 1.2762557 -3.1158509
11.9348029
H -0.3901065 -1.6860973
13.1451674
H -2.0267779 -0.3361666
11.8059912
H -1.9975786 -0.4267752
9.3363976
H -4.7536964 0.3576311
7.7480426
H -6.7151627 0.9950637
9.1100383
H -9.0348832 0.7554914
8.1846224
H -9.3238163 -0.1292090
5.8565939
H -7.3502482 -0.7556837
4.5048332

NiPh$_2$PhenTAA_2eRED_Triplet:
69 atoms
C -0.6230002 -3.1153330
0.7913082
C 0.5563784 -3.4462033
1.4681339
C 0.7550451 -3.0090692
2.7848942
C -0.2142820 -2.2310286
3.4467268
C -1.4275057 -1.8901832
2.7501800
C -1.6056769 -2.3466285
1.4299819
N -2.3553011 -1.2224909
3.5375975
N -0.2005955 -1.8286878
4.7750784
C 0.9181939 -1.5389706
5.5144404
C -3.3125220 -0.3501356
3.0847725
C -4.4382214 0.0275411
3.9302808
C -5.2902383 1.0544415
3.4345723
C -5.1548404 1.6360804
2.1807264
C -4.0869415 1.2403612
1.3580847
C -3.1888791 0.2852543
1.8185964
C 2.1514653 -1.2133156
4.8858007
C 3.2844870 -0.8283159
5.5920238
C 3.2200028 -0.7163019
6.9909348
C 2.0230874 -1.0017707
7.6348920
C 0.8521132 -1.4606143
6.9683516
C -4.6816634 -0.5272760
5.2510243
C -0.3437184 -1.7497962
7.7418270
N -1.5608298 -1.8445664
7.1309663
N -3.7113310 -1.2382450
5.8963605
C -3.9126583 -2.1059017
6.9741290
C -2.7016699 -2.4479602
7.6687169
C -2.7544384 -3.3868541
8.7160433
C -3.9689143 -3.9886717
9.0773810
C -5.1455417 -3.6557288
8.4031823
C -5.1199285 -2.7178697
7.3603304
Ni -1.9345247 -1.4214011
5.3506422
C -0.2823195 -1.7609291
9.2121749
C 0.6829929 -2.5072014
9.9340871
C 0.6882135 -2.5486359
11.3255872
C -0.2761248 -1.8421318
12.0677114
C -1.2467081 -1.1060276
11.3719239
C -1.2554306 -1.0669503
9.9810093
C -5.9091143 -0.1732748
5.9817133
C -5.8476250 0.2331814
7.3420986
C -6.9991870 0.5223392
8.0677355
C -8.2686858 0.4148327
7.4806650
C -8.3532327 0.0000549
6.1387284
C -7.2017046 -0.2856637
5.4104498
H -0.7936769 -3.4690753 -
0.2290276
H 1.3189025 -4.0616802
0.9833006
H 1.6525352 -3.3092847
3.3262894
H -2.5438399 -2.1318176
0.9177883
H -6.0843186 1.4168940
4.0862353
H -5.8514813 2.4158640
1.8628437
H -3.9318929 1.7024074
0.3789800
H -2.3184359 0.0387894
1.2119533
H 2.1820683 -1.2225860
3.7969713
H 4.2015079 -0.5784599
5.0508790
H 4.0823266 -0.3742269
7.5687030
H 1.9698590 -0.8489036
8.7121042
H -1.8390488 -3.6702145
9.2304872
H -3.9825197 -4.7216871
9.8879800
H -6.0939840 -4.1240673
8.6783321
H -6.0369939 -2.4831925
6.8247862
H 1.4212826 -3.0820220
9.3721718
H 1.4432634 -3.1487047
11.8414661
H -0.2800727 -1.8809833
13.1591161
H -2.0117547 -0.5545961
11.9250865
H -2.0241908 -0.5001749
9.4552739
H -4.8691492 0.3097873
7.8167833
H -6.9068884 0.8352559
9.1112893
H -9.1709975 0.6300311
8.0570626
H -9.3311312 -0.1140744
5.6624418
H -7.2849936 -0.6322303
4.3788374

NiPh$_2$PhenTAA_2eRED_Quintet:
69 atoms
C -0.7576726 -3.2549094
0.9474749
C 0.4261145 -3.5883164
1.6263955
C 0.6680993 -3.0830935
2.9056105
C -0.2546511 -2.2244191
3.5445873
C -1.4863485 -1.8768602
2.8373660
C -1.6984156 -2.4158973
1.5487210
N -2.3658435 -1.1192544
3.5697283
N -0.1788267 -1.7371066
4.8245657
C 0.9564055 -1.4909685
5.5608612
C -3.3453572 -0.2794516
3.0913647
C -4.4610926 0.1334044
3.9400611
C -5.2916944 1.1617333
3.4177758
C -5.1563085 1.7082121
2.1433685
C -4.1135322 1.2679329
1.3195745
C -3.2324101 0.3012274
1.8003518
C 2.1945972 -1.2281977
4.9164928
C 3.3405449 -0.8287520
5.6012964
C 3.2803779 -0.6601062
6.9897905
C 2.0791944 -0.9061815
7.6515138
C 0.8962342 -1.3714250
7.0161388
C -4.7612800 -0.3892724
5.2808452
C -0.2798864 -1.6485237
7.8542050
N -1.5338258 -1.5713820
7.3222190
N -3.7706307 -0.9447463
6.0367876
C -3.9152272 -1.8562907
7.0743011
C -2.6822292 -2.2032462
7.7810580
C -2.7364731 -3.2229218
8.7608235
C -3.9311026 -3.8778053
9.0715424
C -5.1113220 -3.5447368
8.3961684
C -5.0986755 -2.5564531
7.4083524
Ni -1.9433724 -1.2739157
5.4364117
C -0.0993241 -1.8126076
9.3029067
C 0.9254026 -2.6142975
9.8701019
C 1.0462165 -2.7899536
11.2450439
C 0.1479823 -2.1631631
12.1319442
C -0.8722608 -1.3659084
11.5930121
C -0.9982831 -1.1931371
10.2175364
C -6.0821145 -0.1278756
5.8685182
C -6.1995883 0.2766536
7.2287805
C -7.4409183 0.4926330
7.8201156
C -8.6283273 0.3156067
7.0954575
C -8.5360965 -0.0897524
5.7490662
C -7.2961879 -0.3009447
5.1544084
H -0.9570293 -3.6668430 -
0.0455275
H 1.1537657 -4.2612507
1.1646343
H 1.5652909 -3.3828691
3.4488448
H -2.6379231 -2.1976415
1.0391952
H -6.0740973 1.5575429
4.0645908
H -5.8372713 2.4980545
1.8159139
H -3.9623438 1.6956039
0.3245044
H -2.3756070 0.0120493
1.1921947
H 2.2175236 -1.2857632
3.8284169
H 4.2597606 -0.6182360
5.0474755
H 4.1472485 -0.3031483
7.5518256

H 2.0362997 -0.7159089
8.7235346
H -1.8175550 -3.5238007
9.2580636
H -3.9319077 -4.6587896
9.8365694
H -6.0465478 -4.0620085
8.6263420
H -6.0105967 -2.3411141
6.8567756
H 1.6186603 -3.1219493
9.1966782
H 1.8381960 -3.4356983
11.6356560
H 0.2366848 -2.3049011
13.2113644
H -1.5835824 -0.8692437
12.2591780
H -1.8039504 -0.5782218
9.8156790
H -5.2870301 0.4086729
7.8104049
H -7.4843454 0.8052541
8.8672563
H -9.6006880 0.4757398
7.5662777
H -9.4461182 -0.2575734
5.1657652
H -7.2437272 -0.6324862
4.1157416

NiPh$_2$PhenTAA_1eRED_Doublet:
69 atoms
C -0.5513681 -2.9886124
0.7252119
C 0.6294230 -3.3201753
1.4027429
C 0.8066707 -2.9339068
2.7331900
C -0.1857906 -2.1973097
3.4058996
C -1.3932344 -1.8579364
2.7128751
C -1.5560869 -2.2703295
1.3773674
N -2.3494189 -1.2471074
3.5181999
N -0.1970091 -1.8526538
4.7538849
C 0.9025444 -1.5524786
5.5043562
C -3.3028647 -0.3696821
3.0898537
C -4.4371503 -0.0264855
3.9341096
C -5.3347038 0.9679280
3.4480378
C -5.1953555 1.5831927
2.2176009
C -4.0903041 1.2518490
1.4094232
C -3.1744726 0.3119661
1.8448091
C 2.1353437 -1.1802109
4.8937540
C 3.2496729 -0.8112850
5.6242957
C 3.1871464 -0.7746650
7.0309525
C 1.9973565 -1.0958221
7.6579351
C 0.8275612 -1.5085815
6.9566814
C -4.6628648 -0.5982031
5.2406480
C -0.3709531 -1.8076065
7.7044479
N -1.5655915 -1.9661911
7.1004670
N -3.7328550 -1.3553369
5.8563663
C -3.9330688 -2.1449027
6.9940465
C -2.7244604 -2.4857672
7.6876626
C -2.7889668 -3.3781555
8.7755291
C -4.0065719 -3.9130282
9.1877140
C -5.1885549 -3.5795426
8.5094610
C -5.1495176 -2.7122277
7.4209989
Ni -1.9497125 -1.5360695
5.3211158
C -0.3149876 -1.8069308
9.1909528
C 0.5287313 -2.6759652
9.9084971
C 0.5342479 -2.6811318
11.3036593
C -0.3011323 -1.8139949
12.0162249
C -1.1404402 -0.9403182
11.3143349
C -1.1476045 -0.9385122
9.9210511
C -5.8971671 -0.2335405
5.9867240
C -5.7950164 0.3714165
7.2532047
C -6.9337616 0.6921151
7.9892077
C -8.2074926 0.4135648
7.4789731
C -8.3245881 -0.1849506
6.2197733
C -7.1836481 -0.5025296
5.4822296
H -0.7055563 -3.3162864 -
0.3047857
H 1.4029943 -3.9083226
0.9050585
H 1.6990563 -3.2450596
3.2759582
H -2.4975929 -2.0664044
0.8678324
H -6.1602665 1.2738347
4.0886093
H -5.9145343 2.3395838
1.8993247
H -3.9298975 1.7581671
0.4548225
H -2.2882457 0.1126807
1.2447413
H 2.1727491 -1.1397391
3.8064376
H 4.1623200 -0.5153036
5.1019638
H 4.0502801 -0.4633109
7.6214785
H 1.9440743 -1.0083427
8.7418974
H -1.8745383 -3.6796350
9.2801091
H -4.0286008 -4.6157767
10.0224800
H -6.1407992 -4.0197329
8.8105876
H -6.0649850 -2.4975965
6.8755789
H 1.1722467 -3.3596421
9.3528255
H 1.1895040 -3.3717784
11.8385442
H -0.3003358 -1.8201823
13.1075448
H -1.7945560 -0.2561699
11.8583140
H -1.8137446 -0.2704051
9.3738006
H -4.8022510 0.5721968
7.6577166
H -6.8284424 1.1621367
8.9689213
H -9.0993403 0.6585283
8.0582771
H -9.3117170 -0.4130704
5.8123197
H -7.2748044 -0.9792552
4.5051714

NiPh$_2$PhenTAA_1eRED_Quartet:
69 atoms
C -0.6467319 -3.1126499
0.8228793
C 0.5410245 -3.4464544
1.5047209
C 0.7570666 -3.0087229
2.8071799
C -0.2141765 -2.2193383
3.4654344
C -1.4380724 -1.8750145
2.7626327
C -1.6244186 -2.3392973
1.4399279
N -2.3196234 -1.1617768
3.5269866
N -0.1827691 -1.7634728
4.7543072
C 0.9483532 -1.5104798
5.5140855
C -3.3130631 -0.3120170
3.0667019
C -4.4419267 0.0166643
3.9150395
C -5.3287705 1.0095791
3.4154097
C -5.1858850 1.6077327
2.1697930
C -4.0954904 1.2711166
1.3565951
C -3.1696792 0.3422349
1.8239621
C 2.1619998 -1.1557242
4.8862302
C 3.3017314 -0.8064784
5.6054014
C 3.2305719 -0.7570394
7.0039584
C 2.0368009 -1.0615006
7.6457377
C 0.8616235 -1.4757411
6.9607781
C -4.6670132 -0.5473241
5.2372050
C -0.3468026 -1.7650776
7.7179638
N -1.5483856 -1.8974001
7.0996644
N -3.7121502 -1.2871096
5.8573683
C -3.9028519 -2.1540859
6.9365509
C -2.6946807 -2.4953109
7.6297106
C -2.7448399 -3.4330985
8.6775413
C -3.9591878 -4.0233683
9.0441479
C -5.1367579 -3.6903873
8.3690085
C -5.1111880 -2.7642506
7.3206238
Ni -1.9055509 -1.3538101
5.3333468
C -0.3116632 -1.7509469
9.1961823
C 0.5883670 -2.5448887
9.9396242
C 0.5543636 -2.5646398
11.3327256
C -0.3823295 -1.7903064
12.0310048
C -1.2862931 -1.0023952
11.3084616
C -1.2550981 -0.9850253
9.9163413
C -5.8799141 -0.1799408
5.9992495
C -5.7625662 0.2909658
7.3257853
C -6.8896542 0.5829354
8.0898080
C -8.1736404 0.4082371
7.5598134
C -8.3093078 -0.0673975
6.2483370
C -7.1816181 -0.3562441
5.4820258
H -0.8146983 -3.4848526 -
0.1895055
H 1.2882431 -4.0758337
1.0176962
H 1.6546642 -3.3064809
3.3483338
H -2.5611646 -2.1214998
0.9278996
H -6.1513129 1.3327810
4.0507491
H -5.9088607 2.3595598
1.8474295
H -3.9477512 1.7492449
0.3867142
H -2.2770787 0.1306774
1.2359638
H 2.1822664 -1.1216727
3.7973023
H 4.2208796 -0.5437700
5.0788467
H 4.0963623 -0.4509175
7.5943577
H 1.9930238 -0.9577629
8.7283258
H -1.8298706 -3.7144927
9.1935528
H -3.9760702 -4.7516040
9.8571709
H -6.0838823 -4.1554776
8.6488437
H -6.0283697 -2.5280417
6.7862217
H 1.3024149 -3.1719306
9.4032259
H 1.2557438 -3.1987219
11.8794378
H -0.4136733 -1.8100065
13.1216345
H -2.0258206 -0.3970967
11.8367604
H -1.9725568 -0.3822641
9.3585640
H -4.7652103 0.4130608
7.7495594
H -6.7662147 0.9480137
9.1115316
H -9.0561919 0.6284903
8.1626699
H -9.3033149 -0.2264285
5.8245762
H -7.2963587 -0.7527245
4.4718867

NiPh$_2$PhenTAA_1eRED_Sextet:
69 atoms
C -0.6985718 -3.1278191
0.9149592
C 0.4826362 -3.4595546
1.5931392
C 0.7274573 -2.9561263
2.8718243
C -0.1959677 -2.0942100
3.4983979
C -1.4226285 -1.7494382
2.7939527
C -1.6431353 -2.2902395
1.5105719
N -2.3142693 -1.0045900
3.5326134
N -0.1269427 -1.6197665
4.7889726
C 1.0162240 -1.4703055
5.5536759
C -3.3583386 -0.2412096
3.0415780
C -4.4952979 0.0805556
3.9010007
C -5.4140473 1.0253912
3.3566869

C -5.3040520 1.5691736
2.0827158
C -4.2234938 1.2259893
1.2606435
C -3.2684686 0.3400437
1.7572224
C 2.2569031 -1.2103854
4.9302631
C 3.4158007 -0.9174414
5.6476566
C 3.3363590 -0.8571114
7.0444790
C 2.1274829 -1.0943534
7.6872921
C 0.9214313 -1.4426267
7.0112663
C -4.7442651 -0.4525652
5.2312034
C -0.2710959 -1.7121348
7.7995651
N -1.5210388 -1.6813581
7.2369580
N -3.7488931 -1.0542253
5.9574192
C -3.9072912 -1.9129820
7.0435564
C -2.6831122 -2.2579938
7.7463017
C -2.7482117 -3.2148699
8.7797602
C -3.9584186 -3.8170570
9.1421911
C -5.1337686 -3.4854785
8.4677785
C -5.1050662 -2.5501603
7.4271623
Ni -1.8940194 -1.1976012
5.3898571
C -0.1437796 -1.8284015
9.2687351
C 0.7576638 -2.7234789
9.8861217
C 0.8158397 -2.8536519
11.2719748
C -0.0252939 -2.0881809
12.0933392
C -0.9238956 -1.1948704
11.4990094
C -0.9844728 -1.0671533
10.1128173
C -6.0404460 -0.1657265
5.8838950
C -6.0690025 0.3693506
7.1920972
C -7.2740511 0.6007402
7.8521774
C -8.4936584 0.3040635
7.2331441
C -8.4854917 -0.2296568
5.9358094
C -7.2807006 -0.4577919
5.2745276
H -0.8962399 -3.5434007 -
0.0748416
H 1.2068552 -4.1340833
1.1326725
H 1.6171267 -3.2638214
3.4201043
H -2.5863484 -2.0833903
1.0062743
H -6.2366696 1.3595315
3.9859304
H -6.0485876 2.2935601
1.7455621
H -4.1008870 1.6696124
0.2715532
H -2.3765739 0.1313252
1.1672232
H 2.2799015 -1.1741838
3.8414999
H 4.3472038 -0.6991642
5.1232185
H 4.2125090 -0.5875321
7.6381459
H 2.0932721 -0.9829936
8.7693099
H -1.8352387 -3.5176122
9.2849963
H -3.9690267 -4.5556239
9.9461233
H -6.0800229 -3.9600179
8.7349227
H -6.0209045 -2.3371814
6.8827976
H 1.4010437 -3.3376083
9.2535101
H 1.5126102 -3.5674880
11.7174128
H 0.0148042 -2.1942476
13.1788255
H -1.5874769 -0.5925214
12.1233294
H -1.6989699 -0.3813801
9.6561572
H -5.1220611 0.5872495
7.6872416
H -7.2619373 1.0148058
8.8627820
H -9.4365993 0.4771411
7.7547045
H -9.4270890 -0.4822895
5.4428882
H -7.2823057 -0.8924776
4.2733435

NiPh$_2$PhenTAA_0eNeutral_CSS:
69 atoms
C -0.5874148 -3.0419849
0.7658456
C 0.5908012 -3.3724254
1.4419117
C 0.7778889 -2.9731485
2.7679409
C -0.2071129 -2.2188706
3.4220257
C -1.4060531 -1.8822680
2.7338278
C -1.5878205 -2.3095206
1.4104082
N -2.3502811 -1.2415689
3.5476578
N -0.2186527 -1.8407630
4.7715825
C 0.8734361 -1.5151926
5.5097663
C -3.2804760 -0.3475801
3.1244325
C -4.3799804 0.0528970
3.9828187
C -5.2653204 1.0656320
3.5170130
C -5.1350315 1.6590875
2.2786715
C -4.0697757 1.2629502
1.4453323
C -3.1792783 0.2911872
1.8520128
C 2.1228187 -1.1977332
4.8969793
C 3.2158581 -0.7840828
5.6296076
C 3.1419290 -0.6688177
7.0322028
C 1.9505498 -0.9653141
7.6609394
C 0.7950550 -1.4035510
6.9544613
C -4.6286239 -0.5408631
5.2732068
C -0.3895507 -1.7351290
7.7071499
N -1.5738292 -1.9005930
7.1236399
N -3.7225388 -1.2954176
5.8897895
C -3.9413600 -2.0834838
7.0529896
C -2.7450678 -2.4206548
7.7397526
C -2.7989823 -3.3018572
8.8274476
C -4.0220769 -3.8294981
9.2463076
C -5.1969762 -3.4982354
8.5718999
C -5.1576088 -2.6369295
7.4735427
Ni -1.9597025 -1.5404758
5.3359014
C -0.2791626 -1.8213707
9.1929374
C 0.5422441 -2.7769079
9.8111130
C 0.5992545 -2.8620617
11.2027536
C -0.1489313 -1.9818449
11.9908827
C -0.9507326 -1.0125947
11.3808391
C -1.0187874 -0.9351747
9.9898787
C -5.9195775 -0.2313090
5.9550639
C -5.9030989 0.4410581
7.1859533
C -7.0954543 0.7182162
7.8543907
C -8.3171908 0.3195632
7.3039444
C -8.3420990 -0.3417645
6.0719742
C -7.1506807 -0.6079397
5.3960710
H -0.7482079 -3.3860455 -
0.2564863
H 1.3546687 -3.9757642
0.9501163
H 1.6655549 -3.2925057
3.3123916
H -2.5320754 -2.1149366
0.9036965
H -6.0642813 1.3973780
4.1777016
H -5.8271486 2.4403865
1.9658530
H -3.9202054 1.7501965
0.4799504
H -2.3277203 0.0498370
1.2199346
H 2.1840620 -1.2153929
3.8111672
H 4.1342488 -0.5116798
5.1060647
H 3.9992268 -0.3236455
7.6094608
H 1.8800209 -0.8400052
8.7399336
H -1.8867526 -3.6081111
9.3302723
H -4.0441468 -4.5263937
10.0845911
H -6.1496778 -3.9326717
8.8760883
H -6.0725121 -2.4281309
6.9275536
H 1.1245819 -3.4586709
9.1901056
H 1.2301096 -3.6172104
11.6736624
H -0.1021525 -2.0473655
13.0786852
H -1.5273465 -0.3160831
11.9909824
H -1.6546208 -0.1918233
9.5075962
H -4.9456261 0.7348782
7.6175970
H -7.0712531 1.2444576
8.8095865
H -9.2488834 0.5291890
7.8310092
H -9.2929061 -0.6508579
5.6356329
H -7.1625626 -1.1216936
4.4339861

NiPh$_2$PhenTAA_0eNeutral_OSS:
69 atoms
C -0.5872558 -3.0418875
0.7656429
C 0.5909504 -3.3723218
1.4417052
C 0.7779486 -2.9731912
2.7678037
C -0.2071106 -2.2190381
3.4219297
C -1.4060384 -1.8824415
2.7337366
C -1.5877426 -2.3095748
1.4102779
N -2.3503386 -1.2418360
3.5476208
N -0.2187070 -1.8410280
4.7715522
C 0.8733284 -1.5153269
5.5097377
C -3.2804639 -0.3477594
3.1244633
C -4.3798749 0.0528410
3.9829149
C -5.2650823 1.0657651
3.5172579
C -5.1348022 1.6592728
2.2789496
C -4.0697127 1.2629366
1.4454896
C -3.1793374 0.2910001
1.8520311
C 2.1227460 -1.1979023
4.8969982
C 3.2156871 -0.7840048
5.6296389
C 3.1416664 -0.6684564
7.0322094
C 1.9502912 -0.9650063
7.6609103
C 0.7949022 -1.4035152
6.9544197
C -4.6285693 -0.5410022
5.2732483
C -0.3896696 -1.7352106
7.7071012
N -1.5739076 -1.9008281
7.1235900
N -3.7225634 -1.2956751
5.8897664
C -3.9414315 -2.0836659
7.0530381
C -2.7451537 -2.4208329
7.7397921
C -2.7990755 -3.3018984
8.8275852
C -4.0222018 -3.8294064
9.2465572
C -5.1970934 -3.4981434
8.5721571
C -5.1577028 -2.6369674
7.4736848
Ni -1.9597839 -1.5408422
5.3358598
C -0.2792555 -1.8214188
9.1928622
C 0.5422902 -2.7767947
9.8110150
C 0.5993554 -2.8619052
11.2026287
C -0.1489200 -1.9818105
11.9907449
C -0.9508645 -1.0127257
11.3807176
C -1.0189662 -0.9353405
9.9897878
C -5.9194885 -0.2313940
5.9550854
C -5.9029868 0.4408906
7.1859773
C -7.0953120 0.7181152
7.8543753

C -8.3170350 0.3196289
7.3038786
C -8.3419638 -0.3416076
6.0719000
C -7.1505727 -0.6078618
5.3960397
H -0.7479871 -3.3858201 -
0.2567447
H 1.3548921 -3.9755331
0.9498621
H 1.6656090 -3.2925708
3.3122495
H -2.5320000 -2.1150187
0.9035610
H -6.0639072 1.3976313
4.1780557
H -5.8267848 2.4407401
1.9662449
H -3.9201634 1.7501689
0.4800991
H -2.3279070 0.0495227
1.2198324
H 2.1840894 -1.2157614
3.8111959
H 4.1340868 -0.5116229
5.1061041
H 3.9988788 -0.3230432
7.6094566
H 1.8796620 -0.8395005
8.7398794
H -1.8868478 -3.6081487
9.3304154
H -4.0442915 -4.5261951
10.0849305
H -6.1498173 -3.9324700
8.8764367
H -6.0726095 -2.4281645
6.9277039
H 1.1247034 -3.4584581
9.1900182
H 1.2303293 -3.6169237
11.6735240
H -0.1020974 -2.0472996
13.0785249
H -1.5275506 -0.3163125
11.9908495
H -1.6549097 -0.1921220
9.5075161
H -4.9455242 0.7345832
7.6176528
H -7.0710991 1.2442820
8.8095777
H -9.2487054 0.5293147
7.8309102
H -9.2927634 -0.6505649
5.6355155
H -7.1624673 -1.1215415
4.4339465

NiPh$_2$PhenTAA_0eNeutral_Triplet:
69 atoms
C -0.6067460 -3.0435748
0.7828801
C 0.5843720 -3.3778484
1.4664265
C 0.7851490 -2.9765038
2.7776898
C -0.2039290 -2.2179103
3.4455262
C -1.4259991 -1.8746273
2.7440273
C -1.5999262 -2.3070275
1.4088792
N -2.3285652 -1.1987248
3.5200270
N -0.1879645 -1.8004919
4.7490348
C 0.9224563 -1.5134401
5.5067132
C -3.2978731 -0.3272432
3.0834044
C -4.4191300 0.0036366
3.9351861
C -5.3194821 0.9915999
3.4575046
C -5.1732962 1.6144935
2.2278081
C -4.0745193 1.2937647
1.4154023
C -3.1548716 0.3520642
1.8476976
C 2.1406156 -1.1351524
4.8888000
C 3.2544817 -0.7649402
5.6246639
C 3.1814106 -0.7340445
7.0260133
C 1.9920638 -1.0651401
7.6562647
C 0.8307305 -1.4733553
6.9495656
C -4.6535224 -0.5799529
5.2476961
C -0.3736982 -1.7853594
7.7047854
N -1.5548185 -1.9686803
7.1062282
N -3.7433534 -1.3522896
5.8495823
C -3.9284038 -2.1453844
6.9865499
C -2.7208728 -2.4856657
7.6798252
C -2.7828301 -3.3806954
8.7680221
C -3.9975053 -3.9154409
9.1729095
C -5.1816327 -3.5816774
8.4930895
C -5.1472254 -2.7142764
7.4106387
Ni -1.9330587 -1.4717115
5.3233975
C -0.3010500 -1.8039138
9.1931016
C 0.5144223 -2.7200966
9.8792105
C 0.5266686 -2.7526493
11.2742351
C -0.2684113 -1.8643276
12.0049014
C -1.0742315 -0.9413631
11.3301308
C -1.0928016 -0.9135658
9.9363514
C -5.9035678 -0.2248422
5.9771856
C -5.8196480 0.4178598
7.2229113
C -6.9735017 0.7207567
7.9443622
C -8.2312567 0.3805890
7.4356389
C -8.3260611 -0.2560267
6.1943540
C -7.1718948 -0.5526153
5.4682434
H -0.7624678 -3.4002497 -
0.2359936
H 1.3381573 -3.9897110
0.9694545
H 1.6782536 -3.2847390
3.3201333
H -2.5402942 -2.1006060
0.8990946
H -6.1520181 1.2858849
4.0934975
H -5.8943780 2.3693191
1.9130979
H -3.9219282 1.8014472
0.4619576
H -2.2657042 0.1537985
1.2509908
H 2.1747023 -1.0921535
3.8011050
H 4.1700309 -0.4704647
5.1097387
H 4.0416425 -0.4237044
7.6197017
H 1.9405911 -0.9917450
8.7407829
H -1.8691373 -3.6791520
9.2747612
H -4.0227418 -4.6188476
10.0058990
H -6.1312855 -4.0244799
8.7954386
H -6.0627552 -2.4972088
6.8671620
H 1.1281900 -3.4172369
9.3067227
H 1.1566029 -3.4770848
11.7927502
H -0.2590818 -1.8900023
13.0953395
H -1.6916534 -0.2401932
11.8935610
H -1.7325623 -0.2061136
9.4070725
H -4.8365194 0.6668239
7.6245698
H -6.8919230 1.2241172
8.9088629
H -9.1333730 0.6120067
8.0034241
H -9.3030787 -0.5266298
5.7910046
H -7.2429915 -1.0562808
4.5030056

NiPh$_2$PhenTAA_0eNeutral_Quintet:
69 atoms
C -0.5795099 -3.0435475
0.7782749
C 0.6080625 -3.3579740
1.4554985
C 0.8147737 -2.9219144
2.7648997
C -0.1649323 -2.1481997
3.4130524
C -1.3845361 -1.8119926
2.7115561
C -1.5732190 -2.2868019
1.3993019
N -2.2982515 -1.1263236
3.4840019
N -0.1539852 -1.7483279
4.7355401
C 0.9614608 -1.5121108
5.5125474
C -3.3239120 -0.3267830
3.0311063
C -4.4535388 -0.0302355
3.9079734
C -5.3813413 0.9262714
3.4031907
C -5.2701761 1.5123640
2.1514972
C -4.1834689 1.2069524
1.3166937
C -3.2230959 0.3148986
1.7744576
C 2.1745985 -1.1074557
4.9058229
C 3.2967236 -0.7751819
5.6513095
C 3.2196377 -0.8023204
7.0502377
C 2.0266231 -1.1493438
7.6719300
C 0.8619216 -1.5266703
6.9590724
C -4.6605374 -0.5817795
5.2259668
C -0.3561162 -1.8262025
7.7058713
N -1.5196538 -2.0308229
7.0790888
N -3.6958490 -1.3355039
5.8561359
C -3.8994221 -2.1865132
6.9420698
C -2.7035116 -2.5534369
7.6392755
C -2.7683820 -3.4742381
8.6912046
C -3.9980440 -4.0293906
9.0699643
C -5.1659343 -3.6770421
8.3922447
C -5.1217627 -2.7690409
7.3311549
Ni -1.9059095 -1.4262582
5.3036482
C -0.3374405 -1.7771116
9.1926782
C 0.4688818 -2.6411699
9.9550672
C 0.4069811 -2.6268300
11.3486235
C -0.4586141 -1.7462350
12.0055123
C -1.2635219 -0.8821088
11.2565220
C -1.2071756 -0.9004459
9.8639885
C -5.8479826 -0.1871998
6.0192871
C -5.6779542 0.3473183
7.3116474
C -6.7774582 0.6658380
8.1061503
C -8.0761210 0.4499776
7.6345522
C -8.2617618 -0.0877146
6.3557890
C -7.1625345 -0.4010177
5.5578535
H -0.7406509 -3.4111525 -
0.2356379
H 1.3674925 -3.9693335
0.9671842
H 1.7112389 -3.2164827
3.3086175
H -2.5161336 -2.0931087
0.8904501
H -6.2059860 1.2328170
4.0422771
H -6.0180579 2.2412251
1.8359108
H -4.0681411 1.6880015
0.3455668
H -2.3285409 0.1375462
1.1792404
H 2.2026341 -1.0194889
3.8204944
H 4.2156159 -0.4732373
5.1479457
H 4.0817224 -0.5244750
7.6574000
H 1.9794746 -1.1108104
8.7581276
H -1.8599605 -3.7705026
9.2099318
H -4.0316107 -4.7414233
9.8950547
H -6.1227815 -4.1125530
8.6823400
H -6.0340941 -2.5187616
6.7964237
H 1.1295857 -3.3427835
9.4431996
H 1.0316901 -3.3115007
11.9241909
H -0.5082983 -1.7364590
13.0949671
H -1.9412688 -0.1919563
11.7608900
H -1.8473345 -0.2398492
9.2778634
H -4.6662299 0.5029912
7.6882364
H -6.6204264 1.0836465
9.1017638
H -8.9364195 0.6919176
8.2596190
H -9.2698365 -0.2751731
5.9823724

H -7.3126937 -0.8464687
4.5733102

NiPh$_2$PhenTAA_1eOX_Doublet:
69 atoms
C -0.6417827 -3.0872851
0.8231054
C 0.5485957 -3.4214736
1.5062212
C 0.7536358 -3.0191325
2.8166686
C -0.2349852 -2.2568023
3.4737764
C -1.4515268 -1.9149346
2.7754275
C -1.6355740 -2.3482247
1.4454857
N -2.3402107 -1.2126984
3.5558054
N -0.2179005 -1.8096966
4.7744173
C 0.8922275 -1.4901027
5.5167618
C -3.2838482 -0.3158993
3.1187900
C -4.3671569 0.0830552
3.9817136
C -5.2458548 1.0922243
3.5220312
C -5.1097368 1.6845983
2.2756181
C -4.0562182 1.2884072
1.4403293
C -3.1633661 0.3130479
1.8547771
C 2.1227420 -1.1719011
4.8903973
C 3.2244393 -0.7573159
5.6215228
C 3.1439635 -0.6363398
7.0155775
C 1.9489490 -0.9323946
7.6537041
C 0.8027499 -1.3719609
6.9503136
C -4.6267998 -0.5201030
5.2832970
C -0.3900622 -1.7137380
7.7158495
N -1.5650944 -1.8783313
7.1333068
N -3.7249828 -1.2699107
5.8930592
C -3.9336886 -2.0827663
7.0413969
C -2.7383835 -2.4197236
7.7275610
C -2.7847063 -3.3202106
8.8013139
C -4.0023354 -3.8686149
9.1965293
C -5.1802025 -3.5364548
8.5204607
C -5.1494959 -2.6534252
7.4439586
Ni -1.9487784 -1.4881407
5.3427339
C -0.2721454 -1.8147308
9.1961867
C 0.5643666 -2.7656548
9.8021705
C 0.6193970 -2.8606623
11.1931272
C -0.1385968 -1.9935460
11.9862060
C -0.9524227 -1.0278207
11.3858534
C -1.0264588 -0.9428464
9.9965740
C -5.9224085 -0.2219357
5.9526849
C -5.9083491 0.4332209
7.1937496
C -7.1048984 0.7059301
7.8545127
C -8.3223395 0.3126623
7.2900781
C -8.3420388 -0.3348837
6.0508415
C -7.1480337 -0.5916147
5.3761235
H -0.8001995 -3.4532770 -
0.1912705
H 1.2960435 -4.0417232
1.0116530
H 1.6423024 -3.3370160
3.3596001
H -2.5782842 -2.1518210
0.9373984
H -6.0464332 1.4251335
4.1793668
H -5.8008455 2.4667970
1.9642484
H -3.9110517 1.7695371
0.4724912
H -2.3081908 0.0694327
1.2278328
H 2.1774307 -1.1894106
3.8038563
H 4.1442165 -0.4927183
5.0987751
H 3.9999433 -0.2892508
7.5928719
H 1.8822463 -0.8071151
8.7324646
H -1.8721682 -3.6207799
9.3069938
H -4.0244536 -4.5828492
10.0193997
H -6.1272815 -3.9898096
8.8125013
H -6.0646070 -2.4387581
6.9005948
H 1.1552718 -3.4381876
9.1792332
H 1.2582136 -3.6111635
11.6593103
H -0.0888474 -2.0651444
13.0729855
H -1.5310304 -0.3393486
12.0022452
H -1.6693884 -0.2005003
9.5221988
H -4.9547694 0.7254851
7.6349718
H -7.0882536 1.2261774
8.8125309
H -9.2569085 0.5180983
7.8126310
H -9.2904528 -0.6380147
5.6067058
H -7.1581858 -1.0946435
4.4083906

NiPh$_2$PhenTAA_1eOX_Quartet:
69 atoms
C -0.5853087 -3.0407428
0.7568788
C 0.5972101 -3.3727685
1.4355330
C 0.8006227 -2.9603306
2.7518990
C -0.1768337 -2.1889664
3.4026016
C -1.3912310 -1.8478240
2.7055445
C -1.5792777 -2.2920489
1.3860133
N -2.3305377 -1.2031996
3.4978395
N -0.1710662 -1.8101656
4.7375250
C 0.9256735 -1.5196253
5.4975524
C -3.2942015 -0.3329436
3.0748119
C -4.4146390 -0.0087726
3.9389029
C -5.3137741 0.9778894
3.4723206
C -5.1646527 1.6158051
2.2477247
C -4.0683982 1.3073434
1.4275504
C -3.1508919 0.3598041
1.8411110
C 2.1475286 -1.1292951
4.8834801
C 3.2479389 -0.7496898
5.6288110
C 3.1628676 -0.7273080
7.0294032
C 1.9728181 -1.0735030
7.6559625
C 0.8211677 -1.4826751
6.9447526
C -4.6618562 -0.5927729
5.2578060
C -0.3833007 -1.7981271
7.7138972
N -1.5541284 -2.0029738
7.1239937
N -3.7717447 -1.3782403
5.8508629
C -3.9461438 -2.1389302
7.0237640
C -2.7409489 -2.4786291
7.7155536
C -2.8020842 -3.3594532
8.8110907
C -4.0201608 -3.8845979
9.2239659
C -5.2011501 -3.5516323
8.5461131
C -5.1656122 -2.6931227
7.4545048
Ni -1.9434756 -1.5494171
5.3034050
C -0.2895651 -1.8127456
9.2017939
C 0.5038443 -2.7620696
9.8669954
C 0.5267046 -2.8044767
11.2614558
C -0.2283952 -1.8910872
12.0030271
C -1.0069577 -0.9338475
11.3452840
C -1.0436460 -0.8980139
9.9516375
C -5.9191950 -0.2264976
5.9704333
C -5.8476459 0.4532012
7.1954142
C -7.0127776 0.7550036
7.8998191
C -8.2585383 0.3696215
7.3951229
C -8.3367748 -0.3064452
6.1739638
C -7.1741061 -0.5971750
5.4594600
H -0.7436965 -3.3940365 -
0.2619053
H 1.3532853 -3.9828067
0.9415635
H 1.6908949 -3.2741207
3.2951313
H -2.5212444 -2.0912996
0.8776417
H -6.1488129 1.2631809
4.1079519
H -5.8852398 2.3743272
1.9430530
H -3.9207959 1.8295631
0.4821049
H -2.2620906 0.1713669
1.2417234
H 2.1877288 -1.0779904
3.7969490
H 4.1649455 -0.4429076
5.1254699
H 4.0164915 -0.4119594
7.6288300
H 1.9193434 -1.0094543
8.7402751
H -1.8898786 -3.6539513
9.3219737
H -4.0476709 -4.5732137
10.0682330
H -6.1531198 -3.9795613
8.8598343
H -6.0824042 -2.4720256
6.9155797
H 1.0884648 -3.4764268
9.2855199
H 1.1356580 -3.5525637
11.7698371
H -0.2065240 -1.9224300
13.0926515
H -1.5877732 -0.2126609
11.9209140
H -1.6617656 -0.1622196
9.4355633
H -4.8733128 0.7387516
7.5940520
H -6.9492777 1.2925539
8.8463096
H -9.1679398 0.5998278
7.9507309
H -9.3058726 -0.6086804
5.7759220
H -7.2317494 -1.1288176
4.5085087

NiPh$_2$PhenTAA_1eOX_Sextet:
69 atoms
C -0.6695423 -3.1486891
0.9009007
C 0.5315203 -3.4371132
1.5603494
C 0.7773363 -2.9349591
2.8414160
C -0.1757052 -2.1141646
3.4663032
C -1.4088660 -1.8039527
2.7813426
C -1.6429874 -2.3502380
1.5084458
N -2.2972345 -1.0598763
3.5353417
N -0.1311949 -1.6575151
4.7700794
C 0.9902655 -1.4576252
5.5366826
C -3.3547331 -0.3104837
3.0461193
C -4.4950801 0.0045836
3.8964370
C -5.4326993 0.9245991
3.3511402
C -5.3214046 1.4616152
2.0738799
C -4.2247058 1.1392786
1.2673170
C -3.2484227 0.2841540
1.7712988
C 2.2136690 -1.0903580
4.9226599
C 3.3437279 -0.7984268
5.6677870
C 3.2746552 -0.8241619
7.0705377
C 2.0797105 -1.1356255
7.7009704
C 0.9012183 -1.4772367
6.9890625
C -4.6892069 -0.4774477
5.2417898
C -0.3119385 -1.7486853
7.7352589
N -1.4916619 -1.8998819
7.0921510
N -3.6576605 -1.1524765
5.9130527
C -3.8510810 -2.1215894
6.8587366
C -2.6421070 -2.5017937
7.5771728

C -2.7068378 -3.4929072
8.5679982
C -3.9244421 -4.1092512
8.8492415
C -5.0938521 -3.7609012
8.1428768
C -5.0650375 -2.7915841
7.1522073
Ni -1.8650704 -1.2063778
5.3564742
C -0.3199895 -1.7304009
9.2167540
C 0.4981368 -2.5977661
9.9659290
C 0.4059249 -2.6260417
11.3569108
C -0.4905063 -1.7818944
12.0200137
C -1.3030716 -0.9136096
11.2835840
C -1.2272085 -0.8943681
9.8928396
C -5.8590757 -0.1098770
6.0510660
C -5.6848978 0.3413539
7.3788831
C -6.7822209 0.6330087
8.1837589
C -8.0815032 0.4611089
7.6950914
C -8.2723764 -0.0058394
6.3890088
C -7.1789063 -0.2829095
5.5735756
H -0.8617152 -3.5683429 -
0.0863722
H 1.2719302 -4.0807924
1.0859455
H 1.6828267 -3.2168397
3.3765703
H -2.5983353 -2.1795105
1.0149306
H -6.2604785 1.2526859
3.9748200
H -6.0771346 2.1640189
1.7219919
H -4.1099037 1.5747373
0.2753200
H -2.3441980 0.1047215
1.1916069
H 2.2399819 -0.9966523
3.8379738
H 4.2701476 -0.5221084
5.1642170
H 4.1485437 -0.5668098
7.6685639
H 2.0375858 -1.0931487
8.7869423
H -1.8073081 -3.7896013
9.1015243
H -3.9671537 -4.8793358
9.6192949
H -6.0328851 -4.2641340
8.3728897
H -5.9667527 -2.5413908
6.5998305
H 1.1813607 -3.2733535
9.4492379
H 1.0331577 -3.3136141
11.9251332
H -0.5563876 -1.8015797
13.1080523
H -1.9981120 -0.2485579
11.7970187
H -1.8662915 -0.2242020
9.3162056
H -4.6742666 0.4756737
7.7657577
H -6.6251513 0.9979658
9.1993047
H -8.9403864 0.6811237
8.3293202
H -9.2815873 -0.1673371
6.0085073
H -7.3374234 -0.6862891
4.5729674

NiPh$_2$PhenTAA_2eOX_CSS:
69 atoms
C -0.7278774 -3.1588140
0.9187909
C 0.4774077 -3.4977236
1.6104877
C 0.7081360 -3.0778702
2.8990796
C -0.2753262 -2.2898005
3.5553209
C -1.5141510 -1.9411513
2.8441776
C -1.7072054 -2.3985468
1.5128227
N -2.3420731 -1.1739683
3.5827601
N -0.2234079 -1.7706538
4.7992242
C 0.9040323 -1.4604782
5.5346082
C -3.2910687 -0.2800656
3.1260680
C -4.3575487 0.1387237
3.9938921
C -5.2163577 1.1519707
3.5277050
C -5.0657545 1.7315811
2.2688480
C -4.0281899 1.3126726
1.4293720
C -3.1501943 0.3255554
1.8557751
C 2.1217696 -1.1566040
4.8826947
C 3.2317173 -0.7280135
5.5977757
C 3.1502792 -0.5787934
6.9862263
C 1.9563651 -0.8662327
7.6459752
C 0.8115757 -1.3163575
6.9616181
C -4.6373697 -0.4636799
5.3049474
C -0.3801095 -1.6632870
7.7489608
N -1.5602306 -1.8008654
7.1756413
N -3.7300823 -1.1891990
5.9301041
C -3.9298233 -2.0272549
7.0596841
C -2.7320048 -2.3653371
7.7468261
C -2.7721393 -3.2856330
8.8058309
C -3.9851330 -3.8547911
9.1782156
C -5.1662430 -3.5211310
8.5010073
C -5.1442055 -2.6157870
7.4456198
Ni -1.9433159 -1.3950068
5.3794410
C -0.2251960 -1.8151335
9.2149929
C 0.6548276 -2.7705218
9.7542722
C 0.7512420 -2.9227947
11.1372418
C -0.0019732 -2.1054384
11.9863167
C -0.8568955 -1.1345130
11.4521020
C -0.9811688 -0.9962048
10.0715700
C -5.9574790 -0.1993535
5.9244705
C -6.0102341 0.4233846
7.1835086
C -7.2423916 0.6674374
7.7857400
C -8.4249314 0.2688691
7.1518554
C -8.3765041 -0.3518460
5.8993388
C -7.1484624 -0.5729119
5.2763188
H -0.8909342 -3.5606568 -
0.0813992
H 1.2005613 -4.1486982
1.1188445
H 1.5938528 -3.4010433
3.4440702
H -2.6523208 -2.2068143
1.0070229
H -6.0144236 1.5037001
4.1783218
H -5.7424451 2.5256587
1.9547242
H -3.8802801 1.7825288
0.4569999
H -2.2931665 0.0661667
1.2366351
H 2.1629473 -1.1861113
3.7951832
H 4.1494992 -0.4736000
5.0675877
H 4.0072112 -0.2156504
7.5527562
H 1.9016375 -0.7241593
8.7233653
H -1.8603509 -3.5827106
9.3149090
H -4.0065964 -4.5855495
9.9863096
H -6.1082578 -3.9917841
8.7814050
H -6.0607501 -2.3967878
6.9064205
H 1.2421257 -3.4052282
9.0894217
H 1.4196891 -3.6766114
11.5532691
H 0.0841434 -2.2186605
13.0671257
H -1.4252511 -0.4823157
12.1153155
H -1.6534681 -0.2475168
9.6509007
H -5.0857585 0.7234618
7.6782861
H -7.2832566 1.1723777
8.7509418
H -9.3868363 0.4510340
7.6314080
H -9.2976130 -0.6591827
5.4041430
H -7.1072629 -1.0552782
4.2988046

NiPh$_2$PhenTAA_2eOX_OSS:
69 atoms
C -0.7240450 -3.1545728
0.9142140
C 0.4812235 -3.4934702
1.6058957
C 0.7107235 -3.0758942
2.8954593
C -0.2738074 -2.2899368
3.5526346
C -1.5126520 -1.9412849
2.8414826
C -1.7045585 -2.3965993
1.5092470
N -2.3416845 -1.1760065
3.5808754
N -0.2228495 -1.7727475
4.7974348
C 0.9040538 -1.4625095
5.5336128
C -3.2910774 -0.2820847
3.1250506
C -4.3579247 0.1354562
3.9931524
C -5.2172554 1.1485792
3.5275925
C -5.0668807 1.7293036
2.2692352
C -4.0287108 1.3119888
1.4297192
C -3.1502077 0.3250482
1.8554557
C 2.1218267 -1.1571216
4.8824259
C 3.2313704 -0.7287448
5.5982415
C 3.1495167 -0.5812011
6.9868433
C 1.9554701 -0.8696673
7.6458787
C 0.8110245 -1.3196049
6.9607829
C -4.6371492 -0.4673312
5.3041023
C -0.3809831 -1.6666606
7.7474921
N -1.5605295 -1.8059939
7.1733943
N -3.7301743 -1.1943724
5.9279667
C -3.9300399 -2.0320808
7.0577432
C -2.7322812 -2.3701433
7.7448662
C -2.7725044 -3.2896968
8.8045004
C -3.9856567 -3.8581380
9.1774716
C -5.1667808 -3.5244877
8.5002265
C -5.1446013 -2.6198611
7.4442231
Ni -1.9434397 -1.3996698
5.3773638
C -0.2281811 -1.8151924
9.2141257
C 0.6510862 -2.7687665
9.7577748
C 0.7450775 -2.9168160
11.1413881
C -0.0098612 -2.0971462
11.9867160
C -0.8641666 -1.1281258
11.4481128
C -0.9859718 -0.9940178
10.0669603
C -5.9555365 -0.2007752
5.9264350
C -6.0035911 0.4222915
7.1855172
C -7.2335498 0.6691595
7.7910660
C -8.4186460 0.2730701
7.1604307
C -8.3749120 -0.3480506
5.9079240
C -7.1490394 -0.5719847
5.2816120
H -0.8861435 -3.5546795 -
0.0868318
H 1.2053696 -4.1427139
1.1134111
H 1.5964156 -3.3993007
3.4403469
H -2.6496758 -2.2051112
1.0033638
H -6.0155400 1.4994293
4.1784050
H -5.7440980 2.5231452
1.9556546
H -3.8807209 1.7829633
0.4578994
H -2.2927500 0.0669901
1.2363588
H 2.1633205 -1.1852496
3.7948925
H 4.1491140 -0.4731651
5.0685438
H 4.0061315 -0.2183623
7.5540453

H 1.9003235 -0.7284385
8.7233521
H -1.8607069 -3.5865996
9.3136902
H -4.0072922 -4.5883139
9.9860941
H -6.1089199 -3.9945850
8.7811543
H -6.0611550 -2.4006957
6.9050817
H 1.2397076 -3.4053461
9.0958892
H 1.4129774 -3.6691932
11.5608832
H 0.0743940 -2.2071004
13.0680086
H -1.4339438 -0.4741235
12.1083303
H -1.6577096 -0.2467683
9.6428302
H -5.0771102 0.7204994
7.6776892
H -7.2706649 1.1743685
8.7562899
H -9.3788601 0.4574479
7.6425289
H -9.2980362 -0.6535035
5.4153156
H -7.1115502 -1.0547336
4.3041263

NiPh$_2$PhenTAA_2eOX_Triplet:
69 atoms
C -0.6606151 -3.1392125
0.8423283
C 0.5204804 -3.4706472
1.5201518
C 0.7380588 -3.0382244
2.8319882
C -0.2339305 -2.2492820
3.4608231
C -1.4384938 -1.9110358
2.7693680
C -1.6490036 -2.3682438
1.4619677
N -2.3446786 -1.2123522
3.5695806
N -0.2271703 -1.8074669
4.7853648
C 0.8782871 -1.4709746
5.5122026
C -3.2668801 -0.3059064
3.1319355
C -4.3544660 0.0951173
3.9960188
C -5.2162907 1.1060415
3.5337009
C -5.0590845 1.7033547
2.2834555
C -4.0040061 1.3085846
1.4486052
C -3.1214362 0.3273150
1.8666920
C 2.1010796 -1.1393235
4.8659051
C 3.2037306 -0.7161515
5.5881230
C 3.1236242 -0.5970869
6.9828719
C 1.9323653 -0.9051230
7.6390298
C 0.7904421 -1.3524867
6.9503469
C -4.6303372 -0.5165905
5.2963915
C -0.3978972 -1.7089549
7.7264729
N -1.5699648 -1.8875064
7.1422513
N -3.7340061 -1.2779243
5.8997048
C -3.9414551 -2.0910118
7.0525445
C -2.7474700 -2.4277330
7.7377713
C -2.7879553 -3.3301818
8.8090772
C -4.0064961 -3.8846411
9.1965394
C -5.1848080 -3.5521372
8.5205002
C -5.1585143 -2.6614016
7.4489183
Ni -1.9636698 -1.5334002
5.3463441
C -0.2706526 -1.8125718
9.2007873
C 0.5922626 -2.7527397
9.7910678
C 0.6586340 -2.8564342
11.1806142
C -0.1093230 -2.0076622
11.9847160
C -0.9485722 -1.0532398
11.3989664
C -1.0415141 -0.9622260
10.0117755
C -5.9258746 -0.2181409
5.9544583
C -5.9218343 0.4146761
7.2092179
C -7.1269220 0.6896081
7.8521215
C -8.3387458 0.3126773
7.2623411
C -8.3465716 -0.3181721
6.0138454
C -7.1456082 -0.5718541
5.3510256
H -0.8294142 -3.5141513 -
0.1669058
H 1.2646610 -4.1017399
1.0348496
H 1.6257958 -3.3569803
3.3760618
H -2.5926042 -2.1729808
0.9549596
H -6.0254114 1.4413114
4.1793126
H -5.7425214 2.4926048
1.9717166
H -3.8509169 1.7975407
0.4864429
H -2.2557556 0.0863935
1.2529767
H 2.1417373 -1.1473054
3.7784789
H 4.1187015 -0.4401691
5.0637813
H 3.9794586 -0.2406947
7.5553541
H 1.8796985 -0.7838111
8.7190378
H -1.8761311 -3.6253355
9.3195232
H -4.0296558 -4.5998180
10.0183480
H -6.1309781 -4.0068074
8.8128356
H -6.0751769 -2.4408295
6.9102630
H 1.1897287 -3.4135029
9.1614512
H 1.3145727 -3.5978784
11.6369894
H -0.0474781 -2.0841523
13.0704611
H -1.5297815 -0.3772838
12.0264239
H -1.7021421 -0.2267154
9.5509038
H -4.9744480 0.6973220
7.6700168
H -7.1230406 1.2010764
8.8148446
H -9.2794235 0.5187882
7.7733914
H -9.2906674 -0.6091083
5.5531016
H -7.1485241 -1.0641066
4.3774681

NiPh$_2$PhenTAA_2eOX_Quintet:
69 atoms
C -0.6697705 -3.1425714
0.8554464
C 0.5120297 -3.4748582
1.5340006
C 0.7413782 -3.0155153
2.8327802
C -0.2083210 -2.1906115
3.4578945
C -1.4231071 -1.8488784
2.7603177
C -1.6413831 -2.3454579
1.4645469
N -2.3316466 -1.1412862
3.5308564
N -0.1727884 -1.7488463
4.7706938
C 0.9431927 -1.5038297
5.5229842
C -3.3156872 -0.3061269
3.0773340
C -4.4406898 0.0435082
3.9333232
C -5.3386684 1.0250777
3.4420622
C -5.1976676 1.6137744
2.1929610
C -4.1078690 1.2693661
1.3776474
C -3.1793671 0.3422220
1.8225006
C 2.1674253 -1.1603953
4.8930517
C 3.2894054 -0.8089726
5.6259188
C 3.2195160 -0.7519239
7.0269069
C 2.0258374 -1.0460441
7.6712773
C 0.8570811 -1.4470760
6.9754229
C -4.6759581 -0.4940786
5.2614410
C -0.3413734 -1.7151527
7.7502792
N -1.5392610 -1.8533351
7.1431147
N -3.7338706 -1.2350925
5.8829047
C -3.9114730 -2.0818344
6.9610915
C -2.6820109 -2.4284944
7.6668184
C -2.7277062 -3.3904698
8.7008479
C -3.9319358 -3.9753919
9.0420091
C -5.1267266 -3.6382492
8.3564696
C -5.1198583 -2.7156496
7.3281510
Ni -1.9110006 -1.3273738
5.3557256
C -0.2781531 -1.7496000
9.2258263
C 0.5971094 -2.6362967
9.8878768
C 0.5861152 -2.7184187
11.2784542
C -0.2674659 -1.8972438
12.0239426
C -1.1242406 -0.9998799
11.3735076
C -1.1432668 -0.9357922
9.9838908
C -5.9179268 -0.1595058
5.9882249
C -5.8455636 0.3919362
7.2827997
C -7.0106535 0.6619450
7.9935325
C -8.2597267 0.3567012
7.4376820
C -8.3404204 -0.2028644
6.1574388
C -7.1799636 -0.4450917
5.4258324
H -0.8481361 -3.5335588 -
0.1460121
H 1.2471765 -4.1226636
1.0570491
H 1.6292782 -3.3341058
3.3771063
H -2.5848465 -2.1491541
0.9571967
H -6.1561782 1.3516385
4.0805386
H -5.9126418 2.3683325
1.8665988
H -3.9678882 1.7536684
0.4110147
H -2.2897474 0.1438696
1.2272671
H 2.2000942 -1.1175604
3.8057325
H 4.2116834 -0.5435190
5.1087529
H 4.0864839 -0.4415064
7.6092124
H 1.9788396 -0.9367346
8.7522302
H -1.8143764 -3.6869372
9.2094963
H -3.9595840 -4.7254741
9.8322571
H -6.0593081 -4.1329354
8.6275890
H -6.0362002 -2.4961271
6.7869158
H 1.2517816 -3.2855647
9.3053936
H 1.2453804 -3.4248664
11.7830628
H -0.2617729 -1.9526589
13.1126595
H -1.7731641 -0.3461576
11.9562986
H -1.8102660 -0.2387721
9.4749723
H -4.8717311 0.6275423
7.7138403
H -6.9505964 1.1172177
8.9821070
H -9.1706795 0.5598801
8.0009640
H -9.3112345 -0.4510038
5.7281493
H -7.2409356 -0.8941710
4.4338194

NiPh$_2$PhenTAA_3eOX_Doublet:
69 atoms
C -0.7192939 -3.1682272
0.9070716
C 0.4797682 -3.5051355
1.5950601
C 0.7142658 -3.0702939
2.8877014
C -0.2614767 -2.2734521
3.5298081
C -1.4926747 -1.9271780
2.8231305
C -1.6976253 -2.3924222
1.5036885
N -2.3428528 -1.1727303
3.5764215
N -0.2170450 -1.7704759
4.7965583
C 0.9002703 -1.4603404
5.5245776
C -3.2815910 -0.2845915
3.1240741
C -4.3549606 0.1363873
3.9973784
C -5.2069316 1.1493373
3.5294293

C -5.0518706 1.7283498
2.2653971
C -4.0164635 1.3059024
1.4184586
C -3.1404642 0.3219625
1.8443577
C 2.1256728 -1.1565321
4.8677050
C 3.2329086 -0.7294369
5.5807543
C 3.1459808 -0.5750914
6.9722935
C 1.9502342 -0.8636043
7.6385434
C 0.8072485 -1.3164374
6.9607121
C -4.6408660 -0.4680365
5.3040127
C -0.3789691 -1.6688553
7.7502217
N -1.5614850 -1.8169891
7.1716184
N -3.7323166 -1.2050058
5.9257639
C -3.9324511 -2.0239003
7.0688215
C -2.7363537 -2.3612637
7.7550703
C -2.7783983 -3.2725210
8.8241157
C -4.0000574 -3.8279358
9.2144323
C -5.1778409 -3.4955514
8.5389005
C -5.1504294 -2.6032588
7.4635446
Ni -1.9521648 -1.4402597
5.3730197
C -0.2294437 -1.8214661
9.2136421
C 0.6824283 -2.7474118
9.7529957
C 0.7775504 -2.8993881
11.1371020
C -0.0076614 -2.1082548
11.9889849
C -0.8970518 -1.1677842
11.4561756
C -1.0257026 -1.0339580
10.0756821
C -5.9568307 -0.2069425
5.9263596
C -6.0097436 0.3728112
7.2140529
C -7.2419739 0.6235428
7.8131954
C -8.4257655 0.2669615
7.1566783
C -8.3779865 -0.3178424
5.8823895
C -7.1495785 -0.5399299
5.2581996
H -0.8892326 -3.5737139 -
0.0910605
H 1.2027984 -4.1614505
1.1092326
H 1.5992759 -3.3976835
3.4318902
H -2.6436344 -2.2052491
0.9972361
H -6.0063244 1.5083047
4.1747736
H -5.7225243 2.5295943
1.9540853
H -3.8693294 1.7812286
0.4482946
H -2.2780096 0.0700704
1.2295101
H 2.1639577 -1.1756707
3.7797741
H 4.1511684 -0.4691966
5.0536406
H 4.0007729 -0.2021874
7.5370931
H 1.9032362 -0.7171431
8.7159085
H -1.8656347 -3.5859038
9.3220012
H -4.0222855 -4.5449722
10.0350018
H -6.1240760 -3.9517828
8.8295905
H -6.0647597 -2.4012238
6.9136343
H 1.2937527 -3.3638017
9.0926957
H 1.4692775 -3.6311920
11.5546627
H 0.0828806 -2.2210959
13.0696204
H -1.4846148 -0.5335499
12.1199833
H -1.7178898 -0.3020895
9.6577469

NiPh$_2$PhenTAA_3eOX_Quartet:
69 atoms
C -0.7319175 -3.2265907
0.9216310
C 0.4494801 -3.5589082
1.5996456
C 0.6862945 -3.0919286
2.8986926
C -0.2648338 -2.2637358
3.5106681
C -1.4719186 -1.9239772
2.8177709
C -1.7034245 -2.4195589
1.5270519
N -2.3553781 -1.1806852
3.5989545
N -0.2337734 -1.7782587
4.8170338
C 0.8857118 -1.4686192
5.5385843
C -3.2899653 -0.2934988
3.1414433
C -4.3777958 0.1348668
3.9999775
C -5.2230925 1.1601761
3.5229611
C -5.0733780 1.7195908
2.2540800
C -4.0332667 1.2841109
1.4216394
C -3.1499649 0.3070692
1.8629883
C 2.1084578 -1.1715780
4.8818021
C 3.2289630 -0.7578200
5.5909025
C 3.1611667 -0.5969145
6.9815586
C 1.9667687 -0.8636382
7.6506347
C 0.8137537 -1.3267921
6.9802848
C -4.6540749 -0.4403299
5.3070567
C -0.3653896 -1.6485469
7.7689460
N -1.5655303 -1.7714552
7.1792702
N -3.7191572 -1.1647638
5.9429163
C -3.9270962 -2.0337152
7.0225663
C -2.7055160 -2.3779472
7.7237124
C -2.7390274 -3.3371884
8.7503837
C -3.9471459 -3.9298760
9.0882660
C -5.1386740 -3.5940222
8.4044694
C -5.1332664 -2.6623917
7.3763358
Ni -1.9521562 -1.4057595
5.3894858
C -0.2411027 -1.7789900
9.2277944
C 0.7246445 -2.6461772
9.7937263
C 0.8001536 -2.7899684
11.1746120
C -0.0441543 -2.0378546
12.0058006
C -0.9826556 -1.1507959
11.4512182
C -1.0986755 -1.0348675
10.0732381
C -5.9469857 -0.1712865
5.9525775
C -5.9783401 0.3395127
7.2724371
C -7.1961716 0.5990265
7.8850041
C -8.3967478 0.3149169
7.2118060
C -8.3746881 -0.2050358
5.9085139
C -7.1603174 -0.4244342
5.2678822
H -0.9162762 -3.6290623 -
0.0745915
H 1.1780074 -4.2181154
1.1273172
H 1.5724872 -3.4157812
3.4428847
H -2.6482909 -2.2282996
1.0202093
H -6.0019942 1.5489690
4.1760405
H -5.7423204 2.5166042
1.9300446
H -3.8825155 1.7434401
0.4440656
H -2.2868399 0.0526197
1.2506014
H 2.1434142 -1.1925568
3.7940475
H 4.1455679 -0.5128319
5.0531609
H 4.0242703 -0.2307334
7.5371647
H 1.9169581 -0.6807475
8.7222920
H -1.8249041 -3.6454763
9.2504997
H -3.9701685 -4.6978289
9.8618094
H -6.0665607 -4.1069069
8.6587957
H -6.0477699 -2.4553766
6.8271184
H 1.3724251 -3.2352818
9.1439651
H 1.5175112 -3.4855326
11.6101988
H 0.0348255 -2.1355398
13.0890955
H -1.6098382 -0.5449582
12.1050671
H -1.8162903 -0.3386660
9.6381384
H -5.0442084 0.5701202
7.7855336
H -7.2234168 1.0353503
8.8834217
H -9.3516125 0.5083432
7.7018550
H -9.3088715 -0.4351185
5.3961426
H -7.1384012 -0.8369927
4.2587980

NiPh$_2$PhenTAA_3eOX_Sextet:
69 atoms
C -0.6948715 -3.2469416
0.9412788
C 0.4985134 -3.5318503
1.6196123
C 0.7336807 -3.0143781
2.8979105
C -0.2195110 -2.1713893
3.5002512
C -1.4514122 -1.8814247
2.8003650
C -1.6766242 -2.4407212
1.5322940
N -2.3702295 -1.1582508
3.5410132
N -0.1623201 -1.6686243
4.7852818
C 0.9517512 -1.4320701
5.5378529
C -3.3427777 -0.3256494
3.0742848
C -4.4611092 0.0696704
3.9302205
C -5.3283215 1.0666151
3.4216606
C -5.1911243 1.6117808
2.1461121
C -4.1262123 1.2102964
1.3248181
C -3.2104828 0.2805584
1.7920763
C 2.1796830 -1.0965480
4.9080510
C 3.3024513 -0.7405553
5.6417149
C 3.2263338 -0.6662319
7.0407588
C 2.0293544 -0.9618274
7.6868369
C 0.8650021 -1.3789848
6.9941932
C -4.7171431 -0.4376996
5.2716007
C -0.3241317 -1.6814159
7.7792977
N -1.5366846 -1.7623202
7.1848580
N -3.7542779 -1.1201763
5.9350812
C -3.9193854 -1.9782575
7.0008611
C -2.6899345 -2.3363339
7.7006154
C -2.7407371 -3.3184971
8.7091662
C -3.9589743 -3.8959964
9.0674532
C -5.1473079 -3.5536547
8.3918922
C -5.1291936 -2.6286898
7.3566154
Ni -1.9194332 -1.2001815
5.3986035
C -0.2162130 -1.8137100
9.2427215
C 0.7316726 -2.6930012
9.8179765
C 0.7877683 -2.8452115
11.1988700
C -0.0596867 -2.0887200
12.0279699
C -0.9882373 -1.1970470
11.4672848
C -1.0847033 -1.0735851
10.0886738
C -5.9866458 -0.1336837
5.9493150
C -5.9776055 0.3613047
7.2828488
C -7.1712535 0.6298582
7.9370017
C -8.3938856 0.3757990
7.2957423
C -8.4154368 -0.1365369
5.9854826
C -7.2254458 -0.3652957
5.3041972
H -0.8769619 -3.6786953 -
0.0430427
H 1.2406370 -4.1854432
1.1608851

H 1.6332233 -3.2993075 3.4423802
H -2.6315307 -2.2891370 1.0296632
H -6.1181306 1.4541613 4.0617115
H -5.8894913 2.3772709 1.8079416
H -3.9902913 1.6583296 0.3399080
H -2.3277322 0.0510323 1.1971395
H 2.2119696 -1.0509670 3.8202155
H 4.2267611 -0.4795607 5.1251811
H 4.0890811 -0.3422084 7.6225550
H 1.9836717 -0.8339902 8.7661359
H -1.8249638 -3.6627165 9.1833690
H -3.9813598 -4.6539547 9.8508595
H -6.0809347 -4.0522473 8.6526507
H -6.0367386 -2.4360889 6.7900131
H 1.3824488 -3.2855608 9.1745183
H 1.4906511 -3.5529140 11.6389609
H 0.0083621 -2.1933344 13.1113379
H -1.6240588 -0.5945264 12.1157852
H -1.7942169 -0.3717486 9.6493404
H -5.0255894 0.5793445 7.7679644
H -7.1609640 1.0514462 8.9420385
H -9.3324379 0.5798221 7.8124147
H -9.3678454 -0.3596758 5.5038206
H -7.2434561 -0.7787522 4.2955374

B3-LYP/def2-TZVP/disp3: NiH_2PhenTAA:

NiH_2PhenTAA_2eRED_CSS:
49 atoms
C -0.8156614 2.0588637 1.9716784
C 0.1267629 2.5522674 2.8758321
C 0.3984666 1.8496333 4.0456875
C -0.2495007 0.6386466 4.3459611
C -1.2217386 0.1299430 3.4137984
C -1.4792426 0.8666278 2.2442929
N -1.8994175 -0.9901308 3.8452750
N -0.1515067 -0.0760535 5.5204949
C 0.9362553 -0.0683173 6.3549256
C -2.5373564 -1.8853619 3.0260864
C -3.5645913 -2.7592961 3.5736474
C -4.2452629 -3.6179911 2.6596942
C -3.9229251 -3.7088695 1.3240767
C -2.8607197 -2.9365621 0.8209241
C -2.1914822 -2.0652782 1.6694878
C 2.2385362 0.2520134 5.9146468
C 3.3377241 0.3063849 6.7607743
C 3.1734562 0.0041088 8.1244682
C 1.9341299 -0.3848843 8.5813810
C 0.7847692 -0.4839339 7.7416274
C -3.8910741 -2.8444832 4.9380211
C -0.3960829 -1.0161226 8.2871452
N -1.5310817 -1.2667989 7.6107912
N -3.2793583 -2.1816582 5.9354784
C -3.6538552 -2.2470000 7.2422476
C -2.6751653 -1.7348641 8.1800423
C -2.9861596 -1.7500314 9.5553411
C -4.1918857 -2.2504456 10.0155289
C -5.1451825 -2.7492127 9.1021398
C -4.8763084 -2.7389273 7.7443484
Ni -1.6760227 -1.1913039 5.7211303
H -0.3438625 -1.3416650 9.3248800
H -4.6384501 -3.5883727 5.2094146
H -1.0522604 2.6097505 1.0664302
H 0.6342564 3.4929439 2.6847253
H 1.0920492 2.2626624 4.7652677
H -2.2439635 0.5163741 1.5645560
H -5.0422319 -4.2401637 3.0594638
H -4.4755332 -4.3807560 0.6736913
H -2.5483747 -3.0297610 -0.2143107
H -1.3575194 -1.4962228 1.2811024
H 2.3794436 0.4583074 4.8621548
H 4.3147197 0.5608050 6.3624017
H 4.0144055 0.0618067 8.8095121
H 1.8118368 -0.6538544 9.6277012
H -2.2790571 -1.3287477 10.2601265
H -4.4143203 -2.2310065 11.0779290
H -6.1028354 -3.1142785 9.4601565
H -5.6318215 -3.0827839 7.0478231

NiH_2PhenTAA_2eRED_OSS:
49 atoms
C -0.7807371 2.0434082 1.9463639
C 0.1593065 2.5356296 2.8482819
C 0.4240900 1.8360240 4.0245426
C -0.2308130 0.6317999 4.3293725
C -1.2022553 0.1234304 3.3979065
C -1.4519148 0.8537749 2.2246672
N -1.8875123 -0.9935148 3.8358787
N -0.1406970 -0.0798303 5.5102054
C 0.9400524 -0.0618046 6.3551232
C -2.5402132 -1.8824439 3.0198156
C -3.5766522 -2.7421978 3.5724759
C -4.2729860 -3.5945947 2.6668481
C -3.9613721 -3.6919354 1.3268175
C -2.8977161 -2.9313910 0.8174434
C -2.2103589 -2.0647382 1.6609551
C 2.2440629 0.2653136 5.9295125
C 3.3350743 0.3293783 6.7901212
C 3.1596446 0.0338364 8.1507270
C 1.9142915 -0.3572976 8.5960902
C 0.7765548 -0.4647125 7.7442235
C -3.9068518 -2.8145398 4.9391316
C -0.4127539 -0.9865232 8.2875005
N -1.5369850 -1.2664917 7.6014084
N -3.2717559 -2.1743395 5.9389944
C -3.6521767 -2.2571588 7.2514838
C -2.6782632 -1.7474877 8.1847411
C -2.9748775 -1.7785049 9.5585156
C -4.1815970 -2.2988413 10.0221800
C -5.1270060 -2.7935183 9.1163062
C -4.8644711 -2.7671824 7.7479777
Ni -1.6725776 -1.1867906 5.7151146
H -0.3722023 -1.2836447 9.3338620
H -4.6763362 -3.5355072 5.2091222
H -1.0088985 2.5870185 1.0344930
H 0.6767717 3.4698736 2.6520500
H 1.1188007 2.2497295 4.7423236
H -2.2152138 0.5042583 1.5433009
H -5.0730014 -4.2076717 3.0741521
H -4.5254412 -4.3591258 0.6816731
H -2.5971801 -3.0236704 -0.2215015
H -1.3752513 -1.5046157 1.2630046
H 2.3976714 0.4687108 4.8785366
H 4.3138668 0.5916608 6.4009983
H 3.9927984 0.0980752 8.8444564
H 1.7808229 -0.6213896 9.6421507
H -2.2664236 -1.3641894 10.2652337
H -4.3923626 -2.3009033 11.0872979
H -6.0757536 -3.1815499 9.4743687
H -5.6182008 -3.1178151 7.0537523

NiH_2PhenTAA_2eRED_Triplet:
49 atoms
C -0.7480827 2.0248425 1.9248887
C 0.1894527 2.5162004 2.8247869
C 0.4483652 1.8195818 4.0070204
C -0.2141828 0.6223942 4.3155742
C -1.1838462 0.1144705 3.3854171
C -1.4260429 0.8372829 2.2078762
N -1.8726889 -1.0013226 3.8308077
N -0.1315507 -0.0896893 5.5004284
C 0.9431925 -0.0600246 6.3547563
C -2.5399758 -1.8828039 3.0162086
C -3.5868373 -2.7250771 3.5721210
C -4.3000187 -3.5701819 2.6753932
C -3.9971136 -3.6748785 1.3315244
C -2.9320459 -2.9264647 0.8163276
C -2.2272035 -2.0638806 1.6545766
C 2.2475471 0.2772820 5.9429222
C 3.3306763 0.3496639 6.8176015
C 3.1444615 0.0607399 8.1747671
C 1.8929633 -0.3304818 8.6099262
C 0.7677357 -0.4466234 7.7454443
C -3.9252526 -2.7725634 4.9400622
C -0.4353437 -0.9463175 8.2847415
N -1.5380490 -1.2734643 7.5856307
N -3.2542279 -2.1724043 5.9406810
C -3.6449662 -2.2689480 7.2580762
C -2.6779546 -1.7622568 8.1849871
C -2.9562947 -1.8102270 9.5599334
C -4.1658491 -2.3507401 10.0250206
C -5.1029344 -2.8419411 9.1267934
C -4.8482429 -2.8017553 7.7465711
Ni -1.6673817 -1.1845171 5.7086123
H -0.4221523 -1.1831846 9.3459141
H -4.7392911 -3.4415818 5.2086422
H -0.9685256 2.5617226 1.0071709
H 0.7156122 3.4445668 2.6239394
H 1.1439583 2.2339182 4.7230621
H -2.1876104 0.4882142 1.5249040
H -5.1039795 -4.1728375 3.0897439
H -4.5713681 -4.3381453 0.6915961
H -2.6421016 -3.0188184 -0.2256385
H -1.3913520 -1.5124352 1.2472931
H 2.4133649 0.4784083 4.8937788

H 4.3112371 0.6181024
6.4371653
H 3.9704916 0.1297795
8.8762727
H 1.7481757 -0.5884861
9.6557395
H -2.2484695 -1.4001919
10.2683475
H -4.3670685 -2.3715978
11.0919888
H -6.0432240 -3.2501218
9.4853938
H -5.6010053 -3.1572066
7.0552083

NiH_2PhenTAA_2eRED_Quintet:
49 atoms
C -0.4815226 1.8575650
1.7661849
C 0.4286762 2.3598376
2.6830248
C 0.6210451 1.6927073
3.8962971
C -0.0649128 0.5161940
4.2243586
C -1.0247851 -0.0104718
3.2662761
C -1.1956418 0.6945093
2.0616999
N -1.7821877 -1.0824360
3.6984209
N -0.0225153 -0.1266149
5.4601873
C 0.9812545 0.0127591
6.4049907
C -2.5975194 -1.8729696
2.9275958
C -3.7162331 -2.5943674
3.5433091
C -4.5760213 -3.3177064
2.6648520
C -4.3544769 -3.4528141
1.3078474
C -3.2218300 -2.8558465
0.7451606
C -2.3794760 -2.0920050
1.5515831
C 2.3021148 0.3468248
6.0595934
C 3.3189558 0.5529904
6.9941728
C 3.0299551 0.4231540
8.3545625
C 1.7615108 0.0266576
8.7353918
C 0.7112131 -0.2592260
7.8161770
C -4.0307361 -2.6735724
4.9258553
C -0.4788188 -0.8097567
8.3681311
N -1.5027982 -1.3346657
7.6774681
N -3.2862184 -2.2223740
5.9592928
C -3.6042276 -2.3997911
7.2885172
C -2.6202417 -1.9101321
8.2351035
C -2.8752261 -2.0631310
9.6082843
C -4.0511974 -2.6722661
10.0697380
C -4.9940892 -3.1367653
9.1640648
C -4.7706096 -3.0029578
7.7850709
Ni -1.5822372 -1.2638089
5.6841968
H -0.5017473 -0.8560596
9.4568484
H -4.9348294 -3.2369798
5.1533989
H -0.6544797 2.3647213
0.8212282
H 0.9852610 3.2688897
2.4755979
H 1.2896004 2.1239217
4.6276099
H -1.9447099 0.3535863
1.3622016
H -5.4331781 -3.8151945
3.1108989
H -5.0441816 -4.0273265
0.6966792
H -2.9909002 -2.9821870 -
0.3080696
H -1.4956963 -1.6551956
1.1079534
H 2.5378312 0.4447326
5.0089678
H 4.3166305 0.8157718
6.6573059
H 3.7902494 0.6128082
9.1065850
H 1.5494976 -0.1214203
9.7909511
H -2.1507434 -1.7024719
10.3275679
H -4.2202529 -2.7719830
11.1380719
H -5.9096231 -3.6036816
9.5156340
H -5.5249314 -3.3554521
7.0931609

NiH_2PhenTAA_1eRED_Doublet:
49 atoms
C -0.7646272 2.0327804
1.9353861
C 0.1761435 2.5246266
2.8371803
C 0.4300593 1.8385899
4.0183790
C -0.2320644 0.6393413
4.3166104
C -1.1949837 0.1358722
3.3936341
C -1.4481061 0.8566283
2.2179835
N -1.8975684 -0.9729077
3.8471985
N -0.1613042 -0.0647822
5.5115050
C 0.9146968 -0.0632134
6.3456807
C -2.5335161 -1.8666794
3.0408277
C -3.5575726 -2.7320149
3.5788240
C -4.2487291 -3.5983570
2.6928191
C -3.9427944 -3.6935610
1.3563468
C -2.8826584 -2.9206640
0.8525816
C -2.2036955 -2.0442511
1.6730865
C 2.2246114 0.2696469
5.9161803
C 3.3008460 0.3120776
6.7778021
C 3.1359002 0.0107237
8.1407073
C 1.8947991 -0.3815701
8.5818327
C 0.7672259 -0.4679147
7.7245987
C -3.8983502 -2.7940738
4.9493425
C -0.4315182 -0.9781172
8.2730162
N -1.5361242 -1.2575833
7.5945254
N -3.2692411 -2.1655483
5.9329045
C -3.6462003 -2.2613783
7.2596611
C -2.6831709 -1.7570933
8.1828813
C -2.9699169 -1.7910112
9.5564376
C -4.1655552 -2.3170422
10.0160025
C -5.1119937 -2.8122495
9.1088098
C -4.8561774 -2.7781286
7.7482858
Ni -1.6918707 -1.1503680
5.7153121
H -0.4068868 -1.2192094
9.3329865
H -4.7069265 -3.4726233
5.2098477
H -0.9906076 2.5776617
1.0258036
H 0.6918521 3.4573490
2.6385564
H 1.1130744 2.2607788
4.7416536
H -2.2221597 0.5172734
1.5444803
H -5.0378684 -4.2176510
3.1080695
H -4.4905638 -4.3703651
0.7113984
H -2.5800011 -3.0208518 -
0.1839755
H -1.3711716 -1.4860533
1.2703759
H 2.3834519 0.4730333
4.8671713
H 4.2818774 0.5645910
6.3906172
H 3.9747748 0.0604340
8.8247879
H 1.7577893 -0.6572404
9.6230136
H -2.2641116 -1.3773151
10.2648973
H -4.3761998 -2.3240090
11.0791239
H -6.0567855 -3.2030321
9.4683121
H -5.6110808 -3.1285486
7.0565371

NiH_2PhenTAA_1eRED_Quartet:
49 atoms
C -0.5977319 1.9558669
1.8391912
C 0.3328087 2.4388621
2.7504556
C 0.5523198 1.7547224
3.9414652
C -0.1245615 0.5670818
4.2478439
C -1.0905656 0.0653977
3.3034163
C -1.3037995 0.7918336
2.1232295
N -1.8534532 -1.0124175
3.7375123
N -0.0715916 -0.0958114
5.4737571
C 0.9606267 -0.0133422
6.3807973
C -2.5846512 -1.8497199
2.9627040
C -3.6428508 -2.6601894
3.5481782
C -4.4173549 -3.4753990
2.6814286
C -4.1724767 -3.5914426
1.3335414
C -3.0906186 -2.8827564
0.7873382
C -2.3345082 -2.0432424
1.5764080
C 2.2795364 0.3089648
5.9971100
C 3.3221586 0.4239492
6.9019667
C 3.0870632 0.2250829
8.2659035
C 1.8241338 -0.1491529
8.6732330
C 0.7396019 -0.3312772
7.7780602
C -3.9765262 -2.7446819
4.9309115
C -0.4645122 -0.8456723
8.3489883
N -1.5103116 -1.3029923
7.6724991
N -3.3043812 -2.2203474
5.9424089
C -3.6241521 -2.3543631
7.2823207
C -2.6463630 -1.8503521
8.2180646
C -2.9275379 -1.9542896
9.5951231
C -4.1033436 -2.5181481
10.0511176
C -5.0535579 -3.0020930
9.1397909
C -4.8091639 -2.9179857
7.7804133
Ni -1.6401854 -1.1767881
5.6946165
H -0.4655986 -0.9144704
9.4361426
H -4.8498758 -3.3597310
5.1490689
H -0.7998229 2.4938628
0.9196694
H 0.8697497 3.3603516
2.5550682
H 1.2262934 2.1773106
4.6725796
H -2.0772042 0.4692282
1.4412920
H -5.2243570 -4.0502250
3.1252075
H -4.7836064 -4.2374070
0.7147249
H -2.8334009 -3.0025633 -
0.2596325
H -1.4859954 -1.5377246
1.1393593
H 2.4837609 0.4455480
4.9449073
H 4.3175130 0.6616321
6.5435195
H 3.8863366 0.3349020
8.9895805
H 1.6447383 -0.3512718
9.7246289
H -2.2158047 -1.5682739
10.3134574
H -4.2955145 -2.5733491
11.1167343
H -5.9840507 -3.4286553
9.4961283
H -5.5622110 -3.2724612
7.0877692

NiH_2PhenTAA_1eRED_Sextet:
49 atoms
C -0.6453995 1.8544931
1.8980522
C 0.3030688 2.3513964
2.8087059
C 0.5821620 1.6659203
3.9706109
C -0.0616628 0.4411910
4.2809998
C -1.0538815 -0.0783374
3.3289291
C -1.3092780 0.6750986
2.1548823
N -1.7090521 -1.1966103
3.7130706
N 0.0787298 -0.2613686
5.4275308
C 1.0647897 -0.0521584
6.3950673
C -2.5685346 -1.9528641
2.9124390

C -3.7129354 -2.6070327
3.5165574
C -4.5914536 -3.2782205
2.6173615
C -4.3329596 -3.4165700
1.2702129
C -3.1606504 -2.8840452
0.7248001
C -2.3067911 -2.1614385
1.5554453
C 2.3735451 0.2871078
6.0410409
C 3.3702193 0.5331628
6.9830648
C 3.0453057 0.4442613
8.3401714
C 1.7789526 0.0549113
8.7217938
C 0.7492174 -0.2728493
7.7928679
C -4.0350541 -2.6679706
4.8976347
C -0.4572514 -0.7969914
8.3266137
N -1.4755653 -1.3281466
7.6238308
N -3.2566591 -2.2578886
5.9171883
C -3.5841096 -2.3904195
7.2534926
C -2.6097267 -1.8817894
8.1870067
C -2.8728460 -1.9873649
9.5593804
C -4.0533787 -2.5690843
10.0268012
C -4.9916342 -3.0586857
9.1279122
C -4.7572939 -2.9707720
7.7538569
Ni -1.5210750 -1.3935813
5.6702427
H -0.5110904 -0.8109317
9.4119680
H -4.9813713 -3.1504996
5.1272611
H -0.8796932 2.4162505
1.0012866
H 0.7980615 3.2953638
2.6121996
H 1.2676063 2.0873629
4.6902735
H -2.0784951 0.3345933
1.4783349
H -5.4850953 -3.7310024
3.0338961
H -5.0318032 -3.9589341
0.6427933
H -2.9160004 -3.0230821 -
0.3207519
H -1.3917598 -1.7549018
1.1452157
H 2.6179467 0.3429373
4.9884161
H 4.3719786 0.7905905
6.6627757
H 3.7916619 0.6586117
9.0972792
H 1.5529212 -0.0483650
9.7779109
H -2.1545608 -1.6093668
10.2742622
H -4.2327485 -2.6327988
11.0941548
H -5.9107181 -3.5082132
9.4866170
H -5.5006382 -3.3549682
7.0685431

NiH$_2$PhenTAA_0eNeutral_CSS:
C -0.7452847 2.0320150
1.9171490
C 0.1927697 2.5236420
2.8172750
C 0.4433042 1.8428313
4.0026312
C -0.2163429 0.6445375
4.2894217
C -1.1716997 0.1440086
3.3730567
C -1.4341899 0.8589388
2.2011082
N -1.8848969 -0.9682407
3.8414228
N -0.1576165 -0.0635815
5.4977840
C 0.9031558 -0.0645786
6.3329745
C -2.5220540 -1.8572424
3.0499210
C -3.5359730 -2.7283442
3.5885100
C -4.2377958 -3.6086411
2.7266706
C -3.9473319 -3.7082739
1.3925818
C -2.9008738 -2.9188054
0.8775770
C -2.2206389 -2.0267376
1.6669396
C 2.2195985 0.2969749
5.9224495
C 3.2762582 0.3127521
6.7969865
C 3.1115038 -0.0162473
8.1565022
C 1.8720863 -0.4128851
8.5814465
C 0.7595379 -0.4809772
7.7051586
C -3.8839512 -2.7647843
4.9587110
C -0.4516016 -0.9688499
8.2482118
N -1.5281965 -1.2614304
7.5720393
N -3.2471684 -2.1615275
5.9243301
C -3.6421360 -2.2675966
7.2744127
C -2.6925513 -1.7702214
8.1846438
C -2.9604018 -1.8044426
9.5523225
C -4.1576550 -2.3375444
10.0121954
C -5.0975044 -2.8299568
9.1113024
C -4.8448086 -2.7916399
7.7460915
Ni -1.6972763 -1.1198202
5.7046462
H -0.4529528 -1.1425500
9.3212332
H -4.7396343 -3.3849767
5.2131512
H -0.9706416 2.5770294
1.0091707
H 0.7061488 3.4558882
2.6182400
H 1.1156050 2.2740712
4.7295192
H -2.2156053 0.5285435
1.5328150
H -5.0141475 -4.2307472
3.1581267
H -4.4868082 -4.3951169
0.7547992
H -2.6113963 -3.0257162 -
0.1613524
H -1.4005549 -1.4676175
1.2440069
H 2.3940591 0.5189772
4.8811433
H 4.2609989 0.5694718
6.4241742
H 3.9500071 0.0171664
8.8386672
H 1.7240596 -0.7068326
9.6146865
H -2.2544120 -1.3970888
10.2625806
H -4.3641063 -2.3511242
11.0745564
H -6.0377897 -3.2279708
9.4702762
H -5.5980949 -3.1487381
7.0577325

NiH$_2$PhenTAA_0eNeutral_Triplet:
49 atoms
C -0.8777854 2.0593992
2.0220850
C 0.0765654 2.5592642
2.9377746
C 0.3778844 1.8685580
4.0846598
C -0.2576815 0.6345798
4.3680887
C -1.2397163 0.1205260
3.4264007
C -1.5242219 0.8724740
2.2598598
N -1.8414661 -1.0239402
3.8214466
N -0.1095598 -0.1177947
5.4816249
C 0.9667321 -0.0838753
6.3420686
C -2.5156877 -1.9059332
3.0045613
C -3.5245696 -2.7600064
3.5596074
C -4.2057373 -3.6381769
2.6874905
C -3.8969598 -3.7336154
1.3479534
C -2.8546129 -2.9570383
0.8297762
C -2.1788420 -2.0710362
1.6472902
C 2.2616044 0.2529081
5.9029486
C 3.3401868 0.2848560
6.7664715
C 3.1667792 -0.0378773
8.1169930
C 1.9231782 -0.4322968
8.5604822
C 0.8010506 -0.4975409
7.7046965
C -3.8611484 -2.8280459
4.9459454
C -0.4079254 -1.0224951
8.2546676
N -1.5174933 -1.2556615
7.5881450
N -3.2639148 -2.1690772
5.9148025
C -3.6439535 -2.2335771
7.2465367
C -2.6843192 -1.7317463
8.1659216
C -2.9851889 -1.7192304
9.5370674
C -4.1966381 -2.2043462
9.9887188
C -5.1442719 -2.6998365
9.0808749
C -4.8751758 -2.7074365
7.7264390
Ni -1.6366393 -1.2199222
5.6956980
H -0.3654578 -1.3040046
9.3039341
H -4.6410200 -3.5389694
5.2074836
H -1.1292985 2.6394982
1.1432114
H 0.5456242 3.5169343
2.7504526
H 1.0617148 2.2884360
4.8076315
H -2.2959575 0.5301337
1.5861268
H -4.9863365 -4.2661395
3.1017873
H -4.4375989 -4.4221879
0.7115438
H -2.5637411 -3.0549696 -
0.2085525
H -1.3488406 -1.5068794
1.2464673
H 2.4152872 0.4637502
4.8542655
H 4.3201307 0.5480415
6.3886244
H 4.0046186 -0.0051659
8.8013903
H 1.7925669 -0.7208079
9.5973184
H -2.2802410 -1.2996195
10.2420335
H -4.4246856 -2.1779928
11.0466560
H -6.1018459 -3.0548674
9.4399385
H -5.6303915 -3.0512491
7.0325906

NiH$_2$PhenTAA_0eNeutral_Quintet:
49 atoms
C -0.6032256 1.8372094
1.8392690
C 0.3304743 2.3445958
2.7633038
C 0.5816672 1.6830875
3.9440118
C -0.0683477 0.4702723
4.2586601
C -1.0420657 -0.0596570
3.2992535
C -1.2782753 0.6735495
2.1048514
N -1.7166845 -1.1600024
3.6930852
N 0.0379196 -0.2083088
5.4394536
C 1.0204827 -0.0213631
6.3963781
C -2.5662867 -1.9314140
2.9103511
C -3.7026468 -2.5802726
3.5227361
C -4.5802219 -3.2707290
2.6480189
C -4.3299962 -3.4238572
1.3005694
C -3.1638244 -2.8918497
0.7451806
C -2.3088712 -2.1547358
1.5501846
C 2.3363702 0.3352903
6.0442868
C 3.3228136 0.5466991
6.9896224
C 3.0296260 0.4163971
8.3479997
C 1.7653252 0.0111242
8.7209350
C 0.7439155 -0.2636341
7.7863407
C -4.0363434 -2.6251501
4.9095414
C -0.4764988 -0.7939781
8.3468960
N -1.4466998 -1.3529663
7.6828546
N -3.2620410 -2.2273796
5.9336268
C -3.5744913 -2.3745328
7.2645088
C -2.6039659 -1.9105317
8.2189783
C -2.8600226 -2.0527209
9.5897379
C -4.0338176 -2.6217227
10.0404368

C -4.9888039 -3.0690088
9.1145995
C -4.7645723 -2.9513225
7.7604922
Ni -1.5092877 -1.3844168
5.6695438
H -0.5336483 -0.7418887
9.4333688
H -4.9878437 -3.0972739
5.1325905
H -0.8201071 2.3899772
0.9340692
H 0.8278189 3.2853094
2.5630130
H 1.2485292 2.1260621
4.6678244
H -2.0458874 0.3319632
1.4275798
H -5.4670531 -3.7244892
3.0749704
H -5.0247572 -3.9809254
0.6844865
H -2.9230844 -3.0520794 -
0.2974952
H -1.3938063 -1.7608712
1.1300768
H 2.5852751 0.4129418
4.9957659
H 4.3233808 0.8073117
6.6678209
H 3.7877845 0.6012983
9.0975605
H 1.5443456 -0.1338846
9.7721940
H -2.1254504 -1.7196171
10.3110026
H -4.2141577 -2.7241276
11.1025147
H -5.9111857 -3.5148468
9.4659015
H -5.5167565 -3.3075306
7.0710479

$NiH_2PhenTAA$_1eOX_Doublet:
49 atoms
C -0.8023403 2.0120691
1.9701892
C 0.1513065 2.5114506
2.8849916
C 0.4299256 1.8384769
4.0484432
C -0.2282366 0.6223408
4.3371825
C -1.2042450 0.1114421
3.4013369
C -1.4737706 0.8417319
2.2224471
N -1.8271353 -1.0236249
3.8208043
N -0.1078762 -0.1239077
5.4689532
C 0.9580357 -0.0794335
6.3369438
C -2.5140925 -1.8960764
3.0093343
C -3.5448881 -2.7179141
3.5566272
C -4.2676894 -3.5771994
2.7063650
C -3.9667937 -3.6907461
1.3661283
C -2.9066397 -2.9425140
0.8477104
C -2.1990573 -2.0651984
1.6469675
C 2.2575005 0.2663252
5.9176845
C 3.3132597 0.3112844
6.8079196
C 3.1244505 0.0188640
8.1612247
C 1.8721127 -0.3652312
8.5898959
C 0.7816942 -0.4543570
7.7028714
C -3.8937631 -2.7451744
4.9441967
C -0.4490797 -0.9428960
8.2452956
N -1.4993499 -1.2817795
7.5674563
N -3.2313020 -2.1882179
5.9076745
C -3.6262554 -2.2744108
7.2636480
C -2.6783831 -1.7782111
8.1720509
C -2.9495861 -1.7978845
9.5405006
C -4.1509184 -2.3159092
9.9956813
C -5.0929079 -2.8091118
9.0929196
C -4.8369504 -2.7859843
7.7317778
Ni -1.6402844 -1.1968110
5.6870540
H -0.4646763 -1.0683154
9.3242230
H -4.7787275 -3.3252787
5.1900285
H -1.0381557 2.5833652
1.0820746
H 0.6341384 3.4591872
2.6863776
H 1.1068443 2.2669121
4.7719253
H -2.2508096 0.5089883
1.5510474
H -5.0657637 -4.1751334
3.1297381
H -4.5250570 -4.3664214
0.7330947
H -2.6229326 -3.0557795 -
0.1908979
H -1.3593385 -1.5295485
1.2302140
H 2.4395688 0.4579919
4.8708595
H 4.3002814 0.5657541
6.4431433
H 3.9517040 0.0679079
8.8556120
H 1.7176167 -0.6265424
9.6298709
H -2.2451818 -1.3943831
10.2539017
H -4.3634172 -2.3209964
11.0563433
H -6.0363593 -3.1968619
9.4530963
H -5.5914744 -3.1462378
7.0470716

$NiH_2PhenTAA$_1eOX_Quartet:
49 atoms
C -0.6647640 1.9442855
1.8694684
C 0.2750168 2.4438231
2.7885444
C 0.5273775 1.7775167
3.9661444
C -0.1291332 0.5677418
4.2681397
C -1.1045109 0.0505217
3.3181090
C -1.3476415 0.7810757
2.1339564
N -1.8025717 -1.0492919
3.7292400
N -0.0253564 -0.1196358
5.4500730
C 0.9925606 -0.0172041
6.3684190
C -2.5645098 -1.8783620
2.9482008
C -3.6288848 -2.6475578
3.5380466
C -4.4203197 -3.4566049
2.6966682
C -4.1668693 -3.5860685
1.3483621
C -3.0732257 -2.9118258
0.7983429
C -2.2957158 -2.0796528
1.5770869
C 2.3070199 0.3315807
5.9961839
C 3.3234289 0.4356850
6.9249391
C 3.0771999 0.2008872
8.2793397
C 1.8156407 -0.1970419
8.6669787
C 0.7589691 -0.3530406
7.7470069
C -3.9568123 -2.7246507
4.9382016
C -0.4565626 -0.8938816
8.3024878
N -1.4965757 -1.2802289
7.6369367
N -3.2865458 -2.2037648
5.9173540
C -3.6178432 -2.3367149
7.2830150
C -2.6593854 -1.8415537
8.2035337
C -2.9231200 -1.9173307
9.5727323
C -4.1040894 -2.4770290
10.0299609
C -5.0469718 -2.9623101
9.1246175
C -4.8073785 -2.8883532
7.7628817
Ni -1.6052426 -1.2084027
5.6666163
H -0.4392850 -1.0171720
9.3843336
H -4.8336028 -3.3313883
5.1588480
H -0.8824980 2.5008238
0.9675070
H 0.7759786 3.3816513
2.5872705
H 1.1958785 2.2140770
4.6923223
H -2.1224527 0.4524491
1.4583246
H -5.2402298 -4.0103662
3.1378834
H -4.7863983 -4.2240960
0.7333472
H -2.8232436 -3.0471513 -
0.2462391
H -1.4312329 -1.6023382
1.1404798
H 2.5306654 0.4807301
4.9504256
H 4.3205908 0.6947251
6.5924504
H 3.8688843 0.3005909
9.0089980
H 1.6272216 -0.4216454
9.7099380
H -2.2146802 -1.5260019
10.2894669
H -4.2980566 -2.5256975
11.0931546
H -5.9746476 -3.3877163
9.4834797
H -5.5610748 -3.2480848
7.0764218

$NiH_2PhenTAA$_1eOX_Sextet:
49 atoms
C -0.6837940 1.8693079
1.8571350
C 0.2205099 2.4080701
2.7821393
C 0.4836305 1.7584225
3.9786231
C -0.1212568 0.5349157
4.2807791
C -1.0670442 -0.0272825
3.3272860
C -1.3268669 0.6838937
2.1281321
N -1.7110707 -1.1465043
3.7327372
N -0.0142104 -0.1383115
5.4849107
C 0.9977804 -0.0191489
6.3889780
C -2.5366942 -1.9423113
2.9382343
C -3.6849568 -2.5905568
3.5278608
C -4.5342531 -3.3030547
2.6410014
C -4.2353736 -3.4856886
1.3007438
C -3.0592418 -2.9622553
0.7810546
C -2.2285954 -2.1951253
1.6041592
C 2.3047529 0.3749563
6.0148118
C 3.3114918 0.5235036
6.9385742
C 3.0718376 0.3033917
8.3068653
C 1.8342196 -0.1301715
8.7042255
C 0.7758973 -0.3539863
7.7851487
C -4.0415755 -2.6341004
4.8968015
C -0.4104362 -0.9003933
8.3481835
N -1.4394049 -1.3656629
7.6741178
N -3.2472699 -2.2147262
5.9453874
C -3.5837969 -2.3348056
7.2369063
C -2.5838910 -1.8885543
8.2194335
C -2.8600959 -2.0109894
9.5897903
C -4.0566028 -2.5341513
10.0176307
C -5.0352703 -2.9542677
9.0767282
C -4.8156049 -2.8577397
7.7331152
Ni -1.5144937 -1.3519718
5.6807352
H -0.4218325 -0.9531219
9.4342928
H -4.9698191 -3.1393368
5.1292488
H -0.9089327 2.4075835
0.9461441
H 0.6943719 3.3596880
2.5799333
H 1.1287789 2.2300849
4.7044874
H -2.0830830 0.3170289
1.4512341
H -5.4362620 -3.7462379
3.0448113
H -4.9072889 -4.0561021
0.6733689
H -2.7832740 -3.1327986 -
0.2505475
H -1.3037890 -1.8050467
1.2021076
H 2.5209390 0.5226451
4.9673723
H 4.3017246 0.8060513
6.6042818
H 3.8626730 0.4453704
9.0304142
H 1.6537446 -0.3420010
9.7510201
H -2.1339703 -1.6865146
10.3218829

H -4.2614092 -2.6227215 11.0758454
H -5.9733603 -3.3558367 9.4376243
H -5.5825306 -3.1774362 7.0442485

NiH$_2$PhenTAA_2eOX_CSS:
49 atoms
C -0.8569607 2.0036963 2.0113606
C 0.1147533 2.5122525 2.9432580
C 0.4109264 1.8555487 4.0933253
C -0.2492832 0.6232252 4.3896842
C -1.2526289 0.0983128 3.4278809
C -1.5231815 0.8434784 2.2387112
N -1.8044082 -1.0438741 3.8046164
N -0.0819482 -0.1428928 5.4555294
C 0.9903825 -0.0858301 6.3374927
C -2.5089309 -1.9163667 2.9840142
C -3.5325469 -2.7312429 3.5436777
C -4.2510473 -3.5860152 2.6972718
C -3.9381076 -3.7028873 1.3509674
C -2.8836493 -2.9605970 0.8262426
C -2.1808293 -2.0758245 1.6299851
C 2.2791246 0.2571099 5.9038543
C 3.3426399 0.2966459 6.7927757
C 3.1458046 0.0034684 8.1394658
C 1.8855039 -0.3752346 8.5780205
C 0.8003914 -0.4643125 7.6960400
C -3.8882666 -2.7509740 4.9401772
C -0.4423770 -0.9480522 8.2424026
N -1.4879455 -1.2935662 7.5679660
N -3.2253543 -2.2026017 5.9031188
C -3.6264055 -2.2672106 7.2595136
C -2.6782088 -1.7710116 8.1681333
C -2.9539302 -1.7716601 9.5361189
C -4.1635288 -2.2713058 9.9865455
C -5.1074591 -2.7653157 9.0820205
C -4.8462550 -2.7619496 7.7228218
Ni -1.6118726 -1.2414310 5.6816500
H -0.4612668 -1.0575733 9.3225365
H -4.7820290 -3.3183300 5.1818218
H -1.0967926 2.5982708 1.1392654
H 0.5723061 3.4719623 2.7401281
H 1.0818308 2.2891097 4.8196812
H -2.3059946 0.5163778 1.5709002
H -5.0543915 -4.1830931 3.1106133
H -4.4940916 -4.3849445 0.7220976
H -2.5985745 -3.0780868 -0.2110348
H -1.3352684 -1.5435102 1.2187260
H 2.4586440 0.4407051 4.8543168
H 4.3306535 0.5467320 6.4290356
H 3.9725367 0.0450612 8.8355897
H 1.7387616 -0.6285347 9.6206362
H -2.2516102 -1.3690580 10.2519935
H -4.3844802 -2.2623797 11.0454096
H -6.0568197 -3.1375571 9.4429077
H -5.6028148 -3.1227334 7.0407339

NiH$_2$PhenTAA_2eOX_Triplet:
49 atoms
C -0.8628979 2.0827638 2.0092979
C 0.0776529 2.5753303 2.9112383
C 0.3756390 1.8754098 4.0772405
C -0.2520243 0.6541637 4.3294283
C -1.2146341 0.1500780 3.4064644
C -1.5217454 0.8817890 2.2577997
N -1.8591334 -1.0012706 3.8441220
N -0.1416780 -0.1020418 5.4907008
C 0.9417080 -0.1106370 6.3115409
C -2.4779942 -1.9002531 3.0339212
C -3.4876356 -2.7657465 3.5791882
C -4.1598613 -3.6408102 2.7233200
C -3.8295157 -3.7386376 1.3763322
C -2.7952316 -2.9540375 0.8604695
C -2.1341916 -2.0503475 1.6675281
C 2.2381088 0.2378048 5.8579585
C 3.3173632 0.2439694 6.7182879
C 3.1504897 -0.0876800 8.0650200
C 1.8971547 -0.4727554 8.5275824
C 0.7898521 -0.5280010 7.6784950
C -3.8689736 -2.7826679 4.9659771
C -0.4480320 -0.9929477 8.2444520
N -1.5230749 -1.2669862 7.5823521
N -3.2531949 -2.1726833 5.9240628
C -3.6606890 -2.2395027 7.2809427
C -2.7132487 -1.7433962 8.1890612
C -2.9883496 -1.7379489 9.5544137
C -4.2017758 -2.2364888 10.0053813
C -5.1437797 -2.7298438 9.1024948
C -4.8810021 -2.7290912 7.7404107
Ni -1.6921848 -1.1425211 5.7117980
H -0.4411154 -1.1554500 9.3182178
H -4.7299049 -3.3987669 5.2081901
H -1.1147225 2.6543193 1.1259683
H 0.5547409 3.5286377 2.7269050
H 1.0442803 2.3081130 4.8066498
H -2.3069368 0.5532275 1.5930065
H -4.9468739 -4.2683983 3.1226572
H -4.3549738 -4.4377885 0.7399268
H -2.4971838 -3.0632382 -0.1740395
H -1.3025311 -1.4892770 1.2679329
H 2.3923393 0.4450397 4.8094494
H 4.3000745 0.4930760 6.3398763
H 3.9947984 -0.0707799 8.7408794
H 1.7788188 -0.7511184 9.5674209
H -2.2865240 -1.3293026 10.2677339
H -4.4237072 -2.2239175 11.0639488
H -6.0946497 -3.0990010 9.4624162
H -5.6380493 -3.0843874 7.0555778

NiH$_2$PhenTAA_2eOX_Quintet:
49 atoms
C -0.6663116 2.0022728 1.8505714
C 0.2725167 2.4939378 2.7510122
C 0.5224003 1.8216131 3.9438388
C -0.1337720 0.6218503 4.2392229
C -1.1098124 0.1106532 3.3032260
C -1.3661137 0.8326310 2.1325222
N -1.8383640 -0.9895744 3.7318746
N -0.0477311 -0.0514691 5.4492392
C 0.9748478 -0.0194535 6.3397762
C -2.5431444 -1.8607530 2.9675709
C -3.5941635 -2.6694947 3.5616928
C -4.3385383 -3.4963504 2.7154603
C -4.0603137 -3.6211499 1.3579452
C -2.9873139 -2.9183764 0.8072840
C -2.2486550 -2.0586591 1.5922820
C 2.2977451 0.3194371 5.9492700
C 3.3301357 0.3851190 6.8607597
C 3.0902916 0.1185805 8.2098058
C 1.8191772 -0.2746884 8.6165998
C 0.7494336 -0.3959223 7.7250927
C -3.9638236 -2.7490500 4.9655310
C -0.4725992 -0.9208266 8.3123051
N -1.5419307 -1.2645804 7.6757939
N -3.3367914 -2.2057666 5.9546969
C -3.6672793 -2.3323734 7.3245301
C -2.7079478 -1.8289825 8.2445111
C -2.9670863 -1.8962238 9.6128919
C -4.1487815 -2.4576356 10.0706489
C -5.0901004 -2.9518861 9.1678922
C -4.8536615 -2.8865587 7.8037306
Ni -1.6832093 -1.1464511 5.7046570
H -0.4214394 -1.0720752 9.3887098
H -4.8286640 -3.3789828 5.1642597
H -0.8855883 2.5481400 0.9426355
H 0.7862812 3.4237365 2.5461854
H 1.1850783 2.2655149 4.6719035
H -2.1557860 0.5160484 1.4673159
H -5.1506193 -4.0770443 3.1351388
H -4.6547427 -4.2845011 0.7441597
H -2.7237858 -3.0553469 -0.2332559
H -1.3883247 -1.5613060 1.1692928
H 2.5052963 0.4751032 4.9008449
H 4.3285577 0.6313880 6.5239535
H 3.8918487 0.1846924 8.9331717
H 1.6584341 -0.5144182 9.6604109
H -2.2622379 -1.4957285 10.3283724
H -4.3447380 -2.4997684 11.1335764
H -6.0160244 -3.3773447 9.5307625
H -5.6076493 -3.2519763 7.1203270

NiH$_2$PhenTAA_3eOX_Doublet:
49 atoms
C -0.8576076 2.0412345 2.0096136
C 0.0989193 2.5411760 2.9262211
C 0.3944725 1.8628901 4.0888443
C -0.2518902 0.6395451 4.3631595
C -1.2380272 0.1243892 3.4186147
C -1.5250649 0.8596850 2.2495456
N -1.8424977 -1.0224048 3.8218776
N -0.1103475 -0.1170934 5.4814689
C 0.9597195 -0.1091217 6.3124282
C -2.4794494 -1.9069795 3.0172746
C -3.4896647 -2.7755366 3.5773664
C -4.1800328 -3.6269053 2.7188005
C -3.8605660 -3.7110796 1.3614510
C -2.8209247 -2.9333825 0.8323401

C -2.1473644 -2.0454647 1.6383571
C 2.2604788 0.2596848 5.8619539
C 3.3298021 0.2848437 6.7266569
C 3.1471682 -0.0437682 8.0773293
C 1.8923755 -0.4497496 8.5380357
C 0.7960623 -0.5343695 7.6839083
C -3.8712887 -2.7928874 4.9663168
C -0.4436174 -1.0002273 8.2506803
N -1.5179737 -1.2831639 7.5887337
N -3.2512737 -2.1899926 5.9278663
C -3.6601064 -2.2451927 7.2776442
C -2.7075970 -1.7467696 8.1903566
C -2.9828415 -1.7408256 9.5579161
C -4.1985984 -2.2358701 10.0057084
C -5.1441342 -2.7309067 9.0996540
C -4.8825573 -2.7352645 7.7375939
Ni -1.6762765 -1.1733217 5.7115317
H -0.4382707 -1.1502947 9.3259389
H -4.7393815 -3.3998617 5.2045431
H -1.1072077 2.6283626 1.1344133
H 0.5624281 3.5010701 2.7344088
H 1.0545681 2.3060840 4.8203815
H -2.3185359 0.5431329 1.5880154
H -4.9735793 -4.2513506 3.1102656
H -4.3987036 -4.4032332 0.7257816
H -2.5324533 -3.0484400 -0.2046772
H -1.3018479 -1.5019087 1.2415073
H 2.4221961 0.4450757 4.8096625
H 4.3166444 0.5356505 6.3590902
H 3.9859795 -0.0143036 8.7616495
H 1.7785624 -0.7180833 9.5810961
H -2.2833424 -1.3353666 10.2753797
H -4.4237212 -2.2224250 11.0641203
H -6.0955383 -3.0976983 9.4621275
H -5.6420928 -3.0935804 7.0570465

$NiH_2PhenTAA\_3eOX\_Quartet$:
49 atoms
C -0.8448570 2.0738002 1.9946200
C 0.0966973 2.5666711 2.8974219
C 0.3884682 1.8708187 4.0709731
C -0.2430613 0.6507833 4.3269435
C -1.2102066 0.1445569 3.3997487
C -1.5130345 0.8754889 2.2477917
N -1.8504952 -1.0041066 3.8360781
N -0.1325090 -0.1049636 5.4829796
C 0.9441084 -0.1015186 6.3192371
C -2.4891469 -1.8979078 3.0288080
C -3.5176817 -2.7537358 3.5785407
C -4.2184089 -3.6222941 2.7143324
C -3.8939749 -3.7268232 1.3703692
C -2.8416842 -2.9614281 0.8620470
C -2.1528069 -2.0635966 1.6717716
C 2.2391628 0.2342627 5.8807623
C 3.3154496 0.2596699 6.7624090
C 3.1442809 -0.0448820 8.1150195
C 1.8908874 -0.4262535 8.5688903
C 0.7819816 -0.5042167 7.6991179
C -3.8780199 -2.7952766 4.9452276
C -0.4247505 -0.9884323 8.2546961
N -1.5152009 -1.2851798 7.5770113
N -3.2393109 -2.1875990 5.9246001
C -3.6518186 -2.2587216 7.2473465
C -2.6739099 -1.7468016 8.1845967
C -2.9550962 -1.7420440 9.5545164
C -4.1630985 -2.2435419 9.9906594
C -5.1242446 -2.7467238 9.0695093
C -4.8763974 -2.7478240 7.7132021
Ni -1.6687193 -1.1652192 5.6995717
H -0.4174769 -1.1719270 9.3244850
H -4.7252546 -3.4257007 5.1961376
H -1.0916187 2.6432775 1.1078523
H 0.5784134 3.5175074 2.7091691
H 1.0545368 2.3105456 4.7992000
H -2.3008944 0.5542290 1.5818606
H -5.0164859 -4.2344788 3.1167548
H -4.4332107 -4.4124203 0.7301128
H -2.5470964 -3.0703735 -0.1745769
H -1.3111215 -1.5249932 1.2610464
H 2.4147984 0.4244911 4.8318756
H 4.2996308 0.5113798 6.3865423
H 3.9842037 -0.0091207 8.7961966
H 1.7614355 -0.6887453 9.6119663
H -2.2608609 -1.3326722 10.2741831
H -4.3967308 -2.2422525 11.0480564
H -6.0697692 -3.1180893 9.4446576
H -5.6401010 -3.1016180 7.0356815

$NiH_2PhenTAA\_3eOX\_Sextet$:
49 atoms
C -0.6807838 2.0135074 1.8585657
C 0.2589445 2.5055864 2.7598920
C 0.5135408 1.8298335 3.9533641
C -0.1347739 0.6231845 4.2457932
C -1.1163304 0.1091965 3.3045387
C -1.3791476 0.8388042 2.1380157
N -1.8295742 -0.9981132 3.7242676
N -0.0361914 -0.0588027 5.4442069
C 0.9820373 -0.0159369 6.3461665
C -2.5499374 -1.8643268 2.9606949
C -3.6048080 -2.6787428 3.5614194
C -4.3668146 -3.5081829 2.7078024
C -4.0974090 -3.6313254 1.3518807
C -3.0222950 -2.9297060 0.8066058
C -2.2662356 -2.0684093 1.5939523
C 2.2994482 0.3197879 5.9693178
C 3.3330755 0.3939285 6.8963654
C 3.0971845 0.1318240 8.2458590
C 1.8290653 -0.2663883 8.6454292
C 0.7532627 -0.3977873 7.7385140
C -3.9516552 -2.7860833 4.9423811
C -0.4335317 -0.9440059 8.3148032
N -1.5229927 -1.3002000 7.6663861
N -3.3102392 -2.2367330 5.9529072
C -3.6570721 -2.3524213 7.2860545
C -2.6637167 -1.8316736 8.2384954
C -2.9423261 -1.8742317 9.6100778
C -4.1299708 -2.4155809 10.0501720
C -5.0909669 -2.9195995 9.1287490
C -4.8600248 -2.8798384 7.7714560
Ni -1.6404367 -1.1890744 5.6832566
H -0.3796747 -1.1258978 9.3854440
H -4.7962006 -3.4380730 5.1519492
H -0.9030639 2.5627407 0.9527386
H 0.7690410 3.4383589 2.5565578
H 1.1739239 2.2793669 4.6807857
H -2.1711538 0.5278812 1.4721286
H -5.1797410 -4.0855106 3.1314677
H -4.6965564 -4.2883452 0.7352592
H -2.7645193 -3.0576343 -0.2375677
H -1.4060842 -1.5804909 1.1582609
H 2.5225829 0.4742214 4.9233096
H 4.3298340 0.6515447 6.5598884
H 3.8977370 0.2064742 8.9700292
H 1.6631663 -0.5049223 9.6891018
H -2.2455838 -1.4710432 10.3313866
H -4.3468623 -2.4511993 11.1105725
H -6.0197909 -3.3286456 9.5065318
H -5.6213793 -3.2413146 7.0947657

$NiMe_2PhenTAA$:
$NiMe_2PhenTAA\_2eRED\_CSS$:
55 atoms
C -0.3767841 2.2199635 2.2002663
C 0.6101845 2.5014064 3.1453173
C 0.7575845 1.6797521 4.2593072
C -0.0625001 0.5564342 4.4610467
C -1.0820629 0.2658077 3.4848187
C -1.2100864 1.1186968 2.3752689
N -1.9250987 -0.7704100 3.8275599
N -0.0944248 -0.2487265 5.5802776
C 0.9579352 -0.5003075 6.4112139
C -2.6511554 -1.5286146 2.9562389
C -3.7754607 -2.3338689 3.4286841
C -4.5265169 -3.0153437 2.4221052
C -4.1846922 -3.0331735 1.0844861
C -3.0267640 -2.3648909 0.6655223
C -2.2901290 -1.6474757 1.5942052
C 2.2968154 -0.3399018 5.9854759
C 3.3924091 -0.5350718 6.8109148
C 3.1829196 -0.9337595 8.1376030
C 1.8962388 -1.1859136 8.5703945
C 0.7362734 -1.0490984 7.7473833
C -4.1411610 -2.5003686 4.7960054
C -0.5261894 -1.4717802 8.2557564
N -1.6798463 -1.3008840 7.5641456
N -3.5253596 -1.8258066 5.7981191
C -3.9728131 -1.6966654 7.0785539
C -2.9432413 -1.4037600 8.0637297
C -3.3457182 -1.1346332 9.3914147
C -4.6759202 -1.1551442 9.7682406
C -5.6742644 -1.4394114 8.8130220
C -5.3213423 -1.6970208 7.5011589
Ni -1.7759496 -1.1146001 5.6841259

C -0.5484518 -2.3063580
9.5187680
C -5.1147388 -3.6047347
5.1493027
H -0.5153557 2.8662096
1.3388013
H 1.2515412 3.3700039
3.0306056
H 1.4907039 1.9325022
5.0133554
H -2.0056896 0.9355337
1.6658020
H -5.4299116 -3.5335776
2.7155535
H -4.8108143 -3.5626895
0.3723032
H -2.6980870 -2.4106157 -
0.3682304
H -1.3828979 -1.1546725
1.2726134
H 2.4633894 -0.0577008
4.9549554
H 4.3958452 -0.3879089
6.4233277
H 4.0172715 -1.0469128
8.8236641
H 1.7638517 -1.4850273
9.6017979
H -2.5952869 -0.8409874
10.1131570
H -4.9506447 -0.9059620
10.7883609
H -6.7225988 -1.4105893
9.0929838
H -6.0982784 -1.8382129
6.7617992
H -1.4719293 -2.8841481
9.5648794
H 0.2927477 -3.0070917
9.5288630
H -0.4868813 -1.7285884
10.4531280
H -4.9933353 -3.8849234
6.1959936
H -6.1748835 -3.3455191
5.0095595
H -4.9264455 -4.4914590
4.5352937

NiMe₂PhenTAA_2eRED_Triplet:
55 atoms
C -0.4004665 2.2377744
2.2242493
C 0.5820724 2.5180586
3.1653087
C 0.7438689 1.6806038
4.2714158
C -0.0626575 0.5493215
4.4617764
C -1.0807403 0.2590844
3.4868422
C -1.2216760 1.1199871
2.3889512
N -1.9058904 -0.8007608
3.8243324
N -0.0828230 -0.2813120
5.5698270
C 0.9697046 -0.5142815
6.4129223
C -2.6488538 -1.5453391
2.9487928
C -3.7576496 -2.3700662
3.4258977
C -4.5004543 -3.0700306
2.4282431
C -4.1850833 -3.0604316
1.0799907
C -3.0672373 -2.3450514
0.6494647
C -2.3269353 -1.6190558
1.5769274
C 2.3051527 -0.2983523
6.0116167
C 3.4019942 -0.5003561
6.8431238
C 3.1948624 -0.9568304
8.1452300
C 1.9058480 -1.2450011
8.5608940
C 0.7491948 -1.0868028
7.7398608
C -4.1286842 -2.5274020
4.7969040
C -0.5193302 -1.5009183
8.2510177
N -1.6703957 -1.3724782
7.5373979
N -3.4796871 -1.8865176
5.8062242
C -3.9664374 -1.6925160
7.0809155
C -2.9466162 -1.4026803
8.0566324
C -3.3354979 -1.0932099
9.3685518
C -4.6870607 -1.0883208
9.7432806
C -5.6691269 -1.3675840
8.8037876
C -5.3113219 -1.6550012
7.4784276
Ni -1.7499675 -1.1553888
5.6689750
C -0.5446511 -2.3136336
9.5297529
C -5.1238213 -3.6148238
5.1484767
H -0.5456721 2.8872623
1.3662220
H 1.2191202 3.3906819
3.0565152
H 1.4780787 1.9338041
5.0234978
H -2.0153352 0.9373872
1.6779639
H -5.3735524 -3.6308682
2.7325054
H -4.8032765 -3.6087663
0.3753390
H -2.7639746 -2.3554637 -
0.3930735
H -1.4426056 -1.0975976
1.2396289
H 2.4786304 0.0210219
4.9939614
H 4.4037681 -0.3108597
6.4696106
H 4.0273458 -1.0911935
8.8295431
H 1.7751954 -1.5947359
9.5757912
H -2.5828342 -0.8080050
10.0905772
H -4.9579903 -0.8396358
10.7648972
H -6.7187034 -1.3403913
9.0805452
H -6.0863160 -1.8042243
6.7393399
H -1.4979919 -2.8274360
9.6345553
H 0.2455749 -3.0720002
9.5114543
H -0.3909080 -1.7203631
10.4419511
H -5.0772936 -3.8432336
6.2112407
H -6.1671373 -3.3615509
4.9137259
H -4.8917551 -4.5325162
4.5971973

NiMe₂PhenTAA_2eRED_Quintet:
55 atoms
C -0.3179993 2.1427430
2.1811613
C 0.6259699 2.4499275
3.1492210
C 0.7771018 1.6050126
4.2525396
C 0.0146320 0.4422979
4.4154849
C -0.9765979 0.1167674
3.4023117
C -1.1055742 0.9975780
2.3131639
N -1.7899620 -0.9589053
3.6893894
N 0.0057909 -0.3695908
5.5453036
C 1.0291789 -0.5029826
6.4639844
C -2.6671475 -1.5819975
2.8387699
C -3.8182839 -2.3317131
3.3631259
C -4.6641841 -2.9328039
2.3789982
C -4.4220475 -2.9060867
1.0148472
C -3.2793541 -2.2677474
0.5396640
C -2.4399832 -1.6257721
1.4480817
C 2.3626167 -0.2394157
6.1032391
C 3.4458988 -0.3563063
6.9753450
C 3.2094083 -0.7515318
8.2871930
C 1.9186306 -1.0790171
8.6736736
C 0.7802775 -1.0352350
7.8098344
C -4.1674021 -2.5318209
4.7440668
C -0.4713012 -1.5154400
8.3315627
N -1.6178244 -1.5323214
7.5982966
N -3.4881635 -1.9652582
5.7893860
C -3.9389433 -1.7996902
7.0797496
C -2.9063747 -1.5453486
8.0755834
C -3.3188240 -1.2220796
9.3812288
C -4.6703847 -1.1903592
9.7482730
C -5.6504123 -1.4489953
8.8027201
C -5.2825556 -1.7350954
7.4810036
Ni -1.6376966 -1.3598352
5.6162344
C -0.4523343 -2.2352593
9.6680720
C -5.2022883 -3.5950728
5.0630234
H -0.4621902 2.7928494
1.3228168
H 1.2380357 3.3430794
3.0673797
H 1.4720840 1.8819977
5.0331066
H -1.8771497 0.8100000
1.5806449
H -5.5662070 -3.4288579
2.7080733
H -5.1206698 -3.3838453
0.3340275
H -3.0320391 -2.2707794 -
0.5176112
H -1.5324852 -1.1696240
1.0786431
H 2.5543672 0.0460698
5.0788087
H 4.4497872 -0.1391133
6.6239150
H 4.0191539 -0.8120268
9.0087382
H 1.7749108 -1.3805031
9.7011620
H -2.5745909 -0.9377005
10.1112537
H -4.9415467 -0.9318407
10.7676871
H -6.7021836 -1.4012594
9.0686187
H -6.0560900 -1.8490751
6.7341937
H -1.3746919 -2.7962699
9.8044196
H 0.3795094 -2.9476752
9.7032469
H -0.3362969 -1.5746099
10.5392739
H -5.1352180 -3.8794457
6.1115990
H -6.2411354 -3.2871858
4.8763427
H -5.0272203 -4.4908293
4.4581277

NiMe₂PhenTAA_1eRED_Doublet:
55 atoms
C -0.4000137 2.2584216
2.2154286
C 0.5850608 2.5391101
3.1584885
C 0.7350186 1.7191939
4.2715652
C -0.0791454 0.5941417
4.4584459
C -1.0903452 0.3060253
3.4903131
C -1.2331216 1.1583904
2.3873732
N -1.9375841 -0.7379392
3.8425621
N -0.1180627 -0.2194915
5.5846059
C 0.9310980 -0.5081559
6.3931773
C -2.6317953 -1.5229860
2.9825532
C -3.7101692 -2.3797476
3.4457091
C -4.4214617 -3.1145011
2.4586508
C -4.0984093 -3.1084675
1.1206621
C -2.9863403 -2.3664715
0.6994222
C -2.2831727 -1.6080426
1.6067011
C 2.2735730 -0.3100160
5.9687344
C 3.3603535 -0.5582001
6.7750597
C 3.1696612 -1.0375754
8.0783622
C 1.8893925 -1.3166534
8.4999078
C 0.7329428 -1.1143855
7.6987644
C -4.1031016 -2.5246302
4.8226908
C -0.5449660 -1.5117494
8.2282216
N -1.6807575 -1.3052143
7.5572318
N -3.5173315 -1.8279993
5.7996020
C -3.9775133 -1.6924466
7.0979982
C -2.9631471 -1.4036665
8.0686799
C -3.3522819 -1.1004538
9.3835509
C -4.6869478 -1.1019541
9.7554464
C -5.6772902 -1.3840630
8.8078189
C -5.3235493 -1.6618656
7.4973274
Ni -1.7941803 -1.0595069
5.6867577

C -0.5757601 -2.3075471 9.5169905
C -5.1120995 -3.5982378 5.1756548
H -0.5424509 2.9092930 1.3602957
H 1.2191998 3.4112093 3.0467510
H 1.4590631 1.9799185 5.0307431
H -2.0360325 0.9840185 1.6849302
H -5.2744043 -3.7033579 2.7630192
H -4.6850349 -3.6835377 0.4143461
H -2.6661204 -2.3945399 -0.3364618
H -1.4076646 -1.0711749 1.2734075
H 2.4387989 0.0245328 4.9555214
H 4.3608077 -0.3922346 6.3904322
H 4.0113816 -1.2052155 8.7395143
H 1.7666040 -1.6974164 9.5033252
H -2.6018374 -0.8009382 10.1011275
H -4.9611715 -0.8402689 10.7705761
H -6.7244281 -1.3426166 9.0834032
H -6.0981031 -1.7967487 6.7559616
H -1.5267837 -2.8244065 9.6221779
H 0.2147867 -3.0597729 9.5105078
H -0.4301718 -1.6941762 10.4126753
H -5.0645756 -3.8305719 6.2369844
H -6.1454848 -3.3200307 4.9423812
H -4.8929313 -4.5132799 4.6229230

NiMe$_2$PhenTAA_1eRED_Quartet:
55 atoms
C -0.5108866 2.2397878 2.3258549
C 0.4846676 2.5235075 3.2790421
C 0.6971104 1.6772703 4.3471447
C -0.0805766 0.5059105 4.5075297
C -1.1122304 0.2119821 3.5198239
C -1.2934705 1.1100445 2.4413761
N -1.8372359 -0.8939734 3.7998102
N -0.0326861 -0.3798315 5.5274757
C 1.0213337 -0.5566935 6.4151792
C -2.6391060 -1.5993801 2.9111319
C -3.7520165 -2.3825759 3.4045250
C -4.5146952 -3.0611350 2.4136891
C -4.1967343 -3.0508858 1.0670655
C -3.0673928 -2.3674229 0.6245173
C -2.3060168 -1.6647214 1.5543602
C 2.3399339 -0.3412943 6.0017550
C 3.4325236 -0.5157622 6.8466622
C 3.2047649 -0.9423302 8.1522537
C 1.9174140 -1.2288646 8.5709059
C 0.7732851 -1.0936382 7.7363590
C -4.1073856 -2.5399164 4.7879609
C -0.5073231 -1.5147729 8.2338954
N -1.6448531 -1.3559544 7.5168434
N -3.4639395 -1.8739052 5.7757816
C -3.9450435 -1.6625689 7.0571374
C -2.9325743 -1.3741828 8.0261229
C -3.3159216 -1.0357414 9.3288047
C -4.6644224 -1.0065160 9.6947868
C -5.6465167 -1.2864113 8.7549550
C -5.2895224 -1.5981263 7.4401721
Ni -1.6858519 -1.2667421 5.6353718
C -0.5431472 -2.3251989 9.5112538
C -5.1011807 -3.6224972 5.1488099
H -0.6839081 2.9286971 1.5072358
H 1.0729123 3.4293555 3.1893164
H 1.4287380 1.9285063 5.1012857
H -2.0887589 0.9261610 1.7337142
H -5.3968235 -3.6056863 2.7174010
H -4.8263415 -3.5870989 0.3656339
H -2.7770476 -2.3788009 -0.4189569
H -1.4058909 -1.1633853 1.2262855
H 2.5102108 -0.0479277 4.9750417
H 4.4355230 -0.3240771 6.4853865
H 4.0308064 -1.0636701 8.8442537
H 1.7827105 -1.5604994 9.5901126
H -2.5612564 -0.7538930 10.0493100
H -4.9369407 -0.7390836 10.7093305
H -6.6947742 -1.2401219 9.0271668
H -6.0617504 -1.7514585 6.6997616
H -1.4986807 -2.8346275 9.6119822
H 0.2439957 -3.0847440 9.5000959
H -0.3936098 -1.7251263 10.4170757
H -5.0513551 -3.8456402 6.2120297
H -6.1405603 -3.3604012 4.9163931
H -4.8755023 -4.5419374 4.6007871

NiMe$_2$PhenTAA_1eRED_Sextet:
55 atoms
C -0.5499170 2.1852367 2.4193094
C 0.4672500 2.4484824 3.3552731
C 0.7397925 1.5484883 4.3599387
C 0.0209771 0.3287937 4.4771288
C -1.0523564 0.0609786 3.5040216
C -1.2908103 1.0261285 2.4940064
N -1.7750312 -1.0559316 3.7267108
N 0.1599006 -0.5858514 5.4532203
C 1.1589488 -0.6088532 6.4249778
C -2.6801599 -1.6610161 2.8482748
C -3.8162145 -2.4199296 3.3537055
C -4.6526040 -2.9854537 2.3436519
C -4.3875175 -2.9110992 0.9878353
C -3.2439418 -2.2628325 0.5352318
C -2.4161849 -1.6529185 1.4772394
C 2.4807698 -0.3262191 6.0755854
C 3.5356439 -0.3829594 6.9850601
C 3.2495951 -0.7307726 8.3020360
C 1.9582967 -1.0566586 8.6736187
C 0.8492658 -1.0593438 7.7727069
C -4.1708619 -2.6434250 4.7302255
C -0.4252494 -1.4987049 8.2674728
N -1.5698630 -1.4702598 7.5136751
N -3.4331601 -2.1504683 5.7639478
C -3.8885527 -1.8393916 7.0281778
C -2.8700020 -1.4797156 7.9918799
C -3.2653068 -1.0509319 9.2639029
C -4.6111271 -0.9822679 9.6301900
C -5.5902153 -1.3121069 8.7052724
C -5.2322160 -1.7172801 7.4186468
Ni -1.5446063 -1.6072757 5.5861364
C -0.4597481 -2.1999028 9.6093495
C -5.2887277 -3.6225413 5.0275211
H -0.7755247 2.9145587 1.6497729
H 1.0197440 3.3794532 3.3045317
H 1.4814612 1.7858900 5.1080799
H -2.1080234 0.8647955 1.8066329
H -5.5528739 -3.4995462 2.6437172
H -5.0738703 -3.3729299 0.2863788
H -2.9922001 -2.2281661 -0.5175290
H -1.5066181 -1.1735693 1.1413657
H 2.6886708 -0.0937148 5.0396415
H 4.5471413 -0.1658593 6.6652666
H 4.0381073 -0.7587727 9.0462709
H 1.7881215 -1.3115718 9.7087088
H -2.5127120 -0.7094874 9.9600793
H -4.8813110 -0.6369691 10.6215179
H -6.6399503 -1.2264056 8.9632447
H -6.0120168 -1.8897955 6.6924958
H -1.3895562 -2.7530389 9.7215168
H 0.3664337 -2.9123933 9.6865758
H -0.3765122 -1.5224705 10.4682830
H -5.2718429 -3.9054044 6.0773577
H -6.2922459 -3.2408767 4.8009292
H -5.1584888 -4.5357229 4.4382308

NiMe$_2$PhenTAA_0eNeutral_CSS:
55 atoms
C -0.4835905 2.3340622 2.2826093
C 0.4988199 2.6144961 3.2237267
C 0.6719562 1.7782597 4.3223538
C -0.1162158 0.6368555 4.4768631
C -1.1196967 0.3504642 3.5156471
C -1.2978019 1.2160428 2.4354175
N -1.9428103 -0.7307888 3.8648327
N -0.1415910 -0.2168759 5.5899956
C 0.9077993 -0.5386847 6.3718166
C -2.6021673 -1.5389232 3.0114491
C -3.6160834 -2.4695209 3.4690538
C -4.2635902 -3.2743434 2.4941356
C -3.9530181 -3.2401284 1.1596850
C -2.9217578 -2.3882422 0.7326491
C -2.2804287 -1.5680034 1.6214673
C 2.2478570 -0.2771515 5.9568197
C 3.3345852 -0.6059205 6.7217070
C 3.1697504 -1.2123437 7.9773910
C 1.9009099 -1.5197304 8.3944579
C 0.7360036 -1.2302267 7.6349937
C -4.0549618 -2.5731499 4.8409347
C -0.5487970 -1.5746121 8.1970903
N -1.6666895 -1.3524824 7.5400311
N -3.4869485 -1.8711642 5.7976225
C -3.9755809 -1.6936028 7.1102961
C -2.9747641 -1.4082621 8.0682333
C -3.3487529 -1.0355335 9.3576522
C -4.6922750 -0.9823292 9.7161734
C -5.6740690 -1.2624649 8.7765642
C -5.3172156 -1.5970406 7.4738086
Ni -1.8026430 -1.0376714 5.6892520

C -0.5884558 -2.2767393 9.5380253
C -5.1400349 -3.5729278 5.1811876
H -0.6449061 3.0013185 1.4452919
H 1.1108015 3.5024739 3.1271981
H 1.3874768 2.0445155 5.0868926
H -2.1068562 1.0471781 1.7395466
H -5.0459616 -3.9488672 2.8049372
H -4.4763313 -3.8747670 0.4572940
H -2.6119389 -2.3885128 -0.3059032
H -1.4604869 -0.9592763 1.2750420
H 2.4051535 0.1438961 4.9766285
H 4.3296350 -0.4105137 6.3392277
H 4.0247504 -1.4550925 8.5938397
H 1.7965023 -2.0021062 9.3537091
H -2.5974762 -0.7283002 10.0694460
H -4.9657548 -0.6790146 10.7185139
H -6.7209051 -1.1798944 9.0388355
H -6.0882831 -1.7241281 6.7290111
H -1.5636574 -2.7147527 9.7240016
H 0.1402192 -3.0857897 9.5451105
H -0.3438663 -1.6077823 10.3658966
H -5.1913853 -3.7486722 6.2507196
H -6.1247603 -3.2534824 4.8333765
H -4.9177113 -4.5257463 4.7034417

$NiMe_2PhenTAA$_0eNeutral_Triplet:
55 atoms
C -0.2664428 2.2626658 2.1091034
C 0.7278498 2.5101424 3.0454015
C 0.8288838 1.7092452 4.1782442
C -0.0351564 0.6304425 4.3797128
C -1.0621941 0.3792778 3.4199079
C -1.1621602 1.2170939 2.3066474
N -2.0077542 -0.5889565 3.7904024
N -0.1192335 -0.1301449 5.5632460
C 0.9091359 -0.4612777 6.3841860
C -2.6701384 -1.4263101 2.9772756
C -3.7161310 -2.3174615 3.4704895
C -4.4144302 -3.0969648 2.5051685
C -4.1115545 -3.1070352 1.1700370
C -3.0149671 -2.3481711 0.7248215
C -2.3259766 -1.5489684 1.5934273
C 2.2602480 -0.3291175 5.9561325
C 3.3290943 -0.6023782 6.7710725
C 3.1202637 -1.0397653 8.0845572
C 1.8325952 -1.2754778 8.5016352
C 0.6900858 -1.0497656 7.6931503
C -4.0957367 -2.4973092 4.8549224
C -0.5971534 -1.4630742 8.2438051
N -1.7262292 -1.1937922 7.6391857
N -3.5891792 -1.7631345 5.8264703
C -4.0211548 -1.7040163 7.1609802
C -3.0261656 -1.4038673 8.1330633
C -3.4157435 -1.1451341 9.4493154
C -4.7502097 -1.2111457 9.8261971
C -5.7233729 -1.5042507 8.8775901
C -5.3621217 -1.7295562 7.5564114
Ni -1.8739421 -0.8150295 5.7079256
C -0.5932423 -2.3221116 9.4942486
C -5.0255549 -3.6460139 5.2022368
H -0.3688014 2.8970475 1.2375353
H 1.4099463 3.3409675 2.9153886
H 1.5591985 1.9548784 4.9357847
H -1.9784181 1.0775194 1.6121291
H -5.2407602 -3.7100694 2.8295433
H -4.6871149 -3.7098101 0.4805129
H -2.7030602 -2.3999589 -0.3119301
H -1.4685253 -1.0009525 1.2356997
H 2.4412178 -0.0214719 4.9381781
H 4.3356826 -0.4823991 6.3881133
H 3.9531902 -1.2200443 8.7508701
H 1.6972917 -1.6449976 9.5062465
H -2.6720482 -0.8422980 10.1721552
H -5.0295915 -1.0005426 10.8503607
H -6.7691204 -1.5249543 9.1565783
H -6.1310075 -1.8806140 6.8128463
H -1.5470633 -2.8278746 9.6133472
H 0.1848453 -3.0802455 9.4261043
H -0.4071057 -1.7378773 10.3983985
H -4.9821042 -3.8660127 6.2650321
H -6.0654380 -3.4315670 4.9452281
H -4.7314247 -4.5433608 4.6609070

$NiMe_2PhenTAA$_0eNeutral_Quintet:
55 atoms
C -0.4061163 2.1674066 2.2506104
C 0.6023057 2.4436195 3.1896448
C 0.8073955 1.6011460 4.2550147
C 0.0270804 0.4273947 4.4243184
C -1.0267457 0.1482819 3.4480548
C -1.2074380 1.0519768 2.3843070
N -1.8314232 -0.9220743 3.7376063
N 0.0807575 -0.4222606 5.4735089
C 1.0852754 -0.5225762 6.4268238
C -2.6664954 -1.5967640 2.8816267
C -3.7800390 -2.3743879 3.3700013
C -4.5745722 -3.0250686 2.4013435
C -4.3057747 -2.9984644 1.0478006
C -3.1726024 -2.3241878 0.5988638
C -2.3809701 -1.6442161 1.4987440
C 2.4144333 -0.2566381 6.0751393
C 3.4673708 -0.3592654 6.9750976
C 3.1817806 -0.7328108 8.2831574
C 1.8878286 -1.0604606 8.6481998
C 0.7825809 -1.0351363 7.7532014
C -4.1858845 -2.5327069 4.7823943
C -0.4800994 -1.5265042 8.2472398
N -1.6459096 -1.4646317 7.5186584
N -3.5909029 -1.8789836 5.7392939
C -3.9847803 -1.7580083 7.0841847
C -2.9253641 -1.5034007 8.0221073
C -3.2923821 -1.1975597 9.3485766
C -4.6191875 -1.1616007 9.7409960
C -5.6400350 -1.4012990 8.8233465
C -5.3141327 -1.6818434 7.5033679
Ni -1.6760938 -1.2863891 5.6169970
C -0.4775179 -2.3269340 9.5302366
C -5.2700148 -3.5439926 5.0942444
H -0.5835193 2.8557386 1.4337507
H 1.1937828 3.3454740 3.0960715
H 1.5308128 1.8594397 5.0132526
H -2.0221091 0.8963561 1.6927085
H -5.4483488 -3.5714686 2.7189005
H -4.9563566 -3.5158869 0.3551960
H -2.9027608 -2.3367511 -0.4500722
H -1.4827203 -1.1586811 1.1475416
H 2.6282004 -0.0101311 5.0449884
H 4.4809609 -0.1564375 6.6558355
H 3.9712524 -0.7899551 9.0227807
H 1.7207030 -1.3475074 9.6745711
H -2.5260332 -0.9298169 10.0592588
H -4.8615731 -0.9057884 10.7653468
H -6.6778137 -1.3309633 9.1210815
H -6.1067391 -1.7820695 6.7763933
H -1.3847265 -2.9220876 9.6081274
H 0.3702333 -3.0153181 9.5445638
H -0.4069202 -1.7166779 10.4378146
H -5.2575405 -3.7931894 6.1515748
H -6.2703198 -3.1829910 4.8430678
H -5.0987913 -4.4589483 4.5285382

$NiMe_2PhenTAA$_1eOX_Doublet:
55 atoms
C -0.5575661 2.3218057 2.3476737
C 0.4399922 2.6064838 3.3031967
C 0.6436820 1.7753289 4.3794306
C -0.1422910 0.6148510 4.5319071
C -1.1663261 0.3227266 3.5511478
C -1.3518788 1.2059374 2.4680448
N -1.8984424 -0.7895376 3.8476338
N -0.1083317 -0.2790553 5.5618548
C 0.9555368 -0.5660580 6.3783218
C -2.6006876 -1.5794875 2.9737175
C -3.6115625 -2.4869733 3.4419756
C -4.2704001 -3.2766090 2.4772465
C -3.9588773 -3.2371271 1.1338552
C -2.9320193 -2.4009428 0.7003091
C -2.2731142 -1.5926700 1.6013359
C 2.2742055 -0.2962650 5.9549575
C 3.3692236 -0.6055193 6.7325419
C 3.1936534 -1.2005332 7.9802693
C 1.9177535 -1.5151083 8.4003025
C 0.7669566 -1.2401842 7.6331678
C -4.0535622 -2.5886162 4.8357820
C -0.5393932 -1.5879858 8.1994562
N -1.6429057 -1.3756049 7.5337877
N -3.4744960 -1.8973967 5.7806596
C -3.9592496 -1.6952383 7.0962266
C -2.9607528 -1.4106598 8.0518592
C -3.3310304 -1.0118264 9.3349322
C -4.6733126 -0.9335289 9.6832679
C -5.6563983 -1.2138974 8.7424831
C -5.3022388 -1.5738599 7.4485691
Ni -1.7597002 -1.1160602 5.6677133

C -0.5749320 -2.2560621
9.5523054
C -5.1599646 -3.5613287
5.1636901
H -0.7289155 3.0145906
1.5343401
H 1.0217361 3.5141784
3.2112589
H 1.3612224 2.0406925
5.1416083
H -2.1585071 1.0363930
1.7703733
H -5.0576172 -3.9462556
2.7834882
H -4.4920201 -3.8668967
0.4350685
H -2.6336367 -2.3936582 -
0.3402553
H -1.4437935 -0.9928648
1.2596518
H 2.4295348 0.1124907
4.9685459
H 4.3640624 -0.3992816
6.3588636
H 4.0438024 -1.4368423
8.6052019
H 1.8162940 -1.9903379
9.3625135
H -2.5807479 -0.7034012
10.0465973
H -4.9481101 -0.6095757
10.6781513
H -6.7019558 -1.1098468
8.9998304
H -6.0735543 -1.6993593
6.7043934
H -1.5493433 -2.6870480
9.7553222
H 0.1523772 -3.0662500
9.5717890
H -0.3202652 -1.5672234
10.3600712
H -5.2298811 -3.7353827
6.2319550
H -6.1309784 -3.2208537
4.7989036
H -4.9492731 -4.5182651
4.6887057

NiMe$_2$PhenTAA_1eOX_Quartet:
55 atoms
C -0.3575206 2.2096619
2.1769976
C 0.6366532 2.4921864
3.1280328
C 0.8011759 1.6841970
4.2312423
C -0.0088673 0.5468505
4.4176727
C -1.0385696 0.2543157
3.4326615
C -1.1853793 1.1197044
2.3309524
N -1.8706666 -0.7891494
3.7514373
N -0.0157443 -0.2620099
5.5259653
C 1.0029364 -0.4878959
6.4102943
C -2.6549605 -1.5287058
2.9095761
C -3.7302844 -2.3472774
3.4208994
C -4.4972350 -3.0540133
2.4712331
C -4.2277283 -3.0422658
1.1176537
C -3.1190942 -2.3383117
0.6504753
C -2.3557502 -1.6037693
1.5296516
C 2.3466753 -0.2659962
6.0302194
C 3.3995646 -0.4822892
6.8903757
C 3.1535985 -0.9389796
8.1841663
C 1.8598462 -1.2423056
8.5572282
C 0.7473757 -1.0719838
7.7069418
C -4.1076505 -2.5182580
4.8354031
C -0.5591633 -1.5075678
8.2318510
N -1.6733103 -1.2549394
7.5981305
N -3.5709747 -1.7954250
5.7820622
C -3.9906598 -1.7083551
7.1264358
C -2.9860602 -1.4221339
8.0878034
C -3.3644907 -1.1313420
9.4007836
C -4.6991903 -1.1510771
9.7760808
C -5.6833047 -1.4316667
8.8343545
C -5.3313922 -1.6919959
7.5186554
Ni -1.7761633 -1.0062854
5.6518269
C -0.5670538 -2.3399985
9.4948015
C -5.0808347 -3.6255680
5.1743524
H -0.5016212 2.8762436
1.3368312
H 1.2525220 3.3747137
3.0148779
H 1.5163846 1.9569300
4.9926889
H -1.9944764 0.9592696
1.6343221
H -5.3449331 -3.6333298
2.8000768
H -4.8555652 -3.6005876
0.4366109
H -2.8486047 -2.3715215 -
0.3971610
H -1.4729892 -1.1003642
1.1662769
H 2.5505004 0.0429301
5.0164290
H 4.4129226 -0.3042947
6.5538711
H 3.9661728 -1.0859513
8.8826274
H 1.7105900 -1.6215309
9.5553427
H -2.6155337 -0.8358113
10.1202784
H -4.9717859 -0.9128056
10.7955810
H -6.7279749 -1.4135938
9.1150736
H -6.1065256 -1.8309836
6.7799085
H -1.5181672 -2.8509111
9.6119822
H 0.2179442 -3.0929445
9.4478878
H -0.3939031 -1.7382908
10.3895237
H -5.0464084 -3.8556370
6.2351542
H -6.1101766 -3.3663021
4.9177392
H -4.8221484 -4.5285775
4.6238597

NiMe$_2$PhenTAA_1eOX_Sextet:
55 atoms
C -0.7497249 2.1446322
2.4464489
C 0.1366647 2.5223061
3.4755285
C 0.4209201 1.6644335
4.5103556
C -0.1525653 0.3726607
4.5610723
C -1.0882028 -0.0085599
3.5036323
C -1.3586289 0.9138755
2.4670499
N -1.7090276 -1.2008763
3.6796706
N 0.0098916 -0.5360320
5.5564499
C 1.0101513 -0.5303835
6.5358963
C -2.5751590 -1.8265174
2.8113283
C -3.7431322 -2.5110589
3.3011431
C -4.5533279 -3.1661421
2.3455179
C -4.2529651 -3.1875099
1.0008257
C -3.0951886 -2.5547333
0.5478471
C -2.2782803 -1.8891207
1.4387317
C 2.2842489 -0.0494515
6.2242813
C 3.3305804 -0.0525758
7.1376850
C 3.1203601 -0.5671865
8.4135722
C 1.8957212 -1.1149494
8.7341727
C 0.8036154 -1.1659856
7.8240284
C -4.1785927 -2.5255366
4.6989097
C -0.3512152 -1.8897851
8.2463384
N -1.5236612 -1.9209411
7.4530510
N -3.4525790 -1.9646361
5.6389107
C -3.8254502 -1.6428311
6.9412690
C -2.7380227 -1.5971007
7.9067587
C -3.0213470 -1.1521100
9.2308075
C -4.2902592 -0.7627265
9.5775604
C -5.3230670 -0.7732407
8.6251078
C -5.0855962 -1.1956639
7.3215099
Ni -1.4471292 -1.8181611
5.5019672
C -0.3183083 -2.8327910
9.4191689
C -5.4447945 -3.2656911
5.0554242
H -0.9808486 2.8445417
1.6543285
H 0.5729668 3.5126476
3.4681870
H 1.0543174 1.9928644
5.3200241
H -2.0868834 0.6630373
1.7103081
H -5.4513916 -3.6715662
2.6631342
H -4.9037194 -3.7048525
0.3093277
H -2.8261716 -2.5925404 -
0.5000979
H -1.3614141 -1.4338314
1.0922687
H 2.4705910 0.3045235
5.2209865
H 4.2979135 0.3361267
6.8484180
H 3.9156977 -0.5622956
9.1470804
H 1.7663079 -1.5264910
9.7239107
H -2.2175499 -1.0992771
9.9489408
H -4.4934295 -0.4209756
10.5837941
H -6.3122684 -0.4328725
8.9010085
H -5.8793712 -1.1361386
6.5914630
H -1.2209641 -3.4408419
9.4453964
H 0.5402280 -3.5060446
9.3562667
H -0.2437375 -2.3117331
10.3797964
H -5.5385712 -3.3903732
6.1294441
H -6.3324756 -2.7483774
4.6851103
H -5.4331548 -4.2571403
4.6048825

NiMe$_2$PhenTAA_2eOX_CSS:
55 atoms
C -0.6069111 2.2998098
2.3863432
C 0.4091785 2.5897418
3.3594991
C 0.6305515 1.7756217
4.4250064
C -0.1597012 0.5988598
4.5840622
C -1.2118621 0.2987836
3.5765038
C -1.3958585 1.1975178
2.4843499
N -1.8705698 -0.8249649
3.8286057
N -0.0799432 -0.3144373
5.5431291
C 0.9884715 -0.5787302
6.3844555
C -2.6029642 -1.6022711
2.9462042
C -3.6080552 -2.5020558
3.4276597
C -4.2728140 -3.2775625
2.4675703
C -3.9586009 -3.2332054
1.1159822
C -2.9351269 -2.4058635
0.6738055
C -2.2694392 -1.6025419
1.5813084
C 2.2962767 -0.3007771
5.9525026
C 3.3961136 -0.6016254
6.7347623
C 3.2097472 -1.1917833
7.9775662
C 1.9273851 -1.5124230
8.4021455
C 0.7850640 -1.2510591
7.6327569
C -4.0527464 -2.6007651
4.8347847
C -0.5348587 -1.5991958
8.2019236
N -1.6299534 -1.3890142
7.5283676
N -3.4654885 -1.9117920
5.7715294
C -3.9507220 -1.6868244
7.0858197
C -2.9530823 -1.4026019
8.0406045
C -3.3193736 -0.9915412
9.3209002
C -4.6619889 -0.9010602
9.6630061
C -5.6461493 -1.1815994
8.7211993
C -5.2945298 -1.5544420
7.4307693
Ni -1.7304361 -1.1711537
5.6535878

C -0.5690914 -2.2456643
9.5607723
C -5.1711603 -3.5555129
5.1560425
H -0.7787894 3.0129935
1.5905046
H 0.9683210 3.5115247
3.2638740
H 1.3414392 2.0480645
5.1911059
H -2.2083329 1.0353146
1.7915225
H -5.0649968 -3.9436713
2.7678176
H -4.4969338 -3.8624614
0.4202403
H -2.6422797 -2.3983313 -
0.3677462
H -1.4305597 -1.0125348
1.2434724
H 2.4507058 0.0950768
4.9598433
H 4.3918280 -0.3932713
6.3662449
H 4.0568310 -1.4268768
8.6076371
H 1.8306794 -1.9813024
9.3676919
H -2.5707840 -0.6845189
10.0346791
H -4.9379997 -0.5675419
10.6542751
H -6.6909562 -1.0673192
8.9768170
H -6.0674704 -1.6811338
6.6887487
H -1.5428486 -2.6734608
9.7726265
H 0.1581193 -3.0564205
9.5894420
H -0.3106876 -1.5450854
10.3575414
H -5.2506310 -3.7291075
6.2235430
H -6.1348980 -3.2022889
4.7833826
H -4.9691172 -4.5155146
4.6822106

$NiMe_2PhenTAA$_2eOX_Triplet:
55 atoms
C -0.5804128 2.3754480
2.3684091
C 0.4047467 2.6566352
3.3121270
C 0.6218767 1.8008598
4.3889911
C -0.1424002 0.6412224
4.5079202
C -1.1508766 0.3535033
3.5420882
C -1.3682986 1.2329472
2.4826981
N -1.9164819 -0.7666481
3.8661625
N -0.1324765 -0.2579567
5.5743668
C 0.9324971 -0.5878048
6.3434062
C -2.5610226 -1.5833920
2.9989647
C -3.5758113 -2.5020060
3.4680019
C -4.2024272 -3.2955941
2.5045342
C -3.8550871 -3.2641781
1.1567734
C -2.8285186 -2.4281912
0.7228950
C -2.1966300 -1.6044776
1.6268587
C 2.2531251 -0.3357729
5.8869483
C 3.3553265 -0.6659628
6.6426415
C 3.1793478 -1.2610004
7.8900342
C 1.8969856 -1.5592710
8.3425146
C 0.7463292 -1.2712440
7.6050965
C -4.0502008 -2.5857267
4.8628222
C -0.5660528 -1.5936773
8.1976852
N -1.6676971 -1.3471694
7.5474046
N -3.4955384 -1.8678629
5.7978290
C -3.9861354 -1.6668645
7.1174495
C -2.9890899 -1.3827688
8.0717295
C -3.3526037 -0.9907924
9.3566795
C -4.6971001 -0.9164844
9.7031199
C -5.6802005 -1.1967434
8.7622689
C -5.3289781 -1.5541044
7.4652804
Ni -1.8051656 -1.0458444
5.6943772
C -0.5898753 -2.2593372
9.5476581
C -5.1545739 -3.5587184
5.1787099
H -0.7620433 3.0668901
1.5565030
H 0.9854562 3.5656867
3.2305450
H 1.3367269 2.0666828
5.1540350
H -2.1777701 1.0637919
1.7876501
H -4.9949181 -3.9673346
2.7917858
H -4.3704536 -3.9089752
0.4577567
H -2.5134329 -2.4369940 -
0.3120964
H -1.3607341 -1.0042206
1.3016361
H 2.3925339 0.0669978
4.8954455
H 4.3493980 -0.4806847
6.2578434
H 4.0339531 -1.5160678
8.5020498
H 1.8143496 -2.0297484
9.3087862
H -2.6035517 -0.6885618
10.0723292
H -4.9732903 -0.5994017
10.6996601
H -6.7253826 -1.0989621
9.0229224
H -6.1023142 -1.6858810
6.7242527
H -1.5662516 -2.6782034
9.7656168
H 0.1276574 -3.0787003
9.5607765
H -0.3171177 -1.5697153
10.3492350
H -5.2420686 -3.7252584
6.2468009
H -6.1199540 -3.2208197
4.7960945
H -4.9353719 -4.5195423
4.7149256

$NiMe_2PhenTAA$_2eOX_Quintet:
55 atoms
C -0.3660044 2.2617567
2.1798333
C 0.6189976 2.5418395
3.1225130
C 0.7873642 1.7197752
4.2320770
C -0.0102485 0.5838441
4.3987829
C -1.0308847 0.2937735
3.4221158
C -1.1958586 1.1559281
2.3342010
N -1.8784288 -0.7566954
3.7519904
N -0.0257619 -0.2302514
5.5246305
C 0.9880644 -0.5115098
6.3705257
C -2.6101338 -1.5352308
2.9268393
C -3.6886529 -2.3706031
3.4433582
C -4.4279518 -3.0777633
2.4918896
C -4.1377459 -3.0670734
1.1290071
C -3.0338002 -2.3592022
0.6588752
C -2.2885364 -1.6143067
1.5423341
C 2.3372123 -0.2980837
5.9696511
C 3.3972728 -0.5278952
6.8148046
C 3.1497995 -0.9906566
8.1053360
C 1.8469326 -1.2898438
8.4987929
C 0.7325032 -1.1117092
7.6751923
C -4.1075536 -2.5292931
4.8632383
C -0.5826282 -1.5254533
8.2371880
N -1.7001200 -1.2551255
7.6247612
N -3.5964453 -1.7949210
5.8100128
C -4.0236331 -1.6970502
7.1566551
C -3.0181280 -1.4106226
8.1188654
C -3.3918024 -1.1208857
9.4316907
C -4.7287418 -1.1383135
9.8032877
C -5.7129609 -1.4189738
8.8614799
C -5.3639303 -1.6830687
7.5445767
Ni -1.8178972 -0.9759233
5.6863123
C -0.5752472 -2.3329691
9.5110469
C -5.0978470 -3.6214368
5.1818130
H -0.5140196 2.9249947
1.3379606
H 1.2373497 3.4229883
3.0140939
H 1.4989439 1.9956377
4.9966157
H -2.0085464 0.9984235
1.6401800
H -5.2752436 -3.6672257
2.8023601
H -4.7549298 -3.6387947
0.4489476
H -2.7555509 -2.4020692 -
0.3858224
H -1.4013023 -1.1080464
1.1927661
H 2.5268512 0.0086984
4.9519911
H 4.4104948 -0.3615211
6.4736855
H 3.9653455 -1.1532982
8.7972156
H 1.7183650 -1.6736730
9.4977379
H -2.6442455 -0.8298710
10.1544201
H -5.0028740 -0.9003696
10.8223260
H -6.7570927 -1.4006819
9.1437379
H -6.1408518 -1.8267260
6.8086616
H -1.5306404 -2.8275819
9.6581033
H 0.1966222 -3.0996087
9.4647567
H -0.3757635 -1.7144704
10.3890121
H -5.0963190 -3.8435264
6.2447134
H -6.1161222 -3.3500217
4.8943199
H -4.8356745 -4.5333127
4.6475394

$NiMe_2PhenTAA$_3eOX_Doublet:
55 atoms
C -0.5885645 2.3317677
2.3741246
C 0.4120403 2.6171500
3.3324025
C 0.6343406 1.7788040
4.4050355
C -0.1437767 0.6139307
4.5423977
C -1.1761886 0.3194847
3.5536589
C -1.3775672 1.2050938
2.4783286
N -1.8917241 -0.8040763
3.8392773
N -0.0960026 -0.2923806
5.5583872
C 0.9569025 -0.5895104
6.3501134
C -2.5671248 -1.5936047
2.9765780
C -3.5821983 -2.5159689
3.4600575
C -4.2304655 -3.2828647
2.4964266
C -3.9010351 -3.2309438
1.1377675
C -2.8732193 -2.3971595
0.6881009
C -2.2217194 -1.5951234
1.5908640
C 2.2850355 -0.3106303
5.9053435
C 3.3799193 -0.6157305
6.6740419
C 3.1881281 -1.2124330
7.9234370
C 1.9010940 -1.5373437
8.3653065
C 0.7579088 -1.2801856
7.6142941
C -4.0528011 -2.6032458
4.8599397
C -0.5583520 -1.6080676
8.2047275
N -1.6568489 -1.3674465
7.5434031
N -3.4859781 -1.8886098
5.7926300
C -3.9770531 -1.6635937
7.1026517
C -2.9759344 -1.3779798
8.0607887
C -3.3387191 -0.9862377
9.3477797
C -4.6838600 -0.9079478
9.6896926
C -5.6699114 -1.1893229
8.7460379
C -5.3218150 -1.5520181
7.4499921
Ni -1.7823493 -1.0903189
5.6837025

C -0.5829784 -2.2591158
9.5592569
C -5.1653386 -3.5641907
5.1731322
H -0.7700436 3.0388899
1.5742205
H 0.9769694 3.5371393
3.2473564
H 1.3418068 2.0559589
5.1735246
H -2.1937996 1.0478377
1.7877468
H -5.0276209 -3.9511976
2.7796284
H -4.4316114 -3.8667587
0.4399149
H -2.5743988 -2.4055655 -
0.3521047
H -1.3721060 -1.0134105
1.2648164
H 2.4305486 0.0712486
4.9056005
H 4.3789127 -0.4242851
6.3042169
H 4.0390518 -1.4552554
8.5477168
H 1.8213029 -2.0019889
9.3349153
H -2.5925891 -0.6870733
10.0676829
H -4.9626375 -0.5882532
10.6851523
H -6.7148220 -1.0883140
9.0083597
H -6.0983159 -1.6872702
6.7129855
H -1.5569470 -2.6838547
9.7778490
H 0.1391718 -3.0753645
9.5821612
H -0.3173935 -1.5614406
10.3571295
H -5.2515570 -3.7370286
6.2405794
H -6.1296129 -3.2161511
4.7950463
H -4.9561522 -4.5260426
4.7048235

NiMe$_2$PhenTAA_3eOX_Quartet:
55 atoms
C -0.5884012 2.3547611
2.3819650
C 0.3977527 2.6362066
3.3266821
C 0.6175280 1.7776825
4.4037261
C -0.1378466 0.6099085
4.5174476
C -1.1510392 0.3208744
3.5470492
C -1.3757124 1.2089565
2.4944113
N -1.9051950 -0.8054692
3.8580350
N -0.1137000 -0.2946575
5.5735328
C 0.9464835 -0.5846497
6.3709513
C -2.5883810 -1.5919307
2.9869110
C -3.6228415 -2.5021848
3.4528770
C -4.2857297 -3.2709598
2.4783657
C -3.9614460 -3.2190838
1.1285013
C -2.9166661 -2.4003397
0.7029309
C -2.2431886 -1.6075288
1.6159924
C 2.2644151 -0.3225001
5.9313842
C 3.3666011 -0.6099008
6.7177502

C 3.1937445 -1.1814458
7.9773279
C 1.9151968 -1.5056247
8.4135425
C 0.7609633 -1.2538827
7.6490074
C -4.0612294 -2.6259413
4.8295556
C -0.5226833 -1.6184165
8.2165043
N -1.6462533 -1.4023552
7.5399010
N -3.4727091 -1.9225311
5.7916871
C -3.9651592 -1.6557593
7.0577676
C -2.9351082 -1.3622969
8.0436326
C -3.3052636 -0.9459720
9.3271330
C -4.6437516 -0.8542799
9.6503106
C -5.6474669 -1.1404412
8.6897441
C -5.3135527 -1.5183771
7.4051892
Ni -1.7675226 -1.1302956
5.6801423
C -0.5733070 -2.3242261
9.5424720
C -5.1310373 -3.6217315
5.1801426
H -0.7730365 3.0491656
1.5725888
H 0.9752169 3.5481359
3.2474408
H 1.3283850 2.0516264
5.1700587
H -2.1887056 1.0480887
1.8010327
H -5.0859613 -3.9339762
2.7672794
H -4.5013906 -3.8348448
0.4213503
H -2.6151415 -2.3944658 -
0.3370358
H -1.3915349 -1.0331341
1.2834904
H 2.4181094 0.0538780
4.9311467
H 4.3612520 -0.4060008
6.3414674
H 4.0479202 -1.4004995
8.6043449
H 1.8285413 -1.9661870
9.3850751
H -2.5578158 -0.6525062
10.0484238
H -4.9365250 -0.5259122
10.6397706
H -6.6890630 -1.0256781
8.9626390
H -6.0897704 -1.6592624
6.6685307
H -1.5534294 -2.7452007
9.7400616
H 0.1410181 -3.1476142
9.5465018
H -0.3035170 -1.6553158
10.3640320
H -5.1982712 -3.7836498
6.2507245
H -6.1097422 -3.3074448
4.8078392
H -4.9020318 -4.5828098
4.7196645

NiMe$_2$PhenTAA_3eOX_Sextet:
55 atoms
C -0.4655823 2.2950420
2.2718460
C 0.5206490 2.5761123
3.2162016
C 0.7197531 1.7293986
4.3043625

C -0.0408997 0.5629300
4.4437530
C -1.0674599 0.2705023
3.4609233
C -1.2673016 1.1632089
2.4018455
N -1.8752234 -0.8142305
3.7542677
N -0.0116917 -0.2834726
5.5382115
C 1.0064684 -0.5294863
6.3967750
C -2.6293361 -1.5650281
2.9164310
C -3.7120308 -2.4162024
3.4218912
C -4.4466652 -3.1277406
2.4545500
C -4.1561767 -3.0963780
1.0958057
C -3.0605332 -2.3638684
0.6407024
C -2.3137820 -1.6230071
1.5365778
C 2.3450981 -0.2958752
5.9963064
C 3.4160171 -0.5188868
6.8404232
C 3.1901764 -1.0036893
8.1281229
C 1.8972229 -1.3208385
8.5271655
C 0.7662008 -1.1408584
7.7085157
C -4.1218250 -2.5984324
4.8141955
C -0.5157359 -1.5716303
8.2657796
N -1.6538761 -1.3713692
7.6114282
N -3.5503037 -1.9112982
5.7964168
C -3.9886032 -1.7056609
7.0850175
C -2.9469484 -1.4089603
8.0818724
C -3.3334807 -1.0389025
9.3798742
C -4.6670323 -0.9892302
9.7165747
C -5.6723935 -1.2758504
8.7545038
C -5.3375974 -1.6100993
7.4620496
Ni -1.7323002 -1.1634674
5.6530047
C -0.5317954 -2.3492345
9.5543189
C -5.1353232 -3.6599008
5.1480868
H -0.6355394 2.9764921
1.4483005
H 1.1157157 3.4756088
3.1252491
H 1.4297052 2.0078695
5.0700159
H -2.0813157 1.0074318
1.7083816
H -5.2877755 -3.7309927
2.7569126
H -4.7628572 -3.6665969
0.4045070
H -2.7855058 -2.3812154 -
0.4064098
H -1.4300670 -1.1091494
1.1872960
H 2.5321838 0.0195745
4.9801831
H 4.4242217 -0.3272348
6.4951905
H 4.0161802 -1.1656229
8.8082698
H 1.7805093 -1.7177727
9.5229712

H -2.5871368 -0.7525772
10.1055814
H -4.9593628 -0.7021795
10.7188786
H -6.7144877 -1.2026691
9.0393774
H -6.1160354 -1.7584225
6.7287980
H -1.4956326 -2.8201805
9.7209057
H 0.2218662 -3.1356490
9.5322613
H -0.3095266 -1.7069272
10.4112828
H -5.1590183 -3.8636472
6.2141267
H -6.1402052 -3.3664783
4.8310307
H -4.8916047 -4.5912570
4.6380892

NiPh$_2$PhenTAA:
NiPh$_2$PhenTAA_2eRED_CSS:
69 atoms
C -0.5684526 -2.9916475
0.7609357
C 0.6076671 -3.3217092
1.4360728
C 0.7877700 -2.9180705
2.7553995
C -0.1898387 -2.1711518
3.4328106
C -1.4025410 -1.8304553
2.7364738
C -1.5588098 -2.2594300
1.4082587
N -2.3504180 -1.2060786
3.5185176
N -0.1807249 -1.8163192
4.7647021
C 0.9267354 -1.5594041
5.5226390
C -3.3310344 -0.3630170
3.0771810
C -4.4849447 -0.0577946
3.9114343
C -5.3840387 0.9194813
3.3966673
C -5.2428331 1.5322347
2.1703227
C -4.1282052 1.2303803
1.3761396
C -3.2014642 0.3201435
1.8427339
C 2.1508060 -1.1820347
4.9171690
C 3.2828159 -0.8495389
5.6333521
C 3.2235662 -0.8455366
7.0335775
C 2.0390789 -1.1672069
7.6602961
C 0.8526721 -1.5591449
6.9768533
C -4.7031046 -0.6133228
5.2250774
C -0.3482490 -1.8404277
7.7257761
N -1.5523482 -1.9705401
7.1062092
N -3.7452913 -1.3526761
5.8468759
C -3.9137104 -2.1783583
6.9280678
C -2.6930435 -2.5227225
7.6286821
C -2.7691130 -3.4427799
8.6967725
C -3.9697369 -4.0042772
9.0818952
C -5.1553621 -3.6694286
8.4017621
C -5.1204683 -2.7789006
7.3477996

Ni -1.9365475 -1.4607153
5.3346214
C -0.3188537 -1.8040101
9.2025381
C 0.5736257 -2.5742415
9.9743264
C 0.5718689 -2.5227655
11.3618485
C -0.3266047 -1.7025094
12.0426099
C -1.2300961 -0.9403824
11.2958597
C -1.2276564 -0.9933345
9.9126222
C -5.9031453 -0.2284114
5.9964135
C -5.7738342 0.2927117
7.2998744
C -6.8773410 0.6571609
8.0519620
C -8.1707039 0.5123585
7.5410923
C -8.3233554 -0.0143297
6.2593802
C -7.2154499 -0.3786576
5.5058762
H -0.7274282 -3.3233478 -
0.2602435
H 1.3747694 -3.9132591
0.9464698
H 1.6789459 -3.2190533
3.2900316
H -2.4889680 -2.0492205
0.8972350
H -6.2185894 1.2214785
4.0114480
H -5.9752089 2.2649658
1.8467188
H -3.9685135 1.7279461
0.4247465
H -2.3069552 0.1406284
1.2632219
H 2.1760570 -1.1164570
3.8385790
H 4.1894944 -0.5601147
5.1114285
H 4.0866529 -0.5603444
7.6266608
H 2.0086340 -1.0921976
8.7368997
H -1.8609827 -3.7390610
9.2000591
H -3.9879787 -4.7286565
9.8893759
H -6.0957670 -4.1331888
8.6804751
H -6.0302303 -2.5620522
6.8082783
H 1.2667023 -3.2305545
9.4626175
H 1.2721608 -3.1385316
11.9180777
H -0.3341519 -1.6665071
13.1261296
H -1.9420983 -0.2962444
11.8015447
H -1.9439682 -0.4094513
9.3488912
H -4.7795644 0.3959787
7.7152889
H -6.7311192 1.0617830
9.0481898
H -9.0350086 0.7904787
8.1335371
H -9.3174986 -0.1540566
5.8456965
H -7.3536706 -0.8031005
4.5191637

NiPh$_2$PhenTAA_2eRED_Triplet:
69 atoms
C -0.6313497 -3.1165314
0.8161072
C 0.5399967 -3.4448861
1.4884297
C 0.7361008 -3.0072096
2.7997357
C -0.2239512 -2.2300359
3.4601900
C -1.4338591 -1.8903718
2.7654272
C -1.6085847 -2.3497614
1.4537912
N -2.3633192 -1.2204333
3.5432652
N -0.2011589 -1.8283515
4.7852625
C 0.9125444 -1.5385544
5.5221541
C -3.3165626 -0.3509163
3.0930272
C -4.4348658 0.0310611
3.9398119
C -5.2764189 1.0605993
3.4491203
C -5.1446005 1.6404030
2.2004123
C -4.0931960 1.2358933
1.3758909
C -3.2043322 0.2768908
1.8287930
C 2.1459006 -1.2233266
4.9021179
C 3.2702866 -0.8292455
5.6058307
C 3.2008163 -0.7025659
6.9943431
C 2.0073768 -0.9864680
7.6330315
C 0.8439316 -1.4531672
6.9716776
C -4.6897441 -0.5290712
5.2577148
C -0.3468757 -1.7525683
7.7517337
N -1.5652736 -1.8327049
7.1534107
N -3.7232791 -1.2248400
5.9139956
C -3.9127670 -2.1041730
6.9763299
C -2.7031661 -2.4454875
7.6704834
C -2.7586307 -3.3937255
8.6995263
C -3.9647596 -4.0029061
9.0536625
C -5.1340979 -3.6724231
8.3832222
C -5.1077369 -2.7301101
7.3523793
Ni -1.9372781 -1.4111385
5.3601948
C -0.2685075 -1.7726715
9.2197220
C 0.6959359 -2.5213717
9.9276435
C 0.7190030 -2.5596214
11.3124535
C -0.2232706 -1.8474552
12.0623875
C -1.1905760 -1.1061823
11.3822494
C -1.2174290 -1.0713340
9.9975983
C -5.9256152 -0.1762819
5.9717688
C -5.8778703 0.2482553
7.3188929
C -7.0298189 0.5476725
8.0277986
C -8.2876365 0.4315442
7.4343570
C -8.3596445 0.0012385
6.1050619
C -7.2077642 -0.2936342
5.3937555
H -0.8014430 -3.4662565 -
0.1972315
H 1.2984364 -4.0548301
1.0080004
H 1.6284256 -3.3055427
3.3335629
H -2.5376405 -2.1374563
0.9420163
H -6.0614191 1.4278392
4.0936741
H -5.8322210 2.4198712
1.8894441
H -3.9437140 1.6895723
0.4008432
H -2.3513128 0.0268033
1.2151868
H 2.1880372 -1.2442975
3.8229477
H 4.1829421 -0.5878576
5.0694826
H 4.0535188 -0.3554707
7.5683970
H 1.9580028 -0.8274492
8.7001787
H -1.8516087 -3.6808067
9.2087035
H -3.9751347 -4.7395744
9.8502815
H -6.0739007 -4.1462963
8.6471189
H -6.0182646 -2.5038983
6.8193361
H 1.4213250 -3.0946590
9.3638526
H 1.4708696 -3.1595842
11.8158476
H -0.2114696 -1.8828326
13.1458878
H -1.9363730 -0.5493528
11.9404503
H -1.9823423 -0.5014899
9.4870460
H -4.9118646 0.3319533
7.7988412
H -6.9468878 0.8741535
9.0594819
H -9.1884207 0.6548985
7.9947748
H -9.3263235 -0.1161961
5.6250821
H -7.2841694 -0.6442729
4.3720324

NiPh$_2$PhenTAA_2eRED_Quintet:
69 atoms
C -0.7177329 -3.1931934
0.9040329
C 0.4499926 -3.5361031
1.5747074
C 0.6708407 -3.0647410
2.8690454
C -0.2502209 -2.2341563
3.5232588
C -1.4672789 -1.8738821
2.8219156
C -1.6624881 -2.3782477
1.5284937
N -2.3844478 -1.1510007
3.5622892
N -0.1747785 -1.8092183
4.8353524
C 0.9462113 -1.5365565
5.5662991
C -3.3510216 -0.2994060
3.1001296
C -4.4618057 0.1165867
3.9496134
C -5.2804822 1.1523017
3.4415044
C -5.1517487 1.7077204
2.1785525
C -4.1225985 1.2620857
1.3528233
C -3.2511775 0.2908329
1.8167093
C 2.1899990 -1.2796170
4.9371444
C 3.3186881 -0.8604572
5.6207546
C 3.2523920 -0.6631078
6.9979932
C 2.0531810 -0.9086626
7.6480019
C 0.8846770 -1.3952342
7.0177559
C -4.7746063 -0.4067877
5.2904841
C -0.2874070 -1.6693611
7.8676901
N -1.5465727 -1.5694540
7.3601890
N -3.7949420 -0.9408437
6.0684966
C -3.9328459 -1.8384133
7.1208881
C -2.7048449 -2.1840760
7.8236522
C -2.7657662 -3.1876854
8.8059635
C -3.9580453 -3.8315116
9.1320072
C -5.1286617 -3.5011952
8.4634944
C -5.1086653 -2.5265895
7.4674291
Ni -1.9499872 -1.2769327
5.4510476
C -0.0830370 -1.8357086
9.3098075
C 0.9333954 -2.6533215
9.8547934
C 1.0838868 -2.8211048
11.2203568
C 0.2279505 -2.1728912
12.1204391
C -0.7825426 -1.3599121
11.6051407
C -0.9390798 -1.1952958
10.2384160
C -6.1048846 -0.1466606
5.8511593
C -6.2498033 0.2971147
7.1877186
C -7.4969450 0.5201833
7.7487256
C -8.6640620 0.3093896
7.0133380
C -8.5453565 -0.1343535
5.6898269
C -7.3003449 -0.3522238
5.1255403
H -0.9095225 -3.5674771 -
0.0966954
H 1.1859241 -4.1817713
1.1058331
H 1.5589811 -3.3760334
3.4032830
H -2.5901597 -2.1548034
1.0182297
H -6.0547059 1.5447926
4.0849812
H -5.8249558 2.4972290
1.8617975
H -3.9755359 1.6882477
0.3648954
H -2.4132021 0.0054035
1.1974362
H 2.2335918 -1.3582538
3.8604972
H 4.2342180 -0.6591353
5.0723151
H 4.1068156 -0.2940950
7.5552958
H 2.0069662 -0.7061385
8.7085056
H -1.8569815 -3.4933247
9.2989798
H -3.9562965 -4.5994494
9.8988393
H -6.0607405 -4.0057675
8.6966824

H -6.0157778 -2.3209146
6.9223592
H 1.5977888 -3.1717117
9.1747531
H 1.8683638 -3.4727996
11.5928273
H 0.3411886 -2.3082451
13.1901296
H -1.4599213 -0.8468115
12.2806766
H -1.7352726 -0.5693110
9.8581186
H -5.3567386 0.4548817
7.7776896
H -7.5608896 0.8628319
8.7768094
H -9.6387948 0.4742757
7.4584097
H -9.4371231 -0.3224644
5.0997077
H -7.2292524 -0.7064637
4.1046623

NiPh$_2$PhenTAA_1eRED_Doublet:
69 atoms
C -0.5566870 -2.9896898
0.7422965
C 0.6172225 -3.3195465
1.4159963
C 0.7906272 -2.9356459
2.7412422
C -0.1949941 -2.2026234
3.4137742
C -1.3978962 -1.8643836
2.7232992
C -1.5564277 -2.2760566
1.3941816
N -2.3579205 -1.2551762
3.5208961
N -0.1989458 -1.8627254
4.7604418
C 0.8903391 -1.5502157
5.5076589
C -3.2995356 -0.3715924
3.1020769
C -4.4241781 -0.0230949
3.9497186
C -5.3102773 0.9795085
3.4731504
C -5.1715093 1.5990474
2.2529249
C -4.0818179 1.2581383
1.4394476
C -3.1791033 0.3097936
1.8610352
C 2.1231536 -1.1806189
4.9055209
C 3.2241635 -0.7959484
5.6346253
C 3.1547964 -0.7435178
7.0338268
C 1.9713682 -1.0703486
7.6539953
C 0.8107163 -1.4968390
6.9551531
C -4.6624001 -0.6048871
5.2492874
C -0.3808999 -1.8111925
7.7071233
N -1.5695215 -1.9796551
7.1170621
N -3.7501243 -1.3652199
5.8651908
C -3.9361907 -2.1516435
6.9982732
C -2.7293452 -2.4918423
7.6910551
C -2.8004006 -3.3804758
8.7766273
C -4.0094354 -3.9108001
9.1892210
C -5.1875757 -3.5786409
8.5129662
C -5.1464541 -2.7191008
7.4300534

Ni -1.9541987 -1.5428404
5.3255759
C -0.3042971 -1.8221902
9.1950409
C 0.5245410 -2.7085050
9.8924280
C 0.5567869 -2.7143295
11.2812480
C -0.2362092 -1.8283312
12.0054541
C -1.0602223 -0.9363863
11.3229429
C -1.0942999 -0.9360404
9.9356756
C -5.9111785 -0.2422143
5.9766626
C -5.8286369 0.3967401
7.2187640
C -6.9733106 0.7277729
7.9299594
C -8.2321025 0.4233797
7.4170166
C -8.3292799 -0.2103202
6.1811957
C -7.1818983 -0.5362580
5.4690061
H -0.7088485 -3.3107412 -
0.2817592
H 1.3876584 -3.8998099
0.9214004
H 1.6784505 -3.2433247
3.2766639
H -2.4885116 -2.0722848
0.8851020
H -6.1273890 1.2876144
4.1080390
H -5.8790778 2.3592932
1.9449955
H -3.9255256 1.7602657
0.4909460
H -2.3112626 0.1049691
1.2522952
H 2.1730812 -1.1544138
3.8272949
H 4.1319035 -0.5040475
5.1178786
H 4.0062815 -0.4219488
7.6212138
H 1.9201318 -0.9789007
8.7285649
H -1.8951364 -3.6847634
9.2769653
H -4.0313590 -4.6084181
10.0179886
H -6.1328468 -4.0158786
8.8118395
H -6.0556964 -2.5119378
6.8889670
H 1.1407471 -3.3994535
9.3307771
H 1.1999625 -3.4158632
11.8000260
H -0.2136766 -1.8333633
13.0886370
H -1.6801148 -0.2394429
11.8747580
H -1.7476978 -0.2536949
9.4069127
H -4.8506573 0.6187468
7.6266072
H -6.8837524 1.2236333
8.8894360
H -9.1260609 0.6759609
7.9745519
H -9.3025212 -0.4558912
5.7719719
H -7.2595223 -1.0307890
4.5088350

NiPh$_2$PhenTAA_1eRED_Quartet:
69 atoms
C -0.6608773 -3.0841235
0.8629753
C 0.5256641 -3.4175650
1.5441294

C 0.7469693 -2.9752676
2.8315100
C -0.2167615 -2.1797177
3.4934226
C -1.4440686 -1.8344703
2.7886635
C -1.6266889 -2.3080956
1.4687352
N -2.3102716 -1.1117130
3.5314501
N -0.1704235 -1.7142195
4.7605138
C 0.9651869 -1.5049750
5.5325344
C -3.3330323 -0.2961228
3.0640665
C -4.4627438 0.0074939
3.9102607
C -5.3675527 0.9729739
3.3993056
C -5.2343257 1.5647680
2.1570170
C -4.1424261 1.2523040
1.3504425
C -3.2024817 0.3493665
1.8266020
C 2.1763877 -1.1618467
4.9158670
C 3.3190199 -0.8435824
5.6358988
C 3.2444638 -0.8181084
7.0266528
C 2.0518978 -1.1138218
7.6603065
C 0.8706678 -1.4934914
6.9730263
C -4.6776218 -0.5547391
5.2306668
C -0.3399282 -1.7772599
7.7213940
N -1.5438257 -1.8782968
7.1041523
N -3.7101391 -1.2676641
5.8601668
C -3.8919939 -2.1484120
6.9254777
C -2.6880109 -2.4880479
7.6165784
C -2.7389423 -3.4376337
8.6433996
C -3.9435861 -4.0405494
8.9995377
C -5.1137008 -3.7102615
8.3281308
C -5.0874504 -2.7748836
7.2957011
Ni -1.9041421 -1.3229238
5.3459594
C -0.3042046 -1.7646821
9.2007364
C 0.5670616 -2.5758935
9.9456286
C 0.5415708 -2.5783210
11.3331312
C -0.3591192 -1.7692148
12.0238735
C -1.2351564 -0.9629601
11.3007992
C -1.2105024 -0.9632820
9.9141737
C -5.8923621 -0.1884779
5.9925327
C -5.7712656 0.3255533
7.2938819
C -6.8896692 0.6343370
8.0535989
C -8.1695564 0.4337324
7.5418745
C -8.3098479 -0.0837995
6.2552788
C -7.1899649 -0.3899564
5.4950227
H -0.8293974 -3.4629932 -
0.1381885

H 1.2613306 -4.0505054
1.0620199
H 1.6390479 -3.2714609
3.3649953
H -2.5542456 -2.0929247
0.9574455
H -6.1953432 1.2821281
4.0184157
H -5.9694011 2.2923335
1.8323280
H -4.0037961 1.7246307
0.3855616
H -2.3113604 0.1599019
1.2446259
H 2.2010380 -1.1071726
3.8364297
H 4.2355643 -0.5884481
5.1180507
H 4.1077002 -0.5391685
7.6200822
H 2.0180848 -1.0286495
8.7353498
H -1.8322068 -3.7242917
9.1526191
H -3.9554226 -4.7766161
9.7949410
H -6.0515765 -4.1848780
8.5922699
H -5.9975252 -2.5490417
6.7624032
H 1.2558367 -3.2249357
9.4191412
H 1.2202842 -3.2237721
11.8793449
H -0.3837803 -1.7746346
13.1069636
H -1.9457516 -0.3316424
11.8219447
H -1.9054860 -0.3465754
9.3590597
H -4.7801151 0.4685179
7.7045042
H -6.7623001 1.0311150
9.0542794
H -9.0431760 0.6674527
8.1384155
H -9.2983723 -0.2609647
5.8465927
H -7.3108766 -0.8125541
4.5053284

NiPh$_2$PhenTAA_1eRED_Sextet:
69 atoms
C -0.7916356 -3.2478776
1.1210153
C 0.4181422 -3.5216572
1.7892579
C 0.7052132 -2.9223905
2.9936044
C -0.1991464 -2.0036938
3.5918606
C -1.4768857 -1.7453768
2.9078689
C -1.7145915 -2.3822021
1.6664118
N -2.3434256 -0.9691943
3.5844785
N -0.0379642 -1.3783199
4.7651361
C 1.1099145 -1.3533940
5.5522938
C -3.4161545 -0.2355881
3.0793331
C -4.5318711 0.1320041
3.9298046
C -5.4478926 1.0528663
3.3595408
C -5.3619222 1.5291873
2.0637788
C -4.3030753 1.1365352
1.2512872
C -3.3435939 0.2825410
1.7782144
C 2.3527377 -1.1205826
4.9543850

C 3.5161061 -0.9294183 5.6896509
C 3.4265205 -0.9399146 7.0788418
C 2.2095155 -1.1589383 7.6968359
C 0.9997856 -1.4171932 6.9951911
C -4.7684352 -0.3412555 5.2908857
C -0.2126607 -1.6896028 7.7498092
N -1.4620183 -1.6268370 7.1807247
N -3.7475971 -0.8406727 6.0485002
C -3.8569568 -1.8191029 7.0339357
C -2.6211787 -2.2400361 7.6532527
C -2.6523388 -3.2941517 8.5753709
C -3.8397244 -3.9338361 8.9159139
C -5.0278106 -3.5375851 8.3184339
C -5.0313154 -2.5039740 7.3869616
Ni -1.8345991 -0.7470569 5.4810367
C -0.1235539 -1.8277655 9.2216208
C 0.7273415 -2.7535695 9.8473348
C 0.7518180 -2.8937466 11.2273393
C -0.0751247 -2.1103598 12.0310756
C -0.9287234 -1.1904268 11.4277895
C -0.9557405 -1.0546185 10.0474774
C -6.0821207 -0.0967970 5.9154956
C -6.1486949 0.4353668 7.2166498
C -7.3639185 0.6407861 7.8519616
C -8.5602165 0.3185150 7.2149949
C -8.5158129 -0.2152245 5.9264280
C -7.3010581 -0.4169620 5.2896659
H -1.0134441 -3.7470935 0.1851165
H 1.1178576 -4.2312880 1.3634352
H 1.6150504 -3.1718233 3.5205621
H -2.6651025 -2.2238093 1.1766792
H -6.2543678 1.4169139 3.9778403
H -6.1060360 2.2297735 1.7023473
H -4.1995825 1.5138513 0.2410976
H -2.4745280 0.0381656 1.1832320
H 2.3836362 -1.0337325 3.8762697
H 4.4562614 -0.7375409 5.1873857
H 4.3027904 -0.7488366 7.6879874
H 2.1791586 -1.1103868 8.7740619
H -1.7281151 -3.6434889 9.0074532
H -3.8228105 -4.7495999 9.6292498
H -5.9586774 -4.0398487 8.5560365
H -5.9605737 -2.2490595 6.9059341
H 1.3596494 -3.3810256 9.2316689
H 1.4110496 -3.6266961 11.6787690
H -0.0605638 -2.2227786 13.1085052
H -1.5829313 -0.5775477 12.0372673
H -1.6366500 -0.3516235 9.5853602
H -5.2229191 0.6713897 7.7252681
H -7.3786671 1.0535929 8.8544443
H -9.5091452 0.4715766 7.7147418
H -9.4356625 -0.4870533 5.4206002
H -7.2800142 -0.8495102 4.2972373

$NiPh_2PhenTAA\_0eNeutral\_CSS$:
69 atoms
C -0.5855845 -3.0436552 0.7701527
C 0.5857279 -3.3724461 1.4424209
C 0.7669138 -2.9782934 2.7646235
C -0.2118859 -2.2297341 3.4192744
C -1.4060602 -1.8942291 2.7336799
C -1.5823236 -2.3187310 1.4162191
N -2.3611842 -1.2645017 3.5429410
N -0.2195425 -1.8668581 4.7727986
C 0.8558316 -1.5214584 5.5080197
C -3.2740119 -0.3602021 3.1363444
C -4.3621661 0.0464684 4.0008259
C -5.2302294 1.0755011 3.5504871
C -5.0977034 1.6808241 2.3275984
C -4.0499717 1.2740907 1.4854726
C -3.1788834 0.2886379 1.8707858
C 2.1062068 -1.1961312 4.9060591
C 3.1792059 -0.7577487 5.6373322
C 3.0950322 -0.6238548 7.0327672
C 1.9124286 -0.9350587 7.6524374
C 0.7702024 -1.3981793 6.9481288
C -4.6242031 -0.5549505 5.2859484
C -0.4075908 -1.7429334 7.7071978
N -1.5794778 -1.9291794 7.1384785
N -3.7437734 -1.3196696 5.8955442
C -3.9493095 -2.1003517 7.0628981
C -2.7567344 -2.4364330 7.7476004
C -2.8152789 -3.3122208 8.8316189
C -4.0314375 -3.8338987 9.2533164
C -5.2006709 -3.5042938 8.5820977
C -5.1584474 -2.6517690 7.4864531
Ni -1.9669639 -1.5708475 5.3335466
C -0.2763442 -1.8301729 9.1932688
C 0.5275414 -2.8000111 9.7944375
C 0.6093132 -2.8836805 11.1786406
C -0.0950129 -1.9854787 11.9752684
C -0.8799609 -1.0026734 11.3807934
C -0.9742065 -0.9290033 9.9968675
C -5.9245917 -0.2379854 5.9506747
C -5.9250849 0.4658635 7.1546113
C -7.1211821 0.7548751 7.7990874
C -8.3285353 0.3335463 7.2510856
C -8.3353024 -0.3631059 6.0460451
C -7.1401730 -0.6384328 5.3936678
H -0.7423747 -3.3769623 -0.2480587
H 1.3487338 -3.9638963 0.9520871
H 1.6504384 -3.2931749 3.3019498
H -2.5170821 -2.1230815 0.9099385
H -6.0182106 1.4097664 4.2083605
H -5.7735495 2.4710990 2.0297054
H -3.9043096 1.7617784 0.5284995
H -2.3485760 0.0410561 1.2284499
H 2.1822221 -1.2303085 3.8306673
H 4.0925775 -0.4847756 5.1214860
H 3.9369760 -0.2608208 7.6067018
H 1.8406278 -0.8035633 8.7215525
H -1.9119581 -3.6178078 9.3317577
H -4.0537006 -4.5210490 10.0894296
H -6.1476013 -3.9307391 8.8874638
H -6.0671574 -2.4466541 6.9463941
H 1.0811305 -3.4906716 9.1710370
H 1.2260769 -3.6475186 11.6355060
H -0.0274550 -2.0478663 13.0541595
H -1.4222631 -0.2949280 11.9952501
H -1.5966748 -0.1753192 9.5313863
H -4.9827764 0.7782534 7.5865861
H -7.1104276 1.3057824 8.7312615
H -9.2604411 0.5518771 7.7573926
H -9.2722275 -0.6890118 5.6118628
H -7.1415734 -1.1720979 4.4516563

$NiPh_2PhenTAA\_0eNeutral\_Triplet$:
69 atoms
C -0.6044397 -3.0081328 0.7985764
C 0.5859747 -3.3416241 1.4818118
C 0.7846816 -2.9461753 2.7827479
C -0.2029373 -2.1930979 3.4588878
C -1.4273597 -1.8497395 2.7559482
C -1.5912362 -2.2804006 1.4189812
N -2.3249900 -1.1788897 3.5152513
N -0.1778709 -1.7814706 4.7481903
C 0.9299734 -1.5193893 5.5171365
C -3.3126890 -0.3285177 3.0805806
C -4.4299538 -0.0159375 3.9319941
C -5.3379949 0.9584752 3.4568222
C -5.2010368 1.5850684 2.2355502
C -4.1055426 1.2824032 1.4253213
C -3.1800943 0.3539959 1.8537194
C 2.1466072 -1.1400397 4.9133759
C 3.2542338 -0.7829856 5.6532502
C 3.1742789 -0.7677970 7.0466323
C 1.9864530 -1.1008600 7.6637959
C 0.8294258 -1.4942596 6.9522978
C -4.6598020 -0.6012047 5.2475077
C -0.3798874 -1.8061156 7.7048688
N -1.5523992 -1.9846447 7.1110467
N -3.7550768 -1.3646680 5.8460851
C -3.9255998 -2.1517820 6.9842372
C -2.7230882 -2.4906174 7.6746977
C -2.7900294 -3.3854334 8.7572932
C -3.9954487 -3.9208541 9.1601850
C -5.1745665 -3.5885566 8.4831944
C -5.1376612 -2.7238764 7.4094430
Ni -1.9329358 -1.4565987 5.3300751
C -0.2990566 -1.8214094 9.1943223
C 0.4870100 -2.7513468 9.8804242
C 0.5147848 -2.7666900 11.2691879
C -0.2352873 -1.8452919 11.9937395
C -1.0125368 -0.9087610 11.3187799
C -1.0465464 -0.8998694 9.9308397
C -5.9123448 -0.2388424 5.9722072
C -5.8280893 0.4475454 7.1855904
C -6.9755779 0.7729807 7.8961642
C -8.2274058 0.4103375 7.4081180
C -8.3225106 -0.2723458 6.1992127
C -7.1743266 -0.5898589 5.4846582
H -0.7550510 -3.3604457 -0.2140209

H 1.3347262 -3.9458194
0.9853531
H 1.6732796 -3.2511661
3.3169725
H -2.5218204 -2.0756387
0.9090518
H -6.1698246 1.2411685
4.0826913
H -5.9274987 2.3254769
1.9264803
H -3.9612704 1.7895113
0.4795119
H -2.2974341 0.1727397
1.2579857
H 2.1895555 -1.0841240
3.8354425
H 4.1668027 -0.4902130
5.1492166
H 4.0263422 -0.4711947
7.6445407
H 1.9381905 -1.0403625
8.7397258
H -1.8857002 -3.6865012
9.2594980
H -4.0189786 -4.6215346
9.9850861
H -6.1172242 -4.0301945
8.7804411
H -6.0466117 -2.5139740
6.8705230
H 1.0699673 -3.4694948
9.3172613
H 1.1212881 -3.5000721
11.7861285
H -0.2131361 -1.8561320
13.0761874
H -1.5946384 -0.1844846
11.8749695
H -1.6631223 -0.1811830
9.4058427
H -4.8533144 0.7159315
7.5729719
H -6.8929038 1.3092660
8.8333587
H -9.1220810 0.6589851
7.9649088
H -9.2923756 -0.5599701
5.8124646
H -7.2492674 -1.1222962
4.5446578

NiPh₂PhenTAA_0eNeutral_Quintet:
69 atoms
C -0.6020593 -3.0622455
0.9252702
C 0.5777820 -3.3608873
1.6313731
C 0.7782639 -2.8760469
2.9075870
C -0.1840550 -2.0606307
3.5309389
C -1.4031979 -1.7339687
2.7951661
C -1.5726359 -2.2738374
1.4939892
N -2.3039206 -1.0003500
3.4786486
N -0.1418861 -1.6082097
4.8185695
C 0.9674018 -1.3972898
5.6006720
C -3.3871855 -0.2970445
2.9761232
C -4.5280613 -0.0179620
3.8269395
C -5.4962871 0.8501018
3.2594038
C -5.4061808 1.3767994
1.9837609
C -4.3053465 1.0920818
1.1830825
C -3.3118205 0.2737820
1.6973297
C 2.2120720 -1.0840556
5.0133513
C 3.3158813 -0.7380665
5.7642846
C 3.2236285 -0.6910091
7.1540038
C 2.0193606 -0.9921493
7.7574153
C 0.8621766 -1.3567258
7.0365091
C -4.7583474 -0.5176468
5.1724238
C -0.3439537 -1.6861080
7.8201256
N -1.5361567 -1.7224214
7.2883638
N -3.7662096 -1.1027945
5.9272003
C -3.9300530 -1.8904638
7.0501477
C -2.7499177 -2.2048100
7.8182106
C -2.8627317 -3.0661189
8.9188366
C -4.0756371 -3.6165247
9.2899703
C -5.2171385 -3.3278718
8.5422059
C -5.1440229 -2.4932016
7.4467903
Ni -1.9185826 -1.1306505
5.4026133
C -0.1335400 -1.9367544
9.2795357
C 0.5918945 -3.0412024
9.7286282
C 0.7476322 -3.2721413
11.0897744
C 0.1988972 -2.3889022
12.0147550
C -0.5060798 -1.2738351
11.5719012
C -0.6754809 -1.0515521
10.2114255
C -6.0449346 -0.1859409
5.8367788
C -6.0401249 0.5342541
7.0374444
C -7.2238361 0.8270430
7.7010699
C -8.4430795 0.4023377
7.1821844
C -8.4648032 -0.3139268
5.9879790
C -7.2807449 -0.6014432
5.3231197
H -0.7610373 -3.4849610 -
0.0586173
H 1.3217902 -4.0075192
1.1833179
H 1.6611769 -3.1645327
3.4598842
H -2.5033570 -2.0991605
0.9744523
H -6.3435197 1.1398642
3.8595328
H -6.1868425 2.0386002
1.6291613
H -4.2002590 1.5251672
0.1970630
H -2.4107699 0.1166356
1.1219024
H 2.2770576 -1.0556175
3.9355756
H 4.2422783 -0.4803381
5.2655496
H 4.0753933 -0.4057636
7.7571626
H 1.9594652 -0.9301393
8.8332059
H -1.9845916 -3.3450472
9.4737426
H -4.1236558 -4.2892745
10.1362424
H -6.1677671 -3.7774521
8.8022316
H -6.0325105 -2.3239433
6.8635116
H 1.0249951 -3.7220693
9.0066252
H 1.2998739 -4.1398248
11.4284591
H 0.3235839 -2.5666851
13.0754748
H -0.9284518 -0.5790741
12.2869353
H -1.2412336 -0.1961735
9.8642257
H -5.0929579 0.8549094
7.4525596
H -7.1947796 1.3885328
8.6270154
H -9.3661756 0.6254516
7.7022700
H -9.4071500 -0.6552477
5.5768517
H -7.3025346 -1.1669483
4.3997621

NiPh₂PhenTAA_1eOX_Doublet:
69 atoms
C -0.6622560 -3.0963296
0.8558143
C 0.5267105 -3.4305056
1.5382889
C 0.7349586 -3.0223555
2.8348832
C -0.2457325 -2.2549274
3.4953512
C -1.4637127 -1.9123171
2.7960444
C -1.6436009 -2.3537050
1.4694803
N -2.3459794 -1.2085055
3.5594961
N -0.2168806 -1.8078903
4.7821793
C 0.8914869 -1.4932146
5.5267936
C -3.2926876 -0.3161859
3.1240907
C -4.3633040 0.0866612
3.9891670
C -5.2277549 1.1041673
3.5398926
C -5.0905794 1.7021810
2.3033485
C -4.0547432 1.2964483
1.4634927
C -3.1762757 0.3120787
1.8672203
C 2.1176349 -1.1758759
4.9072725
C 3.2081812 -0.7449847
5.6343239
C 3.1203685 -0.6069550
7.0185592
C 1.9315838 -0.9104413
7.6511081
C 0.7965076 -1.3657230
6.9520255
C -4.6318325 -0.5170253
5.2957980
C -0.3969121 -1.7102736
7.7273977
N -1.5646426 -1.8632455
7.1609131
N -3.7415972 -1.2498689
5.9109325
C -3.9374311 -2.0531062
7.0686904
C -2.7471236 -2.3888664
7.7518082
C -2.7976514 -3.2882078
8.8183263
C -4.0078687 -3.8344161
9.2152281
C -5.1793213 -3.5037388
8.5431973
C -5.1451610 -2.6257569
7.4715178
Ni -1.9512643 -1.4653045
5.3583001
C -0.2458793 -1.8332908
9.2040694
C 0.5799204 -2.8072989
9.7694780
C 0.6746096 -2.9228542
11.1504417
C -0.0320488 -2.0510230
11.9738912
C -0.8353809 -1.0625994
11.4132001
C -0.9492336 -0.9584741
10.0334980
C -5.9472710 -0.2260026
5.9310283
C -5.9771638 0.4602865
7.1459432
C -7.1903185 0.7297913
7.7648164
C -8.3799889 0.3015445
7.1833796
C -8.3551763 -0.3791385
5.9694289
C -7.1444572 -0.6311728
5.3369462
H -0.8195630 -3.4637700 -
0.1497574
H 1.2657536 -4.0498329
1.0472005
H 1.6200093 -3.3344875
3.3701635
H -2.5760767 -2.1549143
0.9614035
H -6.0188042 1.4411773
4.1917579
H -5.7691088 2.4887510
2.0029119
H -3.9134506 1.7723956
0.5013641
H -2.3378634 0.0636898
1.2346570
H 2.1820193 -1.2058896
3.8303132
H 4.1228770 -0.4854495
5.1164886
H 3.9639109 -0.2482803
7.5922873
H 1.8669828 -0.7785414
8.7200885
H -1.8945443 -3.5911084
9.3199428
H -4.0285476 -4.5415540
10.0338435
H -6.1207378 -3.9509231
8.8337059
H -6.0541388 -2.4174741
6.9336454
H 1.1360760 -3.4784464
9.1272095
H 1.3044099 -3.6891787
11.5835537
H 0.0491471 -2.1370858
13.0497898
H -1.3747989 -0.3741399
12.0508705
H -1.5850342 -0.1997263
9.5949142
H -5.0492226 0.7793798
7.6035271
H -7.2075913 1.2718411
8.7015593
H -9.3247247 0.5041813
7.6712140
H -9.2791443 -0.7087189
5.5121157
H -7.1235658 -1.1524728
4.3881673

NiPh₂PhenTAA_1eOX_Quartet:
69 atoms
C -0.5686079 -2.9668649
0.7623421
C 0.6156593 -3.2989641
1.4417609

C 0.8109627 -2.9025679 2.7467066
C -0.1667760 -2.1448855 3.4190184
C -1.3913811 -1.8013857 2.7163582
C -1.5565495 -2.2386866 1.3884623
N -2.3344714 -1.1570470 3.4731722
N -0.1306626 -1.7759785 4.7379538
C 0.9648556 -1.5389770 5.5221572
C -3.3384543 -0.3289242 3.0517176
C -4.4415604 -0.0021006 3.9264157
C -5.3591231 0.9645854 3.4642131
C -5.2504277 1.5827298 2.2348821
C -4.1796046 1.2636380 1.4018788
C -3.2480246 0.3331554 1.8068265
C 2.2056965 -1.1990951 4.9381604
C 3.2982679 -0.8386299 5.6956989
C 3.1963102 -0.7943367 7.0848905
C 1.9932886 -1.1059625 7.6855388
C 0.8454093 -1.4908229 6.9615146
C -4.6927072 -0.5699995 5.2630717
C -0.3656429 -1.7893860 7.7469375
N -1.5424321 -1.9260095 7.1923057
N -3.8016283 -1.2892148 5.8954562
C -3.9528254 -2.0347923 7.0912664
C -2.7516479 -2.3734187 7.7807459
C -2.8227560 -3.2451597 8.8731214
C -4.0341418 -3.7621360 9.2968256
C -5.2057163 -3.4318163 8.6243781
C -5.1618579 -2.5857220 7.5305512
Ni -1.9322571 -1.4704289 5.3222061
C -0.2194242 -1.8636754 9.2312483
C 0.5308785 -2.8738566 9.8372134
C 0.6024604 -2.9531820 11.2221831
C -0.0540712 -2.0120887 12.0102708
C -0.7843160 -0.9915015 11.4092746
C -0.8739463 -0.9216807 10.0251455
C -5.9931145 -0.2365499 5.9171618
C -5.9883369 0.5179780 7.0904823
C -7.1815187 0.8084410 7.7390405
C -8.3859947 0.3336588 7.2292373
C -8.3960553 -0.4176235 6.0574022
C -7.2059249 -0.6927457 5.3960025
H -0.7214888 -3.3161379 -0.2503762
H 1.3662089 -3.9016603 0.9474145
H 1.6950617 -3.2193201 3.2806427
H -2.4897327 -2.0456882 0.8796319
H -6.1760969 1.2530610 4.1057083
H -5.9790733 2.3246578 1.9378347
H -4.0583399 1.7623239 0.4486518
H -2.3845296 0.1490799 1.1859582
H 2.2755286 -1.1582604 3.8618371
H 4.2232402 -0.5647706 5.2043157
H 4.0404601 -0.4953887 7.6912466
H 1.9288009 -1.0307261 8.7590250
H -1.9218074 -3.5480418 9.3782426
H -4.0579107 -4.4417539 10.1384264
H -6.1534568 -3.8508843 8.9357183
H -6.0726654 -2.3778945 6.9959084
H 1.0472645 -3.6011947 9.2231151
H 1.1744978 -3.7465209 11.6859537
H 0.0084208 -2.0707770 13.0892361
H -1.2866357 -0.2511500 12.0186335
H -1.4555221 -0.1375917 9.5564735
H -5.0487017 0.8726622 7.4953191
H -7.1709101 1.4026769 8.6437358
H -9.3146859 0.5531609 7.7399721
H -9.3313755 -0.7856577 5.6556838
H -7.2133816 -1.2711736 4.4804947

NiPh$_2$PhenTAA_1eOX_Sextet:
69 atoms
C -0.7191924 -3.1466894 1.0006481
C 0.4654013 -3.4621445 1.6879437
C 0.7161232 -2.9416189 2.9440649
C -0.1954674 -2.0673032 3.5511172
C -1.4227295 -1.7291228 2.8392184
C -1.6492912 -2.3075596 1.5652816
N -2.2870106 -0.9410148 3.5166421
N -0.1067533 -1.5759818 4.8310902
C 1.0080937 -1.4076466 5.5991036
C -3.3990122 -0.2692499 3.0013350
C -4.5511107 0.0091915 3.8319908
C -5.5249520 0.8611542 3.2502951
C -5.4316579 1.3703727 1.9663696
C -4.3200853 1.0864087 1.1880453
C -3.3137019 0.2912456 1.7252377
C 2.2705515 -1.1762730 5.0082337
C 3.3814171 -0.8701779 5.7592423
C 3.2916196 -0.7772985 7.1535594
C 2.0833895 -1.0036997 7.7652367
C 0.9082818 -1.3418482 7.0459204
C -4.7701211 -0.4414405 5.1899224
C -0.2903992 -1.6255181 7.8127872
N -1.4806138 -1.6736186 7.2315827
N -3.7091836 -0.9936156 5.9537705
C -3.8537465 -1.9242931 6.9047803
C -2.6475747 -2.2725615 7.6756386
C -2.7387523 -3.2644052 8.6574938
C -3.9350113 -3.9081328 8.9072669
C -5.0932899 -3.5952911 8.1642589
C -5.0587684 -2.6423383 7.1866295
Ni -1.8390020 -0.9617244 5.4051795
C -0.1832015 -1.8038193 9.2853561
C 0.6240066 -2.7968351 9.8486286
C 0.6516615 -2.9747465 11.2259160
C -0.1037368 -2.1479549 12.0523827
C -0.8928394 -1.1437690 11.4979081
C -0.9415870 -0.9791329 10.1213115
C -5.9948888 -0.1069420 5.9326509
C -5.9052636 0.4684974 7.2119213
C -7.0450013 0.7520335 7.9489139
C -8.3037960 0.4511100 7.4377690
C -8.4113246 -0.1334973 6.1776676
C -7.2748920 -0.4045133 5.4316291
H -0.9150816 -3.5945097 0.0353680
H 1.1763216 -4.1466160 1.2437785
H 1.6013372 -3.2463850 3.4831006
H -2.5851676 -2.1180388 1.0610234
H -6.3712221 1.1609847 3.8462139
H -6.2156709 2.0161070 1.5931649
H -4.2115211 1.4991453 0.1941386
H -2.4048418 0.1439044 1.1596651
H 2.3421141 -1.1736522 3.9308777
H 4.3215332 -0.6688129 5.2611816
H 4.1570468 -0.5068638 7.7427689
H 2.0199315 -0.8977938 8.8369601
H -1.8611998 -3.5561794 9.2097827
H -3.9751064 -4.6794646 9.6649428
H -6.0129051 -4.1324044 8.3566214
H -5.9392454 -2.4374145 6.6016951
H 1.2142916 -3.4368504 9.2052981
H 1.2665341 -3.7557245 11.6543677
H -0.0734290 -2.2817631 13.1260372
H -1.4707614 -0.4903542 12.1387446
H -1.5648542 -0.2073121 9.6873293
H -4.9310175 0.7059725 7.6193389
H -6.9517461 1.2112955 8.9248701
H -9.1928630 0.6653933 8.0163838
H -9.3854348 -0.3870759 5.7790918
H -7.3686299 -0.8828222 4.4653137

NiPh$_2$PhenTAA_2eOX_CSS:
69 atoms
C -0.7309463 -3.1277833 0.9332795
C 0.4796355 -3.4682012 1.6281886
C 0.7117333 -3.0554893 2.9022611
C -0.2705833 -2.2675061 3.5717468
C -1.5205153 -1.9157584 2.8540906
C -1.7019973 -2.3766418 1.5166266
N -2.3364256 -1.1560969 3.5705523
N -0.2088686 -1.7552142 4.7923377
C 0.9195894 -1.4748941 5.5453169
C -3.3120924 -0.2842678 3.1154556
C -4.3679072 0.1232111 3.9874286
C -5.2274090 1.1305476 3.5313701
C -5.0864506 1.7162613 2.2801601
C -4.0602620 1.3066999 1.4376946
C -3.1809697 0.3235999 1.8555936
C 2.1338599 -1.1705610 4.9074688
C 3.2357417 -0.7440259 5.6272537
C 3.1432451 -0.5977325 7.0058570
C 1.9490449 -0.8884827 7.6522054
C 0.8146567 -1.3355396 6.9632109
C -4.6420468 -0.4795782 5.3080860
C -0.3874139 -1.6784202 7.7508700
N -1.5563421 -1.8015833 7.1840940
N -3.7391037 -1.1863593 5.9309664
C -3.9292517 -2.0117182 7.0758925
C -2.7395721 -2.3475016 7.7584428
C -2.7832537 -3.2646958 8.8109097
C -3.9894436 -3.8304926 9.1877000
C -5.1628089 -3.4989983 8.5148996
C -5.1356088 -2.6003979 7.4619063

Ni -1.9402139 -1.3703547 5.3858736
C -0.2172421 -1.8352242 9.2156666
C 0.6364099 -2.8098059 9.7424918
C 0.7513860 -2.9588103 11.1181619
C 0.0442000 -2.1168027 11.9722104
C -0.7843475 -1.1269171 11.4496853
C -0.9267506 -0.9927469 10.0761289
C -5.9694343 -0.2136173 5.9135368
C -6.0350148 0.4498598 7.1419332
C -7.2672217 0.7032269 7.7271102
C -8.4369201 0.2742306 7.1048384
C -8.3753815 -0.3881604 5.8817144
C -7.1470483 -0.6178919 5.2764648
H -0.8858758 -3.5269096 -0.0607674
H 1.1955272 -4.1121753 1.1339979
H 1.5925702 -3.3762392 3.4394381
H -2.6376443 -2.1865217 1.0110009
H -6.0199483 1.4755893 4.1769559
H -5.7646423 2.5023028 1.9766415
H -3.9217792 1.7742109 0.4717386
H -2.3340605 0.0760285 1.2329033
H 2.1869739 -1.1944898 3.8290361
H 4.1513458 -0.4940085 5.1076651
H 3.9888907 -0.2397842 7.5774339
H 1.8933513 -0.7511855 8.7208643
H -1.8812264 -3.5643077 9.3161470
H -4.0088887 -4.5522629 9.9932263
H -6.0999897 -3.9614429 8.7943129
H -6.0459209 -2.3884484 6.9279995
H 1.1930457 -3.4590895 9.0783295
H 1.3979854 -3.7259595 11.5234758
H 0.1451583 -2.2264183 13.0440806
H -1.3190278 -0.4604117 12.1136688
H -1.5806685 -0.2319955 9.6687811
H -5.1241171 0.7713585 7.6310196
H -7.3167827 1.2347999 8.6683678
H -9.3961646 0.4634056 7.5687240
H -9.2840817 -0.7187886 5.3960991
H -7.0996846 -1.1256225 4.3211774

NiPh$_2$PhenTAA_2eOX_Triplet:
69 atoms
C -0.6784444 -3.1645859 0.8694670
C 0.4957088 -3.4943060 1.5434223
C 0.7155517 -3.0531204 2.8457543
C -0.2465680 -2.2619015 3.4706317
C -1.4459527 -1.9247890 2.7819823
C -1.6563980 -2.3868808 1.4841454
N -2.3555557 -1.2272043 3.5745353
N -0.2325448 -1.8241824 4.7936514
C 0.8594858 -1.4696839 5.5127990
C -3.2587071 -0.3119192 3.1478590
C -4.3324957 0.1007698 4.0194301
C -5.1636124 1.1299952 3.5750399
C -4.9944473 1.7429593 2.3363260
C -3.9640997 1.3334236 1.4911105
C -3.1110210 0.3278339 1.8896069
C 2.0782112 -1.1298031 4.8698029
C 3.1621317 -0.6684426 5.5838934
C 3.0690081 -0.5235033 6.9673621
C 1.8859588 -0.8527818 7.6236198
C 0.7643566 -1.3332691 6.9463584
C -4.6327358 -0.5254501 5.3196990
C -0.4195063 -1.7122280 7.7389633
N -1.5814416 -1.8884939 7.1726113
N -3.7542989 -1.2764407 5.9249522
C -3.9544949 -2.0785891 7.0875867
C -2.7655734 -2.4138799 7.7699161
C -2.8103047 -3.3072271 8.8396699
C -4.0231263 -3.8525816 9.2335603
C -5.1950816 -3.5217097 8.5613607
C -5.1648920 -2.6427352 7.4889757
Ni -1.9776747 -1.5360842 5.3694905
C -0.2466175 -1.8377833 9.2077613
C 0.6112705 -2.7997002 9.7493734
C 0.7301196 -2.9233874 11.1274636
C 0.0205572 -2.0700055 11.9678958
C -0.8137839 -1.0939040 11.4295937
C -0.9582731 -0.9836791 10.0537315
C -5.9516931 -0.2292630 5.9325859
C -5.9992246 0.4398337 7.1579764
C -7.2242862 0.7161010 7.7486679
C -8.4041744 0.3061868 7.1333905
C -8.3599901 -0.3615528 5.9126100
C -7.1384820 -0.6161816 5.3029652
H -0.8489490 -3.5402299 -0.1305845
H 1.2327409 -4.1247360 1.0642509
H 1.5989393 -3.3666360 3.3834210
H -2.5907476 -2.1898677 0.9783626
H -5.9628025 1.4725736 4.2140256
H -5.6556807 2.5473070 2.0433089
H -3.8098419 1.8236298 0.5389218
H -2.2659592 0.0724964 1.2686177
H 2.1277343 -1.1607850 3.7919998
H 4.0689614 -0.3886849 5.0641204
H 3.9061992 -0.1402069 7.5350579
H 1.8373834 -0.7220436 8.6936378
H -1.9077679 -3.6036533 9.3466686
H -4.0466395 -4.5561184 10.0548939
H -6.1360638 -3.9661543 8.8565795
H -6.0754761 -2.4276054 6.9560543
H 1.1688596 -3.4588064 9.0956732
H 1.3810156 -3.6803146 11.5448416
H 0.1237690 -2.1604793 13.0412890
H -1.3515626 -0.4192744 12.0826821
H -1.6167248 -0.2333300 9.6345033
H -5.0806019 0.7468168 7.6419273
H -7.2598596 1.2506542 8.6887999
H -9.3576973 0.5137989 7.6010644
H -9.2765436 -0.6778616 5.4323389
H -7.1045431 -1.1293426 4.3499449

NiPh$_2$PhenTAA_2eOX_Quintet:
69 atoms
C -0.6005115 -3.0656795 0.7815384
C 0.5725410 -3.3959162 1.4550408
C 0.7771513 -2.9743923 2.7651476
C -0.1855261 -2.1961580 3.4125281
C -1.3984629 -1.8547804 2.7161640
C -1.5857118 -2.3092575 1.4085525
N -2.3513774 -1.1995597 3.4838240
N -0.1494561 -1.8191710 4.7479048
C 0.9241474 -1.5122688 5.5074270
C -3.2983491 -0.3239833 3.0834829
C -4.3934786 0.0414876 3.9729245
C -5.2537811 1.0453093 3.5228947
C -5.1112594 1.6761192 2.2885092
C -4.0659029 1.3192934 1.4390622
C -3.1791548 0.3436857 1.8322582
C 2.1605834 -1.1589921 4.8975399
C 3.2430278 -0.7375442 5.6347185
C 3.1321114 -0.6437244 7.0205480
C 1.9339387 -0.9775386 7.6489387
C 0.8050041 -1.4215341 6.9570674
C -4.7024686 -0.5482098 5.3059190
C -0.3871961 -1.7628581 7.7830409
N -1.5705341 -1.8958054 7.2529101
N -3.8269730 -1.2608445 5.9575577
C -3.9832120 -2.0103831 7.1546039
C -2.7800102 -2.3490909 7.8452495
C -2.8471772 -3.2122274 8.9429185
C -4.0616305 -3.7262917 9.3646371
C -5.2339141 -3.3962241 8.6918109
C -5.1945289 -2.5513510 7.5956231
Ni -1.9743068 -1.5083462 5.3773975
C -0.1811872 -1.8832807 9.2504156
C 0.6242816 -2.8960136 9.7796202
C 0.7572379 -3.0269065 11.1560339
C 0.1140781 -2.1324500 12.0070702
C -0.6683438 -1.1079649 11.4809328
C -0.8264561 -0.9886541 10.1070027
C -6.0326553 -0.2356161 5.8916520
C -6.1017704 0.4976864 7.0782577
C -7.3352388 0.7709318 7.6534386
C -8.5021586 0.2951329 7.0613889
C -8.4365964 -0.4376378 5.8795106
C -7.2070415 -0.6911220 5.2851426
H -0.7634958 -3.4217068 -0.2269427
H 1.3205738 -4.0084308 0.9696351
H 1.6595557 -3.2941623 3.3010379
H -2.5204747 -2.1174954 0.9011169
H -6.0648457 1.3614811 4.1595940
H -5.8062521 2.4554810 2.0059217
H -3.9300608 1.8243155 0.4918311
H -2.3222483 0.1260969 1.2126157
H 2.2189506 -1.1518273 3.8195181
H 4.1597376 -0.4522381 5.1357302
H 3.9655040 -0.2944505 7.6153021
H 1.8818317 -0.8750227 8.7213061
H -1.9468177 -3.5077908 9.4541638
H -4.0888180 -4.4005534 10.2101645
H -6.1810352 -3.8114723 9.0093407

H -6.1071973 -2.3364839
7.0662363
H 1.1312361 -3.5873832
9.1177937
H 1.3676085 -3.8220715
11.5635829
H 0.2283175 -2.2292863
13.0787881
H -1.1555334 -0.4029999
12.1417399
H -1.4469631 -0.2009122
9.6981365
H -5.1930748 0.8551029
7.5465304
H -7.3870714 1.3540922
8.5635444
H -9.4620016 0.5013134
7.5165805
H -9.3429422 -0.8059336
5.4170420
H -7.1572147 -1.2545302
4.3614848

$NiPh_2$PhenTAA_3eOX_Doublet:
69 atoms
C -0.7893738 -3.2080631
0.9920435
C 0.4242431 -3.5494191
1.6889532
C 0.6744397 -3.1073210
2.9498946
C -0.2876632 -2.2820803
3.6011449
C -1.5434222 -1.9287634
2.8799755
C -1.7457192 -2.4265294
1.5600585
N -2.3413601 -1.1371297
3.5820996
N -0.2091419 -1.7371769
4.8066497
C 0.9302105 -1.4834067
5.5610445
C -3.3332039 -0.2840019
3.1131336
C -4.3974753 0.1393921
3.9803063
C -5.2494230 1.1572711
3.5089077
C -5.1185288 1.7089678
2.2433073
C -4.0991620 1.2708658
1.4071984
C -3.2100180 0.2981082
1.8467265
C 2.1428378 -1.2070320
4.9200268
C 3.2628877 -0.7992563
5.6337601
C 3.1845547 -0.6266943
7.0099487
C 1.9878220 -0.8792969
7.6637668
C 0.8394309 -1.3344100
6.9868016
C -4.6611669 -0.4170573
5.3035551
C -0.3506774 -1.6307724
7.7781528
N -1.5476320 -1.7164948
7.1923113
N -3.7158949 -1.1063648
5.9474221
C -3.9038702 -1.9968527
6.9938825
C -2.6773042 -2.3424427
7.6975792
C -2.7104376 -3.3356908
8.6906039
C -3.8989797 -3.9557170
8.9941913
C -5.0924298 -3.6191726
8.3098980
C -5.0963253 -2.6630674
7.3224168
Ni -1.9191778 -1.2701826
5.3977020
C -0.2285688 -1.7695426
9.2304190
C 0.7301128 -2.6320743
9.7984314
C 0.7966999 -2.7811915
11.1730353
C -0.0481627 -2.0365150
11.9979796
C -0.9794908 -1.1532331
11.4440962
C -1.0876763 -1.0347425
10.0719986
C -5.9509785 -0.1573568
5.9457788
C -5.9814539 0.3450255
7.2621507
C -7.1918988 0.5982622
7.8779088
C -8.3877278 0.3135196
7.2121495
C -8.3687550 -0.1995636
5.9139867
C -7.1614759 -0.4097132
5.2700792
H -0.9551807 -3.6312234
0.0093308
H 1.1259376 -4.2165690
1.2044009
H 1.5564184 -3.4279627
3.4854124
H -2.6802963 -2.2362750
1.0523299
H -6.0142645 1.5476376
4.1619822
H -5.7890316 2.4964552
1.9264501
H -3.9643588 1.7127938
0.4282356
H -2.3657030 0.0434698
1.2231303
H 2.1946700 -1.2382760
3.8415759
H 4.1804310 -0.5768230
5.1041269
H 4.0387973 -0.2682016
7.5684211
H 1.9327505 -0.6890016
8.7242625
H -1.8052802 -3.6414547
9.1887226
H -3.9177565 -4.7415273
9.7382335
H -6.0051340 -4.1529300
8.5414017
H -6.0047910 -2.4577377
6.7806841
H 1.3827771 -3.2114605
9.1586737
H 1.5087149 -3.4709570
11.6067649
H 0.0245386 -2.1370700
13.0733848
H -1.6082461 -0.5559180
12.0910789
H -1.8002928 -0.3428642
9.6415939
H -5.0546635 0.5755222
7.7718624
H -7.2156955 1.0258837
8.8715515
H -9.3344012 0.5004377
7.7028433
H -9.2971516 -0.4277118
5.4071274
H -7.1464515 -0.8103205
4.2649416

$NiPh_2$PhenTAA_3eOX_Quartet:
69 atoms
C -0.7336075 -3.2318720
0.9213448
C 0.4477921 -3.5644150
1.5993844
C 0.6851431 -3.0967286
2.8980817
C -0.2655032 -2.2677737
3.5098570
C -1.4727409 -1.9277960
2.8168835
C -1.7045925 -2.4239366
1.5264043
N -2.3559693 -1.1839048
3.5978446
N -0.2339647 -1.7817865
4.8160807
C 0.8850396 -1.4710730
5.5380185
C -3.2902104 -0.2957664
3.1413193
C -4.3757638 0.1352704
4.0017498
C -5.2172813 1.1648052
3.5274467
C -5.0674662 1.7248940
2.2587935
C -4.0315283 1.2850216
1.4234669
C -3.1511561 0.3043156
1.8624386
C 2.1083585 -1.1752134
4.8815741
C 3.2272254 -0.7564547
5.5902681
C 3.1571079 -0.5889302
6.9800193
C 1.9626571 -0.8560937
7.6489977
C 0.8117335 -1.3252874
6.9794673
C -4.6529546 -0.4416127
5.3081072
C -0.3670663 -1.6490163
7.7682148
N -1.5661105 -1.7758467
7.1771633
N -3.7188965 -1.1693315
5.9414396
C -3.9266938 -2.0418996
7.0181187
C -2.7051337 -2.3862195
7.7190990
C -2.7371570 -3.3494117
8.7420424
C -3.9442531 -3.9468811
9.0754052
C -5.1359561 -3.6108308
8.3917361
C -5.1320290 -2.6741909
7.3680532
Ni -1.9523828 -1.4095805
5.3880276
C -0.2453761 -1.7759831
9.2273642
C 0.7278090 -2.6315577
9.7986006
C 0.8019523 -2.7694859
11.1801990
C -0.0524880 -2.0237284
12.0070555
C -0.9997338 -1.1490655
11.4473867
C -1.1132001 -1.0385279
10.0688078
C -5.9436833 -0.1705897
5.9566792
C -5.9702141 0.3299093
7.2809521
C -7.1853684 0.5931939
7.8970942
C -8.3887357 0.3239206
7.2225837
C -8.3718141 -0.1859840
5.9150786
C -7.1597464 -0.4100035
5.2716096
H -0.9183952 -3.6349687 -
0.0745973
H 1.1758519 -4.2244196
1.1273402
H 1.5712630 -3.4208164
3.4423636
H -2.6495386 -2.2325912
1.0196230
H -5.9932794 1.5563940
4.1824351
H -5.7332846 2.5254397
1.9369774
H -3.8812432 1.7440267
0.4456168
H -2.2903163 0.0469604
1.2480151
H 2.1447486 -1.2003692
3.7939350
H 4.1441402 -0.5122490
5.0526002
H 4.0185313 -0.2177640
7.5349591
H 1.9112118 -0.6688160
8.7198997
H -1.8225817 -3.6568019
9.2421767
H -3.9664023 -4.7183579
9.8455081
H -6.0628286 -4.1271615
8.6428900
H -6.0465064 -2.4660261
6.8189659
H 1.3830675 -3.2165500
9.1524746
H 1.5258762 -3.4558576
11.6197840
H 0.0252412 -2.1168162
13.0908927
H -1.6354177 -0.5481538
12.0977653
H -1.8368147 -0.3508290
9.6298881
H -5.0338934 0.5502771
7.7947468
H -7.2082916 1.0214769
8.8992132
H -9.3417163 0.5209216
7.7149737
H -9.3082821 -0.4049884
5.4017967
H -7.1421808 -0.8155118
4.2594969

$NiPh_2$PhenTAA_3eOX_Sextet:
69 atoms
C -0.7095584 -3.1962305
0.9069114
C 0.4641136 -3.5264868
1.5806991
C 0.7015302 -3.0440012
2.8661850
C -0.2227655 -2.1964797
3.4852272
C -1.4422839 -1.8533152
2.7850993
C -1.6641890 -2.3783007
1.5080162
N -2.3587269 -1.1237172
3.5200663
N -0.1456252 -1.7464381
4.7905603
C 0.9529055 -1.5013350
5.5466112
C -3.3381842 -0.2938859
3.0835159
C -4.4300107 0.1073829
3.9690339
C -5.2845898 1.1154606
3.4932231
C -5.1668192 1.6801905
2.2262559
C -4.1464075 1.2645900
1.3753263
C -3.2456902 0.3085869
1.8038929
C 2.1973463 -1.2223239
4.9282063

C 3.3066377 -0.8318631
5.6533500
C 3.2078123 -0.6765387
7.0332323
C 1.9993803 -0.9350610
7.6741528
C 0.8500545 -1.3790037
6.9996849
C -4.7249040 -0.4295026
5.3074194
C -0.3314340 -1.6666918
7.8291077
N -1.5475958 -1.7223838
7.2849875
N -3.7933731 -1.0901350
5.9959069
C -3.9463511 -1.9353706
7.0794150
C -2.7041233 -2.2852065
7.7923283
C -2.7579619 -3.2636980
8.8025486
C -3.9493968 -3.8639419
9.1274008
C -5.1426106 -3.5277726
8.4427525
C -5.1381015 -2.5932282
7.4367672
Ni -1.9262391 -1.2625507
5.4140986
C -0.1598087 -1.8088454
9.2787599
C 0.7841273 -2.7101543
9.8106193
C 0.8843341 -2.8775981
11.1812694
C 0.0899970 -2.1135447
12.0376860
C -0.8261043 -1.1929529
11.5192701
C -0.9686685 -1.0555829
10.1519322
C -6.0349365 -0.1542213
5.9067231
C -6.1057529 0.3907300
7.2036715
C -7.3346391 0.6395151
7.7839640
C -8.5097116 0.3083253
7.1020105
C -8.4508240 -0.2481392
5.8233000
C -7.2249452 -0.4541971
5.2137468
H -0.8983557 -3.5991744 -
0.0795082
H 1.1863268 -4.1857782
1.1172878
H 1.5811810 -3.3689176
3.4037871
H -2.6012939 -2.1920999
1.0025638
H -6.0579121 1.4963443
4.1415226
H -5.8535027 2.4588881
1.9218791
H -4.0282781 1.7158401
0.3984904
H -2.3965630 0.0706338
1.1804847
H 2.2535769 -1.2365940
3.8497730
H 4.2331633 -0.6073227
5.1405954
H 4.0552677 -0.3296453
7.6094707
H 1.9481422 -0.7579848
8.7368925
H -1.8546126 -3.5696779
9.3041117
H -3.9727607 -4.6299881
9.8916734
H -6.0618950 -4.0413809
8.6930266
H -6.0484402 -2.3884196
6.8976988
H 1.3985482 -3.3037283
9.1462830
H 1.5836980 -3.5967046
11.5870368
H 0.1894549 -2.2283065
13.1095087
H -1.4167055 -0.5829469
12.1899721
H -1.6729199 -0.3386591
9.7494646
H -5.1955423 0.6526973
7.7280066
H -7.3893452 1.0981602
8.7624368
H -9.4706905 0.4922232
7.5652452
H -9.3625631 -0.5131293
5.3042902
H -7.1788850 -0.8874122
4.2230704

M06-L/def2-TZVP:
NiH_2PhenTAA:
NiH_2PhenTAA_2eRED_CSS:
49 atoms
C -0.7746069 2.0195141
1.9477660
C 0.1646398 2.5140645
2.8490111
C 0.4265100 1.8221224
4.0225927
C -0.2286157 0.6212087
4.3313566
C -1.1977216 0.1117227
3.4018195
C -1.4447202 0.8376322
2.2279327
N -1.8943818 -0.9890974
3.8405528
N -0.1466494 -0.0713134
5.5161354
C 0.9275079 -0.0477571
6.3601627
C -2.5494048 -1.8706586
3.0276543
C -3.5822856 -2.7293599
3.5790771
C -4.2744278 -3.5757255
2.6707394
C -3.9618726 -3.6698837
1.3363428
C -2.8919102 -2.9170982
0.8310520
C -2.2098668 -2.0570497
1.6747063
C 2.2280943 0.2787793
5.9322353
C 3.3163005 0.3419906
6.7856361
C 3.1423030 0.0426913
8.1445449
C 1.9048097 -0.3564323
8.5878050
C 0.7666287 -0.4616175
7.7427402
C -3.9011400 -2.8223217
4.9402759
C -0.4061313 -1.0064056
8.2827455
N -1.5371537 -1.2632510
7.6073862
N -3.2830042 -2.1711276
5.9378915
C -3.6467695 -2.2583849
7.2455871
C -2.6713217 -1.7501742
8.1786030
C -2.9724730 -1.7765276
9.5492629
C -4.1711749 -2.2880164
10.0108492
C -5.1203283 -2.7849651
9.1023882
C -4.8577725 -2.7625438
7.7453037
Ni -1.6771076 -1.1868507
5.7198597
H -0.3430586 -1.3460575
9.3169156
H -4.6443248 -3.5741983
5.2070945
H -1.0044886 2.5634575
1.0378165
H 0.6767551 3.4498278
2.6520164
H 1.1166066 2.2386364
4.7441849
H -2.2124528 0.4854691
1.5519178
H -5.0757422 -4.1855365
3.0789351
H -4.5254950 -4.3343705
0.6897902
H -2.5849631 -3.0148026 -
0.2042362
H -1.3668101 -1.4994749
1.2882553
H 2.3745704 0.4802677
4.8792453
H 4.2936382 0.6048462
6.3962826
H 3.9757538 0.1054247
8.8362818
H 1.7701726 -0.6299915
9.6307851
H -2.2578921 -1.3633155
10.2514122
H -4.3861316 -2.2831844
11.0738590
H -6.0697605 -3.1667439
9.4620383
H -5.6073312 -3.1174177
7.0473896

NiH_2PhenTAA_2eRED_Triplet:
49 atoms
C -0.7015067 1.9763878
1.8999073
C 0.2343908 2.4685486
2.7974807
C 0.4814880 1.7849005
3.9838571
C -0.1923333 0.5996302
4.3018116
C -1.1587306 0.0919639
3.3751673
C -1.3887788 0.8022379
2.1911150
N -1.8666487 -1.0027653
3.8293123
N -0.1283878 -0.0893516
5.4964127
C 0.9329110 -0.0434545
6.3600453
C -2.5511857 -1.8698629
3.0205338
C -3.6069649 -2.6908297
3.5777918
C -4.3392390 -3.5139350
2.6834818
C -4.0444388 -3.6214383
1.3418932
C -2.9627656 -2.9026969
0.8276319
C -2.2411905 -2.0583432
1.6629584
C 2.2363107 0.2952401
5.9578659
C 3.3063420 0.3846352
6.8401740
C 3.1068761 0.1135394
8.1957471
C 1.8575570 -0.2844394
8.6190219
C 0.7461675 -0.4186688
7.7469676
C -3.9369052 -2.7458646
4.9425378
C -0.4496472 -0.9288289
8.2801615
N -1.5465583 -1.2653658
7.5823132
N -3.2587900 -2.1607835
5.9432213
C -3.6368483 -2.2792725
7.2597260
C -2.6741238 -1.7739957
8.1815890
C -2.9392672 -1.8394673
9.5549585
C -4.1334579 -2.4021456
10.0199054
C -5.0682947 -2.8957185
9.1241087
C -4.8266521 -2.8343997
7.7468408
Ni -1.6688152 -1.1803863
5.7075386
H -0.4317382 -1.1713147
9.3418434
H -4.7542816 -3.4146331
5.2085929
H -0.9142445 2.5062344
0.9774695
H 0.7660995 3.3911487
2.5900296
H 1.1731580 2.2026924
4.7022703
H -2.1547540 0.4526978
1.5127255
H -5.1549526 -4.0954899
3.1034852
H -4.6352510 -4.2691831
0.7029879
H -2.6753653 -3.0026695 -
0.2133728
H -1.3900495 -1.5255825
1.2611409
H 2.4090203 0.4801639
4.9064842
H 4.2884150 0.6555020
6.4677575
H 3.9234684 0.1970422
8.9048340
H 1.6969430 -0.5331221
9.6642302
H -2.2257951 -1.4337341
10.2601681
H -4.3266810 -2.4417725
11.0867110
H -5.9963935 -3.3255389
9.4860902
H -5.5731103 -3.2015105
7.0544746

NiH_2PhenTAA_2eRED_Quintet:
49 atoms
C -0.5128925 1.8398303
1.7957068
C 0.3816831 2.3612039
2.7148775
C 0.5849659 1.6965521
3.9215064
C -0.0738901 0.5051198
4.2398250
C -1.0149068 -0.0398695
3.2824887
C -1.1978812 0.6636378
2.0809890
N -1.7454194 -1.1189633
3.7127647
N -0.0306823 -0.1334857
5.4678796
C 0.9745802 0.0141061
6.4012941
C -2.5751728 -1.8874423
2.9371951
C -3.7011927 -2.5839521
3.5478375
C -4.5587909 -3.3039768
2.6735862
C -4.3321432 -3.4487380
1.3214812

C -3.1942448 -2.8683965 0.7619553
C -2.3520439 -2.1092092 1.5682580
C 2.2824832 0.3793248 6.0531031
C 3.3030760 0.5686602 6.9823829
C 3.0263131 0.3990030 8.3369535
C 1.7684988 -0.0215421 8.7161720
C 0.7180353 -0.2914849 7.8007507
C -4.0254523 -2.6448337 4.9251370
C -0.4661978 -0.8464264 8.3453061
N -1.4931238 -1.3521667 7.6515378
N -3.2921783 -2.1830912 5.9526902
C -3.6075239 -2.3659230 7.2739660
C -2.6160051 -1.9007312 8.2133663
C -2.8658792 -2.0502834 9.5844603
C -4.0439041 -2.6427584 10.0470191
C -4.9946771 -3.0932814 9.1442154
C -4.7767440 -2.9587210 7.7702209
Ni -1.5456132 -1.3184472 5.6763603
H -0.4837972 -0.9043784 9.4359110
H -4.9342346 -3.2078909 5.1489945
H -0.6976325 2.3486545 0.8549109
H 0.9123379 3.2853225 2.5122652
H 1.2335384 2.1415848 4.6633867
H -1.9423160 0.3108703 1.3814818
H -5.4229658 -3.7853067 3.1234522
H -5.0231857 -4.0209668 0.7115116
H -2.9588361 -3.0041678 -0.2880919
H -1.4578535 -1.6823394 1.1340570
H 2.5041505 0.5057109 5.0021320
H 4.2942573 0.8521513 6.6461434
H 3.7897367 0.5750169 9.0872224
H 1.5593133 -0.2001443 9.7671025
H -2.1302378 -1.7007466 10.2982604
H -4.2124014 -2.7426026 11.1142153
H -5.9137724 -3.5476835 9.4997294
H -5.5341771 -3.3007983 7.0760291

NiH$_2$PhenTAA_1eRED_Doublet:
49 atoms
C -0.7274848 1.9946887 1.9151847
C 0.2117696 2.4862559 2.8130703
C 0.4568708 1.8111051 3.9975426
C -0.2143528 0.6230350 4.3055611
C -1.1759078 0.1192546 3.3850706
C -1.4184850 0.8296850 2.2050419
N -1.8929147 -0.9715434 3.8442650
N -0.1575658 -0.0610543 5.5070431
C 0.9068687 -0.0445333 6.3515437
C -2.5460909 -1.8530635 3.0425624
C -3.5754267 -2.7045027 3.5849834
C -4.2779992 -3.5570771 2.7023183
C -3.9791864 -3.6553817 1.3678247
C -2.9125997 -2.9010414 0.8620333
C -2.2224362 -2.0352672 1.6793071
C 2.2141486 0.2918009 5.9334466
C 3.2811559 0.3419254 6.8011365
C 3.1058872 0.0447148 8.1587502
C 1.8659868 -0.3520930 8.5889258
C 0.7484558 -0.4454023 7.7274061
C -3.9073328 -2.7751627 4.9510050
C -0.4422521 -0.9645731 8.2698600
N -1.5428184 -1.2522803 7.5901419
N -3.2701106 -2.1580978 5.9354735
C -3.6374012 -2.2724785 7.2599462
C -2.6781195 -1.7673908 8.1793036
C -2.9558162 -1.8109852 9.5483599
C -4.1427481 -2.3497898 10.0083804
C -5.0835317 -2.8488908 9.1058152
C -4.8342246 -2.8054355 7.7468956
Ni -1.6913894 -1.1476807 5.7135814
H -0.4124471 -1.2155798 9.3290898
H -4.7140994 -3.4593564 5.2098261
H -0.9473319 2.5330358 1.0014530
H 0.7326655 3.4134939 2.6082024
H 1.1386196 2.2353267 4.7211622
H -2.1957893 0.4891293 1.5355268
H -5.0713654 -4.1643082 3.1254578
H -4.5376471 -4.3246021 0.7261781
H -2.6121666 -3.0071912 -0.1735504
H -1.3814814 -1.4874557 1.2788773
H 2.3780743 0.4897145 4.8839973
H 4.2633434 0.5969992 6.4210243
H 3.9376698 0.0987004 8.8490824
H 1.7165204 -0.6301135 9.6270508
H -2.2450772 -1.3992512 10.2528159
H -4.3482734 -2.3667747 11.0712670
H -6.0196587 -3.2557324 9.4672886
H -5.5815045 -3.1687752 7.0534706

NiH$_2$PhenTAA_1eRED_Quartet:
49 atoms
C -0.5793916 1.8608187 1.8022040
C 0.3851859 2.3370667 2.6778139
C 0.6278334 1.6752932 3.8703027
C -0.0618273 0.5076406 4.2244349
C -1.0609583 0.0134849 3.3119131
C -1.2977187 0.7224023 2.1297544
N -1.8248883 -1.0453158 3.7733487
N -0.0012371 -0.1277414 5.4454298
C 0.9808358 0.0086357 6.3722473
C -2.5999380 -1.8565859 2.9817965
C -3.6996843 -2.5996633 3.5557489
C -4.5190767 -3.3379126 2.6736031
C -4.2572529 -3.4664894 1.3289159
C -3.1232562 -2.8503206 0.8034636
C -2.3308938 -2.0613992 1.6169449
C 2.3060128 0.3772734 6.0441797
C 3.2984214 0.5316004 6.9864628
C 3.0203611 0.3304862 8.3415523
C 1.7620482 -0.0876531 8.6965400
C 0.7198052 -0.3028606 7.7647903
C -4.0391747 -2.6797274 4.9342651
C -0.4782699 -0.8384782 8.3088571
N -1.5356482 -1.2633131 7.6409642
N -3.2824560 -2.2746231 5.9421498
C -3.6001237 -2.3960960 7.2699697
C -2.6440723 -1.8558072 8.1996452
C -2.9019385 -1.9673049 9.5709381
C -4.0481877 -2.5755696 10.0398957
C -4.9778164 -3.1027575 9.1379223
C -4.7538055 -3.0103044 7.7810721
Ni -1.5888951 -1.2845153 5.6621606
H -0.4633339 -0.9504819 9.3956122
H -4.9934692 -3.1663968 5.1434279
H -0.7980109 2.3876063 0.8816134
H 0.9314912 3.2448549 2.4512042
H 1.3226265 2.1073074 4.5755291
H -2.1019807 0.4007462 1.4834817
H -5.3717891 -3.8520424 3.1048979
H -4.9080574 -4.0607784 0.6999769
H -2.8540892 -2.9861025 -0.2368560
H -1.4452527 -1.6002688 1.2028353
H 2.5505821 0.4989987 4.9987894
H 4.2997200 0.7961250 6.6670316
H 3.7856879 0.4718119 9.0934185
H 1.5402486 -0.2932126 9.7388210
H -2.1946549 -1.5518350 10.2774317
H -4.2285436 -2.6376781 11.1057565
H -5.8783294 -3.5807021 9.5034645
H -5.4818379 -3.4222149 7.0942775

NiH$_2$PhenTAA_1eRED_Sextet:
49 atoms
C -0.5975629 1.8420052 1.8547076
C 0.3548330 2.3317708 2.7569753
C 0.6095312 1.6645546 3.9321583
C -0.0642256 0.4675807 4.2645984
C -1.0621745 -0.0432598 3.3204047
C -1.2923662 0.6888736 2.1344267
N -1.7435258 -1.1367264 3.7208503
N 0.0495844 -0.2143284 5.4235591
C 1.0301822 -0.0289575 6.3870112
C -2.5761525 -1.9140520 2.9338511
C -3.7008074 -2.5976372 3.5411967
C -4.5585286 -3.2926245 2.6485987
C -4.3032181 -3.4292317 1.3044881
C -3.1510325 -2.8672091 0.7551067
C -2.3188783 -2.1186880 1.5759908
C 2.3366199 0.3219267 6.0386175
C 3.3356697 0.5422183 6.9769466
C 3.0215999 0.4149238 8.3294357
C 1.7612581 0.0125532 8.7043332
C 0.7262151 -0.2861869 7.7800270
C -4.0354578 -2.6596686 4.9150290
C -0.4699226 -0.8153523 8.3249668
N -1.5041516 -1.3219556 7.6372882
N -3.2777786 -2.2364910 5.9405513
C -3.5979494 -2.3863922 7.2672486
C -2.6227039 -1.8831867 8.1994946
C -2.8778134 -2.0030118 9.5701020
C -4.0482001 -2.5946252 10.0324279
C -4.9869700 -3.0825005 9.1337542
C -4.7624626 -2.9808379 7.7649885
Ni -1.5627751 -1.3292007 5.6796648

H -0.4937672 -0.8605241
9.4128648
H -4.9778398 -3.1595494
5.1339517
H -0.8134909 2.3892048
0.9452165
H 0.8720050 3.2596822
2.5459332
H 1.2967633 2.0874103
4.6501123
H -2.0722268 0.3541338
1.4659475
H -5.4375907 -3.7639020
3.0748003
H -4.9883798 -3.9932390
0.6827901
H -2.9049074 -3.0055046 -
0.2894156
H -1.4157229 -1.6869093
1.1647881
H 2.5728836 0.4001505
4.9856811
H 4.3344921 0.8083848
6.6569118
H 3.7711069 0.6074879
9.0874612
H 1.5363400 -0.1236577
9.7565553
H -2.1565291 -1.6213961
10.2804479
H -4.2258758 -2.6686401
11.0984136
H -5.8998347 -3.5405476
9.4946097
H -5.5032623 -3.3608674
7.0741310

NiH_2PhenTAA_0eNeutral_CSS:
49 atoms
C -0.6730052 1.9540673
1.8812045
C 0.2623764 2.4464334
2.7780566
C 0.4950167 1.7849883
3.9723207
C -0.1828463 0.6039913
4.2745724
C -1.1356423 0.1028188
3.3607732
C -1.3780167 0.7995524
2.1772550
N -1.8668830 -0.9835042
3.8392408
N -0.1456237 -0.0789595
5.4897427
C 0.8984733 -0.0462042
6.3452452
C -2.5393506 -1.8483709
3.0498888
C -3.5850998 -2.6724464
3.5883300
C -4.3276022 -3.5075923
2.7250522
C -4.0423980 -3.6166768
1.3931060
C -2.9592081 -2.8863263
0.8835106
C -2.2410437 -2.0303986
1.6758064
C 2.2114240 0.3140162
5.9485506
C 3.2498338 0.3730376
6.8397392
C 3.0609071 0.0914761
8.2003377
C 1.8211203 -0.3091477
8.6121275
C 0.7318086 -0.4242139
7.7208509
C -3.9208074 -2.7128286
4.9544205
C -0.4721319 -0.9225735
8.2524780
N -1.5301686 -1.2638297
7.5659713
N -3.2438670 -2.1597610
5.9254970
C -3.6223262 -2.2937717
7.2701223
C -2.6751882 -1.7960402
8.1772707
C -2.9239929 -1.8652355
9.5431366
C -4.1015918 -2.4298693
10.0030919
C -5.0366697 -2.9264433
9.1059729
C -4.8008877 -2.8592015
7.7431917
Ni -1.6876010 -1.1328744
5.7015521
H -0.4869194 -1.0672100
9.3308724
H -4.8037538 -3.2964728
5.2068670
H -0.8859333 2.4876496
0.9645101
H 0.7863773 3.3694989
2.5688967
H 1.1652007 2.2200413
4.6989752
H -2.1621617 0.4685766
1.5122945
H -5.1307245 -4.0908879
3.1603017
H -4.6155863 -4.2735532
0.7546430
H -2.6681699 -3.0093150 -
0.1523928
H -1.3910527 -1.5099062
1.2611100
H 2.4002606 0.4998462
4.9023325
H 4.2380890 0.6263834
6.4760055
H 3.8831905 0.1594927
8.8979791
H 1.6501544 -0.5753099
9.6488155
H -2.2119446 -1.4650082
10.2510552
H -4.2951728 -2.4706434
11.0663211
H -5.9604060 -3.3571179
9.4679894
H -5.5454557 -3.2341761
7.0550076

NiH_2PhenTAA_0eNeutral_Triplet:
49 atoms
C -0.8161878 2.0210642
1.9787187
C 0.1327059 2.5184710
2.8854627
C 0.4161957 1.8394105
4.0441389
C -0.2298888 0.6234247
4.3387665
C -1.2062626 0.1110084
3.4038725
C -1.4773280 0.8459113
2.2340899
N -1.8405157 -1.0151872
3.8155511
N -0.1059664 -0.1050330
5.4763404
C 0.9508144 -0.0618509
6.3442762
C -2.5267032 -1.8845446
3.0118866
C -3.5399419 -2.7299910
3.5720633
C -4.2346479 -3.5951016
2.7059332
C -3.9430085 -3.6908683
1.3667325
C -2.8979988 -2.9283429
0.8443425
C -2.2062102 -2.0547922
1.6536651
C 2.2470663 0.2883387
5.9263858
C 3.3101621 0.3302466
6.8008408
C 3.1272960 0.0028113
8.1445276
C 1.8865344 -0.4077162
8.5680799
C 0.7769665 -0.4797425
7.7048810
C -3.8690216 -2.8108971
4.9534827
C -0.4206431 -1.0188605
8.2514727
N -1.5313210 -1.2461920
7.5933035
N -3.2775655 -2.1557201
5.9227857
C -3.6426491 -2.2454545
7.2536858
C -2.6844613 -1.7453837
8.1703804
C -2.9732971 -1.7528159
9.5373488
C -4.1731828 -2.2582769
9.9904842
C -5.1164755 -2.7528337
9.0875438
C -4.8581637 -2.7399763
7.7335638
Ni -1.6415329 -1.1998094
5.6901915
H -0.3618790 -1.3214990
9.2969063
H -4.6391576 -3.5386895
5.2084309
H -1.0575130 2.5874885
1.0890566
H 0.6172785 3.4655170
2.6887450
H 1.0984541 2.2630778
4.7670099
H -2.2535510 0.5025798
1.5653207
H -5.0162188 -4.2124285
3.1329670
H -4.4981799 -4.3698304
0.7343168
H -2.6167854 -3.0276234 -
0.1958340
H -1.3723492 -1.4979961
1.2504188
H 2.4088374 0.5017276
4.8793694
H 4.2904953 0.6067056
6.4351534
H 3.9556962 0.0437679
8.8381738
H 1.7404287 -0.7056894
9.5997128
H -2.2618812 -1.3379631
10.2387399
H -4.3918622 -2.2521102
11.0498108
H -6.0637656 -3.1297735
9.4493259
H -5.6078154 -3.0925575
7.0375775

NiH_2PhenTAA_0eNeutral_Quintet:
49 atoms
C -0.5990848 1.8550382
1.8483199
C 0.3609245 2.3399607
2.7500392
C 0.6061805 1.6823296
3.9270151
C -0.0796297 0.4941544
4.2604333
C -1.0802122 -0.0075261
3.3228310
C -1.3083863 0.7181816
2.1343347
N -1.7850012 -1.0866092
3.7343039
N 0.0202879 -0.1792903
5.4300515
C 1.0069172 -0.0216790
6.3776701
C -2.5845984 -1.8845916
2.9501356
C -3.6849046 -2.6014360
3.5427391
C -4.5124422 -3.3396477
2.6704691
C -4.2542589 -3.4726030
1.3268483
C -3.1236721 -2.8658338
0.7839887
C -2.3189414 -2.0839663
1.5869211
C 2.3179760 0.3318322
6.0241940
C 3.3136309 0.5143794
6.9618527
C 3.0226119 0.3556993
8.3145604
C 1.7630936 -0.0487435
8.6881662
C 0.7284826 -0.2991150
7.7624296
C -4.0307281 -2.6657743
4.9235102
C -0.4766621 -0.8235684
8.3179670
N -1.5027028 -1.3116343
7.6487401
N -3.2968771 -2.2369428
5.9362765
C -3.6020812 -2.3751846
7.2673612
C -2.6298053 -1.8734352
8.1967001
C -2.8859405 -1.9913319
9.5708064
C -4.0431829 -2.5733142
10.0283906
C -4.9911703 -3.0653585
9.1219364
C -4.7709806 -2.9682065
7.7696124
Ni -1.5897552 -1.2825381
5.6769006
H -0.4909435 -0.8602884
9.4068447
H -4.9709981 -3.1748152
5.1318055
H -0.8134560 2.4055661
0.9418122
H 0.8795633 3.2651815
2.5364959
H 1.2903826 2.1092359
4.6448963
H -2.0994585 0.3963231
1.4732545
H -5.3685194 -3.8444412
3.1025738
H -4.9095640 -4.0686114
0.7060343
H -2.8679056 -3.0089396 -
0.2571737
H -1.4267421 -1.6359735
1.1718828
H 2.5548449 0.4236283
4.9736109
H 4.3139506 0.7712023
6.6398864
H 3.7846862 0.5180992
9.0643594
H 1.5447922 -0.2168203
9.7361716
H -2.1646609 -1.6106324
10.2813386
H -4.2239894 -2.6473565
11.0923335
H -5.9024439 -3.5200368
9.4870363
H -5.5136257 -3.3485659
7.0813319

NiH$_2$PhenTAA_1eOX_Doublet:
49 atoms
C -0.7320987 1.9458373
1.9307578
C 0.2132995 2.4468164
2.8401316
C 0.4702998 1.7921322
4.0174928
C -0.2024030 0.5953028
4.3189506
C -1.1704392 0.0835606
3.3884411
C -1.4204335 0.7908283
2.1999686
N -1.8195207 -1.0320211
3.8234682
N -0.1051247 -0.1282558
5.4691645
C 0.9430808 -0.0631789
6.3456472
C -2.5251711 -1.8848330
3.0193885
C -3.5778748 -2.6755138
3.5656787
C -4.3294602 -3.4989283
2.7117103
C -4.0354587 -3.6160904
1.3746737
C -2.9501088 -2.9094210
0.8605775
C -2.2162566 -2.0613899
1.6599956
C 2.2400939 0.2961729
5.9403123
C 3.2804456 0.3694041
6.8396556
C 3.0789573 0.0949648
8.1908802
C 1.8315690 -0.3030772
8.6068949
C 0.7557565 -0.4242014
7.7121791
C -3.9190601 -2.7058409
4.9445846
C -0.4630837 -0.9217398
8.2460166
N -1.5085203 -1.2795733
7.5652506
N -3.2352348 -2.1789377
5.9135500
C -3.6156429 -2.2893075
7.2620359
C -2.6688185 -1.7934850
8.1680975
C -2.9234924 -1.8432397
9.5347464
C -4.1072118 -2.3893995
9.9896582
C -5.0450284 -2.8863001
9.0905638
C -4.8051065 -2.8369740
7.7317082
Ni -1.6450773 -1.1895874
5.6888656
H -0.4824526 -1.0441061
9.3267980
H -4.8182994 -3.2661579
5.1904235
H -0.9554595 2.5019984
1.0306871
H 0.7087169 3.3851638
2.6323480
H 1.1421268 2.2268590
4.7422336
H -2.1995845 0.4553049
1.5321159
H -5.1458414 -4.0676618
3.1391663
H -4.6187453 -4.2672610
0.7399173
H -2.6667253 -3.0325579 -
0.1762940
H -1.3565701 -1.5516095
1.2505965
H 2.4288716 0.4736064
4.8920607
H 4.2676924 0.6318196
6.4828604
H 3.8955540 0.1670378
8.8944575
H 1.6608683 -0.5549341
9.6462046
H -2.2130854 -1.4437157
10.2442319
H -4.3091572 -2.4178356
11.0512379
H -5.9742234 -3.3024278
9.4542217
H -5.5515618 -3.2112460
7.0456871

NiH$_2$PhenTAA_1eOX_Quartet:
49 atoms
C -0.6914205 1.9567519
1.8921752
C 0.2745921 2.4365860
2.7794167
C 0.5446611 1.7618898
3.9471258
C -0.1236155 0.5666153
4.2596718
C -1.1240823 0.0718764
3.3386752
C -1.3896783 0.8036287
2.1749827
N -1.8296378 -1.0260697
3.7629793
N -0.0272270 -0.1141995
5.4411978
C 0.9812773 -0.0223119
6.3517238
C -2.5640321 -1.8649211
2.9774636
C -3.6085357 -2.6654518
3.5577229
C -4.3674455 -3.4871287
2.7084244
C -4.1061366 -3.5992499
1.3633307
C -3.0335407 -2.8930570
0.8231114
C -2.2866158 -2.0463695
1.6090185
C 2.2942049 0.3343125
5.9849020
C 3.3068257 0.4273373
6.9104860
C 3.0604523 0.1672282
8.2572522
C 1.8047582 -0.2429599
8.6380784
C 0.7468747 -0.3836719
7.7246714
C -3.9452034 -2.7534717
4.9471937
C -0.4569737 -0.9312481
8.2747415
N -1.5269781 -1.2569822
7.6181227
N -3.3024221 -2.1975395
5.9251102
C -3.6319216 -2.3224590
7.2784120
C -2.6740389 -1.8169776
8.1909634
C -2.9341327 -1.8747538
9.5573327
C -4.1101374 -2.4311031
10.0193923
C -5.0493312 -2.9308432
9.1235311
C -4.8149357 -2.8723676
7.7641163
Ni -1.6372426 -1.1862557
5.6621642
H -0.4085480 -1.1293635
9.3470679
H -4.7995793 -3.3981801
5.1631269
H -0.9218735 2.5126263
0.9935763
H 0.7854037 3.3658110
2.5681905
H 1.2327785 2.1865432
4.6626586
H -2.1882078 0.4842654
1.5216833
H -5.1717424 -4.0635214
3.1488522
H -4.7063875 -4.2485425
0.7423008
H -2.7755151 -3.0138779 -
0.2205919
H -1.4351296 -1.5354024
1.1836375
H 2.5118918 0.4925517
4.9388559
H 4.3029087 0.6940895
6.5830054
H 3.8518312 0.2542755
8.9877766
H 1.6131892 -0.4908809
9.6748625
H -2.2229629 -1.4666457
10.2622851
H -4.3053509 -2.4654550
11.0821716
H -5.9733099 -3.3558963
9.4905001
H -5.5627575 -3.2432305
7.0765509

NiH$_2$PhenTAA_1eOX_Sextet:
49 atoms
C -0.6222603 1.8915272
1.8876595
C 0.3341648 2.3668055
2.7800300
C 0.5888040 1.6882474
3.9535882
C -0.0836999 0.4927782
4.2559224
C -1.0781306 0.0048582
3.3287257
C -1.3307205 0.7359893
2.1673334
N -1.8070767 -1.0794135
3.7599938
N 0.0133680 -0.1994650
5.4273516
C 1.0101463 -0.0449637
6.3639588
C -2.5695904 -1.8883156
2.9735734
C -3.6782080 -2.6234038
3.5535369
C -4.5051376 -3.3513260
2.6720774
C -4.2324688 -3.4705086
1.3335645
C -3.0901067 -2.8580625
0.8046064
C -2.2914181 -2.0796651
1.6065176
C 2.3174317 0.2854637
5.9980058
C 3.3237718 0.4682323
6.9361428
C 3.0306331 0.3327317
8.2855405
C 1.7619645 -0.0495150
8.6744235
C 0.7249877 -0.3065081
7.7539062
C -4.0264558 -2.7020681
4.9245953
C -0.4758305 -0.8142912
8.3055866
N -1.5241748 -1.3038093
7.6090730
N -3.2847467 -2.2764292
5.9342993
C -3.6047629 -2.4000118
7.2532165
C -2.6306265 -1.8485907
8.1762538
C -2.8989169 -1.9212111
9.5558775
C -4.0481697 -2.5114406
10.0093378
C -4.9879259 -3.0563491
9.1111805
C -4.7680233 -3.0002089
7.7608465
Ni -1.5677062 -1.3229581
5.6674733
H -0.4943573 -0.8788405
9.3901971
H -4.9692864 -3.2051135
5.1314421
H -0.8440392 2.4455338
0.9857350
H 0.8521253 3.2932396
2.5743767
H 1.2682405 2.1108690
4.6787113
H -2.1265810 0.4240965
1.5070677
H -5.3643249 -3.8590350
3.0920012
H -4.8780948 -4.0608247
0.6986174
H -2.8303801 -2.9987334 -
0.2356726
H -1.3997269 -1.6268756
1.1970469
H 2.5526711 0.3593253
4.9452805
H 4.3243210 0.7146053
6.6097702
H 3.7946516 0.4979443
9.0320141
H 1.5469169 -0.1898054
9.7263461
H -2.1981447 -1.5045083
10.2645146
H -4.2408261 -2.5595237
11.0724748
H -5.8883876 -3.5167766
9.4931240
H -5.4988925 -3.4176960
7.0827539

NiH$_2$PhenTAA_2eOX_CSS:
49 atoms
C -0.7781819 1.9386668
1.9663489
C 0.1819537 2.4463829
2.8882141
C 0.4518242 1.8048372
4.0558978
C -0.2241648 0.5976071
4.3639193
C -1.2131372 0.0752997
3.4140917
C -1.4632471 0.7919362
2.2173621
N -1.8015685 -1.0520858
3.8124480
N -0.0837539 -0.1468411
5.4605585
C 0.9699606 -0.0740462
6.3443089
C -2.5159247 -1.9054014
3.0008568
C -3.5616191 -2.6937952
3.5563522
C -4.3098815 -3.5103955
2.7030796
C -4.0065332 -3.6253219
1.3599437
C -2.9270481 -2.9204203
0.8408675
C -2.1976328 -2.0683439
1.6457773
C 2.2573335 0.2900076
5.9256876
C 3.3047012 0.3612847
6.8221286

C 3.0987088 0.0810562
8.1676258
C 1.8465124 -0.3186546
8.5934446
C 0.7742964 -0.4424644
7.7047101
C -3.9116892 -2.7179150
4.9419604
C -0.4538487 -0.9353182
8.2447033
N -1.4990690 -1.2921853
7.5705486
N -3.2332114 -2.1948611
5.9117531
C -3.6187278 -2.2817514
7.2593592
C -2.6705521 -1.7849164
8.1668339
C -2.9310180 -1.8150815
9.5336711
C -4.1225251 -2.3438584
9.9837497
C -5.0626200 -2.8427715
9.0823096
C -4.8178584 -2.8128655
7.7254665
Ni -1.6211390 -1.2282882
5.6852626
H -0.4727772 -1.0482345
9.3261987
H -4.8171154 -3.2700520
5.1828056
H -1.0067476 2.5145899
1.0795285
H 0.6571259 3.3948653
2.6772052
H 1.1186628 2.2444949
4.7825513
H -2.2480698 0.4618930
1.5531517
H -5.1316352 -4.0790981
3.1191043
H -4.5885642 -4.2800120
0.7267881
H -2.6443215 -3.0434490 -
0.1957852
H -1.3339742 -1.5587551
1.2427542
H 2.4409784 0.4627794
4.8750740
H 4.2913635 0.6255423
6.4663679
H 3.9159424 0.1482509
8.8717663
H 1.6855241 -0.5642839
9.6353235
H -2.2232553 -1.4142922
10.2449356
H -4.3339348 -2.3576499
11.0438355
H -5.9981764 -3.2432522
9.4474883
H -5.5663649 -3.1868322
7.0416655

NiH$_2$PhenTAA_2eOX_Triplet:
49 atoms
C -0.7813725 1.9983570
1.9643545
C 0.1565335 2.4927936
2.8637309
C 0.4278202 1.8174085
4.0437277
C -0.2221419 0.6150103
4.3170167
C -1.1816395 0.1099638
3.3969699
C -1.4625715 0.8211046
2.2317049
N -1.8472327 -1.0174014
3.8449357
N -0.1317726 -0.1161852
5.4887920
C 0.9339542 -0.0887172
6.3306901
C -2.5027964 -1.8909829
3.0367740
C -3.5395884 -2.7160458
3.5784694
C -4.2531142 -3.5520218
2.7174239
C -3.9332721 -3.6543033
1.3761492
C -2.8656794 -2.9169407
0.8672378
C -2.1680212 -2.0486063
1.6756323
C 2.2267231 0.2717217
5.8948267
C 3.2863238 0.3196357
6.7716577
C 3.1033106 0.0191342
8.1202331
C 1.8566150 -0.3800394
8.5651817
C 0.7663345 -0.4781166
7.6984959
C -3.9011648 -2.7384304
4.9562464
C -0.4576966 -0.9585540
8.2461632
N -1.5227709 -1.2677841
7.5756732
N -3.2515779 -2.1686010
5.9219875
C -3.6399860 -2.2623487
7.2732525
C -2.6938324 -1.7666683
8.1785602
C -2.9515228 -1.7953158
9.5432608
C -4.1461722 -2.3247240
9.9956015
C -5.0842468 -2.8212366
9.0967767
C -4.8382491 -2.7910390
7.7363711
Ni -1.6841687 -1.1463658
5.7057394
H -0.4632749 -1.1105721
9.3229292
H -4.7809835 -3.3281443
5.2027706
H -1.0147538 2.5545270
1.0669562
H 0.6505302 3.4336340
2.6642645
H 1.0951834 2.2541044
4.7722060
H -2.2489223 0.4897861
1.5693657
H -5.0629556 -4.1446100
3.1231793
H -4.4901650 -4.3237167
0.7356625
H -2.5691374 -3.0352786 -
0.1661083
H -1.3131917 -1.5181662
1.2821483
H 2.3930560 0.4563988
4.8438257
H 4.2700535 0.5775203
6.4031775
H 3.9349568 0.0696708
8.8089095
H 1.7157809 -0.6422524
9.6059435
H -2.2437189 -1.3919635
10.2533295
H -4.3557984 -2.3378826
11.0559762
H -6.0206298 -3.2209251
9.4604457
H -5.5880535 -3.1608313
7.0513816

NiH$_2$PhenTAA_2eOX_Quintet:
49 atoms
C -0.6446205 1.9485475
1.8501398
C 0.2910093 2.4427911
2.7492878
C 0.5320649 1.7830397
3.9444214
C -0.1296667 0.5904602
4.2470219
C -1.1025759 0.0804700
3.3154379
C -1.3506895 0.7911223
2.1393742
N -1.8314532 -1.0052910
3.7503629
N -0.0488692 -0.0794255
5.4488404
C 0.9652886 -0.0227131
6.3438727
C -2.5465043 -1.8603012
2.9827344
C -3.6114386 -2.6439555
3.5698103
C -4.3746673 -3.4463635
2.7187139
C -4.0968116 -3.5720870
1.3687979
C -3.0035823 -2.8984767
0.8269739
C -2.2503872 -2.0606803
1.6137489
C 2.2798643 0.3354186
5.9625697
C 3.3002214 0.4257738
6.8789462
C 3.0564565 0.1644483
8.2257210
C 1.7956245 -0.2451606
8.6225024
C 0.7358726 -0.3902184
7.7241187
C -3.9720668 -2.7193871
4.9604007
C -0.4734283 -0.9183694
8.2965710
N -1.5394777 -1.2677125
7.6516844
N -3.3259373 -2.1906312
5.9489837
C -3.6501969 -2.3286322
7.3057093
C -2.6897591 -1.8307487
8.2214545
C -2.9392143 -1.9105830
9.5876260
C -4.1123477 -2.4789755
10.0453982
C -5.0534202 -2.9714164
9.1467267
C -4.8263465 -2.8955327
7.7859880
Ni -1.6693922 -1.1610332
5.6953125
H -0.4287516 -1.0710598
9.3752199
H -4.8485364 -3.3359284
5.1630958
H -0.8550889 2.4855996
0.9355409
H 0.8095464 3.3675890
2.5371704
H 1.1953062 2.2262644
4.6728974
H -2.1411696 0.4675588
1.4777432
H -5.1999399 -4.0039297
3.1433605
H -4.7058047 -4.2157152
0.7494216
H -2.7370546 -3.0417911 -
0.2113653
H -1.3754466 -1.5792276
1.2006829
H 2.4889947 0.4867218
4.9133738
H 4.2958948 0.6879421
6.5476772
H 3.8517756 0.2485175
8.9531682
H 1.6194665 -0.4828378
9.6639958
H -2.2269286 -1.5140515
10.2980029
H -4.3022514 -2.5312569
11.1083359
H -5.9727938 -3.4070797
9.5125163
H -5.5757671 -3.2656922
7.0999114

NiH$_2$PhenTAA_3eOX_Doublet:
49 atoms
C -0.7768191 1.9648984
1.9622710
C 0.1721075 2.4669904
2.8737094
C 0.4392659 1.8112576
4.0535494
C -0.2268675 0.6095005
4.3458396
C -1.2034776 0.0936916
3.4079045
C -1.4656986 0.8033403
2.2244676
N -1.8354245 -1.0332054
3.8269797
N -0.1078296 -0.1241859
5.4831483
C 0.9484332 -0.0880376
6.3296833
C -2.4988262 -1.8987675
3.0240309
C -3.5371415 -2.7270907
3.5785907
C -4.2663448 -3.5401191
2.7141474
C -3.9541193 -3.6302394
1.3642486
C -2.8800288 -2.9013740
0.8449408
C -2.1725611 -2.0461938
1.6529936
C 2.2430215 0.2934082
5.8960737
C 3.2956304 0.3545730
6.7751285
C 3.1014087 0.0500005
8.1256447
C 1.8547151 -0.3696783
8.5699837
C 0.7727870 -0.4900482
7.7010703
C -3.9021036 -2.7498101
4.9570438
C -0.4512142 -0.9720035
8.2515861
N -1.5198958 -1.2823262
7.5847493
N -3.2537139 -2.1835779
5.9275385
C -3.6422604 -2.2666440
7.2715236
C -2.6901698 -1.7683402
8.1819979
C -2.9490090 -1.7946277
9.5491458
C -4.1463341 -2.3205092
9.9987596
C -5.0880583 -2.8200289
9.0965520
C -4.8429878 -2.7955637
7.7357826
Ni -1.6750204 -1.1671303
5.7074836
H -0.4544168 -1.1196984
9.3287826
H -4.7869476 -3.3337476
5.1985697
H -1.0091520 2.5330757
1.0706092
H 0.6543270 3.4140649
2.6684962

H 1.0979434 2.2588348 4.7839367
H -2.2585629 0.4812076 1.5647354
H -5.0837180 -4.1283505 3.1111741
H -4.5228340 -4.2918774 0.7238193
H -2.5902775 -3.0256995 -0.1900950
H -1.3056077 -1.5318668 1.2626561
H 2.4145468 0.4636912 4.8428349
H 4.2819659 0.6168181 6.4155565
H 3.9295619 0.1078803 8.8200618
H 1.7201772 -0.6268267 9.6128937
H -2.2436437 -1.3927205 10.2626155
H -4.3595491 -2.3315636 11.0591621
H -6.0252933 -3.2171345 9.4629550
H -5.5949828 -3.1682465 7.0546674

NiH$_2$PhenTAA_3eOX_Quartet:
49 atoms
C -0.7589232 1.9893125 1.9434596
C 0.1776765 2.4865260 2.8452879
C 0.4405970 1.8176469 4.0341022
C -0.2136930 0.6173686 4.3108898
C -1.1746917 0.1090162 3.3869922
C -1.4494489 0.8155943 2.2162624
N -1.8384055 -1.0155155 3.8356686
N -0.1245307 -0.1128300 5.4800467
C 0.9340119 -0.0781226 6.3343773
C -2.5084649 -1.8867406 3.0329739
C -3.5664194 -2.6987959 3.5797468
C -4.3087536 -3.5262328 2.7130846
C -3.9922053 -3.6405043 1.3757506
C -2.9031371 -2.9271924 0.8725390
C -2.1787406 -2.0644908 1.6810691
C 2.2267020 0.2707071 5.9136676
C 3.2825639 0.3329805 6.8101329
C 3.0941169 0.0533147 8.1643104
C 1.8463678 -0.3403996 8.6002818
C 0.7563325 -0.4565410 7.7142452
C -3.9124296 -2.7403979 4.9419269
C -0.4434105 -0.9523187 8.2543917
N -1.5181084 -1.2841236 7.5691637
N -3.2375781 -2.1802248 5.9245204
C -3.6316551 -2.2844128 7.2497039
C -2.6627150 -1.7749296 8.1773411
C -2.9233547 -1.8034408 9.5452078
C -4.1135344 -2.3380758 9.9860695
C -5.0646221 -2.8463362 9.0734786
C -4.8308439 -2.8178539 7.7166177
Ni -1.6591277 -1.1717482 5.6937575
H -0.4499130 -1.1174376 9.3287128
H -4.7878419 -3.3332359 5.1940241
H -0.9854889 2.5413447 1.0410092
H 0.6757910 3.4250380 2.6416827
H 1.1045092 2.2619942 4.7618089
H -2.2364308 0.4892191 1.5514304
H -5.1316758 -4.0990143 3.1223345
H -4.5631355 -4.2958465 0.7324849
H -2.6094552 -3.0492399 -0.1625552
H -1.3146580 -1.5569479 1.2761671
H 2.4130070 0.4448089 4.8636642
H 4.2680784 0.5951677 6.4457831
H 3.9217209 0.1179744 8.8572231
H 1.6935793 -0.5890078 9.6432017
H -2.2235303 -1.3979523 10.2613258
H -4.3321199 -2.3601134 11.0458761
H -5.9952628 -3.2517264 9.4493927
H -5.5857487 -3.1902643 7.0393663

NiH$_2$PhenTAA_3eOX_Sextet:
49 atoms
C -0.6870551 1.9850176 1.8765977
C 0.2485119 2.4800383 2.7800531
C 0.5013114 1.8096260 3.9699089
C -0.1450713 0.6030155 4.2605306
C -1.1229285 0.0924467 3.3229659
C -1.3836666 0.8156365 2.1555609
N -1.8353939 -1.0056588 3.7459175
N -0.0436423 -0.0860483 5.4464657
C 0.9729369 -0.0279032 6.3435774
C -2.5491513 -1.8643087 2.9770019
C -3.6025917 -2.6757317 3.5691605
C -4.3626758 -3.4982645 2.7125864
C -4.0909260 -3.6155528 1.3623195
C -3.0106845 -2.9168806 0.8237050
C -2.2579437 -2.0640744 1.6135154
C 2.2805517 0.3321873 5.9673266
C 3.3093557 0.4195902 6.8910432
C 3.0810246 0.1411980 8.2384179
C 1.8270304 -0.2822120 8.6333613
C 0.7535521 -0.4209961 7.7283153
C -3.9420817 -2.7798425 4.9441157
C -0.4249978 -0.9734781 8.2947494
N -1.5165501 -1.3104251 7.6424900
N -3.2987152 -2.2221445 5.9466441
C -3.6469299 -2.3362131 7.2749281
C -2.6561595 -1.8292759 8.2174287
C -2.9301112 -1.8667905 9.5857999
C -4.1213322 -2.3945338 10.0270886
C -5.0829011 -2.8869569 9.1117046
C -4.8514319 -2.8510558 7.7560108
Ni -1.6377831 -1.1975180 5.6789000
H -0.3675783 -1.1787528 9.3632988
H -4.7810777 -3.4404772 5.1606849
H -0.9055006 2.5315503 0.9687051
H 0.7571039 3.4127791 2.5751684
H 1.1626171 2.2551438 4.6999023
H -2.1746565 0.4963682 1.4912736
H -5.1753878 -4.0715458 3.1417891
H -4.6895263 -4.2670584 0.7405130
H -2.7484129 -3.0437294 -0.2190956
H -1.3929532 -1.5730479 1.1893224
H 2.4938236 0.4980742 4.9205216
H 4.2998852 0.7010674 6.5561052
H 3.8812985 0.2254591 8.9609000
H 1.6548809 -0.5362453 9.6721600
H -2.2222250 -1.4716170 10.3009551
H -4.3354632 -2.4288841 11.0875684
H -6.0150107 -3.2883494 9.4883823
H -5.6103673 -3.2076259 7.0736547

NiMe$_2$PhenTAA:
NiMe$_2$PhenTAA_2eRED_CSS:
55 atoms
C -0.3367743 2.1724151 2.1732843
C 0.6481558 2.4534638 3.1154188
C 0.7852806 1.6434586 4.2340636
C -0.0434263 0.5328580 4.4454078
C -1.0602382 0.2421882 3.4719545
C -1.1770346 1.0832989 2.3565059
N -1.9195490 -0.7723979 3.8227281
N -0.0892486 -0.2490425 5.5759404
C 0.9542453 -0.4819981 6.4181765
C -2.6617030 -1.5135900 2.9560691
C -3.7890708 -2.3040224 3.4318449
C -4.5521513 -2.9700550 2.4308151
C -4.2223282 -2.9854887 1.0936065
C -3.0591629 -2.3362956 0.6714663
C -2.3099221 -1.6339988 1.5962246
C 2.2915570 -0.3235677 6.0009078
C 3.3799935 -0.5084954 6.8320241
C 3.1632469 -0.8917423 8.1581922
C 1.8773939 -1.1437933 8.5825150
C 0.7258367 -1.0164090 7.7544155
C -4.1481611 -2.4824107 4.7943526
C -0.5293434 -1.4461681 8.2620274
N -1.6818136 -1.3003153 7.5666202
N -3.5213707 -1.8299957 5.8019084
C -3.9646001 -1.7267849 7.0850652
C -2.9414040 -1.4323184 8.0671542
C -3.3423449 -1.1823205 9.3927851
C -4.6702975 -1.2200573 9.7709402
C -5.6617546 -1.5045595 8.8192053
C -5.3073321 -1.7465911 7.5063549
Ni -1.7768477 -1.1114227 5.6865501
C -0.5416459 -2.2643530 9.5227706
C -5.1203418 -3.5775675 5.1331279
H -0.4680488 2.8115721 1.3067450
H 1.2954568 3.3154337 2.9945849
H 1.5166559 1.8960658 4.9900721
H -1.9748169 0.8995408 1.6493621
H -5.4599351 -3.4752119 2.7336983
H -4.8616122 -3.5004984 0.3842884
H -2.7376488 -2.3829394 -0.3634388
H -1.3949156 -1.1539563 1.2759944
H 2.4603887 -0.0540275 4.9668474
H 4.3844160 -0.3619532 6.4499969
H 3.9920966 -0.9964217 8.8503307
H 1.7335284 -1.4351625 9.6146541
H -2.5916751 -0.8933936 10.1167851
H -4.9450462 -0.9875677 10.7939874
H -6.7091137 -1.4937096 9.1000665
H -6.0818431 -1.8956964 6.7656947
H -1.4578571 -2.8501147 9.5810876
H 0.3060731 -2.9550387 9.5442382
H -0.4837506 -1.6818291 10.4524051

H -5.0059585 -3.8717715
6.1754112
H -6.1783406 -3.3148764
4.9968046
H -4.9438963 -4.4583651
4.5089611

NiMe$_2$PhenTAA_2eRED_Triplet:
55 atoms
C -0.3626712 2.1916848
2.1985468
C 0.6192149 2.4713878
3.1370226
C 0.7721353 1.6451686
4.2465450
C -0.0436318 0.5257194
4.4461115
C -1.0589729 0.2358483
3.4747299
C -1.1899995 1.0860087
2.3706885
N -1.8991010 -0.8033128
3.8204850
N -0.0783737 -0.2816858
5.5653464
C 0.9651026 -0.5001430
6.4171878
C -2.6550140 -1.5334333
2.9506503
C -3.7666933 -2.3419564
3.4293338
C -4.5241098 -3.0228709
2.4361889
C -4.2195817 -3.0110411
1.0893887
C -3.0920962 -2.3178548
0.6571081
C -2.3385772 -1.6096408
1.5807410
C 2.2993531 -0.2892495
6.0201044
C 3.3888723 -0.4790127
6.8564904
C 3.1749688 -0.9143353
8.1615954
C 1.8874735 -1.2004608
8.5707526
C 0.7390604 -1.0564073
7.7435641
C -4.1336572 -2.5085198
4.7965383
C -0.5238008 -1.4751942
8.2556050
N -1.6724073 -1.3664222
7.5411683
N -3.4773692 -1.8868711
5.8091737
C -3.9578655 -1.7230072
7.0868204
C -2.9443472 -1.4316149
8.0600126
C -3.3341412 -1.1447502
9.3745007
C -4.6806566 -1.1611387
9.7494749
C -5.6588186 -1.4409487
8.8098609
C -5.3006269 -1.7080310
7.4853733
Ni -1.7506560 -1.1508184
5.6712566
C -0.5404985 -2.2663733
9.5346087
C -5.1308844 -3.5830225
5.1337677
H -0.5018569 2.8359020
1.3368812
H 1.2608200 3.3385652
3.0224762
H 1.5041666 1.8979932
5.0010816
H -1.9869229 0.9033443
1.6630772
H -5.4048501 -3.5671120
2.7490280
H -4.8526275 -3.5419038
0.3864375
H -2.7941420 -2.3303562 -
0.3860359
H -1.4429563 -1.1047467
1.2468915
H 2.4735155 0.0117885
4.9964206
H 4.3916314 -0.2943854
6.4860997
H 4.0022685 -1.0365611
8.8524398
H 1.7453101 -1.5365573
9.5888868
H -2.5823850 -0.8599264
10.0980399
H -4.9538255 -0.9290780
10.7733859
H -6.7070314 -1.4305805
9.0887501
H -6.0730122 -1.8595113
6.7439014
H -1.4836872 -2.7952045
9.6463757
H 0.2627674 -3.0088215
9.5354388
H -0.4005931 -1.6639884
10.4413459
H -5.0848303 -3.8311634
6.1909335
H -6.1728723 -3.3200017
4.9108115
H -4.9185160 -4.4933959
4.5655908

NiMe$_2$PhenTAA_2eRED_Quintet:
55 atoms
C -0.2913265 2.1088416
2.1737913
C 0.6529123 2.4130315
3.1401207
C 0.7959169 1.5742442
4.2425862
C 0.0266060 0.4193330
4.4053237
C -0.9625781 0.0971791
3.3956166
C -1.0844331 0.9743217
2.3054185
N -1.7797779 -0.9634269
3.6881482
N 0.0040345 -0.3826598
5.5340354
C 1.0206861 -0.4980721
6.4586101
C -2.6668861 -1.5699933
2.8409097
C -3.8161477 -2.3051834
3.3684258
C -4.6679281 -2.8976202
2.3909459
C -4.4288978 -2.8771190
1.0293088
C -3.2826281 -2.2558337
0.5508592
C -2.4405814 -1.6176685
1.4546038
C 2.3512189 -0.2431157
6.0968084
C 3.4306267 -0.3550764
6.9697979
C 3.1864516 -0.7252445
8.2833796
C 1.8962430 -1.0433944
8.6687646
C 0.7660587 -1.0130558
7.8015421
C -4.1650568 -2.5048922
4.7473171
C -0.4827600 -1.4895055
8.3255303
N -1.6257490 -1.5115863
7.5941339
N -3.4921905 -1.9380606
5.7917830
C -3.9363609 -1.8091491
7.0825572
C -2.9077008 -1.5553295
8.0753628
C -3.3184891 -1.2606497
9.3856080
C -4.6640538 -1.2566104
9.7576198
C -5.6420806 -1.5193397
8.8140237
C -5.2772021 -1.7789882
7.4915149
Ni -1.6374505 -1.3610811
5.6137368
C -0.4586657 -2.1881750
9.6602623
C -5.2006444 -3.5541989
5.0567705
H -0.4300318 2.7585925
1.3155257
H 1.2679941 3.3023082
3.0555976
H 1.4868892 1.8472854
5.0285356
H -1.8615690 0.7918720
1.5771781
H -5.5761763 -3.3779975
2.7274332
H -5.1342212 -3.3476543
0.3524119
H -3.0359877 -2.2675873 -
0.5055036
H -1.5249391 -1.1733213
1.0896762
H 2.5409293 0.0232121
5.0662762
H 4.4357566 -0.1486029
6.6188367
H 3.9914428 -0.7756963
9.0093728
H 1.7421453 -1.3253315
9.7006257
H -2.5737676 -0.9753853
10.1153114
H -4.9359971 -1.0160637
10.7801922
H -6.6920998 -1.4926650
9.0856703
H -6.0505627 -1.8981465
6.7451311
H -1.3698134 -2.7651652
9.7993726
H 0.3865564 -2.8804040
9.7131784
H -0.3600532 -1.5231437
10.5282990
H -5.1310704 -3.8557545
6.0994968
H -6.2382765 -3.2392468
4.8847400
H -5.0463136 -4.4410258
4.4364250

NiMe$_2$PhenTAA_1eRED_Doublet:
55 atoms
C -0.3560433 2.1993108
2.1856473
C 0.6265340 2.4805656
3.1257139
C 0.7663133 1.6742007
4.2445232
C -0.0579779 0.5627039
4.4432748
C -1.0662674 0.2734306
3.4772693
C -1.1964439 1.1121067
2.3663922
N -1.9275833 -0.7483521
3.8377904
N -0.1103132 -0.2283191
5.5783975
C 0.9303947 -0.4911578
6.4034074
C -2.6437828 -1.5123507
2.9806378
C -3.7341101 -2.3452220
3.4449887
C -4.4667147 -3.0511310
2.4588814
C -4.1521650 -3.0438301
1.1222245
C -3.0238918 -2.3350695
0.7019469
C -2.3011300 -1.6013286
1.6093056
C 2.2697028 -0.2946993
5.9868744
C 3.3501422 -0.5186989
6.8034814
C 3.1516957 -0.9677990
8.1114323
C 1.8729805 -1.2490530
8.5256597
C 0.7250714 -1.0747338
7.7136063
C -4.1178048 -2.5046460
4.8159934
C -0.5429016 -1.4861246
8.2389073
N -1.6788243 -1.3135673
7.5582008
N -3.5107548 -1.8381800
5.8018868
C -3.9651249 -1.7286909
7.1008422
C -2.9548899 -1.4409482
8.0698359
C -3.3433790 -1.1615728
9.3853572
C -4.6745766 -1.1818697
9.7590332
C -5.6596248 -1.4612889
8.8141413
C -5.3061661 -1.7190376
7.5023267
Ni -1.7913938 -1.0648723
5.6878317
C -0.5630871 -2.2651015
9.5246062
C -5.1297606 -3.5643980
5.1530741
H -0.4909369 2.8424634
1.3246995
H 1.2667164 3.3460923
3.0071058
H 1.4892366 1.9353886
5.0046792
H -2.0007722 0.9361279
1.6657942
H -5.3335616 -3.6164804
2.7689433
H -4.7592418 -3.5951757
0.4159070
H -2.7057067 -2.3688052 -
0.3335457
H -1.4096332 -1.0876183
1.2808511
H 2.4368377 0.0161037
4.9659694
H 4.3515071 -0.3568768
6.4221569
H 3.9871680 -1.1127188
8.7839844
H 1.7387756 -1.6095518
9.5352596
H -2.5930995 -0.8659955
10.1051308
H -4.9487086 -0.9378881
10.7777488
H -6.7057936 -1.4365209
9.0918533
H -6.0794167 -1.8561372
6.7599843
H -1.5019643 -2.8004983
9.6346836
H 0.2432863 -2.9982831
9.5345666
H -0.4342235 -1.6466667
10.4176059

H -5.0820643 -3.8200336 6.2076379
H -6.1617642 -3.2747498 4.9344998
H -4.9327634 -4.4704513 4.5799925

NiMe$_2$PhenTAA_1eRED_Quartet:
55 atoms
C -0.2958487 2.1831935 2.1930788
C 0.6575278 2.4571603 3.1632102
C 0.7819767 1.6233448 4.2634626
C -0.0203670 0.4871019 4.4128855
C -1.0211799 0.2133082 3.4158664
C -1.1272459 1.0808066 2.3234186
N -1.8915865 -0.8029091 3.7335109
N -0.0507217 -0.3527789 5.5096993
C 0.9810189 -0.5179280 6.4200736
C -2.6737644 -1.5093813 2.8939986
C -3.7760273 -2.3060307 3.3976172
C -4.5538190 -2.9998346 2.4463120
C -4.2993018 -2.9918846 1.0947390
C -3.1786913 -2.3019899 0.6297023
C -2.3938610 -1.5975971 1.5069373
C 2.3048483 -0.2923965 6.0338679
C 3.3905049 -0.4594148 6.8862369
C 3.1523285 -0.8580386 8.1905709
C 1.8621314 -1.1479551 8.5915941
C 0.7263090 -1.0558364 7.7435052
C -4.1308333 -2.4739880 4.7953218
C -0.5313074 -1.5085321 8.2668211
N -1.6838992 -1.4819189 7.5404933
N -3.5938997 -1.7413862 5.7552464
C -3.9991095 -1.7082487 7.0893422
C -2.9535598 -1.4845478 8.0448258
C -3.3403632 -1.1941163 9.3680922
C -4.6717994 -1.2042709 9.7490416
C -5.6738249 -1.4659179 8.8249890
C -5.3279387 -1.7040463 7.4984696
Ni -1.7120846 -1.2077684 5.6270833
C -0.5246629 -2.2440811 9.5777964
C -5.0632168 -3.6030871 5.1481107
H -0.4135598 2.8413357 1.3408072
H 1.2904898 3.3316985 3.0786463
H 1.4836209 1.8728873 5.0466134
H -1.9131175 0.9192069 1.5993568
H -5.4151534 -3.5531286 2.7919529
H -4.9484176 -3.5261105 0.4137013
H -2.9153018 -2.3275429 -0.4215223
H -1.5027067 -1.1049872 1.1453744
H 2.4856278 -0.0081074 5.0070766
H 4.3950578 -0.2745004 6.5276045
H 3.9668969 -0.9592543 8.8978312
H 1.7162137 -1.4607904 9.6147065
H -2.5849544 -0.9214146 10.0901612
H -4.9294957 -0.9771936 10.7770815
H -6.7151807 -1.4495318 9.1187663
H -6.1041001 -1.8282372 6.7547818
H -1.4507688 -2.7963674 9.7117729
H 0.2954239 -2.9651885 9.6062691
H -0.3975905 -1.6020723 10.4569242
H -4.9902174 -3.8369295 6.2065043
H -6.1112398 -3.3734199 4.9414675
H -4.8172580 -4.4973817 4.5772020

NiMe$_2$PhenTAA_1eRED_Sextet:
55 atoms
C -0.5387340 2.1576057 2.3676304
C 0.4376141 2.4494216 3.3251785
C 0.6838508 1.5748611 4.3612177
C -0.0296794 0.3670199 4.4857929
C -1.0330341 0.0491533 3.4737162
C -1.2611942 0.9883386 2.4450695
N -1.7257227 -1.0900342 3.6833010
N 0.0457138 -0.5011568 5.5209439
C 1.0665582 -0.5844832 6.4590092
C -2.6353160 -1.6586886 2.8145698
C -3.7761433 -2.3853321 3.3416836
C -4.6234610 -2.9713149 2.3582520
C -4.3726356 -2.9213835 1.0040866
C -3.2384014 -2.2775791 0.5248772
C -2.3991237 -1.6544885 1.4386395
C 2.3763333 -0.2868915 6.0806348
C 3.4601708 -0.3789752 6.9456833
C 3.2253577 -0.7836948 8.2514689
C 1.9510273 -1.1348155 8.6470767
C 0.8143173 -1.1058779 7.7917196
C -4.1256655 -2.5608396 4.7164842
C -0.4370737 -1.5650362 8.3212445
N -1.5670310 -1.6164422 7.5657507
N -3.4181127 -1.9942721 5.7474704
C -3.8796155 -1.7704496 7.0221106
C -2.8569248 -1.5432742 8.0186304
C -3.2716783 -1.1560498 9.3020225
C -4.6179114 -1.0440275 9.6304770
C -5.5952663 -1.2854464 8.6784611
C -5.2227384 -1.6296597 7.3834344
Ni -1.5396774 -1.6093654 5.5865061
C -0.4443556 -2.1867749 9.6904342
C -5.1968941 -3.5595131 5.0531807
H -0.7508871 2.8669467 1.5773692
H 0.9816500 3.3847181 3.2751196
H 1.3922867 1.8382466 5.1333586
H -2.0560360 0.7991119 1.7382488
H -5.5210718 -3.4744447 2.6854609
H -5.0640164 -3.3975368 0.3190680
H -3.0016904 -2.2669070 -0.5309379
H -1.4873752 -1.1869048 1.0916217
H 2.5460117 -0.0024785 5.0507656
H 4.4570753 -0.1391440 6.5991345
H 4.0379952 -0.8397833 8.9661840
H 1.8144286 -1.4450578 9.6720797
H -2.5285869 -0.8870176 10.0378929
H -4.8962878 -0.7348635 10.6310699
H -6.6434043 -1.1703572 8.9249633
H -5.9816337 -1.7344566 6.6208048
H -1.3752308 -2.7210172 9.8594542
H 0.3699467 -2.9089024 9.7881543
H -0.3203074 -1.4731285 10.5123856
H -5.1549425 -3.8214001 6.1068967
H -6.2142264 -3.2079855 4.8494070
H -5.0662506 -4.4761717 4.4737403

NiMe$_2$PhenTAA_0eNeutral_CSS:
55 atoms
C -0.4302197 2.2598761 2.2514385
C 0.5545290 2.5351462 3.1861256
C 0.7165381 1.7122136 4.2902712
C -0.0880362 0.5876442 4.4598102
C -1.0920476 0.3049813 3.5033137
C -1.2572264 1.1593202 2.4156375
N -1.9259779 -0.7557471 3.8594874
N -0.1271063 -0.2460243 5.5786051
C 0.9143022 -0.5316179 6.3852977
C -2.6201472 -1.5323522 3.0047545
C -3.6550047 -2.4318157 3.4592999
C -4.3451060 -3.1853511 2.4797444
C -4.0488408 -3.1396598 1.1450274
C -2.9855003 -2.3348765 0.7242101
C -2.3066674 -1.5574376 1.6200656
C 2.2487502 -0.2751044 5.9741340
C 3.3303903 -0.5550923 6.7616180
C 3.1571035 -1.1005095 8.0374555
C 1.8901328 -1.4127293 8.4482158
C 0.7351249 -1.1808353 7.6636202
C -4.0711249 -2.5597478 4.8276551
C -0.5405025 -1.5448796 8.2146437
N -1.6585767 -1.3702296 7.5375602
N -3.4722683 -1.8933750 5.7960747
C -3.9550631 -1.7393250 7.1053522
C -2.9586147 -1.4509342 8.0629694
C -3.3370098 -1.1047336 9.3556184
C -4.6765712 -1.0760522 9.7150558
C -5.6526171 -1.3583936 8.7768851
C -5.2943032 -1.6707484 7.4736514
Ni -1.7912824 -1.0610804 5.6865179
C -0.5759460 -2.2235856 9.5542496
C -5.1508513 -3.5500012 5.1587739
H -0.5840455 2.9214397 1.4094008
H 1.1767332 3.4138852 3.0809974
H 1.4347778 1.9759504 5.0529633
H -2.0709776 0.9943402 1.7242951
H -5.1545745 -3.8266801 2.7923598
H -4.6095578 -3.7345202 0.4382534
H -2.6776044 -2.3367801 -0.3141813
H -1.4593088 -0.9821923 1.2821722
H 2.4074012 0.0993266 4.9749504
H 4.3261084 -0.3691131 6.3785765
H 4.0048298 -1.2981413 8.6780273
H 1.7743262 -1.8529846 9.4265231
H -2.5887954 -0.7955043 10.0699119
H -4.9529002 -0.7879130 10.7203060
H -6.6993228 -1.2939028 9.0414246
H -6.0635380 -1.8008548 6.7277364
H -1.5334062 -2.6999027 9.7313993
H 0.1863319 -2.9980653 9.5913000
H -0.3761988 -1.5426283 10.3824375

H -5.1790205 -3.7633644 6.2210638
H -6.1433824 -3.2185190 4.8507880
H -4.9561352 -4.4868182 4.6421546

NiMe₂PhenTAA_0eNeutral_Triplet:
55 atoms
C -0.3989487 2.2055790 2.2211345
C 0.5936273 2.4875229 3.1680845
C 0.7596711 1.6804057 4.2686226
C -0.0543504 0.5500507 4.4574467
C -1.0768241 0.2580128 3.4793547
C -1.2234281 1.1165620 2.3759636
N -1.8723248 -0.7979945 3.8011527
N -0.0613504 -0.2795567 5.5365853
C 0.9696764 -0.5080051 6.3991789
C -2.6350636 -1.5366184 2.9467076
C -3.7186707 -2.3479022 3.4359223
C -4.4646074 -3.0559877 2.4719664
C -4.1686137 -3.0492419 1.1284942
C -3.0542474 -2.3450548 0.6831160
C -2.3097412 -1.6151077 1.5782764
C 2.3033238 -0.3038190 5.9941859
C 3.3712725 -0.5223912 6.8309869
C 3.1471702 -0.9726566 8.1282882
C 1.8612481 -1.2554622 8.5273975
C 0.7336786 -1.0772070 7.7002824
C -4.0978363 -2.5049700 4.8277321
C -0.5550985 -1.4922865 8.2227560
N -1.6777576 -1.2659269 7.5648903
N -3.5306994 -1.7968556 5.7878299
C -3.9691122 -1.6885587 7.0969113
C -2.9635793 -1.4008723 8.0617119
C -3.3478170 -1.1206177 9.3777425
C -4.6766170 -1.1419588 9.7469919
C -5.6609760 -1.4234487 8.8025158
C -5.3109970 -1.6820780 7.4936109
Ni -1.7528050 -1.0951310 5.6569204
C -0.5743586 -2.2947132 9.4914190
C -5.0830443 -3.5851512 5.1696229
H -0.5438112 2.8700329 1.3796977
H 1.2107813 3.3688575 3.0542375
H 1.4823321 1.9416940 5.0282112
H -2.0290934 0.9433030 1.6770495
H -5.3232308 -3.6246900 2.7949344
H -4.7853107 -3.6054752 0.4360524
H -2.7643634 -2.3731246 -0.3592753
H -1.4209878 -1.1030389 1.2394842
H 2.4807007 0.0052065 4.9742721
H 4.3779393 -0.3511602 6.4726163
H 3.9709867 -1.1231124 8.8121563
H 1.7154873 -1.6213019 9.5323778
H -2.5960174 -0.8302058 10.0975623
H -4.9530536 -0.9029425 10.7651835
H -6.7053730 -1.4046064 9.0834112
H -6.0822490 -1.8271178 6.7511019
H -1.5240558 -2.8076733 9.6085972
H 0.2156981 -3.0435263 9.4784294
H -0.4175922 -1.6866557 10.3848665
H -5.0490332 -3.8170323 6.2294196
H -6.1131995 -3.3172587 4.9253245
H -4.8553538 -4.4947313 4.6164874

NiMe₂PhenTAA_0eNeutral_Quintet:
55 atoms
C -0.3589884 2.1145357 2.1972512
C 0.6417801 2.3987750 3.1286055
C 0.8272993 1.5808387 4.2178111
C 0.0368219 0.4302276 4.4080366
C -1.0049139 0.1399411 3.4396461
C -1.1734837 1.0135529 2.3576166
N -1.8308649 -0.9086295 3.7570558
N 0.0584567 -0.3889651 5.4906258
C 1.0598487 -0.5011948 6.4244661
C -2.6618535 -1.5787376 2.9005690
C -3.7899680 -2.3314641 3.3867276
C -4.5911301 -2.9571166 2.4121505
C -4.3085573 -2.9428548 1.0641448
C -3.1540447 -2.3107504 0.6229771
C -2.3599476 -1.6449495 1.5275733
C 2.3892615 -0.2317870 6.0736504
C 3.4400210 -0.3464715 6.9647473
C 3.1586888 -0.7273379 8.2698214
C 1.8703519 -1.0554204 8.6349564
C 0.7648489 -1.0240343 7.7470934
C -4.1954877 -2.4979394 4.7845024
C -0.4921576 -1.5032275 8.2511590
N -1.6564356 -1.4369689 7.5331102
N -3.5898180 -1.8634370 5.7568597
C -3.9801732 -1.7808918 7.0914906
C -2.9282036 -1.5231867 8.0368021
C -3.2981772 -1.2592993 9.3681307
C -4.6188160 -1.2626710 9.7632708
C -5.6325101 -1.5065242 8.8425977
C -5.3071315 -1.7511112 7.5211014
Ni -1.7005765 -1.2151042 5.6322489
C -0.4850205 -2.2646374 9.5446690
C -5.2760077 -3.4993101 5.0798582
H -0.5248400 2.7822302 1.3620333
H 1.2474024 3.2882437 3.0171974
H 1.5450439 1.8504031 4.9781207
H -1.9908877 0.8480914 1.6711297
H -5.4850112 -3.4723698 2.7269269
H -4.9698034 -3.4408203 0.3687767
H -2.8706348 -2.3415890 -0.4210767
H -1.4432044 -1.1833911 1.1906339
H 2.5955924 0.0187935 5.0428698
H 4.4531676 -0.1501447 6.6426545
H 3.9495472 -0.7897307 9.0062617
H 1.7017209 -1.3479586 9.6596201
H -2.5349218 -0.9868679 10.0807226
H -4.8655766 -1.0313870 10.7915715
H -6.6701681 -1.4642456 9.1434609
H -6.0988594 -1.8550428 6.7938212
H -1.3832492 -2.8689382 9.6372626
H 0.3708496 -2.9383973 9.5826332
H -0.4237066 -1.6366099 10.4389629
H -5.2534259 -3.7818766 6.1278362
H -6.2790928 -3.1301808 4.8589113
H -5.1310537 -4.3980604 4.4843429

NiMe₂PhenTAA_1eOX_Doublet:
55 atoms
C -0.4945048 2.2600410 2.3069362
C 0.5034575 2.5373841 3.2514748
C 0.6899529 1.7188040 4.3390443
C -0.1149341 0.5819997 4.5069133
C -1.1359890 0.2952625 3.5353829
C -1.3080110 1.1617583 2.4457489
N -1.8913917 -0.7991875 3.8461910
N -0.1016681 -0.2924091 5.5562007
C 0.9499910 -0.5584571 6.3840577
C -2.6121583 -1.5656689 2.9781517
C -3.6380503 -2.4527736 3.4437654
C -4.3280645 -3.2031235 2.4745166
C -4.0295085 -3.1538543 1.1329218
C -2.9800219 -2.3501467 0.7035318
C -2.2946764 -1.5744507 1.6063823
C 2.2678149 -0.2943897 5.9648656
C 3.3553678 -0.5690229 6.7579342
C 3.1727646 -1.1171838 8.0220662
C 1.8998658 -1.4317981 8.4370850
C 0.7559244 -1.2001444 7.6519802
C -4.0635280 -2.5721052 4.8285214
C -0.5381649 -1.5572392 8.2108487
N -1.6455249 -1.3735855 7.5353414
N -3.4695208 -1.8999839 5.7838854
C -3.9484347 -1.7240239 7.0949274
C -2.9536908 -1.4354502 8.0509471
C -3.3292127 -1.0731143 9.3405338
C -4.6675605 -1.0294374 9.6917006
C -5.6449748 -1.3128698 8.7520614
C -5.2895470 -1.6414380 7.4551757
Ni -1.7618378 -1.1081119 5.6696475
C -0.5716951 -2.2096110 9.5587216
C -5.1568126 -3.5431631 5.1525117
H -0.6572699 2.9436219 1.4851428
H 1.1019870 3.4322366 3.1495345
H 1.4104497 1.9810680 5.0995685
H -2.1210421 0.9969790 1.7540912
H -5.1353022 -3.8473899 2.7844406
H -4.5898059 -3.7539822 0.4307061
H -2.6840927 -2.3431416 -0.3371148
H -1.4473744 -0.9962487 1.2717643
H 2.4230108 0.0801113 4.9645715
H 4.3511228 -0.3725612 6.3834792
H 4.0173511 -1.3205067 8.6645608
H 1.7869096 -1.8729653 9.4145758
H -2.5819839 -0.7636493 10.0552091
H -4.9462611 -0.7277087 10.6917576
H -6.6907915 -1.2343359 9.0144203
H -6.0587994 -1.7722636 6.7099045
H -1.5317382 -2.6732073 9.7520905
H 0.1846594 -2.9892509 9.6036691
H -0.3574456 -1.5127644 10.3691891

H -5.2027994 -3.7487559 6.2153342
H -6.1383031 -3.1989049 4.8255090
H -4.9661367 -4.4848861 4.6436219

$NiMe_2PhenTAA\_1eOX\_Quartet$:
55 atoms
C -0.3361666 2.1850103 2.1712960
C 0.6369793 2.4713567 3.1275105
C 0.7844303 1.6709310 4.2424963
C -0.0215791 0.5430481 4.4195342
C -1.0290152 0.2461767 3.4318698
C -1.1655880 1.0955716 2.3256729
N -1.8727714 -0.7829687 3.7641171
N -0.0486798 -0.2610251 5.5389858
C 0.9756951 -0.4923814 6.4056629
C -2.6443265 -1.5182433 2.9253796
C -3.7280206 -2.3243966 3.4345834
C -4.4962728 -3.0156802 2.4823047
C -4.2203563 -3.0050371 1.1330953
C -3.1001915 -2.3224329 0.6696511
C -2.3345307 -1.6014878 1.5499029
C 2.3114891 -0.2831826 6.0062931
C 3.3723720 -0.5040413 6.8494884
C 3.1382321 -0.9491352 8.1447039
C 1.8491244 -1.2363861 8.5377190
C 0.7301898 -1.0638617 7.7062207
C -4.1009751 -2.5006894 4.8368644
C -0.5655603 -1.4846158 8.2440652
N -1.6817095 -1.2485627 7.6035693
N -3.5549205 -1.7858659 5.7918395
C -3.9789035 -1.7158927 7.1247029
C -2.9817257 -1.4235151 8.0901613
C -3.3681389 -1.1534148 9.4024721
C -4.6995338 -1.1945745 9.7701402
C -5.6746211 -1.4829510 8.8261345
C -5.3178399 -1.7292296 7.5142235
Ni -1.7767459 -1.0060756 5.6648489
C -0.5684598 -2.2789272 9.5160855
C -5.0676259 -3.5996899 5.1653787
H -0.4674635 2.8405457 1.3216612
H 1.2609820 3.3474232 3.0153001
H 1.4916704 1.9438069 5.0119579
H -1.9684509 0.9260126 1.6234931
H -5.3540614 -3.5800590 2.8121052
H -4.8546078 -3.5518783 0.4497992
H -2.8245397 -2.3631650 -0.3755346
H -1.4383704 -1.1111697 1.2001159
H 2.4965931 0.0224850 4.9872669
H 4.3817767 -0.3375904 6.4983835
H 3.9572962 -1.0991562 8.8334605
H 1.7041742 -1.6056179 9.5406218
H -2.6228551 -0.8577079 10.1261309
H -4.9794156 -0.9693720 10.7899441
H -6.7191854 -1.4841733 9.1048773
H -6.0865481 -1.8796975 6.7709199
H -1.5155713 -2.7904830 9.6513395
H 0.2205362 -3.0266443 9.4968758
H -0.3976973 -1.6586250 10.3968140
H -5.0314685 -3.8446637 6.2214505
H -6.0981901 -3.3374759 4.9221791
H -4.8268573 -4.4966247 4.5998645

$NiMe_2PhenTAA\_1eOX\_Sextet$:
55 atoms
C -0.4910975 2.1676967 2.3286085
C 0.4730519 2.4570993 3.2865604
C 0.6903579 1.5951609 4.3451649
C -0.0298456 0.3979228 4.4564176
C -1.0421497 0.1108249 3.4690209
C -1.2523254 1.0147455 2.4283100
N -1.8416409 -0.9741080 3.7537624
N 0.0426790 -0.4946639 5.4911016
C 1.0432207 -0.5370240 6.4466756
C -2.6506747 -1.6447803 2.8936559
C -3.7765023 -2.4193674 3.3728102
C -4.5566239 -3.0668354 2.3955452
C -4.2622800 -3.0371851 1.0536292
C -3.1222471 -2.3651893 0.6157155
C -2.3433176 -1.6871056 1.5170043
C 2.3539286 -0.2060611 6.1001946
C 3.4072651 -0.2677937 7.0023636
C 3.1508158 -0.6770688 8.2984081
C 1.8752289 -1.0717892 8.6575517
C 0.7787392 -1.0710030 7.7660910
C -4.1919831 -2.5622760 4.7582335
C -0.4443536 -1.6337213 8.2454183
N -1.6135051 -1.6362290 7.4708895
N -3.5378168 -1.9471453 5.7203659
C -3.9389770 -1.7373288 7.0251631
C -2.8599782 -1.5043050 7.9643810
C -3.1856829 -1.1165238 9.2826327
C -4.4966340 -0.9876924 9.6662385
C -5.5340776 -1.2088448 8.7533168
C -5.2552482 -1.5688425 7.4478179
Ni -1.6108374 -1.4786898 5.5754091
C -0.4471227 -2.4754255 9.4821097
C -5.3182595 -3.4954912 5.0825526
H -0.6777350 2.8611788 1.5203950
H 1.0356848 3.3784961 3.2262492
H 1.3861983 1.8683321 5.1242142
H -2.0555394 0.8408160 1.7271930
H -5.4342723 -3.6118571 2.7048626
H -4.8991960 -3.5547344 0.3505899
H -2.8432211 -2.3835077 -0.4292975
H -1.4375101 -1.2006205 1.1857434
H 2.5564408 0.0562651 5.0713696
H 4.4059970 -0.0017819 6.6864318
H 3.9445831 -0.7111353 9.0321357
H 1.7109752 -1.3972058 9.6737108
H -2.3893193 -0.8934028 9.9768568
H -4.7310722 -0.6881722 10.6788504
H -6.5609941 -1.0741472 9.0628965
H -6.0635556 -1.6686698 6.7387836
H -1.3671809 -3.0475584 9.5609246
H 0.3841043 -3.1817267 9.4784865
H -0.3504323 -1.8876966 10.3998027
H -5.3403876 -3.7193896 6.1437270
H -6.2904905 -3.0865512 4.8041067
H -5.2031833 -4.4338907 4.5448480

$NiMe_2PhenTAA\_2eOX\_CSS$:
55 atoms
C -0.5415157 2.2463137 2.3443875
C 0.4720152 2.5270340 3.3010376
C 0.6743474 1.7202624 4.3791437
C -0.1343866 0.5728013 4.5525829
C -1.1770136 0.2798523 3.5612576
C -1.3491524 1.1569560 2.4651767
N -1.8691506 -0.8307465 3.8325423
N -0.0796908 -0.3225861 5.5437092
C 0.9759276 -0.5744739 6.3854290
C -2.6078427 -1.5888846 2.9582472
C -3.6271430 -2.4728079 3.4345930
C -4.3181080 -3.2129042 2.4689690
C -4.0157922 -3.1581174 1.1205493
C -2.9733673 -2.3559590 0.6829234
C -2.2859338 -1.5830127 1.5911526
C 2.2850299 -0.2987166 5.9584167
C 3.3769687 -0.5677708 6.7524520
C 3.1861564 -1.1185868 8.0101535
C 1.9085270 -1.4403973 8.4304833
C 0.7707430 -1.2187437 7.6466472
C -4.0615250 -2.5860959 4.8291948
C -0.5344219 -1.5723153 8.2121049
N -1.6354520 -1.3868356 7.5335870
N -3.4650462 -1.9132672 5.7773677
C -3.9436104 -1.7113335 7.0854123
C -2.9480887 -1.4240155 8.0418566
C -3.3200707 -1.0511366 9.3297010
C -4.6586431 -0.9947810 9.6743563
C -5.6380008 -1.2769585 8.7330129
C -5.2858731 -1.6178283 7.4394504
Ni -1.7387821 -1.1519916 5.6590501
C -0.5665656 -2.2025901 9.5661736
C -5.1696381 -3.5366623 5.1455174
H -0.7091372 2.9489070 1.5394110
H 1.0507874 3.4350885 3.1977998
H 1.3897446 1.9887245 5.1424915
H -2.1683274 0.9987844 1.7790243
H -5.1274259 -3.8571667 2.7727806
H -4.5763067 -3.7617756 0.4208600
H -2.6830430 -2.3470986 -0.3586922
H -1.4334201 -1.0084368 1.2613055
H 2.4366063 0.0698760 4.9547393
H 4.3731250 -0.3665790 6.3831732
H 4.0294227 -1.3243589 8.6542698
H 1.8017062 -1.8793432 9.4093810
H -2.5740549 -0.7439375 10.0462383
H -4.9388575 -0.6831287 10.6708712
H -6.6830880 -1.1861381 8.9940740
H -6.0565031 -1.7486107 6.6959458
H -1.5270403 -2.6602226 9.7703664
H 0.1879480 -2.9839800 9.6198646
H -0.3457550 -1.4930364 10.3640355

H -5.2303558 -3.7384477 6.2081447
H -6.1415931 -3.1779340 4.8051897
H -4.9873333 -4.4828866 4.6410871

NiMe$_2$PhenTAA_2eOX_Triplet:
55 atoms
C -0.5161582 2.2963016 2.3262494
C 0.4732001 2.5719387 3.2624394
C 0.6703870 1.7357233 4.3513714
C -0.1173925 0.5969915 4.4931771
C -1.1269062 0.3128565 3.5326084
C -1.3251158 1.1778993 2.4606662
N -1.9076955 -0.7857653 3.8641413
N -0.1222887 -0.2793218 5.5699875
C 0.9352314 -0.5757956 6.3638388
C -2.5861122 -1.5733689 2.9957003
C -3.6162216 -2.4654384 3.4593159
C -4.2832767 -3.2167964 2.4889278
C -3.9551799 -3.1721003 1.1445854
C -2.9026916 -2.3707523 0.7177593
C -2.2360346 -1.5868013 1.6260858
C 2.2516682 -0.3229386 5.9159159
C 3.3470796 -0.6105589 6.6918836
C 3.1669901 -1.1582804 7.9565590
C 1.8906615 -1.4619486 8.3986472
C 0.7450483 -1.2225107 7.6359226
C -4.0628594 -2.5719505 4.8451985
C -0.5532885 -1.5629666 8.2116401
N -1.6594423 -1.3557295 7.5450396
N -3.4836058 -1.8813704 5.7935885
C -3.9656519 -1.7039460 7.1092273
C -2.9724881 -1.4166610 8.0637536
C -3.3417522 -1.0573110 9.3540056
C -4.6822246 -1.0152911 9.7030586
C -5.6595453 -1.2979535 8.7634077
C -5.3065441 -1.6253343 7.4640286
Ni -1.7957987 -1.0539764 5.6896870
C -0.5803360 -2.2103252 9.5579066
C -5.1584774 -3.5374199 5.1608757
H -0.6843414 2.9775630 1.5037919
H 1.0722709 3.4667512 3.1655066
H 1.3896686 1.9986834 5.1133599
H -2.1399136 1.0123407 1.7707321
H -5.0955141 -3.8622782 2.7828566
H -4.5017602 -3.7824172 0.4393816
H -2.5927226 -2.3758236 -0.3182721
H -1.3810836 -1.0106597 1.3065760
H 2.3922567 0.0464353 4.9113357
H 4.3415148 -0.4287457 6.3081479
H 4.0175927 -1.3755598 8.5875601
H 1.7939429 -1.8995190 9.3794392
H -2.5958486 -0.7528573 10.0720416
H -4.9616840 -0.7180654 10.7041249
H -6.7050477 -1.2233429 9.0276931
H -6.0774421 -1.7600915 6.7213222
H -1.5405376 -2.6690738 9.7609089
H 0.1734470 -2.9927179 9.6018535
H -0.3576352 -1.5084071 10.3620103
H -5.2169413 -3.7405866 6.2233551
H -6.1343261 -3.1875349 4.8223476
H -4.9670738 -4.4811904 4.6557275

NiMe$_2$PhenTAA_2eOX_Quintet:
55 atoms
C -0.3386872 2.2081689 2.1635693
C 0.6469994 2.4880665 3.1012976
C 0.8035186 1.6829151 4.2188681
C -0.0052897 0.5603619 4.4007057
C -1.0248145 0.2688344 3.4274861
C -1.1793028 1.1192201 2.3317936
N -1.8717838 -0.7691680 3.7613584
N -0.0299365 -0.2374537 5.5277654
C 0.9803754 -0.4961570 6.3841345
C -2.6214613 -1.5257474 2.9333476
C -3.7016077 -2.3480296 3.4466771
C -4.4518383 -3.0391917 2.4922944
C -4.1701846 -3.0207005 1.1350553
C -3.0622300 -2.3233858 0.6680747
C -2.3081839 -1.5959999 1.5528387
C 2.3235753 -0.2831580 5.9854349
C 3.3813879 -0.5069846 6.8290093
C 3.1348801 -0.9588214 8.1199570
C 1.8378503 -1.2528110 8.5123553
C 0.7237885 -1.0823118 7.6872790
C -4.1099450 -2.5173518 4.8526900
C -0.5762550 -1.4990712 8.2442122
N -1.6998854 -1.2562346 7.6204021
N -3.5801535 -1.8041247 5.8130549
C -4.0063809 -1.7182680 7.1474724
C -3.0041818 -1.4250933 8.1113564
C -3.3817180 -1.1573490 9.4252287
C -4.7132642 -1.1976735 9.7946355
C -5.6923460 -1.4836683 8.8532302
C -5.3435054 -1.7303755 7.5385941
Ni -1.8082152 -0.9927479 5.6875417
C -0.5682559 -2.2804655 9.5199778
C -5.1054680 -3.5903670 5.1616670
H -0.4765711 2.8605718 1.3124784
H 1.2759746 3.3592113 2.9810508
H 1.5167070 1.9575378 4.9826698
H -1.9948504 0.9591086 1.6414223
H -5.3045271 -3.6176664 2.8094488
H -4.7961493 -3.5796813 0.4533362
H -2.7858752 -2.3622287 -0.3766445
H -1.4139006 -1.0971267 1.2093615
H 2.5085025 0.0145627 4.9636722
H 4.3935859 -0.3461775 6.4845033
H 3.9499366 -1.1177653 8.8121704
H 1.7002165 -1.6282371 9.5136494
H -2.6355497 -0.8655640 10.1491929
H -4.9905140 -0.9748293 10.8155341
H -6.7353389 -1.4851363 9.1374157
H -6.1169691 -1.8818684 6.8008776
H -1.5161477 -2.7851698 9.6725264
H 0.2159201 -3.0329804 9.5004783
H -0.3842565 -1.6494685 10.3904704
H -5.1040403 -3.8295213 6.2193344
H -6.1213861 -3.3034571 4.8867550
H -4.8702481 -4.4969703 4.6099317

NiMe$_2$PhenTAA_3eOX_Doublet:
55 atoms
C -0.5288231 2.2735237 2.3342703
C 0.4746846 2.5503958 3.2793688
C 0.6764906 1.7266795 4.3654081
C -0.1254749 0.5881439 4.5240469
C -1.1545055 0.2998795 3.5469735
C -1.3397532 1.1671640 2.4619757
N -1.8944257 -0.8058790 3.8461946
N -0.0985490 -0.2967945 5.5618736
C 0.9500532 -0.5787577 6.3646919
C -2.5862401 -1.5789188 2.9827752
C -3.6118075 -2.4825170 3.4583103
C -4.2868236 -3.2187577 2.4879500
C -3.9689632 -3.1574324 1.1357599
C -2.9218533 -2.3488430 0.6957638
C -2.2490852 -1.5744785 1.6024617
C 2.2722428 -0.3081033 5.9215717
C 3.3632010 -0.5875324 6.7005421
C 3.1717652 -1.1435606 7.9645447
C 1.8918196 -1.4626861 8.4038729
C 0.7507069 -1.2379406 7.6378311
C -4.0625608 -2.5915600 4.8454519
C -0.5488065 -1.5770593 8.2186628
N -1.6541555 -1.3708373 7.5467786
N -3.4791877 -1.9000328 5.7932891
C -3.9626360 -1.6957269 7.0970951
C -2.9642465 -1.4037326 8.0572729
C -3.3340046 -1.0422906 9.3488369
C -4.6754755 -0.9964546 9.6919507
C -5.6561661 -1.2840027 8.7487730
C -5.3059668 -1.6195014 7.4512504
Ni -1.7859347 -1.0767446 5.6869380
C -0.5761560 -2.2086245 9.5697838
C -5.1640331 -3.5477434 5.1578967
H -0.7001854 2.9692907 1.5232452
H 1.0616381 3.4543856 3.1802483
H 1.3912404 1.9983506 5.1292641
H -2.1616946 1.0124201 1.7772781
H -5.0980608 -3.8678750 2.7774808
H -4.5192250 -3.7685147 0.4321085
H -2.6223452 -2.3541446 -0.3436864
H -1.3879683 -1.0063485 1.2831455
H 2.4151842 0.0498242 4.9125407
H 4.3613323 -0.4057939 6.3254779
H 4.0210668 -1.3619344 8.5990932
H 1.7997061 -1.9003593 9.3853560
H -2.5910218 -0.7397839 10.0706901
H -4.9591061 -0.6942243 10.6910064
H -6.7016296 -1.2063730 9.0149382
H -6.0792141 -1.7614703 6.7124067
H -1.5365337 -2.6644852 9.7792767
H 0.1755954 -2.9935152 9.6215115
H -0.3504964 -1.4986988 10.3668368

H -5.2272869 -3.7515999 6.2201871
H -6.1372675 -3.1921962 4.8158216
H -4.9770579 -4.4942276 4.6546063

NiMe$_2$PhenTAA_3eOX_Quartet:
55 atoms
C -0.5171834 2.2796110 2.3332828
C 0.4746414 2.5535961 3.2690516
C 0.6703236 1.7166660 4.3609548
C -0.1136477 0.5735440 4.5004589
C -1.1277962 0.2900563 3.5374223
C -1.3305669 1.1616188 2.4700015
N -1.9002515 -0.8142301 3.8576618
N -0.1072245 -0.3076793 5.5696588
C 0.9448239 -0.5720442 6.3855770
C -2.6102675 -1.5746438 2.9871168
C -3.6527410 -2.4661031 3.4483496
C -4.3495672 -3.1960964 2.4694388
C -4.0446156 -3.1292194 1.1227185
C -2.9799633 -2.3383536 0.7019423
C -2.2789410 -1.5807060 1.6177574
C 2.2596556 -0.3117419 5.9523348
C 3.3546706 -0.5653910 6.7531100
C 3.1764724 -1.0962380 8.0267915
C 1.9030208 -1.4227216 8.4544778
C 0.7555191 -1.2109210 7.6704641
C -4.0684707 -2.6140461 4.8198290
C -0.5192938 -1.5877287 8.2283468
N -1.6417946 -1.4094143 7.5372754
N -3.4597354 -1.9376269 5.7913245
C -3.9494850 -1.6989265 7.0630090
C -2.9311836 -1.3985845 8.0413380
C -3.3045338 -1.0114500 9.3277406
C -4.6418977 -0.9535978 9.6575699
C -5.6349858 -1.2496564 8.7046279
C -5.2953702 -1.6025003 7.4162060
Ni -1.7625214 -1.1394709 5.6804505
C -0.5682263 -2.2620055 9.5574552
C -5.1322563 -3.6012727 5.1619708
H -0.6868283 2.9629509 1.5121007
H 1.0739906 3.4489644 3.1725126
H 1.3861808 1.9872880 5.1239847
H -2.1507461 1.0063362 1.7835814
H -5.1644862 -3.8377595 2.7671167
H -4.6107194 -3.7142607 0.4110696
H -2.6833492 -2.3272553 -0.3389981
H -1.4132411 -1.0244440 1.2905700
H 2.4136076 0.0373022 4.9419812
H 4.3498635 -0.3665668 6.3768542
H 4.0261146 -1.2843701 8.6687804
H 1.8011538 -1.8556093 9.4377362
H -2.5602696 -0.7109074 10.0493566
H -4.9352177 -0.6417010 10.6515965
H -6.6782608 -1.1626211 8.9792201
H -6.0690735 -1.7529957 6.6792564
H -1.5329412 -2.7169247 9.7480353
H 0.1805294 -3.0503943 9.5999473
H -0.3434753 -1.5716302 10.3725874
H -5.1735771 -3.8020161 6.2258700
H -6.1187295 -3.2690885 4.8326062
H -4.9351319 -4.5450186 4.6575198

NiMe$_2$PhenTAA_3eOX_Sextet:
55 atoms
C -0.4163624 2.2337607 2.2395113
C 0.5762563 2.5091504 3.1768422
C 0.7582760 1.6809850 4.2752204
C -0.0212154 0.5323447 4.4320525
C -1.0493122 0.2434115 3.4550219
C -1.2372937 1.1255931 2.3880870
N -1.8644660 -0.8235136 3.7570257
N -0.0083010 -0.2950963 5.5321238
C 1.0011393 -0.5222684 6.4021048
C -2.6381830 -1.5511588 2.9214677
C -3.7265341 -2.3830551 3.4258530
C -4.4838005 -3.0663518 2.4593678
C -4.2083512 -3.0246197 1.1041263
C -3.1004459 -2.3141605 0.6485108
C -2.3326192 -1.6038063 1.5437720
C 2.3375923 -0.2979076 6.0045143
C 3.4004684 -0.4989910 6.8559946
C 3.1669996 -0.9527511 8.1517257
C 1.8772055 -1.2621579 8.5467660
C 0.7536102 -1.1046444 7.7180579
C -4.1207244 -2.5792946 4.8116056
C -0.5221651 -1.5351225 8.2701634
N -1.6577888 -1.3510767 7.6059645
N -3.5407400 -1.9009227 5.7965057
C -3.9770638 -1.7268156 7.0867354
C -2.9465516 -1.4210586 8.0778130
C -3.3355147 -1.0842275 9.3789153
C -4.6664857 -1.0735422 9.7212948
C -5.6618840 -1.3744143 8.7657362
C -5.3227311 -1.6798292 7.4698666
Ni -1.7322626 -1.1525185 5.6553223
C -0.5368734 -2.2901778 9.5574927
C -5.1209472 -3.6378306 5.1371347
H -0.5717150 2.9041834 1.4048099
H 1.1902043 3.3933017 3.0687776
H 1.4733899 1.9538656 5.0384808
H -2.0574574 0.9713362 1.7011269
H -5.3347921 -3.6504195 2.7729391
H -4.8349786 -3.5702697 0.4117593
H -2.8345253 -2.3246945 -0.4005430
H -1.4386390 -1.1033414 1.1997056
H 2.5229873 -0.0029502 4.9814244
H 4.4097985 -0.3129588 6.5126743
H 3.9884366 -1.0960617 8.8403732
H 1.7445935 -1.6352709 9.5501889
H -2.5917401 -0.7915063 10.1047591
H -4.9600250 -0.8070307 10.7284615
H -6.7043764 -1.3378867 9.0543036
H -6.0973994 -1.8425510 6.7360157
H -1.4911436 -2.7780857 9.7228733
H 0.2356882 -3.0557607 9.5637609
H -0.3402424 -1.6373159 10.4106128
H -5.1293225 -3.8659323 6.1969982
H -6.1312879 -3.3402458 4.8480258
H -4.8983840 -4.5563366 4.5987761

NiPh$_2$PhenTAA:
NiPh$_2$PhenTAA_2eRED_CSS:
69 atoms
C -0.5601719 -2.9576794 0.7647859
C 0.6134292 -3.2864753 1.4377513
C 0.7931618 -2.8841461 2.7526609
C -0.1822203 -2.1401991 3.4296860
C -1.3929983 -1.8018272 2.7360194
C -1.5474969 -2.2282379 1.4105075
N -2.3464406 -1.1934657 3.5171067
N -0.1743147 -1.8002678 4.7609398
C 0.9332188 -1.5736937 5.5232261
C -3.3421602 -0.3762086 3.0717676
C -4.4947992 -0.0764778 3.9043906
C -5.4026153 0.8796399 3.3782070
C -5.2727259 1.4767246 2.1466416
C -4.1576528 1.1831911 1.3577248
C -3.2213221 0.2945978 1.8349293
C 2.1586791 -1.2125178 4.9212522
C 3.2932075 -0.9002675 5.6353297
C 3.2338558 -0.8996201 7.0312476
C 2.0470482 -1.2046464 7.6542909
C 0.8583926 -1.5770533 6.9743791
C -4.7020753 -0.6044410 5.2260946
C -0.3409547 -1.8308729 7.7264962
N -1.5454359 -1.9437468 7.1068734
N -3.7357361 -1.3264081 5.8535885
C -3.9028727 -2.1466821 6.9388374
C -2.6865398 -2.4912795 7.6331047
C -2.7576919 -3.4136871 8.6916885
C -3.9553441 -3.9821201 9.0724637
C -5.1357101 -3.6465621 8.4002157
C -5.1041324 -2.7472679 7.3546738
Ni -1.9311379 -1.4407608 5.3371643
C -0.3206835 -1.7842313 9.1945924
C 0.5820372 -2.5249327 9.9802262
C 0.5480877 -2.4870994 11.3629647
C -0.3898746 -1.7076551 12.0309816
C -1.2999451 -0.9718733 11.2732059
C -1.2693459 -1.0145506 9.8945043
C -5.8867311 -0.2137253 6.0014705
C -5.7491389 0.2640511 7.3185042
C -6.8417042 0.6191700 8.0827230
C -8.1355370 0.5036993 7.5763521
C -8.2986296 0.0172617 6.2839837
C -7.2023744 -0.3326419 5.5158832
H -0.7191860 -3.2906133 -0.2546933
H 1.3790447 -3.8781572 0.9484865
H 1.6824193 -3.1873848 3.2894872
H -2.4801208 -2.0196457 0.9027313
H -6.2364863 1.1822208 3.9935710
H -6.0149355 2.1952721 1.8169857
H -4.0035112 1.6722117 0.4020567
H -2.3197170 0.1221271 1.2633437

H 2.1789190 -1.1408524
3.8427761
H 4.2026240 -0.6217831
5.1145097
H 4.0984944 -0.6261531
7.6253407
H 2.0134590 -1.1263153
8.7306527
H -1.8462059 -3.7136682
9.1887775
H -3.9697157 -4.7130087
9.8729081
H -6.0742014 -4.1142727
8.6744183
H -6.0150988 -2.5297645
6.8154037
H 1.3065482 -3.1561556
9.4803042
H 1.2568718 -3.0838729
11.9275861
H -0.4204849 -1.6821199
13.1132149
H -2.0423140 -0.3560015
11.7683180
H -1.9969910 -0.4514115
9.3226349
H -4.7517342 0.3447369
7.7338289
H -6.6866891 0.9948974
9.0878503
H -8.9932266 0.7757426
8.1790394
H -9.2953762 -0.1017869
5.8724182
H -7.3519905 -0.7282550
4.5186128

NiPh$_2$PhenTAA_2eRED_Triplet:
69 atoms
C -0.6318901 -3.0798789
0.8206591
C 0.5361894 -3.4139223
1.4912177
C 0.7337404 -2.9815240
2.7984456
C -0.2228634 -2.2059874
3.4601927
C -1.4293943 -1.8639851
2.7695183
C -1.6034089 -2.3146028
1.4577432
N -2.3621170 -1.2106334
3.5489900
N -0.1997956 -1.8175479
4.7839385
C 0.9151235 -1.5539522
5.5235251
C -3.3239122 -0.3557175
3.0992876
C -4.4443195 0.0149746
3.9409789
C -5.2973775 1.0239451
3.4379513
C -5.1664853 1.5999748
2.1920790
C -4.1043411 1.2132599
1.3781333
C -3.2088377 0.2714113
1.8397744
C 2.1475412 -1.2550243
4.9026656
C 3.2777490 -0.8851162
5.6012735
C 3.2141794 -0.7648938
6.9873660
C 2.0212857 -1.0305851
7.6254682
C 0.8496950 -1.4720423
6.9695292
C -4.6895022 -0.5245594
5.2628425
C -0.3383831 -1.7416382
7.7528463
N -1.5569668 -1.8103946
7.1575424
N -3.7165289 -1.2028882
5.9251513
C -3.9014420 -2.0712084
6.9922892
C -2.6938614 -2.4115597
7.6803750
C -2.7451968 -3.3605497
8.7059494
C -3.9456239 -3.9703182
9.0595382
C -5.1148850 -3.6390206
8.3956296
C -5.0935199 -2.6961230
7.3715596
Ni -1.9338317 -1.3944325
5.3671960
C -0.2658319 -1.7561001
9.2138474
C 0.7075457 -2.4807881
9.9289574
C 0.7133243 -2.5311045
11.3093250
C -0.2546093 -1.8523596
12.0511784
C -1.2283607 -1.1315656
11.3656522
C -1.2396528 -1.0868618
9.9856816
C -5.9199434 -0.1768455
5.9735737
C -5.8803330 0.2216003
7.3266833
C -7.0320922 0.5169903
8.0292348
C -8.2836956 0.4167401
7.4282601
C -8.3491419 0.0068984
6.0958457
C -7.1973474 -0.2792396
5.3893813
H -0.8023437 -3.4277128 -
0.1920092
H 1.2901128 -4.0267205
1.0098759
H 1.6235194 -3.2850565
3.3338651
H -2.5334707 -2.0972460
0.9493176
H -6.0897262 1.3827710
4.0782569
H -5.8633380 2.3675542
1.8754448
H -3.9512446 1.6704338
0.4063718
H -2.3426908 0.0317648
1.2392485
H 2.1797727 -1.2688214
3.8227499
H 4.1910544 -0.6532530
5.0636677
H 4.0724121 -0.4343385
7.5604273
H 1.9734928 -0.8700894
8.6926023
H -1.8336583 -3.6538665
9.2054976
H -3.9529549 -4.7122347
9.8500698
H -6.0516758 -4.1168631
8.6584996
H -6.0054838 -2.4741071
6.8373251
H 1.4553572 -3.0310832
9.3706570
H 1.4737561 -3.1150480
11.8167526
H -0.2554683 -1.8958643
13.1333618
H -1.9929979 -0.5967182
11.9182591
H -2.0121907 -0.5298718
9.4702401
H -4.9160892 0.2954476
7.8143279
H -6.9538501 0.8319052
9.0640441
H -9.1860277 0.6402706
7.9842178
H -9.3125172 -0.0993259
5.6083086
H -7.2696306 -0.6137504
4.3613423

NiPh$_2$PhenTAA_2eRED_Quintet:
69 atoms
C -0.6893991 -3.2022884
0.9247584
C 0.4834734 -3.5096896
1.5990823
C 0.6941104 -3.0141992
2.8805546
C -0.2441505 -2.1967214
3.5201101
C -1.4697673 -1.8799574
2.8195527
C -1.6498637 -2.4030785
1.5339648
N -2.4036793 -1.1856645
3.5522428
N -0.1819414 -1.7558859
4.8224694
C 0.9354335 -1.5107025
5.5670420
C -3.3730770 -0.3479921
3.0899723
C -4.4688711 0.0902102
3.9442229
C -5.2842017 1.1178985
3.4243893
C -5.1662310 1.6505627
2.1551924
C -4.1521760 1.1838928
1.3274453
C -3.2868950 0.2164672
1.7971279
C 2.1735192 -1.2458882
4.9412892
C 3.3037764 -0.8447590
5.6250848
C 3.2398789 -0.6760528
7.0027989
C 2.0450541 -0.9275319
7.6484919
C 0.8718025 -1.3926149
7.0165647
C -4.7693744 -0.3973466
5.2937540
C -0.2950719 -1.6600985
7.8626473
N -1.5506612 -1.5624933
7.3520707
N -3.7847727 -0.9216776
6.0721063
C -3.9243568 -1.8287379
7.1092060
C -2.7014148 -2.1782682
7.8114738
C -2.7619933 -3.1935786
8.7794862
C -3.9500222 -3.8404112
9.0929835
C -5.1172039 -3.5045272
8.4257392
C -5.0975745 -2.5242624
7.4423250
Ni -1.9593387 -1.2971718
5.4493070
C -0.1024103 -1.8285221
9.2998414
C 0.9365900 -2.6051553
9.8526114
C 1.0724053 -2.7839338
11.2143031
C 0.1775281 -2.1865740
12.1054224
C -0.8558094 -1.4133682
11.5840000
C -0.9981019 -1.2394568
10.2213077
C -6.0808126 -0.1224576
5.8711231
C -6.2005422 0.2707470
7.2236561
C -7.4305820 0.5100827
7.8034630
C -8.6084016 0.3615283
7.0763054
C -8.5162494 -0.0357678
5.7400372
C -7.2873608 -0.2653326
5.1554888
H -0.8710336 -3.5963874 -
0.0690076
H 1.2301837 -4.1486460
1.1409800
H 1.5877142 -3.2975060
3.4214633
H -2.5847813 -2.2091800
1.0242249
H -6.0433406 1.5297860
4.0740579
H -5.8342381 2.4414507
1.8344858
H -4.0100753 1.5947224
0.3333460
H -2.4535464 -0.0849280
1.1783642
H 2.2054609 -1.3000412
3.8623526
H 4.2164609 -0.6311135
5.0785326
H 4.0949562 -0.3204807
7.5654449
H 1.9981455 -0.7376152
8.7111636
H -1.8502251 -3.5111053
9.2617554
H -3.9501795 -4.6194172
9.8473141
H -6.0474759 -4.0139802
8.6509556
H -6.0020411 -2.3205205
6.8894465
H 1.6340812 -3.0865726
9.1775633
H 1.8784108 -3.4048036
11.5907605
H 0.2806973 -2.3284099
13.1741937
H -1.5632530 -0.9373773
12.2543798
H -1.8139735 -0.6416829
9.8359834
H -5.2960885 0.3839596
7.8077144
H -7.4747288 0.8201402
8.8421089
H -9.5720564 0.5421639
7.5367493
H -9.4182582 -0.1770178
5.1538073
H -7.2370796 -0.5836592
4.1208446

NiPh$_2$PhenTAA_1eRED_Doublet:
69 atoms
C -0.5771065 -2.9749995
0.7704677
C 0.5924041 -3.3113649
1.4414473
C 0.7731619 -2.9222501
2.7592057
C -0.2022125 -2.1793250
3.4295022
C -1.4021453 -1.8368137
2.7425174
C -1.5654232 -2.2504314
1.4179781
N -2.3568410 -1.2282629
3.5371089
N -0.1997568 -1.8391590
4.7699242
C 0.8979117 -1.5609086
5.5153135

C -3.3095120 -0.3647806 3.1087554
C -4.4362667 -0.0196426 3.9482432
C -5.3270861 0.9628964 3.4521198
C -5.1920056 1.5635578 2.2255942
C -4.0977729 1.2283258 1.4246552
C -3.1887985 0.3000719 1.8653422
C 2.1308267 -1.2242792 4.9072179
C 3.2440495 -0.8670306 5.6252849
C 3.1830624 -0.8085770 7.0195306
C 1.9974715 -1.1024825 7.6448504
C 0.8257597 -1.5040777 6.9594167
C -4.6685320 -0.5718241 5.2567502
C -0.3631517 -1.7798371 7.7215339
N -1.5607673 -1.9117960 7.1375610
N -3.7396312 -1.2976792 5.8926165
C -3.9230654 -2.0807383 7.0250907
C -2.7181058 -2.4194966 7.7138691
C -2.7839934 -3.3170845 8.7865745
C -3.9878942 -3.8608584 9.1895369
C -5.1619167 -3.5314753 8.5181310
C -5.1260575 -2.6595880 7.4475687
Ni -1.9488145 -1.4912415 5.3459731
C -0.2812833 -1.7987506 9.1989279
C 0.5849202 -2.6575917 9.8822743
C 0.6111414 -2.6964442 11.2656099
C -0.2238243 -1.8698539 12.0054104
C -1.0828079 -1.0037882 11.3412512
C -1.1135840 -0.9715681 9.9588807
C -5.9195743 -0.2249468 5.9666221
C -5.8695129 0.3543811 7.2378223
C -7.0273757 0.6556024 7.9318331
C -8.2707293 0.3794573 7.3778548
C -8.3382472 -0.1948912 6.1157587
C -7.1789031 -0.4891191 5.4191949
H -0.7346365 -3.3020964 -0.2497307
H 1.3543061 -3.9025911 0.9488050
H 1.6581193 -3.2359461 3.2959752
H -2.4982581 -2.0401785 0.9124230
H -6.1456249 1.2766124 4.0827878
H -5.9066301 2.3113528 1.9067107
H -3.9393012 1.7213057 0.4726384
H -2.3098388 0.0979903 1.2706351
H 2.1688607 -1.1992135 3.8282803
H 4.1539017 -0.5974261 5.1021445
H 4.0424408 -0.5029766 7.6016966
H 1.9501903 -0.9972732 8.7186169
H -1.8751977 -3.6271230 9.2791850
H -4.0047865 -4.5705061 10.0070757
H -6.1030497 -3.9822504 8.8062520
H -6.0356989 -2.4598307 6.9019617
H 1.2331611 -3.3084205 9.3080824
H 1.2839903 -3.3798114 11.7690373
H -0.2059798 -1.9000817 13.0872918
H -1.7350855 -0.3491831 11.9061941
H -1.7984033 -0.3073023 9.4462156
H -4.9021328 0.5567278 7.6812059
H -6.9602854 1.1090690 8.9131405
H -9.1770350 0.6098097 7.9233234
H -9.3007163 -0.4192534 5.6723372
H -7.2353202 -0.9417378 4.4365931

NiPh$_2$PhenTAA_1eRED_Quartet:
69 atoms
C -0.6279647 -3.0653911 0.8395984
C 0.5509756 -3.3910998 1.5166526
C 0.7585456 -2.9580485 2.8101683
C -0.2094395 -2.1820302 3.4692855
C -1.4275876 -1.8460612 2.7703241
C -1.6025642 -2.3061363 1.4547303
N -2.3238237 -1.1599834 3.5263187
N -0.1778174 -1.7503982 4.7561165
C 0.9452391 -1.5258820 5.5236204
C -3.3338476 -0.3431678 3.0660952
C -4.4538891 -0.0173995 3.9135061
C -5.3445877 0.9537706 3.4053672
C -5.2159240 1.5359280 2.1621265
C -4.1363030 1.2037510 1.3537302
C -3.2084400 0.2948575 1.8244684
C 2.1597116 -1.1895487 4.9100839
C 3.2902455 -0.8479361 5.6272282
C 3.2113287 -0.7893908 7.0127277
C 2.0200213 -1.0816291 7.6422178
C 0.8516633 -1.4880951 6.9616317
C -4.6723820 -0.5569267 5.2419547
C -0.3520883 -1.7645980 7.7222524
N -1.5493100 -1.8868401 7.1098611
N -3.7180438 -1.2797167 5.8666113
C -3.8958475 -2.1375314 6.9444680
C -2.6937750 -2.4780923 7.6298474
C -2.7433899 -3.4232522 8.6577197
C -3.9452539 -4.0184914 9.0177400
C -5.1149078 -3.6838321 8.3543952
C -5.0913301 -2.7530419 7.3238944
Ni -1.9039806 -1.3201186 5.3447924
C -0.3155596 -1.7474119 9.1925324
C 0.5987707 -2.5074371 9.9360514
C 0.5562523 -2.5364495 11.3179714
C -0.4010878 -1.8023357 12.0093009
C -1.3161940 -1.0439769 11.2904584
C -1.2781452 -1.0201441 9.9091142
C -5.8712803 -0.1741163 6.0039501
C -5.7436939 0.2831282 7.3240252
C -6.8529240 0.5977333 8.0863145
C -8.1319832 0.4546116 7.5640677
C -8.2806203 -0.0114142 6.2628624
C -7.1706092 -0.3181993 5.4971182
H -0.7910255 -3.4315486 -0.1661309
H 1.2975366 -4.0089756 1.0337794
H 1.6498131 -3.2525972 3.3467924
H -2.5332555 -2.0965467 0.9456047
H -6.1603881 1.2783233 4.0327923
H -5.9428142 2.2711767 1.8396416
H -3.9981846 1.6675931 0.3852668
H -2.3227803 0.0879087 1.2397867
H 2.1864513 -1.1606132 3.8298770
H 4.2074230 -0.5990993 5.1087506
H 4.0672640 -0.4873324 7.6029693
H 1.9739241 -0.9693920 8.7147564
H -1.8325220 -3.7145595 9.1593254
H -3.9572603 -4.7531000 9.8132608
H -6.0523961 -4.1528736 8.6251970
H -6.0027582 -2.5242012 6.7916462
H 1.3346819 -3.1018679 9.4084140
H 1.2695512 -3.1449629 11.8608752
H -0.4377284 -1.8282339 13.0906918
H -2.0703621 -0.4680043 11.8128125
H -2.0074397 -0.4397868 9.3573041
H -4.7502431 0.3844821 7.7438256
H -6.7192281 0.9575490 9.0991377
H -9.0005076 0.6952048 8.1636523
H -9.2711530 -0.1451673 5.8449863
H -7.2997571 -0.7000311 4.4916839

NiPh$_2$PhenTAA_1eRED_Sextet:
69 atoms
C -0.8018504 -3.2021850 1.0657798
C 0.3625003 -3.5571769 1.7554630
C 0.6226717 -3.0295431 3.0021844
C -0.2665422 -2.1247599 3.6073541
C -1.4583818 -1.7218087 2.8744950
C -1.6948362 -2.3093586 1.6137190
N -2.2675089 -0.8645923 3.5257087
N -0.1797254 -1.6416774 4.8667504
C 0.9628879 -1.4627626 5.6234538
C -3.3521245 -0.1955251 3.0027557
C -4.4905930 0.1072295 3.8449970
C -5.4364521 0.9975559 3.2777560
C -5.3551256 1.4875828 1.9929741
C -4.2829891 1.1397047 1.1802589
C -3.2997370 0.3186314 1.7021729
C 2.1901249 -1.2390382 4.9832938
C 3.3467008 -0.9161969 5.6686636
C 3.2891269 -0.7774082 7.0488409
C 2.0928037 -0.9773629 7.7052765
C 0.8922193 -1.3563214 7.0642505
C -4.7144244 -0.3977133 5.1813766
C -0.2923817 -1.5847347 7.8761212
N -1.5344523 -1.4644117 7.3229799
N -3.7064194 -0.9909804 5.9069389
C -3.8581162 -1.8876693 6.9422338
C -2.6601035 -2.1672020 7.6998357
C -2.7192523 -3.1936991 8.6555356
C -3.8932112 -3.8855686 8.9076097
C -5.0493926 -3.5907857 8.2000217
C -5.0247114 -2.6103434 7.2220794
Ni -1.8370295 -0.8304973 5.4674140
C -0.1559009 -1.7706242 9.3206728
C 0.8168802 -2.6065393 9.8952095
C 0.8769052 -2.8152123 11.2594420
C -0.0290909 -2.1900166 12.1113767
C -0.9962867 -1.3551123 11.5652023
C -1.0620470 -1.1507427 10.2003485

C -5.9748365 -0.1052274 5.8772566
C -5.9537097 0.3476818 7.2063621
C -7.1221515 0.5771907 7.9080885
C -8.3581546 0.3500491 7.3173729
C -8.4021165 -0.1127358 6.0068414
C -7.2341842 -0.3328392 5.3008872
H -1.0172692 -3.6518059 0.1045487
H 1.0468684 -4.2768891 1.3244196
H 1.4905651 -3.3538090 3.5600337
H -2.6159811 -2.0773722 1.0973294
H -6.2552991 1.3335664 3.8949555
H -6.1172627 2.1698844 1.6364432
H -4.1875625 1.5362860 0.1777375
H -2.4117354 0.1106070 1.1203292
H 2.2063116 -1.2758409 3.9027409
H 4.2674427 -0.7404935 5.1265610
H 4.1653671 -0.4813680 7.6118638
H 2.0630753 -0.8117764 8.7721816
H -1.8237176 -3.4806578 9.1833348
H -3.8937559 -4.6725806 9.6520576
H -5.9648545 -4.1380441 8.3873142
H -5.9098857 -2.4278692 6.6310794
H 1.5148039 -3.1171585 9.2427156
H 1.6286602 -3.4822763 11.6645582
H 0.0148701 -2.3574957 13.1798327
H -1.7095311 -0.8595293 12.2129156
H -1.8291832 -0.5106134 9.7832961
H -4.9953516 0.5132723 7.6825560
H -7.0673572 0.9347504 8.9292509
H -9.2727476 0.5219544 7.8705053
H -9.3563159 -0.3151970 5.5351874
H -7.2841356 -0.7157978 4.2888972

$NiPh_2PhenTAA\_0eNeutral\_CSS$:
69 atoms
C -0.5923137 -3.0095463 0.7853127
C 0.5748610 -3.3436825 1.4548688
C 0.7590228 -2.9532779 2.7724488
C -0.2155460 -2.2059353 3.4291525
C -1.4074789 -1.8671331 2.7467444
C -1.5827380 -2.2837753 1.4296117
N -2.3579839 -1.2439103 3.5535962
N -0.2193972 -1.8464130 4.7756204
C 0.8647122 -1.5384823 5.5150582
C -3.2889426 -0.3621689 3.1391371
C -4.3817712 0.0332911 3.9959491
C -5.2597566 1.0392627 3.5277994
C -5.1338347 1.6293515 2.2994693
C -4.0787774 1.2359471 1.4707047
C -3.1957454 0.2721736 1.8743140
C 2.1141769 -1.2448353 4.9118579
C 3.2016312 -0.8400780 5.6364941
C 3.1253814 -0.7087322 7.0262309
C 1.9378950 -0.9859003 7.6469496
C 0.7843651 -1.4191542 6.9520427
C -4.6319383 -0.5444406 5.2880943
C -0.3934842 -1.7288057 7.7147644
N -1.5737698 -1.8868779 7.1457182
N -3.7319556 -1.2818580 5.9115493
C -3.9345711 -2.0640655 7.0694712
C -2.7427795 -2.3977499 7.7510481
C -2.7960557 -3.2856205 8.8218743
C -4.0053559 -3.8232985 9.2307032
C -5.1725922 -3.4966131 8.5632798
C -5.1371045 -2.6301607 7.4831965
Ni -1.9616804 -1.5371528 5.3448040
C -0.2739372 -1.8058363 9.1925655
C 0.5809623 -2.7200256 9.8053671
C 0.6455257 -2.8061175 11.1855051
C -0.1290557 -1.9657875 11.9719686
C -0.9658057 -1.0375668 11.3707270
C -1.0426790 -0.9616533 9.9906971
C -5.9210468 -0.2327918 5.9550708
C -5.9183762 0.4013657 7.1953579
C -7.1092750 0.6889472 7.8399768
C -8.3181981 0.3346515 7.2600390
C -8.3311071 -0.2945486 6.0237603
C -7.1412243 -0.5680990 5.3708949
H -0.7512156 -3.3432385 -0.2313727
H 1.3328222 -3.9395944 0.9642347
H 1.6401340 -3.2732426 3.3103775
H -2.5179266 -2.0828495 0.9260585
H -6.0508260 1.3747721 4.1819170
H -5.8206210 2.4055709 1.9927182
H -3.9303774 1.7185838 0.5124151
H -2.3510929 0.0336102 1.2465388
H 2.1788512 -1.2725742 3.8353947
H 4.1173457 -0.5870627 5.1164245
H 3.9757199 -0.3677282 7.5990809
H 1.8688658 -0.8438451 8.7152034
H -1.8887314 -3.5959597 9.3144053
H -4.0228380 -4.5256921 10.0531847
H -6.1142917 -3.9405944 8.8564978
H -6.0461211 -2.4322699 6.9380448
H 1.1880337 -3.3704939 9.1879838
H 1.3050700 -3.5282797 11.6482425
H -0.0754953 -2.0296876 13.0507019
H -1.5632128 -0.3714295 11.9792705
H -1.7071504 -0.2483268 9.5194051
H -4.9726208 0.6648788 7.6523582
H -7.0938056 1.1905435 8.7985510
H -9.2488553 0.5546902 7.7662019
H -9.2714278 -0.5686893 5.5637185
H -7.1474882 -1.0529873 4.4026475

$NiPh_2PhenTAA\_0eNeutral\_Triplet$:
69 atoms
C -0.6005187 -3.0011894 0.8000123
C 0.5815652 -3.3328468 1.4774050
C 0.7776416 -2.9374715 2.7790225
C -0.2039846 -2.1884410 3.4501478
C -1.4209333 -1.8480633 2.7535265
C -1.5859707 -2.2744049 1.4247968
N -2.3319431 -1.1926637 3.5198021
N -0.1819079 -1.7928519 4.7494424
C 0.9208493 -1.5363042 5.5133606
C -3.3134243 -0.3477385 3.0877852
C -4.4237862 -0.0170765 3.9413421
C -5.3206931 0.9587778 3.4598010
C -5.1889583 1.5674395 2.2333754
C -4.1059961 1.2444689 1.4212417
C -3.1888563 0.3166935 1.8530916
C 2.1421549 -1.1844832 4.9083468
C 3.2493735 -0.8264639 5.6396053
C 3.1693470 -0.7806071 7.0281392
C 1.9810194 -1.0907990 7.6473489
C 0.8236032 -1.4898575 6.9482718
C -4.6567692 -0.5780776 5.2605269
C -0.3767324 -1.7804888 7.7123113
N -1.5524914 -1.9537985 7.1261347
N -3.7527207 -1.3331112 5.8687017
C -3.9215058 -2.1139015 7.0063894
C -2.7195585 -2.4537831 7.6925283
C -2.7820890 -3.3549344 8.7637942
C -3.9839271 -3.8962029 9.1615871
C -5.1600053 -3.5621015 8.4920539
C -5.1278314 -2.6893394 7.4276023
Ni -1.9304385 -1.4434660 5.3346212
C -0.2951721 -1.7944388 9.1929581
C 0.5446041 -2.6783420 9.8719093
C 0.5594699 -2.7188574 11.2556778
C -0.2560419 -1.8674580 11.9867622
C -1.0855595 -0.9753237 11.3226128
C -1.1094009 -0.9429497 9.9395331
C -5.9041614 -0.2174783 5.9766512
C -5.8326289 0.3925669 7.2288294
C -6.9838371 0.6985775 7.9330271
C -8.2289694 0.3900416 7.4044013
C -8.3136494 -0.2175512 6.1603102
C -7.1624456 -0.5131016 5.4502304
H -0.7537976 -3.3502118 -0.2123661
H 1.3320162 -3.9351015 0.9829358
H 1.6648519 -3.2442580 3.3151276
H -2.5189492 -2.0693431 0.9183131
H -6.1414472 1.2611308 4.0920145
H -5.9092538 2.3122481 1.9232295
H -3.9627866 1.7382720 0.4688401
H -2.3099804 0.1173775 1.2568771
H 2.1819151 -1.1508597 3.8293807
H 4.1644861 -0.5536317 5.1305432
H 4.0212600 -0.4762366 7.6203469
H 1.9281338 -1.0031641 8.7217001
H -1.8731133 -3.6616680 9.2574601
H -4.0042730 -4.6054486 9.9782505
H -6.1000967 -4.0100155 8.7848433
H -6.0378554 -2.4798060 6.8871069
H 1.1789663 -3.3461737 9.3018295
H 1.2095861 -3.4188113 11.7642089
H -0.2435261 -1.8970117 13.0681410
H -1.7184459 -0.3016557 11.8856393
H -1.7704263 -0.2577268 9.4230262
H -4.8607367 0.6194448 7.6501160

H -6.9096304 1.1798432 8.8996173
H -9.1288930 0.6245013 7.9575509
H -9.2803977 -0.4630760 5.7402698
H -7.2293261 -0.9902156 4.4799788

NiPh$_2$PhenTAA_0eNeutral_Quintet:
69 atoms
C -0.5875404 -3.0246315 0.8920837
C 0.5806093 -3.3355424 1.5932923
C 0.7719800 -2.8722558 2.8779599
C -0.1900366 -2.0705550 3.5037927
C -1.4002455 -1.7402687 2.7769696
C -1.5623425 -2.2487212 1.4743850
N -2.3207107 -1.0438385 3.4881563
N -0.1574025 -1.6481597 4.8050519
C 0.9533449 -1.4475808 5.5793562
C -3.3754020 -0.3126862 3.0076345
C -4.4985426 0.0003700 3.8716657
C -5.4411114 0.8988107 3.3194238
C -5.3526425 1.4242479 2.0484869
C -4.2756665 1.1040572 1.2325540
C -3.3071431 0.2552479 1.7262707
C 2.1903792 -1.1418810 4.9839774
C 3.3023964 -0.8029800 5.7208406
C 3.2197629 -0.7598474 7.1062712
C 2.0215444 -1.0522555 7.7190619
C 0.8544312 -1.4083807 7.0159388
C -4.7411916 -0.5014471 5.2092815
C -0.3393595 -1.7104604 7.8076129
N -1.5389476 -1.7603886 7.2736481
N -3.7634283 -1.1057053 5.9535884
C -3.9289787 -1.8889387 7.0732307
C -2.7379507 -2.2220900 7.8172436
C -2.8446393 -3.0938736 8.9075584
C -4.0547407 -3.6345849 9.2857498
C -5.2037009 -3.3288623 8.5616729
C -5.1402000 -2.4828581 7.4798214
Ni -1.9299536 -1.2361499 5.3875415
C -0.1468320 -1.9034717 9.2691677
C 0.6307140 -2.9473352 9.7678534
C 0.7633938 -3.1366537 11.1331309
C 0.1385820 -2.2718797 12.0197578
C -0.6197547 -1.2173844 11.5325219
C -0.7668925 -1.0380265 10.1676720
C -6.0300503 -0.1882670 5.8577850
C -6.0467779 0.4417531 7.1061675
C -7.2390234 0.7097088 7.7545040
C -8.4473858 0.3473846 7.1770841
C -8.4493057 -0.2793784 5.9383019
C -7.2572139 -0.5392968 5.2861427
H -0.7431171 -3.4253275 -0.1005040
H 1.3272107 -3.9740352 1.1399673
H 1.6492112 -3.1708227 3.4346395
H -2.4938292 -2.0677622 0.9570748
H -6.2657677 1.2182511 3.9366323
H -6.1122986 2.1164881 1.7089916
H -4.1679430 1.5407047 0.2491385
H -2.4163244 0.0716923 1.1425382
H 2.2384152 -1.1110135 3.9052605
H 4.2239244 -0.5462236 5.2148297
H 4.0756598 -0.4773320 7.7035345
H 1.9698957 -0.9812389 8.7951545
H -1.9577205 -3.3977187 9.4382250
H -4.0963119 -4.3219744 10.1197235
H -6.1508055 -3.7810446 8.8257957
H -6.0334917 -2.3107290 6.9018550
H 1.1236711 -3.6175718 9.0743208
H 1.3588266 -3.9599764 11.5056008
H 0.2473890 -2.4160663 13.0864342
H -1.0993028 -0.5319422 12.2189412
H -1.3731329 -0.2253374 9.7870578
H -5.1062423 0.7145996 7.5685266
H -7.2258882 1.2060143 8.7163614
H -9.3797817 0.5525318 7.6860370
H -9.3853279 -0.5718025 5.4798918
H -7.2639431 -1.0363061 4.3237605

NiPh$_2$PhenTAA_1eOX_Doublet:
69 atoms
C -0.6531624 -3.0649221 0.8588292
C 0.5274713 -3.4001484 1.5375358
C 0.7321466 -2.9968314 2.8349417
C -0.2461288 -2.2369801 3.4924417
C -1.4572168 -1.8962955 2.7982765
C -1.6345560 -2.3265618 1.4754593
N -2.3487636 -1.2067484 3.5671602
N -0.2202891 -1.8017958 4.7849625
C 0.8850418 -1.5066350 5.5294757
C -3.2997354 -0.3301473 3.1322870
C -4.3734071 0.0697266 3.9927594
C -5.2429096 1.0716176 3.5276331
C -5.1110613 1.6549977 2.2881798
C -4.0723638 1.2531450 1.4565058
C -3.1893064 0.2845383 1.8716583
C 2.1146697 -1.2158246 4.9106785
C 3.2116643 -0.8065182 5.6314044
C 3.1287760 -0.6639030 7.0114492
C 1.9383177 -0.9405930 7.6435843
C 0.7943339 -1.3773554 6.9537128
C -4.6348827 -0.5131657 5.2995009
C -0.3921419 -1.6986455 7.7307753
N -1.5655871 -1.8446634 7.1638592
N -3.7352733 -1.2363620 5.9223943
C -3.9284032 -2.0395898 7.0692564
C -2.7380006 -2.3740832 7.7495047
C -2.7840349 -3.2821415 8.8053202
C -3.9878461 -3.8390280 9.1917126
C -5.1580089 -3.5091710 8.5244817
C -5.1306333 -2.6213090 7.4663272
Ni -1.9521867 -1.4629110 5.3622242
C -0.2537995 -1.8084524 9.2005879
C 0.6219716 -2.7260164 9.7790565
C 0.7016562 -2.8404837 11.1561745
C -0.0716006 -2.0234051 11.9677065
C -0.9264839 -1.0908390 11.3985271
C -1.0258393 -0.9893079 10.0225010
C -5.9354236 -0.2192290 5.9424287
C -5.9536878 0.4007724 7.1905604
C -7.1575817 0.6786651 7.8129848
C -8.3531042 0.3217555 7.2064341
C -8.3427577 -0.2964193 5.9646808
C -7.1410532 -0.5549126 5.3278895
H -0.8112185 -3.4279239 -0.1472977
H 1.2670820 -4.0188183 1.0483108
H 1.6146411 -3.3121009 3.3726172
H -2.5680735 -2.1258131 0.9697185
H -6.0336543 1.4125998 4.1785636
H -5.7945944 2.4335328 1.9808269
H -3.9296370 1.7231508 0.4923783
H -2.3436050 0.0352359 1.2485979
H 2.1706696 -1.2489335 3.8333447
H 4.1284678 -0.5631318 5.1109622
H 3.9776824 -0.3176116 7.5828771
H 1.8731262 -0.7966598 8.7113586
H -1.8765303 -3.5900939 9.2986157
H -4.0043781 -4.5599186 9.9974292
H -6.0946566 -3.9703288 8.8052789
H -6.0400509 -2.4179620 6.9243652
H 1.2287500 -3.3588292 9.1433308
H 1.3733346 -3.5645700 11.5966282
H -0.0018803 -2.1076578 13.0437435
H -1.5180566 -0.4409472 12.0290960
H -1.7018689 -0.2710712 9.5758236
H -5.0186047 0.6683006 7.6666326
H -7.1634652 1.1760100 8.7733568
H -9.2934525 0.5332902 7.6972154
H -9.2733609 -0.5714335 5.4870295
H -7.1304851 -1.0311374 4.3553728

NiPh$_2$PhenTAA_1eOX_Quartet:
69 atoms
C -0.5736700 -2.9594928 0.7590098
C 0.6152449 -3.2701095 1.4183628
C 0.8161117 -2.8710169 2.7224357
C -0.1647302 -2.1347237 3.3990686
C -1.3926370 -1.8156847 2.7164949
C -1.5693394 -2.2502671 1.4012073
N -2.3445383 -1.1953264 3.4945901
N -0.1388249 -1.7850896 4.7243663
C 0.9500275 -1.5542324 5.5015376
C -3.3277588 -0.3531289 3.0793711
C -4.4272686 -0.0174314 3.9525189
C -5.3240635 0.9606828 3.4883081
C -5.2028810 1.5783040 2.2630292
C -4.1367603 1.2509388 1.4335002
C -3.2228644 0.3104147 1.8394460
C 2.1855405 -1.2090829 4.9131039
C 3.2843828 -0.8721615 5.6622010
C 3.1901363 -0.8505068 7.0493850
C 1.9912481 -1.1560449 7.6543404
C 0.8353445 -1.5217609 6.9419197
C -4.6802649 -0.5793251 5.2815002
C -0.3692455 -1.7987689 7.7263015
N -1.5456416 -1.9607195 7.1654683

N -3.7921096 -1.3195213 5.8993329
C -3.9428246 -2.0674243 7.0783828
C -2.7396909 -2.4028727 7.7619917
C -2.8032911 -3.2762980 8.8508409
C -4.0081467 -3.8010918 9.2689887
C -5.1804483 -3.4771884 8.5995402
C -5.1462776 -2.6271545 7.5140408
Ni -1.9410493 -1.5112467 5.3140503
C -0.2515580 -1.8182198 9.2063396
C 0.5272133 -2.7721935 9.8603869
C 0.5556008 -2.8207829 11.2436205
C -0.1725722 -1.9036797 11.9874541
C -0.9319488 -0.9386696 11.3430342
C -0.9805864 -0.9018688 9.9601489
C -5.9580851 -0.2334776 5.9518301
C -5.9330433 0.4226563 7.1803077
C -7.1138829 0.7117835 7.8419356
C -8.3300698 0.3318449 7.2937304
C -8.3633647 -0.3241038 6.0720954
C -7.1851420 -0.5961800 5.3977941
H -0.7314553 -3.3015196 -0.2546063
H 1.3731301 -3.8546752 0.9155710
H 1.7096848 -3.1732292 3.2495047
H -2.5119231 -2.0661823 0.9051500
H -6.1367721 1.2623111 4.1304912
H -5.9205342 2.3308708 1.9680707
H -4.0050205 1.7514584 0.4833011
H -2.3568779 0.1101277 1.2259559
H 2.2362664 -1.1494513 3.8363053
H 4.2069977 -0.5966478 5.1693104
H 4.0393933 -0.5657549 7.6542138
H 1.9335689 -1.0875962 8.7290502
H -1.8968063 -3.5786701 9.3494880
H -4.0282976 -4.4852747 10.1060529
H -6.1232089 -3.9067354 8.9092402
H -6.0582561 -2.4235259 6.9758241
H 1.0964022 -3.4875197 9.2791934
H 1.1505609 -3.5744543 11.7416185
H -0.1431084 -1.9377101 13.0680003
H -1.4893296 -0.2122333 11.9189515
H -1.5867273 -0.1589636 9.4563571
H -4.9819940 0.7050417 7.6150933
H -7.0854721 1.2343633 8.7884966
H -9.2521146 0.5522760 7.8145305
H -9.3096634 -0.6203298 5.6397069
H -7.2097137 -1.1047531 4.4418162

NiPh$_2$PhenTAA_1eOX_Sextet: *69 atoms*
C -0.6811262 -3.1398997 0.9875430
C 0.4983058 -3.4337974 1.6642208
C 0.7333938 -2.9139445 2.9272922
C -0.1964661 -2.0700274 3.5293033
C -1.4145787 -1.7512859 2.8266090
C -1.6320637 -2.3204428 1.5632430
N -2.3010391 -0.9901311 3.5294670
N -0.1410786 -1.6117710 4.8266409
C 0.9673786 -1.4162651 5.5870544
C -3.3881493 -0.3109245 3.0163841
C -4.5196596 0.0127278 3.8561205
C -5.4611396 0.8904132 3.2784907
C -5.3695454 1.3777424 1.9870548
C -4.2888954 1.0413833 1.1934480
C -3.3082277 0.2211058 1.7258821
C 2.2114260 -1.1409212 4.9819440
C 3.3222973 -0.8149218 5.7171714
C 3.2431878 -0.7461836 7.1075861
C 2.0485895 -1.0102913 7.7313502
C 0.8761780 -1.3619435 7.0320762
C -4.7445741 -0.4320199 5.2137036
C -0.3124320 -1.6487987 7.8144672
N -1.5021034 -1.7066371 7.2400787
N -3.6994300 -0.9952556 5.9702755
C -3.8663989 -1.9130961 6.9491367
C -2.6751156 -2.2626281 7.7147122
C -2.7742124 -3.2306164 8.7167251
C -3.9761650 -3.8492131 8.9838441
C -5.1212951 -3.5320109 8.2419429
C -5.0707538 -2.6002118 7.2405014
Ni -1.8697542 -1.0456138 5.4099029
C -0.1866293 -1.8161801 9.2781872
C 0.6679250 -2.7710679 9.8310651
C 0.7119706 -2.9589796 11.2018349
C -0.0753275 -2.1795997 12.0364337
C -0.9117846 -1.2128828 11.4957993
C -0.9770369 -1.0394140 10.1256999
C -5.9718693 -0.1009018 5.9327451
C -5.9091442 0.3874459 7.2481172
C -7.0596021 0.6583753 7.9632500
C -8.3063256 0.4291254 7.3982060
C -8.3898953 -0.0720658 6.1049403
C -7.2430974 -0.3265895 5.3791581
H -0.8710523 -3.5794096 0.0181924
H 1.2252237 -4.0988472 1.2187532
H 1.6197663 -3.2051010 3.4731366
H -2.5745445 -2.1492929 1.0630438
H -6.2835735 1.2322040 3.8857258
H -6.1338149 2.0495851 1.6206366
H -4.1816188 1.4365857 0.1932952
H -2.4076130 0.0365146 1.1575713
H 2.2588247 -1.1237686 3.9030499
H 4.2495843 -0.5806816 5.2113488
H 4.1056002 -0.4595672 7.6923996
H 1.9958361 -0.9164039 8.8052065
H -1.8945315 -3.5342291 9.2614588
H -4.0235393 -4.6085750 9.7522894
H -6.0511385 -4.0460965 8.4446788
H -5.9473918 -2.4028518 6.6446946
H 1.2823885 -3.3790922 9.1785320
H 1.3656175 -3.7128068 11.6195310
H -0.0328380 -2.3211869 13.1079649
H -1.5153323 -0.5929795 12.1450369
H -1.6394586 -0.2951639 9.7012405
H -4.9409813 0.5711654 7.6972028
H -6.9839674 1.0540757 8.9674493
H -9.2067155 0.6342298 7.9614217
H -9.3565556 -0.2752119 5.6634775
H -7.3189111 -0.7438806 4.3830532

NiPh$_2$PhenTAA_2eOX_CSS: *69 atoms*
C -0.7004263 -3.0962596 0.9214819
C 0.4964378 -3.4326465 1.6114608
C 0.7176338 -3.0218301 2.8906945
C -0.2655893 -2.2505846 3.5525847
C -1.5009889 -1.9067321 2.8429164
C -1.6760205 -2.3516514 1.5121900
N -2.3420992 -1.1690001 3.5722439
N -0.2137527 -1.7588955 4.7926419
C 0.9044505 -1.4872388 5.5436368
C -3.3155093 -0.3113207 3.1214703
C -4.3737877 0.0985438 3.9902387
C -5.2384085 1.0909277 3.5176819
C -5.1078207 1.6559823 2.2616972
C -4.0821166 1.2441025 1.4241909
C -3.1976473 0.2794757 1.8532913
C 2.1256496 -1.2065520 4.9103345
C 3.2302198 -0.7998787 5.6257041
C 3.1400254 -0.6472950 7.0010056
C 1.9444732 -0.9129950 7.6440220
C 0.8031664 -1.3460678 6.9621952
C -4.6416731 -0.4804868 5.3097957
C -0.3889361 -1.6717758 7.7495900
N -1.5603789 -1.8029345 7.1791257
N -3.7358292 -1.1919050 5.9333922
C -3.9222537 -2.0216486 7.0615119
C -2.7312448 -2.3588445 7.7409473
C -2.7697364 -3.2906821 8.7775452
C -3.9684813 -3.8700941 9.1386724
C -5.1418693 -3.5354214 8.4724160
C -5.1231824 -2.6210824 7.4393790
Ni -1.9440269 -1.3920734 5.3788784
C -0.2411203 -1.8041927 9.2100635
C 0.6675669 -2.7059782 9.7679478
C 0.7553244 -2.8405361 11.1422787
C -0.0362274 -2.0565275 11.9692322
C -0.9222732 -1.1390028 11.4204074
C -1.0373929 -1.0218312 10.0481653
C -5.9442135 -0.1977548 5.9386178
C -5.9722696 0.3862540 7.2063233
C -7.1822009 0.6590275 7.8163509
C -8.3720830 0.3240227 7.1837529
C -8.3515363 -0.2631023 5.9266143
C -7.1444407 -0.5095390 5.2963493
H -0.8561356 -3.4876641 -0.0746451
H 1.2185827 -4.0729309 1.1234001
H 1.5964910 -3.3416686 3.4317342
H -2.6119840 -2.1618370 1.0068068
H -6.0267357 1.4437840 4.1647973
H -5.7910110 2.4336988 1.9502335
H -3.9466294 1.7007622 0.4533365
H -2.3471105 0.0265780 1.2375103

H 2.1725990 -1.2356377 3.8318745
H 4.1487541 -0.5647904 5.1057819
H 3.9894933 -0.2991348 7.5712072
H 1.8848530 -0.7627827 8.7110476
H -1.8623768 -3.5966076 9.2724266
H -3.9833532 -4.6116243 9.9252875
H -6.0734493 -4.0146015 8.7389552
H -6.0338649 -2.4101627 6.9026622
H 1.2870401 -3.3158611 9.1219098
H 1.4477059 -3.5530134 11.5689726
H 0.0435696 -2.1541071 13.0433466
H -1.5226856 -0.5120190 12.0652499
H -1.7337182 -0.3128304 9.6180603
H -5.0427444 0.6394731 7.7008678
H -7.1992436 1.1372528 8.7861151
H -9.3172205 0.5295821 7.6676961
H -9.2779545 -0.5220882 5.4326194
H -7.1272822 -0.9647443 4.3137062

NiPh$_2$PhenTAA_2eOX_Triplet:
69 atoms
C -0.6619989 -3.1079893 0.8679788
C 0.5080232 -3.4405606 1.5410731
C 0.7225207 -3.0113854 2.8423105
C -0.2416941 -2.2309324 3.4723678
C -1.4387318 -1.8930313 2.7854296
C -1.6404624 -2.3415422 1.4840776
N -2.3517184 -1.2108843 3.5762655
N -0.2282420 -1.8065673 4.7924556
C 0.8700634 -1.4915292 5.5222753
C -3.2809233 -0.3242852 3.1432906
C -4.3591591 0.0758934 4.0064404
C -5.2135213 1.0773245 3.5399267
C -5.0630159 1.6662388 2.2957347
C -4.0225871 1.2693930 1.4633684
C -3.1478557 0.2962779 1.8813084
C 2.0930841 -1.1847637 4.8853713
C 3.1904044 -0.7678021 5.5988922
C 3.1050251 -0.6291433 6.9794389
C 1.9164632 -0.9181404 7.6281775
C 0.7790697 -1.3632420 6.9515916
C -4.6349488 -0.5184291 5.3117657
C -0.4049436 -1.7021144 7.7362577
N -1.5717051 -1.8695307 7.1623928
N -3.7415740 -1.2600543 5.9212162
C -3.9358256 -2.0666631 7.0710749
C -2.7475668 -2.4019699 7.7491011
C -2.7863478 -3.3140379 8.8001008
C -3.9910040 -3.8771042 9.1782308
C -5.1619984 -3.5444507 8.5128829
C -5.1396147 -2.6469166 7.4614821
Ni -1.9681457 -1.5227281 5.3607757
C -0.2658374 -1.8028415 9.2012255
C 0.6361797 -2.6972868 9.7798410
C 0.7166269 -2.8066259 11.1569306
C -0.0773715 -2.0056617 11.9650173
C -0.9579106 -1.0965086 11.3947707
C -1.0649113 -1.0034141 10.0196812
C -5.9280621 -0.2161948 5.9536575
C -5.9391659 0.3781377 7.2162102
C -7.1416044 0.6658110 7.8350321
C -8.3396888 0.3363567 7.2158465
C -8.3353255 -0.2604813 5.9632289
C -7.1359733 -0.5231386 5.3245610
H -0.8287356 -3.4766004 -0.1345454
H 1.2460837 -4.0671444 1.0600414
H 1.6040620 -3.3268915 3.3818414
H -2.5749042 -2.1412292 0.9794909
H -6.0129006 1.4181686 4.1800669
H -5.7414576 2.4500475 1.9886813
H -3.8734012 1.7480831 0.5051087
H -2.2918854 0.0502968 1.2707722
H 2.1358647 -1.2076332 3.8069543
H 4.1032920 -0.5126023 5.0783931
H 3.9530617 -0.2745223 7.5481247
H 1.8628876 -0.7790781 8.6971140
H -1.8787962 -3.6169571 9.2969405
H -4.0083418 -4.6013006 9.9807656
H -6.0977201 -4.0071701 8.7936909
H -6.0508367 -2.4347654 6.9257790
H 1.2567862 -3.3209366 9.1481965
H 1.4048480 -3.5130235 11.5997852
H -0.0041658 -2.0838541 13.0411512
H -1.5611882 -0.4574709 12.0249512
H -1.7579961 -0.3012806 9.5735182
H -5.0033835 0.6267766 7.7012404
H -7.1458812 1.1508561 8.8014917
H -9.2786973 0.5533161 7.7065859
H -9.2681521 -0.5150894 5.4792060
H -7.1314674 -0.9864770 4.3455966

NiPh$_2$PhenTAA_2eOX_Quintet:
69 atoms
C -0.6694040 -3.1952730 0.9479542
C 0.5340081 -3.4336256 1.6014710
C 0.7688778 -2.9014427 2.8564955
C -0.1969173 -2.0985287 3.4772582
C -1.4431989 -1.8635575 2.8003055
C -1.6585372 -2.4256713 1.5479836
N -2.3912566 -1.1935934 3.5480315
N -0.1294128 -1.5961783 4.7470777
C 0.9826380 -1.4311695 5.5166537
C -3.3538558 -0.3536253 3.1131645
C -4.4343499 0.0733384 3.9951376
C -5.3067878 1.0502988 3.4899452
C -5.1921027 1.5818927 2.2237763
C -4.1638234 1.1548268 1.3803256
C -3.2674569 0.2192092 1.8199011
C 2.2182271 -1.1255180 4.9186932
C 3.3287174 -0.7983184 5.6671814
C 3.2311896 -0.7460718 7.0487692
C 2.0258191 -1.0360118 7.6636832
C 0.8748425 -1.4087885 6.9546879
C -4.6995186 -0.4319647 5.3378207
C -0.3277361 -1.7209639 7.7326400
N -1.5125002 -1.8469533 7.1562154
N -3.7648829 -1.0954193 5.9977796
C -3.9088213 -1.9459820 7.0909006
C -2.6860218 -2.3658883 7.7086486
C -2.7159805 -3.3686697 8.6799815
C -3.9125211 -3.9306077 9.0653923
C -5.1062559 -3.5197125 8.4733695
C -5.1066275 -2.5489344 7.4982948
Ni -1.9003309 -1.1328942 5.4046362
C -0.2182316 -1.8175721 9.1986782
C 0.6773143 -2.7123271 9.7934520
C 0.7063131 -2.8545301 11.1688433
C -0.1251404 -2.0805557 11.9656587
C -0.9988748 -1.1711197 11.3833555
C -1.0608701 -1.0520905 10.0087674
C -5.9968451 -0.1322746 5.9647421
C -6.0204603 0.4543774 7.2330382
C -7.2260228 0.7295885 7.8488463
C -8.4198453 0.3930799 7.2234299
C -8.4060763 -0.2007241 5.9691824
C -7.2028409 -0.4479509 5.3327981
H -0.8532229 -3.6345357 -0.0227636
H 1.2825988 -4.0626224 1.1404994
H 1.6805531 -3.1463282 3.3825611
H -2.6175898 -2.3015206 1.0641126
H -6.0892877 1.4218184 4.1331471
H -5.8808971 2.3495212 1.8994116
H -4.0513098 1.5856047 0.3944544
H -2.4282429 -0.0538193 1.1972258
H 2.2645843 -1.0679397 3.8407317
H 4.2567285 -0.5482457 5.1712796
H 4.0815177 -0.4554382 7.6492733
H 1.9693795 -0.9480468 8.7371353
H -1.7941117 -3.7251156 9.1128916
H -3.9190557 -4.7095592 9.8152491
H -6.0395807 -3.9842071 8.7596900
H -6.0355264 -2.2864214 7.0190472
H 1.3230278 -3.3192320 9.1711153
H 1.3836442 -3.5665495 11.6195507
H -0.0881662 -2.1804852 13.0419139
H -1.6310508 -0.5527718 12.0057276
H -1.7483175 -0.3493332 9.5548089
H -5.0884522 0.7096405 7.7218419
H -7.2382967 1.2109472 8.8170905
H -9.3623471 0.6007954 7.7115824
H -9.3350575 -0.4656316 5.4833103
H -7.1909601 -0.9116275 4.3541449

NiPh$_2$PhenTAA_3eOX_Doublet:
69 atoms
C -0.7723243 -3.1816095 0.9939386
C 0.4303716 -3.5155163 1.6834477
C 0.6728863 -3.0723709 2.9479654
C -0.2915893 -2.2663821 3.5942762
C -1.5337621 -1.9240765 2.8843570
C -1.7323052 -2.4066630 1.5706012
N -2.3546737 -1.1514479 3.5987581
N -0.2199320 -1.7390908 4.8178197
C 0.9105363 -1.4838881 5.5620462

C -3.3356742 -0.3035220 3.1312015
C -4.3946251 0.1320978 3.9926429
C -5.2396140 1.1439771 3.5101963
C -5.1107473 1.6797025 2.2412202
C -4.0991140 1.2283608 1.4082089
C -3.2179149 0.2613713 1.8560390
C 2.1277587 -1.2279987 4.9198252
C 3.2488592 -0.8333239 5.6251332
C 3.1755637 -0.6549264 6.9988542
C 1.9810212 -0.8886539 7.6536217
C 0.8267945 -1.3300834 6.9861120
C -4.6611840 -0.4101853 5.3156786
C -0.3536575 -1.6182059 7.7815128
N -1.5542435 -1.6992059 7.2044528
N -3.7178193 -1.0890179 5.9683922
C -3.9023486 -1.9789837 7.0158473
C -2.6830825 -2.3206348 7.7130734
C -2.7132558 -3.3077448 8.7062486
C -3.8992432 -3.9261859 9.0146469
C -5.0854011 -3.5929298 8.3363953
C -5.0897777 -2.6406532 7.3464597
Ni -1.9294776 -1.2747237 5.4122153
C -0.2250179 -1.7602121 9.2270665
C 0.7627407 -2.5903175 9.7855773
C 0.8345103 -2.7536970 11.1536058
C -0.0345447 -2.0525692 11.9847432
C -0.9955423 -1.2002962 11.4426714
C -1.1091096 -1.0710951 10.0768096
C -5.9502492 -0.1512993 5.9452457
C -5.9982292 0.3009283 7.2761146
C -7.2111773 0.5520586 7.8766049
C -8.3961882 0.3136579 7.1814955
C -8.3623477 -0.1521399 5.8703540
C -7.1509157 -0.3576672 5.2430225
H -0.9418994 -3.6004424 0.0107659
H 1.1378802 -4.1794758 1.2047061
H 1.5551059 -3.3891473 3.4855689
H -2.6694353 -2.2185061 1.0665169
H -5.9977325 1.5454804 4.1651396
H -5.7766779 2.4680891 1.9189586
H -3.9634249 1.6586104 0.4248066
H -2.3737567 -0.0100353 1.2389919
H 2.1695898 -1.2683784 3.8413166
H 4.1669090 -0.6208305 5.0932512
H 4.0337094 -0.3023302 7.5535452
H 1.9254715 -0.6904505 8.7129883
H -1.8037546 -3.6197236 9.1944291
H -3.9130647 -4.7132416 9.7566995
H -5.9985086 -4.1257441 8.5663478
H -5.9984580 -2.4409622 6.8010039
H 1.4311439 -3.1411364 9.1360243
H 1.5682151 -3.4246605 11.5785486
H 0.0414848 -2.1642033 13.0583252
H -1.6437460 -0.6326404 12.0959312
H -1.8452190 -0.4000587 9.6524293
H -5.0754740 0.4992493 7.8070665
H -7.2467477 0.9455623 8.8832100
H -9.3479515 0.4998030 7.6616693
H -9.2843801 -0.3502010 5.3409928
H -7.1202324 -0.7284627 4.2262676

NiPh$_2$PhenTAA_3eOX_Quartet:
69 atoms
C -0.7179776 -3.1816665 0.9353998
C 0.4525122 -3.5120893 1.6098569
C 0.6827809 -3.0491043 2.8990827
C -0.2637422 -2.2332924 3.5129749
C -1.4644723 -1.8975319 2.8236191
C -1.6832242 -2.3840449 1.5377992
N -2.3596445 -1.1761103 3.5958370
N -0.2287232 -1.7691539 4.8166234
C 0.8822958 -1.4883306 5.5455017
C -3.3061225 -0.3144284 3.1434998
C -4.3851326 0.1107896 4.0021651
C -5.2286654 1.1215700 3.5201576
C -5.0914910 1.6696183 2.2561516
C -4.0664865 1.2333792 1.4247971
C -3.1854305 0.2701027 1.8663452
C 2.1077788 -1.2177731 4.9032951
C 3.2235225 -0.8220959 5.6091381
C 3.1502900 -0.6537011 6.9867959
C 1.9565449 -0.8987499 7.6434980
C 0.8055954 -1.3456398 6.9793623
C -4.6566781 -0.4425341 5.3140975
C -0.3701077 -1.6450606 7.7714617
N -1.5650117 -1.7551886 7.1894966
N -3.7236104 -1.1492538 5.9543190
C -3.9179225 -2.0134630 7.0253400
C -2.6997259 -2.3564925 7.7207331
C -2.7322685 -3.3254850 8.7275790
C -3.9252800 -3.9262169 9.0541558
C -5.1109128 -3.5904138 8.3797819
C -5.1108912 -2.6527792 7.3737393
Ni -1.9524292 -1.3942017 5.3928395
C -0.2486954 -1.7621850 9.2220307
C 0.7314186 -2.5884346 9.7981187
C 0.7959529 -2.7297001 11.1690655
C -0.0737199 -2.0106439 11.9845206
C -1.0274098 -1.1623529 11.4237629
C -1.1327352 -1.0539700 10.0551387
C -5.9367936 -0.1645600 5.9588515
C -5.9628060 0.3028035 7.2848295
C -7.1659798 0.5657800 7.9005759
C -8.3618839 0.3257741 7.2252715
C -8.3488336 -0.1533902 5.9181308
C -7.1474942 -0.3717514 5.2756334
H -0.8980834 -3.5768947 -0.0550196
H 1.1773990 -4.1644584 1.1425700
H 1.5626695 -3.3679416 3.4400025
H -2.6194704 -2.1913384 1.0330259
H -5.9974791 1.5122355 4.1693517
H -5.7595376 2.4573249 1.9360634
H -3.9247011 1.6797947 0.4494632
H -2.3324698 0.0122602 1.2560485
H 2.1459906 -1.2454355 3.8245245
H 4.1397109 -0.5975602 5.0791561
H 4.0071445 -0.3003826 7.5432903
H 1.9046110 -0.7086440 8.7046788
H -1.8228050 -3.6382025 9.2158553
H -3.9422671 -4.7003422 9.8095581
H -6.0285744 -4.1086813 8.6235053
H -6.0201454 -2.4517813 6.8293753
H 1.3998978 -3.1532184 9.1606193
H 1.5240640 -3.3975298 11.6083008
H -0.0035636 -2.1050041 13.0601379
H -1.6761881 -0.5810988 12.0643794
H -1.8631714 -0.3860452 9.6160611
H -5.0314710 0.5012716 7.8007217
H -7.1845495 0.9691690 8.9037629
H -9.3059179 0.5208266 7.7169281
H -9.2792218 -0.3525620 5.4040612
H -7.1332608 -0.7527890 4.2622047

NiPh$_2$PhenTAA_3eOX_Sextet:
69 atoms
C -0.6982411 -3.1686339 0.9107542
C 0.4728533 -3.4981892 1.5844618
C 0.7023987 -3.0282133 2.8703024
C -0.2291459 -2.1968649 3.4937642
C -1.4450400 -1.8557437 2.7958372
C -1.6571560 -2.3660168 1.5139252
N -2.3617048 -1.1409203 3.5326842
N -0.1606872 -1.7601538 4.7964708
C 0.9362980 -1.5127771 5.5505239
C -3.3410423 -0.3157410 3.0944563
C -4.4322817 0.0882185 3.9708817
C -5.2869594 1.0860054 3.4792049
C -5.1674383 1.6366453 2.2132111
C -4.1415917 1.2203694 1.3727281
C -3.2432590 0.2765427 1.8148712
C 2.1744935 -1.2367646 4.9266619
C 3.2868182 -0.8548961 5.6408762
C 3.1972159 -0.7018471 7.0194372
C 1.9956351 -0.9520975 7.6632671
C 0.8409279 -1.3895671 6.9988393
C -4.7207187 -0.4262878 5.3102729
C -0.3340007 -1.6603158 7.8284320
N -1.5485328 -1.7116077 7.2819446
N -3.7848688 -1.0808680 5.9994747
C -3.9364000 -1.9270920 7.0782409
C -2.7019963 -2.2764305 7.7845703
C -2.7513119 -3.2641754 8.7803511
C -3.9385154 -3.8705915 9.0999174
C -5.1257938 -3.5325493 8.4233213
C -5.1229002 -2.5903584 7.4274814
Ni -1.9290410 -1.2666827 5.4204605
C -0.1730808 -1.7909765 9.2739309
C 0.8034562 -2.6429749 9.8195874
C 0.8891602 -2.8105720 11.1864095
C 0.0463105 -2.0929052 12.0300071
C -0.9039941 -1.2204035 11.5009376
C -1.0306818 -1.0861023 10.1366320

C -6.0171697 -0.1429258 5.9193975
C -6.0751379 0.3442329 7.2371354
C -7.2921485 0.5976157 7.8288049
C -8.4727898 0.3280525 7.1375150
C -8.4296580 -0.1733592 5.8397461
C -7.2142193 -0.3821248 5.2211164
H -0.8792673 -3.5617264 -0.0804019
H 1.2000451 -4.1481485 1.1172113
H 1.5825547 -3.3480388 3.4108339
H -2.5939897 -2.1747869 1.0090004
H -6.0595466 1.4714843 4.1261516
H -5.8537610 2.4115632 1.8998819
H -4.0180066 1.6647343 0.3940011
H -2.3859571 0.0318682 1.2042068
H 2.2165536 -1.2536134 3.8471879
H 4.2104185 -0.6326783 5.1230787
H 4.0482552 -0.3574207 7.5905623
H 1.9439858 -0.7691939 8.7252982
H -1.8435311 -3.5786019 9.2708300
H -3.9562034 -4.6461973 9.8538299
H -6.0440895 -4.0507455 8.6649204
H -6.0322422 -2.3891226 6.8831849
H 1.4518721 -3.2071217 9.1611293
H 1.6137407 -3.4978134 11.6010024
H 0.1339944 -2.2076068 13.1023518
H -1.5338224 -0.6421605 12.1628624
H -1.7605697 -0.4016289 9.7228492
H -5.1563420 0.5631944 7.7667758
H -7.3342344 1.0152244 8.8254127
H -9.4278328 0.5163368 7.6101672
H -9.3479215 -0.3965866 5.3139955
H -7.1771636 -0.7801665 4.2148304

References in the supporting information

(1) Fulmer, G. R.; Miller, A. J. M.; Sherden, N. H.; Gottlieb, H. E.; Nudelman, A.; Stoltz, B. M.; Bercaw, J. E.; Goldberg, K. I. NMR Chemical Shifts of Trace Impurities: Common Laboratory Solvents, Organics, and Gases in Deuterated Solvents Relevant to the Organometallic Chemist. *Organometallics* **2010**, *29* (9), 2176–2179. DOI: 10.1021/om100106e

(2) Stoll, S.; Schweiger, A. EasySpin, a Comprehensive Software Package for Spectral Simulation and Analysis in EPR. *J. Magn. Reson.* **2006**, *178* (1), 42–55. DOI: 10.1016/j.jmr.2005.08.013.

(3) *cwEPR – MATLAB Central File Exchange*. https://www.mathworks.com/matlabcentral/fileexchange/73292–cwepr (accessed 2023-09-17)

(4) Krejčik, M.; Daněl, M.; Hartl, F. Simple construction of an infrared optically transparent thin-layer electrochemical cell: Applications to the redox reactions of ferrocene, $Mn_2(CO)_{10}$ and $Mn(CO)_3$(3,5-di-t-butyl-catecholate). *J. Electroanal. Chem*. **1991**, 317, 179–187. DOI: 10.1016/0022-0728(91)85012-E.

(5) Bruker, SAINT V8.40B, Bruker AXS Inc., Madison, Wisconsin, USA, **2001**.

(6) Krause, L., Herbst-Irmer, R., Sheldrick G.M. & Stalke D., SADABS-2016/2 - Bruker AXS area detector scaling and absorption correction. J. Appl. Cryst. 48 (2015) 3–10. DOI: 10.1107/S1600576714022985

(7) Sheldrick, G. M. SHELXT – Integrated Space–Group and Crystal–Structure Determination. *Acta Crystallogr. Sect. A Found. Crystallogr.* **2015**, *71* (1), 3–8. DOI: 10.1107/S2053273314026370

(8) Sheldrick, G.M. Crystal structure refinement with SHELXL. Acta Crystallogr. Sect. C Struct. Chem. 2015, C71, 3–8. DOI: 10.1107/S2053229614024218

(9) *THE PLATON HOMEPAGE.* http://www.platonsoft.nl/platon/pl000000.html (accessed 2023-09-17).

(10) TURBOMOLE *Version 7.4.1*; TURBOMOLE Gmbh, Karlsruhe, Germany, **2019.**

(11) (a) *PQS Version 2.4*; Parallel Quantum Solutions, Fayetteville, AR, USA, **2001**. (b) Baker, J. An Algorithm for the Location of Transition States. *J. Comput. Chem.* **1986**, *7* (4), 385–395. DOI: 10.1002/jcc.540070402

(12) Budzelaar, P. H. M. Geometry optimization using generalized, chemically meaningful constraints. *J. Comput. Chem.* **2007**, *28*, 2226–2236. DOI: 10.1002/jcc.20740

(13) Grimme, S.; Antony, J.; Ehrlich, S.; Krieg, H. A Consistent and Accurate Ab Initio Parametrization of Density Functional Dispersion Correction (DFT–D) for the 94 Elements H–Pu. *J. Chem. Phys.* **2010**, *132,* 154101-1–154101-19. DOI: 10.1063/1.3382344

(14) Sierka, M.; Hogekamp, A.; Ahlrichs, R. Fast evaluation of the Coulomb potential for electron densities using multipole accelerated resolution of identity approximation. *J. Chem. Phys.* **2003**, *118*, 9136–9148. DOI: 10.1063/1.1567253.

(15) (a) Becke, A. D. Density–functional exchange–energy approximation with correct asymptotic behaviour. *Phys. Rev. A.* **1988**, *38*, 3098–3110. DOI: 10.1103/PhysRevA.38.3098 (b) Perdew, J. P. Density–functional approximation for the correlation energy of the inhomogeneous electron gas. *Phys. Rev. B.* **1986**, *33*, 8822–8824. DOI: 10.1103/PhysRevB.33.8822 (c) Perdew, J. P. Erratum: Density–functional approximation for the correlation energy of the inhomogeneous electron gas. *Phys. Rev. B.* **1986**, *34*, 7406–7406. DOI: 10.1103/PhysRevB.33.8822

(16) (a) Weigend, F. & Ahlrichs, R. Balanced Basis Sets of Split Valence, Triple Zeta Valence and Quadruple Zeta Valence Quality for H to Rn: Design and Assessment of Accuracy. *Phys. Chem. Chem. Phys.* **2005**, *7*, 3297–3305. DOI: 10.1039/B508541A (b) Weigend, F., Häser, M., Patzelt, H. & Ahlrichs, R. RI–MP2: Optimized Auxiliary Basis Sets and Demonstration of Efficiency. *Chem. Phys. Lett.* **1998**, *294*, 143–152. DOI: 10.1016/S0009-2614(98)00862-8.

(17) Ferré, N.; Guihéry, N.; Malrieu, J. P. Spin Decontamination of Broken–Symmetry Density Functional Theory Calculations: Deeper Insight and New Formulations. *Phys. Chem. Chem. Phys.* **2015**, *17* (22), 14375–14382. DOI: 10.1039/C4CP05531D

(18) Neese, F. The ORCA Program System. *Wiley Interdiscip. Rev. Comput. Mol. Sci.* **2012**, *2* (1), 73–78. DOI: 10.1002/wcms.81

(19) (a) Axel, D. B. Density–Functional Thermochemistry. III. The Role of Exact Exchange. *J. Chem. Phys.* **1993**, *98* (7), 5648–5652. DOI: 10.1063/1.464913 (b) Lee, C. Yang, W. Parr, R. G. Development of the Colle–Salvetti correlation–energy formula into a functional of the electron density. *Phys. Rev. B* **1988**, *37*, 785–789. DOI: 10.1103/PhysRevB.37.785

(20) (a) Chang, C.; Pelissier, M.; Durand, P. Regular Two–Component Pauli–like Effective Hamiltonians in Dirac Theory. *Phys. Scr.* **1986**, *34* (5), 394–404. DOI: 10.1088/0031-8949/34/5/007 (b) Heully, J. L.; Lindgren, I.; Lindroth, E.; Lundqvist, S.; Martensson–Pendrill, A. M. Diagonalisation of the Dirac Hamiltonian as a Basis for a Relativistic Many–Body Procedure. *J. Phys. B At. Mol. Phys.* **1986**, *19* (18), 2799–2815. DOI: 10.1088/0022-3700/19/18/011 (c) Van Lenthe, E.; Van Leeuwen, R.; Baerends, E. J.; Snijders, J. G. Relativistic Regular Two–Component Hamiltonians. *J. Chem. Phys.* **1993**, *99,* 4597–4610. DOI: 10.1063/1.466059

(21) Available at www.iboview.org. (a) Knizia, G. Intrinsic Atomic Orbitals: An Unbiased Bridge between Quantum Theory and Chemical Concepts. *J. Chem. Theory Comput.* **2013**, *9* (11), 4834–4843. DOI: 10.1021/ct400687b (b) Knizia, G.; Klein, J. E. M. N. Electron Flow in Reaction Mechanisms – Revealed from First Principles. *Angew. Chemie – Int. Ed.* **2015**, *54* (18), 5518–5522. DOI: 10.1002/anie.201410637

(22) Hirata, S.; Head-Gordon, M. Time-dependent density functional theory within the Tamm–Dancoff approximation. *Chem. Phys. Lett.* **1999**, *314*, 291– 299. DOI: 10.1016/S0009-2614(99)01149-5.

(23) Pang, X.; Lou, Z.; Li, M.; Wen, L.; Chen, C. Tandem Arylation/Friedel–Crafts Reactions of o-Acylanilines with Diaryliodonium Salts: A Modular Synthesis of Acridine Derivatives. *Eur. J. Org. Chem.* **2015**, *15*, 3361–3369. DOI: 10.1002/ejoc.201500161.

(24) Liao, M.S.; Scheiner, S. Electronic structure and bonding in metal porphyrins, metal=Fe, Co, Ni, Cu, Zn. *J. Chem. Phys.* **2002,** *117* (205). DOI: 10.1063/1.1480872.
